# Supplementary material for: 5-Hydroxytryptophan (5-HTP): Natural Occurrence, Analysis, Biosynthesis, Biotechnology, Physiology and Toxicology
Source: Int J Mol Sci. 2020 Dec 26;22(1):181. doi: 10.3390/ijms22010181 (PMC7796270; doi:10.3390/ijms22010181)
Supplement: Supplementary file 1 [file ijms-22-00181-s001.zip › 5-HTP.Data/PDF/0025077987/Wu-1996-Triplet-state-properties-of--azatry.pdf]

Triplet State Properties of 7-Azatryptophan and 5-Hydroxytryptophan: Analysis of Delayed "Slow-Passage" ODMR Responses

Jie Q. Wu, Andrzej Ozarowski, Sara K. Davis, and August H. Maki*

Department of Chemistry, University of California at Davis, Davis, California 95616

Received: February 29, 1996; In Final Form: May 6, 1996⊗

The triplet state properties of 7-azatryptophan (7AW), 5-hydroxytryptophan (5HW), and the 5HW anion (pH = 12.3) have been investigated by optically detected magnetic resonance, ODMR, in zero applied field. Also the triplet state properties of tryptophan (Trp) have been reexamined. Analysis of "slow-passage" ODMR responses during phosphorescence decay (delayed ODMR) that contain the effects of fast passage is introduced using Marquardt−Levenberg nonlinear least-squares fitting. The parameters determined by the analysis of delayed ODMR are the band center frequency (ν_0) and bandwidth ($\nu_{1/2}$) of the inhomogeneously broadened ODMR band, the apparent decay constants of the triplet sublevels, and their relative radiative rate constants. The values of ν_0 and $\nu_{1/2}$ are obtained with greater accuracy than is possible with analysis that does not account for fast passage effects on the band shape. The kinetic parameters are influenced somewhat by residual spin−lattice relaxation (SLR) effects, however. The zero-field magnetic resonance frequencies and band widths have been determined for Trp, 7AW, 5HW, and 5HW anion, along with the apparent kinetic parameters for Trp and 7AW. Microwave-induced delayed phosphorescence transient responses of 5HW and its anion are analyzed globally to give kinetic parameters free of residual effects. The ODMR band widths of 5HW and its anion greatly exceed those of Trp, while those of 7AW are significantly narrower. The zero-field-splitting (ZFS) parameter *E* is reduced in 5HW and its anion to about half its value in Trp, while the *E*-parameter of 7AW increases by about 10%. Sublevel decay constants relative to Trp are hardly changed in 5HW, but increase significantly in the anion. Decay constants of Trp increase moderately with 7-aza substitution, as expected from introduction of spin−orbit coupling at the N atom. The pattern of ODMR bandwidths of Trp and 7AW is consistent with a positive correlation of solvent induced shifts in the ZFS *D*- and *E*-parameters.

Downloaded via UNIV DEGLI STUDI DI TORINO on November 23, 2020 at 07:13:12 (UTC).
See https://pubs.acs.org/sharingguidelines for options on how to legitimately share published articles.

Introduction

The amino acid tryptophan (Trp) has been employed extensively as an intrinsic fluorescence probe of protein structure and function.[1,2] Shortly after the introduction of optically detected magnetic resonance (ODMR) of photoexcited triplet states,[3,4] this method began to be applied to study the phosphorescent state of Trp in proteins.[5,6] A great deal of information concerning protein structure and their interactions has been uncovered from ODMR measurements made on intrinsic Trp residues.[7]

Much interest has been focused recently on tryptophan analogs whose photophysical properties differ in significant ways from those of Trp. Negrerie et al.[8] have pointed out that the red-shifted absorbence edge of 7-azatryptophan (7AW) allows its selective photoexcitation in the presence of a large excess of Trp. Furthermore, in contrast with Trp, its fluorescence decay in protic solvents such as water and alcohols is very close to being a single exponential, even when incorporated into a small peptide,[9] and its short lifetime in such media allows its decay to be resolved from that of Trp. Another Trp analog that has proven of interest is 5-hydroxytryptophan (5HW), which is similar to 7AW, having a red-shifted absorption edge relative to Trp and thus being amenable to selective photoexcitation.[9,10] Biosynthetic incorporation of 7AW and 5HW into proteins has been accomplished already in early experiments,[11−14] and "bait-and-switch" methodology using Trp auxotrophs of *Escherichia coli* has been employed more recently[15−17] to produce useful quantities of proteins containing these analogs in place of Trp. The ability to selectively excite the 7AW and 5HW chromophores when substituted into or bound to proteins in the presence of excess Trp provides an important spectroscopic advantage.[8−10,16,17] The extreme sensitivity of the singlet excited state decay processes of 7AW to the local environment that have been revealed by recent measurements[9,10,18] suggests its value as a spectroscopic reporter of its local environment.

The triplet state properties of 7AW and 5HW have received less attention than have those of the excited singlet state. In aprotic solvents 7-azaindole forms cyclic internally hydrogen bonded dimers that undergo efficient internal double proton tautomerization in the excited singlet state.[19,20] The triplet yield from the tautomer state is low, and most triplet states apparently originate from oligomers and possibly also from noncyclized dimers.[21,22] In protic solvents such as water and alcohols, dimerization and oligomer formation are suppressed; the triplet state is formed at low temperatures by intersystem crossing from monomers that are hydrogen bonded to solvent molecules.[22] At elevated temperatures tautomerization involving solvent protons occurs, leading to quenching of the fluorescence and a relatively short 7AW fluorescence lifetime of ca. 770 ps in H_2O,[9,10] which is reduced to 140 ps in methanol,[9] in which tautomerization apparently occurs more rapidly. The phosphorescence spectrum of 7-azaindole exhibits vibronic structure similar to that of indole; its 0,0-band measured in ethanol glass peaks at 426 nm, and its phosphorescence lifetime is ca. 2 s.[22] The phosphorescent state of 5HW has been studied recently in an aqueous buffer (pH 7.5)−glycerol glass at 77 K.[23] Its structured phosphorescence resembles that of Trp (although red-shifted) with a poorly resolved 0,0-band shoulder located at ca. 415 nm and a single-exponential lifetime of 4.9 s.

* To whom correspondence should be addressed.
⊗ Abstract published in *Advance ACS Abstracts,* June 15, 1996.

 © 1996 American Chemical Society

To provide a basis for subsequent ODMR measurements on 7AW and 5HW residues incorporated into and bound to proteins, we report in this communication the results of our phosphorescence and ODMR measurements on the amino acids themselves. We report our results on both neutral amino acids measured at ca. pH 7 and the 5HW anion at high pH, where the hydroxyl proton at position 5 is ionized. These data are compared with those obtained for Trp which we have reexamined in this work. In the evaluation of slow-passage ODMR spectra we have developed a method of analysis[24] recently that accounts for transient effects[25] on the ODMR signals that result from microwave rapid passage during continuous optical pumping. This method is extended in this communication to the analysis of slow-passage ODMR spectra that are obtained during the decay of the phosphorescent state. The advantages of delayed ODMR measurements have been recognized since they were initially employed.[26] Such spectra normally have improved signal-to-noise ratios relative to those obtained during continuous optical pumping because of the absence of fluorescence, reduced background phosphorescence, and increased spin alignment. In addition, the sublevel decay constants obtained in the analysis are devoid of any influence of optical pumping kinetics. An algorithm for computer analysis of slow-passage ODMR spectra obtained during decay of the phosphorescence is given in the Appendix of this communication.

Experimental Part

L-Tryptophan (>99.5%) was obtained from Fluka, while L-5HW and D,L-7AW hydrates were from Sigma. They were used as received. The amino acids were dissolved in 0.01 M pH 7 phosphate buffer containing 0.1 M NaCl and 10^{-4} M EDTA. Ethylene glycol (30% v/v for 7AW; 50% v/v for Trp and 5HW) was added as cryosolvent. To make the 5HW anion solution, ca. 10^{-2} M NaOH was substituted for the phosphate buffer. The solvent was deoxygenated with Ar gas prior to adding the solid 5HW, and precautions were taken to exclude air from the sample. The final amino acid concentrations were ca. 1 mM.

The sample, contained in a 1 mm i.d. Suprasil tube, was held in a copper helical slow-wave structure that terminated a stainless steel coaxial transmission line that was inserted into a dewar for phosphorescence measurements at 77 K or ODMR measurements at 1.2 K. All ODMR measurements were made in the absence of an applied steady magnetic field. Details of the ODMR spectrometer have been described recently.[24] Phosphorescence and some of the slow-passage ODMR measurements during optical pumping were made using a rotating sector to eliminate fluorescence. The zero-field splittings (ZFS) and ODMR linewidths of 5HW and 5HW anion were estimated from these slow-passage measurements. Microwave-induced delayed phosphorescence (MIDP) measurements[27] were made to obtain sublevel decay constants of 5HW and 5HW anion.

For Trp and 7AW, the sublevel kinetic parameters, relative radiative rate constants, ZFS, and ODMR linewidths were obtained by analyzing the ODMR slow-passage responses during decay of the phosphorescence as described in the Appendix. In these delayed slow-passage measurements, the sample was optically pumped at 1.2 K long enough to effectively achieve photostationary state sublevel populations, N_j^0, $j = x, y, z$. At $t = 0$ the excitation was shuttered and the sample was allowed to decay for a time t_0, when a slow microwave sweep through one of the ODMR transitions and data collection were begun. The decay was monitored for a time sufficient to collect data on the slowest components of the response. The experiment was repeated several times, and the signals were averaged by

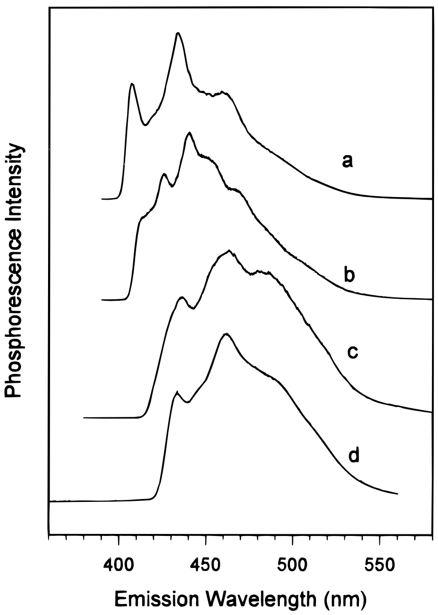

Figure 1. Phosphorescence spectra of (a) Trp, pH = 7, (b) 5HW, pH = 7, (c) 5HW, pH = 12.3, and (d) 7AW, pH = 7 in EG-buffer at 77 K. Samples are excited at 302 nm for Trp and 5HW and 296 nm for 7AW. The concentration of each sample is about 10^{-3} M.

computer. A number of decays without microwave sweep were accumulated and subtracted to obtain the response to be analyzed. The amplitude of this decay was adjusted so that subtraction yielded a zero base line at times prior to the microwave sweep. The measurement of an ODMR transition was repeated several times using different microwave sweep rates and delay times, t_0, and the results of the data analyses were averaged by computer. Each data set consisted of 1024 data points separated by equal time intervals. The resulting data sets were each fitted to eq A5 assuming a Gaussian line shape, g_n, (eq A3) using a Marquardt−Levenberg nonlinear least-squares (χ^2) minimization procedure. In this procedure, there are five unknown parameters to be determined: the band center frequency (ν_0), the half-width at half-height of the Gaussian band shape ($\nu_{1/2}$), the apparent decay constants of the sublevels undergoing resonance (k_i, k_j), and the ratio of their radiative rate constants (R_{ji}). The program was written in C language and run on a 586 PC.

Relative sublevel quantum yields and N_i^0 were obtained by means of the Shain−Sharnoff microwave desaturation response experiment.[28] The latter were used to calculate the N_{ij}^0 (see Appendix), which then were used as input parameters in the analysis of the delayed slow-passage responses.

Results

Phosphorescence and Conventional Slow-Passage ODMR. The phosphorescence spectra of Trp, 7AW, 5HW, and 5HW anion are shown in Figure 1. Results of the analysis of phosphorescence decay kinetics at 77 K are presented in Table 1. Conventional slow-passage ODMR measurements in the presence of continuous optical pumping were carried out on each of these samples at 1.2 K. The spectra are shown in Figure 2. The Trp (Figure 2a) and 7AW (Figure 2d) phosphorescences were accompanied by intense overlapping fluorescence; these spectra were measured using a rotating sector to eliminate this interference. The high-frequency "noise" in these spectra results from incomplete signal averaging of the sector modulation. On the other hand, the emissions of 5HW (Figure 2b) and its anion (Figure 2c) at the monitored wavelengths consist mostly of phosphorescence, so a sector was not used in these measurements. As has been noted previously,[7] the Trp ODMR spectrum

TABLE 1: Phosphorescence 0,0-Band Peak Wavelength and Decay Analysis of Tryptophan and Analogs[a]

sample	$\lambda_{0,0}$ (nm)	lifetime[b] (s)
Trp	406.7	6.8(100)
5HW, pH 7	412.0	5.2(100)
5HW, pH 12.3	436.0	2.2(50), 1.4(49)
7AW	432.5	3.2(100)

[a] Uncertainties in $\lambda_{0,0}$ and lifetimes are ±0.3 nm and ±0.1 s, respectively. T = 77 K. [b] Initial amplitudes, in percent, are in parentheses.

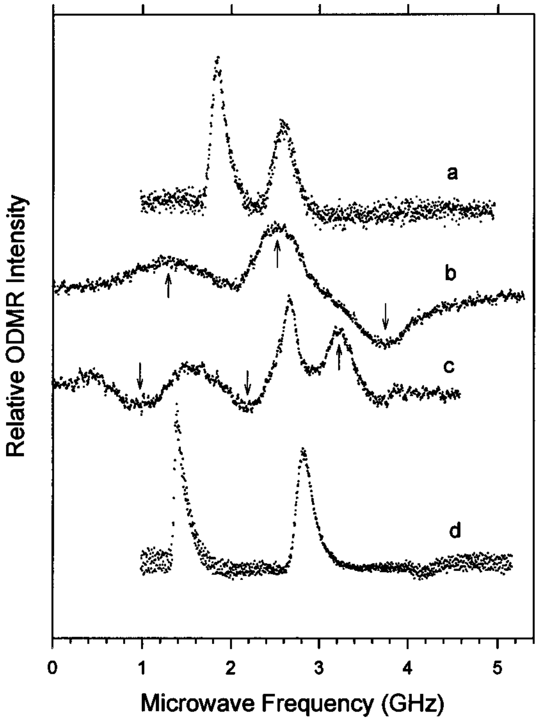

Figure 2. Phosphorescence-detected slow-passage ODMR signals of (a) Trp, pH = 7, (b) 5HW, pH = 7, (c) 5HW, pH = 12.3, and (d) 7AW, pH = 7 in EG-buffer at 1.2 K. The phosphorescence is monitored at (a) 406.7, (b) 425.0, (c) 436.0, and (d) 432.5 nm using a 3 nm band pass. The microwave frequency was swept at (a) 50, (b) 110, (c) 110, and (d) 100 MHz/s, and signal averaging (40−60 accumulations) was carried out to improve the signal/noise.

reveals only two transitions; the highest frequency $D + E$ transition is of vanishing intensity. In contrast, the slow-passage ODMR spectra of 5HW, 5HW anion, and 7AW contain all three expected transitions each. These are most clearly resolved for 7AW (although the high-frequency transition at ca. 4.1 GHz is relatively weak). On the other hand, the ODMR bands of 5HW and its anion are very broad and overlapping. Our assignments of the band centers of 5HW and its anion are shown by the vertical arrows in Figure 2. The relatively narrow positive polarity (i.e., increase in phosphorescence intensity) peak at ca. 2.7 GHz in the 5HW anion spectrum is not assigned. It may originate from a photoproduct or byproduct of the deprotonation of 5HW, although precautions were taken to exclude oxygen. It should be noted that the ν_1 and ν_2 transitions of 5HW are of positive polarity, while the ν_3 transition has negative polarity. The opposite pattern occurs for the 5HW anion. Here, and in the remainder of this communication, the ODMR transitions are numbered in order of increasing frequency. The ODMR band center frequencies and bandwidths of 5HW and its anion were estimated from the spectra in Figure 2 using a Gaussian fitting routine that neglects fast-passage effects, while those of Trp and 7AW were obtained from analysis of delayed slow-passage measurements discussed in the following section.

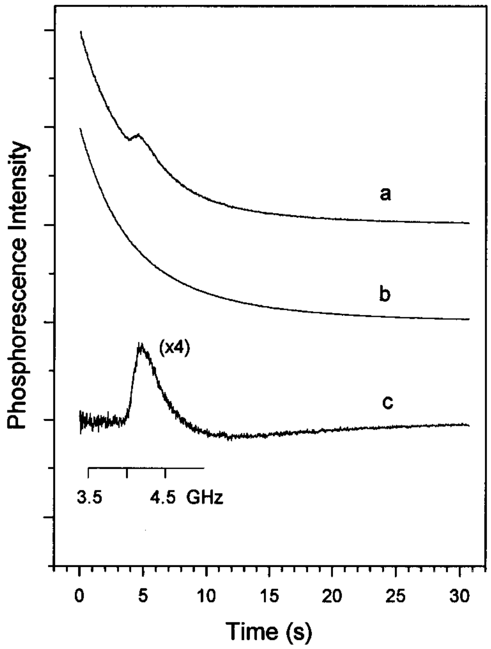

Figure 3. Delayed slow-passage response (a) and the normal phosphorescence decay (b) of 7AW at 1.2 K. Subtracting b from a results in the ODMR signal as shown in part c. The microwave frequency, scale given in the figure, was swept at 160 MHz/s through the $|D| + |E|$ resonance only.

Delayed Slow-Passage ODMR of Trp and 7AW. The analysis of delayed slow-passage ODMR measurements using the algorithm (eq A5) developed in the Appendix requires that the inhomogeneously broadened ODMR bands are well resolved from one another. This is not the case for 5HW and its anion (Figure 2b,c); consequently delayed slow-passage measurements were limited to Trp and to 7AW, whose ODMR spectra are well resolved (Figures 2a,d). A typical delayed slow-passage measurement is illustrated in Figure 3. Delayed slow-passage ODMR spectra of the ν_1 and ν_3 transitions of 7AW obtained over a range of microwave sweep rates and variable delay times, t_0, are shown in Figures 4 and 5, respectively. A similar set of measurements for the ν_1 and ν_3 transitions of Trp are shown in Figures 6 and 7, respectively. (Although the ν_3 transition of Trp is too weak to be observed during optical pumping because $N_{yz}^0 \approx 1$, the delayed slow-passage signal is sufficiently prominent to be analyzed because spin alignment is produced during the decay of the triplet state.) The predicted responses based on least-squares minimization of the data sets with respect to eq A5 are superimposed on the individual data points in Figures 4−7. A Gaussian band shape for the inhomogeneous distribution was assumed for all transitions. The predicted response lies inside the random noise pattern in each case, and it is clear that there are no obvious systematic variations between the computed response and the experimental data points. The results of the analyses are summarized for each of the transitions in Tables 2−5. It is assumed (see below) that the sublevel energies of 7AW are arranged in the same order as they are known to be[7] for Trp, i.e., $E_y > E_x > E_z$. Tables 2−5 list the best-fit parameters for each data set, the mean value of each parameter, and the standard deviation, σ. The standard error of the mean is approximately $\sigma/\sqrt{n}$, where n is the number of spectra in the data set. Delayed measurements of the ν_2 transition of Trp and 7AW were carried out as well, but the details are omitted. The mean values of ν_0 and $\nu_{1/2}$ obtained from analysis of the delayed slow passage responses of Trp and 7AW are summarized in Table 6. The parameters for Trp may be compared with previously reported slow-passage data.

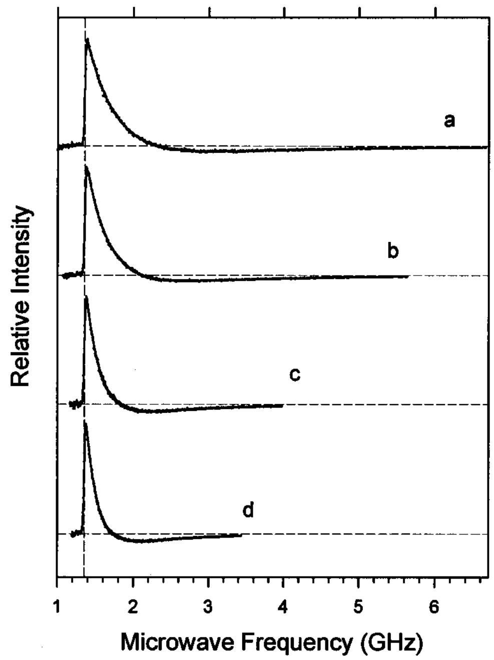

Figure 4. Delayed slow-passage ODMR spectra of the $|D| - |E|$ transition of 7AW in EG-buffer. The microwave sweep rate is (a) 200, (b) 160, (c) 100, and (d) 80 MHz/s, and the microwave output is limited by a 2 GHz low-pass filter. The superimposed solid lines represent the calculated best fit of each experimental spectrum; the values of the fitting parameters are given in Table 2. The dashed vertical line indicates the mean value of ν_0 (1.354 GHz) defined in the text.

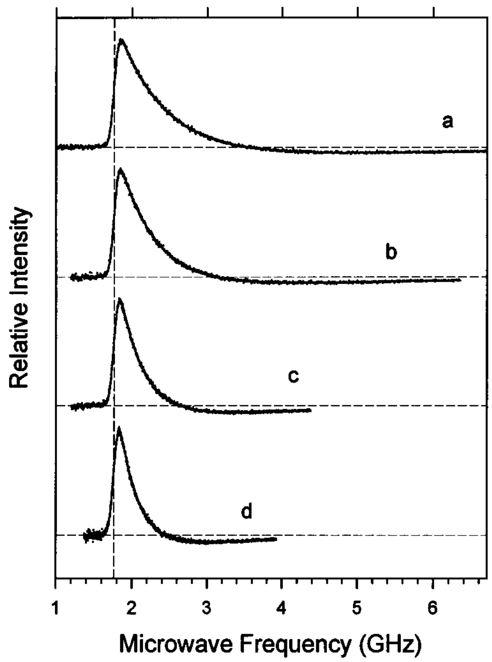

Figure 6. Delayed slow-passage ODMR spectra of the $|D| - |E|$ transition of Trp in EG-buffer. The microwave sweep rate is (a) 200, (b) 160, (c) 100, and (d) 80 MHz/s, and the microwave output is limited by a 2 GHz low-pass filter. The superimposed solid lines represent the calculated best fit of each experimental spectrum; the values of the fitting parameters are given in Table 4. The dashed vertical line indicates the mean value of ν_0 (1.763 GHz) defined in the text.

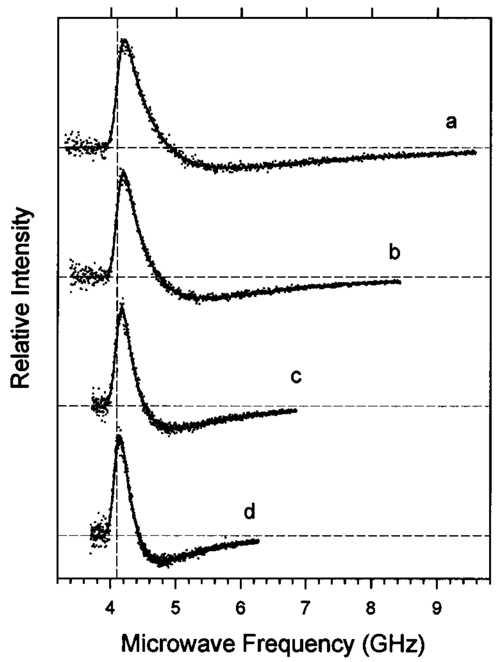

Figure 5. Delayed slow-passage ODMR spectra of the $|D| + |E|$ transition of 7AW in EG-buffer. The microwave sweep rate is (a) 200, (b) 160, (c) 100, and (d) 80 MHz/s, and the microwave sweep ranges from 3.8 to 4.6 GHz and is limited by a 5 GHz low-pass filter. The superimposed solid lines represent the calculated best fit of each experimental spectrum; the values of the fitting parameters are listed in Table 3. The dashed vertical line indicates the mean value of ν_0 (4.097 GHz) defined in the text.

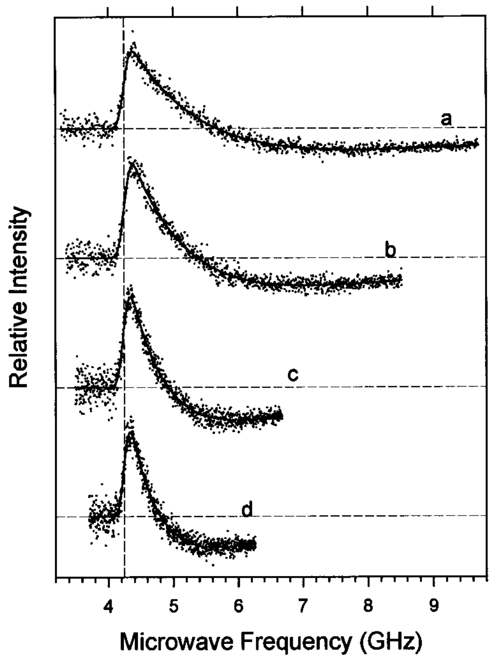

Figure 7. Delayed slow-passage ODMR spectra of the $|D| + |E|$ transition of Trp in EG-buffer. The microwave sweep rate is (a) 200, (b) 160, (c) 100, and (d) 80 MHz/s, and the microwave sweep ranges from 3.8 to 4.6 GHz and is limited by a 5 GHz low-pass filter. The superimposed solid lines represent the calculated best fit of each experimental spectrum; the values of the fitting parameters are listed in Table 5. The dashed vertical line indicates the mean value of ν_0 (4.250 GHz) defined in the text.

Previous reports for $\{D - E, 2E\}$ in gigahertz are {1.742, 2.536}[29] and {1.79, 2.52},[30] which carry estimated errors of ca. ±0.01 GHz. There is reasonable agreement of these $2E$ band center frequencies (but less so with the $D - E$ frequencies) with that obtained in this work.

Kinetics Measurements. The apparent sublevel decay constants of Trp and 7AW were obtained from the delayed slow-passage measurements described above. Although they are not affected by optical pumping kinetics, they are influenced to some extent even at 1.2 K by spin−lattice relaxation (SLR). SLR is

TABLE 2: Characteristic Zero-Field Splitting and Kinetics Parameters for the $|D| - |E|$ Transition of 7AW[a]

sweep rate (MHz/s)	ν_0 (GHz)	$\nu_{1/2}$ (GHz)[b]	R_{zx}	k_x (s^{-1})	k_z (s^{-1})
200[c]	1.3533	0.0156	0.110	0.566	0.092
160[d]	1.3548	0.0160	0.113	0.567	0.091
100[c]	1.3539	0.0163	0.123	0.548	0.089
80[d]	1.3518	0.0156	0.112	0.559	0.081
mean[e]	1.354(1)	0.0159(3)	0.12(1)	0.56(1)	0.09(1)

[a] Parameters are obtained from fitting of delayed slow-passage data. [b] $\nu_{1/2}$ is the half-width at half-maximum. [c] $t_0 = 10.0$ s. [d] $t_0 = 6.0$ s. [e] The standard deviation (σ) in the last digit is given in parentheses.

TABLE 3: Characteristic Zero-Field Splitting and Kinetics Parameters for the $|D| + |E|$ Transition of 7AW[a]

sweep rate (MHz/s)	ν_0 (GHz)	$\nu_{1/2}$ (GHz)[b]	R_{zy}	k_y (s^{-1})	k_z (s^{-1})
200[c]	4.103	0.0870	0.264	0.431	0.091
160[d]	4.103	0.0811	0.282	0.429	0.101
100[c]	4.100	0.0812	0.303	0.394	0.104
80[d]	4.081	0.0779	0.353	0.365	0.115
mean[e]	4.097(10)	0.082(4)	0.30(4)	0.41(3)	0.10(1)

[a] Parameters are obtained from fitting of delayed slow-passage data. [b] $\nu_{1/2}$ is the half-width at half-maximum. [c] $t_0 = 10.0$ s. [d] $t_0 = 6.0$ s. [e] The standard deviation (σ) in the last digit is given in parentheses.

TABLE 4: Characteristic Zero-Field Splitting and Kinetics Parameters for the $|D| - |E|$ Transition of Trp[a]

sweep rate (MHz/s)	ν_0 (GHz)	$\nu_{1/2}$ (GHz)[b]	R_{zx}	k_x (s^{-1})	k_z (s^{-1})
200[c]	1.762	0.0585	0.0871	0.333	0.0421
160[d]	1.765	0.0566	0.0891	0.343	0.0466
160[e]	1.764	0.0584	0.0892	0.348	0.0454
100[c]	1.763	0.0583	0.0976	0.324	0.0457
80[e]	1.763	0.0590	0.0995	0.334	0.0517
mean[f]	1.763(3)	0.058(1)	0.093(6)	0.336(9)	0.046(4)

[a] Parameters are obtained from fitting of delayed slow-passage data. [b] $\nu_{1/2}$ is the half-width at half-maximum. [c] $t_0 = 11.0$ s. [d] $t_0 = 2.0$ s. [e] $t_0 = 1.0$ s. [f] The standard deviation (σ) in the last digit is given in parentheses.

TABLE 5: Characteristic Zero-Field Splitting and Kinetics Parameters for the $|D| + |E|$ Transition of Trp[a]

sweep rate (MHz/s)	ν_0 (GHz)	$\nu_{1/2}$ (GHz)[b]	R_{zy}	k_y (s^{-1})	k_z (s^{-1})
200[c]	4.247	0.0823	0.54	0.155	0.066
160[d]	4.257	0.0852	0.44	0.176	0.053
100[c]	4.244	0.0803	0.63	0.143	0.072
80[d]	4.251	0.0752	0.33	0.203	0.033
mean[e]	4.250(7)	0.081(4)	0.48(13)	0.17(3)	0.056(18)

[a] Parameters are obtained from fitting of delayed slow-passage data. [b] $\nu_{1/2}$ is the half-width at half-maximum. [c] $t_0 = 12.0$ s. [d] $t_0 = 17.0$ s. [e] The standard deviation (σ) in the last digit is given in parentheses.

assumed to be negligible in the development of eq A5. Thus, we refer to them as "apparent" decay constants. Their values are summarized in Table 7 along with the relative sublevel radiative rate constants also obtained from analysis of the delayed slow-passage measurements. The relative radiative rate constants also are influenced by SLR. It may be noticed that trends occur in some of the kinetic and radiative best-fit parameters vs microwave sweep rate in the data sets (Tables 2−4). These trends may be the result of neglecting SLR in the data analysis. Thus, one can expect that the best-fit values of the kinetic parameters incorporate some systematic error.

In the case of 5HW and its anion, sublevel decay kinetics were measured using the MIDP experiment of Schmidt et al.[27] because of the overlapping ODMR bands that made delayed slow passage measurements unreliable. The microwave frequency limits of the fast passage used to induce MIDP signals were confined well within a band in order to avoid exciting more than one transition. Analysis of the data (Simplex method) was done globally using an algorithm that includes SLR effects;[31] thus, the resulting decay constants and relative radiative rate constants have been corrected for the influence of SLR. The sublevel decay constants of 5HW and its anion are given in Table 7 along with the relative radiative rate constants also produced by this analysis.

Global analyses of MIDP measurements on Trp and 7AW also were carried out[31] and provide a quantitative assessment of the effect of SLR on the actual decay constants that are reflected in the apparent values listed in Table 7. The actual values of $\{k_x, k_y, k_z\}$, in s^{-1} obtained from global analysis of the MIDP responses[31] are $\{0.31 \pm 0.01, 0.102 \pm 0.007, 0.000 \pm 0.001\}$ for Trp and $\{0.57 \pm 0.02, 0.38 \pm 0.02, 0.02 \pm 0.01\}$ for 7AW. The standard errors have been included. The major effect of the neglect of SLR in the analysis of delayed slow passage is the overestimation of k_z for both Trp and 7AW. The T_z sublevel decays largely by means of SLR in both triplet states even at $T = 1.2$ K. R_{zx} is severely overestimated in the delayed slow-passage analysis for this reason.

Discussion

The phosphorescence spectra (Figure 1) of the tryptophan analogs are clearly distinguishable from those of Trp itself. The spectra of 7AW, 5HW, and the 5HW anion are shifted to the red of Trp. Each spectrum reveals vibronic structure that is rather similar to that of Trp although more poorly resolved, in general. The phosphorescence lifetime is reduced by the substitutions in the indole chromophore (Table 1). A relatively minor lifetime reduction is caused by the OH substitution, but the effect is much larger in the anion, as would be expected from enhanced spin−orbit coupling effects from the lone pair electrons of oxygen. A significant reduction in the phosphorescence lifetime of tyrosine also results from ionization of the OH proton.[32] Introduction of the aza-nitrogen to form 7AW also shortens the triplet lifetime. This effect is analogous to, but smaller than, that which occurs when naphthalene is converted to quinoline.[33]

Each of the steady state slow-passage ODMR spectra of the tryptophan analogs reveals all three zero-field transitions, in contrast to Trp, in which the high-frequency ν_3 $(D + E)$ transition is of vanishing intensity (Figure 2a). The ODMR bands of 5HW and its anion are very broad relative to those of 7AW and tryptophan, indicating a large degree of inhomogeneous broadening that is probably the result of strong OH−solvent or O^-−solvent interactions. The ODMR bands of 7AW, particularly ν_1, are quite narrow (Table 6). The ODMR frequencies and bandwidths of 5HW and its anion were obtained from the steady state slow-passage measurements, while those of tryptophan and 7AW were obtained from the delayed slow-passage measurements described in the Appendix. Even the $D + E$ band of tryptophan could be observed (Figure 7) and analyzed (Table 5) by this technique.

The ZFS of the triplet states were assigned in a manner that causes the most slowly decaying sublevel to be the lowest in energy. In $^3(\pi, \pi^*)$ states, this is the T_z sublevel (z is the out-of-plane axis). This assignment reveals that the major effect of the hydroxyl at the 5-position is to bring about a large reduction in the magnitude of E. The orientations of the in-plane x- and y-axes of 7AW, 5HW, and its anion are not known; in indole, the x-axis is oriented roughly at right angles to the

TABLE 6: Summary of Characteristic Zero-Field Splitting Parameters of Tryptophan and Analogs[a]

	$\|D\| - \|E\|$		$2\|E\|$		$\|D\| + \|E\|$			
sample	ν_0 (GHz)	$\nu_{1/2}$ (MHz)	ν_0 (GHz)	$\nu_{1/2}$ (MHz)	ν_0 (GHz)	$\nu_{1/2}$ (MHz)	$\|D\|$ (GHz)	$\|E\|$ (GHz)
Trp[b]	1.763(3)	58(1)	2.514(10)	146(6)	4.250(7)	81(4)	3.007	1.250
5HW[c] pH = 7	2.46(1)	252(10)	1.29(3)	440(20)	3.71(2)	410(18)	3.09	0.64
5HW[c] pH = 12.3	2.18(2)	150(20)	0.96(4)	222(20)	3.22(2)	164(20)	2.70	0.50
7AW[b]	1.354(1)	15.9(3)	2.735(3)	55(6)	4.097(10)	82(4)	2.726	1.370

[a] The standard deviation (σ) in the last digit is given in parentheses. [b] Results from the analysis of delayed slow-passage signals, cf. text. [c] Results from fitting of slow-passage signals without considering the fast-passage effects, cf. text.

TABLE 7: Summary of Characteristic Kinetics Parameters of Tryptophan and Analogs

sample	k_x (s^{-1})	k_y (s^{-1})	k_z (s^{-1})	R_{zx}	R_{zy}	R_{yx}
Trp[a]	0.34(1)	0.17(3)	0.046(4)	0.093(6)	0.48(13)	0.19(2)
5HW[b] pH = 7	0.36(1)	0.22(1)	0.00(1)	0.000(4)	*c*	0.57(1)
5HW[b] pH = 12.3	1.32(1)	0.55(1)	0.05(2)	0.04(1)	*c*	0.468(6)
7AW[a]	0.56(1)	0.41(3)	0.10(1)	0.12(1)	0.30(4)	0.3(1)

[a] Results from the analysis of delayed slow-passage signals, cf. text. The standard deviations in the last digit (σ) are given in parentheses. [b] Results from global analysis of MIDP data, cf. text. The standard errors in the last digit (σ) are given in parentheses. [c] Not determined by the experimental method.

ethylenic bond in the five-membered ring.[34] The shortest lived sublevel is intermediate in energy for each molecule, and is labeled T_x.

The large *E*-parameter of Trp, and of 7AW by analogy, results from the contribution of spin density in the ethylenic bond of the five-membered ring which is conjugated with the six-membered ring.[35,36] The large reduction of *E* that occurs when OH or O^- is substituted at the 5-position probably is due to reduced ethylenic spin density in the triplet state. The increase in the value of *E* that occurs upon aza substitution at the 7-position to form 7AW could result from increased conjugation of the ethylenic bond in the triplet state. This substitution also produces a rather large reduction in the *D*-parameter.

The patterns of ODMR linewidths of Trp and 7AW are of interest (Table 6). It has been noted previously[7] that the $2E$ transition of Trp is considerably broader than the $D - E$ transition. This observation has been interpreted to mean that the *E*-parameter is more sensitive to solvent perturbations than is the *D*-parameter. The new data presented in this work allow a more complete interpretation since we have measured the linewidths of all three ODMR transitions of Trp as well as 7AW. If there were no correlation between solvent shifts induced in the *D*- and *E*-parameters, we would expect that the $D - E$ and $D + E$ transitions would have comparable bandwidths. In both Trp and 7AW, however, the $D + E$ transition has a larger bandwidth than the $D - E$ transition. Both *D*- and *E*-parameters are known to be positive in Trp,[7,35] and by analogy also in 7AW. Thus, the bandwidths indicate that there is a positive correlation between solvent perturbations induced in *D* and *E* for each of these triplet states. The tighter correlation appears to occur in 7AW. The extremely narrow $D - E$ band of 7AW suggests the possibility that discrete conformational states frozen into a protein sample incorporating this amino acid could be detected by ODMR.

Analysis of the delayed slow-passage responses of Trp and 7AW was carried out with the assumption that the inhomogeneously broadened band has a Gaussian shape. This need not be the case, in general, except for "random" line-broadening effects. The analytical fitting of the responses assuming a Gaussian band shape produces excellent agreement with the data, as seen in Figures 4−7, suggesting that there is no detectable systematic deviation from a Gaussian shape. The solvent effects are not totally random, however, since the bandwidths suggest a positive correlation between solvent shifts on the *D*- and *E*-parameters, as discussed above. Such correlations could be associated with the observation that for Trp the peak frequencies of the $D - E$ and $2E$ bands do not sum to that of the $D + E$ peak frequency within the expected limits of error (Table 6). Consequently, the standard deviations given in Table 6 are valid only for the center frequencies of the observed ODMR bands and have not been extrapolated to the *D*- and *E*-parameters deduced therefrom.

Conclusions

We have presented the results of phosphorescence and ODMR measurements of the Trp analogs, 7AW, 5HW, and the 5HW anion. The triplet state properties of these analogs differ significantly from those of Trp, which also was reexamined in this work. The properties are consistent with $^3(\pi, \pi^*)$ states in every case. The ODMR frequencies and bandwidths of 7AW and Trp were determined by a new method of analysis of delayed slow-passage spectra that yields more accurate results for the band center frequencies and widths than previous methods. The decay constants obtained from data analysis of delayed slow-passage ODMR responses are influenced to some extent by SLR as are the relative radiative rate constants. The $D + E$ transition of Trp was observed and analyzed for the first time using this method, which generally yields enhanced spin alignment relative to the photostationary state. The ODMR bandwidths of 5HW and its anion are very much larger than those of Trp and 7AW, indicating enhanced solute−solvent interactions. A greatly reduced *E*-parameter in 5HW and its anion relative to Trp points to a large change in spin density distribution with decreased occupation of the ethylenic bond in the five-membered ring. Hydroxyl substitution at the 5-position has little effect on the triplet state decay kinetics, but ionization of the 5HW hydroxyl proton brings about a large decrease in phosphorescence lifetime. Aza substitution at the 7-position of Trp causes relatively minor increases in sublevel decay constants (equally distributed between k_x and k_y), consistent with the introduction of a new spin−orbit coupling pathway involving the N atom lone pair electrons. The 7-aza substitution in Trp also results in a small increase in the *E*-parameter, suggesting that conjugation with the ethylenic bond is enhanced. Analysis of the ODMR bandwidths of Trp and 7AW shows that solvent shifts of the *D*- and *E*-parameters are positively correlated. It is suggested that in the presence of such correlated solvent shifts, the peak frequencies of inhomogeneously broadened ODMR bands need not be numerically related, in the sense that the two lowest peak frequencies must sum to that of the third. On the other hand, the ODMR bands may be skewed and not accurately represented by a symmetrical Gaussian shape, as assumed in the analysis. In either case, the *D* and *E* ZFS parameters appear

to be affected by systematic errors beyond the uncertainties associated with the peak frequencies of the ODMR bands.

The narrow inhomogeneous width of the $D - E$ ODMR band of 7AW ($\nu_{1/2} = 15.9$ MHz) suggests its possible use in detecting discrete frozen out conformers of proteins in which it is incorporated.

Acknowledgment. This publication was made possible by Grant No. ES-02662 from the National Institute of Environmental Health Sciences, NIH.

Appendix

In a recent communication[24] we derived an expression for the "slow-passage" ODMR signal upon saturation of the T_i and T_j sublevels of a photoexcited triplet state during continuous optical pumping. The derivation assumes an inhomogeneously broadened distribution of "isochromats" defined as triplet states whose resonance frequency lies within the homogeneous linewidth, $\delta\nu$. Passage time through an isochromat is assumed to be short enough to fulfill the fast-passage condition such that the ODMR response of the nth isochromat is given by[25]

$$I_n^{ij}(t) = Cg_n[k_i^{(r)} \exp(-k_i t_n) - k_j^{(r)} \exp(-k_j t_n)] \quad \text{(A1)}$$

where I_n^{ij} represents the deviation of the phosphorescence intensity from its steady state level in the absence of microwaves, t_n is measured from the time of fast-passage whose duration is assumed to be negligible relative to sublevel lifetimes, $k_{i,j}$ are the decay constants of the $T_{i,j}$ sublevels, and $k_{i,j}^{(r)}$ are their radiative rate constants. C is a proportionality constant that depends on the spin alignment and the experimental arrangement, while g_n is the population of the nth isochromat. Equation A1 assumes that spin−lattice relaxation (SLR) can be neglected and that optical pumping does not deplete the ground state population significantly.[7] The line shape of the inhomogeneously broadened distribution of isochromats is then a superposition of fast-passage responses each given by eq A1. The response is converted from the time to the frequency domain by a linear transformation using the constant microwave sweep rate. The resulting expression (assuming that each isochromat has the same rate constants) is[24]

$$I^{ij}(p\Delta\nu) = C' \sum_{n \le p} g_n\{\exp[-\kappa_i(p-n)] - R_{ji} \exp[-\kappa_j(p-n)]\} \quad \text{(A2)}$$

Equation A2 is expressed in terms of channels, n and p, of a multichannel analyzer that is normally used to acquire data. The frequency width of a channel is $\Delta\nu$, and $p\Delta\nu$ is the microwave frequency corresponding to the pth channel. In eq A2, $\kappa_{i,j} = k_{i,j}T/N$, where T is the time required to accumulate N channels of data, $R_{ji} = k_j^{(r)}/k_i^{(r)}$, and $C' = Ck_i^{(r)}$.

We have successfully fit eq A2 to slow-passage ODMR signals of tryptophan in hen egg-white lysozyme using a Gaussian distribution for g_n,

$$g_n = \exp[-(\nu - \nu_0)^2 \ln 2/\nu_{1/2}^2] = \exp[-n^2(\Delta\nu)^2 \ln 2/\nu_{1/2}^2] \quad \text{(A3)}$$

where $\nu_{1/2}$ is the half-width at half-height and $n = 0$ defines the band center.[24] The parameters used in a nonlinear least-squares minimization procedure were $k_{i,j}$, ν_0, $\nu_{1/2}$, and R_{ji}.

The procedure outlined above can be adapted to slow-passage measurements carried out during decay of the phosphorescent state by including in it the time dependence of the sublevel populations of T_i and T_j within each isochromat. These enter through the spin alignment, A_n^{ij}, which becomes time-dependent because of the decay of the triplet state after cessation of optical pumping. Ignoring SLR, the time dependence of the spin alignment corresponding to the $T_i \leftrightarrow T_j$ transition of the nth isochromat is

$$A_n^{ij}(t) = [N_{jn}^{\ 0}\, e^{-k_j t_0} \exp(-\kappa_j n) - N_{in}^{\ 0}\, e^{-k_i t_0} \exp(-\kappa_i n)] \quad \text{(A4)}$$

In eq A4, t_0 is the time between cessation of excitation ($t = 0$) and the commencement of the microwave sweep cycle ($n = 0$ occurs at t_0 in eq A4), and $N_{jn}^0 - N_{in}^0$ is the spin alignment of the nth isochromat at $t = 0$. Combining eqs A4 and A2, we get

$$I^{ij}(p\Delta\nu) = C^{ij} \sum_{n \le p} g_n[e^{-k_j t_0} \exp(-\kappa_j n) - N_{ij}^{\ 0}\, e^{-k_i t_0} \exp(-\kappa_i n)] \times \{\exp[-\kappa_i(p-n)] - R_{ji} \exp[-\kappa_j(p-n)]\} \quad \text{(A5)}$$

where $N_{ij}^0 = N_i^0/N_j^0$, g_n is a shape function for the inhomogeneous distribution, and C^{ij} is a factor proportional to $N_j^0\, k_i^{(r)}$. By analogy with eq A2, I^{ij} of eq A5 is the ODMR response during phosphorescence decay after subtracting the intensity of the phosphorescence decay in the absence of microwaves. We have used a nonlinear least-squares minimization procedure to fit eq A5 to experimental data obtained in this work. The parameters used in the least-squares minimization are the same as for eq A2, above. Since the least-squares minimization is extremely insensitive to N_{ij}^0, this quantity is obtained from independent measurements,[28] and its value is fixed in the analysis.

References and Notes

(1) Beechem, J. M.; Brand, L. *Annu. Rev. Biochem.* **1985**, *54*, 43.
(2) Creed, D. *Photochem. Photobiol.* **1984**, *39*, 537.
(3) Kwiram, A. L. *Chem. Phys. Lett.* **1967**, *1*, 272.
(4) Sharnoff, M. *J. Chem. Phys.* **1967**, *46*, 3263.
(5) Zuclich, J.; Schweitzer, D.; Maki, A. H. *Biochem. Biophys. Res. Commun.* **1972**, *46*, 1764; *Photochem. Photobiol.* **1973**, *18*, 161.
(6) Kwiram, A. L. *MTP Int. Rev. Sci. Phys. Chem. Ser. 1* **1972**, *4*, 271.
(7) For reviews of ODMR of proteins, see: Kwiram, A. L. In *Triplet State ODMR Spectroscopy*; Clarke, R. H., Ed.; Wiley: New York, 1982; p 427. Maki, A. H. In *Biological Magnetic Resonance*; Berliner, L. H., Reuben, J., Eds.; Plenum: New York, 1984; Vol. 6, p 187. Hoff, A. J. In *Advanced EPR with Applications to Biology and Biochemistry*; Hoff, A. J., Ed.; Elsevier: Amsterdam, 1989; p 633. Maki, A. H. *Methods Enzymol.* **1995**, *246*, 610.
(8) Negrerie, M. J.; Bellefeuille, S. M.; Whitham, S.; Petrich. J. W.; Thornburg, R. W. *J. Am. Chem. Soc.* **1990**, *112*, 7419.
(9) Chen, Y.; Gai, F.; Petrich, J. W. *J. Phys. Chem.* **1994**, *98*, 2203.
(10) Hogue, C. W.; Szabo, A. G. *Biophys. Chem.* **1993**, *48*, 159.
(11) Pardee, A. B.; Shore, V. G.; Prestidge, L. S. *Biochim. Biophys. Acta* **1956**, *21*, 406.
(12) Sharon, N.; Lipmann, F. *Arch. Biochem. Biophys.* **1957**, *69*, 219.
(13) Schlesinger, S. *J. Biol. Chem.* **1968**, *243*, 3877.
(14) Barlati, S.; Ciferi, O. *J. Bacteriol.* **1970**, *101*, 166.
(15) Foote, J.; Ikeda, D. M.; Kantrowitz, E. R. *J. Biol. Chem.* **1980**, *255*, 5154.
(16) Hogue, C. W. V.; Rasquinha, I.; Szabo, A. G.; MacManus, J. P. *FEBS Lett.* **1992**, *310*, 269.
(17) Ross, J. B. A.; Senear, D. F.; Waxman, E.; Kombo, B. B.; Rusinova, E.; Huang, Y. T.; Laws, W. R.; Hasselbacher, C. A. *Proc. Natl. Acad. Sci. U.S.A.* **1992**, *89*, 12023.
(18) Brennan, J. D.; Clark, I. D.; Hogue, C. W. V.; Ito, A. S.; Juliano, L.; Paiva, A. C. M.; Rajendran, B.; Szabo, A. G. *Appl. Spectrosc.* **1995**, *49*, 51.
(19) Ingham, K. C.; Abu-Elgheit, M.; El-Bayoumi, M. A. *J. Am. Chem. Soc.* **1971**, *93*, 5023.
(20) Hetherington, W. M., III; Micheels, R. H.; Eisenthal, K. B. *Chem. Phys. Lett.* **1979**, *66*, 230.
(21) Waluk, J.; Bulska, H.; Pakula, B.; Sepiol, J. *J. Lumin.* **1981**, *24/25*, 519.

(22) Bulska, H.; Grabowska, A.; Pakula, B.; Sepiol, J.; Wild, U. P. *J. Lumin.* **1984**, *29*, 65.

(23) Sato, A.; Bitten, E.; Lambert, D.; Rousslang, K. *Proc. SPIE-Int. Soc. Opt. Eng.* **1994**, *2137*, 343.

(24) Wu, J. Q.; Ozarowski, A.; Maki, A. H. *J. Magn. Reson. Ser. A* **1996**, *119*, 82.

(25) Winscom, C. J.; Maki A. H. *Chem. Phys. Lett.* **1971**, *12*, 264.

(26) Moore, T. A.; Kwiram, A. L. *Chem. Phys. Lett.* **1976**, *39*, 231.

(27) Schmidt, J.; Antheunis, D.; van der Waals, J. H. *Mol. Phys.* **1971**, *22*, 1.

(28) Shain, A. L.; Sharnoff, M. *J. Chem. Phys.* **1973**, *59*, 2335.

(29) Deranleau, D. A.; Ross, J. B. A.; Rousslang, K. W.; Kwiram, A. L. *J. Am. Chem. Soc.* **1978**, *100*, 1913.

(30) Burns, L. E. Thesis, University of California, Davis, **1992**.

(31) Ozarowski, A.; Wu, J. Q.; Maki, A. H. *J. Magn. Reson. Ser. A*, in press.

(32) Co, T.; Hoover, R. J.; Maki, A. H. *Chem. Phys. Lett.* **1974**, *27*, 5.

(33) McGlynn, S. P.; Azumi, T.; Kinoshita, M. *Molecular Spectroscopy of the Triplet State*; Prentice Hall: Englewood Cliffs, NJ, 1969; Chapter 6.

(34) Smith, C. A.; Maki, A. H. *J. Phys. Chem.* **1993**, *97*, 997.

(35) Kochanski, E.; Pullman, A. *Int. J. Quantum Mech.* **1969**, *3*, 1055.

(36) Harrigan, E. T.; Hirota, N. *J. Am. Chem. Soc.* **1975**, *97*, 6647.

JP9606422
