# Supplementary material for: 5-Hydroxytryptophan (5-HTP): Natural Occurrence, Analysis, Biosynthesis, Biotechnology, Physiology and Toxicology
Source: Int J Mol Sci. 2020 Dec 26;22(1):181. doi: 10.3390/ijms22010181 (PMC7796270; doi:10.3390/ijms22010181)
Supplement: Supplementary file 1 [file ijms-22-00181-s001.zip › 5-HTP.Data/PDF/0297311198/Barbosa-2008-Tryptophan-hydroxylase-is-modulat.pdf]

Available online at www.sciencedirect.com
ScienceDirect
Life Sciences 82 (2008) 529–535
Life Sciences
www.elsevier.com/locate/lifescie

Tryptophan hydroxylase is modulated by L-type calcium channels in the rat pineal gland

Roseli Barbosa [a], Julieta Helena Scialfa [b], Ilza Mingarini Terra [b], José Cipolla-Neto [b], Valérie Simonneaux [c], Solange Castro Afeche [a,*]

[a] Laboratory of Pharmacology, Butantan Institute, São Paulo, SP, Brazil
[b] Department of Physiology and Biophysics, Institute of Biomedical Sciences, University of São Paulo, São Paulo, SP, Brazil
[c] Neurobiologie des Rythmes, Institut de Neurosciences Cellulaires et Intégratives, CNRS/Université Louis Pasteur, Strasbourg, France

Received 25 October 2007; accepted 12 December 2007

Abstract

Calcium is an important second messenger in the rat pineal gland, as well as cAMP. They both contribute to melatonin synthesis mediated by the three main enzymes of the melatonin synthesis pathway: tryptophan hydroxylase, arylalkylamine *N*-acetyltransferase and hydroxyindole-*O*-methyltransferase. The cytosolic calcium is elevated in pinealocytes following α_1-adrenergic stimulation, through IP_3-and membrane calcium channels activation. Nifedipine, an L-type calcium channel blocker, reduces melatonin synthesis in rat pineal glands in vitro. With the purpose of investigating the mechanisms involved in melatonin synthesis regulation by the L-type calcium channel, we studied the effects of nifedipine on noradrenergic stimulated cultured rat pineal glands. Tryptophan hydroxylase, arylalkylamine *N*-acetyltransferase and hydroxyindole-*O*-methyltransferase activities were quantified by radiometric assays and 5-hydroxytryptophan, serotonin, *N*-acetylserotonin and melatonin contents were quantified by HPLC with electrochemical detection. The data showed that calcium influx blockaded by nifedipine caused a decrease in tryptophan hydroxylase activity, but did not change either arylalkylamine *N*-acetyltransferase or hydroxyindole-*O*-methyltransferase activities. Moreover, there was a reduction of 5-hydroxytryptophan, serotonin, *N*-acetylserotonin and melatonin intracellular content, as well as a reduction of serotonin and melatonin secretion. Thus, it seems that the calcium influx through L-type high voltage-activated calcium channels is essential for the full activation of tryptophan hydroxylase leading to melatonin synthesis in the pineal gland.
© 2007 Elsevier Inc. All rights reserved.

Keywords: L-type calcium channel; Melatonin; Arylalkylamine *N*-acetyltransferase; Tryptophan hydroxylase; Hydroxyindole-*O*-methyltransferase; Pineal gland

Introduction

Melatonin is the main hormone synthesized and released from the pineal gland following sympathetic stimulation during the night. Norepinephrine interacts with α-and β-adrenoceptors in the pinealocyte membranes and, consequently, elevates intracellular cAMP and calcium levels (Sugden et al., 1986; Sugden, 1989). These two second messengers participate in some way in the synthesis and activation of the three most important enzymes in the melatonin biosynthetic pathway (Sugden et al., 1986; Ehret et al., 1989, 1991; Klein et al., 1970; Ribelayga et al., 1997, 1999; Yu et al., 1993). Tryptophan hydroxylase (TPH, EC 1.14.16.4) is the first enzyme, catalyzing the transformation of tryptophan into 5-hydroxytryptophan, and is the rate-limiting step in the serotonin synthesis. Serotonin accumulates in the pineal gland during the day and, only at night, due to the activation of the enzyme arylalkylamine *N*-acetyltransferase (AA-NAT; EC 2.3.1.87), it is converted to *N*-acetylserotonin, that is then transformed into melatonin by the catalysis of hydroxyindole-*O*-methyltransferase enzyme (HIOMT, EC 2.1.1.4) (Sugden, 1989; Simonneaux and Ribelayga, 2003).

* Corresponding author. Laboratory of Pharmacology, Butantan Institute, Av. Vital Brazil, 1500, São Paulo, SP, 05503-900, Brazil. Tel.: +55 11 3726 7222; fax: +55 11 3091 7629.
E-mail address: soafeche@butantan.gov.br (S. Castro Afeche).

0024-3205/$ - see front matter © 2007 Elsevier Inc. All rights reserved.
doi:10.1016/j.lfs.2007.12.011

In rat pineal gland, TPH synthesis and activity are stimulated by norepinephrine. cAMP, through the activation of cAMP-dependent protein kinase A (PKA), phosphorylates the transcription factor cAMP response element-binding protein (CREB), which starts the enzyme synthesis (Ehret et al., 1991). In addition, enzyme phosphorylation by PKA, protein kinase C (PKC) and Ca^{2+}/calmodulin-dependent protein kinase and its further association with 14-3-3 protein promotes its activation (Ehret et al., 1989; Banik et al., 1997; Ichimura et al., 1987; Johansen et al., 1995). It was demonstrated that the predominant TPH isoform in the pineal gland, that shows circadian rhythm peaking at night, is TPH1. THP2 is also present, but in much less quantity and does not show the typical daily pattern (Sugden, 2003; Patel et al., 2004).

AA-NAT has its synthesis and activity stimulated by norepinephrine at night. Differently from TPH, however, AA-NAT activity is elevated by 150 times (whereas TPH activity is increased twice at night), and although the same cAMP/PKA/CREB pathway (Maronde et al., 1999; Roseboom and Klein, 1995; Roseboom et al., 1996) is involved in the synthesis of both enzymes, for the AA-NAT this transcriptional process is a pre-requisite for its activation. This, in turn, is accomplished by PKA and PKC phosphorylation, and the subsequent complex formation with 14-3-3 protein (Ganguly et al., 2001).

The HIOMT regulation is different from that of TPH and AA-NAT. HIOMT gene expression is partly activated by norepinephrine at night. In addition HIOMT activity is further stimulated by neuropeptide Y (NPY) that promotes intracellular calcium elevation (Ribelayga et al., 1999; Simonneaux et al., 1999).

The cytosolic calcium level in the pinealocyte is increased by α_1-adrenergic activation, acting in both mobilization from the endoplasmic reticulum by IP_3-stimulation and through the store-operated calcium channels (“SOCs”) (Gomperts et al., 2002; Lee et al., 2006; Parekh and Putney, 2005). Moreover, high voltage-activated calcium channels are also present in the rat pineal gland (Afeche et al., 2006; Chik et al., 1995, 1997; Chin et al., 1992) and these seem to be activated either by acetylcholine or norepinephrine (Afeche et al., 2006; Darvish and Russell, 1998; Letz et al., 1997). We have recently reported that the blockade of L-type calcium channel with nifedipine, a specific L-type calcium channel antagonist belonging to dihydropyridine class, reduced melatonin synthesis (Afeche et al., 2006), but the mechanisms involved were not resolved.

With the purpose of clarifying the modulation of melatonin synthesis and secretion by the L-type calcium channels, we studied the effects of nifedipine, in noradrenergic stimulated cultured rat pineal glands. The results showed that both melatonin synthesis and secretion stimulated by norepinephrine were impaired by nifedipine and that TPH activity is the main step involved. Neither AA-NAT nor HIOMT activities were affected by nifedipine. The indoles 5-hydroxytryptophan (5-HTP), serotonin (5-HT) and *N*-acetylserotonin (NAS) were all reduced in pinealocytes by nifedipine, probably as a consequence of TPH activity reduction.

Materials and methods

Animals

Young male Wistar rats were kept under a 12:12 light–dark cycle (lights on at 7:00 am) in a temperature-controlled (21± 2 °C) room with water and food ad libitum.

All procedures were approved by the Institute of Biomedical Sciences (University of São Paulo, Ethical Committee for Animal Research, Brazil; CEEA) and are in agreement with the ethical principles in animal research adopted by the Brazilian College of Animal Experimentation (COBEA).

Pineal gland culture

The animals (weighing 150–180 g) were decapitated and their pineal glands were removed and immediately placed in ice-cold Biggers, Gwatkin, Judah medium with Fitton–Jackson modification (BGJ_b — Gibco, Grand Island, NY, USA), with the addition of BSA (1 mg/ ml), 2 mM glutamine, 0.1 mg/ ml ascorbic acid, and penicillin (100 U/ ml)–streptomycin (100 µg/ml). Pineal glands were cultured as described by Parfitt et al. (1976). Briefly, pineal glands were incubated (37 °C; 95% O_2–5% CO_2) in BGJ_b medium on 24-well plates (2 glands/well; 200 µl/well) for 48 h (the time needed for the occurrence of total degeneration of the presynaptic elements) before treatments and the medium was changed after the first 24 h. After 48 h of culture, the glands were placed in fresh medium for 1 h and then submitted to one of the treatments described below. After 5 h of treatment, the glands were collected, frozen in dry ice and kept at −85 °C until assayed.

Pinealocyte culture

Pineal glands were dissociated by papain digestion (Papain Dissociation System, Worthington Biochemical Corporation, Freehold, NJ). The glands were isolated (from rats with approximately 100 g) and immediately placed in ice-cold Dulbecco’s Modified Eagle’s Medium (DMEM) (glucose: 1000 mg/l, HEPES: 5.9 g, sodium bicarbonate: 3.7 g) (St. Louis, MO, USA). Then, the tissue was incubated at 37 °C for 50 min in papain (0.01%) and DNase (0.01%) solution. After removal of papain and its blockade with ovomucoid (2 mg/ml), the pinealocytes were mechanically dispersed and resuspended in DMEM supplemented with 10% fetal calf serum and 1% penicillin–streptomycin to obtain the concentration of 2×10^5 cells/ml. Five ml of cells in a culture medium were cultivated in 25 cm^2 culture flasks, at 37 °C, in 5% CO_2/95% air, for 24 h.

Melatonin and indole assays

Melatonin, NAS, 5-HT and 5-HTP contents were determined by HPLC with electrochemical detection (Waters System, Milford, MA, USA). The indolamines were separated on a Resolve C18 column (5 µm, 3.9×150 mm). The chromatographic

system was isocratically operated with the following mobile phase: 0.1 M sodium acetate, 0.1 M citric acid, 0.15 mM EDTA, 30% (melatonin) or 10% (5-HT, NAS and 5-HTP) methanol, pH 3.7, at a flow rate of 1 ml/min. The electrochemical detector potential was adjusted to +900 mV. Each gland was sonicated in a solution of 0.1 M perchloric acid (120 µl) containing 0.02% EDTA and 0.02% sodium bisulfate. After centrifugation, 50 µl of the supernatant (or 50 µl of the 4:1 culture medium in 0.1 M perchloric acid, 0.02% EDTA, 0.02% sodium bisulfate solution) was injected into the chromatographic system (Injector Mod. 7125, 20 µl loop, Rheodyne Inc). The indoles were quantified using the Millennium 32 Software (Waters System, Milford, MA, USA).

TPH activity determination

Each pineal gland was sonicated in sodium phosphate buffer (2 mM, pH 7, 100 µl). To each sample were added: HEPES (50 mM, pH 7), catalase (100µg/ml), tryptophan (50 µM), dithiothreitol (5 mM), Fe $(NH_4)_2$ $(SO_4)_2$ (10 µM), 6-MPH_4 (500 µM) and 1 µl of $[^3H]$tryptophan (1 mCi/ml — previously dried under nitrogen). The material was incubated at 37 °C for 10 min. After a charcoal activated solution has been added (7.5% in 1 M HCl) to interrupt the reaction, 200 µl of the supernatant were transferred to scintillation tubes, liquid scintillation was added and radioactivity was evaluated with a Beckman LS6500 β counter.

AA-NAT activity determination

AA-NAT activity was measured by a radiometric assay (Deguchi and Axelrod, 1972, modified by Parfitt et al., 1975). Briefly, 100 µl of 0.1 M sodium phosphate buffer, pH 6.8, containing 40 mM tryptamine and $[^3H]$-acetyl coenzyme A (2 mM, final specific activity = 4 mCi/mmol) were added to a microcentrifuge tube containing one gland kept at 4 °C. The glands were sonicated and then incubated at 37°C for 20 min. The reaction product *N*-^{3}[H]-acetyltryptamine was extracted with chloroform (1 ml). Samples of 500 µl were evaporated until dry in a scintillation vial and radioactivity was determined with a Beckman LS6500 β counter.

HIOMT activity determination

HIOMT activity was assayed as previously described (Ribelayga et al., 1997; Axelrod and Weissbach, 1961). The pinealocytes were sonicated in a phosphate buffer (0.05 M, pH 7.9, 50 µl) and soon afterwards 150 µl of a solution containing ^{14}C-S-adenosyl-L-methionine (activity 43.8 mCi) and *N*-acetylserotonin (1 mM) was added. The homogenates were incubated for 30 min at 37 °C. The reaction was interrupted by adding 200 µl of sodium borate buffer (12.5 mM, pH = 10) and 1 ml of chloroform saturated in water. The tubes were centrifuged at 13,000g rotation for 5 min at 4 °C. The product ^{14}[C] melatonin was extracted in 800 µl of chloroform, which was subsequently evaporated, and the radioactivity evaluated with a Beckman LS6500 β counter.

Protein assay

Protein content was measured in tissue homogenate as described in Ribelayga et al (1999).

Drugs and reagents

(-) Arterenol bitartrate salt, (-) Isopropylarterenol hydrochloride, N^6, 2-*O*-dibutyryladenosine 3′-5′cyclic monophosphate, nifedipine, 5-hydroxy-l-tryptophan, 5-hydroxytryptamine, *N*-acetylserotonin and melatonin were purchased from Sigma (St. Louis, MO, USA); culture products from Gibco (Grand Island, NY, USA); salts and reagents were purchased from Merck (Brazil). Radiochemicals were obtained from Amersham Biosciences (Brazil).

Norepinephrine and isoproterenol were dissolved in 0.01 N HCl and then in an ascorbic acid solution (50 mg/l). Nifedipine was first dissolved in ethanol (the concentration was always less than 0.1%) and dilutions were made in BGJb medium. Dibutyryl-cAMP was prepared in BGJb medium.

Experimental procedures

The following experimental groups were made and the experiments were replicated at least 3 times.

Pineal glands were stimulated with norepinephrine (NE) (1 µM) in the absence or in the presence of several concentrations of nifedipine (NIF) (0.1 µM to 30 µM) and intracellular melatonin was measured. NIF was added 10 min before the addition of NE (at least $n = 30$ for each NIF concentration).

Pineal glands were stimulated with NE (0.3 nM to 1 µM), isoproterenol (ISO) (0.1 nM to 1 µM) or dibutyryl-cAMP (DBcAMP) (0.1 mM to 3 mM) in the absence or in the presence of NIF (10 µM added 10 min before gland stimulation) and intracellular melatonin was measured (at least $n = 120$ for each dose–response curve).

Pineal glands or pinealocytes were stimulated with NE (1 nM–10 µM) in the absence ($n = 30$) or in the presence of NIF (10 µM, added 10 min before NE; $n = 30$) and TPH, AA-NAT, and HIOMT activities were measured.

Pineal glands were stimulated with NE (1 µM) in the absence ($n = 30$) or in the presence of NIF (10 µM, added 10 min before NE; $n = 32$) and indoles (5-HT, 5-HTP, NAS) were measured.

Pineal glands or pinealocytes were stimulated with NE (1 µM) in the absence ($n = 16$) or presence ($n = 14$) of NIF (10 µM), and the serotonin and melatonin contents in the culture medium were measured.

Statistical analysis

Data were always presented as mean ± SEM. Indoles content in pineal glands, pinealocytes and culture medium were expressed as ng/gland, ng/2×10^5cells and ng/well, respectively. AA-NAT, TPH, and HIOMT activities were expressed in pmol or nmol of radioactive product/µg protein/hour. Statistical analyses (GraphPad Prism 5.0) were performed using ANOVA followed either by the Bonferroni post-hoc test (comparison

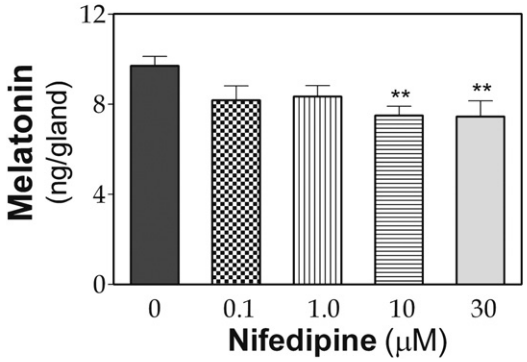

Fig. 1. Effects of several concentrations of nifedipine on melatonin content of cultured rat pineal glands stimulated with norepinephrine (1 μM). $^{**}p<0.01$; significantly different from control.

between all groups) or by the Dunnett's post-hoc test (comparison of groups against the control). When appropriate, Student's *t* test was used.

Results

NIF at the concentrations of 10 and 30 μM significantly reduced melatonin synthesis by 20–25% in cultured pineal glands stimulated by NE (1 μM) ($p<0.01$, $n=20$ for each group) (Fig. 1), whereas with lower doses of NIF there was a reduction, but not statistically significant. Likewise, NIF reduced melatonin released by pinealocytes, as was observed by the quantification of melatonin in the culture medium ($p<0.01$, $n=10$ for each group) (Fig. 2). The effects of NIF on melatonin content appeared to involve a reduction of all the precursors of its synthetic pathway. Accordingly, it was seen a reduction for 5-HTP, 5-HT, and NAS in cultured pineal glands stimulated by norepinephrine 1 μM ($p<0.01$; for each group $n=20$) (Fig. 3).

Besides the intracellular content, serotonin was also measured in the culture medium of glands stimulated by 1 μM NE, and in this condition serotonin was lesser when the glands were previously incubated with NIF ($p<0.01$; $n=10$ for each group) (Fig. 4).

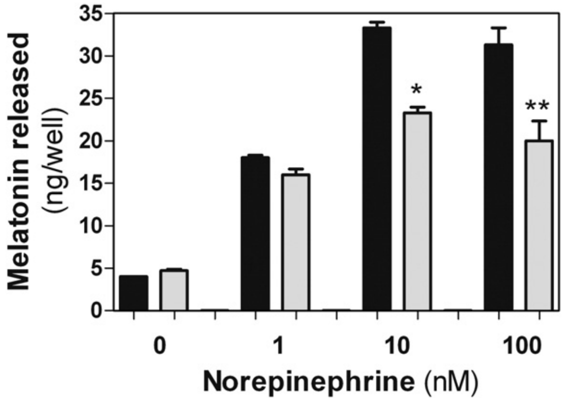

Fig. 2. Melatonin released by rat pinealocytes in culture when stimulated with norepinephrine, in the absence (black bars) or in the presence of nifedipine (10 μM) (grey bars). Melatonin was measured in the culture medium. $^{*}p<0.05$ and $^{**}p<0.01$; significantly different from respective controls.

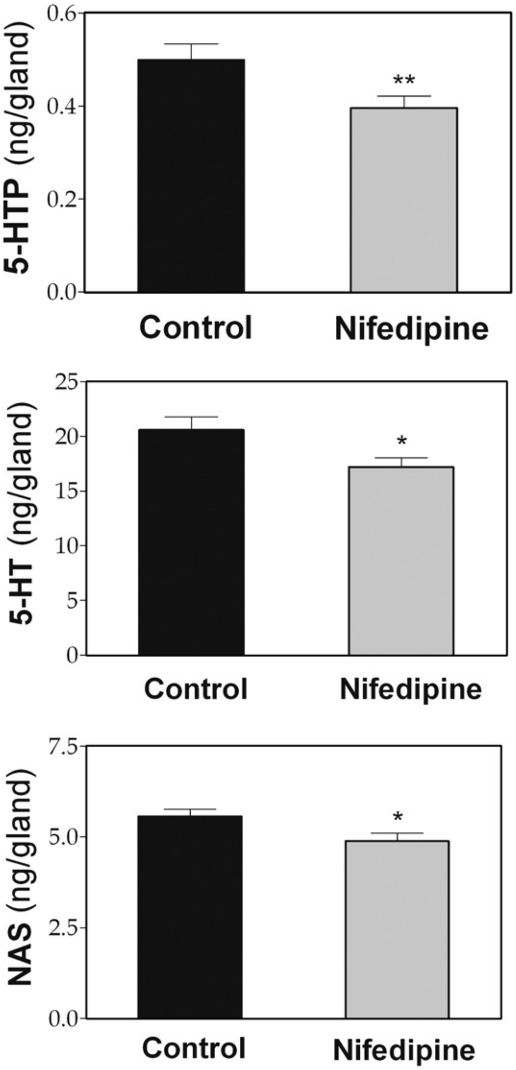

Fig. 3. Effects of nifedipine (10 μM) on the content of 5-hydroxytriptophan (5-HTP), serotonin (5-HT) and *N*-acetylserotonin (NAS) in cultured rat pineal glands, stimulated with norepinephrine (1 μM). $^{*}p<0.05$ and $^{**}p<0.01$; significantly different from respective controls.

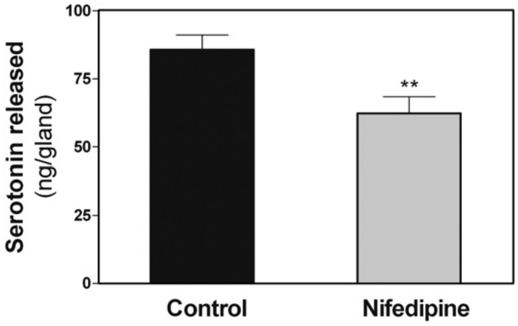

Fig. 4. Effect of nifedipine (10 μM) on the amount of serotonin released by rat pineal glands in culture stimulated with norepinephrine (1 μM). Serotonin was measured in the culture medium. $^{**}p<0.01$; significantly different from control.

Increasing concentrations of NE, ISO and DBcAMP increased pineal gland melatonin synthesis in a concentration-dependent manner, as it would be expected [maximal responses: 7.74±0.45 ng/gland (NE); 7.75±0.33 ng/gland (ISO); 6.78±0.45 ng/gland (DBcAMP)]. The previous addition of NIF to the culture, significantly reduced the maximal responses of melatonin production for all the treatments ($p<0.01$; $n=18$ for each group) (Fig. 5).

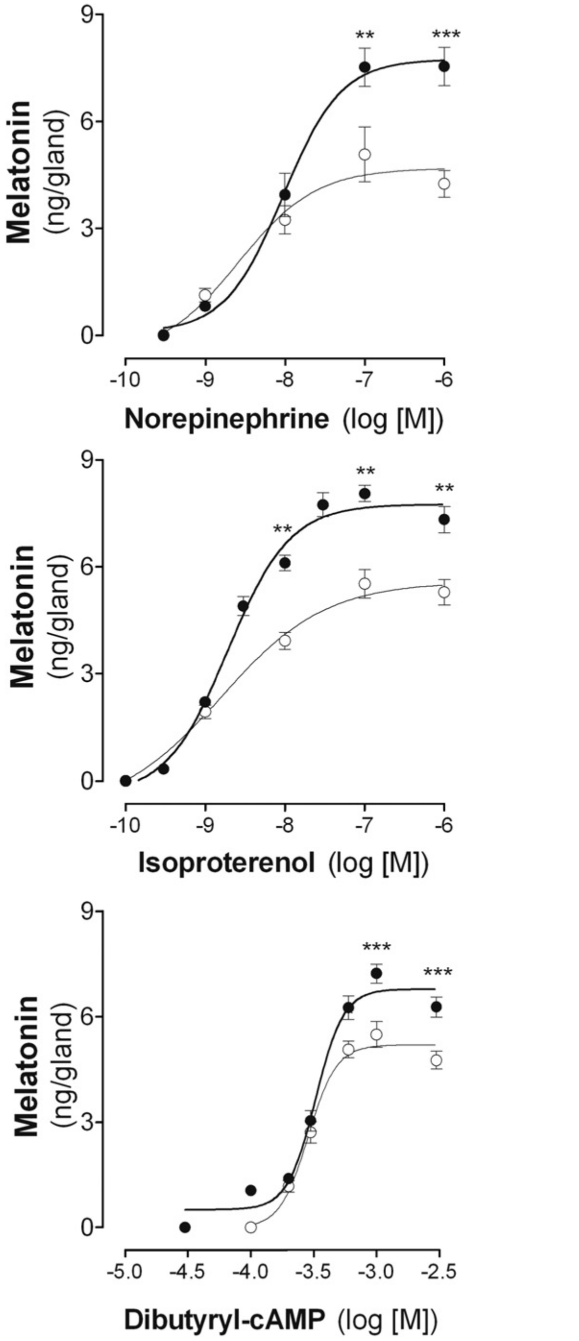

Fig. 5. Dose–response curves of melatonin content of cultured rat pineal glands stimulated with different concentrations of norepinephrine, isoproterenol and dibutyryl-cAMP, in the absence (black dots) or in the presence of nifedipine (10 μM) (white dots). **$p<0.01$; ***$p<0.001$; significantly different from respective controls.

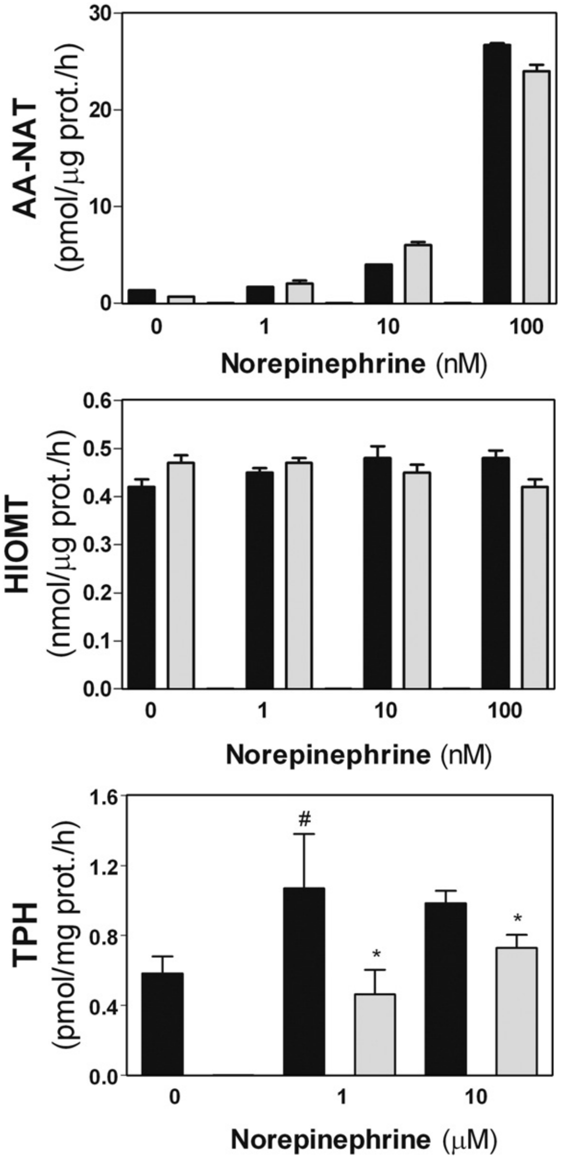

Fig. 6. Effects of nifedipine (10 μM) on AA-NAT, HIOMT, and TPH activities in cultured rat pinealocytes (AA-NAT and HIOMT) or pineal glands (TPH) stimulated with norepinephrine. Black bars=controls; Grey bars=with nifedipine. *Significantly different from respective controls stimulated with norepinephrine 1 μM and 10 μM, $p<0.05$; # significantly different from control without stimulation, $p<0.05$.

For better understanding these effects of NIF on melatonin synthesis and secretion, we analyzed AA-NAT and HIOMT activities in cultured pinealocytes stimulated by NE (1 nM to 100 nM). We observed that AA-NAT activity responds in a concentration-dependent manner, showing the higher response at the NE concentration of 100 nM. HIOMT, on the contrary, did not change their activity in response to different concentrations of NE. In these conditions, previous NIF addition (10 μM) did not alter AA-NAT or HIOMT activity in any NE concentration used ($p>0.05$; $n=10$ for each group) (Fig. 6).

TPH activity, on his turn, increased in approximately two-times by NE stimulation with regard to the control non-stimulated glands ($p<0.05$) (Fig. 6). Moreover, NIF reduced

TPH activity by 55% at 1 μM and by 26% at 10 μM of NE ($p<0.05$; $n=10$ for each group) (Fig. 6).

Discussion

This study showed that the L-type high voltage-activated calcium channels modulate adrenergic-induced melatonin synthesis and secretion in cultured rat pineal glands because the blockade of these channels by nifedipine reduced intracellular and released melatonin.

The concentrations of NIF that were effective in reducing melatonin were 10 μM and 30 μM, and, then, in the subsequent studies we used the minimum concentration (10 μM) that diminished melatonin.

The reduction of melatonin by nifedipine involves the cAMP/β-adrenergic pathway and is dependent on mechanisms situated downward to cAMP synthesis. This conclusion derives from the fact that the concentration–response curves to isoproterenol, a β-adrenergic receptor agonist, and to dibutyryl-cAMP, a permeable cAMP analogue, both have their maximal responses reduced by nifedipine. There are, at least, two possible pathways by which the cAMP/β-adrenoceptor activation would promote the influx of calcium. First, cAMP could act on the described cAMP-dependent nonspecific cationic channels of pineal cells, inducing membrane depolarization and, as a consequence, the opening of L-type calcium channels (Darvish and Russell, 1998). Second, PKA could phosphorylate those calcium channels and facilitate their opening, as demonstrated for L-type calcium channels in the heart (Hille, 2001).

AA-NAT activity was stimulated by NE, as expected (Roseboom et al., 1996), with the maximal response induced by 100 nM of NE. HIOMT activity, in contrast, was not changed by NE addition, which is in accordance with the results found in the literature, that HIOMT activity is not regulated by NE, but instead it depends of NPY (Ribelayga et al., 1997, 1999). AA-NAT and HIOMT activities induced by several concentrations of NE were not modified by the blockade of L-type calcium channels with nifedipine. However, there are evidences that both AA-NAT and HIOMT activities are dependent of calcium. In the absence of calcium AA-NAT activity was reduced in cultured pineal glands stimulated by isoproterenol or dibutyryl-cAMP (Yu et al., 1993; Zatz and Romero, 1978). The possible explanation is that the calcium ions that promote the potentiation of the AA-NAT activity come from a different source, not related to the membrane voltage-dependent channels, as calcium is recognizably compartmentalized within the cells (Gomperts et al., 2002). The lack of action of nifedipine on HIOMT activity of NE-stimulated pinealocytes could be explained by the fact that its regulation, as mentioned before does not depend on the noradrenergic pathway (Ribelayga et al., 1997, 1999).

TPH had its activity stimulated in approximately two-times by NE. In this case, the concentrations of NE that induced this effect were higher (1 μM and 10 μM) than that for inducing the maximal response for AA-NAT activity (100 nM). TPH activity was significantly reduced by the nifedipine-induced L-type calcium channel blockade. The mechanism by which calcium modulates TPH activity may involve the Ca^{2+}/calmodulin system, since it has been demonstrated for the brain TPH, that its phosphorylation and, in consequence, its activation is dependent on Ca^{2+}/calmodulin complex (Ehret et al., 1991). It is not completely clear yet if the situation in the pineal gland is similar to the pattern described in the brain. In support of this hypothesis, it should be mentioned that the phosphorylation of TPH by Ca^{2+}/calmodulin enables its association with the 14-3-3 protein, which was identified in the rat pineal gland. This complex formation promotes an increase of TPH activity by limiting its dephosphorylation (Ichimura et al., 1987; Klein et al., 2003).

Another possibility of calcium action on TPH activity would be through mitogen-activated protein kinase (MAPK). There are data demonstrating that the activation of voltage-dependent calcium channels could stimulate the Ras/MAPK pathway in PC12 cells (Rosen and Greenberg, 1996). In turn, MAPK could activate TPH, either by acting on the TPH gene promoter, inducing the enzyme transcription (Wood and Russo, 2001) or by phosphorylating the CREB (Pende et al., 1997).

We have also observed that 5-HTP, 5-HT and NAS were all reduced by the blockade of the L-type calcium channels. This result could be anticipated in the light of the fact that 5-HTP is the product of the rate-limiting enzyme of the 5-HT synthesis, the tryptophan hydroxylase, and that this, in turn, gives rise to the substrates of AA-NAT and HIOMT, thus expectedly leading to a reduction of the melatonin synthesis (Sugden, 1989).

5-HT release was accordingly also reduced by nifedipine. High concentrations of 5-HT are present in structural vesicles, as well as in the cytoplasm (Juillard and Collin, 1980). The activation of α_1-adrenergic receptors promotes the exocytosis of serotonin contained in the cytoplasmatic vesicles. This indolamine has a paracrine excitatory action on pineal 5-HT_{2c}-type serotonin receptors, increasing melatonin synthesis (Steardo et al., 2000; Sugden, 1990). Therefore, the reduction of serotonin release by nifedipine may have, probably, potentiated the already diminished melatonin synthesis by TPH inhibition.

In summary, this study demonstrated that the L-type high voltage-activated calcium channels of the rat pineal gland have a role on the regulation of melatonin synthesis and release stimulated by norepinephrine, by modulating the activity of the tryptophan hydroxylase enzyme.

Acknowledgements

This work was supported by FAPESP grants (00/08258-7 and 04/06767-2). R.B. was supported by a doctoral fellowship from FAPESP (99/12661-2) and INSERM.

References

Afeche, S.C., Barbosa, R., Scialfa, J.H., Terra, I.M., Cassola, A.C., Cipolla-Neto, J., 2006. Effects of the blockade of high voltage-activated calcium channels on in vitro pineal melatonin synthesis. Cell Biochemistry and Function 24, 499–505.

Axelrod, J., Weissbach, H.L., 1961. Purification and properties of hydroxyindole-O-methyltransferase. Journal of Biological Chemistry 236, 211–213.

Banik, U., Wang, G.A., Wagner, P.D., Kaufman, S., 1997. Interaction of phosphorylated tryptophan hydroxylase with 14-3-3 proteins. Journal of Biological Chemistry 272, 26219–26225.

Chik, C.L., Liu, Q.Y., Li, B., Karpinski, E., Ho, A.K., 1995. cGMP inhibits L-type Ca2+ channel currents through protein phosphorylation in rat pinealocytes. Journal of Neuroscience 15, 3104–3109.

Chik, C.L., Liu, Q.Y., Li, B., Klein, D.C., Zylka, M., Kim, D.S., Chin, H., Karpinski, E., Ho, A.K., 1997. Alpha 1D L-type Ca(2+)-channel currents: inhibition by a beta-adrenergic agonist and pituitary adenylate cyclase-activating polypeptide (PACAP) in rat pinealocytes. Journal of Neurochemistry 68, 1078–1087.

Chin, H., Smith, M.A., Kim, H.L., Kim, H., 1992. Expression of dihydropyridine-sensitive brain calcium channels in the rat central nervous system. FEBS Letters 299, 69–74.

Darvish, N., Russell, J.T., 1998. Neurotransmitter-induced novel modulation of a nonselective cation channel by a cAMP-dependent mechanism in rat pineal cells. Journal of Neurophysiology 79, 2546–2556.

Deguchi, T., Axelrod, J., 1972. Sensitive assay for serotonin *N*-acetyltransferase activity in rat pineal. Analytical Biochemistry 50, 174–179.

Ehret, M., Cash, C.D., Hamon, M., Maitre, M., 1989. Formal demonstration of the phosphorylation of rat brain tryptophan hydroxylase by calcium/calmodulin-dependent protein kinase. Journal of Neurochemistry 52, 1886–1891.

Ehret, M., Pevet, P., Maitre, M., 1991. Tryptophan hydroxylase synthesis is induced by 3′,5′-cyclic adenosine monophosphate during circadian rhythm in the rat pineal gland. Journal of Neurochemistry 57, 1516–1521.

Ganguly, S., Gastel, J.A., Weller, J.L., Schwartz, C., Jaffe, H., Namboodiri, M.A., Coon, S.L., Hickman, A.B., Rollag, M., Obsil, T., Beauverger, P., Ferry, G., Boutin, J.A., Klein, D.C., 2001. Role of a pineal cAMP-operated arylalkylamine N-acetyltransferase/14-3-3-binding switch in melatonin synthesis. Proceedings of the National Academy of Sciences of the United States of America 98, 8083–8088.

Gomperts, B.D., Kramer, I.M., Tatham, P.E.R., 2002. Signal Transduction. Academic Press, San Diego.

Hille, B., 2001. Ion Channels of Excitable Membranes, 3rd ed. Sinauer Ass., Sunderland.

Ichimura, T., Isobe, T., Okuyama, T., Yamauchi, T., Fujisawa, H., 1987. Brain 14-3-3 protein is an activator protein that activates tryptophan 5-monooxygenase and tyrosine 3-monooxygenase in the presence of Ca2+,calmodulin-dependent protein kinase II. FEBS Letters 219, 79–82.

Johansen, P.A., Jennings, I., Cotton, R.G., Kuhn, D.M., 1995. Tryptophan hydroxylase is phosphorylated by protein kinase A. Journal of Neurochemistry 65, 882–888.

Juillard, M.T., Collin, J.P., 1980. Pools of serotonin in the pineal gland of the mouse: the mammalian pinealocyte as a component of the diffuse neuroendocrine system. Cell and Tissue Research 213, 273–291.

Klein, D.C., Berg, G.R., Weller, J., 1970. Melatonin synthesis: adenosine 3′,5′-monophosphate and norepinephrine stimulate *N*-acetyltransferase. Science 168, 979–980.

Klein, D.C., Ganguly, S., Coon, S.L., Shi, Q., Gaildrat, P., Morin, F., Weller, J.F., Obsil, T., Hickman, A., Dyda, F., 2003. 14-3-3 proteins in pineal photoneuroendocrine transduction: how many roles? Journal of Neuroendocrinology 15, 370–377.

Lee, S.Y., Choi, B.H., Hur, E.M., Lee, J.H., Lee, S.J., Lee, C.O., Kim, K.T., 2006. Norepinephrine activates store-operated Ca2+ entry coupled to large-conductance Ca2+-activated K+ channels in rat pinealocytes. American Journal of Physiology. Cell Physiology 290, C1060–C1066.

Letz, B., Schomerus, C., Maronde, E., Korf, H.W., Korbmacher, C., 1997. Stimulation of a nicotinic ACh receptor causes depolarization and activation of L-type Ca2+ channels in rat pinealocytes. Journal of Physiology 499 (Pt 2), 329–340.

Maronde, E., Wicht, H., Tasken, K., Genieser, H.G., Dehghani, F., Olcese, J., Korf, H.W., 1999. CREB phosphorylation and melatonin biosynthesis in the rat pineal gland: involvement of cyclic AMP dependent protein kinase type II. Journal of Pineal Research 27, 170–182.

Parekh, A.B., Putney Jr., J.W., 2005. Store-operated calcium channels. Physiological Review 85, 757–810.

Parfitt, A., Weller, J.L., Klein, D.C., Sakai, K.K., Marks, B.H., 1975. Blockade by ouabain or elevated potassium ion concentration of the adrenergic and adenosine cyclic 3′,5′-monophosphate-induced stimulation of pineal serotonin *N*-acetyltransferase activity. Molecular Pharmacology 11, 241–255.

Parfitt, A., Weller, J.L., Klein, D.C., 1976. Beta adrenergic-blockers decrease adrenergically stimulated *N*-acetyltransferase activity in pineal glands in organ culture. Neuropharmacology 15, 353–358.

Patel, P.D., Pontrello, C., Burke, S., 2004. Robust and tissue-specific expression of TPH2 versus TPH1 in rat raphe and pineal gland. Biological Psychiatry 55, 428–433.

Pende, M., Fisher, T.L., Simpson, P.B., Russell, J.T., Blenis, J., Gallo, V., 1997. Neurotransmitter-and growth factor-induced cAMP response element binding protein phosphorylation in glial cell progenitors: role of calcium ions, protein kinase C, and mitogen-activated protein kinase/ribosomal S6 kinase pathway. Journal of Neuroscience 17, 1291–1301.

Ribelayga, C., Gauer, F., Calgari, C., Pevet, P., Simonneaux, V., 1999. Photoneural regulation of rat pineal hydroxyindole-*O*-methyltransferase (HIOMT) messenger ribonucleic acid expression: an analysis of its complex relationship with HIOMT activity. Endocrinology 140, 1375–1384.

Ribelayga, C., Pevet, P., Simonneaux, V., 1997. Adrenergic and peptidergic regulations of hydroxyindole-*O*-methyltransferase activity in rat pineal gland. Brain Research 777, 247–250.

Roseboom, P.H., Coon, S.L., Baler, R., McCune, S.K., Weller, J.L., Klein, D.C., 1996. Melatonin synthesis: analysis of the more than 150-fold nocturnal increase in serotonin *N*-acetyltransferase messenger ribonucleic acid in the rat pineal gland. Endocrinology 137, 3033–3045.

Roseboom, P.H., Klein, D.C., 1995. Norepinephrine stimulation of pineal cyclic AMP response element-binding protein phosphorylation: primary role of a beta-adrenergic receptor/cyclic AMP mechanism. Molecular Pharmacology 47, 439–449.

Rosen, L.B., Greenberg, M.E., 1996. Stimulation of growth factor receptor signal transduction by activation of voltage-sensitive calcium channels. Proceedings of the National Academy of Sciences of the United States of America 93, 1113–1118.

Simonneaux, V., Ribelayga, C., 2003. Generation of the melatonin endocrine message in mammals: a review of the complex regulation of melatonin synthesis by norepinephrine, peptides, and other pineal transmitters. Pharmacological Review 55, 325–395.

Simonneaux, V., Rodeau, J.L., Calgari, C., Pevet, P., 1999. Neuropeptide Y increases intracellular calcium in rat pinealocytes. European Journal of Neuroscience 11, 725–728.

Steardo, L., Monteleone, P., Trabace, L., Cannizzaro, C., Maj, M., Cuomo, V., 2000. Serotonergic modulation of rat pineal gland activity: in vivo evidence for a 5-Hydroxytryptamine(2C) receptor involvement. Journal of Pharmacology and Experimental Therapeutics 295, 266–273.

Sugden, A.L., Sugden, D., Klein, D.C., 1986. Essential role of calcium influx in the adrenergic regulation of cAMP and cGMP in rat pinealocytes. Journal of Biological Chemistry 261, 11608–11612.

Sugden, D., 1989. Melatonin biosynthesis in the mammalian pineal gland. Experientia 45, 922–932.

Sugden, D., 1990. 5-Hydroxytryptamine amplifies beta-adrenergic stimulation of *N*-acetyltransferase activity in rat pinealocytes. Journal of Neurochemistry 55, 1655–1658.

Sugden, D., 2003. Comparison of circadian expression of tryptophan hydroxylase isoform mRNAs in the rat pineal gland using real-time PCR. Journal of Neurochemistry 86, 1308–1311.

Wood, J.L., Russo, A.F., 2001. Autoregulation of cell-specific MAP kinase control of the tryptophan hydroxylase promoter. Journal of Biological Chemistry 276, 21262–21271.

Yu, L., Schaad, N.C., Klein, D.C., 1993. Calcium potentiates cyclic AMP stimulation of pineal arylalkylamine *N*-acetyltransferase. Journal of Neurochemistry 60, 1436–1443.

Zatz, M., Romero, J.A., 1978. Effects of calcium-free medium on the induction of serotonin *N*-acetyltransferase in the rat pineal. Biochemical Pharmacology 27, 2549–2553.
