# Supplementary material for: 5-Hydroxytryptophan (5-HTP): Natural Occurrence, Analysis, Biosynthesis, Biotechnology, Physiology and Toxicology
Source: Int J Mol Sci. 2020 Dec 26;22(1):181. doi: 10.3390/ijms22010181 (PMC7796270; doi:10.3390/ijms22010181)
Supplement: Supplementary file 1 [file ijms-22-00181-s001.zip › 5-HTP.Data/PDF/0485937316/Wang-2018-Metabolic-pathway-engineering-for-h.pdf]

Metabolic Engineering 48 (2018) 279–287

Contents lists available at ScienceDirect

Metabolic Engineering

journal homepage: www.elsevier.com/locate/meteng

Metabolic pathway engineering for high-level production of 5-hydroxytryptophan in *Escherichia coli*

Haijiao Wang[a,b], Wenqian Liu[b], Feng Shi[c], Lei Huang[b], Jiazhang Lian[a,b,*], Liang Qu[a], Jin Cai[b], Zhinan Xu[a,b,c,*]

[a] *Key Laboratory of Biomass Chemical Engineering (Education Ministry), College of Chemical and Biological Engineering, Zhejiang University, Hangzhou 310027, China*
[b] *Institute of Biological Engineering, College of Chemical and Biological Engineering, Zhejiang University, Hangzhou 310027, China*
[c] *Shandong Institute for Food and Drug Control, Jinan 250101, China*

ARTICLE INFO

Keywords:
5-Hydroxytryptophan
Metabolic pathway engineering
Microbial cell factories
Co-factor regeneration

ABSTRACT

Cellular metabolic networks should be carefully balanced using metabolic engineering to produce the desired products at the industrial scale. As the precursor for the biosynthesis of the neurotransmitter serotonin, 5-hydroxytryptophan (5-HTP) is effective in treating a variety of diseases, such as depression, fibromyalgia, obesity, and cerebellar ataxia. Due to the lack of an efficient synthetic method, commercial production of 5-HTP is only achieved by extracting from the seeds of *Griffonia Smplicifolia*. This study reports efficient microbial production of 5-HTP via metabolically engineered *Escherichia coli.* Firstly, human tryptophan hydroxylase I (*TPH1*) gene was functionally expressed. For endogenous supply of the cofactor tetrahydrobiopterin (BH4), human BH4 biosynthesis and regeneration pathway was reconstituted. Whole-cell bioconversion resulted in high-level production of 5-HTP (~1.2 g/L) from 2 g/L L-tryptophan in shake flasks. Further metabolic engineering efforts were employed to achieve 5-HTP biosynthesis from simple carbon sources. The whole biosynthetic pathway was divided into three functional modules, L-tryptophan module, the hydroxylation module, and the BH4 module. By reducing the copy number of L-tryptophan module, replacing TPH1 with a more stable mutant form, and promoter regulation of the BH4 module, 5-HTP was produced at a final titer of 1.3 g/L in the shake flask and 5.1 g/L in a fed-batch fermenter with glycerol as the carbon source, both of which were the highest ever reported for microbial production of 5-HTP.

1. Introduction

5-HTP is the direct biosynthetic precursor of the neurotransmitter serotonin and has been shown to be effective in the treatment of a variety of diseases, including depression, insomnia, and chronic headaches (Birdsall, 1998; Jacobsen and Frøstrup, 2015). Currently, extraction from the seeds of *Griffonia Simplifolia* is the main commercial way of 5-HTP production (Turner et al., 2006). However, the material supply is seasonally and regionally dependent, resulting in limited output and high market price of 5-HTP.

Genetically engineered microorganisms have been successfully used to produce many important natural products (Chen et al., 2012; Huang et al., 2015; Lynch and Gill, 2012; Zhao et al., 2016) and production of 5-HTP through microbial fermentation provides a desirable alternative to plant extraction. 5-HTP is synthesized in mammalian cells through hydroxylation of L-tryptophan, catalyzed by L-tryptophan hydroxylase (TPH), with Fe^{2+} and tetrahydrobiopterin (BH4) as cofactors and O_2 as a co-substrate (Walther et al., 2003). In human, BH4 is synthesized from GTP via a three-step pathway, containing GTP cyclohydrolase I (GCHI), 6-pyruvate-tetrahydropterin synthase (PTPS), and sepiapterin reductase (SPR) (Yamamoto et al., 2003). BH4 is oxidized to pterin-4α-carbinolamine (BH3OH) during L-tryptophan hydroxylation and regenerated through the function of pterin-4α-carbinolamine dehydratase (PCD) and dihydropteridine reductase (DHPR) (Fig. 1) (Hara and Kino, 2013). The complexity of the cofactor supply system as well as the instability of human TPH in microbial hosts are the major challenges for efficient microbial production of 5-HTP. A previous patent reported the production of 0.9 mM (equivalent to 198 mg/L) 5-HTP from L-tryptophan via heterologous expression of the TPH pathway in *E. coli* (Knight et al., 2013). However, the low production efficiency and the high cost of L-tryptophan supplementation make the process not suitable for industrial production.

To bypass these problems, Lin et al. (2014) modified a prokaryotic phenylalanine 4-hydroxylase (PAH) for the conversion of L-tryptophan

* Corresponding authors at: Key Laboratory of Biomass Chemical Engineering (Education Ministry), College of Chemical and Biological Engineering, Zhejiang University, Hangzhou 310027, China.
E-mail addresses: jzlian@zju.edu.cn (J. Lian), znxu@zju.edu.cn (Z. Xu).

https://doi.org/10.1016/j.ymben.2018.06.007
Received 2 March 2018; Received in revised form 27 May 2018; Accepted 18 June 2018
Available online 19 June 2018
1096-7176/ © 2018 International Metabolic Engineering Society. Published by Elsevier Inc. All rights reserved.

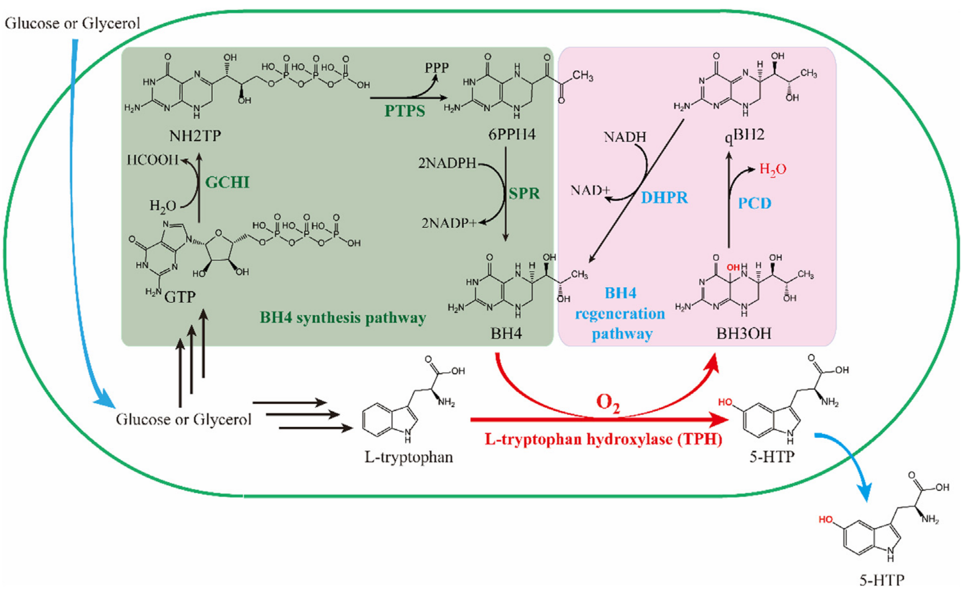

Fig. 1. Pathway for the synthesis of 5-HTP catalyzed by L-tryptophan hydroxylase with tetrahydrobiopterin (BH4) as a cofactor. BH4 is synthesized from GTP by GTP cyclohydrolase I (GCHI), 6-pyruvoyl-tetrahydropterin synthase (PTPS) and sepiapterin reductase (SPR) respectively. The regeneration system is composed of pterin-4a-carbinolamine dehydratase (PCD) and dihydropteridine reductase (DHPR).

to 5-HTP in *E. coli*. With the introduction of a foreign tetrahydromonapterin (MH4) recycling mechanism, the endogenous MH4 could be utilized as a cofactor for L-tryptophan hydroxylation. Coupled with metabolic engineering efforts, ~1.1 g/L and ~150 mg/L of 5-HTP could be produced from L-tryptophan and glucose, respectively. Microbial production of 5-HTP was also achieved by introducing a salicylate 5-hydroxylase into *E. coli*. The salicylate 5-hydroxylase catalyzed the conversion of anthranilate, the precursor for tryptophan biosynthesis, to 5-hydroxyanthranilate (5-HI), which was further converted to 5-HTP using the native L-tryptophan biosynthetic pathway. 98.09 ± 3.24 mg/L of 5-HTP was produced through a two-step fermentation strategy (Sun et al., 2015).

In this work, we constructed the L-tryptophan hydroxylation pathway by co-expressing human TPH and BH4 biosynthesis and regeneration system in *E. coli*, which allowed the conversion of L-tryptophan to 5-HTP without exogenous supplementation of the pterin cofactor. Further introduction of the L-tryptophan synthesis pathway resulted in bioproduction of 5-HTP from simple carbon sources like glucose or glycerol. Furthermore, metabolic engineering strategies, such as plasmid copy number and promoter strengths modulation to balance metabolic fluxes, were employed to improve 5-HTP titer (5.1 g/L) to the highest ever reported in literature. This process holds great potential for scale-up and provides a commercially attractive way of microbial production of 5-HTP.

2. Materials and methods

2.1. Strains and culture conditions

All bacterial strains and plasmids used in this study were listed in Table 1. *E. coli* DH5α was used for plasmid construction and propagation (Yang et al., 2014) and BL21(DE3) was used for protein expression and *in vivo* hydroxylation of L-tryptophan to 5-HTP. As for biosynthesis of 5-HTP, *tnaA* gene was knocked out in BL21(DE3) using the previously established protocol (Datsenko and Wanner, 2000).

Luria-Bertani (LB) medium was used for cell cultivation and enzyme expression (Tang et al., 2016). Modified M9 medium (M9Y) was used for *in vivo* hydroxylation of L-tryptophan to 5-HTP (Lin et al., 2014). Mineral medium containing glucose or glycerol at the indicated concentration, 2 g/L yeast extract, 4 g/L $(NH4)_2HPO_4$, 13.5 g/L KH_2PO_4, 1.4 g/L $MgSO_4{\cdot}7H_2O$, 1.7 g/L citric acid, 5 mL/L 200 × trace elements, and 30 mg/L thiamine was used for production of 5-HTP in shake flasks and a fed-batch fermenter. 200 × trace elements contains 20 g/L $FeSO_4{\cdot}7H_2O$, 4 g/L $CaCl_2$, 4.4 g/L $ZnSO_4{\cdot}7H_2O$, 1 g/L $MnSO_4{\cdot}4H_2O$, 2 g/L $CuSO_4{\cdot}5H_2O$, 0.2 g/L $(NH_4)_6Mo_7O_4{\cdot}4H_2O$, and 0.04 g/L $Na_2B_4O_7{\cdot}10H_2O$ (Luo et al., 2013). When necessary, the medium was supplemented with kanamycin, ampicillin, chloramphenicol, or tetracycline at appropriate concentrations. BH4 was purchased from BioChenPartner, Shanghai, China.

2.2. DNA manipulation

Primer sequences used for PCR amplification were listed in Table S1. Human *TPH1*, *TPH2*, *PTPS*, *SPR*, *PCD* and *DHPR* were all codon optimized and synthesized by Biosune (Shanghai, China). The *E. coli folE* gene and *Bacillus subtilus mtrA* gene, both encoding GCHI, were amplified from their corresponding genomic DNAs.

2.2.1. Construction of TPH expressing plasmids

TPH1, *folE* and *mtrA* genes were inserted into plasmid pET28a using restriction sites *Nco*I and *Xho*I to construct plasmids pET28a-TPH1, pET28a-folE, and pET28a-mtrA, respectively. Truncated forms of *TPH* genes, *TPH99* (*TPH1* mutant with a deletion of first 99 N-terminal and last 24 C-terminal amino acids, NΔ99/CΔ24) and *TPH145* (*TPH2* mutant with a deletion of first 145 N-terminal and 24 C-terminal amino acids, NΔ145/CΔ24) genes were also inserted into pET28a between *Nco*I and *Xho*I to construct pET28a-TPH99 and pET28a-TPH145, respectively.

2.2.2. Construction of BH4 supplementation and L-tryptophan hydroxylation plasmids

PTPS and *SPR* were inserted into pACYCDuet-1 using restriction sites *Nco*I/*Bam*HI and *Bgl*II/*Xho*I to generate pACYC-PS. *folE* gene was amplified from pET28a-folE using primer pair T7_folE-TPH_*Eco*RI/T7_folE-TPH_*Sal*I and inserted into pACYC-PS using *Eco*RI and *Sal*I to construct pACYC-FPS (Fig. S1). Plasmid pACYC-MPS was constructed in the same way except for the replacement of *folE* with *mtrA* (Fig. S2).

pETDuet-PD was constructed by inserting *DHPR* and *PCD* into pETDuet-1 using *Nco*I/*Bam*HI and *Nde*I/*Xho*I. *TPH1* was amplified from pET28a-TPH1 using the same primer pair T7_folE-TPH_*Eco*RI/T7_folE-TPH_*Sal*I and inserted into pETDuet-PD using *Eco*RI and *Sal*I to construct pETDuet-PDT1 (Fig. S3).

To construct pACHTP1 (Fig. S4), pACYC-MPS was linearized using primers inf_pAC_F/inf_pAC_R. The PDT1 segment was amplified using inf_PDT1_F/inf_PDT1_R from pETDuet-PDT1. The two fragments were circularized using ClonExpress Entry One Step Cloning Kit from Vazyme

Table 1
Bacterial strains and plasmids used in this study.

Name	Description[a]	Source or reference
**Strains**		
Bacillus subtilis	Source for *mtrA*	This lab
E. coli DH5α	Cloning host	This lab
E. coli BL21(DE3)	Protein expression host	This lab
E. coli BL21Δ*tnaA*	BL21(DE3)Δ*tnaA*	This study
E. coli BL21/FPS/PDT1	BL21(DE3)/pACYC-FPS/pETDuet-PDT1	This study
E. coli BL21/MPS/PDT1	BL21(DE3)/pACYC-MPS/pETDuet-PDT1	This study
E. coli BL21/PDT1	BL21(DE3)/pETDuet-PDT1	This study
E. coli HTP4-1	BL21Δ*tnaA*/pSGS4/pACHTP1	This study
E. coli HTP4-145	BL21Δ*tnaA*/pSGS4/pACHTP145	This study
E. coli HTP101-145	BL21Δ*tnaA*/pSA101trp/pACHTP145	This study
E. coli HTP101-LMT	BL21Δ*tnaA*/pSA101trp/pACHTP-LMT	This study
**Plasmids**		
pACYCDuet-1	P15A ori, Cm^R, *E. coli* expression vector	This lab
pACYC-PS	pACYCDuet-1, $T7_{pro}$-*PTPS*, $T7_{pro}$-*SPR*	This study
pACYC-FPS	pACYC-PS, $T7_{pro}$-*folE*	This study
pACYC-MPS	pACYC-PS, $T7_{pro}$-*mtrA*	This study
pACHTP1	pACYC-MPS, $T7_{pro}$-*PCD*, $T7_{pro}$-*TPH1*, $T7_{pro}$-*DHPR*	This study
pACHTP145	pACYC-MPS, $T7_{pro}$-*PCD*, $T7_{pro}$-*TPH145*, $T7_{pro}$-*DHPR*	This study
pACYC-LacMPS	pACYCDuet-1, Lac_{pro}-*mtrA-PTPS-SPR*	This study
pACHTP-LMT	pACYC-LacMPS, M1-93_{pro}-*PCD-DHPR*, $T7_{pro}$-*TPH145*	This study
pET28a(+)	ColE1 ori, Kan^R, *E. coli* expression vector	This lab
pET28a-folE	pET28a(+), $T7_{pro}$-*folE*	This study
pET28a-mtrA	pET28a(+), $T7_{pro}$-*mtrA*	This study
pET28a-TPH1	pET28a(+), $T7_{pro}$-*TPH1*	This study
pET28a-TPH99	pET28a(+), $T7_{pro}$-*TPH99*	This study
pET28a-TPH145	pET28a(+), $T7_{pro}$-*TPH145*	This study
pETDuet-1	pBR322 ori, Amp^R, *E. coli* expression vector	This lab
pETDuet-PD	pETDuet-1, $T7_{pro}$-*PCD*, $T7_{pro}$-*DHPR*	This study
pETDuet-PDT1	pETDuet-PD, $T7_{pro}$-*TPH1*	This study
pSA101trp	pSC101 ori, Amp^R, tac_{pro}-*aroH*[b], tac_{pro}-*trpE*[b]*DCBA*	This study
pSGS4	pBR322 ori, Tet^R, tac_{pro}-*aroH*[b], tac_{pro}-*trpE*[b]*DCBA*	(Luo et al., 2013)

[a] Abbreviations: Amp, ampicillin; Kan, kanamycin; Cm, chloramphenicol; Tet, tetracycline.

[b] Feedback inhibition eliminated by point mutation.

Biotech (Nanjing, China). Plasmid pACHTP145 was constructed following the same procedure (Fig. S5).

To construct the BH4 synthesis plasmid pACYC-LacMPS, pACYCDuet-1was linearized with primers p15ori_cat_F/p15ori_cat_R as fragment 1. The *mtrA* gene was amplified with primers Lac_mtrA_F/Lac_mtrA_R as fragment 2. *PTPS* and *SPR* were amplified using primer pairs RBS_PTPS_F/RBS_PTPS_R and RBS_SPR_F/RBS_SPR_R as fragment 3 and 4 respectively. DNA fragments 1–4 were each introduced an overlapping area at both ends by PCR and were joined via Gibson assembly to form plasmid pACYC-LacMPS (Fig. S6) (Gibson et al., 2009). Plasmid pACHTP-LMT was constructed by inserting *PCD*, *DHPR*, and *TPH145* into pACYC-LacMPS via Gibson assembly (Fig. S7). An M1–93 promoter was introduced in front of *PCD* (Lu et al., 2012) and a 5′-untranslated region in front of *DHPR*, respectively.

2.2.3. The L-tryptophan synthesis plasmids

pSGS4 was constructed by inserting an 9.2 kb fragment containing the whole tryptophan operon *trpEDCBA* and *aroH* gene into pBR322 at restriction site *Eco*RI (Fig. S8). Site-directed mutagenesis was conducted to remove the feedback regulation by L-tryptophan for *trpE* (L10Q, K71Q, N94S, and Y465C) and *aroH* (F211S) (Luo et al., 2013).

Plasmid pSA101trp was constructed by inserting *trpEDCBA* and *aroH* gene from pSGS4 and an Amp^R cassette from pUC18 into pSC101 ori via Gibson assembly (Fig. S9).

2.3. Expression of TPH1 and enzyme activity assay

E. coli BL21 (DE3) cells harboring the TPH expression plasmids, pET28a-TPH1, pET28a-TPH99 or pET28a-TPH145, were plated on a LB agar plate with 50 µg/mL kanamycin sulfate (50 Kana) and incubated at 37 °C overnight. Single colonies were inoculated into 5 mL LB (50 Kana) media and incubated at 37 °C, 200 rpm overnight in test tubes. Aliquots were taken to inoculate 50 mL LB (50 Kana) media in 250 mL shake flasks and incubated at 37 °C, 200 rpm until an optical density at 600 nm (OD_{600}) of ~0.8–1.0 was reached. The TPH expression was induced with 0.1 mM IPTG. After incubation at 25 °C, 200 rpm for 6 h, the cells were harvested by centrifugation at 4 °C, 5000 ×*g* for 10 min, washed once with ice cold 50 mM Tris-HCl, pH 7.2, with 5% (v/v) glycerol and pelleted by centrifugation.

The cell pellets from 50 mL of induced bacterial culture were suspended in 25 mL of 50 mM Tris-HCl, pH 7.2, with 5% (v/v) glycerol. Cells were lysed by sonication on ice (20 pulses of 3 s separated by intervals of 8 s) at 200 W. Cell lysates were centrifuged at 4 °C, 12,000 ×*g* for 10 min. The supernatant was used as crude enzyme for direct enzymatic assays.

The TPH activities were assayed at 37 °C in a standard reaction mixture of 2 mL, containing 50 mM HEPES/NaOH, pH 7.2, 200 mM $(NH_4)_2SO_4$, 7 mM dithiothreitol (DTT), 50 µM $(NH_4)_2Fe(SO_4)_2$, and 0.025 g/L catalase. The standard substrate concentrations were 200 µM L-tryptophan and 300 µM BH4. The temperature was maintained at 37 °C by water bath.

2.4. Hydroxylation of L-tryptophan by resting cells with external BH4 supplementation

The cell pellets from 50 mL of induced bacterial culture were suspended with ice-cold 20 mM HEPES/NaOH, pH 7.0, 100 mM $(NH_4)_2SO_4$, and 5% glycerol. Cell catalysis conditions were the same as the TPH enzymatic assay. The test tubes containing the reaction mixture were incubated at 37 °C, 200 rpm for 60 min. Samples were taken and cells were removed by centrifugation. The concentrations of 5-HTP and remaining L-tryptophan were quantitatively measured via high-performance liquid chromatography (HPLC).

2.5. Hydroxylation of L-tryptophan by resting cells without external BH4 supplementation

E. coli BL21(DE3) was transformed with plasmids pACYC-FPS (or pACYC-MPS) and pETDuet-PDT1. The culture conditions were the same as described above and cells were harvested by centrifugation.

Various amount of cells from induced cultures were suspended in 20 mL M9Y medium containing 2 g/L tryptophan and left to grow at 30 °C, 200 rpm. Samples were taken every 4 h. The concentrations of 5-HTP and L-tryptophan were analyzed by HPLC.

2.6. Production of 5-HTP in E. coli without external L-tryptophan supplementation

5-HTP producing strains (HTP4-1, HTP4–145, HTP101–145, HTP101-LMT) were plated on LB agar plates. Single colonies were picked into 5 mL of LB media and incubated at 37 °C, 200 rpm overnight. Aliquots were inoculated into 50 mL glycerol mineral media in 250 mL shake flasks and incubated at 37 °C, 200 rpm until OD_{600} ~ 5 was reached. The cells were induced by adding IPTG to the culture to a

final concentration of 0.01 mM and further cultivated at 30 °C, 200 rpm. Samples were taken every few hours. The OD_{600} values were measured and the concentration of the products were analyzed by HPLC.

2.7. Fed-batch fermentation of E. coli HTP101-LMT for efficient 5-HTP production

A single colony of *E. coli* HTP101-LMT was inoculated into 50 mL LB medium and incubated at 37 °C, 200 rpm overnight. Then, 5% (v/v) of such culture was transferred into a 2000 mL shake flask containing 400 mL LB medium and grew at 37 °C, 200 rpm. When the culture reached an OD_{600} of ~2.5, 5% (v/v) inoculums were transferred into a 10 L bioreactor (BIOTECH-10JS-10JS; Shanghai Baoxing Bio-Engineering Equipment Co. Ltd., Shanghai, China) with 7 L working volume. The temperature was set at 37 °C at the beginning. When OD_{600} reached ~15, the temperature was changed to 30 °C, and the culture was induced with 0.01 mM IPTG. The fermentation pH was maintained at 6.8 with ammonium hydroxide throughout the process. The dissolved oxygen level was maintained at 10–20% saturation by agitation and aeration. After the initial 30 g/L glycerol was consumed, a mixture solution of 400 g/L glycerol and 10 g/L yeast extract was gradually fed into the broth. Samples were taken every 2 h for cell density (OD_{600}) measurement and HPLC analysis.

2.8. HPLC analysis

L-tryptophan from Sangon Biotech (Shanghai, China) and 5-HTP from Aladdin (Shanghai, China) were used as standards. Both the standards and samples were quantified by HPLC (Agilent 1100 series with a UV absorption detector and a reverse phase Agilent TC-C18 (2) column). The mobile phase was 10 mM potassium phosphate buffer, pH 6.5, with 7.5% methanol. Quantification of tryptophan and 5-HTP was based on their absorbance at 276 nm.

3. Results

3.1. Hydroxylation of L-tryptophan by resting cells with external BH4 supplementation

TPH1 was firstly expressed in *E. coli* BL21(DE3) and the enzymatic activity was measured (Fig. S10). Then we tested the conversion of L-tryptophan to 5-HTP using resting cells. When 8.2 mg DCW/mL cells and 3 mM BH4 were added to the reaction, 2 mM L-tryptophan was sufficiently converted to 5-HTP in 1 h, with the molar yield as high as 97.1% (Fig. 2A). In a larger scale (32 mL) experiment containing 10 mM L-tryptophan (~2 g/L) and 12 mM BH4, 8.6 mg DCW/mL cells were able to produce 2.1 g/L 5-HTP in 2 h (Fig. 2B). The resting cell hydroxylation experiment showed a high reaction efficiency and molar yield for human TPH1. However, high biomass was required and the expensive co-factor BH4 was added exogenously in this reaction.

3.2. Hydroxylation of L-tryptophan by resting cells without external BH4 supplementation

After *TPH1* was functionally expressed, we focused on the co-factor biosynthesis and regeneration pathways in *E. coli*. To achieve hydroxylating L-tryptophan without external addition of the expensive co-factor, human BH4 biosynthesis and regeneration pathways were introduced by pACYC-FPS (or pACYC-MPS) and pETDuet-PDT1, respectively.

E. coli strains BL21/PDT1, BL21/FPS/PDT1 and BL21/MPS/PDT1 were precultured and induced for protein expression in LB. Cells were collected and resuspended in M9Y media (3.0 mg DCW /mL) with 2.0 g/L L-tryptophan. Hardly any 5-HTP was produced in BL21/PDT1 due to a lack of the cofactor. However, when 500 μM BH4 was added in the medium, 400 mg/L (1.8 mM) of 5-HTP was produced in 24 h,

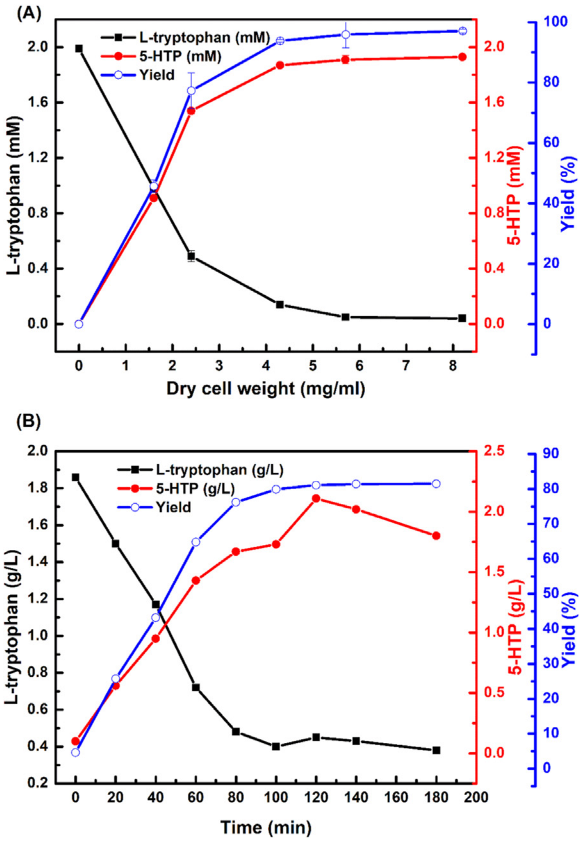

Fig. 2. Hydroxylation of L-tryptophan to 5-HTP with TPH1 expressing cells. Black and red lines indicate the consumption of L-tryptophan and the production of 5-HTP, respectively. Dashed blue line indicates the molar yield of 5-HTP from L-tryptophan. (A) Hydroxylation of L-tryptophan to 5-HTP using different amount of biomass. The reaction conditions were as described in materials and methods. Mixtures were incubated at 37 °C, 200 rpm for 60 min (B) Production of 5-HTP over time. The initial concentration of L-tryptophan and BH4 were 10 mM (2 g/L) and 6 mM respectively in 32 mL solution. Another 6 mM BH4 was supplemented at 40 min. The cell concentration was at OD_{600} ~ 23, which corresponded to 8.6 mg DCW/mL. Aliquots of solution were taken and diluted for HPLC analysis every 20 min.

indicating that BH4 was crucial for TPH1 activity and the BH4 regeneration pathway in pETDuet-PDT1 was functional. When BH4 biosynthesis and regeneration pathways were introduced, BL21/FPS/PDT1 and BL21/MPS/PDT1 could convert L-tryptophan to 5-HTP in the co-factor-free medium. BL21/FPS/PDT1 had a similar profile as BL21/PDT1 with 500 μM BH4, although 5-HTP productivity was slightly lower. 5-HTP productivity of BL21/MPS/PDT1 and BL21/FPS/PDT1 are comparable at the beginning, but was only constantly maintained in BL21/MPS/PDT1, with ~600 mg/L of 5-HTP produced in 24 h (Fig. 3A). As for BL21/MPS/PDT1, the initial conversion rates showed positive correlation with cell concentration, with 907.1, 1236.3, and 1199.1 mg/L of 5-HTP produced from 3.8, 6.8, and 10.2 mg DCW/mL cells, respectively (Fig. 3B).

5-HTP production in BL21/PDT1 (with 500 μM BH4) and BL21/FPS/PDT1 was ceased after 15 h. Presumably, the low activity or stability of the BH4 regeneration pathway resulted in a lack of the reduced form of BH4 for L-tryptophan hydroxylation. Interestingly, 5-HTP production in BL21/MPS/PDT1 was steadily maintained for more than 24 h (Fig. 3A&B). Given that the replacement of *folE* by *mtrA* in pACYC-MPS could help to improve BH4 biosynthesis (Yamamoto et al., 2003), this relatively prolonged hydroxylation activity might result from extra BH4 production. Fig. 3C showed a time-course protein expression profiles of BL21/MPS/PDT1 during L-tryptophan hydroxylation. Five out of six proteins showed a clear band on SDS-PAGE gel except for

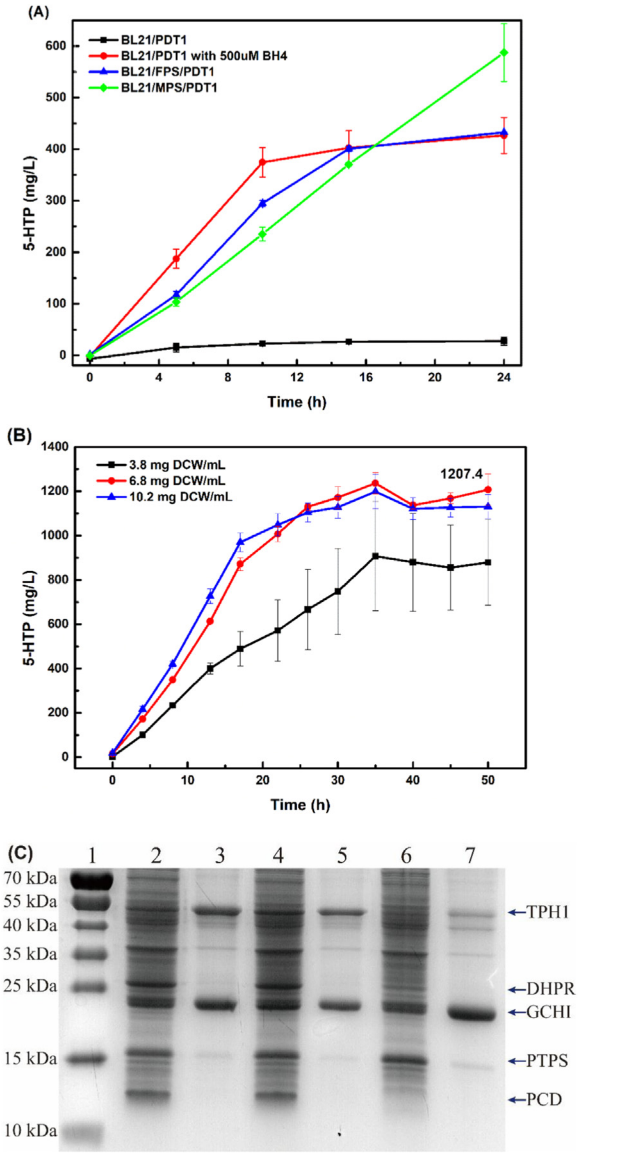

Fig. 3. (A) Whole-cell bioconversion of L-tryptophan into 5-HTP using BL21(DE3)/PDT1, BL21(DE3)/FPS/PDT1 and BL21(DE3)/MPS/PDT1. (B) Whole-cell bioconversion with different initial cell densities of BL21(DE3)/MPS/PDT1. (C) Protein expression in BL21(DE3)/MPS/PDT1. Lane 1. Marker. Lane 2 & 3. Soluble and inclusion expression of BL21(DE3)/MPS/PDT1 induced in LB for 8 h. Lane 4 & 5. Soluble and inclusion expression after 24 h of catalysis. Lane 6 & 7. Soluble and inclusion expression after 48 h of catalysis.

SPR. After 48 h of hydroxylation, PCD and DHPR, the two BH4 regeneration enzymes, were almost completely degraded (Fig. 3C), demonstrating their low stability.

3.3. Production of 5-HTP in E. coli without external L-tryptophan supplementation

After achieving the efficient bioconversion of L-tryptophan to 5-HTP, we proceeded with the construction of a 5-HTP-producing strain based on endogenous L-tryptophan biosynthesis from simple carbon sources (Fig. 1). *E. coli* BL21Δ*tnaA* was transformed with plasmids pSGS4 and pACHTP1 to obtain strain HTP4-1, which produced 49.7 mg/L 5-HTP and 440.2 mg/L L-tryptophan after 60 h′ cultivation in LB media. However, the titers dropped when HTP4-1 was cultivated in M9Y media (29.2 mg/L for 5-HTP and 124.1 mg/L for tryptophan, Fig. S11), probably due to insufficient protein expression and cell growth in M9Y. However, when cultivated in mineral medium with 30 g/L glycerol and induced by 0.05 mM IPTG, this strain could produce 73.1 mg/L 5-HTP and 787.1 mg/L L-tryptophan in 72 h (Fig. S12). Then the induction temperature, IPTG concentration, induction time, and the concentration of antibiotics were optimized (Fig. S13-S16). Under optimal conditions, HTP4-1 strain was able to produce 314.8 mg/L 5-HTP and 3971.8 mg/L L-tryptophan in 116 h of flask fermentation (Fig. 4A). High induction level (more than 0.05 mM of IPTG) appeared to impair cell growth with a long growth recovery period after induction, which might be caused by heavy protein expression burden. After the optimization, constant cell growth was restored and a higher 5-HTP productivity was achieved (Fig. S14). High induction temperature (34 °C, 37 °C) and tetracycline concentration (20 µg/mL) also exhibited negative effects on cell growth or 5-HTP production (Fig. S13 & S16). Induction at log phase resulted in the highest 5-HTP production (Fig. S15).

3.4. Improvement of 5-HTP production with combined metabolic engineering

As shown in Fig. 4A, the accumulation of L-tryptophan was more than 10 times higher than that of 5-HTP, probably due to the unbalanced metabolic fluxes of the L-tryptophan hydroxylation pathway and the L-tryptophan biosynthesis pathway. Therefore, the whole biosynthetic pathways were divided into three functional modules, the L-tryptophan module, the hydroxylation module, and the BH4 module, which should be fine-tuned to achieve optimal 5-HTP production.

3.4.1. Expression of TPH mutants with improved solubility and activity

TPH was well known for its low activity and solubility in bacterial host, resulting in low conversion efficiency of L-tryptophan to 5-HTP (McKinney et al., 2001; Winge et al., 2007). Several studies have been reported on the soluble and stable expression of TPH isoforms in *E. coli* (Murphy et al., 2008; Nielsen et al., 2008). For example, the truncated forms of human TPH were found to have higher solubility and stability (Carkaci-Salli et al., 2006; Murphy et al., 2008). Inspired by these studies, two truncated mutants of human tryptophan hydroxylases, TPH99 (NΔ99/CΔ24 of TPH1) and TPH145 (NΔ145/CΔ24 of TPH2), were constructed and determined to dramatically improve TPH solubility. While the full length TPH1 and TPH2 were mainly expressed as inclusion bodies, TPH99 and TPH145 were exclusively soluble (Fig. 5A). Accordingly, L-tryptophan hydroxylase activities in cell lysates were significantly increased (Fig. 5B). Due to higher solubility and activity, TPH145 was chosen to replace TPH1 in plasmid pACHTP1 to construct pACHTP145 (Fig. S5). HTP4–145 harboring pSGS4 and pACHTP145 produced 424.7 mg/L of 5-HTP, which was 34.9% higher than that with HTP4-1, while L-tryptophan production was slightly decreased (Fig. 4B).

3.4.2. Copy number reduction of L-tryptophan synthesis module

Since the intermediate L-tryptophan remained a high concentration and its biosynthesis consumed too much cellular resources, 5-HTP production might be improved by reducing L-tryptophan production. To test this hypothesis, the tryptophan operon *trpEDCBA* and *aroH* gene were cloned into a low copy number plasmid, yielding pSA101trp (Fig. S9). As expected, L-tryptophan production (207.9 mg/L) was drastically decreased in HTP101–145 harboring pSA101trp and pACHTP145. Meanwhile, 5-HTP production was increased to 532.6 mg/L, 25.4% improvement compared with HTP4–145 (Fig. 4C), indicating that the metabolic flux was partially redirected from L-tryptophan synthesis to hydroxylation. However, the overall production of L-tryptophan and 5-HTP was significantly decreased, indicating that some other factors (i.e. the BH4 biosynthesis and regeneration module) should be further optimized to enhance 5-HTP production.

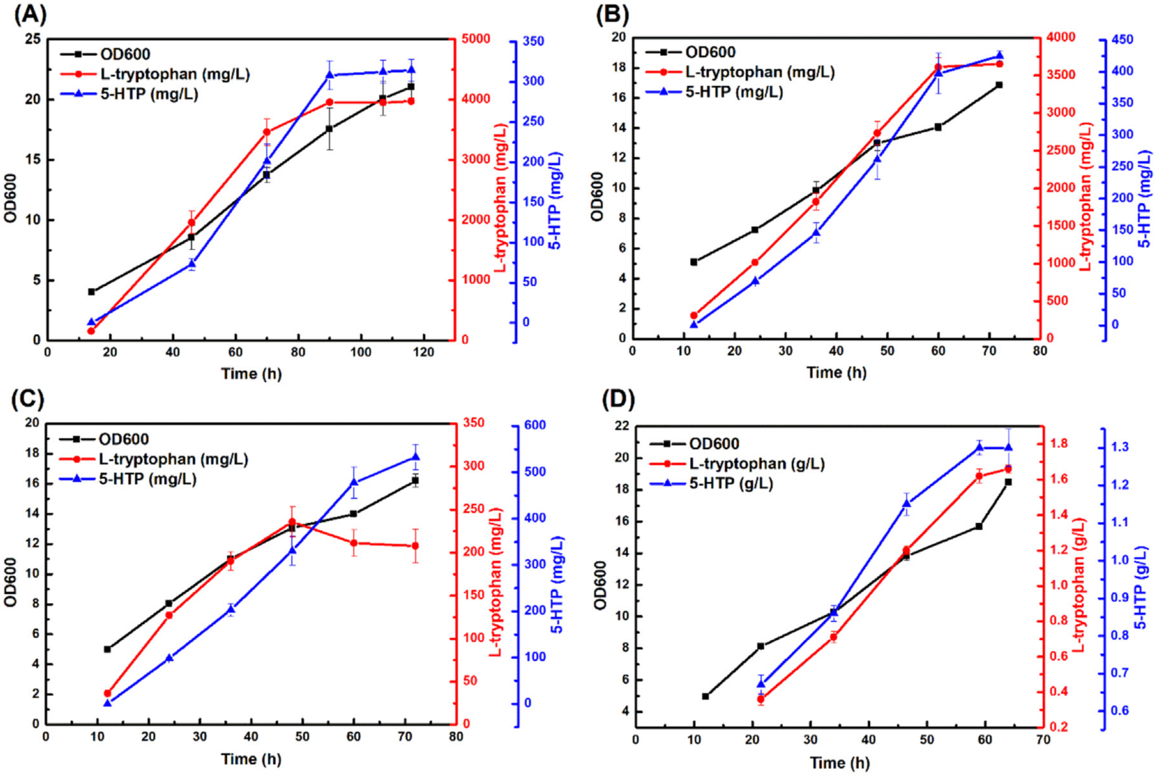

Fig. 4. Production of 5- HTP in the shake flasks with *E. coli* BL21Δ*tnaA* strains. (A) *E. coli* HTP4-1 (C) *E. coli* HTP4–145. (D) *E. coli* HTP101–145 (D) *E. coli* HTP101-LMT.

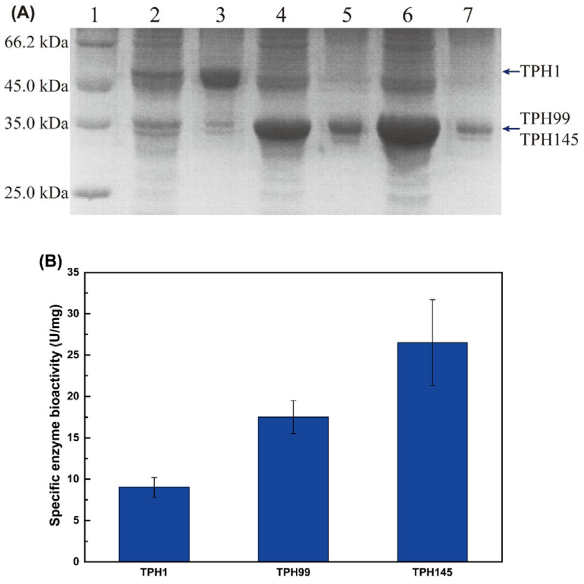

Fig. 5. (A) Protein expression of three types of L-tryptophan hydroxylases in BL21(DE3). Lane 1. Marker. Lane 2 & 3. Soluble and inclusion expression of TPH1. Lane 4 & 5. Soluble and inclusion expression of TPH99. Lane 6 & 7. Soluble and inclusion expression of TPH145. (B) Comparison of specific enzyme bioactivity among three different L-tryptophan hydroxylases. TPH99 was a TPH1 mutant with 99 N-terminus amino acids and 24 C-terminus amino acids deleted. TPH145 was a TPH2 mutant with 145 N-terminus amino acids and 24 C-terminus amino acids deleted. The standard of one unit enzyme activity was defined as the enzyme needed to produce 1 μg 5-HTP in 1 min. Specific enzyme bioactivity was calculated with total cellular lysate protein.

3.4.3. Combinatory regulation of promoter strength for the synthesis and recovery of BH4

The L-tryptophan hydroxylation plasmids pACHTP1 and pACHTP145 were initially constructed with each gene driven by a T7 promoter to ensure sufficient expression (Fig. S4 & S5). However, the excessive expression of the BH4 synthetic and recycling pathway genes might result in high protein expression burdens. Therefore, a new hydroxylation plasmid pACHTP-LMT was designed and constructed (Fig. S7). In this plasmid, the BH4 biosynthesis pathway genes were cloned in a polycistronic structure driven by a *lac* promoter. The BH4 regeneration pathway genes were expressed in a double cistron led by the M1–93 promoter (Lu et al., 2012), and TPH145 was still expressed by the T7 promoter. 5′-Untranslated regions containing ribosome-binding sites (RBSs) were inserted in front of *PTPS*, *SPR*, and *DHPR* to ensure sufficient translation in pACHTP-LMT (Fig. S7) (Salis et al., 2009; Zelcbuch et al., 2013).

The final strain HTP101-LMT, harboring pSA101trp and pACHTP-LMT, showed a significant increase in both 5-HTP and L-tryptophan production (Fig. 4D). In 59 h, HTP101-LMT produced 1.3 g/L 5-HTP, which was 2.4-fold and around 44-fold higher than HTP101–145 and the original strain HTP4-1 in M9Y medium, respectively (Fig. 7), and represented the highest titer ever reported in flask. Surprisingly, compared with HTP101–145, L-tryptophan production was also significantly increased from 207.9 mg/L to 1.7 g/L. The sharp increase of both 5-HTP and L-tryptophan indicated the dramatically alleviated cellular metabolic burdens. In other words, the decrease in protein expression levels converted a considerable amount of carbon fluxes from protein synthesis to L-tryptophan and 5-HTP production.

3.5. Highly efficient production of 5-HTP with E. coli HTP101-LMT using fed-batch fermentation

The production of 5-HTP was evaluated with *E.coli* strain HTP101-LMT in a 10-L bioreactor with 7 L working volume. The results (Fig. 6)

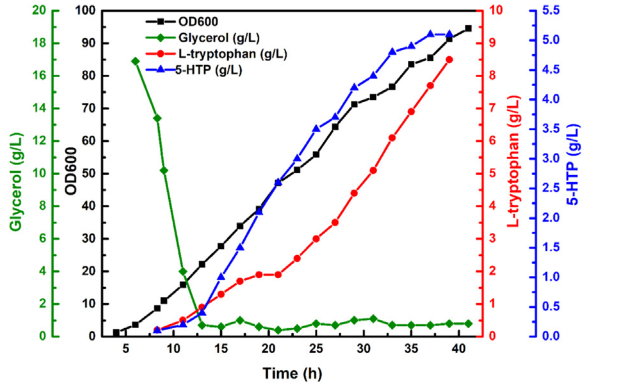

Fig. 6. Production of 5-HTP with *E. coli* HTP101-LMT in a 10 L bioreactor with 7 L working volume.

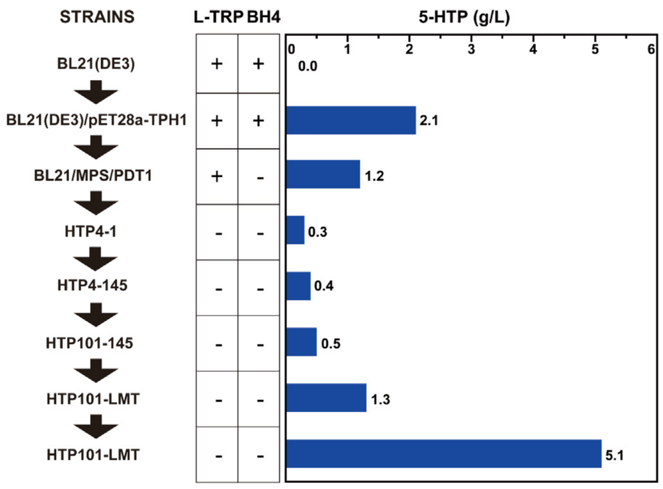

Fig. 7. A schematic summary of metabolic engineering in *E. coli* for 5-HTP production. BL21(DE3) was used as a negative control for L-tryptophan hydroxylation by BL21(DE3)/pET28a-TPH1 resting cells where 10 mM (~2 g/L) L-tryptophan and 12 mM BH4 were externally added. No 5-HTP peak was detected on HPLC for BL21(DE3). For L-tryptophan hydroxylation by BL21/MPS/PDT1 without BH4 addition, 2 g/L L-tryptophan was supplemented. 5-HTP productions by *E. coli* strains HTP4-1, HTP4–145, HTP101–145, and HTP101-LMT were performed in shake flasks, while fed-batch fermentation was performed for HTP101-LMT. L-TRP: abbreviation of L-tryptophan.

showed that glycerol concentration could be controlled under 1 g/L during most of the fermentation process by constant glycerol feeding. Cellular growth was steadily increased, and the production of 5-HTP could be increased to as high as 5.1 g/L, which represented the highest titer ever reported. It was also concluded in Fig. 6 that the biosynthesis of L-tryptophan was cell density dependent, and the production of 5-HTP was subjected to IPTG induction and would drop down in the late fermentation stage. Therefore, extensive optimization of the fermentation conditions would be necessary to further improve the productivity of 5-HTP.

4. Discussion

Heterologous production of 5-HTP by metabolically engineered microorganisms normally have two different strategies. One is through the combination of genes or pathways from a variety of organisms to create an artificial pathway. In this way, the complexity of natural metabolic pathways could be avoided and the metabolic flux could be properly regulated. Lin et al. (2014) reconstructed the L-tryptophan hydroxylation pathway by co-expression of a modified PAH from *Xanthomonas campestris* and a MH4 recycling pathway in *E. coli*, which brought about the accumulation of 5-HTP up to 152.9 mg/L in 48 h. Besides, precursor-directed biosynthesis of 5-HTP was achieved in *E. coli*, but the production (98 mg/L) was not much improved with regard to the previous work (Sun et al., 2015). In the present work, we engineered *E. coli* as the cell factory for 5-HTP production by introducing human L-tryptophan hydroxylation and BH4 synthesis and regeneration pathways, and single-step fermentation was achieved for 5-HTP accumulation by further integration of the L-tryptophan synthetic module. Compared with the PAH pathway, this developed TPH pathway showed higher efficiency (1.3 g/L 5-HTP in 59 h). A similar design of the TPH pathway has been reported in a previous patent, where 0.9 mM (equivalent to 198 mg/L) 5-HTP was produced from L-tryptophan (Knight et al., 2013). However, the production efficiency even with L-tryptophan supplementation is not satisfying for large-scale production and *de novo* production of 5-HTP from simple carbons was not achieved. In the present work, we demonstrated *de novo* 5-HTP production from glucose or glycerol by integrating the L-tryptophan biosynthesis module, the L-tryptophan hydroxylation module, and the BH4 biosynthesis and regeneration module. What is more, we engineered the expression levels and/or enzyme activities of each module to achieve the highest production of 5-HTP ever reported.

It was reported that human TPH exhibited a higher L-tryptophan hydroxylation activity than the W326F mutant of PAH (McKinney et al., 2001). In this study, more stable and soluble expression of this TPH enzyme was achieved through its N-terminal truncation, which increased the productivity of 5-HTP for more than two folds (Fig. 7). Therefore, the enhanced expression and stability of the truncated human TPH was one important reason for higher accumulation of 5-HTP in our constructed strain compared with those using other strategies. However, the TPH pathway has not been adopted since the complex BH4 supply system should be reconstructed to facilitate L-tryptophan hydroxylation in this recombinant *E. coli* strain. In the present work, we expressed the five genes (*mtrA*, *PTPS*, *SPR*, *PCD*, *DHPR*) for BH4 synthesis and regeneration, which would bring about heavy burden on cell growth and metabolic flux unbalance. In order to alleviate these negative effects, it was necessary to fine-tune the expression level of the BH4 synthesis and regeneration modules to maintain moderate cell growth and the full supply of BH4 for 5-HTP synthesis.

It was previously reported that tyrosine hydroxylase (TH) was employed for efficient L-tryptophan hydroxylation and L-DOPA synthesis only with cellular MH4 cofactor (Satoh et al., 2012). This suggested that our employed TPH145 enzyme might not require high amount supply of BH4 during long-term (3–4d) L-tryptophan hydroxylation, indicating that the expression of BH4 synthesis module could be regulated by the weak *lac* promoter in pACHTP-LMT (Fig. S7). Nevertheless, BH4 regeneration should be maintained at high efficiency. It is well known that pterin-4α-carbinolamine (BH3OH), the direct product of BH4 from TPH catalyzed oxidation or auto-oxidation, can be dehydrated to quinonoid-dihydrobiopterin (qBH2) with pterin-4α-carbinolamine dehydratase (PCD) (Thöny et al., 1998). Then, qBH2 can be recycled to BH4 with dihydropteridine reductase (DHPR) in an NADH-dependent reaction (Fig. 1) (Li et al., 2015). However, BH3OH could transform to 7-substituted pterin isomers via nonenzymatic rearrangement in the absence of PCD (Adler et al., 1992; Ayling et al., 2000). In addition, the unstable qBH2 could rapidly rearrange to 7,8-dihydrobiopterin (BH2) in the absence of DHPR (Kirsch et al., 2003). Both 7-substituted pterins and BH2 are competitive inhibitors versus the natural cofactor BH4 (Adler et al., 1992; McKinney et al., 2001). Thus, a high activity of BH4 regeneration pathway was required to avoid such self-arrangements and to ensure efficient supplementation of BH4 for L-tryptophan hydroxylation. Therefore, we chose M1–93, a promoter 5 times as strong as *lac* promoter, to drive the BH4 regeneration pathway expression in pACHTP-LMT (Fig. S7) (Lu et al., 2012). As expected, this regulation of expression pattern for the new strain, *E. coli* HTP101-LMT harboring pACHTP-LMT, significantly reduced the negative effect of multi-protein expression on cell growth and greatly improved the accumulation of 5-

HTP (Fig. 4D & Fig. 6).

In order to produce 5-HTP from single carbon sources (glucose or glycerol) with one-step fermentation without external L-tryptophan supplementation, we introduced the L-tryptophan biosynthetic pathway (L-tryptophan module) into the recombinant *E. coli*. However, it was observed that L-tryptophan was accumulated to much higher levels than 5-HTP during the whole fermentation process. Our results showed that L-tryptophan accumulation could be decreased significantly (from 3652.5 mg/L to 207.9 mg/L) by reducing the copy number of the L-tryptophan module plasmid, and the production of 5-HTP was increased to 532.6 mg/L. It can be concluded that we achieved the highest production through fine-tuning of three metabolic modules (L-tryptophan module, the hydroxylation module, and the BH4 module).

Our first attempt to produce 5-HTP from glucose in M9Y medium resulted in minor efficiency (Fig. S11). This was firstly regarded as the effect of insufficient protein expression, poor cell growth, and the heavily imbalanced metabolic fluxes in *E. coli* HTP4-1 strain. To test the different effects of carbon source on 5-HTP fermentation, the high producing strain *E. coli* HTP101-LMT was cultivated in mineral medium containing 5 g/L and 10 g/L glucose or glycerol in shake flasks. As shown in Fig. S17, the recombinant strain grew faster, and accordingly 5-HTP accumulation stopped at an earlier phase in glucose containing medium. Higher 5-HTP productivity was observed in glycerol than in glucose. Perhaps the fast cell growth in glucose resulted in the accumulation of some toxic metabolites, such as acetic acid (Xue et al., 2010), which might inhibit 5-HTP synthesis. The pH value of the final broth were 6.5 (5 g/L glucose), 6.0 (10 g/L glucose), 7.0 (5 g/L glycerol), 6.5 (10 g/L glycerol), respectively. Higher productivity and longer production time led to higher 5-HTP yield with glycerol as the carbon source.

It should be noted that although the L-tryptophan hydroxylation system was re-designed as three modules with different expression levels in pACHTP-LMT, the optimal promoters for each module were not determined. A more comprehensive investigation in this aspect, such as combinatorial transcriptional engineering, should further improve 5-HTP production (Du et al., 2012; Wu et al., 2017). Consistent with previous studies, the intermediate L-tryptophan was still accumulated to high levels (Fig. 6). A preliminary extraction of 5-HTP from the fermentation broth was performed through depigmentation with activated carbon, adsorption of 5-HTP and L-tryptophan with macroporous resin, and reversed phase chromatography for the separation of 5-HTP and L-tryptophan. The purity of the extracted 5-HTP was as high as 94.3% with an overall yield of 40%. While an effective separation method would help to improve 5-HTP recovery, further metabolic engineering should be carried out to balance metabolic fluxes and minimize L-tryptophan accumulation, to decrease the cost in the down-stream separation process. Moreover, during the conversion of L-tryptophan to 5-HTP by BL21/MPS/PDT1, two enzymes in the BH4 regeneration pathway, PCD and DHPR, were almost completely degraded after 48 h, while other proteins still showed relatively high abundance (Fig. 3C). This indicated that the low stability of PCD and DHPR may be the bottleneck for efficient long-term L-tryptophan hydroxylation. In fact, the conversion dropped at 15 h for BL21/FPS/PDT1 (Fig. 3A) and at 24 h for BL21/MPS/PDT1 (Fig. 3B). Improving the stability or activity of BH4 regeneration pathway represents a new potential route for higher L-tryptophan hydroxylation efficiency.

In summary, the mammalian L-tryptophan hydroxylation pathway and the cofactor BH4 biosynthesis and regeneration system were heterologously constructed in *E. coli* to achieve *de novo* bioproduction of 5-HTP. Through a combined metabolic pathway engineering strategy including protein engineering, plasmid copy number manipulation, and transcriptional fine-tuning, 5-HTP production reached 1.3 g/L in a shake flask and 5.1 g/L in a 7 L fed-batch fermenter, both of which were the highest ever reported in literature.

Acknowledgement

This work was financially supported by National Natural Science Foundation of China (No. 21606205, 21576232 & 21506185) and the Natural Science Foundation of Zhejiang Province (No. LY18B060002).

Appendix A. Supporting information

Supplementary data associated with this article can be found in the online version at http://dx.doi.org/10.1016/j.ymben.2018.06.007.

References

Adler, C., Ghisla, S., Rebrin, I., Haavik, J., Heizmann, C.W., Blau, N., Kuster, T., Curtius, H.-C., 1992. 7-Substituted pterins in humans with suspected pterin-4a-carbinolamine dehydratase deficiency: mechanism of formation via non-enzymatic transformation from 6-substituted pterins. Eur. J. Biochem. 208 (1), 139–144.

Ayling, J.E., Bailey, S.W., Boerth, S.R., Giugliani, R., Braegger, C.P., Thöny, B., Blau, N., 2000. Hyperphenylalaninemia and 7-pterin excretion associated with mutations in 4a-hydroxy-tetrahydrobiopterin dehydratase/DCoH: analysis of enzyme activity in intestinal biopsies. Mol. Genet. Metab. 70, 179–188.

Birdsall, T.C., 1998. 5-Hydroxytryptophan: a clinically-effective serotonin precursor. Altern. Med. Rev. 3, 271–280.

Carkaci-Salli, N., Flanagan, J.M., Martz, M.K., Salli, U., Walther, D.J., Bader, M., Vrana, K.E., 2006. Functional domains of human tryptophan hydroxylase 2 (hTPH2). J. Biol. Chem. 281, 28105–28112.

Chen, D., Zhang, Q., Zhang, Q., Cen, P., Xu, Z., Liu, W., 2012. Improvement of FK506 production in *Streptomyces tsukubaensis* by genetic enhancement of the supply of unusual polyketide extender units via utilization of two distinct site-specific recombination systems. Appl. Environ. Microbiol. 78 (15), 5093–5103.

Datsenko, K.A., Wanner, B.L., 2000. One-step inactivation of chromosomal genes in *Escherichia coli* K-12 using PCR products. Proc. Natl. Acad. Sci. USA 97, 6640–6645.

Du, J., Yuan, Y., Si, T., Lian, J., Zhao, H., 2012. Customized optimization of metabolic pathways by combinatorial transcriptional engineering. Nucleic Acids Res. 40, e142.

Gibson, D.G., Young, L., Chuang, R.Y., Venter, J.C., Hutchison, C.A., Smith, H.O., 2009. Enzymatic assembly of DNA molecules up to several hundred kilobases. Nat. Methods 6, 343–345.

Hara, R., Kino, K., 2013. Enhanced synthesis of 5-hydroxy-L-tryptophan through tetrahydropterin regeneration. AMB Express 3, 70.

Huang, L., Pu, Y., Yang, X., Zhu, X., Cai, J., Xu, Z., 2015. Engineering of global regulator cAMP receptor protein (CRP) in *Escherichia coli* for improved lycopene production. J. Biotechnol. 199, 55–61.

Jacobsen, J.P.R., Frøstrup, B., 2015. (inventors), Duke University, (assignee), Pharmaceutical Compositions of 5-Hydroxytryptophan and Serotonin-Enhancing Compound, US008969400B2 (issued 2015, Mar 3).

Knight, E.M., Zhu, J., Förster, J., Luo, H., 2013. Microorganisms for the production of 5-hydroxytryptophan, WO2013127914.

Kirsch, M., Korth, H.G., Stenert, V., Sustmann, R., Groot, H., 2003. The autoxidation of tetrahydrobiopterin revisited: proof of superoxide formation from reaction of tetrahydrobiopterin with molecular oxygen. J. Biol. Chem. 278, 24481–24490.

Li, W., Gong, M., Shu, R., Li, X., Gao, J., Meng, Y., 2015. Molecular and enzymatic characterization of two enzymes BmPCD and BmDHPR involving in the regeneration pathway of tetrahydrobiopterin from the silkworm *Bombyx mori*. Comp. Biochem. Physiol. B: Biochem. Mol. Biol. 186, 20–27.

Lin, Y., Sun, X., Yuan, Q., Yan, Y., 2014. Engineering bacterial phenylalanine 4-hydroxylase for microbial synthesis of human neurotransmitter precursor 5-hydroxytryptophan. ACS Synth. Biol. 3, 497–505.

Lu, J., Tang, J., Liu, Y., Zhu, X., Zhang, T., Zhang, X., 2012. Combinatorial modulation of galP and glk gene expression for improved alternative glucose utilization. Appl. Microbiol. Biotechnol. 93, 2455–2462.

Luo, W., Huang, J., Zhu, X., Huang, L., Cai, J., Xu, Z., 2013. Enhanced production of L-tryptophan with glucose feeding and surfactant addition and related metabolic flux redistribution in the recombinant *Escherichia coli*. Food Sci. Biotechnol. 22, 207–214.

Lynch, S.A., Gill, R.T., 2012. Synthetic biology: new strategies for directing design. Metab. Eng. 14, 205–211.

McKinney, J., Teigen, K., Frøystein, N.Å., Salaün, C., Knappskog, P.M., Haavik, J., Martínez, A., 2001. Conformation of the substrate and pterin cofactor bound to human tryptophan hydroxylase. Important role of Phe313 in substrate specificity. Biochemistry 40, 15591–15601.

Murphy, K.L., Zhang, X., Gainetdinov, R.R., Beaulieu, J.M., Marc, G.C., 2008. A regulatory domain in the N terminus of tryptophan hydroxylase 2 controls enzyme expression. J. Biol. Chem. 283, 13216–13224.

Nielsen, M.S., Petersen, C.R., Munch, A., Vendelboe, T.V., Boesen, J., Harris, P., Christensen, H.E.M., 2008. A simple two step procedure for purification of the catalytic domain of chicken tryptophan hydroxylase 1 in a form suitable for crystallization. Protein Express. Purif. 57, 116–126.

Salis, H.M., Mirsky, E.A., Voigt, C.A., 2009. Automated design of synthetic ribosome binding sites to precisely control protein expression. Nat. Biotechnol. 27, 946–950.

Satoh, Y., Tajima, K., Munekata, M., Keasling, J.D., Lee, T.S., 2012. Engineering of L-tyrosine oxidation in Escherichia coli and microbial production of hydroxytyrosol. Metab. Eng. 14, 603–610.

Sun, X., Lin, Y., Yuan, Q., Yan, Y., 2015. Precursor-directed biosynthesis of 5-

hydroxytryptophan using metabolically engineered *E. coli*. ACS Synth. Biol. 4 (5), 554–558.
Tang, Y., Yang, X., Hang, B., Li, J., Huang, L., Huang, F., Xu, Z., 2016. Efficient production of hydroxylated human-like collagen via the co-expression of three key genes in *Escherichia coli* origami (DE3). Appl. Biochem. Biotechnol. 178 (7), 1458–1470.
Thöny, B., Neuheiser, F., Kierat, L., Blaskovics, M., Arn, P.H., Ferreira, P., Rebrin, I., Ayling, J., Blau, N., 1998. Hyperphenylalaninemia with high levels of 7-biopterin is associated with mutations in the PCBD gene encoding the bifunctional protein pterin-4acarbinolamine dehydratase and transcriptional coactivator (DCoH). Am. J. Hum. Genet. 62, 1302–1311.
Turner, E.H., Loftis, J.M., Blackwell, A.D., 2006. Serotonin a la carte: supplementation with the serotonin precursor 5-hydroxytryptophan. Pharmacol. Ther. 109 (3), 325–338.
Walther, D.J., Peter, J.U., Bashammakh, S., Hörtnag, H., Voits, M., Fink, H., Bader, M., 2003. Synthesis of Serotonin by a Second Tryptophan Hydroxylase Isoform. Science 299 (5603), 76.
Winge, I., McKinney, J.A., Knappskog, P.M., Haavik, J., 2007. Characterization of wild-type and mutant forms of human tryptophan hydroxylase 2. J. Neurochem. 100, 1648–1657.
Wu, J., Zhang, X., Zhu, Y., Tan, Q., He, J., Dong, M., 2017. Rational modular design of metabolic network for efficient production of plant polyphenol pinosylvin. Sci. Rep. 7, 1459.
Xue, W., Fan, D., Shang, L., Zhu, C., Ma, X., Zhu, X., Yu, Y., 2010. Effects of acetic acid and its assimilation in fed-batch cultures of recombinant *Escherichia coli* containing human-like collagen cDNA. J. Biosci. Bioeng. 109 (3), 257–261.
Yamamoto, K., Kataoka, E., Miyamoto, N., Furukawa, K., Ohsuye, K., Yabuta, M., 2003. Genetic engineering of *Escherichia coli* for production of tetrahydrobiopterin. Metab. Eng. 5, 246–254.
Yang, Y., Huang, L., Wang, J., Wang, X., Xu, Z., 2014. Efficient expression, purification, and characterization of a novel FAD-dependent glucose dehydrogenase from *Aspergillus terreus* in *Pichia pastoris*. J. Microbiol. Biotechnol. 24 (11), 1516–1524.
Zelcbuch, L., Antonovsky, N., Bar-Even, A., Levin-Karp, A., Barenholz, U., Dayagi, M., Liebermeister, W., Flamholz, A., Noor, E., Amram, S., Brandis, A., Bareia, T., Yofe, I., Jubran, H., Milo, R., 2013. Spanning high-dimensional expression space using ribo-some-binding site combinatorics. Nucleic Acids Res. 41, e98.
Zhao, W., Hang, B., Zhu, X., Wang, R., Shen, M., Huang, L., Xu, Z., 2016. Improving the productivity of S-adenosyl-L-methionine by metabolic engineering in an industrial *Saccharomyces cerevisiae* strain. J. Biotechnol. 236, 64–70.
