# Supplementary material for: 5-Hydroxytryptophan (5-HTP): Natural Occurrence, Analysis, Biosynthesis, Biotechnology, Physiology and Toxicology
Source: Int J Mol Sci. 2020 Dec 26;22(1):181. doi: 10.3390/ijms22010181 (PMC7796270; doi:10.3390/ijms22010181)
Supplement: Supplementary file 1 [file ijms-22-00181-s001.zip › 5-HTP.Data/PDF/0516609089/Kang-2009-Biosynthesis-and-biotechnological-p.pdf]

Appl Microbiol Biotechnol (2009) 83:27–34
DOI 10.1007/s00253-009-1956-1

MINI-REVIEW

Biosynthesis and biotechnological production of serotonin derivatives

Kiyoon Kang · Sangkyu Park · Young Soon Kim · Sungbeom Lee · Kyoungwhan Back

Received: 19 February 2009 / Revised: 8 March 2009 / Accepted: 9 March 2009 / Published online: 24 March 2009
© Springer-Verlag 2009

Abstract Serotonin derivatives belong to a class of phenylpropanoid amides found at low levels in a wide range of plant species. Representative serotonin derivatives include feruloylserotonin (FS) and 4-coumaroylserotonin (CS). Since the first identification of serotonin derivatives in safflower seeds, their occurrence, biological significance, and pharmacological properties have been reported. Recently, serotonin *N*-hydroxycinnamoyl transferase (SHT), which is responsible for the synthesis of serotonin derivatives, was cloned from pepper (*Capsicum annuum*) and characterized in terms of its enzyme kinetics. Using the SHT gene, many attempts have been made to either increase the level of serotonin derivatives in transgenic plants or produce serotonin derivatives *de novo* in microbes by dual expression of key genes such as SHT and 4-coumarate-CoA ligase (4CL). Due to the strong antioxidant activity and other therapeutic properties of serotonin derivatives, these compounds may have high potential in treatment and prophylaxis, as cosmetic ingredients, and as major components of functional foods or feeds that have health-improving effects. This review examines the biosynthesis of serotonin derivatives, corresponding enzymes, heterologous production in plants or microbes, and their applications.

Keywords Antioxidant · Caffeoylserotonin · Feruloylserotonin · 4-Coumaroylserotonin · Serotonin derivatives

K. Kang · Y. S. Kim · K. Back (✉)
Department of Biotechnology, Agricultural Plant Stress Research Center, Chonnam National University,
Gwangju 500-757, Republic of Korea
e-mail: kback@chonnam.ac.kr

S. Park
Interdisciplinary Program for Bioenergy and Biomaterials of Graduate School, Chonnam National University,
Gwangju 500-757, Korea

S. Lee
Biological Sciences, Virginia Tech,
Blacksburg, VA 24061, USA

Introduction

Serotonin derivatives are an important class of phenylpropanoid amides (PAs) that play diverse roles in plant–plant, plant–pathogen, and plant–environment interactions (Martin-Tanguy 1985; Facchini et al. 2002). PAs are synthesized by the condensation of two substrates (e.g., cinnamoyl-CoA thioesters and amines), which are linked by an amide bond. The cinnamoyl-CoA thioesters include cinnamoyl-CoA, *p*-coumaroyl-CoA, caffeoyl-CoA, feruloyl-CoA, and sinapoyl-CoA. The amines consist of both aliphatic and aromatic substrates. The aliphatic amine substrates are mono-, di-, and polyamines, such as methylamine, putrescine, and spermine, which are found in at least 34 compounds in plants, whereas the aromatic amines include tyramine, tryptamine, octopamine, tryptamine, serotonin, dopamine, histamine, adrenaline, and noradrenaline (Wink 1997). Thus, the combination of these two substrate types can lead to the production of at least 215 different kinds of PAs. Among the diverse array of PAs, serotonin derivatives represent the condensed products of various cinnamoyl-CoA thioesters and serotonin, and comprise feruloylserotonin (FS), 4-coumaroylserotonin (CS), caffeoylserotonin (CaS), sinapoylserotonin (SS), and cinnamoylserotonin (CiS) (Fig. 1). Furthermore, these serotonin derivatives can be coupled with other phenolic compounds to form macrolactam-type compounds (Jenett-Siems et al.

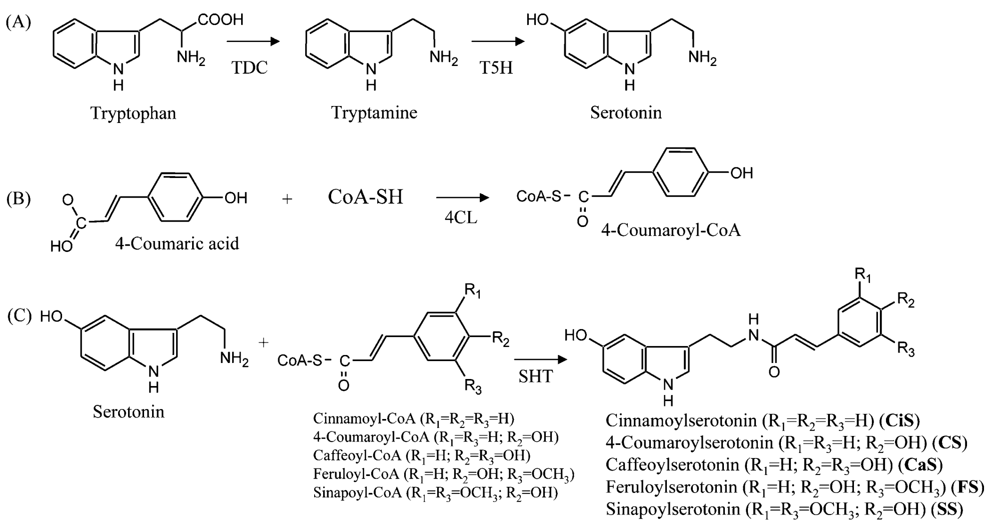

Fig. 1 Biosynthesis of serotonin (**a**), 4-coumaroyl-CoA (**b**), and serotonin derivatives (**c**) by the corresponding enzymes. *TDC* tryptophan decarboxylase; *T5H* tryptamine 5-hydroxylase; *4CL* 4-coumarate-CoA ligase; *SHT* serotonin *N*-hydroxycinnamoyl transferase

2003). Like most plant-specific secondary metabolites, serotonin derivatives play not only a defensive role against pathogen attack (Tanaka et al. 2003) but also exhibit various health-promoting properties such as anti-oxidative and chemotherapeutic properties (Zhang et al. 1996; Park and Schoene 2002). Since their initial identification in safflower seeds (Sakamura et al. 1978), serotonin derivatives have been found in at least 16 plant species representing eight different families. The levels of serotonin derivatives in plants vary greatly among species and tissues, ranging from 0.1 to 740 μg g^{-1}, and are preferentially found in the seeds of Asteraceae. Among the various serotonin derivatives in plants, FS and CS are commonly detected, whereas CaS, SS, and CiS have not been reported. Recently, serotonin *N*-hydroxycinnamoyl transferase (SHT), the enzyme essential for the biosynthesis of serotonin derivatives, was cloned from pepper (*Capsicum annuum*), and its characteristics were reported in detail (Jang et al. 2004; Kang et al. 2006). Since the first cloning of the SHT gene, many attempts have been made to express SHT ectopically in either plants or microbes in order to engineer the production of serotonin derivatives. This review describes the biosynthesis of serotonin derivatives in plants, the enzymes involved and their regulation, metabolic engineering of the pathway in plants, and approaches to overproduce serotonin derivatives in microbes through fermentation processes.

Biosynthesis and regulation of serotonin derivatives in plants

Theoretically, five basic serotonin derivatives can be generated due to the presence of five cinnamoyl-CoA thioesters in plants: FS, CS, CaS, SS, and CiS (Fig. 1). FS and CS were first identified in safflower seeds (*Carthamus tinctorius* L.) as bioactive compounds exhibiting cathartic activity (Sakamura et al. 1978). The richest source of serotonin derivatives in seeds is in *Leuzea carthamoides* (family Asteraceae), which contains up to 600 μg g^{-1} FS and 740 μg g^{-1} CS (Pavlík et al. 2002), whereas many other plants, including rice and pepper, produce low levels of FS and CS in various tissues (Table 1). Pepper flowers contain a relatively high level of CS (7.3 μg g^{-1}) compared to other tissues, although the exact reason for this remains to be examined. So far, CaS, SS, and CiS have not been identified in plants, although caffeoyltyramine and a CaS analog are found in many plant species, including *Lycium chinense* (Lee et al. 2004). In general, PAs such as caffeoylspermidine and feruloyltyramine have been implicated in a series of plant growth and developmental processes, including flowering, tuber formation, storage compounds, cell wall fortification, and alleviation of tyramine toxicity (Facchini et al. 2002). In contrast, studies on the physiological roles of serotonin derivatives have received little attention from plant biologists due to either the low abundance in agricultural plants or their relatively short history since their initial identification. According to Tanaka et al. (2003), FS and CS are induced in twigs of diseased bamboo and show antifungal activity, suggesting that serotonin derivatives play a role as phytoalexins. Similarly, rice leaves also show increased synthesis of FS and CS upon pathogen infection (Ishihara et al. 2008). In addition, FS and CS are highly induced upon senescence in rice plants, indicating that serotonin derivatives may be associated with an aging-inhibitory mechanism because of their strong antioxidant activity (Kang et al., in preparation). Besides importance for the plant itself, plant-specific serotonin derivatives are of interest because they have various health-promoting and disease preventative effects. For example, the anti-oxidative activities of FS and CS are stronger than α-tocopherol, a natural antioxidant, and comparable to that of butylated hydroxyanisole (BHA), a synthetic antioxidant (Zhang et al., 1996). In addition, CS and FS have several biological activities that may be useful

Table 1 The occurrence of serotonin derivatives in plants

Family and species	Serotonin derivatives (μg g^{-1} plant mass)		Reference
	FS	CS	
Araceae			
Amorphophallus konjac	Seeds nd	Seeds 9.2	Niwa et al. 2000
Asteraceae			
Carthamus tinctorius	Oil cakes 13	Oil cakes 41	Zhang et al. 1996
Centaurea cyanus	Seeds 318		Sarker et al. 2001
Centaurea montana	Seeds 225		Shoeb et al. 2006
Centaurea nigra	Seeds 545		Kumarasamy et al. 2003
Leuzea carthamoides	Seeds 600	Seeds 740	Pavlík et al. 2002
Chrysantimum			
Cichorium intybus	Leaves 0.1	Leaves nd	Ly et al. 2008
Lactuca sativa	Leaves 0.1	Leaves nd	Ly et al. 2008
Convolvulaceae			
Ipomoea obscura		Seeds 300	Jenett-Siems et al. 2003
Cruciferae			
Brassica campestris	Leaves 0.1	Leaves nd	Ly et al. 2008
Liliaceae			
Allium fistulosum	Leaves nd	Leaves 0.7	Ly et al. 2008
Poaceae			
Echinochloa utilis		Seeds 16	Watanabe 1999
Oryza sativa	Roots 0.1	Roots 0.3	Jang et al. 2004
Phyllostachys bambusoides	Twigs 2.8	Twigs nd	Tanaka et al. 2003
Solanaceae			
Lycopersicon esculentum	Leaves 0.1	Leaves 0.7	Kang et al. 2009
Capsicum annuum	Leaves nd<br>Stems 0.2<br>Fruits nd<br>Flowers nd	Leaves 0.7<br>Stems 0.7<br>Fruits 0.8<br>Flowers 7.3	Kang and Back 2006

FS feruloylserotonin, *CS* 4-coumaroylserotonin, *nd* not detected

in prophylaxis, such as in atherosclerosis (Koyama et al. 2006), inflammation (Takii et al. 2003), and cardiac ischemia–reperfusion injury (Hotta et al. 2002). CS and FS are also reported to have anti-tumor (Nagatsu et al. 2000; Shoeb et al. 2006), anti-bacterial (Kumarasamy et al. 2003), anti-stress (Yamamotová et al. 2007), and anti-melanogenesis (Roh et al. 2004) effects, and appear to promote the growth of fibroblasts (Takii et al. 1999). In addition to CS and FS, CaS may be involved in reducing depression and anxiety (Park 2008).

SHT, a key enzyme for serotonin biosynthesis, exhibits a broad substrate affinity for both aromatic amines (e.g., serotonin, tyramine, and octopamine) and various cinnamoyl-CoA thioesters, which results in the synthesis of a series of PAs. In contrast, tyramine *N*-hydroxycinnamoyl transferase (THT) lacks serotonin affinity (Kang et al. 2006). SHT shows high substrate affinities for cinnamoyl-CoA thioesters, with K_m values ranging from 3.5 μM (feruloyl-CoA) to 32 μM (cinnamoyl-CoA). In contrast, SHT shows a relatively low substrate affinity for serotonin (K_m=73 μM) compared to cinnamoyl-CoA thioesters. This difference in substrate affinity may be one of factors that make serotonin a limiting role in the biosynthesis of serotonin derivatives in plants. Accordingly, the overexpression of SHT in transgenic rice is insufficient to enhance the production of serotonin derivatives in young seedlings due to the low level of serotonin (Jang et al. 2004). Likewise, serotonin overexpression only is not directly associated with the enhanced synthesis of serotonin derivatives in transgenic rice plants due to the low level of SHT expression (Kang et al. 2007a); therefore, combined serotonin and SHT overexpression are required to increase the production of serotonin derivatives. In plants, serotonin is synthesized in two enzymatic steps, in which tryptophan is first converted to tryptamine by tryptophan decarboxylase (TDC), and this is followed by tryptamine 5-hydroxylase (T5H) to form serotonin (Schröder et al. 1999). However, in St. John's wort (*Hypericum perforatum*), serotonin synthesis is reported to occur via 5-hydroxytryptophan, as in mammals (Murch et al. 2000). T5H enzyme activity is very high in all tissues of rice plants, with a low K_m of 20 μM toward

tryptamine as a substrate, whereas TDC has marginal enzyme activity in all tissues tested (Kang et al. 2007a, b). Since TDC has a high K_m of 690 μM toward tryptophan as a substrate, TDC overexpression is insufficient to increase serotonin levels because tryptophan levels are very low (<15 μM) compared to other amino acids. In addition, the tryptophan pathway in plants is tightly feedback regulated by micromolar concentrations of tryptophan (Radwanski and Last 1995). Hence, the biosynthesis of serotonin derivatives in plants is strictly regulated not only at the substrate concentration level but also at the level of key enzymes such as TDC and SHT. To overcome these bottlenecks in the production of serotonin derivatives in plants, it is necessary to increase the production of tryptophan and serotonin as well as increase the expression of the terminal enzyme SHT. Recently, it was demonstrated that tryptophan levels can be greatly increased by the expression of a mutant form of anthranilate synthase α1 (ASα1 D323N), which is insensitive to feedback inhibition by tryptophan (Tozawa et al. 2001). In addition, TDC from *Cathranthus roseus*, which shows a low K_m of 72 μM toward tryptophan, may be highly beneficial to increase serotonin levels in plants (Noé et al. 1984). In addition to K_m values, the catalytic efficiency of an enzyme which is a function of K_{cat}/K_m has to be considered for all relevant enzymes in the biosynthesis pathway of serotonin derivatives. Taken together, the simultaneous expression of ASα1 (D323N), TDC from *C. roseus*, and SHT could significantly increase the levels of serotonin derivatives in plants (Fig. 2). In contrast to the regulatory roles of AS, TDC, and SHT, the enzyme 4CL, which catalyzes the conversion of cinnamic acids into cinnamoyl-CoA thioesters, does not seem to have a rate-limiting role in the biosynthesis of serotonin derivatives. Instead, 4CL exists as a small gene family in plants with a basal level of constitutive expression and has a high affinity toward its substrates (Ehlting et al. 1999). Thus, cellular pools of cinnamoyl-CoA thioesters may be sufficient for the synthesis of serotonin derivatives without a bottleneck role at the level of substrate.

Heterologous production of serotonin derivatives in plants and microbes

Since the cloning of SHT gene (Jang et al. 2004), a number of experiments have attempted to express SHT heterologously to increase the production of serotonin derivatives in various organisms (Table 2). In parallel, the production of tyramine derivatives such as feruloyltyramine and 4-coumaroyltyramine, which are synthesized by tyramine *N*-hydroxycinnamoyl transferase (THT), has been studied in transgenic tobacco plants (Guillet and De Luca 2005; Hagel and Facchini 2005). SHT was first heterologously introduced in the rice genome to determine whether transgenic rice plants produce high levels of serotonin derivatives. As expected, the transgenic rice leaves expressing SHT under the control of the maize ubiquitin promoter showed abundant FS (200 μg g^{-1} fresh weight [fw]) and CS (224 μg g^{-1} fw) production, with a 170-fold increase compared to the wild type (Jang et al. 2004). However, this enhanced production of serotonin derivatives in transgenic rice leaves required treatment with amines and cinnamic acids. Like leaves, the same transgenic rice also produced 20-fold more FS+CS in seeds than the wild type (Kang et al. 2005). In contrast to the constitutive expression of SHT, the ectopic expression of SHT under the control of the endosperm-specific prolamine promoter produced serotonin derivatives in rice seeds at

Fig. 2 Proposed regulatory steps for the biosynthesis of serotonin derivatives. *ASα1 (D323N)* mutant anthranilate synthase α1, in which Asp-323 has been replaced by Gln; *OsTDC Oryza sativa* tryptophan decarboxylase; *CrTDC Cathranthus roseus* tryptophan decarboxylase; *T5H* tryptamine 5-hydroxylase; *SHT* serotonin *N*-hydroxycinnamoyl transferase

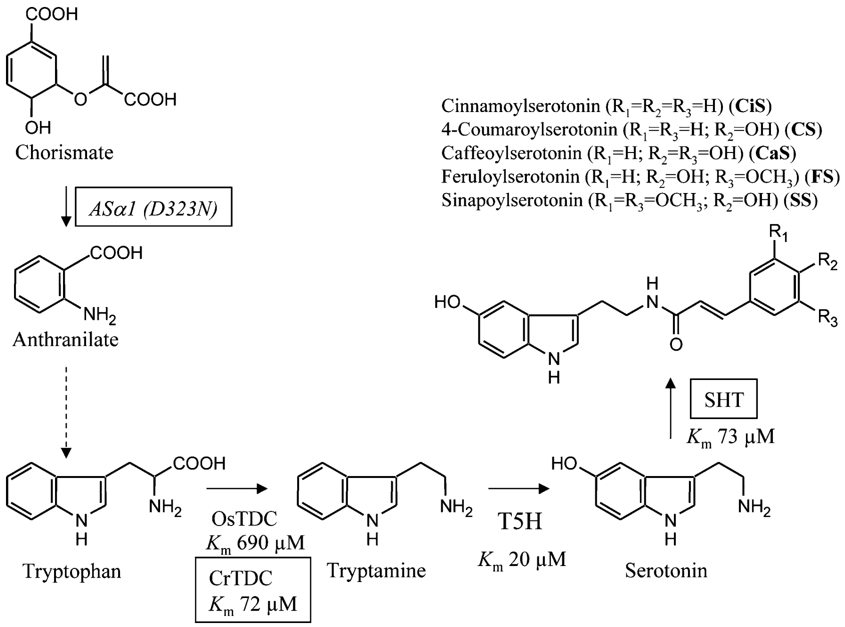

Table 2 Heterologous production of serotonin derivatives in various organisms

Organisms	Introduced gene(s)	Serotonin derivatives	Reference
Rice	SHT	FS 200 μg g^{-1} fw (leaves)<br>CS 224 μg g^{-1} fw (leaves)	Jang et al. 2004
		FS 0.7 μg g^{-1} (seeds)<br>CS 0.4 μg g^{-1} (seeds)	Kang et al. 2005
		FS+CS 1.7 μg g^{-1} (seeds)	Lee et al. 2008
Tomato	SHT	FS 0.6 μg g^{-1} fw (leaves)<br>CS 0.1 μg g^{-1} fw (leaves)	Kang et al. 2009
Yeast	4CL+SHT	FS 4.4 mg l^{-1} (medium)<br>CS 1.4 mg l^{-1} (medium)	Park et al. 2008b
		CaS 2.7 mg l^{-1} (medium)	
E. coli	4CL+SHT	FS 96 mg l^{-1} (medium)<br>CS 215 mg l^{-1} (medium)	Kang and Back 2009
		CaS 11 mg l^{-1} (medium)	
		CiS 39 mg l^{-1} (medium)	
		CS 22 mg l^{-1} (cells)	

SHT serotonin *N*-hydroxycinnamoyltransferase, *4CL* 4-coumarate-CoA ligase, *FS* feruloylserotonin, *CS* 4-coumaroylserotonin, *CaS* caffeoylserotonin, *CiS* cinnamoylserotonin

levels similar to those of the wild type (Lee et al. 2008). In transgenic tomatoes expressing SHT under the control of the constitutive CaMV 35 S promoter, leaves produced 3-fold more FS+CS (675 ng g^{-1} fw) than the wild-type (200 ng g^{-1} fw) upon wounding, whereas the tomato fruits contained similar levels of serotonin derivatives in both transgenic and wild-type plants (Kang et al. 2009). Because the tomato fruits did not show increased serotonin derivative production, despite the presence of abundant serotonin in fruits, metabolic controls other than serotonin level are likely involved in the regulation of serotonin derivative biosynthesis in plants (Hagel and Facchini 2005).

In contrast to the somewhat problematic heterologous production of serotonin derivatives in plants, microbial hosts provide good alternatives for mass production of plant-specific secondary metabolites with important biological functions (Mijts and Schmidt-Dannert 2003). In microbes such as *Escherichia coli* or *Saccharomyces cerevisiae*, the expression of SHT alone is insufficient for the production of serotonin derivatives, because *E. coli* is unable to synthesize cinnamoyl-CoA thioester substrates such as feruloyl-CoA or caffeoyl-CoA, nor are these cinnamoyl-CoA thioesters commercially available. Thus, the expression of 4-coumarate-CoA ligase (4CL), which is responsible for the synthesis of cinnamoyl-CoA thioesters from cinnamic acids, is required together with SHT overexpression for the production of serotonin derivatives in microbes. The first trial of serotonin derivative production in microbes was carried out in yeast (*S. cerevisiae*) via the coordinate expression of SHT and 4CL, which were translationally linked using a foot-and-mouth disease virus (FMDV) 2A sequence in a single open reading frame (Park et al. 2008b). The FMDV 2A sequence, which consists of 16–20 amino acids, is cleaved co-translationally between Gly and Pro at its C-terminus, not by a proteolytic mechanism, but rather by a translational ribosome skipping mechanism (Ryan et al. 1991). The resulting recombinant yeast harboring the SHT–2A–4CL plasmid construct led to the production of serotonin derivatives (Table 2). FS was produced in the extracellular medium fraction at up to 4.4 mg l^{-1} with 2 mM serotonin and ferulic acid treatment. In the presence of 2 mM 4-coumaric acid and serotonin, 1.4 mg l^{-1} CS was produced, whereas the addition of caffeic acid and serotonin produced 2.7 mg l^{-1} CaS. In contrast, neither SS nor CiS was produced in the presence of their corresponding precursors. Although serotonin derivatives were produced successfully in yeast, their levels were relatively low compared to other plant-specific secondary metabolites, such as the indole alkaloid, strictosidine (Geerlings et al. 2001). In another approach, the dual expression of SHT and 4CL in *E. coli* was attempted to determine whether serotonin derivatives were produced at higher levels than in yeast. Unlike the coordinate expression of SHT and 4CL in yeast, SHT and 4CL were co-expressed under the control of separate promoters (Kang and Back 2009; this recombinant strain was deposited by the accession number of KCTC 11272BP in Korean Collection for Type Cultures). Surprisingly, the recombinant *E. coli* showed high extracellular levels of serotonin derivatives within a few hours after the addition of serotonin and various cinnamic acids. The serotonin derivatives produced included CS (215 mg l^{-1}), FS (96 mg l^{-1}), CiS (39 mg l^{-1}), and CaS (11 mg l^{-1}). Considering that *E. coli* cells only contain 22 mg l^{-1} CS, which is a tenth of the extracellular CS level, the majority of serotonin derivatives synthesized in *E. coli* appear to have been secreted into the extracellular medium. This may simplify the purification procedure of serotonin derivatives by using a simple ethylacetate extraction method from an *E. coli* culture system (Kang and Back 2009).

Applications of serotonin derivatives

Since serotonin derivatives, like many other plant-specific secondary metabolites (e.g., lycopene and resveratrol), have a diverse array of biological activities, including anti-oxidative and chemotherapeutic effects, they possess great commercial value in terms of improving the nutritional value of foods and cosmetics. For example, CS and FS were shown to be effective skin depigmentation compounds that inhibited melanin synthesis in B16 melanoma cells without cytotoxicity (Roh et al. 2004). The tyrosinase inhibitory activities of CS and FS were 3- and 10-fold higher, respectively, than that of the known melanogenesis inhibitor arbutin. Based on both their high levels of melanogenesis inhibitory activity and low cytotoxicity, CS and FS are promising novel natural whitening agents. Besides using serotonin derivatives as skin-whitening agents, serotonin derivatives have been implicated as having therapeutic effects in the treatment of atherosclerosis (Koyama et al. 2009), inflammation (Yuji et al. 2007), and osteoporosis (Choi et al. 2002) in several patents. In addition, serotonin derivatives possess great potential as natural antioxidants to retard the oxidation of lipid-containing foodstuffs due to their strong antioxidant activity, as mentioned above. Although no commercial utilization of serotonin derivatives in the cosmetic, pharmaceutical, and food industries has been reported, they will likely be available commercially in the future.

Conclusions and future prospects

Until now, serotonin derivatives have been mainly produced via chemical synthesis in order to study their therapeutic effects, using serotonin and cinnamic acids as starting material. For example, CS was synthesized by converting 4-coumaric acid to the symmetrical anhydride in the presence of 1,3-diisopropylcarbodiimide, followed by the addition of serotonin to produce 4-coumaroylserotonin, with an efficiency yield of 55%. However, these chemical synthesis steps require large quantities of solvents and several days to complete (Ishihara et al. 2000; Park and Schoene 2002). In comparison, the recombinant *E. coli* system requires only a few hours to synthesize serotonin derivatives, without the need for chemical solvents or chemical synthesis techniques. Although *E. coli* culture provides a good system to produce serotonin derivatives, further fermentation processes should be developed to optimize serotonin derivative production. Among the unsolved tasks is product inhibition of SHT enzyme during culture and reduced productivity of other serotonin derivatives, such as CiS, CaS, and SS. During *E. coli* fermentation, CS production inhibits SHT activity by 40%, which may explain the observed CS production ceiling of no more than 215 mg l^{-1}. Minimizing the cellular levels of serotonin derivatives (i.e., retrieval or removal of products) is required to prevent product inhibition. In addition, the yield of FS, CiS, and CaS was relatively low compared to CS production, which may reflect the reduced permeability of cells toward the relevant substrates (i.e., cinnamic acid, caffeic acid, and sinapic acid) compared to 4-coumaric acid. Further studies on cell permeability and, if required, methods to increase the permeability of *E. coli* cells to various cinnamic acids during fermentation are needed. Various cinnamic acids are cheap and commercially available (e.g., 4-coumaric acid costs only 242 yen/g; TCI product no. C0393), whereas serotonin is rather expensive (1,296 yen/g; TCI product no. S0370). This emphasizes the need for an *E. coli* fermentation system that produces serotonin via the simultaneous expression of two serotonin biosynthesis genes, TDC and T5H (Park et al. 2008a), although T5H has not yet been cloned (Kang et al. 2007b). Therefore, either the co-expression of four genes (i.e., SHT, 4CL, TDC, and T5H) or the co-culture of two compatible strains (i.e., one strain expressing SHT+4CL and another expressing TDC+T5H) may be the most economical method to produce serotonin derivatives via *E. coli* fermentation.

Acknowledgments Thanks are expressed to Prof. A. Ishihara of the Graduate School of Kyoto University (Kyoto, Japan) for technical support. This work was supported by the Technology Development Program for Agriculture and Forestry, Ministry of Agriculture, Forestry, and Fisheries, and by the SRC program of MOST/KOSEF, through the Agricultural Plant Stress Research Center (R11-2001-092-05001-0).

References

Choi SW, Park RW, Lee WJ (2002) Novel use of polyphenol compounds isolated from safflower (*Carthamus tincorious* L.) seeds. Korea patent 10-0354791-0000

Ehlting J, Büttner D, Wang Q, Douglas CJ, Somssich IE, Kombrink E (1999) Three 4-coumarate:coenzyme A ligases in *Arabidopsis thaliana* represent two evolutionarily divergent classes in angiosperms. Plant J 19:9–20

Facchini PJ, Hagel J, Zulak KG (2002) Hydroxycinnamic acid amide metabolism: physiology and biochemistry. Can J Bot 80:577–589

Geerlings A, Redondo FJ, Contin A, Memelink J, van der Heijden R, Verpoorte R (2001) Biotransformation of tryptamine and secologanin into plant terpenoid indole alkaloids by transgenic yeast. Appl Microbiol Biotechnol 56:420–424

Guillet G, De Luca V (2005) Wound-inducible biosynthesis of phytoalexin hydroxycinnamic acid amides of tyramine in tryptophan and tyrosine decarboxylase transgenic tobacco lines. Plant Physiol 137:692–699

Hagel JM, Facchini PJ (2005) Elevated tyrosine decarboxylase and tyramine hydroxycinnamoyltransferase levels increase wound-induced tyramine-derived hydroxycinnamic acid amide accumulation in transgenic tobacco leaves. Planta 221:904–914

Hotta Y, Nagatsu A, Liu W, Muto T, Narumiya C, Lu X, Yajima M, Ishikawa N, Miyazeki K, Kawai N, Mizukami H, Sakakibara J (2002) Protective effects of antioxidative serotonin derivatives isolated from safflower against postischemic myocardial dysfunction. Mol Cell Biochem 238:151–162

Ishihara A, Kawata N, Matsukawa T, Iwamura H (2000) Induction of *N*-hydroxycinnamoyltyramine synthesis and tyramine *N*-hydroxycinnamoyltransferase (THT) activity by wounding in maize leaves. Biosci Biotechnol Biochem 64:1025–1031

Ishihara A, Hashimoto Y, Tanaka C, Dubouzet JG, Nakao T, Matsuda F, Nishioka T, Miyagawa H, Wakasa K (2008) The tryptophan pathway is involved in the defense responses of rice against pathogenic infection via serotonin production. Plant J 54:481–495

Jang SM, Ishihara A, Back K (2004) Production of coumaroylserotonin and feruloylserotonin in transgenic rice expressing pepper hydroxycinnamoyl-coenzyme A:serotonin N-(hydroxycinnamoyl) transferase. Plant Physiol 135:346–356

Jenett-Siems K, Weigl R, Kaloga M, Schulz J, Eich E (2003) Ipobscurines C and D: macrolactam-type indole alkaloids from the seeds of *Ipomoea obscura*. Phytochemistry 62:1257–1263

Kang S, Back K (2006) Enriched production of *N*-hydroxycinnamic acid amides and biogenic amines in pepper (*Capsicum annuum*) flowers. Sci Hortic 108:337–341

Kang K, Back K (2009) Production of phenylpropanoid amides in recombinant *Escherichia coli*. Metab Eng 11:64–68

Kang K, Jang SM, Kang S, Back K (2005) Enhanced neutraceutical serotonin derivatives of rice seed by hydroxycinnamoyl-CoA: serotonin N-(hydroxycinnamoyl) transferase. Plant Sci 168:783–788

Kang S, Kang K, Chung GC, Choi D, Ishihara A, Lee DS, Back K (2006) Functional analysis of the amine substrate specificity domain of pepper tyramine and serotonin *N*-hydroxycinnamoyltransferases. Plant Physiol 140:704–715

Kang S, Kang K, Lee K, Back K (2007a) Characterization of rice tryptophan decarboxylases and their direct involvement in serotonin biosynthesis in transgenic rice. Planta 227:263–272

Kang S, Kang K, Lee K, Back K (2007b) Characterization of tryptamine 5-hydroxylase and serotonin synthesis in rice plants. Plant Cell Rep 26:2009–2015

Kang K, Lee K, Sohn SO, Park S, Lee S, Kim SY, Kim YS, Back K (2009) Ectopic expression of serotonin *N*-hydroxycinnamoyltransferase and different production of phenylpropanoid amides in transgenic tomato tissues. Sci Hortic doi:1016/j.scienta.2008.12.015

Koyama N, Kuribayashi K, Seki T, Kobayashi K, Furuhata Y, Suzuki K, Arisaka H, Nakano T, Amino Y, Ishii K (2006) Serotonin derivatives, major safflower (*Carthamus tinctorius* L.) seed antioxidants, inhibit low-density lipoprotein (LDL) oxidation and atherosclerosis in apolipoprotein E-deficient mice. J Agric Food Chem 54:4970–4976

Koyama N, Kuribayashi K, Ishii K, Kobayashi K (2009) Composition for preventing atherosclerosis. US patent 07,485,328

Kumarasamy Y, Middleton M, Reid RG, Nahar L, Sarker SD (2003) Biological activity of serotonin conjugates from the seeds of *Centaurea nigra*. Fitoterapia 74:609–612

Lee DG, Park Y, Kim MR, Jung HJ, Seu YB, Hahm KS, Woo ER (2004) Anti-fungal effects of phenolic amides isolated from the root bark of *Lycium chinense*. Biotechnol Lett 26:1125–130

Lee K, Kang K, Park M, Woo YM, Back K (2008) Endosperm-specific expression of serotonin *N*-hydroxycinnamoyltransferase in rice. Plant Foods Hum Nutr 63:53–57

Ly D, Kang K, Choi JY, Ishihara A, Back K, Lee SG (2008) HPLC analysis of serotonin, tryptamine, tyramine, and the hydroxycinnamic acid amides of serotonin and tyramine in food vegetables. J Med Food 11:385–389

Martin-Tanguy J (1985) The occurrence and possible function of hydroxycinnamoyl acid amides in plants. Plant Growth Regul 3:381–399

Mijts BN, Schmidt-Dannert C (2003) Engineering of secondary metabolite pathways. Curr Opin Biotechnol 14:597–602

Murch SJ, KrishnaRaj S, Saxena PK (2000) Tryptophan is a precursor for melatonin and serotonin biosynthesis in in vitro regenerated St. John's wort (*Hypericum perforatum* L. cv. Anthos) plants. Plant Cell Rep 19:698–704

Nagatsu A, Zhang HL, Mizukami H, Okuyama H, Sakakibara J, Tokuda H, Nishino H (2000) Tyrosinase inhibitory and anti-tumor promoting activities of compounds isolated from safflower (*Carthamus tinctorius* L.) and cotton (*Gossypium hirsutum* L.) oil cakes. Nat Prod Lett 14:153–158

Niwa T, Etoh H, Shimizu A, Shimizu Y (2000) Cis-N-(p-coumaroyl) serotonin from konnyaku, *Amorphophallus konjac* K. Koch. Biosci Biotechnol Biochem 64:2269–2271

Noé W, Mollenschott C, Berlin J (1984) Tryptophan decarboxylase from *Catharanthus roseus* cell suspension cultures: purification, molecular and kinetic data of the homogenous protein. Plant Mol Biol 3:281–288

Park JB (2008) Serotomide and safflomide modulate forskolin-stimulated cAMP formation via 5-HT1 receptor. Phytomedicine 15:1093–1098

Park JB, Schoene N (2002) Synthesis and characterization of *N*-coumaroyltyramine as a potent phytochemical which arrests human transformed cells via inhibiting protein tyrosine kinases. Biochem Biophys Res Commun 292:1104–1110

Park M, Kang K, Park S, Back K (2008a) Conversion of 5-hydroxytryptophan into serotonin by tryptophan decarboxylase in plants, *Escherichia coli*, and yeast. Biosci Biotechnol Biochem 72:2456–2458

Park M, Kang K, Park S, Kim YS, Ha SH, Lee SW, Ahn MJ, Bae JM, Back K (2008b) Expression of serotonin derivative synthetic genes on a single self-processing polypeptide and the production of serotonin derivatives in microbes. Appl Microbiol Biotechnol 81:43–49

Pavlík M, Laudová V, Grüner K, Vokáč K, Harmatha J (2002) High-performance liquid chromatographic analysis and separation of *N*-feruloylserotonin isomers. J Chromatogr 770:291–295

RadWanski ER, Last RL (1995) Tryptophan biosynthesis and metabolism: biochemical and molecular genetics. Plant Cell 7:921–934

Roh JS, Han JY, Kim JH, Hwang JK (2004) Inhibitory effects of active compounds isolated from safflower (*Carthamus tinctorius* L.) seeds for melanogenesis. Biol Pharm Bull 27:1976–1978

Ryan MD, King AMQ, Thomas GP (1991) Cleavage of foot-and-mouth disease virus polyprotein is mediated by residues located within a 19 amino acid sequence. J Gen Virol 72:2727–2732

Sakamura S, Terayama Y, Kawakatsu S, Ichihara A, Saito H (1978) Conjugated serotonins related to cathartic activity in safflower seed (*Carthamus tinctorius* L.). Agric Biol Chem 42:1805–1806

Sarker SD, Laird A, Nahar L, Kumarasamy Y, Jaspars M (2001) Indole alkaloids from the seeds of *Centaurea cyanus* (Asteraceae). Phytochemistry 57:1273–1276

Schröder P, Abele C, Gohr P, Stuhlfauth-Roisch U, Grosse W (1999) Latest on the enzymology of serotonin biosynthesis in walnut seeds. Adv Exp Med Biol 467:637–644

Shoeb M, MacManus S, Jaspars M, Trevidu J, Nahar L, Kong-Thoo-Lin P, Sarker SD (2006) Montamine, a unique dimeric indole alkaloid, from the seeds of *Centaurea montana* (Asteraceae), and its in vitro cytotoxic activity against the CaCo2 colon cancer cells. Tetrahedron 62:11172–11177

Takii T, Hayashi M, Hiroma H, Chiba T, Kawashima S, Zhang HL, Nagatsu A, Sakakibara J, Onozaki K (1999) Serotonin derivative, N-(p-coumaroyl) serotonin, isolated from safflower (*Carthamus tinctorius* L.) oil cake augments the proliferation of normal human and mouse fibroblasts in synergy with basic fibroblast

growth factor (bFGF) or epidermal growth factor (EGF). J Biochem 125:910–915

Takii T, Kawashima S, Chiba T, Hayashi H, Hayashi M, Hiroma H, Kimura H, Inukai Y, Shibata Y, Nagatsu A, Sakakibara J, Oomoto Y, Hirose K, Onozaki K (2003) Multiple mechanisms involved in the inhibition of proinflammatory cytokine production from human monocytes by N-(p-coumaroyl) serotonin and its derivatives. Immunopharmacology 3:273–277

Tanaka E, Tanaka C, Mori N, Kuwahara Y, Tsuda M (2003) Phenylpropanoid amides of serotonin accumulate in witchs' broom diseased bamboo. Phytochemistry 64:965–969

Tozawa Y, Hasegawa H, Teruhiko T, Wakasa K (2001) Characterization of rice anthranilate synthase α-subunit genes *OASA1* and *OASA2*. Tryptophan accumulation in transgenic rice expressing a feedback-insensitive mutant OASA1. Plant Physiol 126:1493–1506

Watanabe M (1999) Antioxidative phenolic compounds from Japanese barnyard millet (*Echinochloa utilis*) grains. J Agric Food Chem 47:4500–4505

Wink M (1997) Special nitrogen metabolism. In: Dey PM, Harborne JB (eds) Plant Biochemistry. Academic, San Diego, pp 439–486

Yamamotová A, Pometlova M, Harmatha J, Raskova H, Rokyta R (2007) The selective effect of *N*-feruloylserotonins isolated from *Leuzea carthamoides* on nociception and anxiety in rats. J Ethnopharm 112:368–374

Yuji N, Naoto K, Katsuya S, Hideaki K, Yuka I (2007) Anti-inflammatory composition. PCT patent 2007129743

Zhang HL, Nagatsu A, Sakakibara J (1996) Novel antioxidants from safflower (*Carthamus tinctorius* L.) oil cake. Chem Pharm Bull 44:874–876
