# Supplementary material for: 5-Hydroxytryptophan (5-HTP): Natural Occurrence, Analysis, Biosynthesis, Biotechnology, Physiology and Toxicology
Source: Int J Mol Sci. 2020 Dec 26;22(1):181. doi: 10.3390/ijms22010181 (PMC7796270; doi:10.3390/ijms22010181)
Supplement: Supplementary file 1 [file ijms-22-00181-s001.zip › 5-HTP.Data/PDF/1242736920/Hoshino-1993-Formations-of--hydroxyindole-s---.pdf]

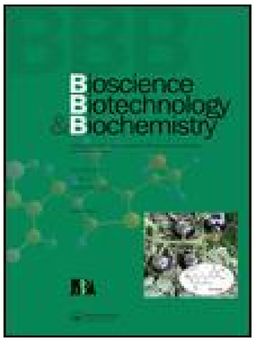


Taylor & Francis
Taylor & Francis Group

Bioscience, Biotechnology, and Biochemistry

ISSN: 0916-8451 (Print) 1347-6947 (Online) Journal homepage: https://www.tandfonline.com/loi/tbbb20

# Formations of (5-Hydroxy)indole *S*-(–)-lactic Acid, *N*-Acetyl-5-hydroxy-L-tryptophan, and (5-Hydroxy)indole Carboxylic Acid in the Metabolism of Tryptophan and 5-Hydroxytryptophan by *Chromobacterium violaceum*

Tsutomu Hoshino, Masahiro Yamamoto & Takeo Uchiyama

**To cite this article:** Tsutomu Hoshino, Masahiro Yamamoto & Takeo Uchiyama (1993) Formations of (5-Hydroxy)indole *S*-(–)-lactic Acid, *N*-Acetyl-5-hydroxy-L-tryptophan, and (5-Hydroxy)indole Carboxylic Acid in the Metabolism of Tryptophan and 5-Hydroxytryptophan by *Chromobacterium violaceum*, Bioscience, Biotechnology, and Biochemistry, 57:9, 1609-1610, DOI: 10.1271/bbb.57.1609

**To link to this article:** https://doi.org/10.1271/bbb.57.1609

Published online: 12 Jun 2014.

Submit your article to this journal

Article views: 179

View related articles

Citing articles: 7 View citing articles

Full Terms & Conditions of access and use can be found at
https://www.tandfonline.com/action/journalInformation?journalCode=tbbb20

*Biosci. Biotech. Biochem.*, **57** (9), 1609–1610, 1993

*Note*

# Formations of (5-Hydroxy)indole *S*-(−)-lactic Acid, *N*-Acetyl-5-hydroxy-L-tryptophan, and (5-Hydroxy)indole Carboxylic Acid in the Metabolism of Tryptophan and 5-Hydroxy-tryptophan by *Chromobacterium violaceum*

Tsutomu HOSHINO, Masahiro YAMAMOTO, and Takeo UCHIYAMA

*Department of Agricultural Chemistry, Faculty of Agriculture, Niigata University, Ikarashi, Niigata 950–21, Japan*
Received April 15, 1993

**Tryptophan metabolism catalyzing the production of (5-hydroxy)indolelactate, (5-hydroxy)indole carboxylate, and *N*-acetyl-5-hydroxytryptophan was found in *Chromobacterium violaceum*. These metabolites have never been reported before. Metabolic pathways from tryptophan and 5-hydroxytryptophan are proposed.**

The bacterium sp. *Chromobacterium violaceum* produces the blue pigment violacein by a series of tryptophan metabolisms. To data, some metabolites of tryptophan and its derivatives with this organism have been reported by other workers: indolepyruvic acid and indole acetic acid[1]; 5-hydroxy-,[2,3] and 6-hydroxytryptophan[4]; indole carboxylic acid[5]; from *N*-carbobenzoxyl(Cbz)-L-tryptophan to *N*-Cbz-dehydrotryptophan and from indole propionic acid to indole acrylic acid.[6] In addition to these metabolites, we found other reactions of tryptophan and/or 5-hydroxytryptophan. This was found from mutants, probably because stimulation of specified reactions occurred in the mutants. We report here the additional metabolisms of tryptophan and its 5-hydroxy analogue involved in *Chromobacterium violaceum*.

Colorless mutants were prepared with the mutagenic agent NTG as described previously.[7] A tryptophan solution was added to washed cells of mutant No. 8, then were incubated under the conditions described previously.[7] Only one metabolite, colored blue with Ehrlich reagent, was detected on TLC. This compound was isolated with reversed phase HPLC (C18) using the following mobile phase: MeOH–$H_2O$–HOAc (100:500:0.4), and identified as indolelactic acid by MS and NMR data[*1]. The conversion ratio from L-tryptophan to indolelactic acid was 65–70% by HPLC. The optimal pH was in the range of 6.5–8.0. The intermediate was identified as indolylpyruvic acid, because the incubation of indolepyruvic acid gave indolelactic acid in a yield of 55–60%. The substrate specificity was examined. 5-Hydroxy-L-tryptophan was incubated under the same conditions to give 5-hydroxy-indolelactic acid in a yield of 40–42%. In the cases of phenylalanine and phenylpyruvic acid, the production of the corresponding lactic acids were not observed, indicating that this biochemical reactions were limited to the indole rings. Optical recognition was also inspected. In cases of D-tryptophan and 5-hydroxy-D-tryptophan, yields of the corresponding lactic acids were 25–27% and 19–20%, respectively. These results are summarized in Table. Stereochemistry of the product was checked by a chiral column (Daicel Chiralpak WH) using a mobile phase of 0.5 mM $CuSO_4$–$CH_3CN$ (9:1). Commercial indolelactic acid was separated into two isomers (A and B), A being the faster fraction and B the slower one. The indolelactic acid prepared by the incubations of both D- and L-tryptophan showed only the A peak, but did not any peak B, suggesting that the reduction process *via* indolylpyruvic acid is completely stereospecific (~100% *e.e.*). The $[\alpha]_D^{25}$ of the indolelactic acid thus isolated from No. 8 was −4.4 (*c* 0.3, MeOH), indicating the L-configuration of indolelactic acid, *i.e.*, *S*-(−)-indolelactic acid.[8] This enzyme reaction was also expressed in a cell-free system prepared by ultrasonication for 5 min. Addition of NAD(P)H (10 mM) enhanced the production of indolelactic acid by about 20%, when compared to that with no addition. The production of indolylpyruvic acid from indoleclatic acid was not found in the presence of excess $NAD(P)^+$, indicating that the formation of indolelactate is dominant. There are several reports related to the metabolism of indolelactic acid; indolelactate dehydrogenase from *Clostridium sporogenes*[9] and an aromatic α-keto acid reductase from a mammal.[10] Substrate specificity from these two sources is not so restricted to aromatic rings, *i.e.*, these are also active on phenyl rings (*e.g.*, phenylpyruvate↔phenyllactate). However, the same reaction of mutant No. 8 was strictly limited to the indole rings (Table). This may be characteristic of this organism. The enzyme catalyzing tryptophan to indolepyruvate is not clear; an amino acid oxidase, transaminase, or dehydrogenase may be involved, and tryptophan racemase may be also responsible for the reaction because

**Table** Conversion Ratio of Aromatic Amino Acid Analogues to the Corresponding Lactic Acids and the Substrate Specificity in Metabolism with Mutant No. 8

| Substrates | Conversion ratio (%) |
|---|---|
| L-Trp | 65–70 |
| D-Trp | 25–27 |
| I-pyA | 55–60 |
| 5-OH-L-Trp | 40–42 |
| 5-OH-D-Trp | 19–20 |
| L-Phe, D-Phe | ≅0 |
| Phe-pyA | ≅0 |

Abbreviations are as follows: Trp for tryptophan, I-pyA for indolepyruvic acid, 5-OH-Trp for 5-hydroxytryptophan, Phe for phenylalanine, and Phe-pyA for phenylpyruvic acid.

Incubation conditions: The cells were harvested after growing for 12 h. To the harvested cells from 200 ml of culture broth, 2.5 mg of each compound was added in pH 7.2 phosphate buffer, and incubated for 24 h with agitation on a rotary shaker at 180 rpm and 25°C (see also ref. 7).

*1 MS: *m/z* 205 ($M^+$); $\delta_C$ (DMSO-$d_6$): 31.52, 73.34, 111.14, 113.06, 119.4, 119.52, 122.16, 124.6, 128.95, 137.95, 177.54.

*2 MS: *m/z* 161 ($M^+$), NMR (DMSO-$d_6$): $\delta_H$; 11.93 (1H, br. s), 11.82 (1H, br. s), 8.02 (2H, m), 8.00 (1H, d, $J$=2.9 Hz), 7.48 (1H, m), 7.18 (2H, m): $\delta_C$; 166.0 (s), 136.6 (s), 132.3 (d), 126.2 (s), 122.3 (d), 121.1 (d), 120.7 (d), 112.3 (d), 107.6 (s).

*3 MS: *m/z* 177 ($M^+$), NMR (DMSO-$d_6$): $\delta_C$; 165.0, 151.3, 131.1, 129.6, 126.1, 111.5, 111.3, 105.6, 103.9.

*4 MS: *m/z* 276 ($M^+$), NMR ($CD_3OD$-$d_4$): $\delta_H$; 7.15 (1H, d, $J$=8.8 Hz), 7.00 (1H, s), 6.89 (1H, d, $J$=2.0 Hz), 6.66 (1H, dd, $J$=8.8, 2.0 Hz), 4.69 (1H, dd, $J$=5.9, 7.8 Hz), 3.66 (3H, s), 3.19 (1H, dd, $J$=14.6, 5.9 Hz), 3.08 (1H, dd, $J$=14.6, 7.8 Hz), 1.93 (3H, s): $\delta_C$; 174.1 (s), 173.3 (s), 151.4 (s), 133.3 (s), 129.5 (s), 125.1 (d), 112.8 (d), 112.5 (d), 109.9 (s), 103.3 (d), 54.9 (d), 52.7 (q), 28.6 (t), 22.3 (q).

NII-Electronic Library Service

D-tryptophan was also active. To make sure, enzyme purification would be necessary.

The washed cells of mutant No. 9 produced chromopyrrolic acid[7] and an unknown metabolite, colored pink with Ehrlich reagent. EIMS and NMR data of the pink compound*2 suggested indole 3-carboxylic acid and were indistinguishable from those of the authentic one in all respects. With 5-hydroxy-L-tryptophan, two major products were found; one was pink and the other dark blue with Ehrlich reagent. The pink compound was identified as 5-hydroxyindole-3-carboxylic acid (isolation yield; about 7%) by means of the spectroscopic data.*3 The alternative metabolite, dark blue, was also acidic (isolation yield; 5.4%). The methylated product*4 was prepared with diazomethane. The 2D NMR indicated the metabolite to be *N*-acetyl-5-hydroxytryptophan, and it was ascertained by the synthesis of *N*-acetyl-5-hydroxytryptophen, prepared with $Ac_2O$/NaOH. The stereochemistry of the metabolite was L-or *S*-configuration, because it was completely hydrolyzed to give 5-hydroxy-L-tryptophan with L-aminoacylase. In the incubation experiment with tryptophan, *N*-acetyltryptophan was not found in spite of rigorously searching for it, suggesting that this acetylation, probably *via* CoASAc, was active only with 5-hydroxytryptophan, but inert to tryptophan. All the biochemical reactions except for indolelactate were only active with the L-configuration.

All materials of the mutants described here were extracellular metabolites and detected also with the parent strain. However, the amounts from the parent strain were smaller, specifically, indolelactate was negligible. For indole carboxylic acid, the conversion ratios by the mutant and the parent strain were 24.5% and 6.5%, respectively, measured by tracer experiments of [3-$^{14}$C]tryptophan. A given metabolism would have been enhanced in the mutants due to the block of the various reactions involved in the parent strain. The metabolic pathway to indole carboxylic acid analogues was considered to be as follows: (5-hydroxy)tryptophan→(5-hydroxy)indole acetic acid→(5-hydroxy)indole carboxaldehyde→(5-hydroxy)indole carboxylic acid; these were isolated at different incubation intervals and analyzed by MS spectroscopies and/or TLC in comparison with those of the authentic samples, and the intermediacy was also verified by a feeding experiment of each compound.

The mutant preparation was intended for searching for the biosynthetic intermediates of violacein. All the metabolites labeled with C-13 and C-14, prepared with [3-$^{13}$C] or [3-$^{14}$C] (5-hydroxy)tryptophan by using washed cells of the mutants, were administered to growing cells or washed cells of the parent strain. However, all the incorporation experiments failed, indicating that these materials are not the biosynthetic intermediates and are produced independently of the biosynthesis of violacein analogues.

To the best of our knowledge, the formation of (5-hydroxy)-indolelactic acid, 5-hydroxyindole carboxylic acid, and *N*-acetyl-5-hydroxytryptophan have never been reported so far in this bacterium. In conclusion, *Chromobacterium violaceum* has the versatility in metabolizing tryptophan and its 5-hydroxy analogue, as seen by this and the reports of other workers.[1-6]

## References

1) R. D. DeMoss and N. R. Evans, *Bacterial Proc.*, **1957**, 117.
2) C. Mitoma, H. Weissbach, and S. Udenfriend, *Arch. Biochem. Biophys.*, **63**, 122–130 (1956).
3) C. H. Letendre, G. Dickens, and G. Guroff, *J. Biol. Chem.*, **249**, 7186–7191 (1974).
4) S. F. Contractor, M. Sandler, and J. Wragg, *Life Sci.*, **3**, 999–1006 (1964).
5) P. J. Davis, M. E. Gustafson, and J. P. Rosazza, *J. Bacteriol.*, **126**, 544–546 (1976).
6) P. J. Davis, M. E. Gustafson, and J. P. Rosazza, *Biochim. Biophys. Acta*, **385**, 133–144 (1975).
7) T. Hoshino, Y. Kojima, T. Hayashi, T. Uchiyama, and K. Kaneko, *Biosci. Biotech. Biochem.*, **57**, 775–781 (1993).
8) F. Aragozzini, A. Ferrari, N. Pacini, and R. Gualandris, *Appl. Envir. Mirobiol.*, **38**, 544–546 (1979).
9) M. Jean and R. D. Demoss, *Can. J. Microbiol.*, **14**, 429–435 (1668).
10) V. G. Zannoni, and W. W. Weber, *J. Biol. Chem.*, **241**, 1340–1344, 1345–1349 (1966).

NII-Electronic Library Service
