# Supplementary material for: 5-Hydroxytryptophan (5-HTP): Natural Occurrence, Analysis, Biosynthesis, Biotechnology, Physiology and Toxicology
Source: Int J Mol Sci. 2020 Dec 26;22(1):181. doi: 10.3390/ijms22010181 (PMC7796270; doi:10.3390/ijms22010181)
Supplement: Supplementary file 1 [file ijms-22-00181-s001.zip › 5-HTP.Data/PDF/1280963201/Zhao-2019-Melatonin-synthesis-and-function-ev.pdf]

frontiers in Endocrinology

REVIEW
published: 17 April 2019
doi: 10.3389/fendo.2019.00249

# Melatonin Synthesis and Function: Evolutionary History in Animals and Plants

**Dake Zhao[1,2,3†], Yang Yu[4†], Yong Shen[5†], Qin Liu[6], Zhiwei Zhao[4], Ramaswamy Sharma[7] and Russel J. Reiter[7*]**

[1] Biocontrol Engineering Research Center of Plant Disease and Pest, Yunnan University, Kunming, China, [2] Biocontrol Engineering Research Center of Crop Disease and Pest, Yunnan University, Kunming, China, [3] School of Life Science, Yunnan University, Kunming, China, [4] State Key Laboratory for Conservation and Utilization of Bio-resources in Yunnan, Yunnan University, Kunming, China, [5] College of Agriculture and Biotechnology, Yunnan Agricultural University, Kunming, China, [6] School of Landscape and Horticulture, Yunnan Vocational and Technical College of Agriculture, Kunming, China, [7] Department of Cell Systems and Anatomy, The University of Texas Health Science Center at San Antonio (UT Health), San Antonio, TX, United States

Melatonin is an ancient molecule that can be traced back to the origin of life. Melatonin's initial function was likely that as a free radical scavenger. Melatonin presumably evolved in bacteria; it has been measured in both α-proteobacteria and in photosynthetic cyanobacteria. In early evolution, bacteria were phagocytosed by primitive eukaryotes for their nutrient value. According to the endosymbiotic theory, the ingested bacteria eventually developed a symbiotic association with their host eukaryotes. The ingested α-proteobacteria evolved into mitochondria while cyanobacteria became chloroplasts and both organelles retained their ability to produce melatonin. Since these organelles have persisted to the present day, all species that ever existed or currently exist may have or may continue to synthesize melatonin in their mitochondria (animals and plants) and chloroplasts (plants) where it functions as an antioxidant. Melatonin's other functions, including its multiple receptors, developed later in evolution. In present day animals, via receptor-mediated means, melatonin functions in the regulation of sleep, modulation of circadian rhythms, enhancement of immunity, as a multifunctional oncostatic agent, etc., while retaining its ability to reduce oxidative stress by processes that are, in part, receptor-independent. In plants, melatonin continues to function in reducing oxidative stress as well as in promoting seed germination and growth, improving stress resistance, stimulating the immune system and modulating circadian rhythms; a single melatonin receptor has been identified in land plants where it controls stomatal closure on leaves. The melatonin synthetic pathway varies somewhat between plants and animals. The amino acid, tryptophan, is the necessary precursor of melatonin in all taxa. In animals, tryptophan is initially hydroxylated to 5-hydroxytryptophan which is then decarboxylated with the formation of serotonin. Serotonin is either acetylated to *N*-acetylserotonin or it is methylated to form 5-methoxytryptamine; these products are either methylated or acetylated, respectively, to produce melatonin. In plants, tryptophan is first decarboxylated to tryptamine which is then hydroxylated to form serotonin.

**Keywords: melatonin, evolution, antioxidant, biological rhythms, biosynthesis enzymes, endosymbiosis, regulation of melatonin**

OPEN ACCESS

***Edited by:***
*Ralf Jockers, Université Paris-Sorbonne, France*

***Reviewed by:***
*Maristela Oliveira Poletini, Federal University of Minas Gerais, Brazil*
*Yves Combarnous, Centre National de la Recherche Scientifique (CNRS), France*

***Correspondence:**
*Russel J. Reiter*
*reiter@uthscsa.edu*

*†These authors have contributed equally to this work*

***Specialty section:***
*This article was submitted to Cellular Endocrinology, a section of the journal Frontiers in Endocrinology*

***Received:*** *06 December 2018*
***Accepted:*** *29 March 2019*
***Published:*** *17 April 2019*

***Citation:***
*Zhao D, Yu Y, Shen Y, Liu Q, Zhao Z, Sharma R and Reiter RJ (2019) Melatonin Synthesis and Function: Evolutionary History in Animals and Plants. Front. Endocrinol. 10:249. doi: 10.3389/fendo.2019.00249*

# INTRODUCTION

After its isolation and identification in the pineal gland of the cow, in subsequent years melatonin was identified in a wide variety of animals and plants (1–7). The extensive distribution of melatonin, especially in the primitive bacteria (cyanobacteria and α-proteobacteria) indicates that the chemical is an ancient molecule that has been retained throughout the evolution of all organisms (8, 9) (**Figure 1**). It is speculated that melatonin evolved in bacteria prior to the process referred to as endosymbiosis. After cyanobacteria and α-proteobacteria were engulfed by early prokaryotes, they eventually evolved into chloroplasts and mitochondria, respectively, such that all unicellular and multicellular organisms ultimately produce this critical indoleamine in these organelles (11–13). With organismal diversification, melatonin universally spread to all organisms and, accordingly, its functions, biosynthetic pathway, generation sites and biosynthetic regulation have also diverged.

The proposed initial function of melatonin was to detoxify free radicals generated during the processes of photosynthesis and metabolism (8, 14–17). With the bio-divergence during organismal evolution, melatonin became a pleiotropic molecule that resists oxidation-related stress but also influences biological rhythms, suppresses inflammation, etc. (18–21).

The genes encoding the biosynthetic enzymes for melatonin have been identified in a number of species; these proteins potentially catalyze different substrates further determining the diverse biosynthetic routes of melatonin (22, 23). The multiple biosynthetic pathways provide direct evidence for melatonin's evolution. From unicellular to multicellular organisms, the subcellular localization of the enzymes related to melatonin biosynthesis may have changed somewhat (24, 25). The separation of the sites of subcellular localization may have been beneficial for the efficient control of melatonin synthesis (22, 26–28).

To exploit the multiple functions of melatonin, organisms developed various mechanisms to regulate its biosynthesis. For example, when faced with stress, activator protein-1 (AP-1), a transcription factor, promotes the synthesis of melatonin via up-regulating melatonin synthesis genes (29–34). Based on its evolutionary history, it seems clear that melatonin not only kept its primary function as an antioxidant but extended its functions to other important biological actions. Moreover, since it co-habitated with other key molecules such as sirtuins for eons, melatonin also learned to functionally cooperate with them (13).

# FUNCTIONAL EVOLUTION OF MELATONIN

Molecular oxygen ($O_2$) began to rise in the Earth's atmosphere (the Great Oxygenation Event) (**Figure 2**) around 2.5 billion years ago due to its persistent release from photosynthetic bacteria that had evolved an estimated billion years earlier (35–37). The rise of atmospheric $O_2$ was a highly selective pressure for the evolution of organisms to use $O_2$ as the basis of their metabolism (38, 39). During aerobic metabolism, reactive oxygen species (ROS) are invariably generated when $O_2$ accepts leaked electrons from the electron transport chain (ETC) (24, 40, 41).

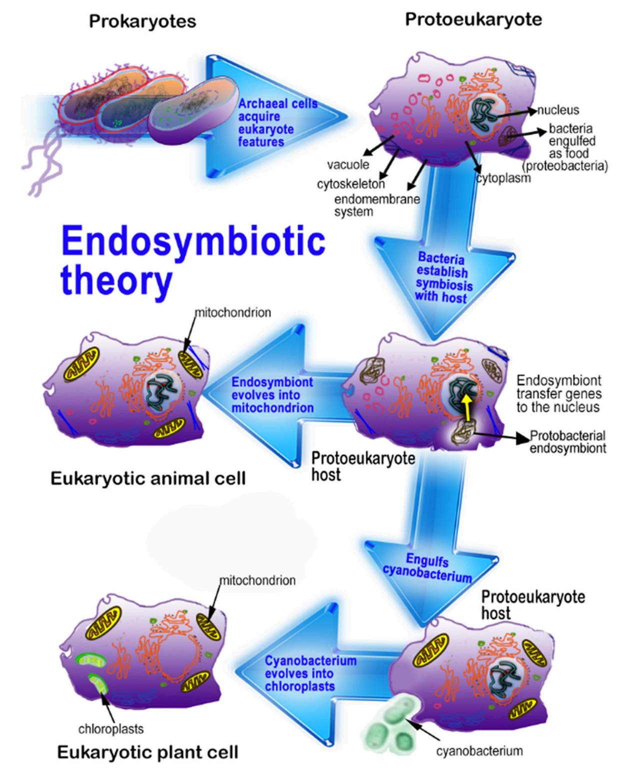


**FIGURE 1 |** This figure illustrates the endosymbiotic origin of mitochondria and chloroplasts. α-Proteobacteria, originally phagocytized for their nutrient value by early eukaryotes eventually evolved into mitochondria. Photosynthetic cyanobacteria were likewise phagocytized by eukaryotes and eventually formed chloroplasts. Since plants have both mitochondria and chloroplasts, plant cells generally have higher concentrations of melatonin than do animal cells. Adapted from Reiter et al. (10).

It is estimated that up to 4% of the $O_2$ consumed by organisms during the aerobic metabolism eventually is reduced to ROS (42, 43). These large amounts of ROS are toxic to cells and organisms, inducing the development of complex and effective mechanisms to neutralize them; this initially occurred in early life forms such as bacteria and subsequent unicellular organisms (8, 44). To control oxidative stress, melatonin presumably emerged primarily as an antioxidant and free radical scavenger in early photosynthetic prokaryotic bacteria (12, 13, 45, 46). Melatonin has retained, until the present time and in all organisms, its ability to control oxidative stress that results from free radical production that occurs during photosynthesis and respiration (8, 47–49).

The special structure of melatonin determines its high efficiency in detoxifying free radicals based on its ability to donate an electron or a hydrogen atom, or depending on the radical type, potentially by other means as well (5, 15, 17). The superior antioxidant capacity of melatonin to limit oxidative stress is, at least partially, attributed to what is referred to as the cascade reaction which occurs when it generates derivatives that are likewise free radical scavengers (12, 15, 50–52). Melatonin interacts with a variety of ROS to produce cyclic 3-hydroxymelatonin and other melatonin metabolites,

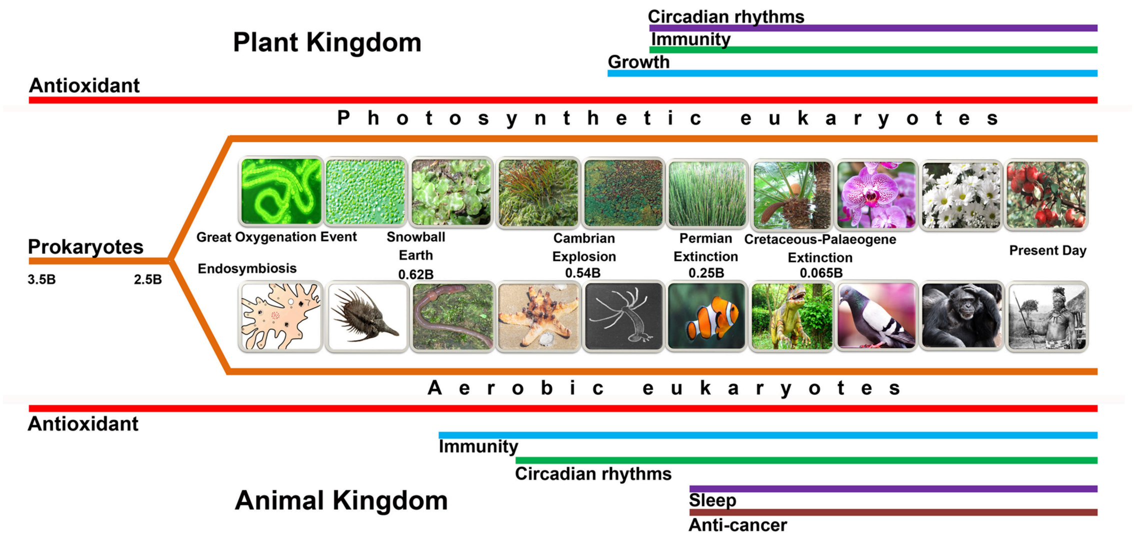


**FIGURE 2 |** This figure summarizes the possible evolution of various functions (not all are depicted in this figure) of melatonin. Melatonin, predictably, initially evolved in bacteria for the purpose of mitigating oxidative stress, i.e., as an antioxidant (red lines). When the bacteria were phagocytized as food by early eukaryotes, they eventually developed a mutually beneficial association with their hosts and evolved into mitochondria and chloroplasts (see **Figure 1**); this series of events is referred to as endosymbiosis. Subsequently, as evolution proceeded, mitochondria (animals and plants) and chloroplasts (plants) were preserved up until the present day. Thus, mitochondria and chloroplasts of every species that has ever existed or exists today, we theorize, presumably produce melatonin. This presumption is supported by recent findings which show that these organelles, in many cases, possess the necessary synthetic machinery to generate melatonin. Melatonin's role as an antioxidant in these organelles is of great importance since they are sites of major free radical production. Other colored lines, which are appropriately labeled, identify other functions of melatonin. It is essential that the time frame for these functions, as illustrated by the length of the colored lines, do not accurately depict the time of evolution of these functions. Major events in the history of the Earth are also identified. The "B" following the numbers refers to "billions of years ago.".

e.g., *N*1-acetyl-*N*2-formyl-5-methoxykynuramine and *N*-acetyl-5-methoxykynuramine (53–56). These metabolites function as radical scavengers, sometimes even more aggressively than melatonin regarding their capacity to neutralize ROS (15, 57).

Despite its very long evolutionary history and its multiple functions, the chemical structure of melatonin has remained unchanged for billions of years (13). Moreover, melatonin may have been retained by all organisms even with their very wide biodiversification during evolution. This relates to the conservation of mitochondria and chloroplasts (or both) in most cells of all organisms. One exception is red blood cells which, during erythropoiesis, eject certain organelles including mitochondria.

As already noted, melatonin originally exclusively functioned as an antioxidant in primitive bacteria; however, over billions of years of evolution it became a pleiotropic molecule in multicellular organisms (**Figure 2**). The development of new functions of melatonin logically expanded the spectrum of its antioxidant activity (8). Regulation of biological rhythms is one of the key functional extensions. In early primitive unicellular plants and animals, more free radicals were produced during the photophase than during the scotophase; thus, larger amounts of melatonin were presumably consumed during the detoxification of excessively-produced free radicals during the day (58, 59) resulting in a diurnal rhythm of melatonin. In contrast to unicellular organisms, which directly perceive photoperiodic changes and synchronize their biological activities accordingly (60), complex multicellular organisms could no longer respond directly to the photoperiodic changes (24). A signaling molecule, therefore, was required to ensure the photic information was transduced into a circadian signal for all cells (61). The alteration in melatonin levels in bacteria due to its differential utilization as a scavenger accurately reflected the photoperiodic changes of the light/dark cycle; theoretically, multiple organisms adopted the melatonin cycle as a signaling system for this purpose (24, 62, 63).

Multicellular organisms, therefore, co-opted a melatonin rhythm that already existed; but rather than depending on the metabolic utilization of melatonin to determine the cycle, they developed the subcellular framework to produce more melatonin during the scotophase than during the photophase, thereby ensuring a day:night melatonin cycle. In most vertebrates, but seemingly not all (64), this required the evolution of the pineal gland which is the location of the circadian production and, importantly, cyclic secretion of melatonin allowing all cells access to light:dark information. In present day animal species, the melatonin cycle, with highest levels at night, is the same regardless of the activity pattern of the species, i.e., nocturnal, diurnal or crepuscular. In addition to the neural connections between the eyes and the pineal gland, most clearly described in mammals, the pineal of some lower vertebrates responds directly to light stimuli (65). While the photic information has a direct impact on the electrophysiology of the organ, there is no proof that it alters melatonin production or secretion. In non-vertebrate animals and in plants, much less is known about the circadian production of melatonin (66, 67), although

these species do exhibit other circadian rhythms (68, 69) as well as possible 24 h fluctuations in melatonin, but sometimes the highest levels occur during the day (70).

Other actions of melatonin that have evolved and relate to the antioxidant activity of melatonin include retarding some age-related processes, anti-inflammatory activity, resisting neurodegenerative changes, the prevention of apoptosis in normal cells, and the preservation of mitochondrial and chloroplast physiology (8, 71–74) (**Figure 2**). These functions are associated, at least in part, with melatonin's ability to neutralize free radicals.

The actions of melatonin in different species have clearly diverged during the differentiation of major animal and plant taxa. These functions show a close relationship with the characteristics of the specific taxa. In mammals, melatonin is a molecule with hormonal properties (10, 75–77). The hormonal properties of melatonin are apparent in the regulation of seasonal reproductive activity, facilitation of sleep physiology, promotion of immunoresponsiveness, suppression of carcinogenesis, promotion of stem cell proliferation, anti-inflammation, and modulating aging (73, 78–83). Some of these actions are surely mandated by the interaction of melatonin with cell membrane receptors, i.e., MT1 and MT2, and/or perhaps with nuclear binding sites and are, therefore, considered hormonal (see below). For example, melatonin's ability to constrain cancer cell proliferation often involves membrane receptors (84, 85). There is also evidence, however, that receptor-independent actions such as its free radical generating capacity ("pro-oxidant"), an action possibly unique to cancer cells (86), also kills tumor cells (87, 88). Moreover, melatonin's multiple means by which it limits cancer metastases have not been unambiguously shown to be receptor-mediated (89). Thus, in mammals, melatonin is not a typical hormone and functions via receptor-dependent and receptor-independent means. On the evolutionary scale, the free radical scavenging properties, which continue to exist in mammalian cells, preceded the evolution of receptors for this indoleamine; thus, the initial actions of melatonin were receptor- independent.

In early non-mammalian vertebrates, the pineal organ directly responded to light that penetrated a cartilaginous plate overlying the epithalamus (90). This photic information was detected by photoreceptive elements similar to those in the retinas, with the electrical messages being sent to adjacent neural structures (91). In higher vertebrates, the pineal gland is no longer directly light-sensitive, although it does contain evolutionary morphological remnants of photoreceptive rods/cones (92), but it remains influenced by light and darkness via complex retina-suprachiasmatic nucleus-sympathetic neural connections (93).

The mammalian pineal gland probably did not evolve as nor should it be strictly classified as an endocrine gland. Endocrine glands are typically regulated by the secretory products from other glands and exhibit either feedback or feedforward responses when contacted by these agents. Also, because of their primary regulation by hormones, hormone production and secretion typically are only modestly impacted by depriving endocrine glands of their sympathetic innervation. These features are in marked contrast with those of the pineal gland, where other hormones have barely perceptible effects on pineal melatonin production (94, 95). The sympathetic denervation destroys the function of the pineal (96), while for other endocrine organs (e.g., anterior pituitary, thyroid gland) denervation is essentially inconsequential. Giving norepinephrine or isoproterenol to animals in which the pineal has been sympathetically denervated induces a rapid increase in pineal melatonin synthesis indicating that pineal synthetic processes are under the control of the nervous system rather than by hormones (97); there may, however, be some unique experimental conditions that perturb melatonin synthesis in the pineal gland independent of its innervation (98); how these actions are mediated remain unknown. For example, high melatonin production can be shifted to immune competent cells (99) under inflammatory conditions. This important observation was unanticipated and opens up the possibility that there may be other conditions under which melatonin synthesis is enhanced in other cell types.

Another argument against melatonin being exclusively a hormone comes from the observation that an estimated 99% of the melatonin in vertebrates is likely not produced in the pineal gland and is never released into the circulation. The discovery of melatonin in mitochondria, where it likely functions as a direct free radical scavenger and as an indirect antioxidant, means that the total quantity of melatonin synthesized in vertebrates is much greater than originally envisioned. In addition to functioning as a scavenger at the site at which it is produced, i.e., mitochondria, melatonin generated at the subcellular level may be locally released to function as a paracrine or autocrine agent (100). There is also preliminary evidence that mitochondria-produced melatonin is discharged from this organelle after which it interacts with receptors on the outer mitochondrial membrane where it may influence the release of cytochrome c (101) (**Figure 3**). In invertebrates (104) which lack a pineal gland, endogenous melatonin production may respond to external environmental alterations that do not involve the light:dark cycle.

In the plant kingdom, melatonin is demonstrated to be a multi-regulatory molecule with diverse functions in plant growth and development, such as seed protection and germination, root development, fruit ripening, and senescence (105–108). Compared to animals, plants face more environmental challenges because of their sessile nature. As a protection against these stresses, they rapidly unregulate melatonin synthesis which then functions in the protection against oxidative stress induced by these challenges (109). As mentioned, melatonin works independent of receptors when it clears ROS (110, 111). The major membrane melatonin receptors in animals, MT1 and MT2, activate different signaling cascades to improve or antagonize biological effects (112–115). To date, only one phytomelatonin receptor (CAND2/PMTR1) has been identified; it regulates stomatal closure via a $H_2O_2$ and $Ca^{2+}$signaling transduction cascade (116). In addition, phytomelatonin can interact with unknown receptors with active $H_2O_2$/NO signaling pathways, and further improve plant stress tolerance by activating a variety of antioxidant enzymes, alleviating photosynthesis inhibition, and modulating transcription factors; these transcription factors are involved with stress resistance, chelating and promoting transport of heavy metals, or activating other stress-relevant

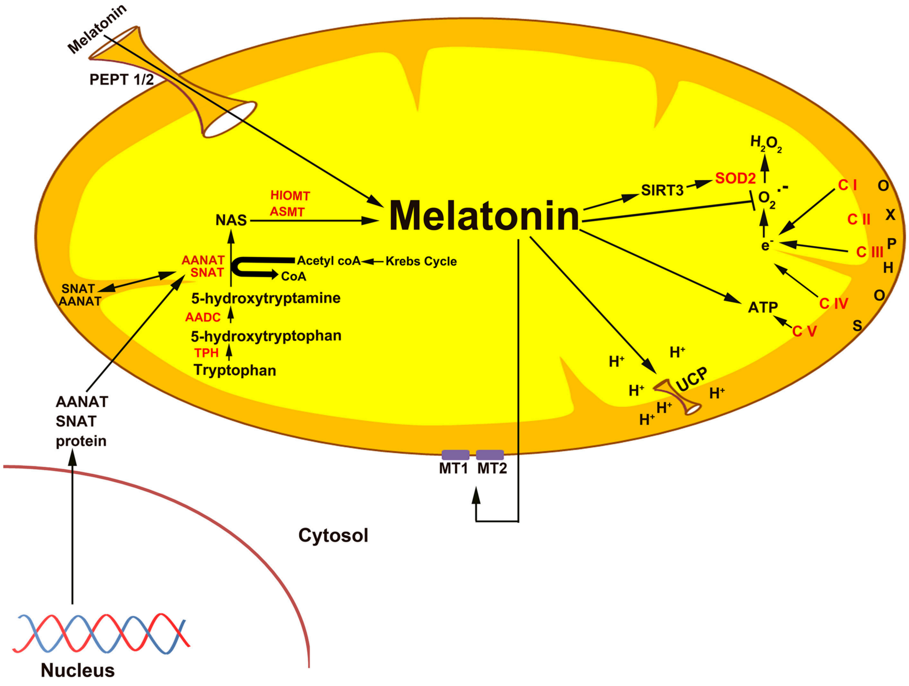


**FIGURE 3 |** The association of melatonin with mitochondria is predicted on the basis of the origin of these organelles as specified in the text. Current evidence suggests that melatonin is synthesized in some species in the mitochondrial matrix as illustrated here. Also, exogenously administered melatonin concentrates in the mitochondria (102), i.e., melatonin is a mitochondria-targeted agent. Given that melatonin functions as an antioxidant is particularly important in mitochondria since these organelles are a major site of free radical generation. In addition to directly neutralizing reactive oxygen species, melatonin also stimulates the antioxidant enzyme superoxide dismutase (SOD2), an action that involves an elevated level of sirtuin 3 (SIRT3) (39). Melatonin potentially enters mitochondria through the oligopeptide transporters, PEPT1/2 (103). Melatonin also influences mitochondrial membrane potential by influencing uncoupling protein (UCP). Also, melatonin from the matrix may leak out of the mitochondria to interact with the melatonin receptors, MT1 and MT2, to control the release of cytochrome c.

hormones such as salicylic acid, ethylene, and jasmonic acid (109, 117, 118). In animals, melatonin also binds directly to the catalytic site of quinone reductase 2 (QR2, E.C. 1.10.99.2), a cytosolic molecule, to modulate the activity of this enzyme; that modulation may be either up or down regulation (119, 120). Importantly, the change in QR2 activity may further play a key role in ROS generation or detoxification (121).

## ORIGIN OF MELATONIN RECEPTORS

The presumed original function of melatonin, i.e., as a direct free radical scavenger, required nothing of the cell except positioning melatonin in close proximity to where the bulk of the ROS are usually formed. Such positioning of an antioxidant is essential since free radicals have an extremely short half-life and instantaneously damage molecules in the immediate neighborhood of where they are formed. If a free radical scavenger is not properly situated, it cannot prevent the initial damage inflicted by a highly reactive radical. To accomplish this proper placement, evolution arranged for the uptake by and synthesis of melatonin in mitochondria (101, 102) and chloroplasts (108, 122), both major contributors to the total oxidative burden of cells.

In currently-surviving vertebrates, melatonin has a very extensive physiological toolkit. To broaden its functional repertoire, it was necessary for its binding sites/receptors and associated signaling transduction processes to also evolve. Many of the currently known activities of melatonin are mediated by G-protein coupled receptors in the membranes of animal cells (113, 123, 124). The best known receptors associated with cell membranes are members of the G-protein coupled receptor family (111, 125, 126); they are designated MT1 and MT2 (127–129).

Perhaps, of special relevance to the current discussion, is the finding that the MT1 receptor, generally considered to be confined to the limiting membrane of cells has also been recently associated with the outer membrane of mitochondria (101). According to the researchers who made this discovery, melatonin from the mitochondrial matrix diffuses out of these structures and interacts with MT1 receptors on the outer membrane of these organelles; they coined the term "automitocrine" to define this process. Via this receptor-mediated pathway, mitochondria-generated melatonin may control the release of cytochrome c from the matrix. This self-regulatory process has implications for apoptosis resulting from extensive free radical damage.

In addition to the well-characterized and highly relevant cell membrane receptors which are indispensable for a number of

melatonin's key functions, there are also binding sites in the cytosol (130) and in the nucleus (131–133). In the cytosol, the enzyme quinone reductase 2 (QR2) has been designated as receptor MT3 (134). The activity of this detoxifying enzyme may to be related to some of the actions of melatonin in reducing oxidative damage. Melatonin also couples with calmodulin in the cytosol, an action that is reportedly linked to the cancer-inhibitory effect of the indoleamine (135, 136).

In an invertebrate, the crayfish (*Procambarus clarkii*), melatonin functions in the modulation of the reticular photoreceptor potential amplitude with the intensity change differing between the day and night (137). With the aid of the commonly used melatonin receptor blockers, the authors deduced that the actions of melatonin on visual photoreceptors are mediated by a site reminiscent of the mammalian MT2 receptor. In another crustacean, the crab (*Neohelice granulata*), some of the metabolic actions of melatonin are inhibited by luzindole, a classic MT1/MT2 receptor blocker (138).

In the honey bee, *Apis cerana*, a melatonin receptor with the typical seven transmembrane domains has been characterized (139); the authors named it AccMTNR1A. It mediates the response of this species to cold stress, a feature that is common with that of plants. Silencing of the receptor also interfered with the transcription of some antioxidant signaling pathways. This illustrates that antioxidant enzyme activity may be regulated in the honey bee as they are in plants (105). Collectively, the data from invertebrates show that not only do they produce melatonin but they probably have receptors that mediate some of its actions. Fossil records indicate that insects evolved more than 400 million years ago, so the melatonin receptor has likely existed for at least the same time duration.

*Tetrahymena* are nuclear dimorphic, unicellular, ciliated eukaryotes. Like many other eukaryotes, *Tetrahymena* feed on bacteria so this evolutionarily-early organism would be expected, due to endosymbiosis, to have retained the melatonin synthetic potential of the engulfed bacteria. There is evidence that *Tetrahymena* contain biogenic amines including possibly melatonin (140). Whether this species possesses melatonin binding sites/receptors has not been established. If they are found to contain melatonin-binding molecules, it would show that some type of melatonin receptor evolved about one million years after melatonin arose. As it currently stands, there is little information related to when melatonin receptors originated.

As noted above, melatonin was discovered in land plants about 25 years ago (2, 3) where it functions as an antioxidant in a receptor-independent manner (141) as in animals. The first phytomelatonin receptor, designated CAND2/PMTR1, was described in *Arabidopsis thaliana* (116). This receptor is located on epithelial cells which govern the closure of the leaf stomata. For this process, the signal transduction cascade involves Gα subunit–activated $H_2O_2$ production and $Ca^{2+}$ signaling. It is estimated that land plants came into existence on Earth about 200 million years ago. Whether the first land plant that appeared or any plants that preceded them possessed melatonin receptors is unknown.

In addition to the direct scavenging of radicals and radical products by melatonin, land plants also have many of the antioxidant enzymes that exist in animals. In plants, the enzymes are melatonin-influenced and are quickly upregulated when the plant is exposed to an abiotic stress, e.g., draft, heat, cold, toxin, etc. (105, 142). It is presumed that this upregulation involves melatonin receptors as is likely the case for animals as well.

For additional details on the pharmacological characterization, cloning and signal transduction pathways of melatonin receptors, the reader is referred to comprehensive reviews of this subject by the groups of Jockers et al. (114, 115), Oishi et al. (130), Tosini et al. (143), Liu et al. (128), Dubocovich (114). Obviously, melatonin receptors developed subsequent to the evolution of melatonin. Based on what is currently known of their distribution, they probably originated with the origin of multicellular organisms, both plant and animal.

## DIVERGENCE OF MELATONIN BIOSYNTHESIS IN DIFFERENT TAXA

Melatonin is believed to exist in all living organisms including bacteria, yeasts, fungi, animals, and plants (144, 145). This molecule is formed exclusively from the amino acid tryptophan (146). While tryptophan is consumed in the diet, it can also be synthesized via the shikimic acid pathway starting with D-erythrose-4-phosphate, phosphoenolpyruvate, or carbon dioxide in some species (147). With the evolution of organisms (apart from animals) bacteria, fungi, and plants retained the ability to synthesize tryptophan (24). Conversely, mammals only attain tryptophan, an essential amino acid, during food intake. A reduction of tryptophan leads to the marked lowering of melatonin production in animals compared to that in plants (145, 148). Since plants cannot behaviorally avoid extremely stressful conditions, they require extra protection from stress; hence, the biosynthesis of tryptophan is presumably retained in plants to ensure that melatonin is available for relieving oxidative stress levels under environmentally-stressful conditions.

Beginning with tryptophan, melatonin biosynthesis includes four enzymatic steps in all organisms (22, 67). During its evolution lasting billions of years, the pattern of the melatonin synthesis became diversified (**Figure 4**). Tryptophan is first converted to serotonin which involves decarboxylation and hydroxylation. There are two strategies for the synthesis of serotonin that leads to melatonin production in different taxa. The biosynthetic pathway of serotonin in microorganisms and plants is different from that of vertebrates. Tryptophan is decarboxylated to tryptamine by tryptophan decarboxylase (TDC), followed by serotonin biosynthesis catalyzed by tryptamine 5-hydroxylase (T5H) in plants (149, 150). In contrast, rather than tryptophan decarboxylation being the initial step in serotonin production, animals first hydroxylate tryptophan using tryptophan hydroxylase (TPH) to form 5-hydroxytryptophan and then 5-hydroxytryptophan is decarboxylated by aromatic amino acid decarboxylase (AADC) to form serotonin (7).

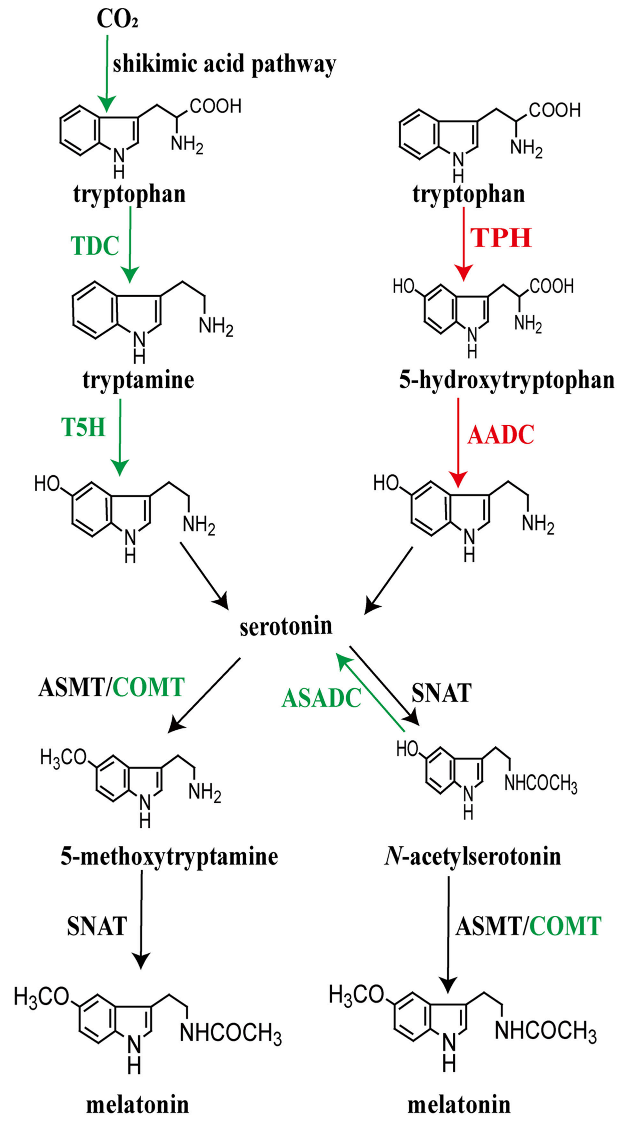


**FIGURE 4 |** Pathways of melatonin synthesis in different plant (left) and animal (right) taxa. Depending on the organism, not all of the events necessarily take place in the chloroplasts or mitochondria of every species. For the species, plant and animal, that have been investigated, the published data provide strong evidence that these organelles are critically involved with melatonin production.

Between tryptophan and melatonin, serotonin is a key intermediate after which the biosynthetic process utilizes two potential pathways, each of which includes two consecutive enzymatic steps to generate melatonin (15, 22). These steps catalyze serotonin to form the final product, melatonin; this involves serotonin *N*-acetyltransferase (NAT) and *N*-acetylserotonin *O*-methyltransferase (ASMT; formerly known as hydroxyindole-*O*-methyltransferase, HIOMT) (151–155). The penultima enzyme, NAT, plays a key role in the conversion of serotonin to *N*-acetylserotonin while the last enzyme, ASMT, catalyzes NAS to produce melatonin (153, 154, 156). Alternatively, serotonin can be first O-methylated to 5-methoxytryptamine (5-MT) by AMST; thereafter, 5-MT is N-acetylated by NAT to produce melatonin (7, 155). Differing from the formation of serotonin, the two alternative pathways for the conversion of serotonin to melatonin, likely occur in both plants and animals as well as in the microorganisms. However, different homologs of NAT have been detected between plants and animals, as well as ASMT in the two groups, revealing their different origins during evolution (122, 157–159). NAT seems to have originated independently as indicated by the few shared amino acid residues between animals and plants as well as between primitive cyanobacteria and archaea (24, 158). Thus, the enzymes controlling the biosynthetic steps of melatonin seem to have different origins at the emergence of melatonin; these enzymes further evolved divergently after endosymbiosis.

As already mentioned, the intermediate processes in melatonin production display a variety of differences among taxa. The present-day organisms possess diverse melatonin biosynthetic pathways. The enzymes that produce melatonin may play roles in catalyzing different substrates. Beyond the four key enzymes, other evolved enzymes are reported to directly participate in the biosynthesis of melatonin. This is supported by evidence that plants evolved caffeic acid O-methyltransferase (COMT), which is involved in the synthesis of 5-methoxytryptamine/melatonin by methylating serotonin/*N*-acetylserotonin (160). Apart from the classic enzymes strictly required for the melatonin biosynthetic pathways, Lee et al. (161) found that *N*-acetylserotonin can be converted to serotonin in rice seedlings by *N*-acetylserotonin deacetylase (ASDAC), which may result in a reduction in the content of melatonin (**Figure 4**).

## SUBCELLULAR LOCALIZATIONS OF ENZYMES ASSOCIATED WITH MELATONIN BIOSYNTHESIS FROM THE VIEW OF ENDOSYMBIOSIS

Based on the endosymbiotic theory, when the early eukaryotic cells (having nuclei but no mitochondria) endocytosed α-proteobacteria or cyanobacteria (**Figure 1**), rather than digesting these bacteria, the proto-eukaryotic cells developed a symbiotic association with them (162–164). The observations that the NAT protein, the rate-limiting enzyme of melatonin synthesis, is abundantly located in mitochondria of animals and chloroplasts of plants further support the different origins of the melatonin biosynthetic enzymes as a result of endosymbiosis (26, 165, 166). Furthermore, the DNA sequences and protein residues of cyanobacterium, a plant-type species, and rice are closely related, implying that the plant NAT gene was likely endosymbiotically-derived from cyanobacteria (167, 168). NAT genes of other eukaryotic organisms including fungi, invertebrates, and vertebrates seemingly evolved from *Rhodospirillum rubrum* (the presumed precursor of mitochondria) or closely related species since their NAT genes share similarity to some extent (24, 122, 169).

With endosymbiotic evolution, the function of melatonin synthesis was carried into multicellular organisms. Thus,

mitochondria and chloroplast, which resulted from the endosymbiosis of α-proteobacteria or cyanobacteria, respectively, became the major melatonin generating subcellular organelle in both animals and plants (165, 166). In terms of function, melatonin produced in these two organelles most likely detoxifies excessive ROS and reactive nitrogen species (RNS) generated during oxidative phosphorylation and other metabolic actions (55, 73). The production of melatonin in mitochondria provides maximal on-site protection of these critical organelles (**Figure 4**). In plants, melatonin in chloroplasts provides a similar defense against oxidative stress with subsequent evolution after endosymbiosis. The melatonin-associated genes of the incorporated bacteria were gradually transferred from both mitochondria and chloroplasts to the nuclear genome of each host (24, 164–166, 170). While mitochondria and chloroplasts are considered major sites of melatonin synthesis, it does not preclude the possibility that some melatonin is not also formed in the cytosol ((171); also, see below).

With subsequent evolutionary processes, melatonin-related genes were modified by mutations and in response to natural selective pressures in different species (24, 155, 172). Specifically, the major structural differences of the melatonin synthases among phylogenetically distant species are the regulatory regions which could further influence the subcellular localization of these proteins (7, 24). At least in plants, the subcellular localization analysis documented that the rate-limiting enzyme for melatonin synthesis, NAT, is found in both chloroplasts and mitochondria (26). TPH, ASMT/COMT, and TDC/AADC locate in the cytoplasm while T5H is distributed in the endoplasmic reticulum (7, 22, 150, 154). This subcellular location of melatonin synthesis enzymes suggests that during evolution (**Figure 2**), the sites of melatonin synthesis became more diverse and extended to the cytoplasm and endoplasmic reticulum (22). This divergent distribution of melatonin production shows a good relationship with the transformation from prokaryotic cell to eukaryotic cell. Regarding the efficiency of melatonin biosynthesis, the present biosynthetic model is consistent with adequate substrate availability. For example, acetyl-CoA, a key substrate for melatonin production, is synthesized in the mitochondria through pyruvate dehydrogenase complex reaction (39, 173, 174). Furthermore, the different subcellular sites of melatonin synthetase avoid substrate competition by other enzymes preferring the same substrate (13, 22, 175). Thus, multiple subcellular sites of melatonin biosynthesis in both plants and animals could promote synthetic efficiency of this essential molecule (171).

## MULTIPLE MECHANISMS PRECISELY REGULATE MELATONIN BIOSYNTHESIS

The presence of melatonin at several sites correlates with its biological functions. As in animals, 24-h rhythms have been described in plants, e.g., in *Chenopodium*, which shows a nocturnal maximum growth rate around light/dark transition (176). Also, the dinoflagellate *Lingulodinium* and numerous other microalgae including chlorophyceans (like plants, members of viridiplantae), exhibit robust circadian rhythms of melatonin (177). In comparison to animal cells, plant cells contain much higher levels of melatonin, probably because they have two melatonin producing organelles, mitochondria and chloroplasts (77, 158, 178). In some species such as in *Glycyrrhiza uralensis*, cranberry and several medicinal herbs melatonin levels are reportedly several orders of magnitude higher than those in the serum of animals. Remarkably, the levels of melatonin in the pistachio nut may reach 230 μg/g (15, 145, 179, 180). This may relate to the high environmental stress condition under which this plant normally grows. The immobility of plants results in them being subjected to more unavoidable environmental stressors, causing elevated ROS production and oxidative damage. Thus, they require additional protection from stressors by means of intrinsic mechanisms including high levels of endogenously-produced antioxidants, such as melatonin (158). This speculation is supported by the observation that a variety of environmental insults induce a dramatic increase in melatonin levels in plants (24, 141, 181). Under some conditions, stressful situations may also induce melatonin production in animals, e.g., physiological ischemia/reperfusion events (182). Also, plant cells generally have higher levels of melatonin than animal cells; this likely relates to the fact that plant cells have two sources of melatonin (mitochondria and chloroplasts) while animal cells have a single source.

The presence of melatonin in both plants and animals raises the question as to whether animals and plants have different mechanisms for modulating the biosynthesis of this indole-containing compound. Current data indicate that the regulatory mechanisms of melatonin synthesis are fundamentally different between animals and plants (**Figure 5**). In vertebrates, melatonin is referred to as the chemical expression of darkness (based only on pineal and blood levels); in general, in plants a day/night rhythm is less common, although not totally absent in some species, with the melatonin concentrations sometimes not varying much throughout the light:dark cycle (24, 183, 184). Hence, melatonin synthesis in some plants is non-rhythmic, as in the mitochondria of animals (101).

In vertebrates, light detection by the retinas suppresses the activity of NAT and melatonin production (185, 186). In plants, there is positive correlation between light intensity and melatonin levels with plants growing in habitats exposed to high light intensities, such as Mediterranean or alpine environments, usually having higher melatonin levels than the same or related species growing in other locations (187). In some species, melatonin was reported to be enhanced by darkness of even short duration, e.g., 1 h in rice seedlings (188), findings supported by an elevated ASMT expression in response to darkness (189). Moreover, heat-induced elevation of melatonin was antagonized by light in Oryza (188). In addition to these exceptions, the signal transduction pathways and regulatory mechanisms in vertebrates differ substantially from those in plants. The major regulatory components of melatonin synthesis in mammals, e.g., norepinephrine and its receptors, are not detected in plants and are unlikely to exist, indicating the loss of this pathway in the plants during evolution (24).

As noted above, the biosynthesis of melatonin is closely associated with four successive enzymes leading to the production of this compound. NAT usually is believed to

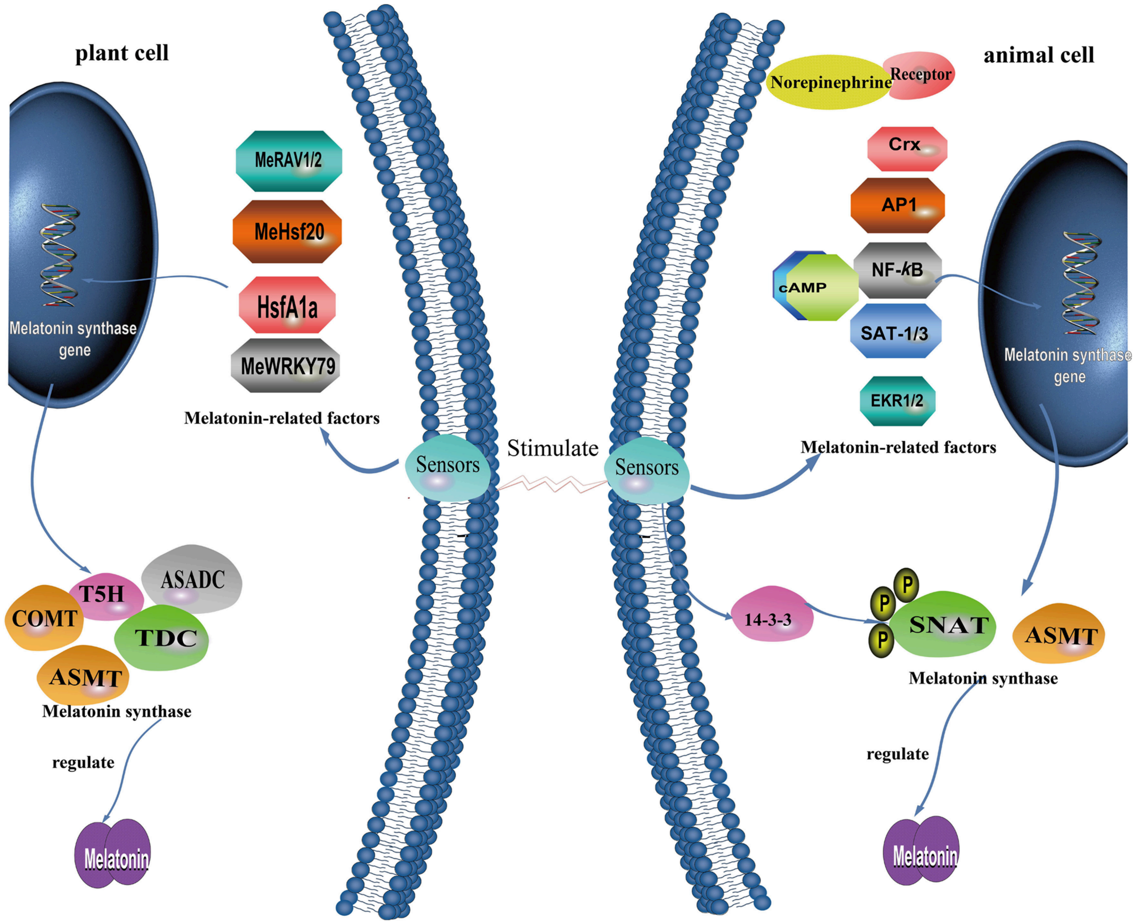


**FIGURE 5 |** A summary of what is known concerning the molecular mechanism governing melatonin production in plant cells and animal cells.

be the rate-limiting enzyme in pineal melatonin synthesis in vertebrates, although ASMT may limit this level around the nocturnal melatonin maximum (190). For plants, under most circumstances, NAT activity correlates well with the quantity of melatonin produced. In rice seedlings, however, melatonin was reported to be highest at the time of enhanced ASMT expression (191, 192). Typical for animals, the activities of NAT and melatonin production usually exhibit a good positive relationship (185, 193). The regulation of NAT in the pineal gland depends on the animal taxa examined. In many mammals, NAT gene expression in the pineal is either up- or down-regulated by the signals received from the suprachiasmatic nucleus (SCN) while in some species with a predominantly transcriptional control of NAT, its mRNA levels are stimulated up to two orders of magnitude (194). For primates and ungulates, NAT is mainly controlled post-translationally by phosphorylation and dephosphorylation, and stabilization of the phosphorylated form by a 14-3-3 protein (195). While the four successive enzymes positively regulate the pineal concentration of melatonin in all organisms, the overexpression of melatonin biosynthetic genes, such as *TDC*, *NAT*, and *ASMT* do not always lead to the accumulation of melatonin in plants (33, 122, 155, 159, 196, 197). Recently, the *ASDAC* gene was found to catalyze the conversion of *N*-acetylserotonin to serotonin, a reverse reaction from the usual melatonin biosynthetic pathway in plants (161). Clearly, plants possess two genes encoding both NAT and ASDAC proteins, which impact the biosynthesis of melatonin; NAT favors melatonin synthesis, whereas ASDAC lowers melatonin levels.

Especially in plants, melatonin is maintained at a relatively constant level under normal conditions; however, it can be greatly and rapidly upregulated in response to unfavorable conditions such as cold, heat, salt, drought, oxidative and nutrient stress, and bacterial infection (142, 198–203). The underlying mechanisms for the rapid regulation of melatonin production have not been identified including the translation and post-translational regulation of melatonin synthesis enzymes, and the upstream transcription factors of these rate-limiting enzymes or isoenzymes (204, 205). Melatonin biosynthesis genes may have a role at the transcriptional level to control the content of melatonin (**Figure 5**). For this process, different taxa evolved divergently to work with other factors for self-development or coping with stressful conditions.

Activator protein-1 (AP-1) is a stress-responsive transcription factor that can be regulated by oxidative stress in many cell types (206). AP-1 seems to promote both NAT and ASMT activities to enhance melatonin synthesis. Structural analysis of the human *ASMT* gene promoter shows that it contains an AP-1 site at position−166 and similarly, there is an AP-1 transcription factor-binding site in the *AANAT* mouse gene (29, 30). Interestingly, stress uniformly stimulates glucocorticoid production in organisms (207). Glucocorticoids upregulate the transcriptional activity AP-1 and thereby promote gene expression for melatonin synthesis (171, 208). AP-1, in addition to acting as a signal transducer and activator of transcription-1 and 3 (STAT-1; STAT-3), competes with NF-κB for binding to nat-κB1 to regulate the transcription of NAT (32). Transcription of NAT driven by NF-κB dimers mediates pathogen-associated molecular patterns (PAMPs) or pro-inflammatory cytokine-induced melatonin synthesis in macrophages by binding to one or two upstream κB binding sites (nat-κB1 and nat-κB2) of the NAT promoter in RAW 264.7 cells (209). LIM homeobox transcription factor Isl1 positively modulates melatonin synthesis by targeting NAT at the (ATTA/TAAT) motif, via the ERK signaling pathway of norepinephrine (34). Regarding the binding site of TFs, the chicken NAT gene does not contain a canonical cAMP-response element (CRE) sequence TGACGTCA (210) but a TTATT8 repeat sequence and a CLS (6/8 identical to the canonical CRE) in basal and cAMP-driven promoters which bind c-Fos, JunD, and CREB to enhance basal and forskolin-stimulated NAT transcription (211). This motif was not found in NAT genes from other species, including mouse, rat, and zebrafish (211). For the rat, a cAMP- response element (CRE) -like sequence (CLS; TGCGCCA)-CCAAT complex in the flanking region and a canonical CRE in the first intron drives cAMP-dependent induction of the NAT gene as well (212, 213). In addition, the cone-rod homeobox (Crx) transcription factor was reported to regulate the expression of NAT in the mouse pineal gland.

For plants, limited information related to transcriptional regulation is available compared with that of animals. Evidence from recent studies show that a multifunctional enzyme, namely caffeic acid O-methyltransferase (COMT), can also catalyze the last step of melatonin biosynthesis (28). In rice, melatonin biosynthesis requires ASMT or COMT activity (154). Cai et al. (33) revealed that, in tomato, cadmium stress induces the expression of HsfA1a, which acts as a positive regulator of COMT1 transcript levels by binding to the COMT1 gene promoter heat-shock element (HSE) sequence (GAANNTTC), and induces melatonin accumulation. For cassava, three TFs were found to modulate melatonin biosynthesis. Cassava bacterial blight induces the expression of MeWRKY79 and MeHsf20, which activate the expression of MeASMT2 via binding to W-box (TTGACC/T) and HSEs (GAAnnTTC) in the MeASMT2 promoter; this, in turn, increases melatonin accumulation and confers improved disease resistance (205). MeRAV1 and MeRAV2 may directly regulate three melatonin biosynthesis genes (*MeTDC2*, *MeT5H*, and *MeASMT*) by binding their promoter containing CAACA motif as transcriptional activators, and thus up-regulate melatonin biosynthesis in response to disease resistance against cassava bacterial blight (214).

## CONCLUSION REMARKS

The acquisition of additional functions by melatonin, which is believed to have originally evolved to provide molecular protection from free radicals, occurred over a very long evolutionary period. It is theorized that melatonin first appeared in bacteria about 3.0–2.5 billion years ago. When these melatonin-synthesizing bacteria were phagocytized by early eukaryotes as food, over time they established a symbiotic association with their hosts and developed into mitochondria and chloroplasts. Since the bacteria that were ingested had the ability to synthesize melatonin, this important function was retained by the mitochondria and chloroplasts. As a consequence, we hypothesize that these organelles have produced melatonin in every plant and animal species that has ever existed and that this occurs in present day animal and plant cells as well. Thus, every cell that possesses mitochondria (animals and plants) or chloroplasts (plants), we feel, has the capacity to produce melatonin. Melatonin at these sites is important to provide protection against free radicals which are abundantly generated in these organelles. Over its very long evolutionary history, melatonin has acquired other essential functions that have been retained by this physiologically-diverse molecule.

Tryptophan is the starting molecule for melatonin production in cell species. The sequence of the enzymatic steps that convert tryptophan to melatonin, however, varies among species. These steps include hydroxylation, decarboxylation, acetylation, and methylation. In some plant species, melatonin may not be the end product; in at least one variety of rice, melatonin can be hydroxylated at either 2, 4, or 6 position with 2-hydroxymelatonin possessing significant antioxidant activity, like melatonin itself. While the synthetic pathway of melatonin has changed throughout evolution and differs among plant and animal species, the structure of melatonin persists as originally designed in bacteria billions of years ago. It is pointed out, however, that what is known about melatonin synthesis has come primarily from mammals and the pathway in other vertebrates has been sparingly investigated. Moreover, the pathway of melatonin production in invertebrates remains to be examined.

## AUTHOR CONTRIBUTIONS

RR initiated the review and checked all drafts of the report. DZ, YY, and YS worked together to write the initial drafts of the manuscript. QL and RS prepared figures for the article and read the final version. ZZ and RS participated in the discussion of the functional evolution of melatonin.

## FUNDING

This research was funded by the National Natural Science Foundation of China (81560622).

## REFERENCES


1. Lerner AB, Case JD, Takahashi Y, Lee TH, Mori W. Isolation of melatonin, the pineal gland factor that lightens melanocyteS[1]. *J Am Chem Soc.* (1958) 80:2587. doi: 10.1021/ja01543a060
2. Dubbels R, Reiter R, Klenke E, Goebel A, Schnakenberg E, Ehlers C, et al. Melatonin in edible plants identified by radioimmunoassay and by high performance liquid chromatography-mass spectrometry. *J Pineal Res.* (1995) 18:28–31. doi: 10.1111/j.1600-079X.1995.tb00136.x
3. Hattori A, Migitaka H, Iigo M, Itoh M, Yamamoto K, Ohtani-Kaneko R, et al. Identification of melatonin in plants and its effects on plasma melatonin levels and binding to melatonin receptors in vertebrates. *Biochem Mol Biol Int.* (1995) 35:627–34.
4. Manchester LC, Tan DX, Reiter RJ, Qi W, Karbownik M, Calvo JR. High levels of melatonin in edible seeds: possible function in germ cell protection. *Life Sci.* (2000) 67:3023–9. doi: 10.1016/S0024-3205(00)00896-1
5. Reiter RJ, Tan DX, Galano A. Melatonin: exceeding expectations. *Physiology.* (2014) 29:325–33. doi: 10.1152/physiol.00011.2014
6. Shi H, Chen K, Wei Y, He C. Fundamental issues of melatonin-mediated stress signaling in plants. *Front Plant Sci.* (2016) 7:1124. doi: 10.3389/fpls.2016.01124
7. Tan DX, Hardeland R, Back K, Manchester LC, Alatorre-Jimenez MA, Reiter RJ. On the significance of an alternate pathway of melatonin synthesis via 5-methoxytryptamine: comparisons across species. *J Pineal Res.* (2016) 61:27–40. doi: 10.1111/jpi.12336
8. Manchester LC, Coto-Montes A, Boga JA, Andersen LPH, Zhou Z, Galano A, et al. Melatonin: an ancient molecule that makes oxygen metabolically tolerable. *J Pineal Res.* (2015) 59:403–19. doi: 10.1111/jpi.12267
9. Pshenichnyuk SA, Modelli A, Jones D, Lazneva EF, Komolov AS. Low-energy electron interaction with melatonin and related compounds. *J Phys Chem B.* (2017) 121:3965–74. doi: 10.1021/acs.jpcb.7b01408
10. Reiter RJ, Tan DX, Rosales-Corral S, Galano A, Jou MJ, Acuna-Castroviejo D. Melatonin mitigates mitochondrial meltdown: interactions with SIRT3. *Int J Mol Sci.* (2018) 19:E2439. doi: 10.3390/ijms19082439
11. Margulis L. Symbiotic theory of the origin of eukaryotic organelles; criteria for proof. *Symp Soc Exp Biol.* (1975) 29:21–38.
12. Tan DX, Manchester LC, Liu X, Rosales-Corral SA, Acuna-Castroviejo D, Reiter RJ. Mitochondria and chloroplasts as the original sites of melatonin synthesis: a hypothesis related to melatonin's primary function and evolution in eukaryotes. *J Pineal Res.* (2013) 54:127–38. doi: 10.1111/jpi.12026
13. Reiter RJ, Rosales-Corral S, Tan DX, Jou MJ, Galano A, Xu B. Melatonin as a mitochondria-targeted antioxidant: one of evolution's best ideas. *Cell Mol Life Sci.* (2017) 74:3863–81. doi: 10.1007/s00018-017-2609-7
14. Tan DX, Chen LD, Poeggeler B, Manchester LC, Reiter RJ. Melatonin: a potent, endogenous hydroxyl radical scavenger. *Endocr J.* (1993) 1:57–60.
15. Tan DX, Manchester LC, Esteban-Zubero E, Zhou Z, Reiter R. Melatonin as a potent and inducible endogenous antioxidant: synthesis and metabolism. *Molecules.* (2015) 20:18886. doi: 10.3390/molecules201018886
16. Galano A, Reiter RJ. Melatonin and its metabolites vs. oxidative stress: from individual actions to collective protection. *J Pineal Res.* (2018) 65:e12514. doi: 10.1111/jpi.12514
17. Galano A, Tan DX, Reiter RJ. Melatonin: a versatile protector against oxidative DNA damage. *Molecules.* (2018) 23:E530. doi: 10.3390/molecules23030530
18. Tan DX, Hardeland R, Manchester LC, Paredes SD, Korkmaz A, Sainz RM, et al. The changing biological roles of melatonin during evolution: from an antioxidant to signals of darkness, sexual selection and fitness. *Biol Rev.* (2010) 85:607–23. doi: 10.1111/j.1469-185X.2009.00118.x
19. Lochner A, Marais E, Huisamen B. Melatonin and cardioprotection against ischaemia/reperfusion injury: what's new? A review. *J Pineal Res.* (2018) 65:e12490. doi: 10.1111/jpi.12490
20. Onaolapo AY, Onaolapo OJ. Circadian dysrhythmia-linked diabetes mellitus: examining melatonin's roles in prophylaxis and management. *World J Diabetes*. (2018) 9:99–114. doi: 10.4239/wjd.v9.i7.99
21. Tamtaji OR, Mobini M, Reiter RJ, Azami A, Gholami MS, Asemi Z. Melatonin, a toll-like receptor inhibitor: current status and future perspectives. *J Cell Physiol.* (2018) 2018:27698. doi: 10.1002/jcp.27698
22. Back K, Tan DX, Reiter RJ. Melatonin biosynthesis in plants: multiple pathways catalyze tryptophan to melatonin in the cytoplasm or chloroplasts. *J Pineal Res.* (2016) 61:426–37. doi: 10.1111/jpi.12364
23. Ye T, Yin X, Yu L, Zheng SJ, Cai WJ, Wu Y, et al. Metabolic analysis of the melatonin biosynthesis pathway using chemical labeling coupled with liquid chromatography-mass spectrometry. *J Pineal Res.* 2018: e12531. doi: 10.1111/jpi.12531
24. Tan DX, Zheng X, Kong J, Manchester L, Hardeland R, Kim S, et al. Fundamental Issues related to the origin of melatonin and melatonin isomers during evolution: relation to their biological functions. *Int J Mol Sci.* (2014) 15:15858. doi: 10.3390/ijms150915858
25. Lee K, Choi GH, Back K. Cadmium-induced melatonin synthesis in rice requires light, hydrogen peroxide, and nitric oxide: key regulatory roles for tryptophan decarboxylase and caffeic acid *O*-methyltransferase. *J Pineal Res.* (2017) 63:e12441. doi: 10.1111/jpi.12441
26. Byeon Y, Lee HY, Lee K, Park S, Back K. Cellular localization and kinetics of the rice melatonin biosynthetic enzymes SNAT and ASMT. *J Pineal Res.* (2014) 56:107–14. doi: 10.1111/jpi.12103
27. Byeon Y, Lee HY, Hwang OJ, Lee HJ, Lee K, Back K. Coordinated regulation of melatonin synthesis and degradation genes in rice leaves in response to cadmium treatment. *J Pineal Res.* (2015) 58:470–8. doi: 10.1111/jpi.12232
28. Lee HY, Byeon Y, Lee K, Lee HJ, Back K. Cloning of *Arabidopsis* serotonin N-acetyltransferase and its role with caffeic acid *O*-methyltransferase in the biosynthesis of melatonin *in vitro* despite their different subcellular localizations. *J Pineal Res.* (2014) 57:418–26. doi: 10.1111/jpi.12181
29. Rodriguez IR, Mazuruk K, Schoen TJ, Chader GJ. Structural analysis of the human hydroxyindole-*O*-methyltransferase gene. Presence of two distinct promoters. *J Biol Chem.* (1994) 269:31969–77.
30. Estrada-Rodgers L, Levy GN, Weber WW. Characterization of a hormone response element in the mouse *N*-Acetyltransferase 2 (Nat2*) promoter. *Gene Expr.* (1998) 7:13–24.
31. Korkmaz A, Reiter RJ, Topal T, Manchester LC, Oter S, Tan DX. Melatonin: an established antioxidant worthy of use in clinical trials. *Mol Med.* (2009) 15:43–50. doi: 10.2119/molmed.2008.00117
32. Muxel SM, Laranjeira-Silva MF, Carvalho-Sousa CE, Floeter-Winter LM, Markus RP. The RelA/cRel nuclear factor-κB (NF-κB) dimer, crucial for inflammation resolution, mediates the transcription of the key enzyme in melatonin synthesis in RAW 264.7 macrophages. *J Pineal Res.* (2016) 60:394–404. doi: 10.1111/jpi.12321
33. Cai SY, Zhang Y, Xu YP, Qi ZY, Li MQ, Ahammed GJ, et al. HsfA1a upregulates melatonin biosynthesis to confer cadmium tolerance in tomato plants. *J Pineal Res.* (2017) 62:e12387. doi: 10.1111/jpi.12387
34. Zhang J, Qiu J, Zhou Y, Wang Y, Li H, Zhang T, et al. LIM homeobox transcription factor Isl1 is required for melatonin synthesis in the pig pineal gland. *J Pineal Res.* (2018) 65:e12481. doi: 10.1111/jpi.12481
35. Luo G, Ono S, Beukes NJ, Wang DT, Xie S, Summons RE. Rapid oxygenation of earth's atmosphere 2.33 billion years ago. *Sci Adv.* (2016) 2:e1600134. doi: 10.1126/sciadv.1600134
36. Izon G, Zerkle AL, Williford KH, Farquhar J, Poulton SW, Claire MW. Biological regulation of atmospheric chemistry en route to planetary oxygenation. *Proc Natl Acad Sci USA.* (2017) 114:201618798. doi: 10.1073/pnas.1618798114
37. Taverne YJ, Merkus D, Bogers AJ, Halliwell B, Duncker DJ, Lyons TW. Reactive oxygen species: radical factors in the evolution of animal life: a molecular timescale from earth's earliest history to the rise of complex life. *Bioessays.* (2018) 40:1700158. doi: 10.1002/bies.201700158
38. Kump LR, Barley ME. Increased subaerial volcanism and the rise of atmospheric oxygen 2.5 billion years ago. *Nature.* (2007) 448:1033–6. doi: 10.1038/nature06058
39. Reiter RJ, Rosales-Corral S, Zhou X, Tan DX. Role of SIRT3/SOD2 signaling in mediating the antioxidant actions of melatonin in mitochondria. *Curr Trends Endocrinol.* (2017) 9:45–9.
40. Donaghy L, Hong HK, Jauzein C, Choi KS. The known and unknown sources of reactive oxygen and nitrogen species in haemocytes of marine bivalve molluscs. *Fish Shellfish Immunol.* (2015) 42:91–7. doi: 10.1016/j.fsi.2014.10.030

41. Dharmaraja AT. Role of reactive oxygen species (ROS) in therapeutics and drug resistance in cancer and bacteria. *J Med Chem.* (2017) 60:3221–40. doi: 10.1021/acs.jmedchem.6b01243
42. Casteilla L, Rigoulet M, Pénicaud L. Mitochondrial ROS metabolism: modulation by uncoupling proteins. *IUBMB Life.* (2001) 52:181–8. doi: 10.1080/15216540152845984
43. Treberg JR, Braun K, Zacharias P, Kroeker K. Multidimensional mitochondrial energetics: applications to the study of electron leak and hydrogen peroxide metabolism. *Comp Biochem Physiol B Biochem Mol Biol.* (2018) 224:121–8. doi: 10.1016/j.cbpb.2017.12.013
44. Case A. On the origin of superoxide dismutase: an evolutionary perspective of superoxide-mediated redox signaling. *Antioxidants.* (2017) 6:82. doi: 10.3390/antiox6040082
45. Manchester LC, Poeggeler B, Alvares FL, Ogden GB, Reiter RJ. Melatonin immunoreactivity in the photosynthetic prokaryote *Rhodospirillum rubrum*: implications for an ancient antioxidant system. *Chem Mol Biol Res.* (1995) 41:391–5.
46. Mayo JC, Sainz RM, González Menéndez P, Cepas V, Tan DX, Reiter RJ. Melatonin and sirtuins: a "not-so unexpected" relationship. *J Pineal Res.* (2017) 62:e12391. doi: 10.1111/jpi.12391
47. Acuña-Castroviejo D, Martín M, Macías M, Escames G, León J, Khaldy H, et al. Melatonin, mitochondria, and cellular bioenergetics. *J Pineal Res.* (2001) 30:65–74. doi: 10.1034/j.1600-079X.2001.300201.x
48. Leon J, Acuna-Castroviejo D, Escames G, Tan DX, Reiter RJ. Melatonin mitigates mitochondrial malfunction. *J Pineal Res.* (2005) 38:1–9. doi: 10.1111/j.1600-079X.2004.00181.x
49. Hardeland R. Melatonin and retinoid orphan receptors: demand for new interpretation after their exclusion as nuclear melatonin receptors. *Melatonin Res.* (2018) 65:e12525. doi: 10.32794/mr11250005
50. Hardeland R, Tan DX, Reiter RJ. Kynuramines, metabolites of melatonin and other indoles: the resurrection of an almost forgotten class of biogenic animals. *J Pineal Res.* (2009) 47:109–26. doi: 10.1111/j.1600-079X.2009.00701.x
51. Galano A, Tan DX, Reiter RJ. On the free radical scavenging activities of melatonin's metabolites, AFMK and AMK. *J Pineal Res.* (2013) 54:245–57. doi: 10.1111/jpi.12010
52. Jou MJ, Peng TI, Reiter RJ. Protective stabilization of mitochondrial permeability transition and mitochondrial oxidation during mitochondrial $Ca^{2+}$ stress by melatonin's cascade metabolites C3-OHM and AFMK in RBA1 astrocytes. *J Pineal Res.* (2018) 2018:e12538. doi: 10.1111/jpi.12538
53. Hevia D, Mayo JC, Tan DX, Rodriguez-Garcia A, Sainz RM. Melatonin enhances photo-oxidation of 2′, 7′-dichlorodihydrofluorescein by an antioxidant reaction that renders *N*1-acetyl-*N*2-formyl-5-methoxykynuramine (AFMK). *PloS ONE.* (2014) 9:e109257. doi: 10.1371/journal.pone.0109257
54. Vielma JR, Bonilla E, Chacin-Bonilla L, Mora M, Medina-Leendertz S, Bravo Y. Effects of melatonin on oxidative stress, and resistance to bacterial, parasitic, and viral infections: a review. *Acta Trop.* (2014) 137:31–8. doi: 10.1016/j.actatropica.2014.04.021
55. Reiter RJ, Mayo JC, Tan DX, Sainz RM, Alatorre-Jimenez M, Qin L. Melatonin as an antioxidant: under promises but over delivers. *J Pineal Gland.* (2016) 61:253–78. doi: 10.1111/jpi.12360
56. Hardeland R. Taxon- and site-specific melatonin catabolism. *Molecules.* (2017) 22:E2015. doi: 10.3390/molecules22112015
57. Lee K, Zawadzka A, Czarnocki Z, Reiter RJ, Back K. Molecular cloning of melatonin 3-hydroxylase and its production of cyclic 3-hydroxymelatonin in rice (*Oryza sativa*). *J Pineal Res.* (2016) 61:470–8. doi: 10.1111/jpi.12361
58. Vass I. Role of charge recombination processes in photodamage and photoprotection of the photosystem II complex. *Physiol Plant.* (2011) 142:6–16. doi: 10.1111/j.1399-3054.2011.01454.x
59. Vass I. Molecular mechanisms of photodamage in the Photosystem II complex. *Biochim Biophys Acta.* (2012) 1817:209–17. doi: 10.1016/j.bbabio.2011.04.014
60. Hardeland R, Fuhrberg B, Uria H, Behrmann G, Meyer TJ, Burkhardt S, et al. Chronobiology of indoleamines in the dinoflagellate Gonyaulax polyedra: metabolism and effects related to circadian rhythmicity and photoperiodism. *Braz J Med Biol Rep.* (1996) 29:119–23.
61. Latifi A, Ruiz M, Zhang CC. Oxidative stress in cyanobacteria. *FEMS Microbiol Rev.* (2009) 33:258–78. doi: 10.1111/j.1574-6976.2008.00134.x
62. Claustrat B, Brun J, Chazot G. The basic physiology and pathophysiology of melatonin. *Sleep Med Rev.* (2005) 9:11–24. doi: 10.1016/j.smrv.2004.08.001
63. Venegas C, García JA, Escames G, Ortiz F, López A, Doerrier C, et al. Extrapineal melatonin: analysis of its subcellular distribution and daily fluctuations. *J Pineal Res.* (2012) 52:217–27. doi: 10.1111/j.1600-079X.2011.00931.x
64. Roth JJ, Gern WA, Roth EL, Ralph CL, Jacobson E. Nonpineal melatonin in the alligator (*Alligator mississippiensis*). *Science.* (1980) 210:548–50. doi: 10.1126/science.7423204
65. Oksche A. The development of the concept of photoneuroendocrine systems: historical perspective. In: *Klein DC, Moor RY, Reppert SM.* editors. *Suprachiasmatic Nucleus.* New York, NY: Oxford University Press (1991). pp. 5–11.
66. Kolář J, Johnson CH, Macháčková I. Presence and possible role of melatonin in a short-day flowering plant, *Chenopodium rubrum*. *Adv Exp Med Biol.* (1999) 460:391–3. doi: 10.1007/0-306-46814-X_46
67. Hardeland R. Melatonin, hormone of darkness and more - occurrence, control mechanisms, actions and bioactive metabolites. *Cellular and Molecular Life Sci.* (2008) 65:2001–18. doi: 10.1007/s00018-008-8001-x
68. Johnson CH. Precise circadian clocks in prokaryotic cyanobacteria. *Curr Issues Mol Biol.* (2004) 6:103–10.
69. Mori T, Mchaourab H, Johnson CH. Circadian clocks: unexpected biochemical cogs. *Curr Biol.* (2015) 25:R842–4. doi: 10.1016/j.cub.2015.08.026
70. Tan DX, Manchester LC, Di Mascio R, Martinez GR, Prado FM, Reiter RJ. Novel rhythms of *N*1-acetyl-*N*2-formyl-5-methoxykynuramine and its precursor melatonin in the water hyacinth: importance in phytoremediation. *FASEB J.* (2007) 21:1724–9. doi: 10.1096/fj.06-7745com
71. Poeggeler B. Melatonin, aging, and age-related diseases. *Endocrine.* (2005) 27:201–12. doi: 10.1385/ENDO:27:2:201
72. Jou MJ, Peng TI, Yu PZ, Jou SB, Reiter RJ, Chen JY, et al. Melatonin protects against common deletion of mitochondrial DNA-augmented mitochondrial oxidative stress and apoptosis. *J Pineal Res.* (2007) 43:389–403. doi: 10.1111/j.1600-079X.2007.00490.x
73. Majidinia M, Reiter RJ, Shakouri SK, Yousefi B. The role of melatonin, a multitasking molecule, in retarding the processes of ageing. *Ageing Res Rev.* (2018) 47:198–213. doi: 10.1016/j.arr.2018.07.010
74. Nabavi SM, Nabavi SF, Sureda A, Xiao J, Dehpour AR, Shirooie S, et al. Anti-inflammatory effects of Melatonin: a mechanistic review. *Crit Rev Food Sci Nutr.* (2018) 14:1–13. doi: 10.1080/10408398.2018.1487927
75. Pevet P, Klosen P, Felder-Schmittbuhl MP. The hormone melatonin: animal studies. *Best Pract Res Clin Endocrinol Metab.* (2017) 31:547–59. doi: 10.1016/j.beem.2017.10.010
76. Simonneaux V. Naughty melatonin: how mothers tick off their fetus. *Endocrinology.* (2011) 152:1734–8. doi: 10.1210/en.2011-0226
77. Reiter RJ, Tan DX, Sharma R. Historical perspective and evaluation of the mechanisms by which melatonin mediates seasonal reproduction in mammals. *Melaton Res.* (2018) 1:58–76. doi: 10.32794/mr11250004
78. Carrillo-Vico A, Reiter RJ, Lardone PJ, Herrera JL, Fernández-Montesinos R, Guerrero JM, et al. The modulatory role of melatonin on immune responsiveness. *Curr Opin Investig Drugs.* (2006) 7:423–31.
79. Claustrat B, Leston J. Melatonin: physiological effects in humans. *Neurochirurgie.* (2015) 61:77–84. doi: 10.1016/j.neuchi.2015.03.002
80. Kriegsfeld LJ, Ubuka T, Bentley GE, Tsutsui K. Seasonal control of gonadotropin-inhibitory hormone (GnIH) in birds and mammals. *Front Neuroendocrinol.* (2015) 37:65–75. doi: 10.1016/j.yfrne.2014.12.001
81. Mendivil-Perez M, Soto-Mercado V, Guerra-Librero A, Fernandez-Gil BI, Florido J, Shen YQ, et al. Melatonin enhances neural stem cell differentiation and engraftment by increasing mitochondrial function. *J Pineal Res.* (2017) 63:e12415. doi: 10.1111/jpi.12415
82. Najafi M, Shirazi A, Motevaseli E, Rezaeyan AH, Salajegheh A, Rezapoor S. Melatonin as an anti-inflammatory agent in radiotherapy. *Inflammopharmacology.* (2017) 25:403–13. doi: 10.1007/s10787-017-0332-5
83. Bondy S, Campbell A. Mechanisms underlying tumor suppressive properties of melatonin. *Int J Mol Sci.* (2018) 19:E2205. doi: 10.3390/ijms19082205

84. Hill SM, Belancio VP, Dauchy RT, Xiang S, Brimer S, Mao L, et al. Melatonin: an inhibitor of breast cancer. *Endocr Relat Cancer.* (2015) 22:R183–204. doi: 10.1530/ERC-15-0030
85. Gonzalez-Gonzalez A, Mediavilla MD, Sanchez-Barcelo EJ. Melatonin: a molecule for reducing breast cancer risk. *Molecules.* (2018) 23:E336. doi: 10.3390/molecules23020336
86. Bizzarri M, Proietti S, Cucina A, Reiter RJ. Molecular mechanisms of the pro-apoptotic actions of melatonin in cancer: a review. *Exp Opin Ther Targets.* (2013) 17:1483–96. doi: 10.1517/14728222.2013.834890
87. Leja-Szpak A, Jaworek J, Pierzchalski P, Reiter RJ. Melatonin induces pro-apoptotic signaling pathway in human pancreatic carcinoma cells (PANC-1). *J Pineal Res.* (2010) 49:248–55. doi: 10.1111/j.1600-079X.2010.00789.x
88. Quintana C, Cabrera J, Perdomo J, Estévez F, Loro JF, Reiter RJ, et al. Melatonin enhances hyperthermia-induced apoptotic cell death in human leukemia cells. *J Pineal Res.* (2016) 61:381–95. doi: 10.1111/jpi.12356
89. Su SC, Hsieh MJ, Yang WE, Chung WH, Reiter RJ, Yang S. Cancer metastasis: mechanisms of inhibition by melatonin. *J Pineal Res.* (2017) 62:12370. doi: 10.1111/jpi.12370
90. Reiter RJ. The mammalian pineal gland: structure and function. *Am J Anat.* (1981) 162:287–313. doi: 10.1002/aja.1001620402
91. Dodt E, Meissl H. The pineal and parietal organs of lower vertebrates. *Experientia.* (1982) 36:996–1000. doi: 10.1007/BF01955342
92. Kramm CM, de Gip WJ, Korf HW. Rod-opsin immunoreaction in the pineal organ of the pigmented mouse does not indicate the presence of a functional photopigment. *Cell Tissue Res.* (1993) 274:71–8. doi: 10.1007/BF00327987
93. Moore RY. Neural control of the pineal gland. *Behav Brain Res.* (1996) 73:125–30. doi: 10.1016/0166-4328(96)00083-6
94. Cardinali DP, Vacas MI. Feedback control of pineal function by reproductive hormones – a neuroendocrine paradigm. *J Neural Trans Suppl.* (1978) 175–201.
95. Reiter RJ, Richardson BA. Some perturbations that disturb the circadian melatonin rhythm. *Chronobiol Int.* (1992) 9:314–21. doi: 10.3109/07420529209064541
96. Reiter RJ, Hester RJ. Interrelationships of the pineal gland, the superior ganglia and the photoperiod in the regulation of the endocrine systems of hamsters. *Endocrinology.* (1966) 79:1168–70. doi: 10.1210/endo-79-6-1168
97. Klein DC, Berg GR. Pineal gland: stimulation of melatonin production by norepinephrine involves cyclic AMP-mediated stimulation of N-acetyltransferase. *Adv Biochem Psychopharmacol.* (1970) 31:241–63.
98. Markus RP, Fernandes PA, Kinker GS, da Silveira Cruz-Machado S, Marcola M. Immune-pineal axis - acute inflammatory responses coordinate melatonin synthesis by pinealocytes and phagocytes. *Br J Pharmacol.* (2017) 175:3239–50. doi: 10.1111/bph.14083
99. Markus R, Cecon E, Pires-Lapa M. Immune-pineal axis: nuclear factor κB (NF-kB) mediates the shift in the melatonin source from pinealocytes to immune competent cells. *Int J Mol Sci.* (2013) 14:10979–97. doi: 10.3390/ijms140610979
100. Tan DX, Manchester LC, Hardeland R, Lopez-Burillo S, Mayo JC, Sainz RM, et al. Melatonin: a hormone, a tissue factor, an autocoid, a paracoid, and an antioxidant vitamin. *J Pineal Res.* (2003) 34:75–8. doi: 10.1034/j.1600-079X.2003.02111.x
101. Suofu V, Li W, Jean-Alphonse FG, Jia J, Khattar NK, Li J, et al. Dual role of mitochondria in producing melatonin and driving GPCR signaling to block cytochrome c release. *Proc Natl Acad Sci USA.* (2017) 114:E7997–8006. doi: 10.1073/pnas.1705768114
102. Acuna-Castroviejo D, Noguiera-Navarro MT, Reiter RJ, Escames G. Melatonin actions in the heart: more than a hormone. *Melaton Res.* (2018) 1:21–6. doi: 10.32794/mr11250002
103. Huo X, Wang C, Yu Z, Wang S, Peng S, Zhang S, et al. Human transporters, PEPT1/2, facilitate melatonin transportation into mitochondria of cancer cells: an implication of the therapeutic potential. *J Pineal Res.* (2017) 62:12390. doi: 10.1111/jpi.12390
104. Tosches MA, Bucher D, Vopalensky P, Arendt D. Melatonin signaling controls circadian swimming behavior in marine zooplankton. *Cell.* (2014) 159:46–57. doi: 10.1016/j.cell.2014.07.042
105. Arnao MB, Hernández-Ruiz J. Functions of melatonin in plants: a review. *J Pineal Res.* (2015) 59:133–50. doi: 10.1111/jpi.12253
106. Hu W, Yang H, Tie W, Yan Y, Ding Z, Liu Y, et al. Natural variation in banana varieties highlights the role of melatonin in postharvest ripening and quality. *J Agric Food Chem.* (2017) 65:9987–94. doi: 10.1021/acs.jafc.7b03354
107. Liang C, Li A, Yu H, Li W, Liang C, Guo S, et al. Melatonin regulates root architecture by modulating auxin response in rice. *Front Plant Sci.* (2017) 8:134. doi: 10.3389/fpls.2017.00134
108. Zhang N, Zhang HJ, Sun QQ, Cao YY, Li X, Zhao B, et al. Proteomic analysis reveals a role of melatonin in promoting cucumber seed germination under high salinity by regulating energy production. *Sci Rep.* (2017) 7:503. doi: 10.1038/s41598-017-00566-1
109. Reiter JR, Tan DX, Zhou Z, Cruz HM, Fuentes-Broto L, Galano A. Phytomelatonin: assisting plants to survive and thrive. *Molecules.* (2015) 20:7396–437. doi: 10.3390/molecules20047396
110. Reiter RJ, Tan DX, Manchester LC, Pilar TM, Terron PM, Koppisepi S. Medical implications of melatonin: receptor-mediated and receptor-independent actions. *Adv Med Sci.* (2007) 52:11–28.
111. Slominski RM, Reiter RJ, Schlabritz-Loutsevitch N, Ostrom RS, Slominski AT. Melatonin membrane receptors in peripheral tissues: Distribution and functions. *Mol Cell Endocrinol.* (2012) 351:152–66. doi: 10.1016/j.mce.2012.01.004
112. Jockers R, Maurice P, Boutin JA, Delagrange P. Melatonin receptors, heterodimerization, signal transduction and binding sites: what's new? *Br J Pharmacol.* (2008) 154:1182–95. doi: 10.1038/bjp.2008.184
113. Jockers R, Delagrange P, Dubocovich ML, Markus RP, Renault N, Tosini G, et al. Update on melatonin receptors: IUPHAR Review 20. *Br J Pharmacol.* (2016) 173:2702–25. doi: 10.1111/bph.13536
114. Dubocovich ML, Delagrange P, Krause DN, Sugden D, Cardinali DP, Olcese J. International Union of Basic and Clinical Pharmacology. LXXV Nomenclature, classification, and pharmacology of G protein-coupled melatonin receptors. *Pharmacol Rev.* (2010) 62:343–80. doi: 10.1124/pr.110.002832
115. Alkozi HA, Sánchez JM, Doadrio AL, Pintor J. Docking studies for melatonin receptors. *Expert Opin Drug Discov.* (2017) 13:241–8. doi: 10.1080/17460441.2018.1419184
116. Wei Y, Chang Y, Zeng H, Liu G, He C, Shi H. RAV transcription factors are essential for disease resistance against cassava bacterial blight via activation of melatonin biosynthesis genes. *J Pineal Res.* (2018) 64:e12454. doi: 10.1111/jpi.12454
117. Lee HY, Back K. Melatonin is required for $H_2O_2$- and NO-mediated defense signaling through MAPKKK3 and OXI1 in *Arabidopsis thaliana*. *J Pineal Res.* (2017) 62:e12379. doi: 10.1111/jpi.12379
118. Yu Y, Lv Y, Shi Y, Li T, Chen Y, Zhao D, et al. The role of phyto-melatonin and related metabolites in response to stress. *Molecules.* (2018) 23:1887. doi: 10.3390/molecules23081887
119. Boutin JA, Saunier C, Guenin SP, Berger S, Moulharat N, Gohier A, et al. Studies of the melatonin binding site location onto quinone reductase 2 by directed mutagenesis. *Arch Biochem Biophys.* (2008) 477:12–9. doi: 10.1016/j.abb.2008.04.040
120. Cassagnes LE, Chhour M, Pério P, Sudor J, Gayon R, Ferry G, et al. Oxidative stress and neurodegeneration: the possible contribution of quinone reductase 2. *Free Radic Biol Med.* (2018) 120:56–61. doi: 10.1016/j.freeradbiomed.2018.03.002
121. Reybier K, Perio P, Ferry G, Bouajila J, Delagrange P, Boutin JA, et al. Insights into the redox cycle of human quinone reductase 2. *Free Radic Res.* (2011) 45:1184–95. doi: 10.3109/10715762.2011.605788
122. Choi GH, Lee HY, Back K. Chloroplast overexpression of rice caffeic acid *O*-methyltransferase increases melatonin production in chloroplasts via the 5-methoxytryptamine pathway in transgenic rice plants. *J Pineal Res.* (2017) 63:e12412. doi: 10.1111/jpi.12412
123. Wu YH, Ursinus J, Zhou JN, Scheer FA, Ai-Min B, Jockers R, et al. Alterations of melatonin receptors MT1 and MT2 in the hypothalamic suprachiasmatic nucleus during depression. *J Affect Disord.* (2013) 148:357–67. doi: 10.1016/j.jad.2012.12.025
124. Benleulmi-Chaachoua A, Chen L, Sokolina K, Wong V, Jurisica I, Emerit MB, et al. Protein interactome mining defines melatonin MT1 receptors as integral component of presynaptic protein complexes of neurons. *J Pineal Res.* (2016) 60:95–108. doi: 10.1111/jpi.12294

125. Hardeland R. Melatonin: signaling mechanisms of a pleiotropic agent. *Biofactors.* (2009) 35:183–92. doi: 10.1002/biof.23
126. Cutando A, Aneiros-Fernandez J, Lopez-Valverde A, Arias-Santiago S, Aneiros-Cachaza J, Reiter RJ. A new perspective in oral health: potential importance and actions of melatonin receptors MT1, MT2, MT3, and RZR/ROR in the oral cavity. *Arch Oral Biol.* (2011) 56:944–50. doi: 10.1016/j.archoralbio.2011.03.004
127. Liu J, Clough SJ, Hutchinson AJ, Adamah-Biassi EB, Popovska-Gorevski M, Dubocovich ML. MT1 and MT2 melatonin receptors: a therapeutic perspective. *Ann Rev Pharmacol Toxicol.* (2016) 56:361–83. doi: 10.1146/annurev-pharmtox-010814-124742
128. Liu J, Clough SJ, Dubocovich ML. Role of the MT1 and MT2 melatonin receptors in mediating depressive- and anxiety-like behaviors in C3H/HeN mice. *Genes Brain Behav.* (2017) 16:546–53. doi: 10.1111/gbb.12369
129. Oishi A, Cecon E, Jockers R. Melatonin receptor signaling: impact of receptor oligomerization on receptor function. *Int Rev Cell Mol Biol.* (2018) 338:59–77. doi: 10.1016/bs.ircmb.2018.02.002
130. Boutin JA, Ferry G. Is there sufficient evidence that the melatonin binding Site MT3 is Quinone Reductase 2? *J Pharmacol Exp Ther.* (2019) 368:59–65. doi: 10.1124/jpet.118.253260
131. Hill SM, Frasch T, Xiang S, Yuan L, Duplessis T, Mao L. Molecular mechanisms of melatonin anticancer effects. *Integr Cancer Ther.* (2009) 8:337–46. doi: 10.1177/1534735409353332
132. Wang RX, Liu H, Xu L, Zhang H, Zhou RX. Involvement of nuclear receptor RZR/RORgamma in melatonin-induced HIF-1alpha inactivation in SGC-7901 human gastric cancer cells. *Oncol Rep.* (2015) 34:2541–6. doi: 10.3892/or.2015.4238
133. Zhao Y, Xu L, Ding S, Lin N, Ji Q, Gao L, et al. Novel protective role of the circadian nuclear receptor retinoic acid-related orphan receptor-alpha in diabetic cardiomyopathy. *J Pineal Res.* (2017) 62:e12378. doi: 10.1111/jpi.12378
134. Boutin JA. Quinone reductase 2 as a promising target of melatonin therapeutic actions. *Expert Opin Ther Targets.* (2016) 20:303–17. doi: 10.1517/14728222.2016.1091882
135. Benitez-King G, Huerto-Delgadillo L, Anton-Tay F. Binding of 3H-melatonin to calmodulin. *Life Sci.* (1993) 53:201–7. doi: 10.1016/0024-3205(93)90670-X
136. Menendez-Menendez J, Martinez-Campa C. Melatonin: an anti-tumor agent in hormone-dependent cancers. *Int J Endocrinol.* (2018) 2018:3271948. doi: 10.1155/2018/3271948
137. Mendoza-Vargas L, Solis-Chagoyan H, Benitez-King G, Fuentes-Pardo B. MT2-like melatonin receptor modulates amplitude receptor potential in visual cells of crayfish during a 24-hour cycle. *Comp Biochem Physiol A.* (2009) 154:486–92. doi: 10.1016/j.cbpa.2009.07.025
138. Maciel FE, Geihs MA, Cruz BP, Vargas MA, Allodi S, Marins LF, et al. Melatonin as a signaling molecule for metabolism regulation in response to hypoxia in the crab *Neohelice granulata*. *Int J Mol Sci.* (2014) 15:22405–20. doi: 10.3390/ijms151222405
139. Li G, Zhang Y, Ni Y, Wang Y, Xu B, Guo X. Identification of a melatonin receptor type 1A gene (AccMTNR1A) in Apis cerana cerana and its possible involvement in the response to low temperature stress. *Naturwissenschaften.* (2018) 105:24. doi: 10.1007/s00114-018-1546-0
140. Csaba G. Biogenic amines at a low level of evolution: production, functions and regulation in the unicellular Tetrahymena. *Acta Microbiol Immunol Hungar.* (2015) 62:93–108. doi: 10.1556/030.62.2015.2.1
141. Arnao MB, Hernández-Ruiz J. Growth conditions influence the melatonin content of tomato plants. *Food Chem.* (2013) 138:1212–4. doi: 10.1016/j.foodchem.2012.10.077
142. Shi H, Jiang C, Ye T, Tan DX, Reiter RJ, Zhang H, et al. Comparative physiological, metabolomic, and transcriptomic analyses reveal mechanisms of improved abiotic stress resistance in bermudagrass [Cynodon dactylon (L). Pers] by exogenous melatonin. *J Exp Bot.* (2015) 66:681–94. doi: 10.1093/jxb/eru373
143. Tosini G, Owino S, Guillaume JL, Jockers R. Understanding melatonin receptor pharmacology: latest insights from mouse models, and their relevance to human disease. *Bioessays.* (2014) 36:778–87. doi: 10.1002/bies.201400017
144. Reiter RJ, Tan DX, Rosales-Corral S, Manchester LC. The universal nature, unequal distribution and antioxidant functions of melatonin and its derivatives. *Mini Rev Med Chem.* 13:373–84. doi: 10.2174/1389557113804999810
145. Hardeland R. Melatonin in plants - diversity of levels and multiplicity of functions. *Front Plant Sci.* (2016) 7:198. doi: 10.3389/fpls.2016.00198
146. Hardeland R. Melatonin in plants and other phototrophs: advances and gaps concerning the diversity of functions. *J Exp Bot.* (2015) 66:627–46. doi: 10.1093/jxb/eru386
147. Bochkov DV, Sysolyatin SV, Kalashnikov AI, Surmacheva IA. Shikimic acid: review of its analytical, isolation, and purification techniques from plant and microbial sources. *J Chem Biol.* (2012) 5:5–17. doi: 10.1007/s12154-011-0064-8
148. Fuhrberg B, Hardeland R, Poeggeler B, Behrmann C. Dramatic rises of melatonin and 5-methoxytryptamine in Gonyaulax exposed to decreased temperature. *J Interdiscip Cycle Res.* (1997) 28:144–50. doi: 10.1076/brhm.28.1.144.12978
149. De Luca V, Marineau C, Brisson N. Molecular cloning and analysis of cDNA encoding a plant tryptophan decarboxylase: comparison with animal dopa decarboxylases. *Proc Natl Acad Sci USA.* (1989) 86:2582–6. doi: 10.1073/pnas.86.8.2582
150. Park M, Kang K, Park S, Back K. Conversion of 5-hydroxytryptophan into serotonin by tryptophan decarboxylase in plants, *Escherichia coli*, and yeast. *Biosci Biotech Bioch.* (2008) 72:2456–8. doi: 10.1271/bbb.80220
151. Axelrod J, Weissbach H. Enzymatic *O*-methylation of *N*-acetylserotonin to melatonin. *Science.* (1960) 131:1312. doi: 10.1126/science.131.3409.1312
152. Weissbach H, Redfield BG, Axelrod J. Biosynthesis of melatonin: enzymic conversion of serotonin to *N*-acetylserotonin. *Biochim Biophys Acta.* (1960) 43:352–3. doi: 10.1016/0006-3002(60)90453-4
153. Kang K, Lee K, Park S, Byeon Y, Back K. Molecular cloning of rice serotonin *N*-acetyltransferase, the penultimate gene in plant melatonin biosynthesis. *J Pineal Res.* (2013) 55:7–13. doi: 10.1111/jpi.12011
154. Byeon Y, Choi GH, Lee HY, Back K. Melatonin biosynthesis requires *N*-acetylserotonin methyltransferase activity of caffeic acid *O*-methyltransferase in rice. *J Exp Bot.* (2015) 66:6917–25. doi: 10.1093/jxb/erv396
155. Byeon Y, Lee HJ, Lee HY, Back K. Cloning and functional characterization of the *Arabidopsis N*-acetylserotonin *O*-methyltransferase responsible for melatonin synthesis. *J Pineal Res.* (2016) 60:65–73. doi: 10.1111/jpi.12289
156. Klein DC. Arylalkylamine *N*-acetyltransferase: "the timezyme". *J Biol Chem.* (2007) 282:4233–7. doi: 10.1074/jbc.R600036200
157. Kang K, Kong K, Park S, Natsagdorj U, Kim YS, Back K. Molecular cloning of a plant *N*-acetylserotonin methyltransferase and its expression characteristics in rice. *J Pineal Res.* (2011) 50:304–9. doi: 10.1111/j.1600-079X.2010.00841.x
158. Tan DX, Hardeland R, Manchester LC, Korkmaz A, Ma S, Rosales-Corral S, et al. Functional roles of melatonin in plants, and perspectives in nutritional and agricultural science. *J Exp Bot.* (2012) 63:577–97. doi: 10.1093/jxb/err256
159. Lee K, Back K. Overexpression of rice serotonin *N*-acetyltransferase 1 in transgenic rice plants confers resistance to cadmium and senescence and increases grain yield. *J Pineal Res.* (2017) 62:e12392. doi: 10.1111/jpi.12392
160. Byeon Y, Lee HY, Lee K, Back K. Caffeic acid *O*-methyltransferase is involved in the synthesis of melatonin by methylating *N*-acetylserotonin in *Arabidopsis*. *J Pineal Res.* (2014) 57:219–27. doi: 10.1111/jpi.12160
161. Lee K, Lee HY, Back K. Rice histone deacetylase 10 and *Arabidopsis* histone deacetylase 14 genes encode *N*-acetylserotonin deacetylase, which catalyzes conversion of *N*-acetylserotonin into serotonin, a reverse reaction for melatonin biosynthesis in plants. *J Pineal Res.* (2017) 64:e12460. doi: 10.1111/jpi.12460
162. Esser C, Ahmadinejad N, Wiegand C, Rotte C, Sebastiani F, Gelius-Dietrich G, et al. A genome phylogeny for mitochondria among α-Proteobacteria and a predominantly eubacterial ancestry of yeast nuclear genes. *Mol Biol Evol.* (2004) 21:1643–60. doi: 10.1093/molbev/msh160
163. Archibald JM. Endosymbiosis and eukaryotic cell evolution. *Curr Biol.* (2015) 25:R911–21. doi: 10.1016/j.cub.2015.07.055
164. Gentil J, Hempel F, Moog D, Zauner S, Maier U. Origin of complex algae by secondary endosymbiosis: a journey through time. *Protoplasma.* (2017) 254:1835–43. doi: 10.1007/s00709-017-1098-8

165. Wang L, Feng C, Zheng X, Guo Y, Zhou F, Shan D, et al. Plant mitochondria synthesize melatonin and enhance the tolerance of plants to drought stress. *J Pineal Res.* (2017) 63:e12429. doi: 10.1111/jpi.12429
166. Zheng X, Tan DX, Allan AC, Zuo B, Zhao Y, Reiter RJ, et al. Chloroplastic biosynthesis of melatonin and its involvement in protection of plants from salt stress. *Sci Rep.* (2017) 7:41236. doi: 10.1038/srep41236
167. Byeon Y, Lee K, Park YI, Park S, Back K. Molecular cloning and functional analysis of serotonin *N*-acetyltransferase from the cyanobacterium *Synechocystis* sp. PCC 6803. *J Pineal Res.* (2013) 55:371–6. doi: 10.1111/jpi.12080
168. Byeon Y, Yool Lee H, Choi DW, Back K. Chloroplast-encoded serotonin *N*-acetyltransferase in the red alga *Pyropia yezoensis*: gene transition to the nucleus from chloroplasts. *J Exp Bot.* (2015) 66:709–17. doi: 10.1093/jxb/eru357
169. Coon SL, Klein DC. Evolution of arylalkylamine *N*-acetyltransferase: emergence and divergence. *Mol Cell Endocrinol.* (2006) 252:2–10. doi: 10.1016/j.mce.2006.03.039
170. Kurland CG, Andersson SGE. Origin and evolution of the mitochondrial proteome. *Microbiol Mol Biol Rev.* (2000) 64:786–820. doi: 10.1128/MMBR.64.4.786-820.2000
171. Tan DX, Reiter RJ. Mitochondria: the birth place, battle ground and site of melatonin metabolism in cells. *Melatonin Res.* (2019) 2:44–66. doi: 10.32794/nr11250011
172. Falcón J, Coon SL, Besseau L, Cazaméa-Catalan D, Fuentès M, Magnanou E, et al. Drastic neofunctionalization associated with evolution of the timezyme AANAT 500 Mya. *Proc Natl Acad Sci USA*. (2014) 111:314–9. doi: 10.1073/pnas.1312634110
173. Shi L, Tu BP. Acetyl-CoA and the regulation of metabolism: mechanisms and consequences. *Curr Opin Cell Biol.* (2015) 33:125–31. doi: 10.1016/j.ceb.2015.02.003
174. Mehta MM, Weinberg SE, Chandel NS. Mitochondrial control of immunity: beyond ATP. *Nat Rev Immunol.* (2017) 17:608. doi: 10.1038/nri.2017.66
175. Park S, Byeon Y, Kim YS, Back K. Kinetic analysis of purified recombinant rice N-acetylserotonin methyltransferase and peak melatonin production in etiolated rice shoots. *J Pineal Res.* (2013) 54:139–44. doi: 10.1111/j.1600-079X.2012.01019.x
176. Lecharny A, Wagner E. Stem extension rate in light-grown plants. Evidence for an endogenous circadian rhythm in *Chenopodium rubrum. Physiol Plant.* (1984) 60:437–43. doi: 10.1111/j.1399-3054.1984.tb06089.x
177. Kolář J, Macháčková I. Melatonin in higher plants: occurrence and possible functions. *J Pineal Res.* (2005) 39:333–41. doi: 10.1111/j.1600-079X.2005.00276.x
178. Reiter RJ, Tan DX, Burkhardt S, Manchester LC. Melatonin in plants. *Nutr Rev.* (2001) 59:286–90. doi: 10.1111/j.1753-4887.2001.tb07018.x
179. Murch SJ, Simmons CB, Saxena PK. Melatonin in feverfew and other medicinal plants. *Lancet.* (1997) 350:1598–9. doi: 10.1016/S0140-6736(05)64014-7
180. Brown PN, Turi CE, Shipley PR, Murch SJ. Comparisons of large (*Vaccinium macrocarpon* Ait.) and small (*Vaccinium oxycoccos* L., *Vaccinium vitis-idaea* L.) cranberry in British Columbia by phytochemical determination, antioxidant potential, and metabolomic profiling with chemometric analysis. *Planta Med.* (2012) 78:630–40. doi: 10.1055/s-0031-1298239
181. Arnao MB, Hernández-Ruiz J. Growth conditions determine different melatonin levels in Lupinus albus L. *J Pineal Res.* (2013) 55:149–55. doi: 10.1111/jpi.12055
182. Tan DX, Manchester LC, Sainz RM, Mayo JC, Leon J, Reiter RJ. Physiological ischemia/reperfusion phenomena and their relation to endogenous melatonin production: a hypothesis. *Endocrine.* (2005) 27:149–58. doi: 10.1385/ENDO:27:2:149
183. Murch SJ, KrishnaRaj S, Saxena PK. Tryptophan is a precursor for melatonin and serotonin biosynthesis in *in vitro* regenerated St. *John's wort (Hypericum perforatum L cv Anthos)* plants. *Plant Cell Rep.* (2000) 19:698–704. doi: 10.1007/s002990000206
184. Reiter RJ. Melatonin: the chemical expression of darkness. *Mol Cell Endocrinol.* (1991) 79:C153–8.
185. Ebihara S, Adachi A, Hasegawa M, Nogi T, Yoshimura T, Hirunagi K. *In vivo* microdialysis studies of pineal and ocular melatonin rhythms in birds. *Neurosignals.* (1997) 6:233–40. doi: 10.1159/000109133
186. Kim TD, Woo KC, Cho S, Ha DC, Jang SK, Kim KT. Rhythmic control of AANAT translation by hnRNP Q in circadian melatonin production. *Genes Dev.* (2007) 21:797–810. doi: 10.1101/gad.1519507
187. Hardeland R, Pandi-Perumal S, Poeggeler B. Melatonin in plants - focus on a vertebrate night hormone with cytoprotective properties. *Funct Plant Sci Biotechnol.* (2007) 1:32–45.
188. Byeon Y, Back K. Melatonin synthesis in rice seedlings *in vivo* is enhanced at high temperatures and under dark conditions due to increased serotonin *N*-acetyltransferase and *N*-acetylserotonin methyltransferase activities. *J Pineal Res.* (2014) 56:189–95. doi: 10.1111/jpi.12111
189. Park S, Lee DE, Jang H, Byeon Y, Kim YS, Back K. Melatonin-rich transgenic rice plants exhibit resistance to herbicide-induced oxidative stress. *J Pineal Res.* (2013) 54:258–63. doi: 10.1111/j.1600-079X.2012.01029.x
190. Liu T, Borjigin J. *N*-acetyltransferase is not the rate-limiting enzyme of melatonin synthesis at night. *J Pineal Res.* (2005) 39:91–6. doi: 10.1111/j.1600-079X.2005.00223.x
191. Zuo B, Zheng X, He P, Wang L, Lei Q, Feng C, et al. Overexpression of MzASMT improves melatonin production and enhances drought tolerance in transgenic *Arabidopsis thaliana* plants. *J Pineal Res.* (2014) 57:408–17. doi: 10.1111/jpi.12180
192. Byeon Y, Back K. Low melatonin production by suppression of either serotonin *N*-acetyltransferase or *N*-acetylserotonin methyltransferase in rice causes seedling growth retardation with yield penalty, abiotic stress susceptibility, and enhanced coleoptile growth under anoxic conditions. *J Pineal Res.* (2016) 60:348–59. doi: 10.1111/jpi.12317
193. Klein DC. Evolution of the vertebrate pineal gland: the AANAT hypothesis. *Chronobiol Int.* (2006) 23:5–20. doi: 10.1080/07420520500545839
194. Schomerus C, Korf HW. Mechanisms regulating melatonin synthesis in the mammalian pineal organ. *Ann N Y Acad Sci.* (2005) 1057:372–83. doi: 10.1196/annals.1356.028
195. Pozdeyev N, Taylor C, Haque R, Chaurasia SS, Visser A, Thazyeen A, et al. Photic regulation of arylalkylamine N-acetyltransferase binding to 14-3-3 proteins in retinal photoreceptor cells. *J Neurosci.* (2006) 26:9153–61. doi: 10.1523/jneurosci.1384-06.2006
196. Byeon Y, Park S, Lee HY, Kim YS, Back K. Elevated production of melatonin in transgenic rice seeds expressing rice tryptophan decarboxylase. *J Pineal Res.* (2014) 56:275–82. doi: 10.1111/jpi.12120
197. Li MQ, Hasan MK, Li CX, Ahammed GJ, Xia XJ, Shi K, et al. Melatonin mediates selenium-induced tolerance to cadmium stress in tomato plants. *J Pineal Res.* (2016) 61:291–302. doi: 10.1111/jpi.12346
198. Shi H, Chen Y, Tan DX, Reiter RJ, Chan Z, He C. Melatonin induces nitric oxide and the potential mechanisms relate to innate immunity against bacterial pathogen infection in *Arabidopsis*. *J Pineal Res.* (2015) 59:102–8. doi: 10.1111/jpi.12244
199. Shi H, Qian Y, Tan DX, Reiter RJ, He C. Melatonin induces the transcripts of CBF/DREB1s and their involvement in both abiotic and biotic stresses in *Arabidopsis*. *J Pineal Res.* (2015) 59:334–42. doi: 10.1111/jpi.12262
200. Shi H, Reiter RJ, Tan DX, Chan Z. *INDOLE-3-ACETIC ACID INDUCIBLE 17* positively modulates natural leaf senescence through melatonin-mediated pathway in *Arabidopsis*. *J Pineal Res.* (2015) 58:26–33. doi: 10.1111/jpi.12188
201. Shi H, Tan DX, Reiter RJ, Ye T, Yang F, Chan Z. Melatonin induces class A1 heat-shock factors (HSFA1s) and their possible involvement of thermotolerance in *Arabidopsis*. *J Pineal Res.* (2015) 58:335–42. doi: 10.1111/jpi.12219
202. Shi H, Wang X, Tan DX, Reiter RJ, Chan Z. Comparative physiological and proteomic analyses reveal the actions of melatonin in the reduction of oxidative stress in Bermuda grass (*Cynodon dactylon* (L). Pers). *J Pineal Res.* (2015) 59:120–31. doi: 10.1111/jpi.12246
203. Zhang N, Sun Q, Zhang H, Cao Y, Weeda S, Ren S, et al. Roles of melatonin in abiotic stress resistance in plants. *J Exp Bot.* (2015) 66:647–56. doi: 10.1093/jxb/eru336
204. Ganguly S, Weller JL, Ho A, Chemineau P, Malpaux B, Klein DC. Melatonin synthesis: 14-3-3-dependent activation and inhibition of arylalkylamine *N*-acetyltransferase mediated by phosphoserine-205. *Proc Natl Acad Sci USA.* (2005) 102:1222–7. doi: 10.1073/pnas.0406871102
205. Wei Y, Liu G, Bai Y, Xia F, He C, Shi H. Two transcriptional activators of *N*-acetylserotonin *O*-methyltransferase 2 and melatonin biosynthesis in cassava. *J Exp Bot.* (2017) 68:4997–5006. doi: 10.1093/jxb/erx305

206. Scortegagna M, Galdzicki Z, Rapoport SI, Hanbauer I. Activator protein-1 DNA binding activation by hydrogen peroxide in neuronal and astrocytic primary cultures of trisomy-16 and diploid mice. *Mol Brain Res.* (1999) 73:144–50. doi: 10.1016/S0169-328X(99)00257-0
207. Seematter G, Binnert C, Martin JL, Tappy L. Relationship between stress, inflammation and metabolism. *Curr Opin Clin Nutr Metab Care.* (2004) 7:169–73. doi: 10.1097/00075197-200403000-00011
208. da Silveira Cruz-Machado S, Tamura EK, Carvalho-Sousa CE, Rocha VA, Pinato L, Fernandes PAC, et al. Daily corticosterone rhythm modulates pineal function through NFkappaB-related gene transcriptional program. *Sci Rep.* (2017) 7:2091. doi: 10.1038/s41598-017-02286-y
209. Muxel SM, Pires-Lapa MA, Monteiro AWA, Cecon E, Tamura EK, Floeter-Winter LM, et al. NF-κB drives the synthesis of melatonin in RAW 264.7 macrophages by inducing the transcription of the arylalkylamine-*N*-acetyltransferase (AA-NAT) gene. *PloS ONE.* (2012) 7:e52010. doi: 10.1371/journal.pone.0052010
210. Chong NW, Bernard M, Klein DC. Characterization of the chicken serotonin *N*-acetyltransferase gene: activation via clock gene heterodimer/E-box interaction. *J Biol Chem.* (2000) 275:32991–8. doi: 10.1074/jbc.M005671200
211. Haque R, Chong NW, Ali F, Chaurasia SS, Sengupta T, Chun E, et al. Melatonin synthesis in retina: cAMP-dependent transcriptional regulation of chicken arylalkylamine *N*-acetyltransferase by a CRE-like sequence and a TTATT repeat motif in the proximal promoter. *J Neurochem.* (2011) 119:6–17. doi: 10.1111/j.1471-4159.2011.07397.x
212. Baler R, Covington S, Klein DC. Rat arylalkylamine *N*-acetyltransferase gene: upstream and intronic components of a bipartite promoter. *Biol Cell.* (1999) 91:699–705. doi: 10.1111/j.1768-322X.1999.tb01114.x
213. Burke Z, Wells T, Carter D, Klein D, Baler R. Genetic targeting: the serotonin *N*-acetyltransferase promoter imparts circadian expression selectively in the pineal gland and retina of transgenic rats. *J Neurochem.* (1999) 73:1343–9.doi: 10.1046/j.1471-4159.1999.0731343.x
214. Wei J, Li DX, Zhang JR, Shan C, Rengel Z, Song ZB, et al. Phytomelatonin receptor PMTR1-mediated signaling regulates stomatal closure in *Arabidopsis thaliana*. *J Pineal Res.* (2018) 65:e12500. doi: 10.1111/jpi.12500

**Conflict of Interest Statement:** The authors declare that the research was conducted in the absence of any commercial or financial relationships that could be construed as a potential conflict of interest.

*Copyright © 2019 Zhao, Yu, Shen, Liu, Zhao, Sharma and Reiter. This is an open-access article distributed under the terms of the Creative Commons Attribution License (CC BY). The use, distribution or reproduction in other forums is permitted, provided the original author(s) and the copyright owner(s) are credited and that the original publication in this journal is cited, in accordance with accepted academic practice. No use, distribution or reproduction is permitted which does not comply with these terms.*
