# Supplementary material for: 5-Hydroxytryptophan (5-HTP): Natural Occurrence, Analysis, Biosynthesis, Biotechnology, Physiology and Toxicology
Source: Int J Mol Sci. 2020 Dec 26;22(1):181. doi: 10.3390/ijms22010181 (PMC7796270; doi:10.3390/ijms22010181)
Supplement: Supplementary file 1 [file ijms-22-00181-s001.zip › 5-HTP.Data/PDF/1545623328/annurev.biochem.68.1.355.pdf]

Annu. Rev. Biochem. 1999. 68:355–381
Copyright © 1999 by Annual Reviews. All rights reserved.

Annu. Rev. Biochem. 1999.68:355-381. Downloaded from www.annualreviews.org
Access provided by Universita Degli Studi di Torino - All Libraries on 11/16/20. For personal use only.

TETRAHYDROPTERIN-DEPENDENT AMINO ACID HYDROXYLASES

Paul F. Fitzpatrick
Department of Biochemistry and Biophysics and Department of Chemistry, Texas A&M University, College Station, Texas 77843-2128; e-mail: fitzpat@tamu.edu

Key Words tyrosine, phenylalanine, tryptophan, mechanism, structure

■ **Abstract** Phenylalanine hydroxylase, tyrosine hydroxylase, and tryptophan hydroxylase constitute a small family of monooxygenases that utilize tetrahydropterins as substrates. When from eukaryotic sources, these enzymes are composed of a homologous catalytic domain to which are attached discrete N-terminal regulatory domains and short C-terminal tetramerization domains, whereas the bacterial enzymes lack the N-terminal and C-terminal domains. Each enzyme contains a single ferrous iron atom bound to two histidines and a glutamate. Recent mechanistic studies have begun to provide insights into the mechanisms of oxygen activation and hydroxylation. Although the hydroxylating intermediate in these enzymes has not been identified, the iron is likely to be involved. Reversible phosphorylation of serine residues in the regulatory domains affects the activities of all three enzymes. In addition, phenylalanine hydroxylase is allosterically regulated by its substrates, phenylalanine and tetrahydrobiopterin.

CONTENTS

Introduction 356
Structure 357
Primary Structures 357
Three-Dimensional Structures 359
Mechanism 362
Metal Requirement 362
Steady State Kinetics 362
Substrate Specificity 363
Rate-Determining Step 366
Chemical Mechanism 366
Regulatory Properties 370
Phenylalanine Hydroxylase 371
Tyrosine Hydroxylase 373
Tryptophan Hydroxylase 376
Perspectives 377

0066-4154/99/0701-0355$08.00

Annu. Rev. Biochem. 1999.68:355-381. Downloaded from www.annualreviews.org
Access provided by Universita Degli Studi di Torino - All Libraries on 11/16/20. For personal use only.

INTRODUCTION

Hydroxylation of an aromatic ring is a fundamental reaction in biology. It occurs frequently in the activation of compounds during catabolism and in the biosynthesis of natural products. Not surprisingly, organisms have developed several methods of catalyzing such a reaction. Among the most common are the cytochrome P-450–dependent hydroxylases and the flavoprotein phenol hydroxylases. Because of the ubiquity of these two families of enzymes, their mechanisms and structures have been the subjects of extensive studies. These investigations have been greatly aided by the absorbency properties of the tightly bound heme and flavin cofactors in these enzymes and by relevant chemical studies of model compounds. In contrast, the small group of enzymes that utilize a tetrahydropterin substrate to catalyze the hydroxylation of an aromatic ring has seen significantly less study, owing to a combination of the difficulty of obtaining the proteins and the lack of a convenient chromophore. Still, the last few years have seen a significant increase in our understanding of the mechanistic and regulatory properties of this family of enzymes.

The reactions carried out by the three members of this group are illustrated in Figure 1. Phenylalanine hydroxylase catalyzes the formation of tyrosine; mutations in this enzyme are the most common cause of phenylketonuria. Tyrosine hydroxylase catalyzes the formation of dihydroxyphenylalanine, the first step in the biosynthesis of the catecholamine neurotransmitters. Tryptophan hydroxylase catalyzes the formation of 5-hydroxytryptophan, the first step in the biosynthesis of the neurotransmitter serotonin. These enzymes are monooxygenases, incorporating one atom of oxygen from molecular oxygen into the substrate and reducing the other atom to water. The two electrons required for the reduction of the second atom to water are supplied by the tetrahydrobiopterin (BH_4) substrate. It should be emphasized that, in contrast to hemes and flavins, tetrahydropterins act as substrates rather than tightly bound cofactors, binding and dissociating each turnover.

Phenylalanine hydroxylase is present at relatively abundant levels in liver. This enzyme has also been isolated from at least two bacterial strains (1, 2). Both tyrosine and tryptophan hydroxylase are found in the central nervous system. Tyrosine hydroxylase is also present in the adrenal gland, a common source of the naturally occurring enzyme. There are reports of a bacterial tryptophan hydroxylase (3), but this has not been characterized.

As shown in Figure 1, the pterin product of the reactions catalyzed by these enzymes is a hydroxypterin. This was first established with phenylalanine hydroxylase (4), but more recently has been shown to be the case with the other two enzymes (5–7). With phenylalanine hydroxylase the stereochemistry of the hydroxypterin has been established as *S* at the hydroxyl (8). The hydroxypterin rapidly dehydrates in solution (9); the resulting quinonoid dihydropterin can be reduced to the tetrahydropterin by the pyridine-nucleotide-dependent enzyme dihydropteridine reductase (10).

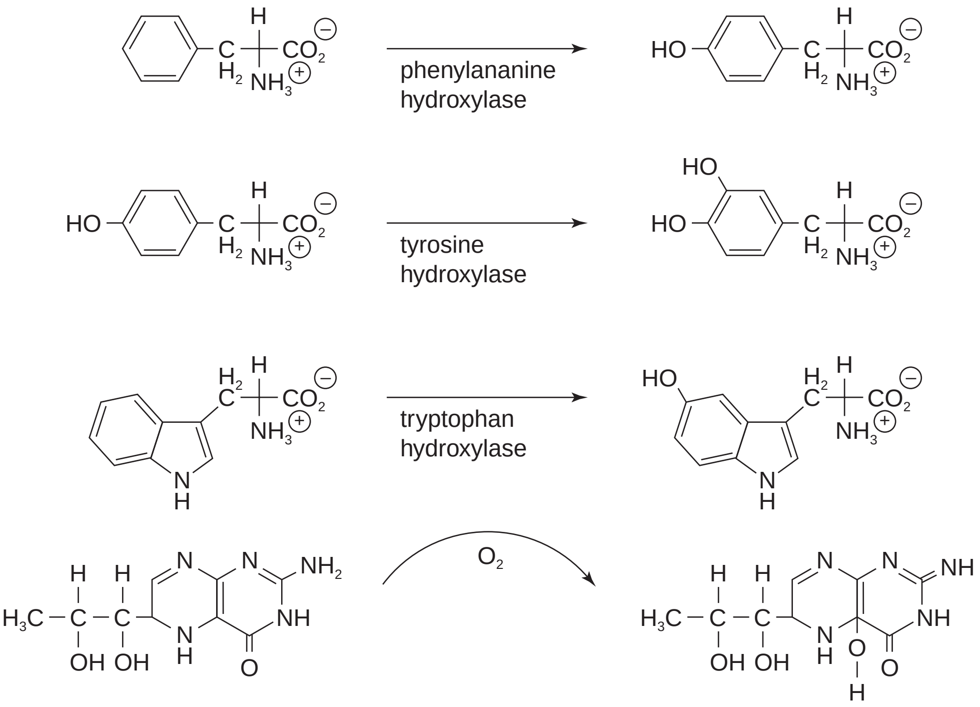

Figure 1 The reactions catalyzed by the tetrahydropterin-dependent hydroxylases.

Annu. Rev. Biochem. 1999.68:355-381. Downloaded from www.annualreviews.org
Access provided by Universita Degli Studi di Torino - All Libraries on 11/16/20. For personal use only.

STRUCTURE

Primary Structures

Comparison of the amino acid sequences of the enzymes from a variety of eukaryotic sources readily establishes the structural similarities of the three hydroxylases. There are differences in lengths: rat phenylalanine hydroxylase has 453 amino acid residues if the N-terminal methionine is counted, rat tyrosine hydroxylase has 498 residues, and rat tryptophan hydroxylase has 444 residues. There are also some species-specific differences in size. All three enzymes are composed of an N-terminal region, the length and sequence of which is specific for the specific enzyme, and a highly conserved C-terminal region (Figure 2). The conserved C-terminal region begins at a valine 118 of rat phenylalanine hydroxylase, 164 of rat tyrosine hydroxylase, and 105 of rat tryptophan hydroxylase. The C-terminal 334–339 amino acid residues of the eukaryotic enzymes are highly conserved such that these regions can be aligned with no gaps. The two bacterial phenylalanine hydroxylases that have been described lack the N-terminal region; that from *Pseudomonas aeruginosa* contains 264 residues, beginning at residue 157 of the rat sequence (11), whereas that from

Chromobacterium violaceum contains 280 residues, beginning at residue 144 (12). Alignment of the bacterial enzymes with the conserved C-terminal regions of the eukaryotic enzymes also requires that several gaps be introduced into the bacterial sequences (11). In addition, the bacterial enzymes lack 25–30 residues found at the C termini of the eukaryotic enzymes. There are no similarities between the C-terminal 38 amino acid residues of the bacterial phenylalanine hydroxylases and the eukaryotic enzymes (11, 13).

Alternative splicing of mRNA can generate four different forms of human tyrosine hydroxylase; all of the differences are in the N-terminus (14–16). The differences in the sequences of these proteins are shown in Figure 3. Isoform hTH1 corresponds to the rat enzyme. The other three isoforms contain insertions between methionine 30 and serine 31 of hTH1. As discussed below, serine 31 is phosphorylated as part of the regulation of tyrosine hydroxylase; changes in the amino acid sequence immediately preceding this residue would be expected to affect its recognition by specific protein kinases (14).

The sequence similarities were detected after the first few members of this family had been sequenced, and these similarities led to the proposal that the C-terminal portions of all three enzymes were homologous, arising from a common ancestor, and represented the catalytic core (17). The amino terminal regions were proposed to be responsible for substrate specificity (18). Studies of trun-

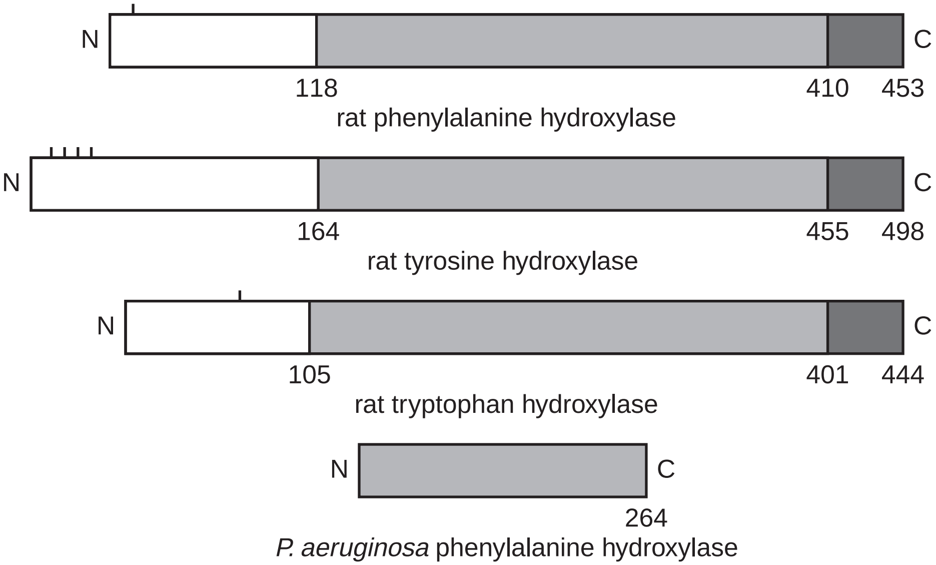

Figure 2 Comparison of the domain structures of the tetrahydropterin-dependent hydroxylases. The functional domains defined using truncated proteins are indicated: the regulatory domains are *white*, the catalytic domains *shaded*, and the tetramerization domains *hatched*. The phosphorylation sites in the regulatory domains are indicated by *vertical* lines.

Annu. Rev. Biochem. 1999.68:355-381. Downloaded from www.annualreviews.org
Access provided by Universita Degli Studi di Torino - All Libraries on 11/16/20. For personal use only.

hTH1-4	MPTPDATTPQAKGFRRAV**S**ELDAKQAEAIM	30
hTH1	-------------------------------**S**PRFIGRRQ**S**	40
hTH2	VRGQ---------------------------**S**PRFIGRRQ**S**	44
hTH3	----GAPGPSLTGSPTPGTAAPAASYTPTPR**S**PRFIGRRQ**S**	67
hTH4	VRGQGAPGPSLTGSPTPGTAAPAASYTPTPR**S**PRFIGRRQ**S**	71

Figure 3 Sequences of the human tyrosine hydroxylase isoforms. The *first line* indicates the common N-terminal sequence; the *next four lines* show the inserts. The remainder of the sequences are identical. *Hyphens* indicate deletions. The phosphorylated serine residues are indicated in *bold*.

Annu. Rev. Biochem. 1999.68:355-381. Downloaded from www.annualreviews.org
Access provided by Universita Degli Studi di Torino - All Libraries on 11/16/20. For personal use only.

cated eukaryotic enzymes have supported the concept of a common catalytic core. Phenylalanine hydroxylase lacking 141 N-terminal residues or 43 C-terminal residues retains catalytic activity (19), tryptophan hydroxylase lacking as many as 106 N-terminal residues (20) and as many as 28 C-terminal residues (7) retains activity, and tyrosine hydroxylase lacking 157 N-terminal residues or 43 C-terminal residues retains catalytic activity (21). The correspondence between the sequences of the bacterial phenylalanine hydroxylases and the smallest active versions of the eukaryotic enzymes further supports identification of a common C-terminal catalytic core.

Three-Dimensional Structures

The eukaryotic hydroxylases are all homotetramers (22–24), although phenylalanine hydroxylase readily forms dimers (22, 25). Deletion of residues from the N-termini does not alter the quaternary structures (26–28), whereas further removal of 20–43 residues from the C-termini converts the enzymes to monomers (7, 19, 29, 30). Bacterial phenylalanine hydroxylase, which lacks both regions, is monomeric (1, 2). Although the C-terminal 20 amino acid residues are not conserved in the eukaryotic enzymes, they all contain hydrophobic residues at the first and fourth positions of each seven-residue sequence. Based on these results, the C-terminus of each subunit has been proposed to form a helix; packing of the helices from four subunits would be responsible for the tetrameric structure of these enzymes (30, 31).

The three-dimensional structures have been determined for human phenylalanine hydroxylase lacking the first 102 and the last 25 residues and for rat tyrosine hydroxylase lacking the first 155 residues (32, 33). Neither structure contains bound substrates or inhibitors. The two structures are essentially identical, with a root mean square deviation of only 0.66 Å for the C_α atoms. Consistent with previous studies of the quaternary structures of these enzymes,

phenylalanine hydroxylase is a dimer in the crystals, whereas tyrosine hydroxylase is tetrameric. Figure 4 shows the overall structure of the tyrosine hydroxylase tetramer. As predicted from mutational and proteolytic studies, the primary tetramer interface is a 24-residue α-helix at the C-terminus. Phenylalanine hydroxylase remains a dimer owing to additional interactions involving residues 401–420.

In the absence of bound substrates or inhibitors, the active site can be tentatively identified from the location of the iron atom. This assignment is supported by the results of Martinez et al (34–36), who used the paramagnetic effects of the metals in tyrosine and phenylalanine hydroxylase to show that both the aromatic ring of the amino acid substrates and the tetrahydropterin bind closely to the metal. In both structures the iron is located in a hydrophobic cleft 10 Å from the surface. The surface of this cleft is made up primarily of four helices containing mostly hydrophobic residues, consistent with the hydrophobic nature of the substrates. There is a short loop, which covers the entrance to the active site, containing residues 378-381 in phenylalanine hydroxylase and 427–429 in tyrosine hydroxylase. Binding to rat phenylalanine hydroxylase of an antibody

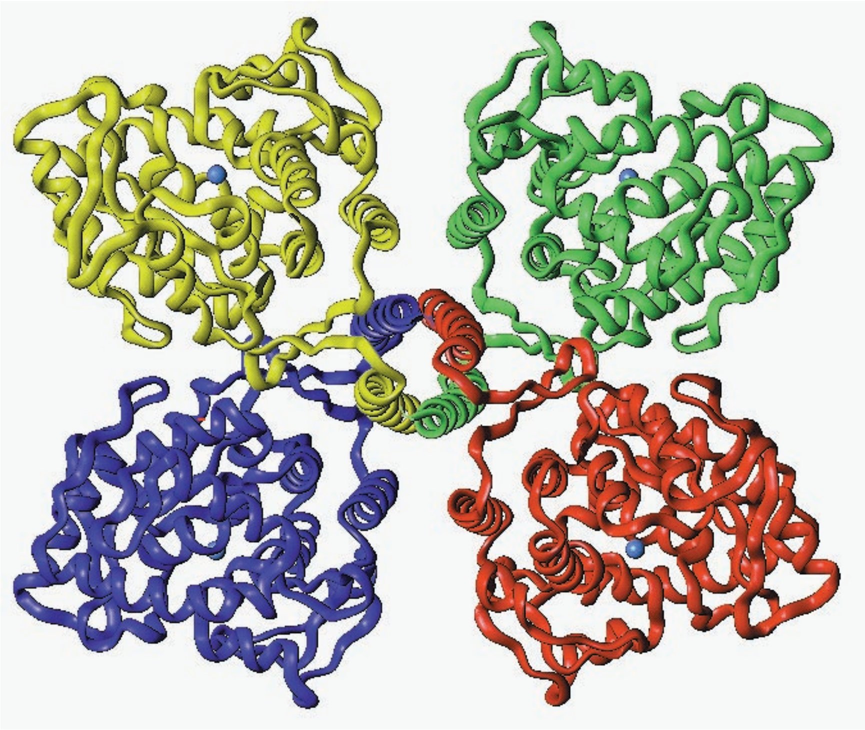

Figure 4 Ribbon diagram of the tetrameric catalytic domain of rat tyrosine hydroxylase. This figure was prepared using PDB file 1TOH.

Annu. Rev. Biochem. 1999.68:355-381. Downloaded from www.annualreviews.org
Access provided by Universita Degli Studi di Torino - All Libraries on 11/16/20. For personal use only.

Annu. Rev. Biochem. 1999.68:355-381. Downloaded from www.annualreviews.org
Access provided by Universita Degli Studi di Torino - All Libraries on 11/16/20. For personal use only.

raised to a synthetic peptide corresponding to residues 263–289 is prevented by dihydropterin (37); this peptide contains 10 residues found in the proposed active site cleft (32), further supporting the assignment of the active site to the cleft containing the iron.

The roles of the majority of the individual active-site residues have not been established. The structure of the iron site in tyrosine hydroxylase is shown in Figure 5. There are three amino acid ligands to the metal, histidines 331 and 336 and glutamate 376. In addition, there are two solvent molecules 2.0 Å from the iron, resulting in square pyramidal geometry with histidine 331 as the axial ligand. The iron site in phenylalanine hydroxylase is essentially identical, except that three water molecules are within 2.3 Å of the iron (32). The difference in the number of water molecules may be caused by the higher resolution of the phenylalanine hydroxylase structure, 2.0 Å compared with 2.3 Å for tyrosine hydroxylase. The additional water molecule results in a six-coordinate iron atom with octahedral geometry; the axial ligands are histidine 285 and a water molecule. In both enzymes, these histidine residues had previously been identified as iron ligands by site-directed mutagenesis (29, 38). Mutation to alanine of glutamate 286 of rat phenylalanine hydroxylase increases the K_m value for BH_4 about 100-fold, suggesting that this residue has a role in pterin binding (19). In the structure of human phenylalanine hydroxylase, this residue is hydrogen bonded to one of the water ligands to the iron (32).

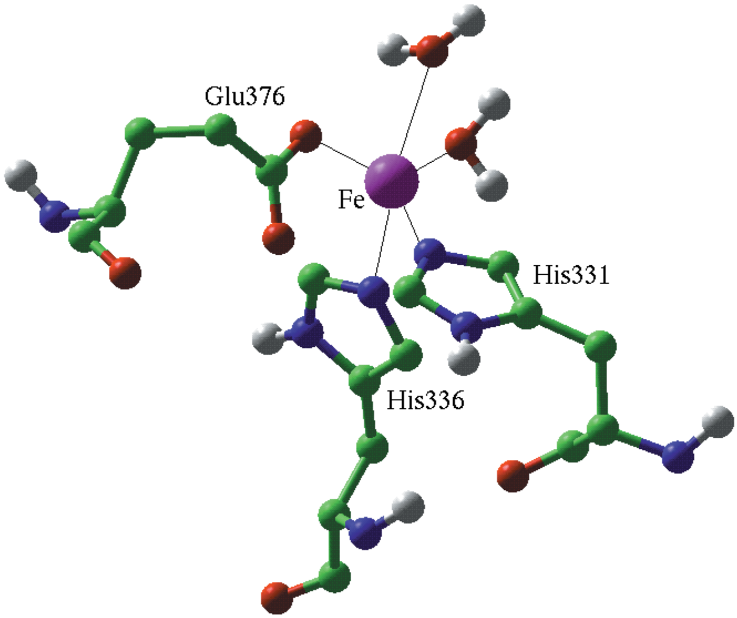

Figure 5 The iron site of rat tyrosine hydroxylase.

Annu. Rev. Biochem. 1999.68:355-381. Downloaded from www.annualreviews.org
Access provided by Universita Degli Studi di Torino - All Libraries on 11/16/20. For personal use only.

MECHANISM

In light of the homologies of the catalytic domains of these enzymes, it is not surprising that their mechanisms appear to be similar. Indeed, in the following discussion the assumption is made that the mechanisms are identical. This allows results obtained with either phenylalanine or tyrosine hydroxylase to be used to build up a self-consistent mechanistic picture.

Metal Requirement

Both phenylalanine and tyrosine hydroxylase were found to contain slightly less than one iron atom per monomer when first purified (39, 40). A direct relationship between the iron content and the activity was subsequently established for both enzymes (41, 42). The iron is in the ferric form when the enzymes are purified (39, 43). Reduction of the iron to the ferrous form is required for phenylalanine hydroxylase to be active (44, 45), although metal reconstitution studies have shown that tyrosine hydroxylase requires ferrous iron for activity (42). Tetrahydropterins can reduce the iron atom in either enzyme, suggesting that BH_4 is the physiological reductant (44-46). The ferrous iron is readily oxidized by molecular oxygen (46, 47). Tryptophan hydroxylase has recently been shown to require ferrous iron for activity (7). No metal has been found which can replace the iron in forming active enzyme with any of the three hydroxylases, although several other metals will bind in place of the iron (48, 49).

In contrast to the eukaryotic enzymes, phenylalanine hydroxylase from *C. violaceum* contains a stoichiometric amount of copper when purified (2). Catalysis requires reduction of the copper, with dithiothreitol being the most effective reductant (50). The presence of a metal other than iron in a protein that was homologous to the eukaryotic enzymes raised obvious questions about the mechanistic role of the metal in these enzymes. The situation was further complicated with the report that reduction of the copper by thiols led to its dissociation, and the copper-free enzyme was fully active (51). This result led to the proposal that this phenylalanine hydroxylase does not require any metal for activity (51, 52). However, Chen & Frey (13) have recently reported that the *C. violaceum* enzyme is unable to hydroxylate phenylalanine in the absence of iron and requires a stoichiometric amount of iron for full activity.

Steady State Kinetics

Complete steady state kinetic mechanisms have been described for *C. violaceum* phenylalanine hydroxylase (53) and for rat tyrosine hydroxylase (54). Both studies are consistent with a tri-bi sequential mechanism in which no chemistry occurs until all substrates are bound. In the case of phenylalanine hydroxylase, the data are consistent with binding of oxygen as the first substrate, followed by binding of phenylalanine and 6,7-dimethyltetrahydropterin in random order; some preference for binding of the pterin before the amino acid was noted (53).

Annu. Rev. Biochem. 1999.68:355-381. Downloaded from www.annualreviews.org
Access provided by Universita Degli Studi di Torino - All Libraries on 11/16/20. For personal use only.

Less complete analyses have been carried out with eukaryotic phenylalanine hydroxylase; in the rat enzyme the tetrahydropterin appears to bind before phenylalanine (47). The data with tyrosine hydroxylase were interpreted in favor of a fully ordered mechanism, with 6-methyltetrahydropterin (6-MePH_4), oxygen, and tyrosine binding in that order; oxygen binds in rapid equilibrium, so that no K_m value is observed for this substrate (54).

Substrate Specificity

Pterin Specificity A variety of substitutions at the 6 position of the tetrahydropterin ring (I), where the natural substrate has a dihydroxypropyl side chain, are tolerated in substrates for at least one of the pterin-dependent hydroxylases. The most commonly used substrate has been 6-MePH_4, a good substrate for all three enzymes (55-57). Phenyl, ethyl, hydroxymethyl, and trihydroxypropyl side chains are also tolerated at the 6 position (56, 58), but not a carboxylate (59), consistent with the hydrophobic nature of the active sites. Substitution of a methyl group at the 7 position is tolerated, but this results in some of the tetrahydropterin being consumed unproductively by both phenylalanine and tyrosine hydroxylase (55, 56). Disubstitution at the 6 position is also tolerated by tyrosine hydroxylase (60). Tetrahydropterins substituted at the 5 or 8 position are not substrates for tyrosine hydroxylase (56, 60). 2,4-Diamino-6,7-dimethyl-tetrahydropterin is reported to be a substrate for phenylalanine hydroxylase (59), but replacement of the oxygen at C2 results in loss of activity with both phenylalanine and tyrosine hydroxylase (59, 60).

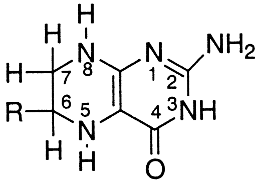

I

The complete pyrazine ring of the pterin is not required for activity, because 2,5,6-triamino-4-pyrimidinone (II) and 5-benzylamino-2,6-diamino-4-pyrimidinone will support hydroxylation by phenylalanine hydroxylase (61, 62). The pyrimidodiazepine III is also a slow substrate for phenylalanine hydroxylase (63), further establishing that the pyrimidine ring is critical.

Amino Acid Specificity There are two aspects to the amino acid specificity of the pterin-dependent hydroxylases: the relative specificities for the three aromatic amino acids as substrates and the range of amino acids that can be hydroxylated. The relative affinities of tyrosine and phenylalanine hydroxylase

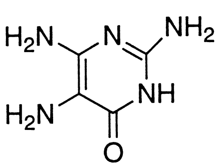

II

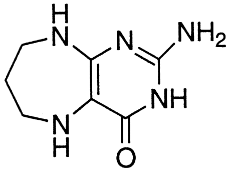

III

for different amino acid substrates are affected by the presence of the regulatory domains (64). In addition, discussions of the substrate specificity of phenylalanine hydroxylase are complicated by the need for activation by an amino acid substrate when BH_4 is used for the nonactivated wild-type enzyme (65). With respect to the intact enzymes, each of the three hydroxylases will utilize all three aromatic amino acids as substrates to some extent. Phenylalanine hydroxylase will hydroxylate tryptophan (55), with a K_m value 100-fold and a V_{max} value 2.6% those for phenylalanine (65). With tyrosine as the substrate, a tetrahydropterin oxidase activity results, in that no dihydroxyphenylalanine can be detected (66); the V_{max} and V/K_{tyr} values for this reaction are only 5% the values found with phenylalanine as substrate (64). An inability to hydroxylate tyrosine is consistent with the physiological role of this enzyme of producing tyrosine. In contrast, tyrosine hydroxylase will use phenylalanine as a substrate (67, 68), forming both tyrosine and small amounts of 3-hydroxyphenylalanine (69). The V_{max} value for this reaction is within a factor of four of that seen with tyrosine, whereas the K_m value for phenylalanine is sixfold that for tyrosine (64). This has led to the suggestion that tyrosine hydroxylase could catalyze the formation of dihydroxyphenylalanine from phenylalanine in cells (70). Tryptophan is a poor substrate for tyrosine hydroxylase, with a K_m value some 20-fold that of tyrosine and a significantly lower V_{max} value (71). Tryptophan hydroxylase can hydroxylate phenylalanine (57); the kinetic parameters obtained with the recombinant catalytic core are very similar to those for tryptophan (7). Tyrosine is a poor substrate for this enzyme, with most of the tetrahydropterin being consumed unproductively (7, 57).

In light of the homology of the catalytic domains, the regulatory domains have been proposed to be responsible for the substrate specificity of these enzymes (72). Indeed, the isolated catalytic domains of rat phenylalanine and tyrosine hydroxylase show reduced specificities for phenylalanine versus tyrosine, consistent with such a role for the regulatory domains (64). However, the isolated catalytic domain of phenylalanine hydroxylase is still unable to form tyrosine. Chimeric proteins containing the regulatory domain of one of these enzymes and the catalytic domain of the other have substrate specificities similar to that of the intact protein that supplied the catalytic domain. Thus, the absolute substrate specificity is determined by the catalytic domain. The effect of the regulatory domain on specificity is on the general shape and flexibility of the active site (64).

Both phenylalanine and tyrosine hydroxylase will accept as substrates a range of *p*-substituted phenylalanines. For bovine tyrosine hydroxylase there is a

Annu. Rev. Biochem. 1999.68:355-381. Downloaded from www.annualreviews.org
Access provided by Universita Degli Studi di Torino - All Libraries on 11/16/20. For personal use only.

correlation between the K_i values of these compounds versus tyrosine and the size of the *p*-substituent (73), suggesting a constricted binding site. Table 1 lists different amino acids that have been reported to be substrates for these enzymes, along with the reported products. The range of reactions includes aromatic hydroxylation of heteroatom-substituted compounds, benzylic and aliphatic hydroxylation, epoxidation, and sulfoxidation.

Annu. Rev. Biochem. 1999.68:355-381. Downloaded from www.annualreviews.org
Access provided by Universita Degli Studi di Torino - All Libraries on 11/16/20. For personal use only.

Table 1 Alternate amino acid substrates for the tetrahydropterin-dependent hydroxylases

Enzyme	Substrate	Products
Phenylalanine hydroxylase	Tryptophan	5-HO-Tryptophan[a]
	4-F-Phenylalanine	Tyrosine[a]
	4-Cl-Phenylalanine	4-Cl-3-HO-Phenylalanine, tyrosine, 3-Cl-tyrosine[b]
	4-Br-Phenylalanine	3-Br-Tyrosine[b]
	4-Ch_3-Phenylalanine	4-$HOCH_2$-Phenylalanine, 4-CH_3-3-HO-phenylalanine, 3-CH_3-tyrosine[c]
	L-Methionine	Methionine sulfoxide[d]
	D-Methionine	D-Methionine sulfoxide[d]
	Norleucine	e-Hydroxynorleucine[d]
	D-Phenylalanine	D-Tyrosine[d]
	[2,5-H_2]Phenylalanine	[2,5-H_2]Phenylalanine epoxide[e]
	Cyclohexylalanine	7-Hydroxy-cyclohexylphenylalanine[f]
Tyrosine hydroxylase	Phenylalanine	Tyrosine,[g] 3-HO-phenylalaine[h]
	4-F-Phenylalanine	Tyrosine[i]
	4-Cl-Phenylalanine	4-Cl-3-HO-Phenylalanine, tyrosine, 3-Cl-tyrosine[i]
	4-Br-Phenylalanine	4-Br-3-HO-Phenylalanine, tyrosine, 3-Cl-tyrosine[i]
	4-CH_3-Phenylalanine	4-$HOCH_2$-Phenylalanine, 4-CH_3-3-HO-phenylalanine, 3-CH_3-tyrosine[i]
	4-CH_3O-Phenylalanine	4-CH_3O-3-HO-Phenylalanine[i]
	3-HO-Phenylalanine	Dihydroxyphenylalanine[j]
	3-F-Tyrosine	Dihydroxyphenylalanine[j]
	3-Cl-Tyrosine	Dihydroxyphenylalanine[j]
	3-Br-Tyrosine	Dihydroxyphenylalanine[j]
Tryptophan hydroxylase	Phenylalanine	Tyrosine[k]

[a]Reference 55.
[b]Pseudomonad enzyme (74).
[c]No 4-CH3-3-HO-phenylalanine was detected using the enzymes from rat liver enzyme (75) or *Chromobacterium violaceum* (52), but all three were reported as products with the pseudomonad enzyme (76).
[d]Reference 65.
[e]Reference 77.
[f]Reference 52.
[g]Reference 67, 68.
[h]Reference 69.
[i]Reference 78.
[j]Reference 71.
[k]Reference 57.

Catechol Inhibition Phenylalanine and tyrosine hydroxylase will each form very tight complexes with catechols, with K_d values in the nanomolar range (47, 79). In both cases, spectroscopic analyses indicate that both oxygens of the catechol are ligands to the iron in the ferric state (80, 81). Catecholamines are also reported to be inhibitors of tryptophan hydroxylase, but the interaction has not been characterized (82). Only for tyrosine hydroxylase is there evidence that this inhibition is physiologically relevant. Indeed, when tyrosine hydroxylase is isolated from nonrecombinant sources, the enzyme contains a mixture of catecholamines bound to the iron (83, 84).

Rate-Determining Step

A minimal kinetic mechanism for a hydroxylase would involve substrate binding, formation of the hydroxylating intermediate, hydroxylation, and product dissociation. The most extensive kinetic studies have been carried out with rat tyrosine hydroxylase. With tyrosine as substrate, there is no burst of dihydroxyphenylalanine formation after the first turnover, ruling out slow product release as rate limiting (71). There is no significant isotope effect when [3,5-$^2H_2$]-tyrosine is used as substrate, ruling out carbon-hydrogen bond cleavage during hydroxylation as rate limiting (54). When the rate of tetrahydropterin oxidation is monitored, the V_{max} value for a series of substituted phenylalanines is independent of the identity of the amino acid substrate, suggesting that no chemistry involving the amino acid occurs in the rate-limiting step (71). These observations are consistent with formation of the hydroxylating intermediate being rate limiting; the subsequent hydroxylation and product release steps would be much faster. With phenylalanine hydroxylase there is no significant kinetic-isotope effect with ring-deuterated phenylalanine as substrate when BH_4 is used (85). However, with 6-MePH_4 a small kinetic isotope effect is seen, suggesting that the rates of oxygen activation and hydroxylation are comparable in this case.

Chemical Mechanism

Identity of the Hydroxylating Intermediate As shown in Figure 1, one of the products of the reactions catalyzed by all three hydroxylases is a 4a-hydroxypterin. The oxygen atoms in both the amino acid and the pterin products have been shown to come from molecular oxygen (8, 86). The formation of this compound establishes that there must be a reaction between molecular oxygen and tetrahydropterin during catalysis. Model studies of the reaction of oxygen with reduced tetrahydropterins (87) and the well-established formation of 4-peroxyflavins by flavin monooxygenases (88) suggest that a 4a-peroxytetrahydropterin is the immediate result of the reaction of the tetrahydropterin and molecular oxygen. Based on model studies with both flavins and pterins, this reaction is expected to occur in two steps: an initial slow, single-electron transfer to form superoxide and the pterin cation radical followed by rapid combination of the two radicals (Figure 6) (87, 89). The ^{18}O kinetic-isotope effect of 1.016 seen with tyrosine hydroxylase is consistent with such a reaction (90).

Annu. Rev. Biochem. 1999.68:355-381. Downloaded from www.annualreviews.org
Access provided by Universita Degli Studi di Torino - All Libraries on 11/16/20. For personal use only.

Annu. Rev. Biochem. 1999.68:355-381. Downloaded from www.annualreviews.org
Access provided by Universita Degli Studi di Torino - All Libraries on 11/16/20. For personal use only.

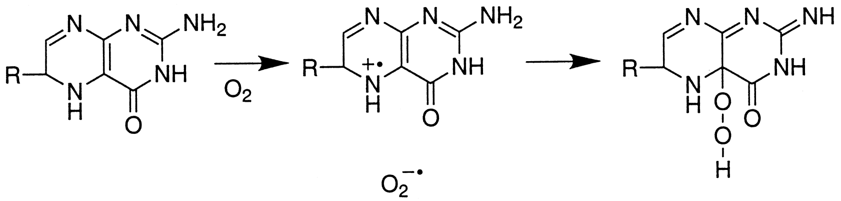

Figure 6 Mechanism of reaction of tetrahydropterins with oxygen.

The 4a-peroxypterin must be considered as a candidate for the hydroxylating intermediate, since the analogous 4a-peroxyflavin is thought to be the hydroxylating intermediate in the flavin phenol hydroxylases (88). However, the range of reactions listed in Table 1 resembles the reactions catalyzed by cytochrome P-450–dependent hydroxylases, in which the hydroxylating intermediate is a high-valence iron-oxo species (91), rather than those catalyzed by the flavin phenol hydroxylases (88). For example, *p*-hydroxybenzoate hydroxylase cannot hydroxylate the nonactivated aromatic ring in benzoate (92), a reaction analogous to that catalyzed by phenylalanine hydroxylase, and has not been reported to carry out epoxidation or aliphatic hydroxylation reactions. Thus, it seems likely that a more reactive species containing the iron, either a pterin-oxygen-iron species or an iron-oxygen species (8), is involved.

The products from uncoupled turnover by phenylalanine hydroxylase have been used as a probe for the mechanistic fate of such a peroxypterin. With a variety of nonphysiological substrates, all three enzymes oxidize significantly more than 1 mol of tetrahydropterin per mol of amino acid hydroxylated. If a peroxypterin is the hydroxylating intermediate, such uncoupled turnover could result in production of hydrogen peroxide and quinonoid dihydropterin as products upon the breakdown of the peroxypterin. Dix and Benkovic (93) reported that about threefold as much tetrahydropterin is consumed as hydroxylated amino acid is produced when 4-Cl-phenylalanine is a substrate for phenylalanine hydroxylase. The amount of hydroxylated amino acid formed agreed well with the amount of 4-hydroxypterin produced, suggesting that the oxygen-oxygen bond is not cleaved unless hydroxylation occurs. No hydrogen peroxide was detected; these authors proposed that oxygen is reduced to water in unproductive turnover. Contradictory results were reported by Davis & Kaufman (66, 94) with tyrosine as the substrate for this enzyme. Tyrosine is not hydroxylated by phenylalanine hydroxylase, but rather converts the enzyme to a slow tetrahydropterin oxidase. Both the hydroxypterin and hydrogen peroxide were detected as products in this reaction. Such a result requires cleavage of the oxygen-oxygen bond of a peroxytetrahydropterin intermediate even in the absence of oxygen atom transfer to an amino acid substrate. These authors suggested that the peroxypterin is an intermediate in the formation of the true hydroxylating intermediate, a high-valence iron-oxo compound. The branched pathway shown in Figure 7 was proposed to account for the results (94).

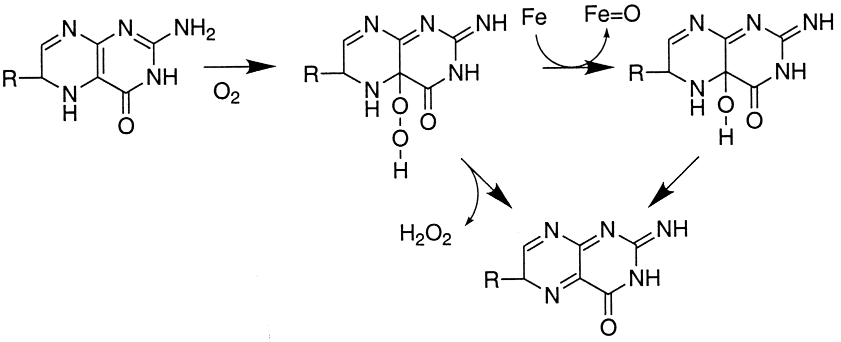

Figure 7 Mechanism of Davis & Kaufman (94) for turnover of phenylalanine hydrozylase in the presence of tyrosine.

An alternative possibility involving activation of the peroxypterin via a pyrazine ring-opened species was proposed by Hamilton for both pterin and flavin-dependent monooxygenases (95). The loss of substituted amines from the 4 position of triaminopyrimidines with phenylalanine hydroxylases was initially interpreted in favor of such a mechanism (96, 97). However, such an intermediate seems unlikely in light of the inability of this enzyme to catalyze the ring closure reaction required to regenerate the intact pterin (4). Instead, the loss of the benzylamino group would be caused by cleavage of the CN bond in the carbinolamine product being more favorable than cleavage of the CO bond, as a result of the relative leaving group propensities of the two substituents.

Although the range of reactions carried out by these enzymes and the reported production of the hydroxypterin in uncoupled turnover with phenylalanine hydroxylase are consistent with the hydroxylating intermediate being an iron-oxo species, it should be emphasized that there is no direct evidence for such a role for the iron in these enzymes. Iron is clearly required for activity, and mutation of the ligands to the iron results in loss of detectable activity (29, 38). However, the electron paramagnetic resonance (EPR) spectrum of enzyme frozen at 7 K during turnover does not show any EPR-detectable species; this can be rationalized by formation of the hydroxylating intermediate being much slower than its subsequent reaction (46). An additional potential role for the iron atom would be to assist in the reaction of tetrahydropterin and molecular oxygen to form the peroxypterin. The ^{18}O kinetic-isotope effects determined by Francisco et al (90) are consistent with either a single-electron transfer from tetrahydropterin to oxygen, as shown in Figure 6 or with equilibrium binding of O_2 to the iron atom followed by single-electron transfer from the tetrahydropterin to the iron-bound oxygen. Chen & Frey (13) have reported that metal-free phenylalanine hydroxylase from *C. violaceum* will catalyze tetrahydropterin oxidation but not phenylalanine hydroxylation. This suggests that the iron is required for formation of the hydroxylating intermediate but is not involved in the initial reaction of oxygen and the tetrahydropterin. If this is the case, the peroxypterin must react with

Annu. Rev. Biochem. 1999.68:355-381. Downloaded from www.annualreviews.org
Access provided by Universita Degli Studi di Torino - All Libraries on 11/16/20. For personal use only.

Annu. Rev. Biochem. 1999.68:355-381. Downloaded from www.annualreviews.org
Access provided by Universita Degli Studi di Torino - All Libraries on 11/16/20. For personal use only.

the iron en route to formation of any iron-oxo hydroxylating species. A reasonable intermediate for this reaction suggested by Dix et al (8) is the peroxypterin-iron complex IV. Heterolytic cleavage of the oxygen-oxygen bond in such a species would generate the peroxypterin and the iron-oxo hydroxylating species directly (98). Complex IV must also be considered a viable candidate for the hydroxylating intermediate.

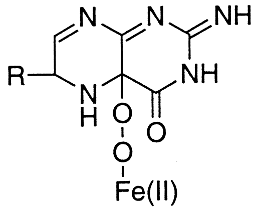

IV

The ability of cytochrome P-450–dependent hydroxylases to utilize peroxides and other oxidants in place of reducing equivalents and oxygen, the "peroxide shunt," has provided a more direct probe of the role of the iron in those systems (91). In pterin-dependent enzymes, it has been reported that tyrosine hydroxylase can hydroxylate tyrosine to dihydroxyphenylalanine by using hydrogen peroxide in the absence of any tetrahydropterin (5), suggesting that the pterin is not part of the hydroxylating intermediate. However, Ribeiro et al (68) reported that they were unable to reproduce this result.

Mechanism of Hydroxylation It was noted early on that the tyrosine produced from [p-^{3}H] phenylalanine by phenylalanine or tyrosine hydroxylase and the [5-HO]-tryptophan produced from [5-^{3}H]-tryptophan by tryptophan hydroxylase still contained most of the radioactivity (86, 99, 100). In each case the label was attached to the carbon adjacent to the site of hydroxylation. As is clear from the products obtained with substituted phenylalanines as substrates for both phenylalanine and tyrosine hydroxylase, substituents other than hydrogen isotopes can also undergo such an NIH shift. These include Cl, Br, and methyl substituents, but not F or methoxy. The observation of an NIH shift in a variety of hydroxylation reactions led Guroff et al (101) to propose the cationic cyclohexadienyl intermediate V for phenylalanine hydroxylase. Such an intermediate could then rearomatize, losing a proton or tritium. A significant kinetic isotope effect on the proton loss step would result in preferential retention of tritium. The observation that arene oxides are products of a number of aromatic hydroxylation reactions led to the proposal that the arene oxide VI was formed as a precursor to V (102). The formation of an epoxide from [2,5-H_2]phenylalanine by phenylalanine hydroxylase is consistent with the involvement of an arene oxide (77), although it rigorously only establishes the ability of the enzyme to carry out epoxidation reactions. As a more direct probe for the involvement of an arene oxide, the isotope effects

on product distribution upon hydroxylation of [4-^{2}H]phenylalanine and [3,5-$^2H_2$]phenylalanine by tyrosine hydroxylase were determined (69). In both cases there was a decrease in the hydroxylation at the site of the isotope, but no detectable increase in hydroxylation at the nondeuterated position. These results rule out the partitioning of a common intermediate such as an arene oxide in the formation of these two products.

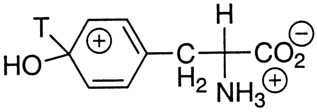

V

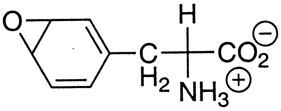

VI

More recently, the product distribution from a series of *p*-substituted phenylalanines as substrates for tyrosine hydroxylase was used as a mechanistic probe (78). There was a good correlation of the site of oxygen addition with the size of the substituent at the 4 position of the aromatic ring, with a transition from primarily *p*-hydroxylation with phenylalanine to exclusively *m*-hydroxylation with 4-CH_3O-phenylalanine. Thus, with this enzyme at least, the site of hydroxylation of these nonphysiological substrates is determined primarily by steric factors. Increasing amounts of tetrahydropterin were oxidized without concomitant hydroxylation of the amino acid as the electron-donating ability of the substituent decreased, giving a ρ-value of about -5 with both 6-MePH_4 and BH_4. This result is consistent with a cationic intermediate. Moreover, the amount of NIH shift seen varied with the identity of the substituent in a manner consistent with a cationic species. The cationic cyclohexadiene VII was proposed as the product of the initial oxygen addition; the similarity of this compound to V is obvious. VII would then rearrange directly to the phenolic product.

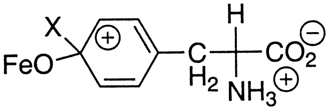

VII

REGULATORY PROPERTIES

The catalytic domains of the pterin-dependent hydroxylases are sufficiently similar to assume that all three enzymes have essentially the same catalytic mechanism. In contrast, the regulatory domains of the eukaryotic enzymes are not homologous. Thus, it is not surprising that the regulatory properties are distinct. As a result, the regulatory properties of each enzyme will be discussed separately.

Annu. Rev. Biochem. 1999.68:355-381. Downloaded from www.annualreviews.org
Access provided by Universita Degli Studi di Torino - All Libraries on 11/16/20. For personal use only.

Annu. Rev. Biochem. 1999.68:355-381. Downloaded from www.annualreviews.org
Access provided by Universita Degli Studi di Torino - All Libraries on 11/16/20. For personal use only.

Phenylalanine Hydroxylase

Virtually all of the characterization to date has been done with the enzyme from rat liver. A variety of treatments, some of which are clearly not physiological, will increase the activity of phenylalanine hydroxylase from eukaryotic sources. When the concentration of phenylalanine is varied, sigmoidal kinetics are observed if BH_4 is used as substrate (18); the effect is less pronounced if other tetrahydropterins are used (103, 104). Treatment of the enzyme with chymotrypsin or lysolecithin increases the measured V_{max} value and gives sigmoidal kinetics. Activation by proteolysis correlates with the loss of about 100 residues from the amino terminus (22); this would correspond to removal of the regulatory domain. Consistent with this, the isolated catalytic domain does not require pretreatment for full activity (64, 105). Treatment with thiol-modifying reagents such as N-ethyl maleimide has a similar activating effect (103). Indeed, treatment with lysolecithin exposes a thiol residue (18), suggesting that the two treatments result in similar conformational changes. The reactive thiol has been identified as cysteine 236 (106). With other tetrahydropterins such as 6-$MePH_4$ and 6,7-dimethyltetrahydropterin, the enzyme activity is reported to be high without such pretreatment (103, 104).

The effects of phenylalanine and BH_4 on the activity are more likely to be physiologically relevant than these chemical modifications. If assays are started by adding enzyme, there is a lag in the rate of tyrosine formation (107). Pretreatment of the enzyme with phenylalanine abolishes the lag, whereas pretreatment with BH_4 or 6-$MePH_4$ increases it (107). Treatment with lysolecithin also abolishes the lag. Based on an extensive analysis of the effects of phenylalanine and pterins on the kinetics, Shiman and coworkers have proposed a model (Figure 8) for the effects of amino acids and pterins on the activity of phenylalanine hydroxylase (47, 108, 109). The enzyme as isolated is proposed to be in an inactive form, E_i. Binding of phenylalanine converts it to an active form, E_a; this would involve formation of an active site for phenylalanine (108). Tetrahydropterins bind to E_i, trapping the enzyme in the inactive form. BH_4 binds E_i more tightly than 6-$MePH_4$ (109). Kinetic modeling based on such a model successfully predicts the low activity of phenylalanine hydroxylase in tissues, owing to the predominance of the BH_4-E_i complex (110). This regulatory mechanism results in a large increase in phenylalanine hydroxylase activity in response to an increase in plasma phenylalanine levels (109).

Data from Parniak & Kaufman (103) are suggestive of two separate phenylalanine-binding sites, a regulatory site and a separate active site. The unmodified enzyme is reported to bind 1.5 equivalents of phenylalanine per monomer with strong positive cooperativity, whereas the thiol-modified enzyme binds only one equivalent with no cooperativity. In agreement with a two-site model, the activating phenylalanine remains bound during multiple turnovers without being hydroxylated (111). Activation by phenylalanine has been attributed to displacement of an inhibitory peptide, presumably in the regulatory domain; proteolysis would remove

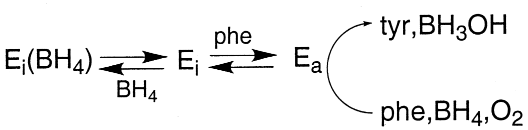

Figure 8 Regulatory mechanism of phenylalanine hydroxylase.

the peptide completely, whereas chemical modification would disrupt its interaction with the catalytic domain (103). Several observations are suggestive of a conformational change upon conversion from E_i to E_a. Activation by phenylalanine or lysolecithin converts the enzyme to a form that will bind phenylalanine (107, 112); this is used as a purification step for phenylalanine hydroxylase (112). The activated enzyme is slightly more sensitive to proteases, whereas binding of BH_4 decreases the protease sensitivity (104). Phenylalanine binding alters the fluorescence properties (111, 113) and increases the total volume about 10% (25). BH_4 has an opposite effect on the fluorescence properties (113). These data are consistent with E_a having a more open conformation than E_i. Although activation is proposed to result in formation of a competent active site for binding phenylalanine (108), the iron environments in the two enzyme forms are indistinguishable (25).

Amino acids other than phenylalanine will activate the enzyme, although frequently not as well (65, 107). The E_a form of the enzyme is also reported to have a broader substrate specificity (65), although the need to separate the specificities for activation and catalysis clearly complicates such analyses.

In addition to the regulation by substrates, phenylalanine hydroxylase is regulated by phosphorylation of serine 16 (114). Interpretation of the effects of phosphorylation is complicated by the presence of 0.2-0.3 phosphates per monomer in the enzyme as isolated (111, 115), although some recent studies have been carried out with recombinant enzyme which avoid this problem (116, 117). Treatment with cAMP-dependent protein kinase A (PKA) increases the amount of phosphate per monomer to one (115). A calmodulin-dependent protein kinase (CaM-PK) will phosphorylate the same site as PKA (118). The phosphorylated enzyme is reported to be two- to fourfold as active as the unphosphorylated enzyme (115, 116), but the activation is reported to depend on whether BH_4 is used (115). The S16D and S16E mutants have similar activities to the phosphorylated enzyme, consistent with the effect being caused by the introduction of the negative charge (116). Both the phosphorylated enzyme and the mutants are activated further by lysolecithin (115, 116). The phosphorylated enzyme still shows sigmoidal kinetics with varied phenylalanine (115); the increased activity is primarily owing to an increased V_{max} value (116).

A critical observation in interpreting the effects of phosphorylation on phenylalanine hydroxylase is that activation of the phosphorylated enzyme by phenylalanine is more rapid and occurs at lower concentrations than with the unphosphorylated enzyme (119). A similar observation has been made with the recombinant human enzyme (117). Thus, phosphorylation appears to modulate the

Annu. Rev. Biochem. 1999.68:355-381. Downloaded from www.annualreviews.org
Access provided by Universita Degli Studi di Torino - All Libraries on 11/16/20. For personal use only.

balance between the E_a and E_i forms of phenylalanine hydroxylase. Moreover, BH_4 inhibits the phosphorylation (117, 120, 121), suggesting that the BH_4-E_i complex is phosphorylated more slowly or not at all. In light of the complexity of the regulatory properties of the unphosphorylated enzyme, it seems likely that the magnitude of the effects of phosphorylation on activity described to date does not directly reflect the magnitude of the change in a specific rate or equilibrium constant. Clearly defining the effects of phosphorylation will likely require an extensive analysis comparable to that carried out on the unphosphorylated enzyme.

Annu. Rev. Biochem. 1999.68:355-381. Downloaded from www.annualreviews.org
Access provided by Universita Degli Studi di Torino - All Libraries on 11/16/20. For personal use only.

Tyrosine Hydroxylase

The principal means of post-translational regulation of tyrosine hydroxylase appears to be reversible phosphorylation of serine residues in the regulatory domain (122). Despite more than two decades of study by a number of different laboratories, the details of the effects of phosphorylation and the physiological relevance of the different phosphorylation sites are still not fully established. This is caused in part by the difficulty of obtaining purified nonrecombinant protein, the complexity of the problem, and the lack of realization in earlier studies that the enzyme isolated from eukaryotic sources contains bound catecholamines. Because of space limitations, an extensive discussion of the literature cannot be given here. Many of the earlier studies are described in a review by Zigmond et al (122), and a more extensive discussion of agents that modulate phosphorylation of tyrosine hydroxylase in cells is given by Haycock (123).

There are four serine residues in rat tyrosine hydroxylase, which are phosphorylated both in vivo and in vitro, at positions 8, 19, 31, and 40 in the rat enzyme (124, 125). Human tyrosine hydroxylase has a threonine residue at position 8 (14). There is general consensus that PKA is specific for phosphorylation of serine 40 (125, 127). Purified PKA will phosphorylate serine 40 specifically (125), and agents that increase cAMP levels in cells increase the level of phosphorylation of this residue (123, 124, 128). Serine 19 can be phosphorylated in the pure enzymes by CaM-PKII (125, 127, 129) and by mitogen-activated protein (MAP) kinase-activated protein kinases (130). CaM-PKII also phosphorylates serine 40 (125, 127, 129) and has been reported to phosphorylate serine 31 in the human isoform 2. MAP kinase-activated protein kinase 1 phosphorylates both serine 19 and 40 in the human hydroxylase, but MAP kinase-activated protein kinase 2 phosphorylates only serine 40 (130). In cells, calcium or agents that increase the levels of intracellular calcium also increase the level of phosphorylation of serine 19 (124, 131–133), although additional residues have been reported to be phosphorylated under these conditions (123, 131, 133, 134). In the pure enzyme, serine 31 can be phosphorylated by the MAP kinases ERK1 and ERK2 (130, 135, 136). In cells, phorbol esters and nerve growth factor increase the activities of ERK1 and ERK2 and the level of phosphorylation of serine 31 (123, 124, 131, 132, 135). No kinase has been identified that phosphorylates serine 8. In cells only phosphatase inhibitors have been found to alter the level of phosphorylation of this residue

(124). Thus the most likely candidates for the physiological kinases are CaM-PKII for serine 19, ERK for serine 31, and PKA for serine 40 (123).

Evaluation of the effects of phosphorylation on the properties of tyrosine hydroxylase is clearly complicated by the number of phosphorylation sites. It has been further complicated by the fact that enzyme isolated from tissues contains substoichiometric amounts of covalent phosphate (43, 137); the studies cited above suggest that the phosphate is distributed among the different serine residues. The discussion here will be limited to studies carried out with purified proteins. The earliest studies were done with wild-type enzymes isolated from tissues. These showed that rat tyrosine hydroxylase could be phosphorylated by PKA (138), with the incorporation of up to 0.7 mol of phosphate per monomer (139). When the enzyme was assayed at pH 6, the effect of phosphorylation was to decrease the value of the K_m for 6-$MePH_4$ without affecting the V_{max} value or the K_m value for tyrosine (138, 139). It was subsequently reported with the bovine brain enzyme that phosphorylation increases the pH optimum from 6 to about 6.6, so that the effects of phosphorylation by PKA depend on the pH of the assays (140). At pH 6, phosphorylation led to a decrease in the K_m for BH_4 of about threefold and an increase in the K_i value for dopamine of about twofold; at pH 7, the K_m value for BH_4 was down twofold, the V_{max} value was up threefold, and the K_i value for dopamine was up eightfold.

The observation that tyrosine hydroxylase purified from bovine or rat cells contained tightly bound catecholamines is clearly relevant to these early studies. Andersson et al (141) reported that the EPR spectrum of the enzyme was insensitive to the presence of substrate unless it was phosphorylated by PKA (43, 141). Upon phosphorylation the iron in the purified enzyme could be reduced by a tetrahydropterin (141). The same group subsequently reported that phosphorylation increased the rate of dissociation of bound norepinephrine by about sixfold; a pK_a value of 5.3 was measured for the dissociation of the unphosphorylated enzyme (142). These data thus suggested that a major effect of phosphorylation was to release a bound inhibitor rather than to simply increase the rate of catalytic steps. By using recombinant rat enzyme, Daubner et al (143) were able to separate the effects of phosphorylation of serine 40 on catecholamine binding and catalysis. The recombinant enzyme lacked both covalent phosphate and bound catecholamine; it also had a much higher specific activity than nonrecombinant enzyme. At pH 7, phosphorylation of serine 40 with PKA had only a modest effect, decreasing the K_m value for BH_4 about twofold, consistent with previous studies of the nonrecombinant enzyme. Binding of dopamine to the recombinant enzyme decreased its activity by a factor of 20; the specific activity of this dopamine-complexed enzyme was the same as nonrecombinant rat enzyme, consistent with the presence of catecholamines in the latter. Subsequent phosphorylation of the dopamine-bound recombinant enzyme resulted in a large increase in activity at pH 7 owing to a decrease of twofold in the K_m for BH_4 and an increase of tenfold in the V_{max} value. An identical effect was seen upon phosphorylation of the nonrecombinant enzyme. Thus, the effects of phosphorylation

Annu. Rev. Biochem. 1999.68:355-381. Downloaded from www.annualreviews.org
Access provided by Universita Degli Studi di Torino - All Libraries on 11/16/20. For personal use only.

of serine 40 on tyrosine hydroxylase can be attributed to a modest decrease in the K_m value for the tetrahydropterin and a release from the inhibition by bound catecholamines. Ramsey & Fitzpatrick (79) subsequently measured the effects of phosphorylation of serine 40 on the binding of dopamine and dihydroxyphenylalanine to both the inactive ferric and to the active ferrous forms of tyrosine hydroxylase. No significant effect was found on the binding of either catecholamine to the ferrous form of the enzyme. In contrast, there was a large effect on binding to the ferric enzyme. The affinity for dihydroxyphenylalanine decreased some 18-fold and that for dopamine over 300-fold upon phosphorylation; the effects were due to comparable increases in the dissociation rates.

Thus, with regard to serine 40, the effects of phosphorylation on activity can be explained by the mechanism shown in Figure 9. The unphosphorylated enzyme with ferrous iron is fully active. Oxidation of the iron to the ferric form results in inactive enzyme, but this can be reduced by BH_4. Accumulation of the product dihydroxyphenylalanine or dopamine results in formation of the inactive enzyme-catecholamine complex. Phosphorylation leads to dissociation of the inhibitory catecholamine, allowing the iron to be reduced and catalysis to commence.

The effects of phosphorylation at the other serine residues are much less understood. Vulliet and coworkers (144) showed that the rat enzyme could be phosphorylated by CaM-PK and that the phosphorylation occurred at a separate site from that labeled by PKA. No change in enzyme activity was detected on incorporation of 0.3 mol of phosphate per monomer catalyzed by CaM-PK. When the stoichiometry of labeling was 1.3/monomer, both serines 19 and 40 were labeled (125, 145). Treatment of the rat enzyme with CaM-PKII has been reported to have no effect on the activity unless a separate activator protein is included; in its presence the activity increases about twofold at pH 6 or 6.5 (146, 147). The activator protein has been identified as a neuronal protein of the 14-3-3 family (148, 149); this is a highly conserved family of brain proteins involved in a number of signal transduction pathways (150). Atkinson et al (147) also reported that no increase in the activity of the rat enzyme occurs upon phosphorylation by CaM-PKII unless a separate activator protein is included, in which case there is a 2.5-fold increase. In this case, when the stoichiometry of phosphate incorporation was 0.8/monomer, the majority of the label (~65%) was at a single site, but two other tryptic peptides were labeled. Treatment of tyrosine hydroxylase from bovine chromaffin cells with a MAP kinase isolated from the same cells resulted in phosphorylation of serines 31 and 40, with a twofold activation (136).

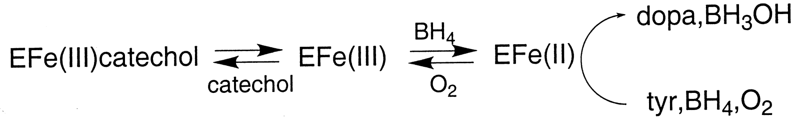

Figure 9 Regulatory mechanism for tyrosine hydroxylase.

Annu. Rev. Biochem. 1999.68:355-381. Downloaded from www.annualreviews.org
Access provided by Universita Degli Studi di Torino - All Libraries on 11/16/20. For personal use only.

Annu. Rev. Biochem. 1999.68:355-381. Downloaded from www.annualreviews.org
Access provided by Universita Degli Studi di Torino - All Libraries on 11/16/20. For personal use only.

There have been a series of studies with the different isoforms of human tyrosine hydroxylase. Because they were done with recombinant enzymes, there is no endogenous phosphate or bound catecholamine to complicate interpretation of the results. As noted above, there are four isoforms of the human enzyme owing to differential splicing, differing in the size of an insert before serine 31 of hTH1. PKA phosphorylates isoforms hTH1, hTH2, and hTH3 on the same residue, equivalent to serine 40 of hTH1 and the rat enzyme. With hTH1 and hTH2, this results in a twofold decrease in the measured value of the K_m for BH_4, no change in the V_{max} value, and a threefold increase in the K_d for dopamine (127). These effects on K_m and V_{max} values are comparable to the results reported for the recombinant rat enzyme. In contrast, phosphorylation of hTH3 by PKA resulted in an increase of 2.5-fold in the values of both the K_m for BH_4 and the K_i for dopamine (129). CaM-PKII phosphorylated both serine 19 and the residue corresponding to serine 40 in isoforms hTH1, hTH2, and hTH3 (127, 129). In addition, the serine corresponding to serine 31 was phosphorylated by CaM-PKII in hTH2 (127). Phosphorylation by CaM-PKII was reported to have no significant effects on the activity of hTH1, hTH2, or hTH3, with the exception of a twofold increase in the K_i value for dopamine with hTH2 (127). However, in a subsequent report an increase in activity upon phosphorylation of hTH1 by CaM-PKII was described; the increase showed a correlation with the extent of phosphorylation of serine 40 by this kinase (130). No further activation was seen upon the addition of 14-3-3 protein. MAP kinase (ERK) phosphorylates all four isoforms at different rates, with hTH3 and hTH4 being good substrates for the kinase, whereas hTH2 was phosphorylated very slowly (130). In hTH1, hTH3, and hTH4, phosphorylation occurred at serine 31 or the analogous residue. In the cases of hTH1 and hTH3, the activity doubled upon incorporation of a stoichiometric amount of phosphate. Finally, MAP kinase-activated kinase 1 phosphorylates all four isoforms on serine 40, whereas MAP kinase-activated kinase 2 phosphorylates all four isoforms on both serine 19 and 40 (130). With both kinases, the activities of all the tyrosine hydroxylase isoforms increased up to threefold; the amount of the increase correlated with the extent of phosphorylation of serine 40.

Tryptophan Hydroxylase

Because of the lack of pure enzyme, the regulatory properties of tryptophan hydroxylase have seen relatively little study. The activity of the enzyme is affected by phosphorylation. The site of phosphorylation by PKA has been identified as serine 58 by site-directed mutagenesis (20, 151). However, the effects of phosphorylation on activity, which have been described to date, have been quite modest. Incubation of rat brain extracts with ATP, $MgCl_2$, and $CaCl_2$ was initially shown by Yamauchi & Fujisawa to increase the activity of tryptophan hydroxylase about twofold (152). The requirement for Ca^{2+} suggested a need for a calcium-dependent kinase. This was confirmed when these authors reported that incubation of a partially pure preparation of the rat brain enzyme with CaM-

Annu. Rev. Biochem. 1999.68:355-381. Downloaded from www.annualreviews.org
Access provided by Universita Degli Studi di Torino - All Libraries on 11/16/20. For personal use only.

PKII increased the activity about twofold (153). The preparation used also contained an activator protein; this activator protein was subsequently identified as a 14-3-3 protein (146, 148), also involved in activation of tyrosine hydroxylase. Direct phosphorylation of tryptophan hydroxylase by CaM-PKII was demonstrated by Ehret et al (153a). In addition PKA has been shown to phosphorylate this enzyme, resulting in twofold activation if the activator protein is present (154). The effects of PKA and CaM-PKII were the same and were not additive, suggesting that the same site is phosphorylated by both. The activation was caused by a 30% decrease in the K_m value for 6-MePH$_4$ and a 50% increase in the V_{max} value. Direct binding of a 14-3-3 protein to tryptophan hydroxylation was demonstrated by Furukawa et al (155). Binding required prior phosphorylation by CaM-PKII. The K_d value for binding of the phosphorylated enzyme to bovine 14-3-3 proteins was measured as 30 nM by Banik et al (156). These data are all consistent with a single phosphorylation site, serine 58, and with an additional requirement for a 14-3-3 protein for activation. However, there have been reports of activation by phosphorylation alone (151, 156).

PERSPECTIVES

Phenylalanine, tyrosine, and tryptophan hydroxylase have attracted a great deal of scientific interest over the years because they catalyze reactions critical for the proper functioning of the central nervous system and because they constitute a self-contained family of monooxygenases with novel properties. In recent years the availability of the recombinant enzymes has allowed significant progress in elucidating the mechanisms, structures, and regulation of these pterin-dependent monooxygenases. The homologous catalytic core of each protein has been identified, and the structures of the catalytic domains of phenylalanine and tyrosine hydroxylase have been determined. This will allow more detailed analyses of the roles of individual amino acid residues in catalysis and stability. Details of the catalytic events are beginning to emerge. However, the specific role of the iron atom and the structure of the hydroxylating intermediate remain elusive. Knowledge of the regulatory properties, critical to understanding the biology of these systems, has progressed unevenly. With phenylalanine hydroxylase, phenylalanine and tetrahydrobiopterin levels interact in determining the enzyme activity; how this is modulated by phosphorylation has not been determined in any quantitative fashion. For tyrosine hydroxylase, detailed analyses of the effects of phosphorylation have been carried out for only one of the several phosphorylation sites. Understanding the regulatory properties of tryptophan hydroxylase has only just begun.

ACKNOWLEDGMENTS

Work described in this review from the author's laboratory is supported in part by NIH grant GM47291.

Annu. Rev. Biochem. 1999.68:355-381. Downloaded from www.annualreviews.org
Access provided by Universita Degli Studi di Torino - All Libraries on 11/16/20. For personal use only.

LITERATURE CITED

1. Letendre CH, Dickens G, Guroff G. 1975. *J. Biol. Chem.* 250:6672–78
2. Nakata H, Yamauchi T, Fujisawa H. 1979. *J. Biol. Chem.* 254:1829–33
3. Letendre CH, Dickens G, Guroff G. 1974. *J. Biol. Chem.* 249:7186–91
4. Lazarus RA, Dietrich RF, Wallick DE, Benkovic SJ. 1981. *Biochemistry* 20: 6834–41
5. Dix TA, Kuhn DM, Benkovic SJ. 1987. *Biochemistry* 26:3354–61
6. Haavik J, Flatmark T. 1987. *Eur. J. Biochem.* 168:21–26
7. Moran GR, Daubner SC, Fitzpatrick PF. 1998. *J. Biol. Chem.* 273:12259–66
8. Dix TA, Bollag GE, Domanico PL, Benkovic SJ. 1985. *Biochemistry* 24: 2955–58
9. Bailey SW, Rebrin I, Boerth SR, Ayling JE. 1995. *J. Am. Chem. Soc.* 117: 10203–11
10. Shiman R. 1985. In *Folates and Pterins*, 2:ed. RL Blakley, SJ Benkovic, 179–249. New York: Wiley
11. Zhao G, Xia T, Song J, Jensen RA. 1994. *Proc. Natl. Acad. Sci. USA* 91:1366–70
12. Onishi A, Liotta LJ, Benkovic SJ. 1991. *J. Biol. Chem.* 266:18454–59
13. Chen D, Frey PA. 1998. *J. Biol. Chem.* 273:25594–601
14. Grima B, Lamouroux A, Boni C, Julien J–F, Javoy–Agid F, Mallet J. 1987. *Nature* 326:707–11
15. Kaneda N, Kobayashi K, Ichinose H, Kishi F, Nakazawa A, et al. 1987. *Biochem. Biophys. Res. Commun.* 147: 971–75
16. O'Malley KL, Anhalt MJ, Martin BM, Kelsoe JR, Winfield SL, Ginns EI. 1987. *Biochemistry* 26:6910–14
17. Grenett HE, Ledley FD, Reed LL, Woo SLC. 1987. *Proc. Natl. Acad. Sci. USA* 84: 5530–34
18. Fisher DB, Kaufman S. 1973. *J. Biol. Chem.* 248:4345–53
19. Dickson PW, Jennings IG, Cotton RGH. 1994. *J. Biol. Chem.* 269:20369–75
20. Kumer SC, Mockus SM, Rucker PJ, Vrana KE. 1997. *J. Neurochem.* 69:1738–45
21. Ribeiro P, Wang Y, Citron BA, Kaufman S. 1993. *J. Mol. Neurosci.* 4:125–39
22. Iwaki M, Phillips RS, Kaufman S. 1986. *J. Biol. Chem.* 261:2051–56
23. Okuno S, Fujisawa H. 1982. *Eur. J. Biochem.* 122:49–55
24. Nakata H, Fujisawa H. 1982. *Eur. J. Biochem.* 124:595–601
25. Kappock TJ, Harkins PC, Friedenberg S, Caradonna JP. 1995. *J. Biol. Chem.* 270: 30532–44
26. Knappskog PM, Flatmark T, Aarden JM, Haavik J, Martinez A. 1996. *Eur. J. Biochem.* 242:813–21
27. Daubner SC, Lohse DL, Fitzpatrick PF. 1993. *Protein Sci.* 2:1452–60
28. D'Sa C, Arthur R Jr, Kuhn D. 1996. *J. Neurochem.* 67:917–26
29. Gibbs BS, Wojchowski D, Benkovic SJ. 1993. *J. Biol. Chem.* 268:8046–52
30. Lohse DL, Fitzpatrick PF. 1993. *Biochem. Biophys. Res. Commun.* 197:1543–48
31. Liu X, Vrana KE. 1991. *Neurochem. Int.* 18:27–31
32. Erlandsen H, Fusetti F, Martinez A, Hough E, Flatmark T, Stevens RC. 1997. *Nat. Struct. Biol.* 4:995–1000
33. Goodwill KE, Sabatier C, Marks C, Raag R, Fitzpatrick PF, Stevens RC. 1997. *Nat. Struct. Biol.* 4:578–85
34. Martinez A, Abeygunawardana C, Haavik J, Flatmark T, Mildvan AS. 1993. *Biochemistry* 32:6381–90
35. Martinez A, Abeygunawardana C, Haavik J, Flatmark T, Mildvan AS. 1993. In *Chemistry and Biology of Pteridines and Folates*, ed. JE Ayling, HG Nair, CM Baugh, pp. 77–80. New York: Plenum
36. Martinez A, Andersson KK, Haavik J, Flatmark T. 1991. *Eur. J. Biochem.* 198: 675–82
37. Jennings IG, Kemp BE, Cotton RGH. 1991. *Proc. Natl. Acad. Sci. USA* 88: 5734–38

Annu. Rev. Biochem. 1999.68:355-381. Downloaded from www.annualreviews.org
Access provided by Universita Degli Studi di Torino - All Libraries on 11/16/20. For personal use only.

38. Ramsey AJ, Daubner SC, Ehrlich JI, Fitzpatrick PF. 1995. *Protein Sci.* 4:2082–86
39. Fisher DB, Kirkwood R, Kaufman S. 1972. *J. Biol. Chem.* 247:5161–67
40. Hoeldtke R, Kaufman S. 1977. *J. Biol. Chem.* 252:3160–69
41. Gottschall DW, Dietrich RF, Benkovic SJ. 1982. *J. Biol. Chem.* 257:845–49
42. Fitzpatrick PF. 1989. *Biochem. Biophys. Res. Commun.* 161:211–15
43. Haavik J, Andersson KK, Petersson L, Flatmark T. 1988. *Biochim. Biophys. Acta* 953:142–56
44. Marota JJA, Shiman R. 1984. *Biochemistry* 23:1303–11
45. Wallick DE, Bloom LM, Gaffney BJ, Benkovic SJ. 1984. *Biochemistry* 23: 1295–302
46. Ramsey AJ, Hillas PJ, Fitzpatrick PF. 1996. *J. Biol. Chem.* 271:24395–400
47. Shiman R, Gray DW, Hill MA. 1994. *J. Biol. Chem.* 269:24637–46
48. Haavik J, Le Bourdelles B, Martinez A, Flatmark T, Mallet J. 1991. *Eur. J. Biochem.* 19:371–78
49. Haavik J, Martinez A, Olafsdottir S, Mallet J, Flatmark T. 1992. *Eur. J. Biochem.* 210:23–31
50. Pember SO, Villafranca JJ, Benkovic SJ. 1986. *Biochemistry* 25:6611–19
51. Carr RT, Benkovic SJ. 1993. *Biochemistry* 32:14132–38
52. Carr RT, Balasubramanian S, Hawkins PCD, Benkovic SJ. 1995. *Biochemistry* 34:7525–32
53. Pember SO, Johnson KA, Villafranca JJ, Benkovic SJ. 1989. *Biochemistry* 28: 2124–30
54. Fitzpatrick PF. 1991. *Biochemistry* 30: 3658–62
55. Storm CB, Kaufman S. 1968. *Biochem. Biophys. Res. Commun.* 32:788–80
56. Ellenbogen L, Taylor RJ, Brundage GB. 1965. *Biochem. Biophys. Res. Commun.* 19:708–15
57. Tong JH, Kaufman S. 1975. *J. Biol. Chem.* 250:4152–58
58. Bailey SW, Dillard SB, Thomas KB, Ayling JE. 1989. *Biochemistry* 28: 494– 504
59. Ayling JE, Boehm GR, Textor SC, Pirson RA. 1973. *Biochemistry* 12:2045–51
60. Nagatsu T, Mizutani K, Nagatsu I, Matsuura S, Sugimoto T. 1972. *Biochem. Pharmacol.* 21:1945–53
61. Bailey SW, Ayling JE. 1978. *Biochem. Biophys. Res. Commun.* 85:1614–21
62. Kaufman S. 1979. *J. Biol. Chem.* 254: 5150–54
63. Pike DC, Hora MT, Bailey SW, Ayling JE. 1986. *Biochemistry* 25:4762–71
64. Daubner SC, Hillas PJ, Fitzpatrick PF. 1997. *Biochemistry* 36:11574–82
65. Kaufman S, Mason K. 1982. *J. Biol. Chem.* 257:14667–78
66. Davis MD, Kaufman S. 1988. *J. Biol. Chem.* 263:17312–16
67. Fukami MH, Haavik J, Flatmark T. 1990. *Biochem. J.* 268:525–28
68. Ribeiro P, Pigeon D, Kaufman S. 1991. *J. Biol. Chem.* 266:16207–11
69. Fitzpatrick PF. 1994. *J. Am. Chem. Soc.* 116:1133–34
70. Tong JH, D'Iorio A, Benoiton NL. 1971. *Biochem. Biophys. Res. Commun.* 44: 229–36
71. Fitzpatrick PF. 1991. *Biochemistry* 30: 6386–91
72. Ledley FD, DiLella AG, Kwok SCM, Woo SLC. 1985. *Biochemistry* 24: 3389–94
73. Meyer MM, Fitzpatrick PF. 1992. *Neurochem. Int.* 21:191–96
74. Guroff G, Kondo K, Daly J. 1966. *Biochem. Biophys. Res. Commun.* 25: 622–28
75. Siegmund H–U, Kaufman S. 1991. *J. Biol. Chem.* 266:2903–10
76. Daly J, Guroff G. 1968. *Arch. Biochem. Biophys.* 125:136–41
77. Miller RJ, Benkovic SJ. 1988. *Biochemistry* 27:3658–63
78. Hillas PJ, Fitzpatrick PF. 1996. *Biochemistry* 35:6969–75
79. Ramsey AJ, Fitzpatrick PF. 1998. *Biochemistry* 37:8980–86
80. Cox DD, Benkovic SJ, Bloom LM, Bradley FC, Nelson MJ, et al. 1988. *J. Am. Chem. Soc.* 110:2026–32

Annu. Rev. Biochem. 1999.68:355-381. Downloaded from www.annualreviews.org
Access provided by Universita Degli Studi di Torino - All Libraries on 11/16/20. For personal use only.

81. Michaud–Soret I, Andersson KK, Que L Jr, Haavik J. 1995. *Biochemistry* 34:5504–10
82. Johansen PA, Wolf WA, Kuhn DM. 1991. *Biochem. Pharmacol.* 41:625–28
83. Andersson KK, Cox DD, Que L Jr, Flatmark T, Haavik J. 1988. *J. Biol. Chem.* 263:18621–26
84. Andersson KK, Vassort C, Brennan BA, Que L Jr, Haavik J, et al. 1992. *Biochem. J.* 284:687–95
85. Abita J–P, Parniak M, Kaufman S. 1984. *J. Biol. Chem.* 259:14560–66
86. Daly J, Levitt M, Guroff G, Udenfriend S. 1968. *Arch. Biochem. Biophys.* 126:593–98
87. Eberlein G, Bruice TC, Lazarus RA, Henrie R, Benkovic SJ. 1984. *J. Am. Chem. Soc.* 106:7916–24
88. Massey V. 1994. *J. Biol. Chem.* 269:22459–62
89. Eberlein G, Bruice TC. 1983. *J. Am. Chem. Soc.* 105:6685–97
90. Francisco WA, Tian G, Fitzpatrick PF, Klinman JP. 1998. *J. Am. Chem. Soc.* 120:4057–62
91. Sono M, Roach MP, Coulter ED, Dawson JH. 1996. *Chem. Rev.* 96:2841–88
92. Spector T, Massey V. 1972. *J. Biol. Chem.* 247:4679–87
93. Dix TA, Benkovic SJ. 1985. *Biochemistry* 24:5839–46
94. Davis MD, Kaufman S. 1989. *J. Biol. Chem.* 264:8585–96
95. Hamilton GA. 1971. In *Progress in Biorganic Chemistry,* ed. ET Kaiser, FJ Kezdy, 1:83–157. New York: Wiley
96. Bailey SW, Ayling JE. 1980. *J. Biol. Chem.* 255:7774–81
97. Bailey SW, Weintraub ST, Hamilton SM, Ayling JE. 1982. *J. Biol. Chem.* 257:8253–60
98. Benkovic S, Wallick D, Bloom L, Gaffney BJ, Domanico P, et al. 1985. *Biochem. Soc. Trans.* 13:436–38
99. Renson JD, Daly J, Weissbach H, Witkop B, Udenfriend S. 1966. *Biochem. Biophys. Res. Commun.* 25:504–13
100. Guroff G, Daly J. 1967. *Arch. Biochem. Biophys.* 122:212–17
101. Guroff G, Daly JW, Jerina DM, Renson J, Witkop B, Udenfriend S. 1967. *Science* 157:1524–30
102. Jerina DM, Daly JW. 1974. *Science* 185:573–82
103. Parniak MA, Kaufman S. 1981. *J. Biol. Chem.* 256:6876–82
104. Philips RS, Iwaki M, Kaufman S. 1983. *Biochem. Biophys. Res. Commun.* 110:919–25
105. Daubner SC, Hillas PJ, Fitzpatrick PF. 1997. *Arch. Biochem. Biophys.* 348:295–302
106. Gibbs BS, Benkovic SJ. 1991. *Biochemistry* 30:6795–802
107. Shiman R, Gray DW. 1980. *J. Biol. Chem.* 255:4793–800
108. Shiman R, Xia T, Hill MA, Gray DW. 1994. *J. Biol. Chem.* 269:24647–56
109. Xia T, Gray DW, Shiman R. 1994. *J. Biol. Chem.* 269:24657–65
110. Mitnaul LJ, Shiman R. 1995. *Proc. Natl. Acad. Sci. USA* 92:885–89
111. Shiman R, Jones SH, Gray DW. 1990. *J. Biol. Chem.* 265:11633–42
112. Shiman R, Gray DW, Pater A. 1979. *J. Biol. Chem.* 254:11300–6
113. Koizumi S, Tanaka F, Kaneda N, Kano K, Nagatsu T. 1988. *Biochemistry* 27:640–46
114. Wretborn M, Humble E, Ragnarsson U, Engstrom L. 1980. *Biochem. Biophys. Res. Commun.* 93:403–8
115. Abita J–P, Milstien S, Chang N, Kaufman S. 1976. *J. Biol. Chem.* 251:5310–14
116. Kowlessur D, Yang X–J, Kaufman S. 1995. *Proc. Natl. Acad. Sci. USA* 92:4743–47
117. Doskeland AP, Martinez A, Knappskog PM, Flatmark T. 1996. *Biochem. J.* 313:409–14
118. Doskeland AP, Schworer CM, Doskeland SO, Chrisman TD, Soderling TR, et al. 1984. *Eur. J. Biochem.* 145:31–37

Annu. Rev. Biochem. 1999.68:355-381. Downloaded from www.annualreviews.org
Access provided by Universita Degli Studi di Torino - All Libraries on 11/16/20. For personal use only.

119. Shiman R, Mortimore GE, Schworer CM, Gray DW. 1982. *J. Biol. Chem.* 257: 11213–16
120. Doskeland AP, Doskeland SO, Ogreid D, Flatmark T. 1984. *J. Biol. Chem.* 259: 11242–48
121. Doskeland AP, Haavik J, Flatmark T, Doskeland SO. 1987. *Biochem. J.* 242: 867–74
122. Zigmond RE, Schwarzschild MA, Rittenhouse AR. 1989. *Annu. Rev. Neurosci.* 12: 415–61
123. Haycock JW. 1993. *Neurochem. Res.* 18: 15–26
124. Haycock JW. 1990. *J. Biol. Chem.* 265: 11682–91
125. Campbell DG, Hardie DG, Vulliet PR. 1986. *J. Biol. Chem.* 261:10489–92
126. Deleted in proof
127. Le Bourdellès B, Horellou P, Le Caer J–P, Denèfle P, Latta M, et al. 1991. *J. Biol. Chem.* 266:17124–30
128. Pfleiderer W, Zondler H, Mengel R. 1970. *Liebigs Ann. Chem.* 741:64–78
129. Alterio J, Ravassard P, Haavik J, Le Caer J, Biguet N, et al. 1998. *J. Biol. Chem.* 273:10196–201
130. Sutherland C, Alterio J, Campbell DG, Le Bourdelles B, Mallet J, et al. 1993. *Eur. J. Biochem.* 217:715–22
131. Mitchell JP, Hardie DG, Vulliet PR. 1998. *J. Biol. Chem.* 265:22358–64
132. Haycock JW, Haycock DA. 1991. *J. Biol. Chem.* 266:5650–57
133. Griffith LC, Schulman H. 1988. *J. Biol. Chem.* 263:9542–49
134. Waymire JC, Johnston JP, Hummer–Lickteig K, Lloyd A, Vigny A, Craviso GL. 1988. *J. Biol. Chem.* 263:12439–47
135. Haycock JW, Ahn NG, Cobb MH, Krebs EG. 1992. *Proc. Natl. Acad. Sci. USA* 89: 2365–69
136. Halloran SM, Vulliet PR. 1994. *J. Biol. Chem.* 269:30960–65
137. Nelson TJ, Kaufman S. 1987. *Arch. Biochem. Biophys.* 257:69–84
138. Joh TH, Park DH, Reis DJ. 1978. *Proc. Natl. Acad. Sci. USA* 75:4744–48
139. Vulliet PR, Langan TA, Weiner N. 1980. *Proc. Natl. Acad. Sci. USA* 77:92–96
140. Lazar MA, Lockfeld AJ, Truscott RJW, Barchas JD. 1982. *J. Neurochem.* 39: 409–22
141. Andersson KK, Haavik J, Martinez A, Flatmark T, Petersson L. 1989. *FEBS Lett.* 258:9–12
142. Haavik J, Martinez A, Flatmark T. 1990. *FEBS Lett.* 262:363–65
143. Daubner SC, Lauriano C, Haycock JW, Fitzpatrick PF. 1992. *J. Biol. Chem.* 267: 12639–46
144. Vulliet PR, Woodgett JR, Cohen P. 1984. *J. Biol. Chem.* 259:13680–83
145. Vulliet PR, Woodgett JR, Ferrari S, Hardie DG. 1985. *FEBS Lett.* 182: 335–39
146. Yamauchi T, Nakata H, Fujisawa H. 1981. *J. Biol. Chem.* 256:5404–9
147. Atkinson J, Richtand N, Schworer C, Kuczenski R, Soderling T. 1987. *J. Neurochem.* 49:1241–49
148. Ichimura T, Isobe T, Okuyama T, Yamauchi T, Fujisawa H. 1987. *FEBS Lett.* 219:79–82
149. Ichimura T, Isobe T, Okuyama T, Takahashi N, Araki K, et al. 1988. *Proc. Natl. Acad. Sci. USA* 85:7084–88
150. Aitken A. 1996. *Trends Cell Biol.* 6: 341–47
151. Kuhn DM, Arthur R Jr, States JC. 1997. *J. Neurochem.* 68:2220–23
152. Yamauchi T, Fujisawa H. 1979. *Arch. Biochem. Biophys.* 198:219–26
153. Yamauchi T, Fujisawa H. 1983. *Eur. J. Biochem.* 132:15–21
153a. Ehret M, Cash CD, Hamon M, Maitre M. 1989. *J. Neurochem.* 52:1886–91
154. Makita Y, Okuno S, Fujisawa H. 1990. *FEBS Lett.* 268:185–88
155. Furukawa Y, Ikuta N, Omata S, Yamauchi T, Isobe T, Ichimura T. 1993. *Biochem. Biophys. Res. Commun.* 194: 144–49
156 Banik U, Wagner PD, Kaufman S. 1997. *J. Biol. Chem.* 272:26219–25
