# Supplementary material for: 5-Hydroxytryptophan (5-HTP): Natural Occurrence, Analysis, Biosynthesis, Biotechnology, Physiology and Toxicology
Source: Int J Mol Sci. 2020 Dec 26;22(1):181. doi: 10.3390/ijms22010181 (PMC7796270; doi:10.3390/ijms22010181)
Supplement: Supplementary file 1 [file ijms-22-00181-s001.zip › 5-HTP.Data/PDF/1562169351/Eser-2007-Direct-spectroscopic-evidence-for-a.pdf]

J|A|C|S
COMMUNICATIONS

Published on Web 08/23/2007

Direct Spectroscopic Evidence for a High-Spin Fe(IV) Intermediate in Tyrosine Hydroxylase

Bekir E. Eser,‡ Eric W. Barr,§ Patrick A. Frantom,† Lana Saleh,§ J. Martin Bollinger, Jr.,*,§,⊥ Carsten Krebs,*,§,⊥ and Paul F. Fitzpatrick*,‡,†

Departments of Chemistry and of Biochemistry and Biophysics, Texas A&M University, College Station, Texas 77843, and Departments of Biochemistry and Molecular Biology and of Chemistry, The Pennsylvania State University, University Park, Pennsylvania 16802

Received June 18, 2007; Email: jmb21@psu.edu; ckrebs@psu.edu; fitzpat@tamu.edu

Downloaded via UNIV DEGLI STUDI DI TORINO on November 16, 2020 at 16:27:06 (UTC).
See https://pubs.acs.org/sharingguidelines for options on how to legitimately share published articles.

Tyrosine hydroxylase (TyrH[1]), the key enzyme in the biosynthesis of catecholamine neurotransmitters, is one of three members of the aromatic amino acid hydroxylase enzyme family.[2,3] The enzyme is found in the brain and adrenal gland where it catalyzes the conversion of L-tyrosine to L-DOPA. The other members of the family are phenylalanine hydroxylase, which catabolizes excess phenylalanine to tyrosine, and tryptophan hydroxylase, which catalyzes the rate-limiting step in the biosynthesis of the neurotransmitter serotonin. All three enzymes have a mononuclear nonheme iron, coordinated by the common His_2-Glu facial triad motif,[4,5] and use a tetrahydropterin to activate dioxygen for hydroxylation of the aromatic side chains of their corresponding amino acid substrates.[2,3] In the proposed mechanism[6−8] (Scheme 1), oxygen reacts with ferrous iron and tetrahydropterin to produce a Fe(IV)O (ferryl) hydroxylating intermediate and 4a-hydroxypterin (4a-$HOPH_3$). Then, through an electrophilic aromatic substitution, the ferryl species reacts with the aromatic side chain of the tyrosine substrate (Tyr) to form the product dihydroxyphenylalanine (DOPA). To date there has been no direct evidence for this ferryl species. Here, we report the detection of an Fe(IV) intermediate, which is likely to be the proposed ferryl species, in the TyrH reaction by the use of rapid reaction methods.

Scheme 1

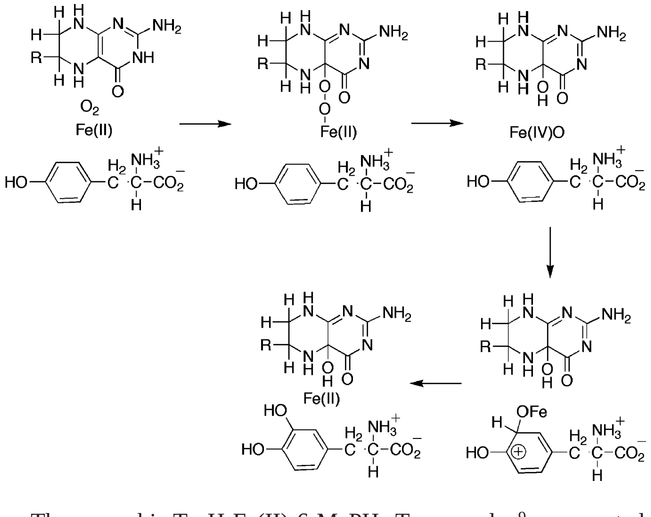

The anaerobic TyrH·Fe(II)·6-$MePH_4$·Tyr complex[9] was reacted with oxygen and quenched by rapid-freeze at time points from 20 to 390 ms.[10] Figure 1 (left panel) shows representative Mössbauer spectra of the samples from such a time course. The spectrum of the reactant complex reveals the presence of two broad lines with parameters typical of high-spin Fe(II). The asymmetry suggests the presence of at least two distinct Fe(II) complexes. A new line at ∼0.9 mm/s is observed in the spectra of samples in which the reactant complex was exposed to oxygen for either 20 or 100 ms, but it is not detected in the spectrum of a sample reacted for 390 ms. Thus, this peak is associated with a reaction intermediate which exhibits a quadrupole doublet in a weak external magnetic field. The low-energy line of this quadrupole doublet overlaps with the low-energy line of the Fe(II). The features of the intermediate are similar to those observed for Fe(IV) intermediates in other mononuclear nonheme enzymes.[11,12]

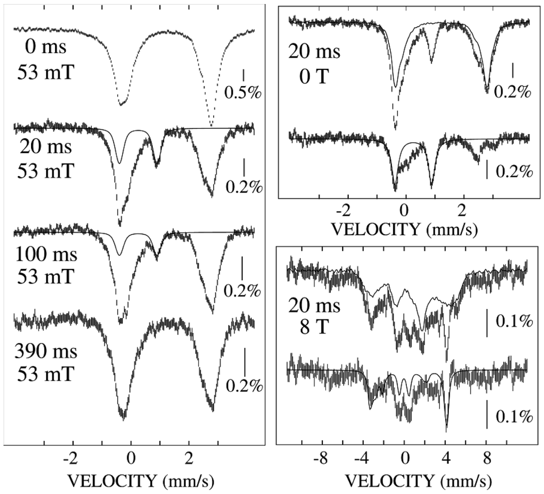

Figure 1. Mössbauer spectra at 4.2-K of the reaction at 5 °C of the TyrH·Fe(II)·6-$MePH_4$·Tyr complex (2.15 mM TyrH, 1.95 mM Fe(II), 3.7 mM 6-$MePH_4$ and 3.7 mM Tyr in 200 mM Hepes, 10% glycerol, 0.1 M KCl at pH 7.5) with 1.9 mM oxygen-containing buffer in a ratio of 1:2. Reaction times and magnetic field strengths are as indicated. Left panel: spectra (hashed marks) at various reaction times. The solid lines are quadrupole doublet simulations of the spectra of the Fe(IV) intermediate ($\delta = 0.25$ mm/s and $\Delta E_Q = 1.27$ mm/s). Right panel: deconvolution of the spectrum of the 20-ms sample in zero-field (top panel) and an 8-T field (bottom panel). The spectrum of the anaerobic control scaled to 60% of the total intensity is shown as a solid line overlaid with the raw data. Difference spectra of the Fe(IV) intermediate (24% intensity) were simulated with the following spin Hamiltonian parameters: $S = 2$, $D = 12.5$ cm^{-1}, $E/D = 0.05$, $\delta = 0.25$ mm/s, $\Delta E_Q = -1.27$ mm/s, $\eta = -0.5$, $\mathbf{A}/g_N\beta_N = (-18.0, -18.0, -31.0)$ T.

To characterize this intermediate further, we recorded spectra of the 20-ms sample (this sample contains a maximum amount of the intermediate, 24% of total Fe) without an applied field (Figure 1, top right panel) and with an 8-T applied field (bottom right panel). The spectrum of the 20-ms sample is shown as the top spectrum in

‡ Department of Chemistry, Texas A&M University.
† Department of Biochemistry and Biophysics, Texas A&M University.
§ Department of Biochemistry and Molecular Biology, The Pennsylvania State University.
⊥ Department of Chemistry, The Pennsylvania State University.

10.1021/ja074446s CCC: $37.00 © 2007 American Chemical Society

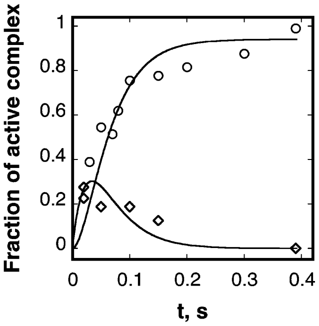

Figure 2. Comparison of time courses for Fe(IV)O formation and decay (diamonds) and for DOPA formation (circles). DOPA was quantified by rapid-quench of the reaction at 5 °C of the complex of 500 μM TyrH, 480 μM Fe(II), 1 mM Tyr, and 2 mM 6-MePH$_4$ with an equal volume of 1.9 mM oxygen-containing buffer. The lines are simulations using the mechanism of Scheme 2 and values of k_1 and k_2 of 24 and 35 s^{-1}, respectively, assuming that 80% of the enzyme complex is active.

each panel (vertical bars). Removal of the spectral contribution of the starting material (55% of the total intensity, shown as a solid line in the top spectra) results in the spectra depicted as vertical bars (lower spectrum in each panel). The zero-field spectrum reveals the position of the low-energy line and allows the isomer shift (δ) and quadrupole splitting (ΔE_Q) of the intermediate to be determined: $\delta = 0.25$ mm/s and $\Delta E_Q = 1.27$ mm/s. These parameters are similar to those experimentally observed[10−14] and theoretically predicted[15,16] for nonheme ferryl intermediates and strongly suggest the presence of such a complex in TyrH.[17] In addition, the spectrum reveals two broad lines at 0 and 2.4 mm/s. These features are associated with a high-spin Fe(II) complex formed during the reaction.

The 8-T spectrum provides further insight into the electronic structure of the Fe(IV) intermediate. In particular, the sharp line at 4 mm/s is associated with the intermediate and not the reactant complex. The spectrum resulting after removal of the 55%-contribution of the reactant complex reflects the Fe(IV) intermediate (24%) and the new high-spin Fe(II) complex (20%). The contribution of the Fe(IV) complex was simulated according to the spin Hamiltonian formalism with parameters typical of high-spin Fe(IV) complexes.[10−15,18]

The presence of a Fe(IV) complex is also supported by the EPR spectra of a 20-ms sample (prepared under identical conditions) recorded before and after exposure to γ-radiation at 77 K (cryoreduction). The hallmark features of a high-spin Fe(III) complex at $g = 4.3$ become much more intense, suggesting that a Fe(III) complex is formed during cryoreduction (Figure S1). Similar results have been observed for a nonheme Fe(IV)O intermediate.[11]

An additional rapid-reaction experiment was performed to establish the kinetic competency as the hydroxylating intermediate of the Fe(IV) complex detected by Mössbauer spectroscopy. The TyrH•Fe(II)•6-MePH$_4$•Tyr complex was reacted with oxygen in the same way as for the Mössbauer study, except that the reaction was quenched with acid and the amount of DOPA quantified (Figure 2).[19] The data could be fit reasonably well as a single exponential increase with a rate constant of 15 ± 2 s^{-1}. This is significantly faster than the k_{cat} value at this temperature. The kinetics of formation of both DOPA and the Fe(IV) species were then analyzed[20] according to the mechanism of Scheme 2, with the single

Scheme 2

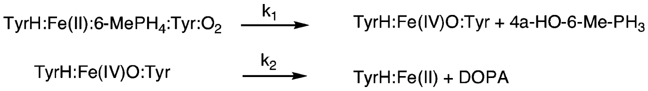

rate constant for DOPA formation as an initial estimate. In this kinetic mechanism, the first step is the concomitant formation of Fe(IV)O and 4a-hydroxypterin. Fe(IV)O and tyrosine then react to form the product DOPA. Both time courses were well fit with values for the rate constants k_1 and k_2 of 24 and 35 s^{-1}, respectively, consistent with the Fe(IV) intermediate being the hydroxylating species.

To conclude, this work shows direct spectroscopic evidence for a high-spin Fe(IV) species, presumably the postulated Fe(IV)O, as the hydroxylating intermediate in the reaction catalyzed by TyrH. This is the first example for this family of enzymes as well as the first for a mononuclear nonheme enzyme which catalyzes aromatic hydroxylation.

Acknowledgment. This work was supported by grants from the National Institutes of Health (Grant GM-47291 to P.F.F.), The Welch Foundation (Grant A1245 to P.F.F.), the donors of the Petroleum Research Fund (Grant 41170-G3 to C.K.), the National Science Foundation (Grant NSF-642058 to J.M.B. and C.K.), the Beckman Foundation (Young Investigator Award to C.K.), and the Dreyfus Foundation (Teacher Scholar Award to C.K.). We thank Candace C. Davison for help with the cryoreduction experiments.

Supporting Information Available: EPR spectra of a 20-ms sample recorded before and after cryoreduction and the spin Hamiltonian used for analysis of the Mössbauer spectra. This material is available free of charge via the Internet at http://pubs.acs.org.

References

(1) Abbreviations used: TyrH, tyrosine hydroxylase; Tyr, tyrosine; DOPA, dihydroxyphenylalanine; 6-MePH$_4$, 6-methyl tetrahydropterin; 4a-HOPH$_3$, 4a-hydroxypterin.
(2) Fitzpatrick, P. F. *Ann. Rev. Biochem.* **1999**, *68*, 355−381.
(3) Fitzpatrick, P. F. In *Advances in Enzymology and Related Areas of Molecular Biology*; Purich, D. L., Ed.; John Wiley & Sons: New York, 2000; Vol. 74, pp 235−294.
(4) Que, L., Jr. *Nat. Struct. Biol.* **2000**, *7*, 182−184.
(5) Goodwill, K. E .; Sabatier, C.; Marks, C.; Raag, R.; Fitzpatrick, P. F.; Stevens, R. C. *Nat. Struct. Biol.* **1997**, *4*, 578−585.
(6) Fitzpatrick, P. F. *Biochemistry* **2003**, *42*, 14083−14091.
(7) Hillas, P. J.; Fitzpatrick, P. F. *Biochemistry* **1996**, *35*, 6969−6975.
(8) Moran, G. R.; Derecskei-Kovacs, A.; Hillas, P. J.; Fitzpatrick, P. F. *J. Am. Chem. Soc.* **2000**, *122*, 4535−4541.
(9) Frantom, P. A.; Seravalli, J.; Ragsdale, S. W.; Fitzpatrick, P. F. *Biochemistry* **2006**, *45*, 2372−2379.
(10) Krebs, C.; Price, J. C.; Baldwin, J.; Saleh, L.; Green, M. T.; Bollinger, J. M., Jr. *Inorg. Chem.* **2005**, *44*, 742−757.
(11) Price, J. C.; Barr, E. W.; Tirupati, B.; Bollinger, J. M., Jr.; Krebs, C. *Biochemistry* **2003**, *42*, 7497−7508.
(12) Hoffart, L. M.; Barr, E. W.; Guyer, R. B.; Bollinger, J. M., Jr.; Krebs, C. *Proc. Natl. Acad. Sci, U.S.A.* **2006**, *103*, 14738−14743.
(13) Galonić, D. P.; Barr, E. W.; Walsh, C. T.; Bollinger, J. M., Jr.; Krebs, C. *Nature Chem. Biol.* **2007**, *3*, 113−116.
(14) Krebs, C.; Galonić Fujimori, D.; Walsh, C. T.; Bollinger, J. M., Jr. *Acc. Chem. Res.* **2007**, *40*, 484−492.
(15) Sinnecker, S.; Svensen, N.; Barr, E. W.; Ye, S.; Bollinger, J. M., Jr.; Neese, F.; Krebs, C. *J. Am. Chem. Soc.* **2007**, *129*, 6168−6179.
(16) Neese, F. *J. Inorg. Biochem.* **2006**, *100*, 716−727.
(17) While $\delta = 0.25$ mm/s would also be consistent with a low-spin Fe(III) complex, such a complex would not be expected to exhibit a quadrupole doublet in low fields because of its $S = 1/2$ ground state.
(18) Pestovsky, O.; Stoian, S.; Bominaar, E. L.; Shan, X.; Münck, E.; Que, L., Jr.; Bakac, A. *Angew. Chem., Int. Ed.* **2005**, *44*, 6871−6874.
(19) Fitzpatrick, P. F. *Biochemistry* **1991**, *30*, 3658−3662.
(20) Kuzmic, P. *Anal. Biochem.* **1996**, *237*, 260−273.

JA074446S
