# Supplementary material for: 5-Hydroxytryptophan (5-HTP): Natural Occurrence, Analysis, Biosynthesis, Biotechnology, Physiology and Toxicology
Source: Int J Mol Sci. 2020 Dec 26;22(1):181. doi: 10.3390/ijms22010181 (PMC7796270; doi:10.3390/ijms22010181)
Supplement: Supplementary file 1 [file ijms-22-00181-s001.zip › 5-HTP.Data/PDF/1571752588/Chen-2017-Sensitive-detection-of-l--hydroxytr.pdf]

Analytical Biochemistry 526 (2017) 58–65

Contents lists available at ScienceDirect

Analytical Biochemistry

journal homepage: www.elsevier.com/locate/yabio

Sensitive detection of L-5-hydroxytryptophan based on molecularly imprinted polymers with graphene amplification

Lu Chen, Hui-Ting Lian, Xiang-Ying Sun, Bin Liu *

College of Materials Science and Engineering, Huaqiao University, Xiamen, 361021, People's Republic of China

ARTICLE INFO

Article history:
Received 20 December 2016
Received in revised form
5 March 2017
Accepted 18 March 2017
Available online 19 March 2017

Keywords:
L-5-Hydroxytryptophan
Graphene
Molecularly imprinting
Chitosan

ABSTRACT

A novel electrochemical sensor was presented for the determination of L-5-hydroxytryptophan (L-5-HTP) based on a graphene-chitosan molecularly imprinted film modified on the surface of glassy carbon electrode (GR-MIP/GCE). The morphology and composition of the imprinted film were observed in field emission scanning electron microscopy (FESEM), raman spectroscopy and fourier transform infrared (FTIR). The properties of the sensor were evaluated by electrochemical techniques. Under the optimal conditions, the peak currents of L-5-HIP were found to be linear in the concentration range of 0.05–7.0 μM, while the sensor also exhibited great features such as low detection limit of 6.0 nM (S/N = 3), superb selectivity against the structural analogues, good antidisturbance ability among coexisting components, excellent repeatability and stability. Moreover, the proposed method had been applied to the detection of L-5-HTP in human blood serum with a satisfactory recoveries ranging from 90.6% to 105.6%.

© 2017 Published by Elsevier Inc.

Introduction

L-5-Hydroxytryptophan (L-5-HTP) is a naturally occurring aromatic amino acid, which is an intermediate precursor in the biosynthesis of an important neurotransmitter, Serotonin (5-HT) [1–3]. Studies in past research have proved that L-5-HTP can easily pass through the blood–brain barrier without requiring transport molecules, and then efficiently converted into serotonin in the central nervous system (CNS), leading to the abnormally increased serotonin level in the brain and CNS [4–6]. The estimation of L-5-HTP is thus directly related to some clinically important hormones in the living body, such as prolactin and serum corticosterone. Low levels of L-5-HTP are related to several pathological conditions such as depression, migraine, bipolar disorder, schizophrenia and carcinoid syndrome [7], its excessive use may result in blood abnormalities (eosinophilia), sexual problems, heart burns, nausea [5] etc. As a result, monitoring the accurate levels of L-5-HTP in the body is of great significance for the early diagnosis of related diseases.

Methods for the quantification of L-5-HTP mainly depend on ultraviolet visible spectroscopy [1], chromatography [8], capillary electrophoresis [9,10]. However, these methods also suffer from high cost, time-consuming and complicated operation. Electrochemical sensors have attracted much attention due to their characteristics of high sensitivity and miniaturization [7,11] and they are expected to overcome the above shortcomings. Various types of the surface modified electrodes have been developed to determine 5-HTP such as CNSs modified GCE [7], Ru^{II}terpyridine-doped composite electrode [12], and gold modified pencil graphite electrode [13] etc. But the sensitivity and selectivity of them are limited. Recently, studies have demonstrated that Graphene (GR) can be used as modified material for the electrode surface to conduct targets analysis because of its large surface area, high mechanical strength and electrical conductivity [14–16]. Furthermore, owing to its high specificity and selectivity to the templates, molecular imprinting technique (MIT) has been widely utilized for the determination of bio-samples [17,18]. Meanwhile, to the best of our knowledge, the MIP based sensor hasn't been reported in the literature. Thus, the attempt to develop a highly sensitive and selective sensor for the analysis of L-5-HTP in biological samples has been made.

Chitosan (CS), a natural polysaccharide polymer, has been applied for electrode fabrication due to its good film forming property [19]. In this work, graphene doped with CS matrix was used to fabricate a desirable film to improve sensitivity. To achieve excellent selectivity, MIT was employed and L-5-HTP was used as

* Corresponding author.
E-mail address: bliu@hqu.edu.cn (B. Liu).

http://dx.doi.org/10.1016/j.ab.2017.03.017
0003-2697/© 2017 Published by Elsevier Inc.

template molecule. Based on this, the molecularly imprinted film was successfully synthesized on the glassy carbon electrode (GCE) via constant potential electrodeposition of CS. The results reveal that the proposed method was validated in terms of the linearity, sensitivity, repeatability, and accuracy, and it was used for analysis of real serum samples with a satisfactory recovery. Thus, we believe this developed sensor is useful for disease-related biomarkers assay that will provide a prospect in clinical diagnosis and medical investigation.

Experimental

Reagents

GR were ordered from Xfnano (Nanjing, China). 5-Hydroxy-L-tryptophan, 5-hydroxytryptamine, dopamine hydrochloride (DA), L-Tyrosine (L-tyr), L-tryptophan (L-try), D-tryptophan (D-try), L-proline (L-Pro), L-histidine (L-His), Epinephrine hydrochloride (EP), Norepinephrine (NE) and Chitosan ($\geq$90% deacetylated) were purchased from J&K Chemical Ltd. (Shanghai, China). L-Dopa, DL-5-Hydroxytryptophan (DL-5-HTP) were obtained from Aladdin Chemistry Co., Ltd. (Shanghai, China). $Na_2HPO_4 \cdot 12H_2O$, $NaH_2PO_4 \cdot 2H_2O$, NaOH, HCl, potassium ferrocyanide trihydrate and potassium ferricyanide were obtained from Guoyao Chemical Reagent Co., Ltd. (Shanghai, China). Chemicals were all of analytical grades and used without further purification. Deionized water used in experiments was obtained from a Milli-Q Plus system (18.2 MΩ cm, Millipore Inc., USA). Human serum was provided by school infirmary (Huaqiao University, China). 0.1 M Phosphate buffer solutions (PBS) with different pH values were configurated through adjusting the mole ratio of NaH_2PO_4 and Na_2HPO_4.

Instrumentation

Cyclic voltammetry (CV), electrochemical impedance spectroscopy (EIS) and differential pulse voltammetry (DPV) were performed on CHI660E workstation (ChenHua Instruments, Shanghai, China) with a conventional three-electrode system. The bare or modified glassy carbon electrode (GCE, 2 mm in diameter) were served as working electrode, a platinum wire electrode and a saturated calomel electrode (SCE) were served as counter electrode and reference electrode respectively. A S-4800 Field emission scanning electron microscope (Hitachi, Japan), a Renishaw inVia Raman microscope (UK) and a Nexus-470 Fourier transform infrared spectrometer (Nicolet, USA) were used to characterize the formation of molecularly imprinted films.

Preparation of electrodeposition solution

Firstly, 0.35 g chitosan was dissolved in 20.0 mL 0.1 M HCl, which was then diluted with deionized water and adjusted pH to 5.0 with 0.1 M NaOH to get 7.0 mg mL^{-1} CS solution. GR suspension was prepared by adding 0.015 g graphene into 8.0 mL CS solution to get a homogeneous dispersion solution with ultrasonically dispersing and magnetic stirring. Finally, the electrodeposition solution was obtained by adding 2.0 mL 5.0 mM L-5-HTP dropwise into the being stirred GR suspension.

Preparation of the GR-MIP/GCE

To obtain a mirrorlike surface of electrode, the bare GCE was polished with 1.0, 0.3, 0.05 μm alumina slurry, and then rinsed and ultrasonicated sequentially in 1:1 diluted HNO_3, ethanol, deionized water. To construct GR-MIPs film, the rinsed GCE was immersed into the electrodeposition solution and applied a potential of −1.1 V for 120 s. It was then taken out for drying at room temperature. Finally, the L-5-HTP molecules were removed from the electrodeposited film via a constant potential of 1.0 V for 800 s in 0.01 M NaOH solution containing 100 μL ethanol. The schematic diagram for the preparation of GR-MIP/GCE was illustrated in Scheme 1. As a comparison, GR-NIP/GCE was prepared in the same way except the L-5-HTP in the electrodeposition solution. The NIP/GCE and MIP/GCE were prepared according to the above process without graphene.

Electrochemical measurement

Different modified electrodes were characterized by EIS at a frequency range from 0.1 Hz to 100 kHz in 10 mL 5.0 mM $K_3[Fe(CN)_6]$ containing 0.1 M KCl. Cycle voltammetric experiments were performed in the potential range from 0.0 to 1.0 V at different scan rate and DPV measurements were carried out in PBS of pH 6.0 with a potential range of 0.1–0.7 V, pulse width 50 ms, an amplitude of 50 mV and a pulse period of 0.5 s.

Results and discussion

Electrochemical properties of different modified electrodes

The CV and EIS experiments have been carried out for investigating interfacial electron transfer between solution and the modified GCE. The experiments were carried out in 10 mL 5.0 mM $Fe(CN)_6^{3-/4-}$ containing 0.10 M KCl solution. The equivalent circuit model in the inset of Fig. 1B is used to fit the Nyquist plots. R_s is the electrolyte resistance, R_{et} is the charge transfer resistance, and C_{dl} is the double-layer capacitance. EIS consists of two parts, semicircle part at high frequency corresponds to the electron transfer resistance (R_{et}) on the surface of the electrode and linear part at low frequency is related with the solution of the diffusion resistance (R_s). From Fig. 1A and B, a pair of well-defined redox peak with a peak potential difference (ΔE_p) of 116 mV appears at the bare GCE and the electron transfer resistance (R_{et}) of it is 37.01 Ω. However, the peak currents at the NIP/GCE and MIP/GCE decrease significantly and the ΔE_p increase to 161 mV and 141 mV respectively, while the R_{et} from the fitting equivalent circuit are 46.09 Ω and 39.37 Ω. The larger ΔE_p and the greater R_{et} at NIP/GCE and MIP/GCE imply that the CS modified film was poor conductor. But small ΔE_p and high peak currents at the GR-NIP/GCE ($\Delta E_p = 82$ mV) and GR-MIP/GCE ($\Delta E_p = 76$ mV) in accordance with the reduction of R_{et} for GR-NIP/GCE ($R_{et} = 36.85$ Ω) and GR-MIP/GCE ($R_{et} = 35.72$ Ω) verify that the addition of GR was conducive to the reversibility of GCE. That maybe the large specific area and high electrical conductivity of graphene facilitates the electron transfer. In addition, it can be seen that the smallest ΔE_p and the least R_{et} at GR-MIP/GCE, showing that both imprinted cavities and graphene contributed to the sensitivity.

The electrochemical behaviors of L-5-HTP

The electrochemical behaviors of L-5-HTP at NIP/GCE (a), MIP/GCE (b), bare GCE (c), GR-NIP/GCE (d), GR-MIP/GCE (e) were also investigated by DPV, as shown in Fig. 2. The order of the peak currents at the modified GCE is NIP/GCE < MIP/GCE < bare < GR-NIP/GCE < GR-MIP/GCE. Comparing e and b, d and a, it is found that the peak current increases significantly at the sensor decorated with graphene, which demonstrates that the special nanostructure of graphene with a large surface area and countless active sites catalyzed the L-5-HTP oxidation process. Furthermore, the peak currents at MIPs based sensor (b, e) are higher than those at the NIPs (a, d), imply that the nanopores structure helped more L-5-

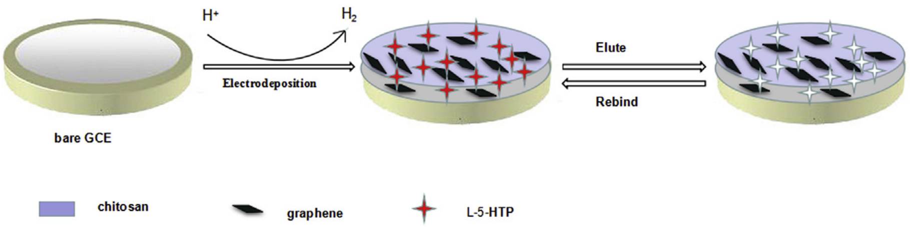

Scheme 1. Schematic illustration of the preparation of the GR-MIP/GCE.

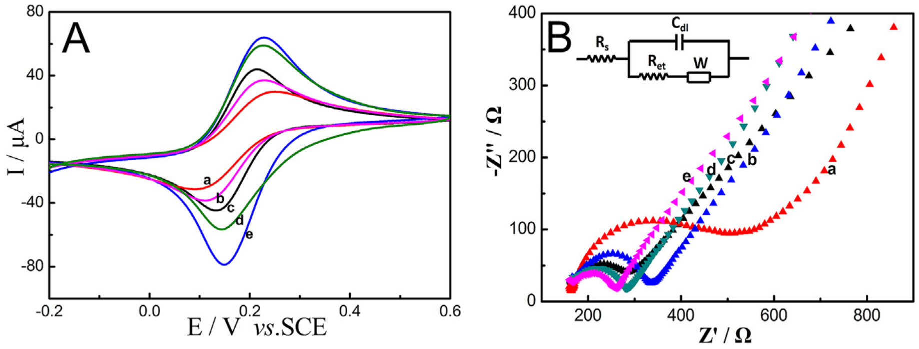

Fig. 1. Cyclic voltammograms (A) and EIS (B) of NIP/GCE (a), MIP/GCE (b), bare GCE (c), GR-NIP/GCE (d) and GR-MIP/GCE (e) in 10 mL 5.0 mM K_3 [$Fe(CN)_6$] containing 0.10 M KCl. Frequency range: 100 KHz to 0.1 Hz.

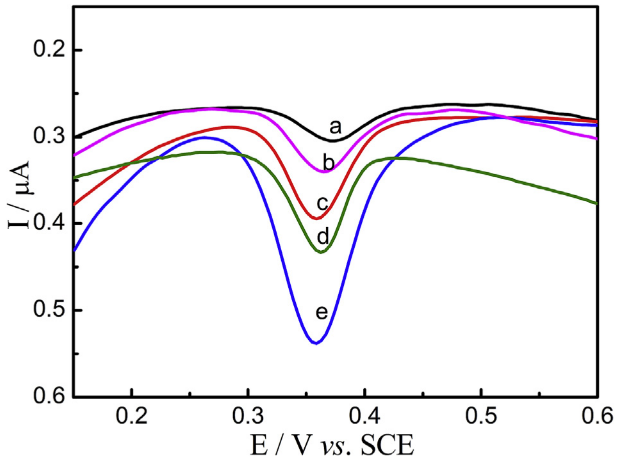

Fig. 2. DPVs of NIP/GCE (a), MIP/GCE (b), bare GCE (c), GR-NIP/GCE (d) and GR-MIP/GCE (e) in 10 mL 0.1 M PBS (pH = 6.0) containing 5.0 μM L-5-HTP.

HTP reacting on the electrode surface. The above results show that the graphene can amplify the response signal and the imprinting process is more beneficial to L-5-HTP identification.

Characterization of the GR-CS-MIP sensor

FESEM images

The GR-MIP electrodes were characterized by field emission scanning electron microscopy (FESEM) which can display a series of regular patterns on the surface of the modified electrodes [20]. As shown in Fig. 3a, a smooth surface is observed for the bare GCE. The FESEM images of the electrodeposited GR-CS-L-5-HTP films on GCE is shown in Fig. 3b. It is found that the surface morphology of electrodes are uneven and wrinkled, showing that modified membrane was formed. After the removal of template molecules from the electrodeposited films, as shown in Fig. 3c, the films exhibit a rough and multihole surface topography, which maybe the recognition sites left in the composite.

Raman spectra

Raman spectra were recorded to prove the existence of graphene on the modified electrode surface. As shown in Fig. 4A, a strong diamondoid (D) band at 1359 cm^{-1} and a weak graphitic (G) band at 1591 cm^{-1} are both appeared for the Raman spectra of GR-CS-L-5-HTP composite (curve a) and GR-CS composite (curve b) compared to CS composite (curve c), which are related to two vibration modes of GR [21]. The D-band and G-band are assigned to the breathing mode of *k*-point phonons of A_{1g} and the E_{2g} phonon of sp^2 carbon atoms respectively [11]. The results indicate that graphene can be dispersed in CS and was successfully modified onto the surface of GCE via CS electrodeposition.

FTIR spectra

The FTIR was used to investigate the composition of the modified films and the interaction of the functional group. As can be seen in Fig. 4B (curve a), the band at 1654 cm^{-1} (C=O stretching vibration) and 1560 cm^{-1} (N-H in-plane bending) are corresponding to the amide I and the amide II of CS respectively [22]. C_3-OH (C-O stretching vibration) of CS is located at 1077 cm^{-1}. In Fig. 4B (curve b), C=O, N-H and C_3-OH band have shifted to 1637 cm^{-1}, 1518 $cm^{-1}$and 1086 cm^{-1} respectively, that indicate CS has reacted with graphene during the electrodeposition process [23]. From Fig. 4B (curve c), the characteristic bands of L-5-HTP are observed at 1738 cm^{-1} and 704 cm^{-1} for carboxyl groups and C-H of benzenoid rings stretching vibrations respectively [23], and the 1518 cm^{-1} band of -NH_2 in CS shifted to 1505 cm^{-1}, showing that a chemical bond exists between L-5-HTP and CS. Moreover, the identification of a wide and weak band for C=C at 1580-1600 cm^{-1} shows that GR was decorated in the sensing membrane [24,25]. FTIR data indicate that GR-CS-L-5-HTP was modified on GCE successfully.

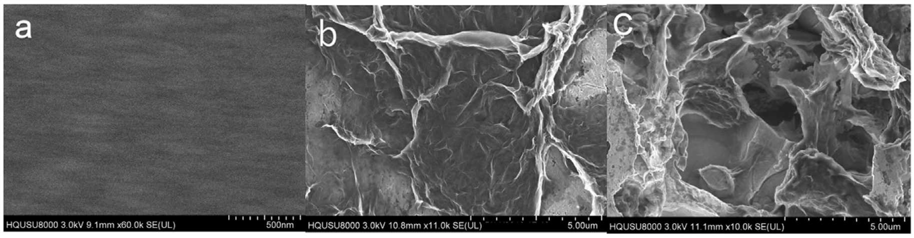

Fig. 3. FESEM images of the surface for bare GCE (a), GR-CS-L-5-HTP/GCE (b) and GR-MIP/GCE (c).

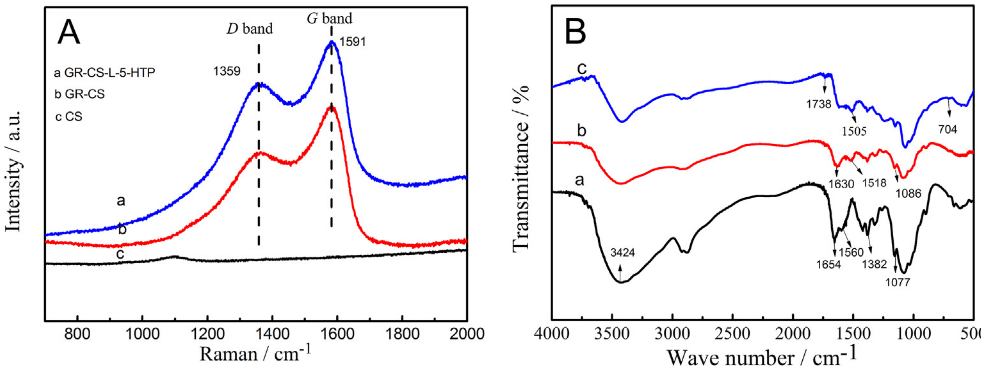

Fig. 4. (A) Raman spectra of GR-CS-L-5-HTP (a), GR-CS (b) and CS (c) composite; 532 nm radiation was used for excitation. (B) FTIR spectra of CS (a), GR-CS (b) and GR-CS-L-5-HTP (c) composite.

Optimization studies

Optimization of experimental conditions

Several important factors including GR doping quantity, electrodeposition time, eluent, elution time were optimized to get a highly sensitive, selective and stable GR-MIPs based sensor. Pivotal steps are GR doping quantity in deposition solution which influences the amount of GR in imprinted films and electrodeposition time which affects the thickness of GR-MIPs films. The results showed that 1.5 g L^{-1}GR and 120 s electrodeposition time would met the require.

Furthermore, the eluent and elution time were optimized. Ethanol-NaOH (1:100,V/V) was considered as an ideal extract solvent for removing the template molecules completely from the polymer matrix. And 800 s was taken as the optimum elution time, because the DPV oxidation peak of the template molecule disappeared (see Fig. S1 in the Supporting Information) after the polymer film was treated in elution solution at a potential of 1.0 V with stirred for 800 s.

The effect of pH

Different pH can effect the electrochemical behavior of L-5-HTP. The pH ranging from 5.0 to 7.5 on GR-MIP/GCE was investigated because the isoelectric point of L-5-HTP is 5.89. From the Fig. 5A, it is found that pH affect both the oxidation peak current and the peak potential of L-5-HTP, it also can be found that a negative potential of the broad oxidation peak shifts with increasing pH, and a linear relation between E_p and pH can be expressed by the equation: $E_p = -0.04021$ pH $+ 0.5806$ ($R^2 = 0.9975$). A slope of -0.0402 mV pH^{-1} is close to the Nernst equation value of -0.0591 mV pH^{-1}, which indicates that equal numbers of electrons and protons were participated in reaction [26,27]. It also suggests that the electro-oxidation of L-5-HTP proceeds with a mechanism as shown in Scheme 2 [28,29]. Fig. 5B (curve b) confirms that proton transfer and electron transfer reached an equilibrium in the process of the oxidation of L-5-HTP, and the maximum current can be got when the pH is 6.0.

The influence of scan rate

The influence of scan rate on the redox reaction of L-5-HTP at GR-MIP/GCE was observed by cyclic voltammetry under the condition of changing scanning rate. As can be seen from Fig. 6A, when scan rate changes from 0.01 V s^{-1} to 0.27 V s^{-1}, the oxidation peak currents increase gradually and vary linearly with the square root of the scan rates (inset in Fig. 6A), and the linear regression equation is I (μA) = 1.823 $v^{1/2}$ ($V^{1/2}$ $s^{-1/2}$)-0.1207 (R^2 = 0.9852). The linear relationship between peak current and the square root of scan rate indicates that the oxidation of L-5-HTP on the GR-MIP/GCE was a diffusion-controlled process.

The relationship between the oxidation peak potential and the scan rate was also explored in the Fig. 6B. It can be seen that the oxidation peak potentials shift positively with the increasing scan rate, and the linear regression equation is got as $E_{pa}(V) = 0.02901$ log υ (V s^{-1})+0.4275 (R^2 = 0.9642). For a diffusion-controlled process, the information about the rate determining step can be obtained by a Tafel plot using the following equation [11,30]:

$$E_p = (2.303RT/2n\alpha F)\log \upsilon + \cos \text{tan}t \quad (1)$$

where α is the electron-transfer coefficient, n is the electron-transfer number, υ is the scan rate. The slope of E_p vs. log υ for GR-MIP/GCE was found to be 0.02901. For the oxidation of L-5-HTP is a completely irreversible electrode process, α is assumed to be 0.6 [30]. Thus, n is calculated to be 2.

Specific recognition studies

Selectivity is the most important performance for GR-MIP/GCE. The recognition to structural analogues and the anti-interference ability among coexisting components were investigated as follows.

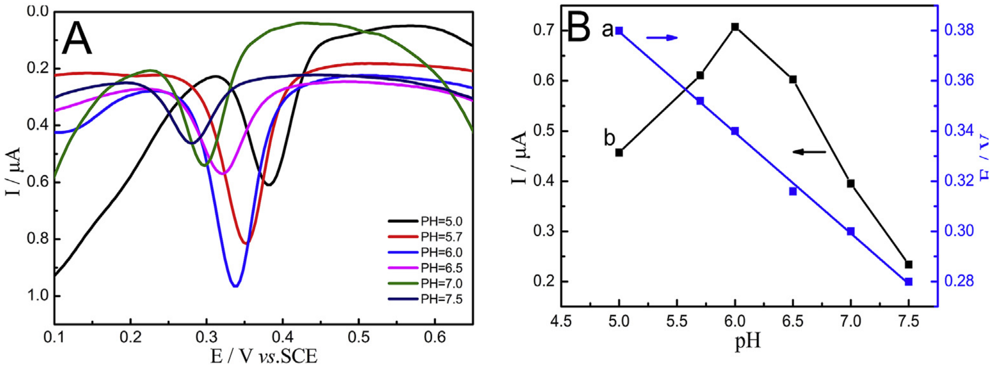

Fig. 5. (A) DPVs of 5.0 μM L-5-HTP containing varied pH solution at the GR-MIP/GCE. (B) The dependency of the peak potential (curve a) and peak current (curve b) of 5.0 μM L-5-HTP in different pH solutions. The pH range: 5.0 to 7.5.

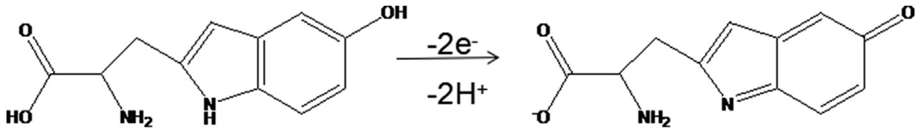

Scheme 2. The oxidization of L-5-HTP at GR-MIP/GCE.

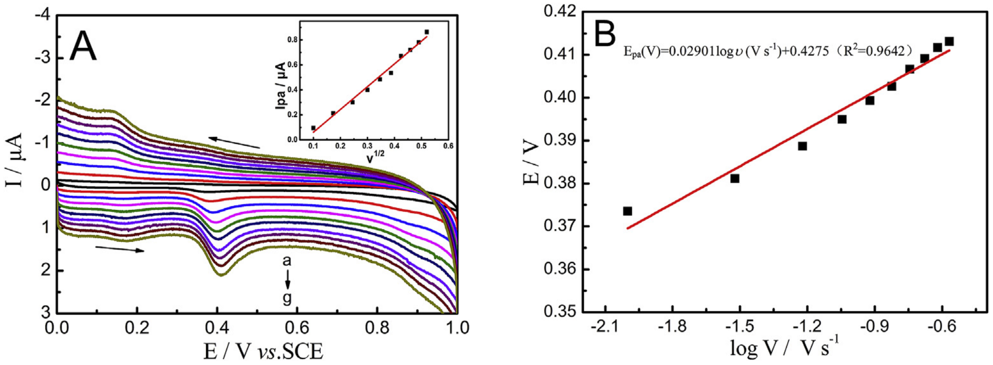

Fig. 6. (A) CVs of 5.0 μM L-5-HTP at the GR-MIP/GCE with different scan rates from 0.01 V s^{-1}(a) to 0.27 V s^{-1} (g). Inset is the dependency of the peak current with respect to the square root of the scan rate. (B) Dependence of oxidation peak potentials on logarithm of the scan rate.

Firstly, specific recognition of a series of structural analogues (5-HT, L-trp, D-trp and L-tyr) of L-5-HTP were carried out. Fig. 7A reveals that the current of L-5-HTP is the highest of all and changes significantly with the increasing concentration. However, there are small current response and low sensitivity to the other structural analogues. The obvious distinction maybe attribute to the complementary cavities in the polymer which are in match with the L-5-HTP stereo structure and multiple interactions between functional groups in the imprinted polymer and template are to improve the specificity [31].

Secondly, some possible coexisting components such as L-pro, L-His, L-tyr, EP, L-dopa, DA, NE, 5-HT, D-5-HTP were selected as interferents to evaluate the selectivity performance of the prepared sensor. The currents change ratio I/I_0 represents the anti-interference ability of GR-MIP/GCE. A series of I values were recorded for the current response of 5.0 μM L-5-HTP after adding different concentrations of interferents and I_0 was recorded without interference. As shown in Fig. 7B, when L-Pro, L-His, L-tyr, EP, L-dopa, DA, NE, 5-HT, D-5-HTP are 1000, 1000, 10, 4, 1, 1, 4, 1 and 1 times larger than L-5-HTP, respectively, the I/I_0 changes not more than 18%. Nevertheless, an obvious increase of the I/I_0 is observed after adding an equivalent amount of 5-HT. This distinct interference from 5-HT maybe due to its high electrochemical activity and structural similarity with L-5-HTP, but 5-HT will not influence on the detection of L-5-HTP in real samples at low concentration levels (0.01 μM-0.1 μM). Above results imply that the GR-MIP based sensor has excellent anti-interference capability in practical testing.

Analytical model and application

Adsorption kinetics curve can be used to study the dynamic adsorption performance of the established sensor. The models that best fit our data are the Langmuir adsorption model [32] with the following formula:

$$I_p = I_{pm} \cdot \frac{t}{K + t} \quad (2)$$

where I_p (μA) is the current response of L-5-HTP, I_{pm} (μA) is the current value at saturation capacity, t (s) is the adsorption time and K (s^{-1}) is the kinetic rate constant.

The absorbance of L-5-HTP at different modified electrodes was carried out in 10 mL PBS containing 5.0 μM L-5-HTP. As is shown in

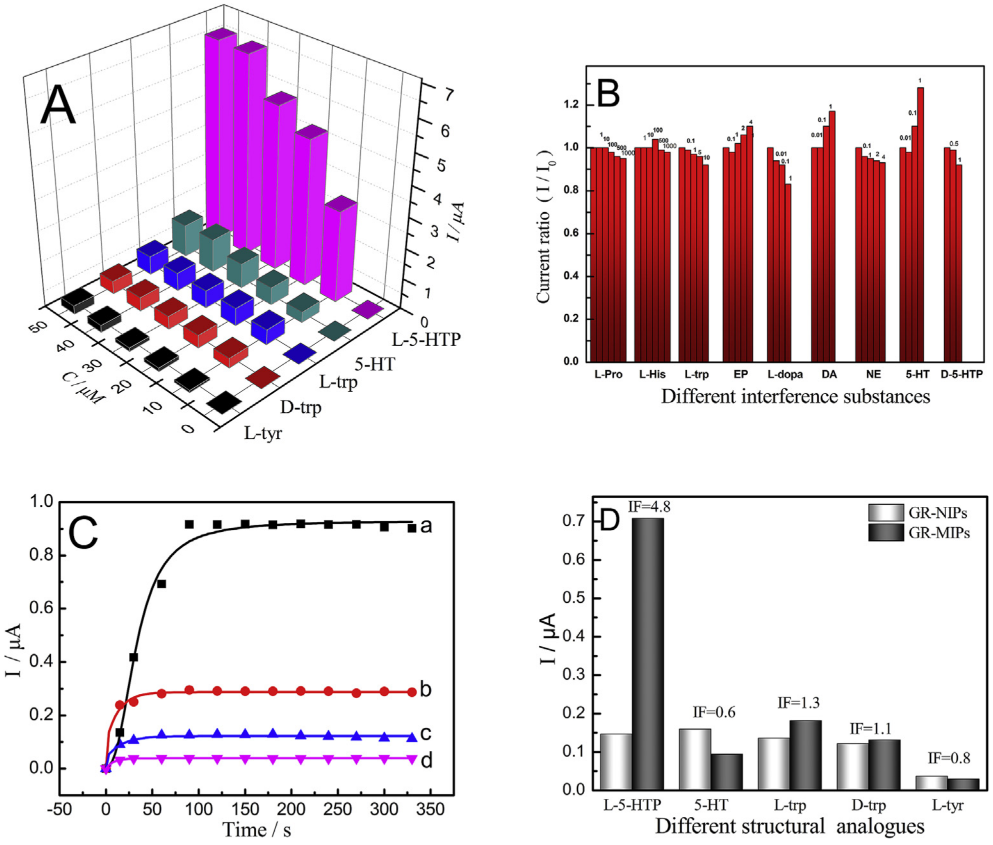

Fig. 7. (A) I_p of the GR-MIP/GCE for different structural analogues. (B) Peak Current ratio (I/I_0) of the GR-MIP/GCE for 5.0 μM L-5-HTP in the presence of varied concentrations of different interference substances. (C) Adsorption dynamic curve of the GR-MIP/GCE(a), GR-NIP/GCE(b), MIP/GCE(c), NIP/GCE(d). (D) I_{pm} of 5.0 μM L-5-HTP, 5-HT, L-trp, D-trp and L-tyr at GR-MIP/GCE (black) and GR-NIP/GCE (gray) respectively.

Table 1
The fitted parameters based on Langmuir adsorption model at different modified electrodes.

Sensor Type	R^2	I_{pm}/μA	K/s^{-1}
NIP/GCE	0.998	0.019	7.87
MIP/GCE	0.962	0.12	17.17
GR-NIP/GCE	0.984	0.29	13.59
GR-MIP/GCE	0.990	0.92	59.27

Fig. 7C. It can be seen that the I_p has a dramatically increase within the first 30 s, and then it increases slowly. One minutes later, the I_p reaches equilibrium. The reason of this phenomenon is that the L-5-HTP adsorption is fast in the surface of the cavity at the beginning, but after the adsorption on surface is saturated, the penetration of L-5-HTP molecule into the internal cavity becomes much more difficult.

The fitted parameters based on Langmuir adsorption model at different modified electrodes are shown in Table 1. The order of the I_{pm} at the modified sensor is GR-MIP/GCE > GR-NIP/GCE > MIP/GCE > NIP/GCE. Clearly, the I_{pm} at the imprinted based sensor is higher than that at nonimprinted. It easily demonstrates that MIP is effective for the determination of L-5-HTP. Besides, the increment of I_p at the sensor doped with graphene is larger than the sensor modified with CS means that the existence of graphene is in favor of gaining more reaction sites, improving binding stability, and accelerating the accumulation rate.

To further illustrate specific recognition performance of the imprinted sensor, the imprinting factor (IF) [33] can be calculated by the fitting parameters of I_{pm}.

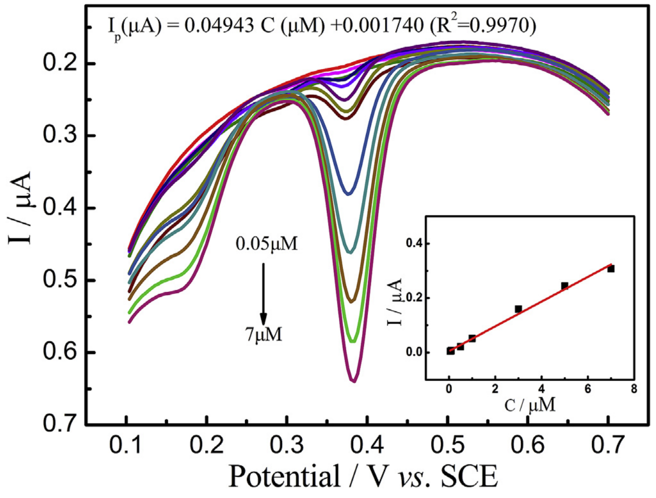

Fig. 8. DPV responses of the GR-MIP/GCE for L-5-HTP from 0.05 μM to 7.0 μM. The inset is the corresponding calibration plots of increasing L-5-HTP concentration obtained at GR-MIP/GCE.

$$IF = \frac{I_{pm}(MIP)}{I_{pm}(NIP)} \quad (3)$$

I_{pm}(MIP) is the response signal of GR-MIP/GCE to the analyte and I_{pm}(NIP) is corresponding to GR-NIP/GCE. A higher IF means a better selectivity of the GR-MIP/GCE. As shown in Fig. 7D, the current of

Table 2
Comparison of the different sensors for the detection of L-5-HTP.

Method	Modified electrode	linear range (μM)	Detection limit (μM)	Ref.
Differential pulse voltammetry	CNSs modified GCE	0.05–1.0	0.03	[7]
Amperometric detection	Ru^{II}terpyridine-doped composite electrode	1.0–40	0.05	[34]
Linear scan voltammetry	ITO/npSG/CoHCF[a] electrode	10–1000	2.1	[12]
Square wave voltammetry	PdNPs:MWCNTs/GCE	1–100	0.077	[5]
Linear sweep voltammetry	NGD[b]/PGE	0.02–2.0, 4.0–10.0	0.006	[35]
Differential pulse voltammetry	GR-MIPs/GCE	0.05–7.0	0.006	This work

[a] Indium tin oxide/nanoscale pores sol–gel/cobalt hexacyanoferrate.
[b] Nanomixture of graphite-diamond film.

Table 3
Results of L-5-HTP in human blood serum samples.

Sample	Added (μM)	Found (μM)	Recovery (%)	RSD (%) (n = 3)
blood serum-1	0	0.0657	–	1.0
	0.1	0.163	97.1	2.9
	0.3	0.362	98.7	1.2
	0.5	0.519	90.6	1.9
blood serum-2	0	0.0770	–	1.8
	0.1	0.175	98.4	3.1
	0.3	0.390	104.3	4.3
	0.5	0.568	98.2	1.5
blood serum-3	0	0.0500	–	1.7
	0.1	0.155	105.6	2.1
	0.3	0.360	103.6	1.6
	0.5	0.529	95.9	1.7

GR-MIP/GCE for L-5-HTP at a concentration of 5.0 μM shows much higher when compared with 5-HT, L-trp, D-trp and L-tyr, and the GR-MIP/GCE exhibits significant selectivity for L-5-HTP with the highest *IF* of 4.8.

Determination of L-5-HTP

Under optimal conditions, DPV responses of L-5-HTP were performed on the GR-MIP/GCE with various concentrations. As shown in Fig. 8, the current gradually increases with the increase of L-5-HTP concentration and fine linear relationships are obtained in the concentration range of 0.05–7.0 μM. The linear regression equation of I_p (μA) = 0.04943 C (μM) +0.001740 (R^2 = 0.9970) and the detection limit (LOD) is calculated to be 6 nM (S/N = 3). In addition, fine linear relationships are also obtained at the NIP/GCE, MIP/GCE and GR-NIP/GCE in the ranges of 0.7–7.0 μM, 0.5–10.0 μM and 0.1–5.0 μM, respectively, and the linear regression equations are as following: I_p (μA) = 0.009283 C (μM) +0.003430 (R^2 = 0.9889), I_p (μA) = 0.01906 C (μM) +0.006950 (R^2 = 0.9877), I_p (μA) = 0.02507 C (μM) +0.001381 (R^2 = 0.9986) (see Fig. S2 in the Supporting Information). It can be seen that GR-MIP/GCE offers higher sensitivity and wider linear range than other modified sensors.

The repeatability of GR-MIP/GCE was investigated by continuous determinations of 5 μM L-5-HTP in PBS (pH = 6.0) for 10 times with a RSD of 4.7%, which demonstrated an excellent repeatability for the sensor. What is more, the GR-MIP based sensors could be reused for 5 times, and its RSD was 3.4%, indicating good practicability for this sensor. Comparision to other literature listed in Table 2, the proposed sensor also displays many merits of low detection limit, wide detection range and specific recognition.

Real sample analysis

In order to evaluate the applicability of GR-MIPs based sensor in real sample, the sensor was applied to human blood serum for the determination of L-5-HTP. The samples were diluted with 0.1 M PBS (pH = 6.0) for 500-folds before measurement. As shown in Table 3, the recoveries from the three samples were in the range from 90.6% to 105.6%, with the RSD ranged from 1.0% to 4.3%, illustrated that the proposed sensor is effective and reliable for determination of L-5-HTP in blood serum.

Conclusion

In summary, owing to imprinted process and graphene magnified ability, an expected sensor was obtained for selective recognition and sensitive detection of L-5-HTP. The prepared procedure of the sensor was simple but exhibited many advantages, including good sensitivity, selectivity, repeatability, a wide linear range with low detection limit. It has been applied to detect L-5-HTP in human blood serum successfully which indicates that it has the potential to exploit medical diagnosis and practical analyses for related diseases.

Acknowledgements

This work was supported by the National Natural Science Foundation of China (Grant no. 21575044, 21404045 and 21275059), the Natural Science Foundation of Fujian Province of China (Grant no. 2013J01047, 2015J01054 and 2016J01062).

Appendix A. Supplementary data

Supplementary data related to this article can be found at http://dx.doi.org/10.1016/j.ab.2017.03.017.

References

[1] I.J. Tunna, B.A. Patel, Analysis of 5-hydroxytryptophan in the presence of excipients from dietary capsules: comparison between cyclic voltammetry and UV visible spectroscopy, Anal. Methods 5 (2013) 2523–2528.
[2] C.P. Lynn-Bullock, K. Welshhans, S.L. Pallas, P.S. Katz, The effect of oral 5-HTP administration on 5-HTP and 5-HT immunoreactivity in monoaminergic brain regions of rats, J. Chem. Neuroanat. 27 (2004) 129–138.
[3] E.H. Turner, J.M. Loftis, A.D. Blackwell, Serotonin a la carte: supplementation with the serotonin precursor 5-hydroxytryptophan, Pharmacol. Ther. 109 (2006) 325–338.
[4] L. Wang, H. Erlandsen, J. Haavik, P.M. Knappskog, R.C. Stevens, Three-dimensional structure of human tryptophan hydroxylase and its implications for the biosynthesis of the neurotransmitters serotonin and melatonin, Biochemistry 41 (2002) 12569–12574.
[5] N. Kumar, Rosy, R.N. Goyal, Palladium nano particles decorated multi-walled carbon nanotubes modified sensor for the determination of 5-hydroxytryptophan in biological fluids, Sens. Actuators, B 239 (2017) 1060–1068.
[6] T.C. Birdsall, 5-Hydroxytryptophan: a clinically-effective serotonin precursor, Altern. Med. Rev. 3 (4) (1998) 271–280.
[7] Y. Chen, G. Li, Y. Hu, A sensitive electrochemical method for the determination of 5-hydroxytryptophan in rats' brain tissue based on a carbon nanosheets-modified electrode, Anal. Methods 7 (2015) 1971–1976.
[8] G. Koppisetti, A. Siriki, K. Sukala, G.V. Subbaraju, Estimation of l-5-hydroxytryptophan in rat serum and Griffonia seed extracts by liquid chromatography–mass spectrometry, Anal. chim. acta 549 (2005) 129–133.
[9] W. Tseng, T. Cheng, Ultrasensitive detection of indoleamines by combination of nanoparticle-based extraction with capillary electrophoresis/laser-induced

native fluorescence, J. Chromatogr. A 1216 (2009) 6451–6458.
[10] A.G. Coelho, F.P. Aguiar, D.P.D. Jesus, A rapid and simple method for determination of 5-hydroxytryptophan in dietary supplements by capillary electrophoresis, J. Braz. Chem. Soc. 25 (2014) 783–787.
[11] P.K. Aneesh, S.R. Nambiar, T.P. Rao, A. Ajayaghosh, Electrochemically synthesized partially reduced graphene oxide modified glassy carbon electrode for individual and simultaneous voltammetric determination of ascorbic acid, dopamine and uric acid, Anal. Methods 6 (2014) 5322–5330.
[12] D. Ranganathan, S. Zamponi, M. Berrettoni, B.L. Mehdi, J.A. Cox, Oxidation and flow-injection amperometric determination of 5-hydroxytryptophan at an electrode modified by electrochemically assisted deposition of a sol–gel film with templated nanoscale pores, Talanta 82 (2010) 1149–1155.
[13] H.C.B. Kalachar, Y.A. Naik, S. Basavanna, R. Viswanatha, T.G. Venkatesha, T.Sheela, Amperometric and differential pulse voltammetric determination of 5-hydroxy-l-tryptophan in pharmaceutical samples using gold modified pencil graphite electrode, J. Chem. Pharm. Res. 3 (3) (2011) 530–539.
[14] M. Pumera, A. Ambrosi, A. Bonanni, E.L.K. Chng, H.L. Poh, Graphene for electrochemical sensing and biosensing, TrAC, Trends Anal. Chem. 29 (2010) 954–965.
[15] Y. Shao, J. Wang, H. Wu, J. Liu, I.A. Aksay, Y. Lin, Graphene based electrochemical sensors and biosensors: a review, Electroanalysis 22 (2010) 1027–1036.
[16] M. Pumera, Electrochemistry of graphene: new horizons for sensing and energy storage, Chem. Rec. 9 (2009) 211–223.
[17] S.A. Piletsky, A.P.F. Turner, Electrochemical sensors based on molecularly imprinted polymers, Electroanalysis 45 (2002) 317–323.
[18] M.C. Blanco-López, M.J. Lobo-Castañón, A.J. Miranda-Ordieres, P. Tuñón-Blanco, Electrochemical sensors based on molecularly imprinted polymers, Trends Anal. Chem. 23 (2004) 36–48.
[19] Y.P. Chen, B. Liu, H.T. Lian, X.Y. Sun, Preparation and application of urea electrochemical sensor based on chitosan molecularly imprinted films, Electroanalysis 23 (2011) 1454–1461.
[20] Y. Li, Y. Liu, Y. Yang, F. Yu, J. Liu, H. Song, J. Liu, H. Tang, B. Ye, Z. Sun, Novel electrochemical sensing platform based on a molecularly imprinted polymer decorated 3D nanoporous nickel skeleton for ultrasensitive and selective determination of metronidazole, ACS Appl. Mater. Inter 7 (2015) 15474–15480.
[21] S. Yang, S. Luo, C. Liu, W. Wei, Direct synthesis of graphene–chitosan composite and its application as an enzymeless methyl parathion sensor, Colloids Surf. B 96 (2012) 75–79.
[22] D. Han, T. Han, C. Shan, A. Ivaska, L. Niu, Simultaneous determination of ascorbic acid, dopamine and uric acid with chitosan-graphene modified electrode, Electroanalysis 22 (2010) 2001–2008.
[23] L. Lin, H. Lian, X. Sun, Y. Yu, B. Liu, An L-dopa electrochemical sensor based on a graphene doped molecularly imprinted chitosan film, Anal. Methods 7 (2015) 1387–1394.
[24] C. Zhang, D.M. Dabbs, L. Liu, I.A. Aksay, R. Car, A. Selloni, Combined effects of functional groups, lattice defects, and edges in the infrared spectra of graphene oxide, J. Phys. Chem. C 119 (2015) 18167–18176.
[25] M. Acik, G. Lee, C. Mattevi, A. Pirkle, R.M. Wallace, M. Chhowalla, K. Cho, Y. Chabal, The role of oxygen during thermal reduction of graphene oxide studied by infrared absorption spectroscopy, J. Phys. Chem. C 115 (2011) 19761–19781.
[26] S. Deng, G. Jian, J. Lei, Z. Hu, H. Ju, A glucose biosensor based on direct electrochemistry of glucose oxidase immobilized on nitrogen-doped carbon nanotubes, Biosens. Bioelectron 25 (2009) 373–377.
[27] Z. Kun, C. Hongtao, Y. Yue, B. Zhihong, L. Fangzheng, L. Sanming, Platinum nanoparticle-doped multiwalled carbon-nanotube-modified glassy carbon electrode as a sensor for simultaneous determination of atenolol and propranolol in neutral solution, Ionics 21 (2015) 1129–1140.
[28] X. Lin, Y. Li, Monolayer covalent modification of 5-hydroxytryptophan on glassy carbon electrodes for simultaneous determination of uric acid and ascorbic acid, Electrochim. Acta 51 (2006) 5794–5801.
[29] K. Humphries, G. Dryhurst, Electrochemical oxidation of 5-hydroxytryptophan in acid solution, J. Pharm. Sci. 76 (1987) 839–847.
[30] Y. Wang, L. Wang, H. Chen, X. Hu, S. Ma, Fabrication of highly sensitive and stable hydroxylamine electrochemical sensor based on gold nanoparticles and metal–metalloporphyrin framework modified electrode, ACS Appl. Mater. Inter 8 (2016) 18173–18181.
[31] H. Kim, G. Guiochon, Adsorption on molecularly imprinted polymers of structural analogues of a template. Single-component adsorption isotherm data, Anal. Chem. 77 (2005) 6415–6425.
[32] T.Y. Guo, Y.Q. Xia, G.J. Hao, M.D. Song, B.H. Zhang, Adsorptive separation of hemoglobin by molecularly imprinted chitosan beads, Biomaterials 25 (2004) 5905–5912.
[33] A. Mehdinia, M.O. Aziz-Zanjani, M. Ahmadifar, A. Jabbari, Design and synthesis of molecularly imprinted polypyrrole based on nanoreactor SBA-15 for recognition of ascorbic acid, Biosens. Bioelectron. 39 (2013) 88–93.
[34] J.L. Cohen, J. Widera, J.A. Cox, Electrocatalytic Oxidation and Flow Injection Amperometric determination of 5-hydroxytryptophan, Electroanalysis 14 (2002) 231–234.
[35] S. Shahrokhian, M. Bayat, Pyrolytic graphite electrode modified with a thin film of a graphite/diamond nano-mixture for highly sensitive voltammetric determination of tryptophan and 5-hydroxytryptophan, Microchim. Acta 174 (2011) 361–366.
