# Supplementary material for: 5-Hydroxytryptophan (5-HTP): Natural Occurrence, Analysis, Biosynthesis, Biotechnology, Physiology and Toxicology
Source: Int J Mol Sci. 2020 Dec 26;22(1):181. doi: 10.3390/ijms22010181 (PMC7796270; doi:10.3390/ijms22010181)
Supplement: Supplementary file 1 [file ijms-22-00181-s001.zip › 5-HTP.Data/PDF/1637466468/Hara-2013-Enhanced-synthesis-of--hydroxy-l-tr.pdf]

Hara and Kino *AMB Express* 2013, **3**:70
http://www.amb-express.com/content/3/1/70

AMB Express
a SpringerOpen Journal

ORIGINAL ARTICLE Open Access

Enhanced synthesis of 5-hydroxy-L-tryptophan through tetrahydropterin regeneration

Ryotaro Hara[1] and Kuniki Kino[1,2*]

Abstract

5-Hydroxy-L-tryptophan (5-HTP) is a naturally occurring aromatic amino acid present in the seeds of the African plant *Griffonia simplicifolia*. Although 5-HTP has therapeutic effects in various symptoms, efficient method of producing 5-HTP has not been established. In this study, we developed a novel cofactor regeneration process to achieve enhanced synthesis of 5-HTP by using modified L-phenylalanine 4-hydroxylase of *Chromobacterium violaceum*. For the synthesis of 5-HTP using *Escherichia coli* whole cell bioconversion, L-tryptophan and 5-HTP degradation by *E. coli* endogenous catabolic enzymes should be considered. The tryptophanase gene was disrupted using the λ red recombination system, since tryptophanase is postulated as an initial enzyme for the degradation of L-tryptophan and 5-HTP in *E. coli*. For regeneration of the cofactor pterin, we screened and investigated several key enzymes, including dihydropteridine reductase from *E. coli*, glucose dehydrogenase from *Bacillus subtilis*, and pterin-4α-carbinolamine dehydratase from *Pseudomonas syringae*. Genes encoding these three enzymes were overexpressed in an *E. coli* tryptophanase-deficient host, resulting in the synthesis of 0.74 mM 5-HTP in the presence of 0.1 mM pterin and the synthesis of 0.07 mM 5-HTP in the absence of regeneration of pterin. These results clearly indicated the successful regeneration of pterin. Following optimization of the reaction conditions, 2.5 mM 5-HTP was synthesized with cofactor regeneration, while 0.8 mM 5-HTP was recovered without cofactor regeneration under the same reaction conditions, suggesting that the principle described here provides a new method for cofactor regeneration.

Keywords: 5-hydroxy-L-tryptophan; L-phenylalanine 4-hydroxylase; Tetrahydropterin; NAD(P)H; Cofactor regeneration

Introduction

5-Hydroxy-L-tryptophan (5-HTP) is a serotonin pathway metabolite of L-tryptophan in the brain. 5-HTP controls the concentration of serotonin, and therefore plays a significant role in the serotonin pathway. In this pathway, L-tryptophan is hydroxylated by tryptophan 5-hydroxylase to form 5-HTP, following which aromatic amino acid decarboxylase (Zhu and Juorio 1995) catalyzes the formation of 5-hydroxytryptamine (serotonin). The tryptophan hydroxylation reaction has been identified as the rate-limiting step in the serotonin pathway, and this reaction regulates the formation of serotonin. Hence, serotonin levels are elevated by oral administration of 5-HTP, leading to therapeutic effects in the case of various symptoms, including depression (Angst et al. 1977; Persson and Roos 1967; Takahashi et al. 1975; van Praag and de Hann 1980), chronic headache (Bono et al. 1984; Ribeiro 2000), and insomnia (Guilleminault et al. 1973; Wyatt et al. 1971). Moreover, 5-HTP acts as an antioxidant agent (Cadenas et al. 1989; Lysek et al. 2003).

Numerous studies have reported the drug evaluation and medicinal properties of 5-HTP; however, the production of 5-HTP is currently dependent mainly on extraction from the seeds of the African plant *Griffonia simplicifolia*. This method is not suitable for efficient production of 5-HTP; therefore, a more practical method providing better yield is required. Alternatively, pterin-dependent aromatic amino acid hydroxylases (Fitzpatrick 2000), which are family of enzymes that require tetrahydropterin as a cofactor, including phenylalanine hydroxylase, and both tyrosine and tryptophan hydroxylases (rate-limiting enzymes for catecholamine and serotonin pathways, respectively),

* Correspondence: kkino@waseda.jp
[1]Research Institute for Science and Engineering, Waseda University, 3-4-1 Ohkubo, Shinjuku-ku, Tokyo 169-8555, Japan
[2]Department of Applied Chemistry, Faculty of Science and Engineering, Waseda University, 3-4-1 Ohkubo, Shinjuku-ku, Tokyo 169-8555, Japan

Springer
© 2013 Hara and Kino; licensee Springer. This is an Open Access article distributed under the terms of the Creative Commons Attribution License (http://creativecommons.org/licenses/by/2.0), which permits unrestricted use, distribution, and reproduction in any medium, provided the original work is properly cited.

have been identified as candidates capable of efficiently synthesizing hydroxy aromatic amino acids. These enzymes are widely distributed throughout vertebrate species, and the human pterin-dependent aromatic amino acid hydroxylases in particular have been well elucidated. Tryptophan 5-hydroxylase is capable of efficiently synthesizing 5-HTP via the hydroxylation of L-tryptophan; however, this method is not suitable for industrial use due to low hydroxylation activity and poor stability when expressed both in prokaryotes and eukaryotes (Wang et al. 2002). In contrast, some prokaryotes, such as *Chromobacterium violaceum* (Nakata et al. 1979; Onishi et al. 1991) and *Pseudomonas aeruginosa* (Zhao et al. 1994), also produce L-phenylalanine 4-hydroxylase, but tryptophan 5-hydroxylase is not found in any of these microbes.

L-Phenylalanine 4-hydroxylase from *C. violaceum* (CviPAH) can hydroxylate the C-5 position of L-tryptophan as well as the C-4 position of L-phenylalanine (Fujisawa and Nakata 1987; Nakata et al. 1979; Pember et al. 1987). However, the hydroxylation rate of L-tryptophan is relatively lower than that of L-phenylalanine (~0.4%). In our previous study, we performed structure-based rational designing of CviPAH, and the resulting mutant enzyme, CviPAH-L101Y-W180F, exhibited a 5.2-fold higher k_{cat} value than the wild-type enzyme during L-tryptophan hydroxylation (Kino et al. 2009). This enzyme reaction should have high potential for practical production of 5-HTP because no by-products are formed, and the substrate, L-tryptophan, is available through industrial microbial fermentation performed using metabolically engineered *Corynebacterium glutamicum* (58 g/L) (Ikeda and Katsumata 1999) and *Escherichia coli* (54.6 g/L) (Azuma et al. 1993), both of which provide excellent yield. However, CviPAH-L101Y-W180F still requires pterin as a cofactor for enzyme activity, which is similar to the requirements of other types of aromatic amino acid hydroxylases. We attempted to regenerate the cofactor to overcome this problem, since pterin is an expensive compound. Here, we report the development of an enhanced L-tryptophan hydroxylation process coupled with regeneration of the cofactors pterin and NAD(P)H. Pterin biosynthesis and regeneration are observed in mammals; therefore, we cloned and reconstructed the corresponding enzymes derived from various microorganisms in *E. coli* with the aim of developing an enhanced enzymatic selective synthesis method for the production of 5-HTP.

Materials and methods

Chemicals and strains

6,7-Dimethyl-5,6,7,8-tetrahydropterine ($DMPH_4$) hydrochloride was purchased from Sigma (St. Louis, MO). All other chemicals were of the highest analytical grade available. *Bacillus subtilis* ATCC 23857 and genomic DNA of *Pseudomonas syringae* ATCC BAA-978D were obtained from American Type Culture Collection.

Disruption of the chromosomal *tnaA* gene in *E. coli*

E. coli BL21(DE3) *tnaA*$^-$ was constructed using the λ Red recombination system (Datsenko and Wanner 2000). A deletion cassette for *tnaA* gene, which encodes tryptophanase, was amplified by PCR using FRT-PKG-gb2-neo-FRT (Gene Bridges; Heidelberg, Germany) as template DNA and the following primers: forward 1 (5′-TGTAATATTCACAGGGATCACTGTAATTAAAATAAATGAAGGATTATGTAAATTAACCCTCACTAAAGGGCG-3′) and reverse 1 (5′-TGTAGGGTAAGAGAGTGGCTAACATCCTTATAGCCACTCTGTAGTATTAATAATACGACTCACTATAGGGCTC-3′). KOD -plus-DNA polymerase (Toyobo; Osaka, Japan) was used, and the PCR program was as follows: 94°C for 2 min and 25 cycles each of 94°C for 20 s, 60°C for 15 s, and 68°C for 2 min. According to the manufacturer's recommendation, mutant strains were selected and transferred onto Luria Bertani agar (10 g/L Bacto Tryptone, 5 g/L Bacto Yeast Extract, 10 g/L NaCl and 15 g/L Bacto Agar) containing 30 μg/mL kanamycin, and the resulting *tnaA*-deficient mutant was collected and used as a host for the subsequent experiments. Degradation of L-tryptophan and 5-HTP was performed in a reaction mixture containing 100 mM HEPES-NaOH buffer (pH 7.5), 5 mM L-tryptophan, 0.1 mM $DMPH_4$, 0.1 mM $FeSO_4$, 50 mM D-glucose, 1% (v/v) Triton X-100, and *E. coli* whole cells (OD_{660} = 50) in a total volume of 1 mL.

Construction of expression vectors

The gene encoding PAH-L101Y-W180F was amplified by PCR using the following primers: forward 2 (5′-ATATA*CATATG*AACGACCGCGCCGACTTTGTGGTGC-3′; the *Nde* I site is underlined) and reverse 2 (5′-TTCAT*CTCGAG*ATTAGACGTCTTCGGTATCCGCCCATCC-3′; the *Xho* I site is underlined) from pEPAH-L101Y-W180F (Kino et al. 2009). The gene encoding pterin-4α-carbinolamine dehydratase (PCD, GenBank Protein ID: AAZ35428) was amplified using the following primers: forward 3 (5′-AATAG*CCATGG*GTACCTTGAATCAAGCCCATTGCG-3′; the *Nco* I site is underlined) and reverse 3 (5′-AATAT*GTCGAC*ATTATTTGCGGCCTTCGGCGCCACTG-3′; the *Sal* I site is underlined) from the chromosomal DNA of *P. syringae*. The amplified genes were inserted into the corresponding restriction site of pETDuet-1 (Novagen; Madison, WI). The resulting plasmid was labeled pEDPCD-PAH-L101Y-W180F and used for the expression of PCD and PAH-L101Y-W180F genes in *E. coli*. The gene encoding dihydropteridine reductase (DPR, GenBank Protein ID: AAC73679) was amplified by PCR using the following primers: forward 4 (5′-TCATTAT*CATATG*GATATCA

TTTCTGTCGCCTTAAAGCGT-3′; the *Nde* I site is underlined) and reverse 4 (5′- TGTAATAG*CTCGAG* TTTACACTTCGGTTAAGGTGATGTTTTGCG-3′; the *Xho* I site is underlined) from the chromosomal DNA of *E. coli* K-12 MG1655. The glucose dehydrogenase (GDH, GenBank Protein ID: CAB12201) gene was amplified using the following primers: forward 5 (5′-CTA TA*GAATTC*GTATCCGGATTTAAAAGGAAAAG-3′; the *Eco*R I site is underlined) and reverse 5 (5′-*ATTATGTC GACA*TTAACCGCGGCCTGCCTGGAATGAAG-3′; the *Sal* I site is underlined) from the chromosomal DNA of *B. subtilis*. The amplified genes were inserted into the corresponding restriction site of pACYCDuet-1 (Novagen). The resulting plasmid was labeled pAGDH-DPR and was used for the expression of GDH and DPR genes in *E. coli*.

Preparation of recombinant *E. coli* BL21(DE3) *tnaA*$^{-}$ expressing PAH-L101Y-W180F, PCD, DPR and GDH genes

E. coli BL21(DE3) *tnaA*$^{-}$ harboring pEDPCD-PAH-L101Y-W180F and pAGDH-DPR was cultivated at 37°C in LB medium containing 50 μg/mL ampicillin and 30 μg/mL chloramphenicol. Isopropyl-β-D-thiogalactopyranoside (IPTG) was added to the culture when the OD_{660} reached 0.5, to a final concentration of 0.1 mM, and the cells were then cultivated at 25°C with shaking. After a 20-h induction period, *E. coli* BL21(DE3) harboring plasmids were harvested by centrifugation at 5,000 × *g* for 5 min at 4°C. The cell pellet was washed twice with 200 mM HEPES-NaOH buffer (pH 8.0) and resuspended in the same buffer. To confirm the expression of enzymes, SDS-PAGE analysis was carried out on supernatant of disrupted cells.

Synthesis of 5-HTP thorough cofactor regeneration

5-HTP was synthesized in a reaction mixture containing 100 mM HEPES-NaOH buffer (pH 8.0), 5 mM L-tryptophan, $DMPH_4$ at different concentrations, 0.1 mM $FeSO_4$, 50 mM D-glucose, 1% (v/v) Triton X-100, and *E. coli* whole cells (OD_{660} = 50) in a total volume of 1 mL. The reaction was performed at 30°C. After the reaction, cells were removed by centrifugation at 20,000 × *g* for 5 min at 4°C. The concentration of 5-HTP in the reaction mixture was determined by high performance liquid chromatography as described previously (Kino et al. 2009).

Results

Construction of the *E. coli* BL21(DE3) *tnaA*$^{-}$ strain

During the production of 5-HTP, the endogenous metabolic enzymes of *E. coli* (e.g., tryptophanase) degrade L-tryptophan to indole, pyruvate, and ammonia; and 5-HTP is degraded to 5-hydroxyindole, pyruvate, and ammonia (Nakazawa et al. 1972a; Nakazawa et al. 1972b; Watanabe and Snell 1972). We also confirmed the degradation of 5-HTP as well as of L-tryptophan in the wild-type *E. coli* whole cell reaction (Figure 1), and then attempted to delete the gene encoding tryptophanase (*tnaA*), which is the enzyme that initially degrades L-tryptophan, to overcome these drawbacks.

To prevent the degradation of L-tryptophan and 5-HTP, the *tnaA* gene of *E. coli* BL21 (DE3) was disrupted using the Red/ET recombination system. Consequently, the degradation of L-tryptophan and 5-HTP was clearly inhibited by disruption of the *tnaA* gene of *E. coli* (Figure 1), suggesting that this host strain contributes to enhance 5-HTP production.

Synthesis of 5-HTP catalyzed by PAH without $DMPH_4$ regeneration

To achieve L-tryptophan hydroxylation using PAH-L101Y-W180F, it is necessary to add the coenzyme $DMPH_4$ to the reaction mixture. Using *E. coli* BL21 (DE3) *tnaA*$^{-}$ whole cells expressing only PAH-L101Y-W180F gene, 5-HTP was synthesized in the presence of 0.1–5 mM $DMPH_4$ and was observed to increase with increase in the concentration of $DMPH_4$ up to 18% (0.9 mM) at 5 mM $DMPH_4$, indicating that synthesis of 5-HTP is coenzyme $DMPH_4$-dependent. We also attempted to develop higher yield synthesis of 5-HTP by reduced addition of $DMPH_4$.

Effect of coexpression of the DPR, GDH, and PCD genes

To construct $DMPH_4$ regeneration through oxidation-reduction reactions, two plasmids were constructed: pEDPCD-PAH-L101Y-W180F and pAGDH-DPR. Both

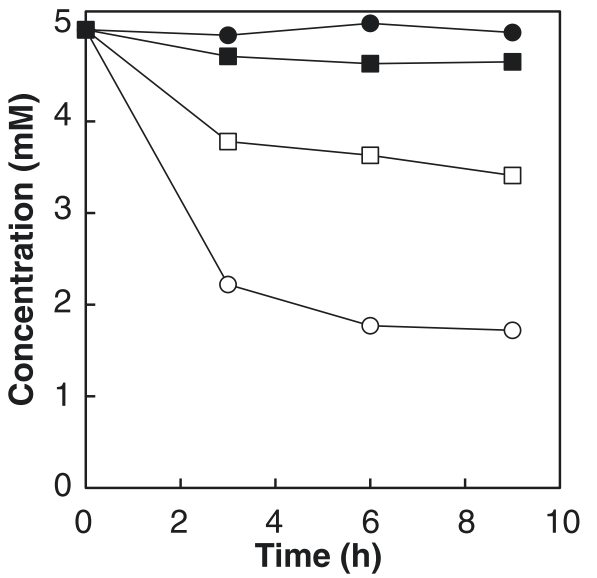

Figure 1 Degradation of L-tryptophan and 5-HTP using *E. coli* cells. *Open circles*, L-tryptophan using wild-type *E. coli*; *filled circles*, L-tryptophan using *E. coli tnaA* disruptant; *open squares*, 5-HTP using wild-type *E. coli*; *filled squares*, 5-HTP using *E. coli tnaA* disruptant.

plasmids were introduced into *E. coli* BL21(DE3) *tnaA*$^-$, and the genes encoding PAH-L101Y-W180F, PCD, DPR, and GDH were coexpressed in the same *E. coli* cells. SDS-PAGE analysis revealed that all of enzymes were successfully expressed in *E. coli* compared to negative control (Figure 2). Hence, we applied this strain as a catalyst for L-tryptophan hydroxylation using a whole cell reaction (Figure 3).

In order to initially regenerate $DMPH_4$ from 6,7-dimethyl-5,6,7,8-dihydropterine ($DMPH_2$), which is an oxidized form of $DMPH_4$, DPR from *E. coli* K-12 (Vasudevan et al. 1988) and PAH-L101Y-W180F were coexpressed in the same *E. coli* cells. DPR was selected for $DMPH_4$ regeneration because it exhibits tetrahydropterin-dependent NAD(P)H oxidoreductase activities. This approach resulted in an increase in 5-HTP synthesis compared to that obtained with PAH-L101Y-W180F alone with 0.1 mM $DMPH_4$ (Figure 4). Furthermore, 0.35 mM 5-HTP was synthesized in the presence of DPR with only 0.1 mM $DMPH_4$, suggesting that DPR served as a $DMPH_4$ regeneration enzyme.

To further accelerate $DMPH_4$ regeneration using *E. coli* endogenous NAD(P)H, it is necessary to regenerate NAD(P)H from NAD(P)$^+$ in the *E. coli* cell. For NAD(P)H regeneration, GDH from *B. subtilis* was selected since it serves as a cofactor in NAD(P)H regeneration.

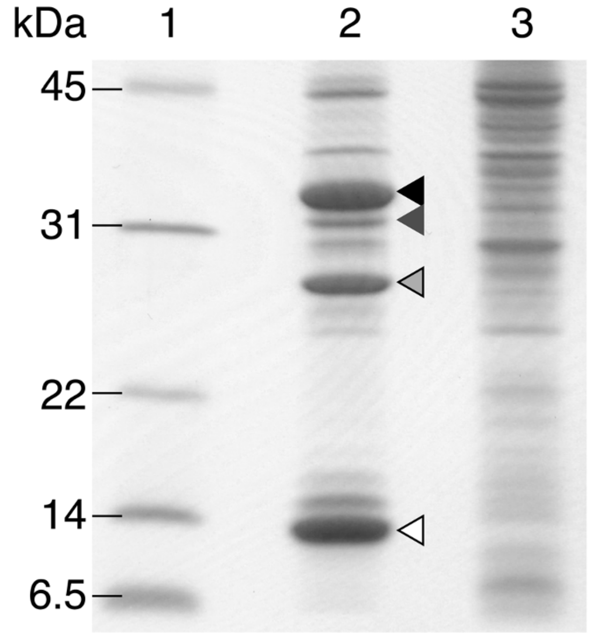

Figure 2 SDS-PAGE analysis of soluble proteins in *E. coli* cells. Lanes 1, molecular mass standards with size indicated; 2, soluble protein of *E. coli* expressing heterologous genes; 3, soluble protein of *E. coli* (negative control). Triangles indicate as follows: *filled*, L-phenylalanine 4-hydroxylase; *dark gray*, glucose dehydrogenase; *light gray*, dihydropteridine reductase; *open*, pterin-4α-carbinolamine dehydratase.

Moreover, GDH has high specific activity and prefers NADP$^+$ over NAD$^+$ (Yun et al. 2005). Similarly, DPR prefers NADPH over NADH. Thus, the genes encoding GDH, DPR, and PAH-L101Y-W180F were coexpressed in *E. coli* BL21(DE3) *tnaA*$^-$. The coexpression of the GDH gene, in addition to the PAH-L101Y-W180F and DPR genes in *E. coli*, led to a 1.6-fold increase in 5-HTP as compared to that for the PAH-L101Y-W180F and DPR genes alone, suggesting that NAD(P)H regeneration stimulated $DMPH_4$ regeneration.

During human pterin regeneration, aromatic amino acid hydroxylation is followed by the conversion of pterin to pterine-4α-carbinolamine. Pterine-4α-carbinolamine is subsequently converted to dihydropteridine, and DPR catalyzes the reduction of dihydropteridine to tetrahydropterin. In this process, PCD catalyzes the dehydration of pterine-4α-carbinolamine to produce dihydropteridine. However, pterine-4α-carbinolamine is affected by a non-enzymatic rearrangement with opening of the pyridine ring, yielding the 7-substituted pterin (Davis et al. 1991; Schallreuter et al. 1994). Additionally, in the absence of PCD, 7-substituted pterin is formed and competes with the natural cofactor tetrahydropterin for PAH, leading to a critically reduced hydroxylation rate (Davis et al. 1991). Therefore, to accelerate $DMPH_4$ regeneration and increase the 5-HTP yield, more PCD was added and the corresponding gene was coexpressed in *E. coli* BL21(DE3) *tnaA*$^-$. The results of this approach indicated a nearly 11-fold (0.74 mM) increase in the synthesis yield of 5-HTP when compared to samples lacking cofactor regeneration (Figure 4).

Optimization of reaction conditions for synthesis of 5-HTP

Enhanced synthesis of 5-HTP was investigated using PAH-L101Y-W180F, and an oxidation-reduction enzyme process for $DMPH_4$ regeneration was established. It was then necessary to optimize the reaction conditions using *E. coli* whole cells with the four enzymes coexpressed in the same *E. coli* cell.

Effects of pH and temperature on 5-HTP synthesis were examined over a range of pH values (pH 5–10) and temperatures (20–50°C). The maximal yield of 5-HTP was obtained at pH 8.0 and 30°C (Figure 5).

Finally, the effects of $DMPH_4$ concentration (0.1–5 mM) on 5-HTP synthesis were determined at optimal pH and temperature. The molar yield of 5-HTP reached 50% when $DMPH_4$ exceeded 3 mM (Figure 6).

Discussion

In the present study, we developed a novel cofactor regeneration system for L-tryptophan hydroxylation to synthesize 5-HTP. In this coupling system of pterin and NAD(P)H regeneration, 5-HTP was synthesized in quantities

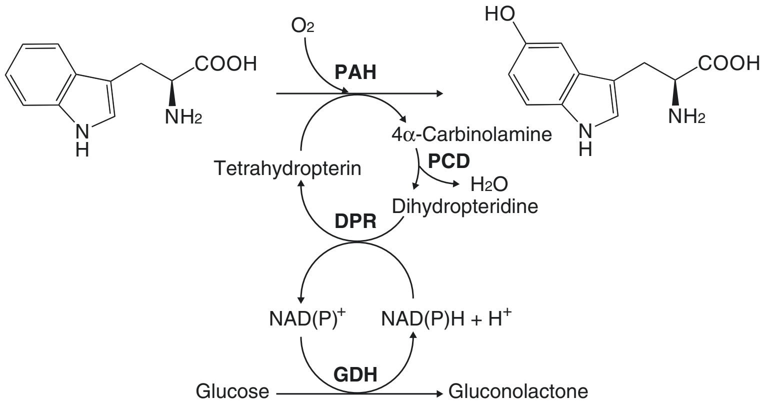

Figure 3 Scheme of 5-HTP synthesis through cofactor regeneration. PAH, L-phenylalanine 4-hydroxylase; PCD, pterin-4α-carbinolamine dehydratase; DPR, dihydropteridine reductase; GDH, glucose dehydrogenase.

exceeding the concentration of the pterin added, which is necessary for L-tryptophan hydroxylation.

We recently used site-directed mutagenesis to engineer CviPAH, which exhibited high L-tryptophan hydroxylation activity. Comparison of the crystal structures of CviPAH with human L-tryptophan 5-hydroxylase led to the hypothesis that L101 and W180 in CviPAH were responsible for cofactor and substrate affinity, respectively, and we consequently engineered the CviPAH L101Y-W180F mutant enzyme (Kino et al. 2009). While higher hydroxylation activity toward L-tryptophan was achieved by CviPAH L101Y-W180F, the cofactor pterin is still essential for the aromatic amino acid hydroxylase reaction, and because pterin is an expensive reagent for industrial applications, there is a demand for a more cost-effective process to provide pterin. To accomplish this, we focused on developing a pterin-regeneration system. Additionally, since human pterin recycling enzymes from the brain and other tissues have been investigated and it is known that NAD(P)H is necessary for pterin regeneration, we hypothesized that if this process could be reconstructed in *E. coli*, L-tryptophan hydroxylation with regeneration of the cofactors pterin and NAD(P)H could be established, leading to efficient 5-HTP synthesis (Figure 3). NAD(P)H can be regenerated using dehydrogenase-coupling processes, including GDH, formate dehydrogenase, and alcohol dehydrogenase; therefore, we utilized GDH from *Bacillus subtilis* to regenerate NAD(P)H and pterin due to its high enzyme activity and stability.

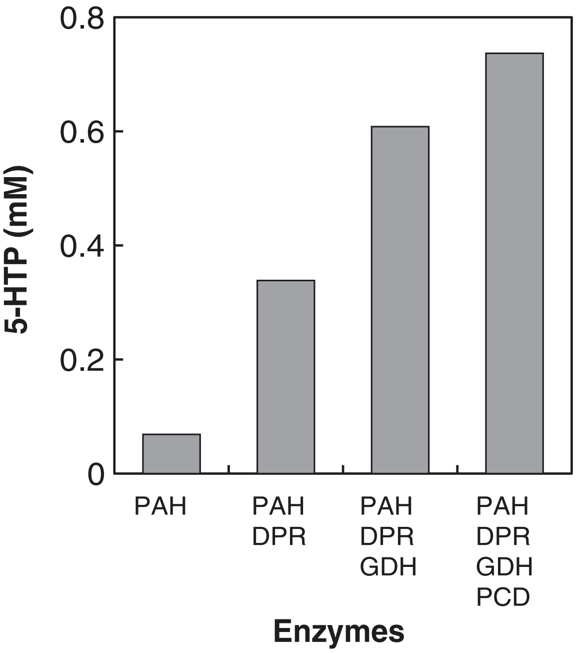

Figure 4 Effects of DMPH$_4$ regeneration enzymes on synthetic yield of 5-HTP using PAH. The basal reaction condition is described in the text. In this experiment, 0.1 mM DMPH$_4$ was applied.

Currently, 5-HTP is produced by extraction from the seeds of the African plant *G. simplicifolia*. Several attempts have been made to synthesize 5-HTP using chemical or enzymatic syntheses; however, these have proven to be unsuitable from an industrial perspective. Therefore, we focused our attention on microbial L-phenylalanine 4-hydroxylase because this enzyme hydroxylates L-tryptophan to 5-HTP, and L-phenylalanine to L-tyrosine. Although engineered CviPAH exhibited high L-tryptophan hydroxylation activity, the enzyme required L-tryptophan and equimolar concentrations of the cofactor pterin, which is an expensive and scarce compound in the industry. Hence, we proposed and constructed a novel cofactor regeneration process coupled with amino acid hydroxylation. Using microbial genome information, we reconstituted three enzymes, PCD, DPR and GDH, for pterin regeneration, and coexpressed the corresponding genes in *E. coli*. When the resulting *E. coli* tryptophanase-

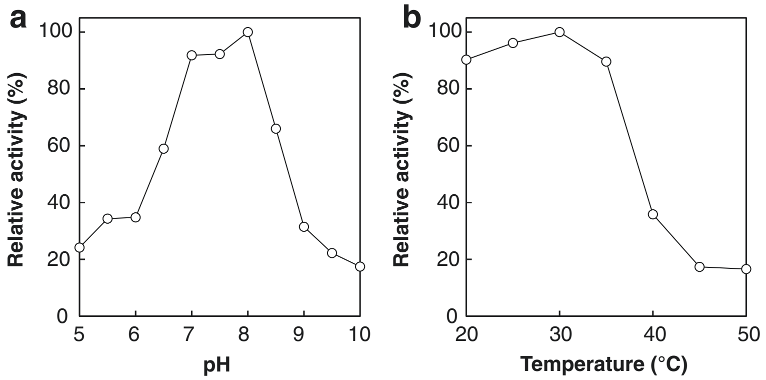

Figure 5 Optimal pH and temperature for the synthesis of 5-HTP, using the *E. coli* whole cell reaction. (a) The reaction conditions, except for pH and buffer, are described in the text. The following buffers were used: acetate buffer, pH 5.0–5.5; maleate buffer, pH 6.0; MOPS-NaOH buffer, pH 6.5; HEPES-NaOH buffer, pH 7.0–8.0; Tris–HCl buffer, pH 8.5; borate-NaOH buffer, pH 9.0–10. **(b)** The reaction conditions, except for temperature, are described in the text.

deficient mutant expressing CviPAH-L101Y-W180F, PCD, DPR, and GDH was used as a whole cell catalyst, higher L-tryptophan hydroxylation activity was observed than that seen with *E. coli* expressing the gene encoding Cvi-PAH alone (Figure 4), suggesting that pterin regeneration was functionally affected.

Indeed, the regeneration process is effective for the production of 5-HTP to some extent, although this approach could not effect conversion of all of the available L-tryptophan into 5-HTP. As shown in Figure 4, the maximum yield of 5-HTP was 0.74 mM in the presence of 0.1 mM $DMPH_4$, suggesting that the total turnover number is 7.4 mole/mole. On the other hand, the total turnover number is less than 1 mole/mole in case of >3 mM $DMPH_4$ addition (Figure 6). Previous studies suggested that GDH should promote further NAD(P)H regeneration; however, complete pterin regeneration was still difficult to achieve, which may be due to instability of the pterin. Several studies have reported that pterin-4α-carbinolamine, an oxidized form of pterin, can spontaneously convert to an undesirable 7-substituted pterin (Curtius et al. 1990a; Curtius et al. 1990b; Davis et al. 1991; Thony et al. 2000). Once this 7-substituted compound is formed, the pterin regeneration cycle is hampered, since the hydroxylase reaction is partially uncoupled (Schallreuter et al. 1994). To avoid the formation of 7-substituted pterin and accelerate pterin regeneration, PCD, or "phenylalanine hydroxylase-stimulating protein" (Hauer et al. 1993; Huang et al. 1973), was supplied to stimulate the regeneration system. While a minor stimulatory effect was observed, only a slight increase in 5-HTP was achieved (Figure 4). This effect is likely due to the unnecessary accumulation of pterin analog such as 7-substituted pterin, and this problem must be resolved in order to achieve complete conversion of L-tryptophan to 5-HTP.

In summary, we report the development of a novel pterin regeneration process for enhanced L-tryptophan hydroxylation. Although the efficiency of pterin regeneration is expected to improve with further enhancements to the regeneration enzymes, this regeneration process represents a powerful tool for practical amino acid hydroxylation using aromatic amino acid hydroxylases, including L-phenylalanine 4-hydroxylase, L-tyrosine 3-hydroxylase, and L-tryptophan 5-hydroxylase.

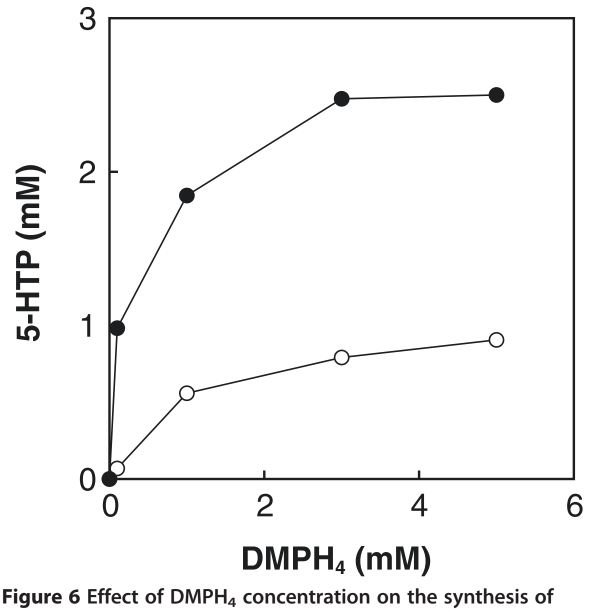

Figure 6 Effect of $DMPH_4$ concentration on the synthesis of 5-HTP, using the *E. coli* whole cell reaction. *Open circles*, L-tryptophan hydroxylation using *E. coli tnaA* disruptant expressing PAH gene alone; *filled circles*, L-tryptophan hydroxylation using *E. coli tnaA* disruptant expressing the genes encoding PAH, PCD, DPR, and GDH.

Competing interests
The authors declare that they have no competing interests.

Acknowledgements
We are grateful to Mr. T. Abe for his valuable technical assistance throughout the experiments.

Received: 2 December 2013 Accepted: 3 December 2013
Published: 9 December 2013

References

Angst J, Woggon B, Schoepf J (1977) The treatment of depression with L-5-hydroxytryptophan versus imipramine. Results of two open and one double-blind study. Arch Psychiatr Nervenkr 224:175–186
Azuma S, Tsunekawa H, Okabe M, Okamoto R, Aiba S (1993) Hyper-production of L-tryptophan via fermentation with crystallization. Appl Microbiol Biotechnol 39:471–476
Bono G, Micieli G, Sances G, Calvani M, Nappi G (1984) L-5HTP treatment in primary headaches - an attempt at clinical-identification of responsive patients. Cephalalgia 4:159–165
Cadenas E, Simic MG, Sies H (1989) Antioxidant activity of 5-hydroxytryptophan, 5-hydroxyindole, and DOPA against microsomal lipid peroxidation and its dependence on vitamin E. Free Radic Res Commun 6:11–17
Curtius HC, Adler C, Rebrin I, Heizmann C, Ghisla S (1990a) 7-Substituted pterins: formation during phenylalanine hydroxylation in the absence of dehydratase. Biochem Biophys Res Commun 172:1060–1066
Curtius HC, Matasovic A, Schoedon G, Kuster T, Guibaud P, Giudici T, Blau N (1990b) 7-Substituted pterins. A new class of mammalian pteridines. J Biol Chem 265:3923–3930
Datsenko KA, Wanner BL (2000) One-step inactivation of chromosomal genes in *Escherichia coli* K-12 using PCR products. Proc Natl Acad Sci USA 97:6640–6645
Davis MD, Kaufman S, Milstien S (1991) Conversion of 6-substituted tetrahydropterins to 7-isomers via phenylalanine hydroxylase-generated intermediates. Proc Natl Acad Sci U S A 88:385–389
Fitzpatrick PF (2000) The aromatic amino acid hydroxylases. Adv Enzymol Relat Areas Mol Biol 74:235–294
Fujisawa H, Nakata H (1987) Phenylalanine 4-monooxygenase from *Chromobacterium violaceum*. Methods Enzymol 142:44–49
Guilleminault C, Cathala JP, Castaigne P (1973) Effects of 5-hydroxytryptophan on sleep of a patient with a brain-stem lesion. Electroencephalogr Clin Neurophysiol 34:177–184
Hauer CR, Rebrin I, Thony B, Neuheiser F, Curtius HC, Hunziker P, Blau N, Ghisla S, Heizmann CW (1993) Phenylalanine hydroxylase-stimulating protein/pterin-4 alpha-carbinolamine dehydratase from rat and human liver. Purification, characterization, and complete amino acid sequence. J Biol Chem 268:4828–4831
Huang CY, Max EE, Kaufman S (1973) Purification and characterization of phenylalanine hydroxylase-stimulating protein from rat liver. J Biol Chem 248:4235–4241
Ikeda M, Katsumata R (1999) Hyperproduction of tryptophan *by Corynebacterium glutamicum* with the modified pentose phosphate pathway. Appl Environ Microbiol 65:2497–2502
Kino K, Hara R, Nozawa A (2009) Enhancement of L-tryptophan 5-hydroxylation activity by structure-based modification of L-phenylalanine 4-hydroxylase from *Chromobacterium violaceum*. J Biosci Bioeng 108:184–189
Lysek N, Kinscherf R, Claus R, Lindel T (2003) L-5-Hydroxytryptophan: antioxidant and anti-apoptotic principle of the intertidal sponge *Hymeniacidon heliophila*. Z Naturforsch C 58:568–572
Nakata H, Yamauchi T, Fujisawa H (1979) Phenylalanine hydroxylase from *Chromobacterium violaceum*. Purification and characterization. J Biol Chem 254:1829–1833
Nakazawa H, Enei H, Okumura S, Yamada H (1972a) Bacterial synthesis of L-tryptophan and its analogs.1. Synthesis of L-tryptophan from pyruvate, ammonia and indole. Agr Biol Chem 36:2523–2528
Nakazawa H, Enei H, Okumura S, Yoshida H, Yamada H (1972b) Enzymatic preparation of L-tryptophan and 5-hydroxy-L-tryptophan. FEBS Lett 25:43–45
Onishi A, Liotta LJ, Benkovic SJ (1991) Cloning and expression of *Chromobacterium violaceum* phenylalanine hydroxylase in *Escherichia coli* and comparison of amino acid sequence with mammalian aromatic amino acid hydroxylases. J Biol Chem 266:18454–18459
Pember SO, Villafranca JJ, Benkovic SJ (1987) *Chromobacterium violaceum* phenylalanine 4-monooxygenase. Methods Enzymol 142:50–56
Persson T, Roos BE (1967) 5-Hydroxytryptophan for depression. Lancet 290:987–988
Ribeiro CA (2000) L-5-Hydroxytryptophan in the prophylaxis of chronic tension-type headache: a double-blind, randomized, placebo-controlled study. For the Portuguese Head Society. Headache 40:451–456
Schallreuter KU, Wood JM, Pittelkow MR, Gutlich M, Lemke KR, Rodl W, Swanson NN, Hitzemann K, Ziegler I (1994) Regulation of melanin biosynthesis in the human epidermis by tetrahydrobiopterin. Science 263:1444–1446
Takahashi S, Kondo H, Kato N (1975) Effect of L-5-hydroxytryptophan on brain monoamine metabolism and evaluation of its clinical effect in depressed patients. J Psychiatr Res 12:177–187
Thony B, Auerbach G, Blau N (2000) Tetrahydrobiopterin biosynthesis, regeneration and functions. Biochem J 347:1–16
van Praag H, de Hann S (1980) Depression vulnerability and 5-hydroxytryptophan prophylaxis. Psychiatry Res 3:75–83
Vasudevan SG, Shaw DC, Armarego WL (1988) Dihydropteridine reductase from *Escherichia coli*. Biochem J 255:581–588
Wang L, Erlandsen H, Haavik J, Knappskog PM, Stevens RC (2002) Three-dimensional structure of human tryptophan hydroxylase and its implications for the biosynthesis of the neurotransmitters serotonin and melatonin. Biochemistry 41:12569–12574
Watanabe T, Snell EE (1972) Reversibility of the tryptophanase reaction: synthesis of tryptophan from indole, pyruvate, and ammonia. Proc Natl Acad Sci U S A 69:1086–1090
Wyatt RJ, Zarcone V, Engelman K, Dement WC, Snyder F, Sjoerdsma A (1971) Effects of 5-hydroxytryptophan on the sleep of normal human subjects. Electroencephalogr Clin Neurophysiol 30:505–509
Yun H, Choi HL, Fadnavis NW, Kim BG (2005) Stereospecific synthesis of (*R*)-2-hydroxy carboxylic acids using recombinant *E. coli* BL21 overexpressing YiaE from *Escherichia coli* K12 and glucose dehydrogenase from *Bacillus subtilis*. Biotechnol Prog 21:366–371
Zhao G, Xia T, Song J, Jensen RA (1994) *Pseudomonas aeruginosa* possesses homologues of mammalian phenylalanine hydroxylase and 4α-carbinolamine dehydratase/DCoH as part of a three-component gene cluster. Proc Natl Acad Sci U S A 91:1366–1370
Zhu MY, Juorio AV (1995) Aromatic L-amino acid decarboxylase: biological characterization and functional role. Gen Pharmacol 26:681–696

doi:10.1186/2191-0855-3-70
Cite this article as: Hara and Kino: **Enhanced synthesis of 5-hydroxy-L-tryptophan through tetrahydropterin regeneration.** *AMB Express* 2013 **3**:70.

Submit your manuscript to a SpringerOpen journal and benefit from:

- ► Convenient online submission
- ► Rigorous peer review
- ► Immediate publication on acceptance
- ► Open access: articles freely available online
- ► High visibility within the field
- ► Retaining the copyright to your article

Submit your next manuscript at ► springeropen.com
