# Supplementary material for: 5-Hydroxytryptophan (5-HTP): Natural Occurrence, Analysis, Biosynthesis, Biotechnology, Physiology and Toxicology
Source: Int J Mol Sci. 2020 Dec 26;22(1):181. doi: 10.3390/ijms22010181 (PMC7796270; doi:10.3390/ijms22010181)
Supplement: Supplementary file 1 [file ijms-22-00181-s001.zip › 5-HTP.Data/PDF/1755330793/Kino-2009-Enhancement-of-l-tryptophan--hydrox.pdf]

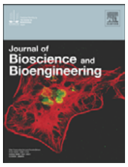

Journal of Bioscience and Bioengineering
VOL. 108 No. 3, 184–189, 2009

www.elsevier.com/locate/jbiosc

Enhancement of L-tryptophan 5-hydroxylation activity by structure-based modification of L-phenylalanine 4-hydroxylase from *Chromobacterium violaceum*

Kuniki Kino,* Ryotaro Hara, and Ai Nozawa

Department of Applied Chemistry, Faculty of Science and Engineering, Waseda University, 3-4-1 Ohkubo, Shinjuku-ku, Tokyo 169-8555, Japan

Received 6 December 2008; accepted 3 April 2009

The objective of this study was to enhance L-tryptophan hydroxylation activity of L-phenylalanine 4-hydroxylase. It had been known that L-phenylalanine 4-hydroxylase from *Chromobacterium violaceum* could convert L-tryptophan to 5-hydroxy-L-tryptophan and L-phenylalanine to L-tyrosine; however, the activity for L-tryptophan was extremely low compared to L-phenylalanine activity levels. We used the information on the crystal structures of aromatic amino acid hydroxylases to generate *C. violaceum* L-phenylalanine 4-hydroxylase with high L-tryptophan hydroxylating activity. *In silico* structural modeling analysis suggested that hydrophobic and/or stacking interactions with the substrate and cofactor at L101 and W180 in *C. violaceum* L-phenylalanine 4-hydroxylase would increase hydroxylation activity. Based on this hypothesis, we introduced a saturation mutagenesis towards these sites followed by the evaluation of 5-hydroxy-L-tryptophan productivity using a modified Gibbs assay. Three and nine positive mutants were obtained from the L101 and W180 mutant libraries, respectively. Among the mutants, L101Y and W180F showed the highest L-tryptophan hydroxylation activity at the respective residues. Steady-state kinetic analysis revealed that k_{cat} values for L-tryptophan hydroxylation were increased from 0.40 (wild-type) to 1.02 (L101Y) and 0.51 s^{-1} (W180F). In addition, the double mutant (L101Y-W180F) displayed higher L-tryptophan hydroxylation activity than the wild-type and the W180F and L101Y mutants. The k_{cat} value of L101Y-W180F increased to 2.08 s^{-1}, showing a 5.2-fold increase compared to wild-type enzyme levels.

© 2009, The Society for Biotechnology, Japan. All rights reserved.

[**Key words:** 5-Hydroxy-L-tryptophan; L-Phenylalanine 4-hydroxylase; Aromatic amino acid hydroxylase; *Chromobacterium violaceum*; Saturation mutagenesis]

L-Phenylalanine 4-hydroxylase (PAH) belongs to the aromatic amino acid hydroxylase family, including PAH (EC 1.14.16.1), tyrosine 3-hydroxylase (TH, EC 1.14.16.2) and tryptophan 5-hydroxylase (TPH, EC 1.14.16.4). These hydroxylases are 5,6,7,8-tetrahydrobiopterin (BH_4) and non-heme iron-dependent enzymes that catalyze hydroxylation of L-phenylalanine, L-tyrosine and L-tryptophan, respectively. L-Phenylalanine 4-hydroxylases exist in many species, ranging from microorganisms to vertebrates, and play a significant role in L-phenylalanine metabolism. On the other hand, TH and TPH are involved in the synthesis of catecholamine and serotonin, respectively.

Since 5-HTP acts as an intermediate in the mammalian serotonin pathway, it has been widely used as a tranquilizer or a sleep-inducing drug. It is generally obtained by extraction from seeds of the African plant *Griffonia simplicifolia*. The extraction method, however, requires many complex purification steps and generates a large amount of waste materials. To overcome these drawbacks, an efficient synthesis method should be explored. In a previous study, 5-HTP was synthesized from 5-hydroxyindole, pyruvate and ammonia using tryptophanase from the *Proteus rettgeri* strain Aj 2770 (1). This enzymatic method is simple and economical; however, 5-hydroxyindole is a highly toxic and harmful compound. It is suggested that PAH from *Chromobacterium violaceum* (CviPAH) can hydroxylate not only the C-4 position of L-phenylalanine but also the C-5 position of L-tryptophan (Fig. 1) (2). Mammalian aromatic amino acid hydroxylases can also hydroxylate aromatic amino acids; however, they are known to be unstable, and the enzyme activities are strictly regulated by their product concentrations (3, 4). These characteristics are not sufficient for practical use. Conversely, bacterial aromatic amino acid hydroxylase is more stable and easier to use than that of the mammalian form. Because of the lack of a regulation domain, CviPAH should be more favorable for industrial application and is expected to be one of the most efficient and cost effective biocatalyst for 5-HTP production. The hydroxylation velocity for L-tryptophan is only 0.4% that of L-phenylalanine, however, which is extremely low in comparison (2).

The X-ray crystal structures of CviPAH (5) and human aromatic amino acid hydroxylases were recently elucidated (6). A mutation study of mammalian aromatic amino acid hydroxylases revealed the role of F313 in human TPH (HumTPH) in substrate specificity (7). Similarly, it was demonstrated that mutation of F313W of the rabbit TPH preferably catalyzes L-phenylalanine hydroxylation rather than L-tryptophan hydroxylation (8).

* Corresponding author. Tel.: +81 3 5286 3211; fax: +81 3 3232 3889.
E-mail address: kkino@waseda.jp (K. Kino).

1389-1723/$ - see front matter © 2009, The Society for Biotechnology, Japan. All rights reserved.
doi:10.1016/j.jbiosc.2009.04.002

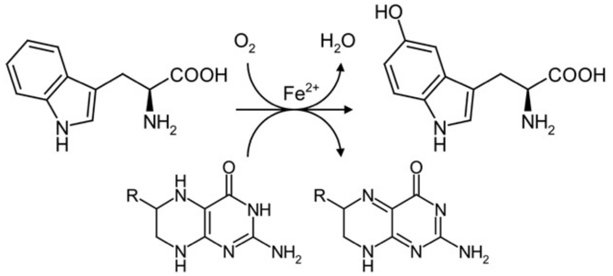

FIG. 1. Synthesis of 5-hydroxy-L-tryptophan using *C. violaceum* L-phenylalanine 4-hydroxylase.

Here, we enhanced L-tryptophan hydroxylation activity of CviPAH using information on the crystal structures of aromatic amino acid hydroxylases including CviPAH, human PAH (HumPAH) and HumTPH. Based on the tertiary structures of these hydroxylases, we performed saturation mutagenesis of two residues (L101 and W180) that were expected to interact with cofactor and substrate specificity, respectively. Colorimetric screening following construction of the mutant library revealed that several mutations yielded higher L-tryptophan hydroxylation activity than the wild-type enzyme. Among them, unique mutants that preferred L-tryptophan hydroxylation to L-phenylalanine hydroxylation were obtained.

MATERIALS AND METHODS

Chemicals and materials 6,7-Dimethyl-5,6,7,8-tetrahydropterine hydrochloride ($DMPH_4$) and catalase from bovine liver were purchased from Sigma (St. Louis, MO, USA). The Protino Ni-TED 2000 column was purchased from Macherey-Nagel (Düren, Germany). The PD-10 column was obtained from GE Healthcare (Piscataway, NJ, USA). All other chemicals and materials used were of the highest purity grade available and obtained from commercial sources.

C. violaceum NBRC 12614^T was obtained from the National Institute of Technology and Evaluation (Chiba, Japan). *Escherichia coli* JM109 (Nippon Gene, Tokyo, Japan) and BL21(DE3) (Novagen, Madison, WI, USA) were used as the hosts for gene cloning and expression, respectively.

Computational analysis Amino acid sequences were aligned by ClustalW (version 1.83) multiple-sequencing alignment program on the DNA Data Bank of Japan. The crystal structures of aromatic amino acid hydroxylases were retrieved from the Protein Data Bank (PDB), and the PDB codes were follows: CviPAH, 1LTZ; HumPAH, 1DMW; and HumTPH, 1MLW. Viewing and manipulation of three-dimensional structures were carried out using PyMOL (http://pymol.sourceforge.net/).

DNA manipulation The chromosomal DNA of *C. violaceum* was prepared using DNeasy Tissue kits (Qiagen, Hilden, Germany) according to the manufacturer's instructions. The CviPAH gene was amplified with KOD-plus DNA polymerase (Toyobo, Osaka, Japan) using the following primer set: WT-F (5′-TCATTATCATATGAACGACCGCG-CCGACTTTG-3′) and WT-R (5′-AGCTTCTCGAGGACGTCTTCGGTATCCGCCC-3′). Subsequently, the amplified gene was cloned into NdeI and XhoI sites of pET-21a(+) (Novagen) to yield the plasmid pEPAH-wt carrying the wild-type CviPAH gene.

Site-directed mutagenesis experiments were carried out using QuickChange site-directed mutagenesis kit (Stratagene, La Jolla, CA, USA). A gene library encoding all possible amino acids at amino acid position L101 and W180 of the CviPAH was constructed by replacing the target codon with NNN (where N was A, T, G or C). The introduction of the single amino acid mutation in the CviPAH gene was achieved using pEPAH-wt as the template DNA together with the following mutagenic oligonucleotide primer sets: L101X-F (5′-TGCCGGGCNNNATTCCAGACGACG-3′) and L101X-R (5′-TGGAATNNNGCCCGGCACCGCGACG-3′); W180X-F (5′-CTGTACNNNTACACGGTGGAA-TTCGGCCTGATC-3′) and W180X-R (5′-CGTGTANNNGTACAGCCGCGCCAGCATC-3′). The double amino acid mutation in the CviPAH gene was introduced using pEPAH-W180F as a template DNA, which carried the W180F mutation in the CviPAH gene, together with the primer set of W101Y-F (5′-TGCCGGGCTATATTCCAGACGACG-3′) and W101Y-R (5′-TGGAATATAGCCCGGCACCGCGACG-3′). The resulting plasmid was introduced into *E. coli* JM109 separately by the $CaCl_2$ method (9). All mutations were then verified by DNA sequencing. The obtained mutant plasmid was introduced into *E. coli* BL21 (DE3) for gene expression.

Screening procedure Each colony of the mutant library was picked into 500 μl of LB medium containing 50 μg/ml ampicillin. The cultures were incubated overnight at 37 °C with vigorous shaking. A total of 20 μl of overnight culture was inoculated into 2 ml of the same medium with 0.1 mM isopropyl-β-D-thiogalactopyranoside (IPTG). The cultures were incubated at 25 °C for 8 h with vigorous shaking. The cells were then centrifuged at 3000 ×*g* for 10 min at 4 °C, washed twice in 100 mM HEPES-NaOH buffer (pH 7.5), and the cell pellet was resuspended to a final optical density at 660 nm ($O.D._{660}$) of approximately 10 with a reaction mixture containing 5 mM L-tryptophan, 1 mM $DMPH_4$, 0.1 mM $FeSO_4$, 0.5% (v/v) Triton X-100 and the same buffer in a total volume of 200 μl. The reaction was carried out at 30 °C for 1 h with vigorous shaking. After the reaction, resting cells were removed by centrifugation at 20,000 ×*g* for 10 min at 4 °C, and the supernatant was subjected to the following assay.

L-Tryptophan hydroxylation activities were determined with a modified Gibbs assay (10) according to the following protocol: 50 μl of 500 mM borate-NaOH buffer (pH 9) were added to 50 μl of the supernatant described above, followed by the addition of 2 μl of 0.5% (w/v) 2,6-dichloroquinone-4-chloroimide (Gibbs' reagent) ethanol solution. After incubating for 30 min at room temperature, absorbance at 580 nm was measured for colored products resulting from 5-HTP, followed by coupling with Gibbs' reagent using the Ultraspec 3300 pro spectrophotometer (GE Healthcare).

Protein purification and SDS-PAGE analysis *E. coli* BL21(DE3) harboring each recombinant plasmid was cultivated in LB medium containing 50 μg/ml ampicillin with routine shaking at 37 °C until $O.D._{660}$ reached 0.5. Subsequently, IPTG was added to the culture to the final concentration of 0.1 mM, and the cells were continuously cultivated at 25 °C for 20 h. After cultivations, *E. coli* BL21(DE3) cells harboring recombinant plasmid were harvested by centrifugation at 5000 ×*g* for 10 min at 4 °C. The cell pellet was resuspended in 3 ml of 20 mM phosphate buffer (pH 7.5) containing 300 mM NaCl. After ultrasonic disruption of the cells, a crude extract was obtained by centrifugation at 20,000 ×*g* for 10 min at 4 °C. Since all CviPAH genes were overexpressed in *E. coli* BL21 (DE3) as C-terminal His×6 tagged proteins, the enzymes were purified by using Protino Ni-TED 2000 column according to the manufacturer's recommendations. Thereafter, imidazole and NaCl were removed using a PD-10 desalting column and the buffer was exchanged with 100 mM HEPES buffer (pH 7.5). The purified enzyme concentrations were determined by the Bradford method with bovine serum albumin serving as the standard (11). The obtained wild-type and mutant CviPAH were analyzed by sodium dodecyl sulfate-polyacrylamide gel electrophoresis (SDS-PAGE). Proteins were visualized by staining with CBB Stain One (Nacalai Tesque, Kyoto, Japan).

Determination of steady-state kinetic parameters of wild-type and mutant CviPAHs Activity of CviPAH was measured at 30 °C in a total volume of 500 μl of reaction mixture containing variable concentrations of L-tryptophan (1–21 mM) or L-phenylalanine (0.04–1 mM), $DMPH_4$ (0.02–2 mM), 0.1 mM $FeSO_4$, 500 U catalase, 5 mM dithiothreitol and 50 mM HEPES-NaOH buffer (pH 7.5). Steady-state kinetic parameters were obtained by measuring the activity with various concentrations of L-tryptophan (1–21 mM), L-phenylalanine (0.04–1 mM) and $DMPH_4$ (0.02–2 mM).

The reaction was initiated by adding the enzyme and performed at 30 °C for 10 min with reciprocal shaking. After the reaction was terminated by the addition of 50% (v/v) methanol, the amount of synthesized 5-HTP or L-tyrosine was measured using HPLC. The kinetic parameters were calculated using a Hanes-Woolf plot. One unit of the activity was defined as the amount of enzyme that catalyzed the hydroxylation of amino acid to synthesize 1 μM of the product per minute at 30 °C.

HPLC analysis The amount of 5-HTP or L-tyrosine was determined by the post-column derivatization method with *o*-phthalaldehyde (OPA) using the L-2000 series HPLC system (Hitachi, Tokyo, Japan). The HPLC system was equipped with an Intertsil ODS-3 column (4.6 mm i.d.×150 mm; GL Sciences, Tokyo, Japan). Chromatographic conditions were follows: a mobile phase containing 20 mM phosphate buffer (pH 2.5) and 10% (v/v) methanol was used, and the flow rate was maintained at 0.7 ml/min. The column temperature was kept at 40 °C. The injection volume of the enzyme reaction mixture was 10 μl. After elution through the column, 400 mM borate buffer (pH 9) containing 0.2% (v/v) 2-mercaptoethanol and 0.8 g/l OPA was mixed with a mobile phase. The flow rate of the OPA solution was maintained at 0.3 ml/min. The OPA derivatization mixture was kept at 25 °C for a few minutes to derivatize separated amino acids. The OPA-derivatized amino acid in the reaction mixture was detected spectrofluorometrically with an excitation wavelength of 340 nm and an emission wavelength of 450 nm.

RESULTS

Structural modeling analysis of amino acid hydroxylases Amino acid sequences of aromatic amino acid hydroxylases were aligned using the ClustalW program. Multiple alignments of CviPAH and the catalytic domain of mammalian aromatic amino acid hydroxylases are illustrated in Fig. 2. In this alignment, a consensus sequence, biopterin-dependent aromatic amino acid hydroxylases signature (underlined), was conserved. Furthermore, the 2-His-1-carboxylate facial triad (12), which is involved in the iron-binding motif, was observed (open triangles in Fig. 2). There were no significant regions that were conserved in the N-terminal or C-terminal regions between CviPAH and mammalian aromatic amino acid hydroxylases. This is because the N-terminal region is involved in the regulation of enzyme activity and C-terminal region is involved in oligomerization of enzymes in the mammalian aromatic amino acid hydroxylases (13). These two regions are characteristic of mammalian aromatic amino acid hydroxylases.

```
CviPAH     1 MNDRADFVVPDITTRKNVGLSHDANDFTLPQPLDR--YSAEDHATWATLYQRQCKLLPGR
HumPAH   143 DADHPGFKDPVYRARRKQ-FADIAYNYRHGQPIPRVEYMEEEKKTWGTVFKTLKSLYKTH
RatPAH   143 DADHPGFKDPVYRARRKQ-FADIAYNYRHGQPIPRVEYTEEEKQTWGTVFRTLKALYKTH
HumTPH   130 DADHPGFKDNVYRKRRKY-FADLAMNYKHGDPIPKVEFTEEEIKTWGTVFQELNKLYPTH
RatTPH   130 DADHPGFKDNVYRRRRKY-FAELAMNYKHGDPIPKIEFTEEEIKTWGTIFRELNKLYPTH
               *   *       *        *       *        *   ** *       *

CviPAH    59 ACDEFMEGLERLEVDA----DRVPDFNKLNQKLMAATGWKIVAVPGLIPDDVFFEHLANR
HumPAH   202 ACYEYNHIFPLLEKYCGFHEDNIPQLEDVSQFLQTCTGFRLRPVAGLLSSRDFLGGLAFR
RatPAH   202 ACYEHNHIFPLLEKYCGFREDNIPQLEDVSQFLQTCTGFRLRPVAGLLSSRDFLGGLAFR
HumTPH   189 ACREYLKNLPLLSKYCGYREDNIPQLEDVSNFLKERTGFSIRPVAGYLSPRDFLSGLAFR
RatTPH   189 ACREYLRNLPLLSKYCGYREDNVPQLEDVSNFLKERTGFSIRPVAGYLSPRDFLSGLAFR
             ** *       *        *  *        *   **     * *      *   ** *

CviPAH   115 RFPVTWWLREPHQLDYLQEPDVFHDLFGHVPLLINPVFADYLEAYGKGGVKAKALGALPM
HumPAH   262 VFHCTQYIRHGSKPMYTPEPDICHELLGHVPLFSDRSFAQFSQEIGLASLGAPDE-YIEK
RatPAH   262 VFHCTQYIRHGSKPMYTPEPDICHELLGHVPLFSDRSFAQFSQEIGLASLGAPDE-YIEK
HumTPH   249 VFHCTQYVRHSSDPFYTPEPDTCHELLGHVPLLAEPSFAQFSQEIGLASLGASEE-AVQK
RatTPH   249 VFHCTQYVRHSSDPLYTPEPDTCHELLGHVPLLAEPSFAQFSQEIGLASLGASEE-TVQK
              *  *   *      *  ***  * * *****     **      *     *

CviPAH   175 LARLYWYTVEFGLINTPAGMRIYGAGILSSKSESIYCLDSASPNRVGFDLMRIMNTRYRI
HumPAH   321 LATIYWFTVEFGLCKQGDSIKAYGAGLLSSFGELQYCLSEK-PKLLPLELEKTAIQNYTV
RatPAH   321 LATIYWFTVEFGLCKEGDSIKAYGAGLLSSFGELQYCLSDK-PKLLPLELEKTACQEYSV
HumTPH   308 LATCYFFTVEFGLCKQDGQLRVFGAGLLSSISELKHALSGH-AKVKPFDPKITCKQECLI
RatTPH   308 LATCYFFTVEFGLCKQDGQLRVFGAGLLSSISELRHALSGH-AKVKPFDPKVACKQECLI
             **  *  ******          *** ***  *    *

CviPAH   235 DTFQKTYFVIDSFKQLFDAT-----------APDFAPLYLQLADAQPWGAGDVAPDDLVL
HumPAH   380 TEFQPLYYVAESFNDAKEKVRNFAATIPRPFSVRYDPYTQRIEVLDNTQQLKILADSINS
RatPAH   380 TEFQPLYYVAESFSDAKEKVRTFAATIPRPFSVRYDPYTQRVEVLDNTQQLKILADSINS
HumTPH   367 TTFQDVYFVSESFEDAKEKMREFTKTIKRPFGVKYNPYTRSIQILKDTKSITSAMNELQH
RatTPH   367 TSFQDVYFVSESFEDAKEKMREFAKTVKRPFGVKYNPYTQSIQVLRDSKSITSAMNELRH
               **  * *  **                       *

CviPAH   284 NAGDRQGWADTEDV----
HumPAH   440 EIGILCSALQKIK-----
RatPAH   440 EVGILCNALQKIKS----
HumTPH   427 DLDVVSDALAKVSRKPSI
RatTPH   427 DLDVVNDALARVSRWPSV
```

FIG. 2. Alignment of amino acid sequences for aromatic amino acid hydroxylases. The amino acid sequence of *C. violaceum* L-phenylalanine 4-hydroxylase (CviPAH) is aligned with those of the catalytic domains of human L-phenylalanine 4-hydroxylase (HumPAH) and rat L-phenylalanine 4-hydroxylase (RatPAH), human L-tryptophan 5-hydroxylase (HumTPH) and rat L-tryptophan 5-hydroxylase (RatTPH). The biopterin-dependent aromatic amino acid hydroxylase signature is underlined and the 2-His-1-carboxylate facial triad (iron-binding motif) conserved within the pterin-dependent hydroxylase family is indicated by open inverted triangles. Closed inverted triangles indicate the residue targets for saturation mutagenesis. Asterisks indicate amino acid residues that conserved in all five sequences.

The three-dimensional structural modeling around the active sites of CviPAH, HumPAH and HumTPH are shown in Fig. 3. The H138, H143 and E184 regions in CviPAH corresponded to the H285, H290 and E330 regions in HumPAH and H272, H277 and E317 regions in HumTPH. These residues were strictly conserved because they coordinate the iron center in all aromatic amino acid hydroxylases. On the other hand, the residue lying adjacent to substrate of CviPAH and that of HumPAH were W180 and W326, respectively, whereas the residue in HumTPH was F313. These residues were apparently different from PAH and TPH, and thus could be involved in the recognition of the substrate. Moreover, the pterin binding site of CviPAH and that of HumPAH were L101 and L248, respectively. Conversely, the pterin binding site of HumTPH was Y235. These residues were also apparently different between PAH and TPH, and thus could play an important role in pterin recognition. The L101 and W180 sites in CviPAH were therefore selected as targets for mutagenesis study (closed triangles in Fig. 2).

Saturation mutagenesis of CviPAH Two active site residues in CviPAH, L101 and W180, were predicted using the comparison with tertiary structures of CviPAH, HumPAH and HumTPH. Saturation mutagenesis in the CviPAH gene corresponding to L101 and W180 was subsequently carried out to construct a mutant enzyme library, followed by the screening of the beneficial mutant enzyme for L-tryptophan hydroxylation. To screen for positive mutants, we selected a colorimetric method for the monitoring of 5-HTP formations using a modified Gibbs assay (10). After L-tryptophan hydroxylating reaction using resting cells as biocatalysts, the cells were removed by centrifugation and the supernatant was subjected to a colorimetric assay. UV–visible absorbance spectrum for the colored product resulting from the 5-HTP formation followed by coupling with Gibbs' reagent is shown in Fig. 4. There was a linear relationship between absorbance at 580 nm and 5-HTP concentration, indicating that the absorbance reflected the relative amount of 5-HTP.

Using the Gibbs' reagent-based screening method described above, three positive clones (L101M, L101F and L101Y) from the L101 mutant library and nine positive clones (W180R, W180H, W180M, W180C, W180V, W180L, W180I, W180F and W180Y) from the W180 mutant library were obtained to test the improved activity of L-tryptophan

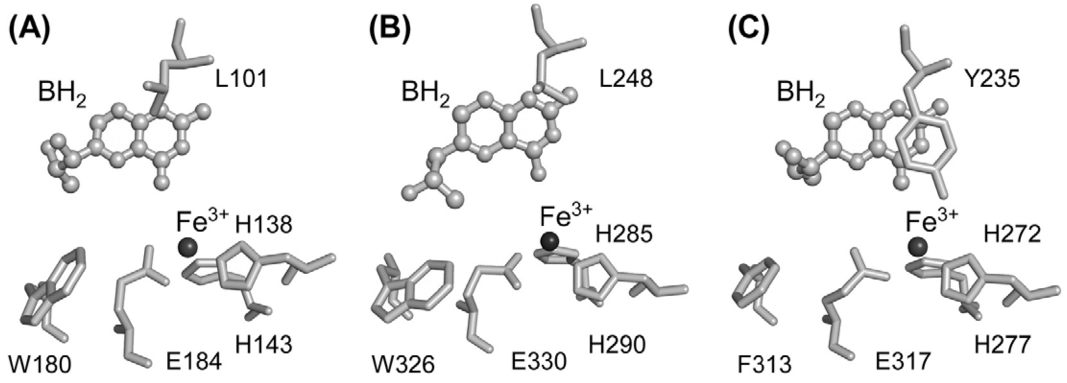

FIG. 3. Active sites of aromatic amino acid hydroxylases. (A) CviPAH (PDB code: 1LTZ), (B) HumPAH (1DMW) and (C) HumTPH (1MLW). Active site residues at the ligand-binding sites are shown as sticks, and 7,8-dihydrobiopterin (BH_2) is shown as a sphere and stick. The ferric iron is shown in dark gray.

hydroxylation (Table 1). Among the positive mutants, L101Y and W180F showed the most improved activity of L-tryptophan hydroxylation at each amino acid position.

SDS-PAGE analysis of mutant enzymes To confirm the improved activity of L-tryptophan hydroxylation caused by catalytic activity, the expression levels of L101Y and W180F were compared to that of the wild-type enzyme using SDS-PAGE analysis. No apparent differences between mutant and wild-type enzyme (34 kDa) were observed (Fig. 5). Thus, the enhanced activity of L-tryptophan hydroxylation was most likely not a result of the improvement in expression levels or solubility. After Ni^{2+} affinity chromatography, the enzymes were purified to near homogeneity in the SDS-PAGE analysis. The purified enzymes were then subjected to the experiments described below.

Enzymatic properties of mutant enzymes The L101Y and W180F residues showed the most enhanced L-tryptophan hydroxylation activity among the single mutations at L101 and W180, respectively. We therefore investigated the hydroxylase activities for L-tryptophan and L-phenylalanine in detail, both individually and within the same experiment. Although the Gibbs assay is a simple and rapid screening method, it was difficult to obtain the kinetic parameters of wild-type and mutant enzymes because of the lack of sensitivity in trace amounts of alteration. We then chose HPLC analysis to determine the substrate and product amino acids for the measurement of the initial velocity of the enzymes. In our preliminary experiment, HPLC co-elution with authentic 5-HTP and L-tyrosine was employed to identify the product, suggesting that no other regioisomers were detected. In addition, the mutations had no effect on the regioselectivity of the enzyme, because except for signal intensity, no changes in the chromatogram pattern were observed.

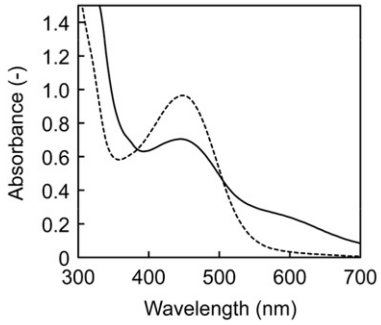

FIG. 4. UV–visible absorption spectrum of colored product resulting from a Gibbs assay for 5-hydroxy-L-tryptophan with *E. coli* resting cells. The solid curve indicates *E. coli* harboring wild-type CviPAH gene expression vector and the dashed curve indicates *E. coli* harboring empty vector.

In the hydroxylation of L-tryptophan, kinetic parameters were obtained by measuring the activity with various concentrations of L-tryptophan (1–21 mM) and 1.25 mM $DMPH_4$. All mutants showed higher velocity compared to the wild-type enzyme (Table 2). Of these mutants, L101Y-W180F showed the highest velocity in this reaction at a 5.2-fold increase compared to the wild-type enzyme. Only W180F showed a lower K_m value compared to wild-type. Moreover, L101Y had a K_m value similar to that of the wild-type enzyme. In contrast, however, L101Y-W180 showed a slight increase in the K_m value. In addition, kinetic parameters of the mutant enzymes with $DMPH_4$ were obtained by measuring the activity with 5 mM L-tryptophan and various concentrations of $DMPH_4$ (0.02–2 mM). L101Y showed a 2.9-fold increase in velocity compared to the wild-type enzyme (Table 3); however, W180F was not affected. Moreover, the velocity of L101Y-W180F for $DMPH_4$ was much higher than L101Y and the wild-type enzyme.

In the hydroxylation of L-phenylalanine, kinetic parameters were obtained by measuring the activity with various concentrations of L-phenylalanine (0.04–1 mM) and 1.25 mM $DMPH_4$. Since a serious substrate inhibition was observed for L-phenylalanine at concentration above 2 mM (data not shown), L-phenylalanine was maintained at 1 mM or less. Only L101Y showed a three-fold increase in velocity compared to the wild-type enzyme (Table 2). The velocity of W180F decreased from 3367 to 307 mU mg^{-1}, suggesting an alteration of

TABLE 1. Relative L-tryptophan hydroxylation activity of CviPAH mutant.

Residue	Relative activity (%)[a]	
	L101	W180
W	55	100
Y	153	115
F	133	204
L	100	174
I	51	113
V	26	155
M	102	166
C	47	119
Q	30	17
N	15	49
A	26	66
S	28	46
T	26	44
G	20	8
P	9	15
D	5	3
E	9	6
K	29	4
R	29	85
H	16	73

[a] The activity of wild-type enzyme is defined as 100%.

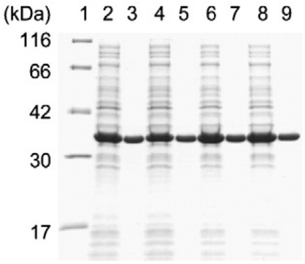

FIG. 5. Purification of His×6 tagged wild-type and mutant CviPAHs from recombinant *E. coli*. Cell-free extracts and purified CviPAHs were loaded on SDS-polyacrylamide gel and stained with Coomassie brilliant blue after electrophoresis. Lanes: 1, molecular mass standards; lanes 2 and 3, wild-type enzymes; lanes 4 and 5, L101Y mutant enzymes; lanes 6 and 7, W180F mutant enzymes; lanes 8 and 9, L101Y-W180F enzymes. Lanes 2, 4, 6 and 8 indicate cell-free extracts from each recombinant *E. coli*, and lanes 3, 5, 7 and 9 indicate purified enzymes.

substrate preference. Although L101Y-W180F showed a lower velocity than the wild-type enzyme and L101Y, it showed higher activity compared to W180F.

DISCUSSION

We have demonstrated saturation mutagenesis of L101 and W180 in CviPAH followed by a rapid screening and obtained 12 mutant CviPAHs with higher L-tryptophan hydroxylation activity than the wild-type enzyme. By comparing amino acid sequences and tertiary structures of aromatic amino acid hydroxylases, several considerable residues were identified. The iron-binding sites (H138, H143 and E184 in CviPAH) were completely conserved (Fig. 2), as these residues are essential for iron coordination. They are a common structural motif that binds the divalent iron center, defined as the 2-His-1-carboxylate facial triad (12). This motif is commonly observed among non-heme iron-dependent oxygenases, for example, extradiol-cleaving catechol dioxygenases (14), Rieske dioxygenases (15), 2-oxoglutarate dependent dioxygenases (16) and pterin-dependent hydroxylases (17). According to structural modeling and comparison of aromatic amino acid hydroxylases, L101 in CviPAH would be involved in cofactor binding, because L101 was located within 4 Å from the cofactor analog 7,8-dihydrobiopterin (BH_2), which is the oxidized form of the natural cofactor BH_4 (5). This residue was the conserved L248 in HumPAH. The corresponding residue was Y235 in HumTPH. We predicted that the substitution of L101 in CviPAH could influence catalytic activity. Although the structure of the natural substrate-enzyme complex has not been elucidated, W180 in CviPAH could be involved as a substrate binding site, similar to W326 in HumPAH (3). Thus, we investigated the role of L101 and W180 in hydroxylation activity by saturation mutagenesis and kinetic analysis.

When BH_2 and the iron bounded forms of the tertiary structure of CviPAH and that of HumTPH were compared, L101 and L248 were located around BH_2 in CviPAH and HumPAH, respectively (Fig. 3). Apparent tight interactions between the leucine residue and BH_2, however, were not observed. In contrast, Y235 in HumTPH was parallel to BH_2, thereby a π–π stacking interaction between Y235 and BH_2 was assumed and the enzyme–cofactor complex could be stabilized and orientated, resulting in a high affinity with a cofactor in HumTPH. Recently, the increase in the K_m value for L-tryptophan is reported in mutant Y235A and Y235L of rabbit TPH (18). Furthermore, mutant Y235A and Y235L of HumTPH also exhibited a decrease in the K_m value for BH_4, which can in turn affect the apparent affinity for L-tryptophan (7). These findings are consistent with our findings for the properties of L101Y mutant, as hydroxylation activities were enhanced for both L-tryptophan and L-phenylalanine using this residue. In fact, the K_m value of L101Y mutant for cofactor $DMPH_4$ was decreased, resulting in an overall improvement in enzyme activity (Table 3).

TABLE 3. Steady-state kinetic parameters of wild-type and mutant CviPAH for cofactor.

Enzyme	Specific activity (mU mg^{-1} protein)	K_m (mM)	k_{cat} (s^{-1})	k_{cat}/K_m (s^{-1} mM^{-1})	Relative k_{cat}/K_m
Wild-type	347	0.140	0.20	1.41	1.0
L101Y	1000	0.099	0.57	5.73	4.0
W180F	342	0.035	0.20	5.80	4.1
L101Y-W180F	1050	0.086	0.60	6.98	5.0

The natural substrate-bound form of X-ray crystal structure has yet to be elucidated; however, it was reported that the interaction between L-tryptophan and F313 in HumTPH using NMR spectroscopy (7). From the results of mutation study, it was predicted that F313 would stabilize and orientate L-tryptophan suitably by a π–π stacking interaction (7). Thus, we hypothesized that the W180F mutation in CviPAH would induce the same effect that would be favorable for the hydroxylation of L-tryptophan due to π–π stacking interactions and volume effects. Similarly, the F313W mutation of rabbit TPH decreased the K_m value for L-tryptophan and increased the K_m value for L-phenylalanine (8). While the origins are different than those employed here, this result is consistent with our mutation study. In fact, as shown in Table 2, the reactive preference for L-tryptophan and L-phenylalanine was switched in W180F. This result strongly supports the discrimination, or the substrate specificity, of substrates in the aromatic amino acid hydroxylase. In addition, W180F mutant exhibits specific activity of the 893 mU mg^{-1} protein with L-tryptophan as the substrate, indicating that it shows higher L-tryptophan hydroxylation activity compared to the W326F mutant of HumPAH (211 mU mg^{-1}protein) or wild-type HumTPH (240 mU mg^{-1} protein) (7).

When L101Y mutation was combined with W180F mutation in CviPAH, a further improvement of L-tryptophan hydroxylation activity was observed compared to the corresponding single mutants. Steady-state kinetic analysis revealed that hydroxylation velocity for L-tryptophan was increased from 690 to 3623 mU mg^{-1} protein and that for L-phenylalanine was decreased from 3370 to 1000 mU mg^{-1} protein in L101Y-W180F mutant. This result indicates that the double mutation effect is additive or synergistic for L-tryptophan hydroxylation. The effect of L101 and W180 mutations on substrate and/or cofactor affinity was also revealed;

TABLE 2. Steady-state kinetic parameters of wild-type and mutant CviPAH for amino acid.

Enzyme	Substrate	Specific activity (mU mg^{-1} protein)	K_m (mM)	k_{cat} (s^{-1})	k_{cat}/K_m (s^{-1} mM^{-1})	Relative k_{cat}/K_m
Wild-type	L-Tryptophan	690	3.44	0.40	0.12	1.0
	L-Phenylalanine	3370	0.111	1.93	17.4	1.0
L101Y	L-Tryptophan	1770	3.54	1.02	0.29	2.5
	L-Phenylalanine	10160	0.478	5.83	12.2	0.7
W180F	L-Tryptophan	893	1.00	0.51	0.51	4.4
	L-Phenylalanine	307	0.033	0.18	5.44	0.3
L101Y-W180F	L-Tryptophan	3620	4.06	2.08	0.51	4.4
	L-Phenylalanine	1000	0.253	0.57	2.25	0.1

however, the influence of the mutations on molecular oxygen affinity could not be elucidated.

In conclusion, we successfully enhanced the L-tryptophan hydroxylation activity of CviPAH based on its tertiary structure. To our best knowledge, no other microbial hydroxylase is reported to exhibit such high L-tryptophan hydroxylation activity. Structural comparison and experimental evidence suggest that L101 and W180 in CviPAH play an important role in cofactor and substrate specificity, respectively. Cofactor supplementation or regeneration is one of the most difficult problems encountered in industrial production of 5-HTP, as it is thought to be recycled by coupling with a reductase. The obtained double mutant in this study, L101Y-W180F, would be helpful to the practical bioconversion process to produce 5-HTP from L-tryptophan without the formation of regioisomers.

ACKNOWLEDGMENTS

This work was financially supported in part by the Global COE program of the MEXT “Center for Practical Chemical Wisdom” and in part by Grant-in-Aid for Scientific Research (B) 18360401.

References

1. **Nakazawa, H., Enei, H., Okumura, S., Yoshida, H., and Yamada, H.:** Enzymatic preparation of L-tryptophan and 5-hydroxy-L-tryptophan, FEBS Lett., **25**, 43–45 (1972).
2. **Nakata, H., Yamauchi, T., and Fujisawa, H.:** Phenylalanine hydroxylase from *Chromobacterium violaceum*, purification and characterization, J. Biol. Chem., **254**, 1829–1833 (1979).
3. **Erlandsen, H., Bjorgo, E., Flatmark, T., and Stevens, R. C.:** Crystal structure and site-specific mutagenesis of pterin-bound human phenylalanine hydroxylase, Biochemistry, **39**, 2208–2217 (2000).
4. **Bjørgo, E., Knappskog, P. M., Martinez, A., Stevens, R. C., and Flatmark, T.:** Partial characterization and three-dimensional-structural localization of eight mutations in exon 7 of the human phenylalanine hydroxylase gene associated with phenylketonuria, Eur. J. Biochem., **257**, 1–10 (1998).
5. **Erlandsen, H., Kim, J. Y., Patch, M. G., Han, A., Volner, A., Abu-Omar, M. M., and Stevens, R. C.:** Structural comparison of bacterial and human iron-dependent phenylalanine hydroxylases: similar fold, different stability and reaction rates, J. Mol. Biol., **320**, 645–661 (2002).
6. **Wang, L., Erlandsen, H., Haavik, J., Knappskog, P. M., and Stevens, R. C.:** Three-dimensional structure of human tryptophan hydroxylase and its implications for the biosynthesis of the neurotransmitters serotonin and melatonin, Biochemistry, **41**, 12569–12574 (2002).
7. **McKinney, J., Teigen, K., Froystein, N. A., Salaun, C., Knappskog, P. M., Haavik, J., and Martinez, A.:** Conformation of the substrate and pterin cofactor bound to human tryptophan hydroxylase, important role of Phe313 in substrate specificity, Biochemistry, **40**, 15591–15601 (2001).
8. **Daubner, S. C., Moran, G. R., and Fitzpatrick, P. F.:** Role of tryptophan hydroxylase Phe313 in determining substrate specificity, Biochem. Biophys. Res. Commun., **292**, 639–641 (2002).
9. **Sambrook, J. and Russel, D. W.:** Molecular Cloning: A Laboratory Manual, 3rd ed. Cold Spring Harbor Laboratory Press, Cold Spring Harbor, N.Y., 2001
10. **Quintana, M. G., Didion, C., and Dalton, H.:** Colorimetric method for a rapid detection of oxygenated aromatic biotransformation products, Biotechnol. Techniques, **11**, 585–587 (1997).
11. **Bradford, M. M.:** A rapid and sensitive method for the quantitation of microgram quantities of protein utilizing the principle of protein-dye binding, Anal. Biochem., **72**, 248–254 (1976).
12. **Koehntop, K. D., Emerson, J. P., and Que Jr., L., Jr.:** The 2-His-1-carboxylate facial triad: a versatile platform for dioxygen activation by mononuclear non-heme iron (II) enzymes, J. Biol. Inorg. Chem., **10**, 87–93 (2005).
13. **Erlandsen, H., Patch, M. G., Gamez, A., Straub, M., and Stevens, R. C.:** Structural studies on phenylalanine hydroxylase and implications toward understanding and treating phenylketonuria, Pediatrics, **112**, 1557–1565 (2003).
14. **Han, S., Eltis, L. D., Timmis, K. N., Muchmore, S. W., and Bolin, J. T.:** Crystal structure of the biphenyl-cleaving extradiol dioxygenase from a PCB-degrading pseudomonad, Science, **270**, 976–980 (1995).
15. **Kauppi, B., Lee, K., Carredano, E., Parales, R. E., Gibson, D. T., Eklund, H., and Ramaswamy, S.:** Structure of an aromatic-ring-hydroxylating dioxygenase-naphthalene 1,2-dioxygenase, Structure, **6**, 571–586 (1998).
16. **Valegard, K., van Scheltinga, A. C., Lloyd, M. D., Hara, T., Ramaswamy, S., Perrakis, A., Thompson, A., Lee, H. J., Baldwin, J. E., Schofield, C. J., Hajdu, J., and Andersson, I.:** Structure of a cephalosporin synthase, Nature, **394**, 805–809 (1998).
17. **Erlandsen, H., Fusetti, F., Martinez, A., Hough, E., Flatmark, T., and Stevens, R. C.:** Crystal structure of the catalytic domain of human phenylalanine hydroxylase reveals the structural basis for phenylketonuria, Nat. Struct. Biol., **4**, 995–1000 (1997).
18. **Jiang, G. C., Yohrling IV, G. J., IV, Schmitt, J. D., and Vrana, K. E.:** Identification of substrate orienting and phosphorylation sites within tryptophan hydroxylase using homology-based molecular modeling, J. Mol. Biol., **302**, 1005–1017 (2000).
