# Supplementary material for: 5-Hydroxytryptophan (5-HTP): Natural Occurrence, Analysis, Biosynthesis, Biotechnology, Physiology and Toxicology
Source: Int J Mol Sci. 2020 Dec 26;22(1):181. doi: 10.3390/ijms22010181 (PMC7796270; doi:10.3390/ijms22010181)
Supplement: Supplementary file 1 [file ijms-22-00181-s001.zip › 5-HTP.Data/PDF/1982330815/279322.pdf]

Review

Pharmacology

Pharmacology 2010;85:95–109
DOI: 10.1159/000279322

Received: November 15, 2009
Accepted: November 17, 2009
Published online: February 3, 2010

Tryptophan Hydroxylase as Novel Target for the Treatment of Depressive Disorders

Susann Matthes Valentina Mosienko Saleh Bashammakh Natalia Alenina
Michael Bader

Max Delbrück Center for Molecular Medicine, Berlin-Buch, Germany

Key Words
Serotonin · Depression · Schizophrenia · Antidepressants

Abstract
Serotonin (5-HT) is a monoamine implicated in a variety of physiological processes that functions either as a neurotransmitter or as a peripheral hormone. Pharmacological and genetic studies in humans and experimental animals have shown that 5-HT is important for the pathophysiology of depressive disorders. The 5-HT system is thus already a main target for the therapy of these diseases. The peripheral and cerebral biosynthesis of 5-HT is initiated by two distinct tryptophan hydroxylases: TPH1 and TPH2. This duality of the serotonergic system and the existence of a brain-specific TPH isoform provide a promising new target for pharmacological intervention with higher selectivity and specificity and, therefore, possibly with reduced side effects and increased efficiency. This paper summarizes the data which support TPH2 as novel drug target and discusses strategies for its pharmacological exploitation.

Copyright © 2010 S. Karger AG, Basel

Introduction

Since its discovery as a vasoconstrictor substance in the blood [1], serotonin (5-hydroxytryptamine, 5-HT) has been revealed to be of importance in a broad range of physiological processes as it has been shown to be involved in the control of smooth muscle tone and vascular function [2–4], hemostasis and platelet function [5, 6], hepatitis and liver regeneration [7–10], mammary gland plasticity [11], insulin secretion [12], development and wiring of neurons [13], as well as regulating sleep/wakefulness, appetite, gastrointestinal motility, temperature, pain sensation and nociception, mood, stress, maternal or sexual behavior and aggression [14–16].

The biosynthesis of the monoamine 5-HT starts with the essential amino acid tryptophan, which is metabolized to 5-hydroxytryptophan (5-HTP) by tryptophan hydroxylase (TPH) in an initial, rate-limiting step; 5-HTP is then further decarboxylated to 5-HT by aromatic amino acid decarboxylase (fig. 1). TPH belongs to the superfamily of aromatic amino acid hydroxylases which also includes tyrosine hydroxylase and phenylalanine hydroxylase (PAH). These are iron (Fe^{2+})- and tetrahydrobiopterin-dependent monooxygenases with substantial similarities in structure and catalytic mechanism [17].

KARGER

Fax +41 61 306 12 34
E-Mail karger@karger.ch
www.karger.com

© 2010 S. Karger AG, Basel
0031–7012/10/0852–0095$26.00/0

Accessible online at:
www.karger.com/pha

Prof. Dr. Michael Bader
Max Delbrück Center for Molecular Medicine
Robert-Rössle-Strasse 10
DE–13125 Berlin-Buch (Germany)
Tel. +49 30 9406 2193, Fax +49 30 9406 2110, E-Mail mbader@mdc-berlin.de

All aromatic amino acid hydroxylases are composed of 3 functional domains, a regulatory N-terminal domain, a catalytic domain and a C-terminal oligomerization domain [17, 18].

The identification of a second *Tph* gene in 2003, named *Tph2*, unravelled the existence of two independent 5-HT systems in vertebrates (fig. 2). Whereas TPH2 is specifically expressed in the brain, TPH1 is responsible for 5-HT synthesis in peripheral tissues [19]. Within the last years, different studies of mRNA and protein levels in rodent and human tissues confirmed TPH2 to be the central isoform, predominantly expressed in the neurons of raphe nuclei in the brain stem [20–22] and in peripheral myenteric neurons in the gut [23, 24] but not in peripheral organs (lung, heart, kidney or liver) [25, 26]. On the other hand, TPH1 is mainly expressed in the enterochromaffin cells of the gut and also in the pineal gland, where it produces 5-HT as a precursor of melatonin synthesis [19, 27] (fig. 1).

5-HT System and Depression

Based on its functions as a neurotransmitter influencing various neurological and behavioral processes in the central nervous system, 5-HT has also been suggested to play a role in different diseases of the central nervous system [15, 28]. The biosynthesis and release of neuronal 5-HT depend on circulating levels of free tryptophan in the blood and brain. Experimental tryptophan depletion was associated with increased symptom severity in depressed patients [29–31] and lowered 5-HT and/or 5-hydroxyindoleacetic acid, its degradation product (fig. 1), have been found in the cerebrospinal fluid of suicidees and depressed suicide attempters [32, 33]. Disturbed social behavior, increased irritability or lack of impulse control could also be observed in inflammatory and other diseases that are accompanied by low tryptophan levels [34]. The fact that the 5-HT system also became an efficient target for antidepressant therapies confirms that alterations in the 5-HT system may be related to the development and pathophysiology of affective disorders [15, 35–38]. Affective disorders are a group of psychiatric conditions among which major (unipolar) depression and bipolar disorder (major depression plus mania) are the most prevalent types [39, 40]. Patients with major depression suffer predominately from mood disturbances, but also from psychomotor retardation, sleep disorders, fatigue or thoughts of death [41]. This paper intends to give an overview on recent genetic studies in humans and animals investigating a possible connection of TPH and psychiatric disorders, with particular emphasis on affective disorders.

Genetic Studies on TPH in Humans

In recent years, there has been a dramatic increase in the number of publications dealing with molecular variants of genes that are involved in 5-HT synthesis (TPH), 5-HT neurotransmission (5-HT receptors and 5-HT transporter, SERT) and 5-HT catabolism (monoamine oxidases) (fig. 1) to identify alterations that may contribute to a dysfunction within the serotonergic system. A huge amount of data has accumulated; in the following we will only focus on genetic studies on *TPH* (table 1).

It has been shown that the prevalence of affective disorders is affected by gender, age and genetic background. Depressive disorders have been found to be more frequent in women, and twin studies suggest a heritability of up to 50% [42, 43]. But at present the results on *TPH* polymorphisms remain inconclusive. Several single nucleotide polymorphisms (SNPs) in the *TPH2* gene have been found to be associated with depression [44–49], suicidal behavior [46, 47, 50–55] and bipolar disorder [48, 50–56]. On the other hand, a number of publications found no association between *TPH2* SNPs and major depression or suicide [57–62]. These discrepant results may reflect the heterogeneous nature of neuropsychiatric diseases and populations as well as methodological differences, sample size and statistical power.

In particular, the nonsynonymous SNP (G1463A) published by Zhang et al. [44] in 2005 seemed to be a promising step towards identifying a link between TPH*2* and depressive disorders. The resulting missense mutation in the TPH2 protein (R441H) that they found in an elderly cohort of unipolar depressed patients showed an 80% decrease in 5-HT production in PC12 cells. The fact that the mutation is located within the part of the oligomerization domain that has previously been shown to be pivotal for TPH*2* activity [63] together with the description of a corresponding severe pathogenic mutation in PAH (R408W) led to the expectation that the first loss-of-function mutation in human *TPH2* had been identified [64]. However, this functional polymorphism could not be confirmed in any other study [62, 65–72]. Thus it is either a rare mutation within a very unique cohort with unipolar depression or the result of a genotyping error.

As the coding sequence of *TPH2* represents less than 2% of the gene, coding sequence variants are expected to

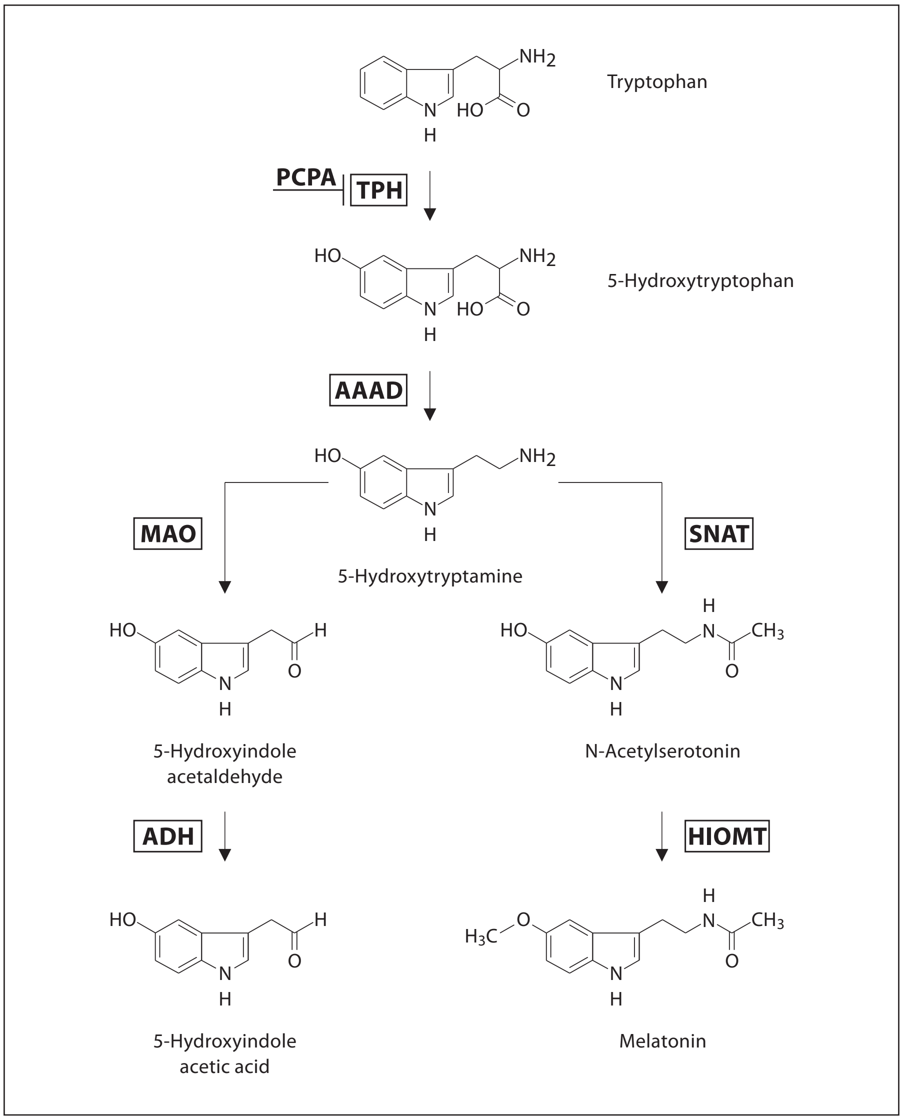

Fig. 1. 5-HT metabolism. AAAD = Aromatic amino acid decarboxylase; SNAT = serotonin N-acetyltransferase; HIOMT = hydroxy-indole-O-methyl transferase; MAO = monoamine oxidase; ADH = alcohol dehydrogenase. The other abbreviations are explained in the text.

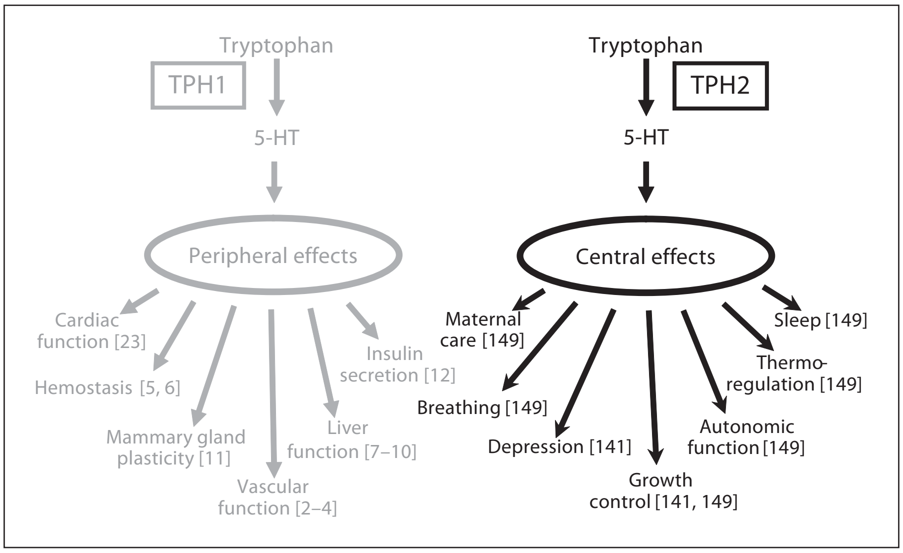

Fig. 2. Duality of the 5-HT system. Functions of peripheral (TPH1-derived) and central (TPH2-derived) 5-HT that are confirmed in *Tph1*- and *Tph2*-deficient animals are shown.

Table 1. TPH2 SNPs and association with affective disorders

SNP	Location	Function	Allele	Protein residue	Disorder	Association	Reference
G1463A	exon 11	missense	G/A	Arg/His	major depression	yes	[44]
rs1386494	intron 5		A/G	–	major depression	yes	[45][1, 2]
rs17110747	3′ UTR		A/G	–	major depression	yes	[49][2]
rs17110563<br>rs11179003<br>rs1386494<br>rs1007023<br>rs1473473<br>rs17110747	exon 6<br>intron 4<br>intron 5<br>intron 8<br>intron 8<br>3′ UTR	missense	C/T<br>C/T<br>A/G<br>G/T<br>A/G<br>A/G	Ser/Pro<br>–<br>–<br>–<br>–<br>–	major depression<br>suicidal behavior	yes	[46][1]
g.22879<br>rs1386493<br>rs1386494	exon 6[3]/intron 5[4]<br>intron 5<br>intron 5		A/G<br>C/T<br>A/G	–<br>–<br>–	major depression<br>suicidal behavior	yes	[47][1, 2]
rs11178997<br>rs4131348	5′ near gene<br>upstream 5′ region	synonymous	A/T<br>C/T	–<br>–	major depression<br>bipolar disorder	yes	[48][1, 2]
rs4290270	exon 9	synonymous	A/T	–	bipolar disorder	yes	[51][1]
rs17110563<br>rs11178997	exon 6<br>5′ near gene	missense<br>synonymous	C/T<br>A/T	Ser/Pro<br>–	bipolar disorder	yes	[50][1, 2]
rs1386482<br>rs1386486	intron 8<br>intron 8		A/C<br>C/T	–<br>–	bipolar disorder	yes	[54][1]
rs7305115<br>rs17840794<br>rs1007023<br>rs11615016<br>rs4290270	exon 7<br>intron 7<br>intron 8<br>intron 8<br>exon 9	synonymous<br><br><br><br>synonymous	A/G<br>C/T<br>G/T<br>A/G<br>A/T	–<br>–<br>–<br>–<br>–	bipolar disorder	yes	[52][1]
rs1386494<br>rs1007023<br>rs9325202	intron 5<br>intron 8<br>intron 8		A/G<br>G/T<br>A/G	–<br>–<br>–	bipolar disorder<br>suicidal behavior	yes	[53][1]
rs7305115	exon 7	synonymous	A/G	–	suicidal behavoir	yes	[191]
rs4448731<br>rs4641527	5′ near gene<br>intron 1		C/T<br>G/T	–<br>–	suicidal behavoir	yes	[192][1]
rs1386494	intron 5		A/G	–	suicidal behavoir	yes	[55][1, 2]
rs11178997	5′ near gene	synonymous	A/T	–	suicidal behavoir	yes	[193]
rs4570625	5′ near gene		G/T	–	suicidal behavoir	yes	[194]
rs1843809	intron 5		G/T	–	ADHD	yes	[84][1, 2]
rs4570625<br>rs11178997	5′ near gene<br>5′ near gene	 synonymous	G/T<br>A/T	–<br>–	ADHD	yes	[85][1]
rs4341581<br>rs11179000	intron 1<br>intron 4		G/T<br>A/T	–<br>–	autism	yes	[86][1]
rs4570625<br>rs4565946	5′ near gene<br>intron 2		G/T<br>C/T	–<br>–	OCD	yes	[87][1, 2]
rs4570625	5′ near gene		G/T	–	panic disorder	yes	[88][2]
G1463A	exon 11	missense	G/A	Arg/His	major depression	no	[62, 65–72]

Table 1 (continued)

SNP	Location	Function	Allele	Protein residue	Disorder	Association	Reference
rs7488262	exon 11	missense	T/G	–	major depression	no	[62]
rs1386494	intron 5		A/G	–	major depression	no	[60]
rs10748185	intron 2		A/G	–	major depression	no	[57][1, 2]
rs2129575	intron 4		G/T	–			
rs1386495	intron 5		C/T	–			
rs1386494	intron 5		A/G	–			
rs7305115	exon 7	synonymous	A/G	–			
rs4131347	upstream 5′ region		C/G	–	major depression<br>bipolar disorder<br>suicidal behavior	no	[61]
rs1487280	intron 9		A/G	–	bipolar disorder	no	[58][1, 2]
rs4760816	intron 6		C/T	–	suicidal behavior		
rs10784941	intron 6		A/G	–			
rs6582071	5′ near gene		A/G	–	suicidal behavior	no	[59][1, 2]
rs4570625	5′ near gene		G/T	–			
rs11178997	5′ near gene	synonymous	A/T	–			
rs11178998	5′ UTR		A/G	–			
rs11178999	intron 1		A/G	–			
rs1386494	intron 5		A/G	–			
rs1386493	intron 5		C/T	–			
rs1386491	intron 5		C/G	–			
rs7305115	exon 7	synonymous	A/G	–			
rs1386498	intron 8		A/G	–			
rs1487278	intron 8		C/T	–			
rs11179044	intron 8		C/T	–			
rs4290270	exon 9	synonymous	A/T	–			
rs11179064	intron 9		A/G	–			
rs17110747	3′ UTR		G/A	–			
rs4570625	5′ near gene		G/T	–	panic disorder	no	[90][1, 2]
rs4565946	intron 2		C/T	–			
rs11178997	5′ near gene	synonymous	A/T	–	suicidal behavior	no	[89][1, 2]
rs10784941	intron 2		A/G	–	schizophrenia		
rs11178997	5′ near gene	synonymous	A/T	–	suicidal behavior	no	[91]
rs4131347	upstream 5′ region		C/G	–			

[1] Haplotype analysis. [2] Single SNP analysis. [3] TPH2 short transcript isoform (ENST00000266669). [4] TPH2 long transcript isoform (ENST00000333850). ADHD = Attention-deficit hyperactivity disorder, OCD = obsessive-compulsive disorder.

be rather rare [73]. Therefore, most of the *TPH2* SNPs known so far are located in introns and promoter regions. Although they are not likely to be of importance for protein function, they could affect gene expression on the transcriptional level, e.g. via mRNA stability or splicing processes [74]. The T allele of the *TPH2* promotor polymorphism rs4570625 (–703G/T) has been shown to be involved in increased prefrontal brain activity [75], anxiety-related personality disorders [76] and amygdala reactivity [77, 78] and therefore might show an impact on heightened stress responsivity and anxiety due to alterations in TPH2 expression [79]. Another intronic *TPH2* SNP has been reported to reduce promoter activity by reduced binding of transcription factor POU3F2 [56, 80]. Evidence for an inhibitory effect of the *TPH2* 5′-UTR on gene expression has been derived from studies on com-

mon polymorphisms and haplotypes in this region [81]. From the *TPH2* missense mutations known so far, 4 have been reported in patients with clinical symptoms [82]. Nevertheless, there is still a great demand for functional and clinical data to define the role of *TPH2* polymorphisms in particular phenotypes of depressive disorders [83].

Furthermore, discrepant results have been published on other types of affective disorders. Some studies showed *TPH2* polymorphisms to be related to schizophrenia, obsessive-compulsive disorder, attention-deficit hyperactivity disorder, and autism or panic disorder [84–88] while others did not observe any association [89–91].

Concordant response rates from several studies on relatives suggest that antidepressant treatment response is also an inheritable trait possibly influenced by *TPH2* polymorphisms [92, 93]. However, whereas some studies show a significant link between a *TPH2* SNP haplotype and specific responses to 5-HT reuptake inhibitors (SSRIs) [49, 94, 95], others, here again, do not confirm any association between this trait and *TPH2* SNPs in various ethnic groups [60, 62, 72].

Although TPH1 is responsible for peripheral 5-HT synthesis, there are several studies on the influence of *TPH1* polymorphisms A218C and A779C in affective disorders, with contrasting outcomes. Both SNPs are proposed to be involved in suicidal behavior [16, 96–100], depression [101–103], bipolar disorder [104–106] or altered antidepressant drug response [107–109]. But other studies could not find any association of *TPH1* SNPs and suicidal behavior [110–114], major depression [113, 115–118], bipolar disorder [113, 119] or antidepressant treatment response [115, 120]. It is not clear whether 5-HT synthesis through TPH1 in the brain just serves for melatonin synthesis or whether there might be some contribution to the neurotransmitter pool [121]. Effects of TPH1 on central 5-HT synthesis can be caused by altered enzyme activity during ontogeny as an impact of TPH1 on the development and maturation of 5-HT neurons has been suggested by studies in mice [122–124], which was contradicted by others, however [125].

Affective disorders are known to be complex and heterogeneous disorders characterized by polygenic influences, different clinical profiles and different responsiveness to drugs [28, 126]. It is also known from studies on multifactorial disorders that different mutations within a single gene may be linked to a spectrum of clinical phenotypes [82]. As the 5-HT system exhibits an extensive network of different genes involved in the development, function and plasticity of 5-HT neurons as well as several 5-HT receptors and 5-HT transporter-associated proteins, contributions from many points in this network in a disease as complex as depression should be expected [68]. In order to improve the success of genetic studies of depression and optimize the phenotypic definition of depression, there is a need to dissect possible psychopathological and biological endophenotypes [127].

Studies in Mouse Models

Validity of Mouse Models of Depression

Studies in genetically modified laboratory animals have an important impact on our understanding of depressive disorders and are necessary to test new pharmacological tools that could be further used in treating these diseases in humans. The mouse – a species in which human mutations can be easily mimicked by genetic modifications of its genome – is especially valuable in this respect. However, the usefulness and validity of mouse models in evaluating human depression has always been a subject of debate. As there are differences in brain anatomy and capacity for processing complex psychological concepts between humans and mice, it is impossible to model certain aspects of disease symptoms, such as low self-esteem, suicidal ideation or 'fear of going crazy' in mice. On the other hand, the brains of mammals have a common structural organization, similar circuits connecting these structures, and conserved physiological and behavioral responses. Therefore, to a certain extent, the mouse can be used as a model for understanding human behavior and disease, but the results of such studies must be interpreted with caution [128].

Current models gauge an animal's 'depression-related' responses to acute or chronic inescapable stress. These include the forced swimming test (FST) which quantifies immobility in a water bath, and the tail suspension test (TST), which measures periods of agitation and immobility of a mouse suspended by the tail; these periods of agitation and immobility resemble 'behavioral despair' observed in depressions. Other assays include measurement of social interaction (model of social estrangement in depression-related conditions), the learned-helplessness test (measures the passive responses to inescapable foot shock), and intracranial self-stimulation (evaluation of the animal's effort to stimulate the cerebral reward circuit electrically) [129–132]. These assays are usually accompanied by a battery of tests evaluating activity and anxiety, such as open field and elevated plus maze to exclude the misinterpretation of results.

Pharmacological Mouse Models of Depression

The first studies to investigate the role of TPH in depressive disorders were pharmacological models in which enzyme activity was specifically blocked with *p*-chlorophenylalanine (PCPA), an irreversible TPH inhibitor (fig. 1) [133]. Mice prenatally exposed to PCPA show increased depression-related behavior in FST and TST, decreased anxiety and increased activity in open field [134–136]. Another pharmacological tool to study the relation between depressive behavior and TPH is local intracerebroventricular application of the neurotoxin 5,7-dihydroxytryptamine (5,7-DHT), which selectively kills serotonergic neurons, and was shown to cause significant depletion of 5-HT levels in the brains of mice [137, 138]. Due to the complicated procedure of intracerebroventricular injection, most of the studies with neurotoxins were done on rats; they showed that 5,7-DHT treatment induces a depressive-like behavior [139, 140]. The few studies in mice demonstrated that 5,7-DHT injections in the striatum significantly decrease locomotor activity [138]. However, these pharmacological models have a number of limitations: (1) PCPA is not specific for a certain form of TPH; therefore it is difficult to distinguish effects caused by TPH1 or TPH2 depletion; (2) 5,7-DHT is a neurotoxin that kills serotonergic neurons; hence, this model does not exclude behavioral abnormalities aroused by the absence of other components of the serotonergic system and cotransmitters of these cells; (3) PCPA and 5,7-DHT induce partial but not complete 5-HT reductions in brain regions, and these approaches do not tell whether the residual amount of 5-HT is enough for normal brain functioning. Therefore, it is more appropriate to use genetically modified mouse models to investigate the role of TPH isoenzymes in depressive disorders.

Genetic Mouse Models of Depression: Role of Tph1

Although several studies showed the discrete expression pattern of *Tph* genes, with neuronal localization of *Tph2* in dorsal raphe and myenteric neurons and non-neuronal localization of *Tph1* in pineal gland and duodenum, the presence and role of *Tph1* transcripts in the raphe region remain a subject of controversy [123, 125]. According to Nakamura et al. [123], there is a peak of *Tph1* expression in the brain stem at weaning in mice whereas in adulthood its level drops to nearly zero. Moreover, in these young mice both the affinity for tryptophan and enzymatic activity of TPH1 are higher than those of TPH2, partially due to the low tetrahydrobiopterin concentration in the developing brain stem and, therefore, TPH1 was considered as a main 5-HT-producing enzyme in the mouse brain at weaning [123]. In the search for the linkage between *Tph1* and murine behavior, two polymorphisms – in the promoter region and in the 3′ UTR of the *Tph1* gene – were recently identified in New Zealand white and SWR mice [123]. Due to these mutations, both strains were shown to have lower *Tph1* expression in the brain stem at 21 days of age in comparison to other laboratory mouse lines and, as a consequence, decreased brain 5-HT levels at this time point, but normal 5-HT levels in adulthood. Interestingly, both strains have shown an increased depression-like behavior in FST and TST tests. However, further studies, on congenic lines carrying the above-mentioned mutations, as well as evaluation of this behavior in *Tph1*-deficient mice, have to be conducted in order to clarify whether impairment in *Tph1* expression can contribute to depressive disorders.

Moreover, other studies did not find considerable TPH1 activity in the mouse raphe nuclei during embryogenesis, postnatal development and in adulthood [19, 125]. Furthermore, *Tph1* remained undetectable even in raphe of *Tph2*-deficient mice, in which compensatory up-regulation may have been expected [125]. In addition, studies in *Tph1*-knockout mice did not reveal any changes in 5-HT-related behavior in anxiety tests [19, 141], rendering the contribution of TPH1-produced 5-HT to 5-HT signaling in the brain unlikely.

A second way by which TPH1-derived 5-HT can influence behavior is via its transformation to melatonin in the pineal gland. Melatonin mediates photoperiodic effects on reproduction and may influence a variety of circadian activities. However, most laboratory mouse strains cannot synthesize melatonin due to a lack of 5-HT-N-acetyltransferase (SNAT) that converts 5-HT to an intermediate precursor of melatonin, N-acetylserotonin [121] (fig. 1). Thus, at least in laboratory mice, *Tph1* cannot influence behavior via melatonin.

Genetic Mouse Models of Depression: Role of Tph2

Tph2 Polymorphism

It is known that certain mouse strains markedly differ in depression-like behavior. Zhang et al. [142] first surmised that such a difference between 129X1/SvJ and BALB/cJ mice may be caused by altered TPH2 activity. Indeed, sequencing analysis revealed an (C1473G) SNP in the coding region of *Tph2* between these two lines resulting in the substitution of a highly conserved proline residue with an arginine at position 447 and, as a consequence, lowering of enzyme activity in in vitro experiments [142, 143]. Afterwards, it was found that the 5-HT content in the brain was reduced by approximately 50%

in the mouse strains carrying only the 1473G allele in comparison to mice homozygous for the 1473C allele; an observation that was also confirmed in an F2 intercross [142, 144, 145]. This led to the hypothesis of a direct link between the *Tph2* polymorphism and the development of a depressive state in those mice. However, attempts to create congenic lines only differing in the mTPH2$_{1473}$ polymorphic allele yielded controversial results and put this hypothesis into question. Osipova et al. [144] showed that transfer of the 1473G allele into the C57BL/6J genome over 3 generations [B6–1473G (G/G)] significantly decreased TPH2 activity in the brain and led to shorter immobility time in the FST. However, we created two congenic lines carrying homozygous 1473G or 1473C alleles obtained from breeding the 1473G allele from DBA/2 mice over 8 generations to the C57BL/6 background and did not find any differences in the 5-HT content of brain regions or changes in depression-related behavior [146], indicating that not *Tph2*, but other variations in the genetic background are responsible for the interstrain differences. Moreover, no association was found between the murine *Tph2* C1473G polymorphism and depressive state in TST [147]. This discrepancy in the results from different investigators can be explained by different experimental protocols or by the degree of genetic background homogeneity of the mice. Furthermore, it cannot be ruled out that *Tph2* is closely linked to some other genes that directly influence FST immobility in mice.

Tph2 Knockin

Beaulieu et al. [148] generated knockin mice expressing a mutant form of TPH2 that contains the rare human mutation R441H identified in few individuals with unipolar depression (see above). TPH2 activity in R439H *Tph2*-knockin mice was reduced by 80% in the brain while the expression of SERT, a target of most antidepressants, was unchanged. Expression of the mutant *Tph2* results in increased anxiety, depression-related behavior in TST and enhanced intermale aggression. The pharmacological or genetic inhibition of GSK3β prevented the behavioral changes, suggesting that drugs that enhance 5-HT transmission may exert some of their actions through GSK3β.

Tph2 Knockout

Several groups recently reported the generation of *Tph2*-knockout mice [22, 141, 149]. We showed increased aggressive behavior in *Tph2*-deficient female mice that cannibalized their pups in contrast to wild-type mice that never ate the whole litter [149]. Female aggression is directly linked to anxiety and depressive disorders [150]. Surprisingly, female *Tph2*-deficient mice behaved normally in the FST in contrast to the TST in which they showed longer immobility times [141]. *Tph2*-deficient males did not show any significant behavioral changes in a standard TST; however, decreased immobility times were recorded in the FST [141]. Different results obtained on the TST and FST are likely due to the different neurochemical and neuroanatomical pathways involved in the modulation of the response to different stress stimuli [132]. Testing *Tph2*-deficient mice for the anxiety-related phenotypes also yielded inconsistent results: open field did not reveal any linkage between TPH2 and anxiety while the buried-marble test showed an increased level of anxiety in these mice [141]. However, it cannot be excluded that increased marble burying is an indication of obsessive/compulsive behavior rather than anxiety-like behavior in these animals [151]. Interestingly, differences observed in depression- and anxiety-like behavior were more pronounced in *Tph2/Tph1* double-knockout mice than in *Tph2*-deficient mice, which may be due to peripheral and developmental discrepancies caused by the additional absence of *Tph1* [141].

Pah-Deficient Mice

PAH is a hepatic enzyme which metabolizes phenylalanine to tyrosine. Deficiency in this enzyme leads to the accumulation of phenylalanine and its conversion into phenylpyruvate and causes a severe disease in humans: phenylketonuria, which is accompanied by postnatal brain damage and mental retardation. *Pah*-deficient mice (*Pah*$^{Enu2-/-}$ mice [152]) exhibit behavioral alterations [153–155]; however, no evaluation of depression-like behavior was made. Interestingly, it was recently shown that PAH ablation in the mouse causes 70% reduction in 5-HT production in the brain without a loss of the 5-HT precursor tryptophan [156]. Nonetheless, a dramatic reduction in brain 5-HTP levels as well as in the 5-HTP/tryptophan ratio was observed in *Pah*$^{Enu2-/-}$ mice, suggesting that accumulation of phenylalanine leads to the inhibition of Tph2 activity in vivo, corresponding to its effect on TPH2 activity in vitro [156, 157]. Therefore, the behavioral phenotype observed in *Pah*-deficient mice can be partially attributed to lower TPH2 activity in the brain. Interestingly, administration of 5-HTP to *Pah*-deficient mice restored the cortical release of several monoamines in a model of restraint stress [157].

For the same reason, a lack of PAH in *Drosophila melanogaster*, Henna, leads to a dramatic decrease in 5-HT. Therefore, for a long time Henna has been considered to

be the PAH and the TPH of insects until the main TPH, CG9122, was discovered [158].

Serotonergic Neuron Ablation

Ablation of TPH2-producing neurons is another possibility to study the association between TPH2 and depressive disorders. Hendricks et al. [159] demonstrated that expression of the transcription factor Pet-1 is restricted to serotonergic neurons and its disruption in mice leads to abnormal development and loss of these cells but does not influence brain morphology and other monoamine systems [159]. *Pet-1* null male mice show dramatically increased anxiety and intermale aggressive behavior supporting the link between aggression and serotonergic hypofunction. Moreover, a profound deficit in offspring survival born from *Pet-1*$^{-/-}$ dams was reported as a result of deficient maternal behavior [160]. However, the level of female anxiety and aggressiveness that could contribute to offspring mortality was not assessed in these studies.

Another mouse model, in which *Lmx1b* (LIM homeobox transcription factor 1β) was only deleted in *Pet-1*-expressing cells, i.e. Lmx1b(f/f/p) mice, lack nearly all serotonergic neurons but do not show any alteration in other monoamine systems [161]. These animals display normal locomotion in Rotarod and open field tests [161], but show reduced basal sensitivity to mechanic stimuli and exhibit enhanced inflammatory pain response [162]. Moreover, this model was used to study the mechanism of analgesic actions of antidepressants, including SSRIs, 5-HT-norepinephrine reuptake inhibitors, and tricyclic antidepressants [162]. The results revealed that the analgesic effect of antidepressant drugs, including those affecting both norepinephrine and 5-HT levels, was abolished in the acute thermal pain model.

Pharmacological Targeting of TPH2

The actual treatment strategies for depressive disorders enhance the general serotonergic tone either by inhibition of the 5-HT transporter by SSRIs, or tricyclic antidepressants or by prevention of 5-HT degradation using monoamine oxidase inhibitors [15, 35]. Also the intermediate product in 5-HT synthesis, 5-HTP (fig. 1), has been successfully used for antidepressive therapy [163–165]. Upon oral administration 5-HTP can reach the brain due to its ability to cross the blood-brain barrier and effectively increase central 5-HT synthesis. The onset of any antidepressant drug effect usually takes up to 4 weeks. Therefore, it is suggested that not just neurochemical but also structural changes, like the stimulation of neurogenesis in the hippocampus [166] or other long-term adaptive changes in 5-HT neurotransmission, may be responsible for behavioral effects of chronic antidepressant treatment [167].

All of the currently applied antidepressant drug therapies show wide variability among patients in treatment response [49]. While 30–40% of patients with major depression do not respond to SSRI treatment [95], just about one third of patients gains full remission after antidepressant therapy [168]. The nonspecific inhibition of the 5-HT transporter can cause peripheral side effects, like a high risk of bleeding [169, 170] or skeletal changes [171]. Further adverse effects, such as weight gain, insomnia and sexual dysfunction, are another problematic issue in humans and have been reported to be the main reason for noncompliance, discontinuation or premature termination of treatment by depressed patients [172]; these adverse effects stress the need for better treatment strategies. Therefore, the duality of the serotonergic system and the existence of a brain-specific TPH isoform constitute a promising new target for pharmacological intervention in the treatment of neuropsychiatric diseases. Instead of acting on already produced 5-HT, the opportunity to specifically influence the initial enzyme of 5-HT synthesis, TPH2 might enable selective manipulation of central 5-HT synthesis with less side effects and a better response rate.

Inhibitors of both TPH enzymes, such as PCPA (fig. 1), have been known for a long time and others have recently been published; however, they act only on *Tph1* due to permeability barriers [173, 174], and the treatment of central diseases needs specific activation of TPH2.

Despite their 70% sequence homology, TPH1 and TPH2 show some remarkable differences which might allow specific modulation of the two isoenzymes. The in vitro expression of recombinant fusion proteins indicated that TPH2 is more soluble than TPH1, and exhibits less substrate inhibition by tryptophan and tetrahydrobiopterin [175, 176]. In addition, these enzymes seem to differ in their substrate specificities: TPH2 showed lower catalytic efficiency towards phenylalanine in vitro [175, 177, 178]. The most important difference is an additional N-terminal regulatory domain in TPH2 consisting of 44 amino acids, which is absent in TPH1 [179]. We have shown that this domain is associated with an inhibitory effect on TPH2 activity [63]. Other studies reported a negative effect of this domain on translational efficiency, stability and solubility of TPH2 compared to tyrosine hydroxylase and PAH [180–183].

Aromatic amino acid hydroxylases share several conserved phosphorylation sites. However, the serin 19 residue in the N-terminal domain of TPH2 is a unique phosphorylation target for protein kinase A and calcium/calmodulin-dependent protein kinase II [184, 185]. This phosphorylation seems to stabilize the protein [184, 186, 187] and is also thought to be relevant for enzyme activation and 14-3-3 protein binding [184, 188]. However, recent studies did not confirm an alteration in the catalytic function of TPH2 upon phosphorylation [185] or binding to the 14-3-3 protein BMH1 [175].

Its unique structural properties, in particular its inhibitory N-terminal domain, may allow specific activation of TPH2 by low molecular-weight substances. Such drugs can be tested either by fluorescence-based activity assays in test tubes [189] or in a cell-based system, for example using the suicide pro-drug 7-hydroxytryptophan, which kills cells depending on their intrinsic TPH activity [190]. Furthermore, identifying the still missing X-ray structure of TPH2 will be another important step in understanding the catalytic mechanism of the enzyme in order to develop novel drugs by virtual design.

Conclusions

In this review, we have shown that variations in the genes for TPH1 and TPH2, the rate-limiting enzymes in 5-HT synthesis, were linked to neuropsychiatric diseases, such as bipolar disorder and major depression in humans, and to depression-like behavior in mice. However, these data are not always reproducible and in particular it is not obvious how alterations in the peripheral enzyme TPH1 may influence functions of the central nervous system. Nevertheless, genetically altered animal models with changes in TPH2 expression support a role of this enzyme in brain function and depression-like behavior. Thus, activation of TPH2 may open new perspectives in the treatment of neurological and psychiatric disorders caused by alterations in brain 5-HT levels. Since TPH2 carries an internal inhibitory domain, activation by small molecules should be feasible. The discrimination between TPH1 and TPH2 is a major task in the search for such compounds since some peripheral actions of 5-HT are deleterious and should not be stimulated. TPH2-activating drugs may exhibit higher efficiency and specificity with less side effects than existing therapies for the treatment of depressive disorders, which are increasingly relevant for public health.

References

1 Rapport MM, Green AA, Page IH: Partial purification of the vasoconstrictor in beef serum. J Biol Chem 1948;174:735–741.
2 Morecroft I, Dempsie Y, Bader M, Walther DJ, Kotnik K, Loughlin L, Nilsen M, MacLean MR: Effect of tryptophan hydroxylase 1 deficiency on the development of hypoxia-induced pulmonary hypertension. Hypertension 2007;49:232–236.
3 Dempsie Y, Morecroft I, Welsh DJ, MacRitchie NA, Herold N, Loughlin L, Nilsen M, Peacock AJ, Harmar A, Bader M, MacLean MR: Converging evidence in support of the serotonin hypothesis of dexfenfluramine-induced pulmonary hypertension with novel transgenic mice. Circulation 2008;117:2928–2937.
4 Nocito A, Dahm F, Jochum W, Jang JH, Georgiev P, Bader M, Graf R, Clavien PA: Serotonin regulates macrophage-mediated angiogenesis in a mouse model of colon cancer allografts. Cancer Res 2008;68:5152–5158.
5 Walther DJ, Peter JU, Winter S, Höltje M, Paulmann N, Grohmann M, Vowinckel J, Alamo-Bethencourt V, Wilhelm CS, Ahnert-Hilger G, Bader M: Serotonylation of small GTPases is a signal transduction pathway that triggers platelet α-granule release. Cell 2003;115:851–862.
6 Duerschmied D, Canault M, Lievens D, Brill A, Cifuni SM, Bader M, Wagner DD: Serotonin stimulates platelet receptor shedding by tumor necrosis factor-α-converting enzyme (ADAM17). J Thromb Haemost 2009; 7:1163–1171.
7 Lesurtel M, Graf R, Aleil B, Walther DJ, Tian Y, Jochum W, Gachet C, Bader M, Clavien PA: Platelet-derived serotonin mediates liver regeneration. Science 2006;312:104–107.
8 Nocito A, Georgiev P, Dahm F, Jochum W, Bader M, Graf R, Clavien PA: Platelets and platelet-derived serotonin promote tissue repair after normothermic hepatic ischemia in mice. Hepatology 2007;45:369–376.
9 Nocito A, Dahm F, Jochum W, Jang JH, Georgiev P, Bader M, Renner EL, Clavien PA: Serotonin mediates oxidative stress and mitochondrial toxicity in a murine model of nonalcoholic steatohepatitis. Gastroenterology 2007;133:608–618.
10 Lang PA, Contaldo C, Georgiev P, El-Badry AM, Recher M, Kurrer M, Cervantes-Barragan L, Ludewig B, Calzascia T, Bolinger B, Merkler D, Odermatt B, Bader M, Graf R, Clavien PA, Hegazy AN, Lohning M, Harris NL, Ohashi PS, Hengartner H, Zinkernagel RM, Lang KS: Aggravation of viral hepatitis by platelet-derived serotonin. Nat Med 2008; 14:756–761.
11 Matsuda M, Imaoka T, Vomachka AJ, Gudelsky GA, Hou Z, Mistry M, Bailey JP, Nieport KM, Walther DJ, Bader M, Horseman ND: Serotonin regulates mammary gland development via an autocrine-paracrine loop. Dev Cell 2004;6:193–203.
12 Paulmann N, Grohmann M, Voigt JP, Bert B, Vowinckel J, Bader M, Skelin M, Jevsek M, Fink H, Rupnik M, Walther DJ: Intracellular serotonin modulates insulin secretion from pancreatic beta-cells by protein serotonylation. PLoS Biol 2009;7:e1000229.
13 Gaspar P, Cases O, Maroteaux L: The developmental role of serotonin: news from mouse molecular genetics. Nat Rev Neurosci 2003; 4:1002–1012.
14 Jacobs BL, Azmitia EC: Structure and function of the brain serotonin system. Physiol Rev 1992;72:165–229.
15 Lucki I: The spectrum of behaviors influenced by serotonin. Biol Psychiatry 1998;44: 151–162.

16 Veenstra-VanderWeele J, Anderson GM, Cook EH Jr: Pharmacogenetics and the serotonin system: initial studies and future directions. Eur J Pharmacol 2000;410:165–181.
17 Fitzpatrick PF: Tetrahydropterin-dependent amino acid hydroxylases. Annu Rev Biochem 1999;68:355–381.
18 Flatmark T, Stevens RC: Structural insight into the aromatic amino acid hydroxylases and their disease-related mutant forms. Chem Rev 1999;99:2137–2160.
19 Walther DJ, Bader M: A unique central tryptophan hydroxylase isoform. Biochem Pharmacol 2003;66:1673–1680.
20 Walther DJ, Peter JU, Bashammakh S, Hortnagl H, Voits M, Fink H, Bader M: Synthesis of serotonin by a second tryptophan hydroxylase isoform. Science 2003;299:76.
21 Patel PD, Pontrello C, Burke S: Robust and tissue-specific expression of TPH2 versus TPH1 in rat raphe and pineal gland. Biol Psychiatry 2004;55:428–433.
22 Gutknecht L, Waider J, Kraft S, Kriegebaum C, Holtmann B, Reif A, Schmitt A, Lesch KP: Deficiency of brain 5-HT synthesis but serotonergic neuron formation in *Tph2* knockout mice. J Neural Transm 2008;115:1127–1132.
23 Cote F, Thevenot E, Fligny C, Fromes Y, Darmon M, Ripoche MA, Bayard E, Hanoun N, Saurini F, Lechat P, Dandolo L, Hamon M, Mallet J, Vodjdani G: Disruption of the nonneuronal *Tph1* gene demonstrates the importance of peripheral serotonin in cardiac function. Proc Natl Acad Sci USA 2003;100:13525–13530.
24 Neal KB, Parry LJ, Bornstein JC: Strain-specific genetics, anatomy and function of enteric neural serotonergic pathways in inbred mice. J Physiol 2009;587:567–586.
25 Zill P, Buttner A, Eisenmenger W, Bondy B, Ackenheil M: Regional mRNA expression of a second tryptophan hydroxylase isoform in postmortem tissue samples of two human brains. Eur Neuropsychopharmacol 2004;14:282–284.
26 Sakowski SA, Geddes TJ, Thomas DM, Levi E, Hatfield JS, Kuhn DM: Differential tissue distribution of tryptophan hydroxylase isoforms 1 and 2 as revealed with monospecific antibodies. Brain Res 2006;1085:11–18.
27 Slominski A, Fischer TW, Zmijewski MA, Wortsman J, Semak I, Zbytek B, Slominski RM, Tobin DJ: On the role of melatonin in skin physiology and pathology. Endocrine 2005;27:137–148.
28 Lesch KP: Gene-environment interaction and the genetics of depression. J Psychiatry Neurosci 2004;29:174–184.
29 Ruhe HG, Mason NS, Schene AH: Mood is indirectly related to serotonin, norepinephrine and dopamine levels in humans: a meta-analysis of monoamine depletion studies. Mol Psychiatry 2007;12:331–359.
30 Smith KA, Fairburn CG, Cowen PJ: Relapse of depression after rapid depletion of tryptophan. Lancet 1997;349:915–919.
31 Neumeister A, Nugent AC, Waldeck T, Geraci M, Schwarz M, Bonne O, Bain EE, Luckenbaugh DA, Herscovitch P, Charney DS, Drevets WC: Neural and behavioral responses to tryptophan depletion in unmedicated patients with remitted major depressive disorder and controls. Arch Gen Psychiatry 2004;61:765–773.
32 Mann JJ, Malone KM: Cerebrospinal fluid amines and higher-lethality suicide attempts in depressed inpatients. Biol Psychiatry 1997;41:162–171.
33 Asberg M: Neurotransmitters and suicidal behavior. The evidence from cerebrospinal fluid studies. Ann NY Acad Sci 1997;836:158–181.
34 Russo S, Kema IP, Bosker F, Haavik J, Korf J: Tryptophan as an evolutionarily conserved signal to brain serotonin: molecular evidence and psychiatric implications. World J Biol Psychiatry 2007;1–11.
35 Gainetdinov RR, Caron MG: Monoamine transporters: from genes to behavior. Annu Rev Pharmacol Toxicol 2003;43:261–284.
36 Owens MJ, Nemeroff CB: Role of serotonin in the pathophysiology of depression: focus on the serotonin transporter. Clin Chem 1994;40:288–295.
37 Ressler KJ, Nemeroff CB: Role of serotonergic and noradrenergic systems in the pathophysiology of depression and anxiety disorders. Depress Anxiety 2000;12(suppl 1):2–19.
38 Hornung JP: The human raphe nuclei and the serotonergic system. J Chem Neuroanat 2003;26:331–343.
39 Fava M, Kendler KS: Major depressive disorder. Neuron 2000;28:335–341.
40 Kessler RC, Chiu WT, Demler O, Merikangas KR, Walters EE: Prevalence, severity, and comorbidity of 12-month DSM-IV disorders in the National Comorbidity Survey Replication. Arch Gen Psychiatry 2005;62:617–627.
41 Wurtman RJ: Genes, stress, and depression. Metabolism 2005;54:16–19.
42 Utge S, Soronen P, Partonen T, Loukola A, Kronholm E, Pirkola S, Nyman E, Porkka-Heiskanen T, Paunio T: A population-based association study of candidate genes for depression and sleep disturbance. Am J Med Genet B Neuropsychiatr Genet 2009 Jun 22. Epub ahead of print.
43 Toney SD: Identifying and managing depression in women. J Manag Care Pharm 2007;13:S16–S22.
44 Zhang X, Gainetdinov RR, Beaulieu JM, Sotnikova TD, Burch LH, Williams RB, Schwartz DA, Krishnan KR, Caron MG: Loss-of-function mutation in tryptophan hydroxylase-2 identified in unipolar major depression. Neuron 2005;45:11–16.
45 Zill P, Baghai TC, Zwanzger P, Schule C, Eser D, Rupprecht R, Moller HJ, Bondy B, Ackenheil M: SNP and haplotype analysis of a novel tryptophan hydroxylase isoform *(TPH2)* gene provide evidence for association with major depression. Mol Psychiatry 2004;9:1030–1036.
46 Zhou Z, Roy A, Lipsky R, Kuchipudi K, Zhu G, Taubman J, Enoch MA, Virkkunen M, Goldman D: Haplotype-based linkage of tryptophan hydroxylase 2 to suicide attempt, major depression, and cerebrospinal fluid 5-hydroxyindoleacetic acid in 4 populations. Arch Gen Psychiatry 2005;62:1109–1118.
47 Haghighi F, Bach-Mizrachi H, Huang YY, Arango V, Shi S, Dwork AJ, Rosoklija G, Sheng HT, Morozova I, Ju J, Russo JJ, Mann JJ: Genetic architecture of the human tryptophan hydroxylase 2 Gene: existence of neural isoforms and relevance for major depression. Mol Psychiatry 2008;13:813–820.
48 Van Den Bogaert A, Sleegers K, De Zutter S, Heyrman L, Norrback KF, Adolfsson R, Van Broeckhoven C, Del-Favero J: Association of brain-specific tryptophan hydroxylase, TPH2, with unipolar and bipolar disorder in a Northern Swedish, isolated population. Arch Gen Psychiatry 2006;63:1103–1110.
49 Tsai SJ, Hong CJ, Liou YJ, Yu YW, Chen TJ, Hou SJ, Yen FC: Tryptophan hydroxylase 2 gene is associated with major depression and antidepressant treatment response. Prog Neuropsychopharmacol Biol Psychiatry 2009;33:637–641.
50 Cichon S, Winge I, Mattheisen M, Georgi A, Karpushova A, Freudenberg J, Freudenberg-Hua Y, Babadjanova G, Van Den Bogaert A, Abramova LI, Kapiletti S, Knappskog PM, McKinney J, Maier W, Jamra RA, Schulze TG, Schumacher J, Propping P, Rietschel M, Haavik J, Nothen MM: Brain-specific tryptophan hydroxylase 2 (TPH2): a functional Pro206Ser substitution and variation in the 5′-region are associated with bipolar affective disorder. Hum Mol Genet 2008;17:87–97.
51 Harvey M, Gagne B, Labbe M, Barden N: Polymorphisms in the neuronal isoform of tryptophan hydroxylase 2 are associated with bipolar disorder in French Canadian pedigrees. Psychiatr Genet 2007;17:17–22.
52 Harvey M, Shink E, Tremblay M, Gagne B, Raymond C, Labbe M, Walther DJ, Bader M, Barden N: Support for the involvement of *TPH2* gene in affective disorders. Mol Psychiatry 2004;9:980–981.
53 Lopez VA, Detera-Wadleigh S, Cardona I, Kassem L, McMahon FJ: Nested association between genetic variation in tryptophan hydroxylase II, bipolar affective disorder, and suicide attempts. Biol Psychiatry 2007;61:181–186.
54 Roche S, McKeon P: Support for tryptophan hydroxylase-2 as a susceptibility gene for bipolar affective disorder. Psychiatr Genet 2009;19:142–146.
55 Zill P, Buttner A, Eisenmenger W, Moller HJ, Bondy B, Ackenheil M: Single nucleotide polymorphism and haplotype analysis of a novel tryptophan hydroxylase isoform *(TPH2)* gene in suicide victims. Biol Psychiatry 2004;56:581–586.

56 Lin YM, Chao SC, Chen TM, Lai TJ, Chen JS, Sun HS: Association of functional polymorphisms of the human tryptophan hydroxylase 2 gene with risk for bipolar disorder in Han Chinese. Arch Gen Psychiatry 2007;64: 1015–1024.
57 Gizatullin R, Zaboli G, Jonsson EG, Asberg M, Leopardi R: The tryptophan hydroxylase *(TPH)* 2 gene unlike *TPH1* exhibits no association with stress-induced depression. J Affect Disord 2008;107:175–179.
58 De Luca V, Mueller DJ, Tharmalingam S, King N, Kennedy JL: Analysis of the novel *Tph2* gene in bipolar disorder and suicidality. Mol Psychiatry 2004;9:896–897.
59 Mouri K, Hishimoto A, Fukutake M, Shiroiwa K, Asano M, Nagasaki Y, Ueno Y, Shirakawa O, Nishiguchi N, Maeda K: *TPH2* is not a susceptibility gene for suicide in Japanese population. Prog Neuropsychopharmacol Biol Psychiatry 2009;33:1546–1550.
60 Illi A, Setala-Soikkeli E, Viikki M, Poutanen O, Huhtala H, Mononen N, Lehtimaki T, Leinonen E, Kampman O: *5-HTR1A, 5-HTR2A, 5-HTR6, TPH1* and *TPH2* polymorphisms and major depression. Neuroreport 2009;20: 1125–1128.
61 Mann JJ, Currier D, Murphy L, Huang YY, Galfalvy H, Brent D, Greenhill L, Oquendo M: No association between a *TPH2* promoter polymorphism and mood disorders or monoamine turnover. J Affect Disord 2008; 106:117–121.
62 Garriock HA, Allen JJ, Delgado P, Nahaz Z, Kling MA, Carpenter L, Burke M, Burke W, Schwartz T, Marangell LB, Husain M, Erickson RP, Moreno FA: Lack of association of *TPH2* exon XI polymorphisms with major depression and treatment resistance. Mol Psychiatry 2005;10:976–977.
63 Tenner K, Walther D, Bader M: Influence of human tryptophan hydroxylase 2 N- and C-terminus on enzymatic activity and oligomerization. J Neurochem 2007;102:1887–1894.
64 Zhang X, Beaulieu JM, Gainetdinov RR, Caron MG: Functional polymorphisms of the brain serotonin synthesizing enzyme tryptophan hydroxylase-2. Cell Mol Life Sci 2006;63:6–11.
65 Van Den Bogaert A, De Zutter S, Heyrman L, Mendlewicz J, Adolfsson R, Van Broeckhoven C, Del-Favero J: Response to Zhang et al (2005): Loss-of-function mutation in tryptophan hydroxylase-2 identified in unipolar major depression. Neuron 45, 11–16. Neuron 2005;48:704; author reply 705–706.
66 Zhou Z, Peters EJ, Hamilton SP, McMahon F, Thomas C, McGrath PJ, Rush J, Trivedi MH, Charney DS, Roy A, Wisniewski S, Lipsky R, Goldman D: Response to Zhang et al. (2005): Loss-of-function mutation in tryptophan hydroxylase-2 identified in unipolar major depression. Neuron 45, 11–16. Neuron 2005;48:702–703; author reply 705–706.
67 Bicalho MA, Pimenta GJ, Neves FS, Correa H, de Moraes EN, De Marco L, Romano-Silva MA: Genotyping of the G1463A (Arg441His) *TPH2* polymorphism in a geriatric population of patients with major depression. Mol Psychiatry 2006;11:799–800.
68 Blakely RD: Overview: a rare opportunity or just one less reason to be depressed. Neuron 2005;48:701–702; author reply 705–706.
69 Delorme R, Durand CM, Betancur C, Wagner M, Ruhrmann S, Grabe HJ, Nygren G, Gillberg C, Leboyer M, Bourgeron T, Courtet P, Jollant F, Buresi C, Aubry JM, Baud P, Bondolfi G, Bertschy G, Perroud N, Malafosse A: No human tryptophan hydroxylase-2 gene R441H mutation in a large cohort of psychiatric patients and control subjects. Biol Psychiatry 2006;60:202–203.
70 Glatt CE, Carlson E, Taylor TR, Risch N, Reus VI, Schaefer CA: Response to Zhang et al. (2005): Loss-of-function mutation in tryptophan hydroxylase-2 identified in unipolar major depression. Neuron 45, 11–16. Neuron 2005;48:704–705; author reply 705–706.
71 Henningsson S, Annerbrink K, Olsson M, Allgulander C, Andersch S, Sjodin I, Gustafson D, Skoog I, Eriksson E, Westberg L: Absence of the Arg441His polymorphism in the tryptophan hydroxylase 2 gene in adults with anxiety disorders and depression. Am J Med Genet B Neuropsychiatr Genet 2007; 144B:816–817.
72 Peters EJ, Slager SL, Jenkins GD, Reinalda MS, Garriock HA, Shyn SI, Kraft JB, McGrath PJ, Hamilton SP: Resequencing of serotonin-related genes and association of tagging SNPs to citalopram response. Pharmacogenet Genomics 2009;19:1–10.
73 Sachidanandam R, Weissman D, Schmidt SC, Kakol JM, Stein LD, Marth G, Sherry S, Mullikin JC, Mortimore BJ, Willey DL, Hunt SE, Cole CG, Coggill PC, Rice CM, Ning Z, Rogers J, Bentley DR, Kwok PY, Mardis ER, Yeh RT, Schultz B, Cook L, Davenport R, Dante M, Fulton L, Hillier L, Waterston RH, McPherson JD, Gilman B, Schaffner S, Van Etten WJ, Reich D, Higgins J, Daly MJ, Blumenstiel B, Baldwin J, Stange-Thomann N, Zody MC, Linton L, Lander ES, Altshuler D: A map of human genome sequence variation containing 1.42 million single nucleotide polymorphisms. Nature 2001;409:928–933.
74 Breidenthal SE, White DJ, Glatt CE: Identification of genetic variants in the neuronal form of tryptophan hydroxylase *(TPH2).* Psychiatr Genet 2004;14:69–72.
75 Reuter M, Esslinger C, Montag C, Lis S, Gallhofer B, Kirsch P: A functional variant of the tryptophan hydroxylase 2 gene impacts working memory: a genetic imaging study. Biol Psychol 2008;79:111–117.
76 Gutknecht L, Jacob C, Strobel A, Kriegebaum C, Muller J, Zeng Y, Markert C, Escher A, Wendland J, Reif A, Mossner R, Gross C, Brocke B, Lesch KP: Tryptophan hydroxylase-2 gene variation influences personality traits and disorders related to emotional dysregulation. Int J Neuropsychopharmacol 2007;10:309–320.
77 Brown SM, Peet E, Manuck SB, Williamson DE, Dahl RE, Ferrell RE, Hariri AR: A regulatory variant of the human tryptophan hydroxylase-2 gene biases amygdala reactivity. Mol Psychiatry 2005;10:884–888, 805.
78 Canli T, Congdon E, Gutknecht L, Constable RT, Lesch KP: Amygdala responsiveness is modulated by tryptophan hydroxylase-2 gene variation. J Neural Transm 2005;112: 1479–1485.
79 Stein DJ, Westenberg HG, Liebowitz MR: Social anxiety disorder and generalized anxiety disorder: serotonergic and dopaminergic neurocircuitry. J Clin Psychiatry 2002; 63(suppl 6):12–19.
80 Scheuch K, Lautenschlager M, Grohmann M, Stahlberg S, Kirchheiner J, Zill P, Heinz A, Walther DJ, Priller J: Characterization of a functional promoter polymorphism of the human tryptophan hydroxylase 2 gene in serotonergic raphe neurons. Biol Psychiatry 2007;62:1288–1294.
81 Chen GL, Vallender EJ, Miller GM: Functional characterization of the human *TPH2* 5′ regulatory region: untranslated region and polymorphisms modulate gene expression in vitro. Hum Genet 2008;122:645–657.
82 McKinney JA, Turel B, Winge I, Knappskog PM, Haavik J: Functional properties of missense variants of human tryptophan hydroxylase 2. Hum Mutat 2009;30:787–794.
83 Haavik J, Blau N, Thony B: Mutations in human monoamine-related neurotransmitter pathway genes. Hum Mutat 2008;29:891–902.
84 Sheehan K, Lowe N, Kirley A, Mullins C, Fitzgerald M, Gill M, Hawi Z: Tryptophan hydroxylase 2 *(TPH2)* gene variants associated with ADHD. Mol Psychiatry 2005;10: 944–949.
85 Walitza S, Renner TJ, Dempfle A, Konrad K, Wewetzer C, Halbach A, Herpertz-Dahlmann B, Remschmidt H, Smidt J, Linder M, Flierl L, Knolker U, Friedel S, Schafer H, Gross C, Hebebrand J, Warnke A, Lesch KP: Transmission disequilibrium of polymorphic variants in the tryptophan hydroxylase-2 gene in attention-deficit/hyperactivity disorder. Mol Psychiatry 2005;10:1126–1132.
86 Coon H, Dunn D, Lainhart J, Miller J, Hamil C, Battaglia A, Tancredi R, Leppert MF, Weiss R, McMahon W: Possible association between autism and variants in the brain-expressed tryptophan hydroxylase gene *(TPH2).* Am J Med Genet B Neuropsychiatr Genet 2005;135B:42–46.

87 Mossner R, Walitza S, Geller F, Scherag A, Gutknecht L, Jacob C, Bogusch L, Remschmidt H, Simons M, Herpertz-Dahlmann B, Fleischhaker C, Schulz E, Warnke A, Hinney A, Wewetzer C, Lesch KP: Transmission disequilibrium of polymorphic variants in the tryptophan hydroxylase-2 gene in children and adolescents with obsessive-compulsive disorder. Int J Neuropsychopharmacol 2006;9:437–442.
88 Kim YK, Lee HJ, Yang JC, Hwang JA, Yoon HK: A tryptophan hydroxylase 2 gene polymorphism is associated with panic disorder. Behav Genet 2009;39:170–175.
89 De Luca V, Voineskos D, Wong GW, Shinkai T, Rothe C, Strauss J, Kennedy JL: Promoter polymorphism of second tryptophan hydroxylase isoform *(TPH2)* in schizophrenia and suicidality. Psychiatry Res 2005;134: 195–198.
90 Mossner R, Freitag CM, Gutknecht L, Reif A, Tauber R, Franke P, Fritze J, Wagner G, Peikert G, Wenda B, Sand P, Rietschel M, Garritsen H, Jacob C, Lesch KP, Deckert J: The novel brain-specific tryptophan hydroxylase-2 gene in panic disorder. J Psychopharmacol 2006;20:547–552.
91 De Luca V, Hlousek D, Likhodi O, Van Tol HH, Kennedy JL, Wong AH: The interaction between *TPH2* promoter haplotypes and clinical-demographic risk factors in suicide victims with major psychoses. Genes Brain Behav 2006;5:107–110.
92 O'Reilly RL, Bogue L, Singh SM: Pharmacogenetic response to antidepressants in a multicase family with affective disorder. Biol Psychiatry 1994;36:467–471.
93 Franchini L, Serretti A, Gasperini M, Smeraldi E: Familial concordance of fluvoxamine response as a tool for differentiating mood disorder pedigrees. J Psychiatr Res 1998;32:255–259.
94 Peters EJ, Slager SL, McGrath PJ, Knowles JA, Hamilton SP: Investigation of serotonin-related genes in antidepressant response. Mol Psychiatry 2004;9:879–889.
95 Tzvetkov MV, Brockmoller J, Roots I, Kirchheiner J: Common genetic variations in human brain-specific tryptophan hydroxylase-2 and response to antidepressant treatment. Pharmacogenet Genomics 2008; 18:495–506.
96 Liu X, Li H, Qin W, He G, Li D, Shen Y, Shen J, Gu N, Feng G, He L: Association of *TPH1* with suicidal behaviour and psychiatric disorders in the Chinese population. J Med Genet 2006;43:e4.
97 Mann JJ, Malone KM, Nielsen DA, Goldman D, Erdos J, Gelernter J: Possible association of a polymorphism of the tryptophan hydroxylase gene with suicidal behavior in depressed patients. Am J Psychiatry 1997;154:1451–1453.
98 Rujescu D, Giegling I, Sato T, Hartmann AM, Moller HJ: Genetic variations in tryptophan hydroxylase in suicidal behavior: analysis and meta-analysis. Biol Psychiatry 2003;54:465–473.
99 Bellivier F, Chaste P, Malafosse A: Association between the *TPH* gene A218C polymorphism and suicidal behavior: a meta-analysis. Am J Med Genet B Neuropsychiatr Genet 2004;124B:87–91.
100 Nielsen DA, Virkkunen M, Lappalainen J, Eggert M, Brown GL, Long JC, Goldman D, Linnoila M: A tryptophan hydroxylase gene marker for suicidality and alcoholism. Arch Gen Psychiatry 1998;55:593–602.
101 Jokela M, Raikkonen K, Lehtimaki T, Rontu R, Keltikangas-Jarvinen L: Tryptophan hydroxylase 1 gene *(TPH1)* moderates the influence of social support on depressive symptoms in adults. J Affect Disord 2007; 100:191–197.
102 Sun HS, Tsai HW, Ko HC, Chang FM, Yeh TL: Association of tryptophan hydroxylase gene polymorphism with depression, anxiety and comorbid depression and anxiety in a population-based sample of postpartum Taiwanese women. Genes Brain Behav 2004;3:328–336.
103 Gizatullin R, Zaboli G, Jonsson EG, Asberg M, Leopardi R: Haplotype analysis reveals tryptophan hydroxylase *(TPH)* 1 gene variants associated with major depression. Biol Psychiatry 2006;59:295–300.
104 Bellivier F, Leboyer M, Courtet P, Buresi C, Beaufils B, Samolyk D, Allilaire JF, Feingold J, Mallet J, Malafosse A: Association between the tryptophan hydroxylase gene and manic-depressive illness. Arch Gen Psychiatry 1998;55:33–37.
105 Chen C, Glatt SJ, Tsuang MT: The tryptophan hydroxylase gene influences risk for bipolar disorder but not major depressive disorder: results of meta-analyses. Bipolar Disord 2008;10:816–821.
106 Zaboli G, Gizatullin R, Nilsonne A, Wilczek A, Jonsson EG, Ahnemark E, Asberg M, Leopardi R: Tryptophan hydroxylase-1 gene variants associate with a group of suicidal borderline women. Neuropsychopharmacology 2006;31:1982–1990.
107 Serretti A, Zanardi R, Rossini D, Cusin C, Lilli R, Smeraldi E: Influence of tryptophan hydroxylase and serotonin transporter genes on fluvoxamine antidepressant activity. Mol Psychiatry 2001;6:586–592.
108 Serretti A, Zanardi R, Cusin C, Rossini D, Lorenzi C, Smeraldi E: Tryptophan hydroxylase gene associated with paroxetine antidepressant activity. Eur Neuropsychopharmacol 2001;11:375–380.
109 Ham BJ, Lee BC, Paik JW, Kang RH, Choi MJ, Choi IG, Lee MS: Association between the tryptophan hydroxylase-1 gene A218C polymorphism and citalopram antidepressant response in a Korean population. Prog Neuropsychopharmacol Biol Psychiatry 2007;31:104–107.
110 Lalovic A, Turecki G: Meta-analysis of the association between tryptophan hydroxylase and suicidal behavior. Am J Med Genet 2002;114:533–540.
111 Yoon HK, Kim YK: Association between serotonin-related gene polymorphisms and suicidal behavior in depressive patients. Prog Neuropsychopharmacol Biol Psychiatry 2008;32:1293–1297.
112 Viana MM, De Marco LA, Boson WL, Romano-Silva MA, Correa H: Investigation of A218C tryptophan hydroxylase polymorphism: association with familial suicide behavior and proband's suicide attempt characteristics. Genes Brain Behav 2006;5: 340–345.
113 Furlong RA, Ho L, Rubinsztein JS, Walsh C, Paykel ES, Rubinsztein DC: No association of the tryptophan hydroxylase gene with bipolar affective disorder, unipolar affective disorder, or suicidal behaviour in major affective disorder. Am J Med Genet 1998;81: 245–247.
114 Stefulj J, Kubat M, Balija M, Skavic J, Jernej B: Variability of the tryptophan hydroxylase gene: study in victims of violent suicide. Psychiatry Res 2005;134:67–73.
115 Ham BJ, Lee MS, Lee HJ, Kang RH, Han CS, Choi MJ, Lee SH, Ryu SH: No association between the tryptophan hydroxylase gene polymorphism and major depressive disorders and antidepressant response in a Korean population. Psychiatr Genet 2005;15: 299–301.
116 Yoshida K, Naito S, Takahashi H, Sato K, Ito K, Kamata M, Higuchi H, Shimizu T, Itoh K, Inoue K, Tezuka T, Suzuki T, Ohkubo T, Sugawara K, Otani K: Monoamine oxidase: A gene polymorphism, tryptophan hydroxylase gene polymorphism and antidepressant response to fluvoxamine in Japanese patients with major depressive disorder. Prog Neuropsychopharmacol Biol Psychiatry 2002;26:1279–1283.
117 Du L, Faludi G, Palkovits M, Bakish D, Hrdina PD: Tryptophan hydroxylase gene 218A/C polymorphism is not associated with depressed suicide. Int J Neuropsychopharmacol 2000;3:215–220.
118 Souery D, Van Gestel S, Massat I, Blairy S, Adolfsson R, Blackwood D, Del-Favero J, Dikeos D, Jakovljevic M, Kaneva R, Lattuada E, Lerer B, Lilli R, Milanova V, Muir W, Nothen M, Oruc L, Papadimitriou G, Propping P, Schulze T, Serretti A, Shapira B, Smeraldi E, Stefanis C, Thomson M, Van Broeckhoven C, Mendlewicz J: Tryptophan hydroxylase polymorphism and suicidality in unipolar and bipolar affective disorders: a multicenter association study. Biol Psychiatry 2001;49:405–409.
119 Tsai SJ, Hong CJ, Wang YC: Tryptophan hydroxylase gene polymorphism (A218C) and suicidal behaviors. Neuroreport 1999; 10:3773–3775.

120 Kato M, Wakeno M, Okugawa G, Fukuda T, Azuma J, Kinoshita T, Serretti A: No association of *TPH1* 218A/C polymorphism with treatment response and intolerance to SSRIs in Japanese patients with major depression. Neuropsychobiology 2007;56: 167–171.
121 Coon SL, Mazuruk K, Bernard M, Roseboom PH, Klein DC, Rodriguez IR: The human serotonin N-acetyltransferase (EC 2.3.1.87) gene *(AANAT)*: structure, chromosomal localization, and tissue expression. Genomics 1996;34:76–84.
122 Holmes A: Genetic variation in cortico-amygdala serotonin function and risk for stress-related disease. Neurosci Biobehav Rev 2008;32:1293–1314.
123 Nakamura K, Sugawara Y, Sawabe K, Ohashi A, Tsurui H, Xiu Y, Ohtsuji M, Lin QS, Nishimura H, Hasegawa H, Hirose S: Late developmental stage-specific role of tryptophan hydroxylase 1 in brain serotonin levels. J Neurosci 2006;26:530–534.
124 Nakamura K, Hasegawa H: Developmental role of tryptophan hydroxylase in the nervous system. Mol Neurobiol 2007;35:45–54.
125 Gutknecht L, Kriegebaum C, Waider J, Schmitt A, Lesch KP: Spatio-temporal expression of tryptophan hydroxylase isoforms in murine and human brain: convergent data from *Tph2* knockout mice. Eur Neuropsychopharmacol 2009;19:266–282.
126 Malhotra AK, Murphy GM Jr, Kennedy JL: Pharmacogenetics of psychotropic drug response. Am J Psychiatry 2004;161:780–796.
127 Hasler G, Drevets WC, Manji HK, Charney DS: Discovering endophenotypes for major depression. Neuropsychopharmacology 2004;29:1765–1781.
128 Cryan JF, Holmes A: The ascent of mouse: advances in modelling human depression and anxiety. Nat Rev Drug Discov 2005;4: 775–790.
129 Cryan JF, Mombereau C, Vassout A: The tail suspension test as a model for assessing antidepressant activity: review of pharmacological and genetic studies in mice. Neurosci Biobehav Rev 2005;29:571–625.
130 Invernizzi RW: Role of *TPH-2* in brain function: news from behavioral and pharmacologic studies. J Neurosci Res 2007;85: 3030–3035.
131 Krishnan V, Nestler EJ: The molecular neurobiology of depression. Nature 2008;455: 894–902.
132 Renard CE, Dailly E, David DJ, Hascoet M, Bourin M: Monoamine metabolism changes following the mouse forced swimming test but not the tail suspension test. Fundam Clin Pharmacol 2003;17:449–455.
133 Koe BK, Weissman A: *p*-Chlorophenylalanine: a specific depletor of brain serotonin. J Pharmacol Exp Ther 1966;154:499–516.
134 Vataeva LA, Khozhai LI, Makukhina GV, Otellin VA: Behavior of female and male mice submitted to action of *p*-chlorophenylalanine in prenatal ontogenesis (in Russian). Zh Evol Biokhim Fiziol 2007;43:346–351.
135 Vataeva LA, Kudrin VS, Vershinina EA, Mosin VM, Tiul'kova EI, Otellin VA: Behavioral alteration in the adult rats prenatally exposed to para-chlorophenylalanine. Brain Res 2007;1169:9–16.
136 Vataeva LA, Kudrin VS, Vershinina EA, Mosin VM, Tiul'kova EI, Otellin VA: Maternal para-chlorophenylalanine exposure modifies central monoamines and behaviors in the adult offspring. Brain Res 2008; 1234:1–7.
137 Baumgarten HG, Lachenmayer L: Serotonin neurotoxins – past and present. Neurotox Res 2004;6:589–614.
138 Chia LG, Ni DR, Cheng FC, Ho YP, Kuo JS: Intrastriatal injection of 5,7-dihydroxytryptamine decreased 5-HT levels in the striatum and suppressed locomotor activity in C57BL/6 mice. Neurochem Res 1999; 24:719–722.
139 Hall FS, Devries AC, Fong GW, Huang S, Pert A: Effects of 5,7-dihydroxytryptamine depletion of tissue serotonin levels on extracellular serotonin in the striatum assessed with in vivo microdialysis: relationship to behavior. Synapse 1999;33:16–25.
140 Kostowski W, Krzascik P: Neonatal 5-hydroxytryptamine depletion induces depressive-like behavior in adult rats. Pol J Pharmacol 2003;55:957–963.
141 Savelieva KV, Zhao S, Pogorelov VM, Rajan I, Yang Q, Cullinan E, Lanthorn TH: Genetic disruption of both tryptophan hydroxylase genes dramatically reduces serotonin and affects behavior in models sensitive to antidepressants. PLoS One 2008;3:e3301.
142 Zhang X, Beaulieu JM, Sotnikova TD, Gainetdinov RR, Caron MG: Tryptophan hydroxylase-2 controls brain serotonin synthesis. Science 2004;305:217.
143 Sakowski SA, Geddes TJ, Kuhn DM: Mouse tryptophan hydroxylase isoform 2 and the role of proline 447 in enzyme function. J Neurochem 2006;96:758–765.
144 Osipova DV, Kulikov AV, Popova NK: C1473G polymorphism in mouse *tph2* gene is linked to tryptophan hydroxylase-2 activity in the brain, intermale aggression, and depressive-like behavior in the forced swim test. J Neurosci Res 2009;87:1168–1174.
145 Kulikov AV, Osipova DV, Popova NK: The C1473G polymorphism in gene *tph2* is the main factor mediating the genetically defined variability of tryptophan hydroxylase-2 activity in the mouse brain (in Russian). Genetika 2007;43:1676–1681.
146 Tenner K, Qadri F, Bert B, Voigt JP, Bader M: The m*TPH2* C1473G single nucleotide polymorphism is not responsible for behavioural differences between mouse strains. Neurosci Lett 2008;431:21–25.
147 Crowley JJ, Blendy JA, Lucki I: Strain-dependent antidepressant-like effects of citalopram in the mouse tail suspension test. Psychopharmacology (Berl) 2005;183:257–264.
148 Beaulieu JM, Zhang X, Rodriguiz RM, Sotnikova TD, Cools MJ, Wetsel WC, Gainetdinov RR, Caron MG: Role of GSK3 beta in behavioral abnormalities induced by serotonin deficiency. Proc Natl Acad Sci USA 2008;105:1333–1338.
149 Alenina N, Kikic D, Todiras M, Mosienko V, Qadri F, Plehm R, Boye P, Vilianovitch L, Sohr R, Tenner K, Hortnagl H, Bader M: Growth retardation and altered autonomic control in mice lacking brain serotonin. Proc Natl Acad Sci USA 2009;106:10332–10337.
150 Bosch OJ, Meddle SL, Beiderbeck DI, Douglas AJ, Neumann ID: Brain oxytocin correlates with maternal aggression: link to anxiety. J Neurosci 2005;25:6807–6815.
151 Londei T, Valentini AM, Leone VG: Investigative burying by laboratory mice may involve non-functional, compulsive, behaviour. Behav Brain Res 1998;94:249–254.
152 Shedlovsky A, McDonald JD, Symula D, Dove WF: Mouse models of human phenylketonuria. Genetics 1993;134:1205–1210.
153 Mochizuki S, Mizukami H, Ogura T, Kure S, Ichinohe A, Kojima K, Matsubara Y, Kobayahi E, Okada T, Hoshika A, Ozawa K, Kume A: Long-term correction of hyperphenylalaninemia by AAV-mediated gene transfer leads to behavioral recovery in phenylketonuria mice. Gene Ther 2004;11: 1081–1086.
154 Zagreda L, Goodman J, Druin DP, McDonald D, Diamond A: Cognitive deficits in a genetic mouse model of the most common biochemical cause of human mental retardation. J Neurosci 1999;19:6175–6182.
155 Cabib S, Pascucci T, Ventura R, Romano V, Puglisi-Allegra S: The behavioral profile of severe mental retardation in a genetic mouse model of phenylketonuria. Behav Genet 2003;33:301–310.
156 Pascucci T, Ventura R, Puglisi-Allegra S, Cabib S: Deficits in brain serotonin synthesis in a genetic mouse model of phenylketonuria. Neuroreport 2002;13:2561–2564.
157 Pascucci T, Andolina D, Mela IL, Conversi D, Latagliata C, Ventura R, Puglisi-Allegra S, Cabib S: 5-Hydroxytryptophan rescues serotonin response to stress in prefrontal cortex of hyperphenylalaninaemic mice. Int J Neuropsychopharmacol 2009;12: 1067–1079.
158 Coleman CM, Neckameyer WS: Serotonin synthesis by two distinct enzymes in *Drosophila melanogaster.* Arch Insect Biochem Physiol 2005;59:12–31.

159 Hendricks TJ, Fyodorov DV, Wegman LJ, Lelutiu NB, Pehek EA, Yamamoto B, Silver J, Weeber EJ, Sweatt JD, Deneris ES: Pet-1 *ETS* gene plays a critical role in 5-HT neuron development and is required for normal anxiety-like and aggressive behavior. Neuron 2003;37:233–247.
160 Lerch-Haner JK, Frierson D, Crawford LK, Beck SG, Deneris ES: Serotonergic transcriptional programming determines maternal behavior and offspring survival. Nat Neurosci 2008;11:1001–1003.
161 Zhao ZQ, Scott M, Chiechio S, Wang JS, Renner KJ, Gereau RW 4th, Johnson RL, Deneris ES, Chen ZF: *Lmx1b* is required for maintenance of central serotonergic neurons and mice lacking central serotonergic system exhibit normal locomotor activity. J Neurosci 2006;26:12781–12788.
162 Zhao ZQ, Chiechio S, Sun YG, Zhang KH, Zhao CS, Scott M, Johnson RL, Deneris ES, Renner KJ, Gereau RW 4th, Chen ZF: Mice lacking central serotonergic neurons show enhanced inflammatory pain and an impaired analgesic response to antidepressant drugs. J Neurosci 2007;27:6045–6053.
163 Agren H, Reibring L: PET studies of presynaptic monoamine metabolism in depressed patients and healthy volunteers. Pharmacopsychiatry 1994;27:2–6.
164 Turner EH, Loftis JM, Blackwell AD: Serotonin a la carte: supplementation with the serotonin precursor 5-hydroxytryptophan. Pharmacol Ther 2006;109:325–338.
165 Birdsall TC: 5-Hydroxytryptophan: a clinically-effective serotonin precursor. Altern Med Rev 1998;3:271–280.
166 Santarelli L, Saxe M, Gross C, Surget A, Battaglia F, Dulawa S, Weisstaub N, Lee J, Duman R, Arancio O, Belzung C, Hen R: Requirement of hippocampal neurogenesis for the behavioral effects of antidepressants. Science 2003;301:805–809.
167 Leonard BE: New approaches to the treatment of depression. J Clin Psychiatry 1996; 57(suppl 4):26–33.
168 Trivedi MH, Rush AJ, Wisniewski SR, Nierenberg AA, Warden D, Ritz L, Norquist G, Howland RH, Lebowitz B, McGrath PJ, Shores-Wilson K, Biggs MM, Balasubramani GK, Fava M: Evaluation of outcomes with citalopram for depression using measurement-based care in STAR*D: implications for clinical practice. Am J Psychiatry 2006;163:28–40.
169 Turner MS, May DB, Arthur RR, Xiong GL: Clinical impact of selective serotonin reuptake inhibitors therapy with bleeding risks. J Intern Med 2007;261:205–213.
170 Reeves RR, Wise PM, Cox SK: SSRIs and the risk of abnormal bleeding. J Psychosoc Nurs Ment Health Serv 2007;45:15–21.
171 Warden SJ, Robling AG, Haney EM, Turner CH, Bliziotes MM: The emerging role of serotonin (5-hydroxytryptamine) in the skeleton and its mediation of the skeletal effects of low-density lipoprotein receptor-related protein 5 (LRP5). Bone 2009 Jul 8. Epub ahead of print.
172 Perlis RH: Pharmacogenetic studies of antidepressant response: how far from the clinic? Psychiatr Clin North Am 2007;30: 125–138.
173 Liu Q, Yang Q, Sun W, Vogel P, Heydorn W, Yu XQ, Hu Z, Yu W, Jonas B, Pineda R, Calderon-Gay V, Germann M, O'Neill E, Brommage R, Cullinan E, Platt K, Wilson A, Powell D, Sands A, Zambrowicz B, Shi ZC: Discovery and characterization of novel tryptophan hydroxylase inhibitors that selectively inhibit serotonin synthesis in the gastrointestinal tract. J Pharmacol Exp Ther 2008;325:47–55.
174 Jin H, Cianchetta G, Devasagayaraj A, Gu K, Marinelli B, Samala L, Scott S, Stouch T, Tunoori A, Wang Y, Zang Y, Zhang C, David Kimball S, Main AJ, Ding ZM, Sun W, Yang Q, Yu XQ, Powell DR, Wilson A, Liu Q, Shi ZC: Substituted 3-(4-(1,3,5-triazin-2-yl)-phenyl)-2-aminopropanoic acids as novel tryptophan hydroxylase inhibitors. Bioorg Med Chem Lett 2009;19:5229–5232.
175 McKinney J, Knappskog PM, Haavik J: Different properties of the central and peripheral forms of human tryptophan hydroxylase. J Neurochem 2005;92:311–320.
176 Windahl MS, Boesen J, Karlsen PE, Christensen HE: Expression, purification and enzymatic characterization of the catalytic domains of human tryptophan hydroxylase isoforms. Protein J 2009 Oct 9. Epub ahead of print.
177 Nakata H, Fujisawa H: Purification and properties of tryptophan 5-monooxygenase from rat brain-stem. Eur J Biochem 1982;122:41–47.
178 Nakata H, Fujisawa H: Tryptophan 5-monooxygenase from mouse mastocytoma P815. A simple purification and general properties. Eur J Biochem 1982;124:595–601.
179 McKinney J, Knappskog PM, Pereira J, Ekern T, Toska K, Kuitert BB, Levine D, Gronenborn AM, Martinez A, Haavik J: Expression and purification of human tryptophan hydroxylase from *Escherichia coli* and *Pichia pastoris*. Protein Expr Purif 2004;33:185–194.
180 Murphy KL, Zhang X, Gainetdinov RR, Beaulieu JM, Caron MG: A regulatory domain in the N terminus of tryptophan hydroxylase 2 controls enzyme expression. J Biol Chem 2008;283:13216–13224.
181 Carkaci-Salli N, Flanagan JM, Martz MK, Salli U, Walther DJ, Bader M, Vrana KE: Functional domains of human tryptophan hydroxylase 2 (hTPH1). J Biol Chem 2006; 281:28105–28112.
182 Thorolfsson M, Ibarra-Molero B, Fojan P, Petersen SB, Sanchez-Ruiz JM, Martinez A: L-Phenylalanine binding and domain organization in human phenylalanine hydroxylase: a differential scanning calorimetry study. Biochemistry 2002;41:7573–7585.
183 Thorolfsson M, Doskeland AP, Muga A, Martinez A: The binding of tyrosine hydroxylase to negatively charged lipid bilayers involves the N-terminal region of the enzyme. FEBS Lett 2002;519:221–226.
184 Winge I, McKinney JA, Ying M, D'Santos CS, Kleppe R, Knappskog PM, Haavik J: Activation and stabilization of human tryptophan hydroxylase 2 by phosphorylation and 14-3-3 binding. Biochem J 2008; 410:195–204.
185 Kuhn DM, Sakowski SA, Geddes TJ, Wilkerson C, Haycock JW: Phosphorylation and activation of tryptophan hydroxylase 2: identification of serine-19 as the substrate site for calcium, calmodulin-dependent protein kinase II. J Neurochem 2007;103: 1567–1573.
186 Moy LY, Tsai LH: Cyclin-dependent kinase 5 phosphorylates serine 31 of tyrosine hydroxylase and regulates its stability. J Biol Chem 2004;279:54487–54493.
187 Royo M, Fitzpatrick PF, Daubner SC: Mutation of regulatory serines of rat tyrosine hydroxylase to glutamate: effects on enzyme stability and activity. Arch Biochem Biophys 2005;434:266–274.
188 Johansen PA, Jennings I, Cotton RG, Kuhn DM: Phosphorylation and activation of tryptophan hydroxylase by exogenous protein kinase A. J Neurochem 1996;66:817–823.
189 Moran GR, Fitzpatrick PF: A continuous fluorescence assay for tryptophan hydroxylase. Anal Biochem 1999;266:148–152.
190 Walther DJ, Peter JU, Bader M: 7-Hydroxytryptophan, a novel, specific, cytotoxic agent for carcinoids and other serotonin-producing tumors. Cancer 2002;94:3135–3140.
191 Ke L, Qi ZY, Ping Y, Ren CY: Effect of SNP at position 40237 in exon 7 of the *TPH2* gene on susceptibility to suicide. Brain Res 2006;1122:24–26.
192 Lopez de Lara C, Brezo J, Rouleau G, Lesage A, Dumont M, Alda M, Benkelfat C, Turecki G: Effect of tryptophan hydroxylase-2 gene variants on suicide risk in major depression. Biol Psychiatry 2007;62:72–80.
193 Jollant F, Buresi C, Guillaume S, Jaussent I, Bellivier F, Leboyer M, Castelnau D, Malafosse A, Courtet P: The influence of four serotonin-related genes on decision-making in suicide attempters. Am J Med Genet B Neuropsychiatr Genet 2007;144B:615–624.
194 Yoon HK, Kim YK: *TPH2* –703G/T SNP may have important effect on susceptibility to suicidal behavior in major depression. Prog Neuropsychopharmacol Biol Psychiatry 2009;33:403–409.
