# Supplementary material for: 5-Hydroxytryptophan (5-HTP): Natural Occurrence, Analysis, Biosynthesis, Biotechnology, Physiology and Toxicology
Source: Int J Mol Sci. 2020 Dec 26;22(1):181. doi: 10.3390/ijms22010181 (PMC7796270; doi:10.3390/ijms22010181)
Supplement: Supplementary file 1 [file ijms-22-00181-s001.zip › 5-HTP.Data/PDF/2183587662/1-s2.0-S0006899306004550-main.pdf]

available at www.sciencedirect.com
SCIENCE DIRECT
www.elsevier.com/locate/brainres
BRAIN RESEARCH

Research Report

Differential tissue distribution of tryptophan hydroxylase isoforms 1 and 2 as revealed with monospecific antibodies

Stacey A. Sakowski[b], Timothy J. Geddes[a,d], David M. Thomas[a,d], Edi Levi[c], James S. Hatfield[c], Donald M. Kuhn[a,b,d,*]

[a]Department of Psychiatry and Behavioral Neurosciences, Wayne State University School of Medicine, USA
[b]Center for Molecular Medicine and Genetics, Wayne State University School of Medicine, USA
[c]Pathology and Laboratory Service, John D. Dingell VA Medical Center, Detroit, MI, USA
[d]Research and Development Service, John D. Dingell VA Medical Center, Detroit, MI, USA

ARTICLE INFO

Article history:
Accepted 13 February 2006
Available online 11 April 2006

Keywords:
Tryptophan hydroxylase
TPH1
TPH2
Monospecific antibodies
Serotonin

Abbreviations:
5HT, serotonin
αTPH1, antibody against TPH isoform 1
αTPH2, antibody against TPH isoform 2
BH_4, tetrahydrobiopterin
DTT, dithiothreitol
MW, molecular weight
PBS, phosphate-buffered saline
TBST, Tris-buffered saline containing Triton X-100
TPH, tryptophan hydroxylase
TPH1, TPH isoform 1
TPH2, TPH isoform 2

ABSTRACT

Tryptophan hydroxylase (TPH) is the rate-limiting enzyme in the synthesis of the neurotransmitter serotonin. Once thought to be a single-gene product, TPH is now known to exist in two isoforms—TPH1 is found in the pineal and gut, and TPH2 is selectively expressed in brain [Walther, D.J., Peter, J.U., Bashammakh, S., Hortnagl, H., Voits, M., Fink, H., Bader, M., 2003. Synthesis of serotonin by a second tryptophan hydroxylase isoform. Science 299, 76.]. Heretofore, probes used for localization of TPH protein or mRNA could not distinguish between the TPH isoforms because of extensive homology shared by them at the nucleotide and amino acid level. We have produced monospecific polyclonal antibodies against TPH1 and TPH2 using peptide antigens from nonoverlapping sequences in the respective proteins. These antibodies allow the differentiation of TPH1 and TPH2 upon immunoblotting, immunoprecipitation, and immunocytochemical staining of tissue sections from brain and gut. TPH1 and TPH2 antibodies do not cross-react with either tyrosine hydroxylase or phenylalanine hydroxylase. Analysis of mouse tissues confirms that TPH1 is the predominant form expressed in pineal gland and in P815 mastocytoma cells with a molecular weight of 51 kDa. TPH2 is the predominant enzyme form expressed in brain extracts from mesencephalic tegmentum, striatum, and hippocampus with a molecular weight of 56 kDa. Antibody specificity against TPH1 and TPH2 is retained across mouse, rat, rabbit, primate, and human tissues. Antibodies that distinguish between the isoforms of TPH will allow studies of the differential regulation of their expression in brain and periphery.

© 2006 Elsevier B.V. All rights reserved.

* *Corresponding author.* John D. Dingell VA Medical Center, Research and Development Service (11R), 4646 John R, Detroit, MI 48201, USA.
E-mail address: donald.kuhn@wayne.edu (D.M. Kuhn).

0006-8993/$ – see front matter © 2006 Elsevier B.V. All rights reserved.
doi:10.1016/j.brainres.2006.02.047

1. Introduction

Tryptophan hydroxylase (TPH, EC 1.14.16.4) is the initial and rate-limiting enzyme in the biosynthesis of serotonin (5HT). The vast majority of 5HT is found in the gut where it modulates motility and initiates peristaltic and secretory reflexes (Gershon, 2003, 2004). In brain, 5HT serves as a neurotransmitter where it mediates a large variety of physiological processes including sleep, food intake, and sexual behavior (Lucki, 1998). Alterations in 5HT synthesis and function in the gut can lead to abnormal gastrointestinal motility and other clinically significant disorders (Gershon, 2004), while disruptions in brain 5HT function are thought to play a role in various psychiatric conditions including anxiety, drug abuse, and mood control (Brawman-Mintzer and Yonkers, 2004; Johnson, 2004; Joiner et al., 2005).

It was generally accepted that TPH was a single-gene product and that tissue-specific differences in the enzyme's molecular or catalytic properties reflected differential translational efficiency (Dumas et al., 1989) or post-translational events (Kim et al., 1991; Kuhn et al., 1979, 1980). However, Walther et al. (2003) made the extremely interesting and important observation that TPH actually exists in two distinct molecular forms. The original isoform, referred to as TPH1, was first cloned from rabbit pineal gland (Grenett et al., 1987) and is found primarily in the gut and other locations outside of the central nervous system, including the pineal. The newly discovered isoform, referred to as TPH2, is selectively expressed by 5HT producing neurons in brain (Walther and Bader, 2003; Walther et al., 2003) and is thought to be the form of TPH that controls brain 5HT synthesis (Zhang et al., 2004).

Even though the TPH isoforms are distinct gene products (chromosomal locations of TPH1 and TPH2 are 7 and 10 in mice, respectively, and 11 and 12 in humans, respectively), they possess about 70% homology across species in their deduced amino acid sequences. Apart from the recent work of De Luca et al. (2005), probes used to study differential tissue expression of either enzyme form at the nucleotide or protein level generally have not yielded results that are specific for TPH1 or TPH2, reflecting the extensive similarities between them. We have used peptides from nonoverlapping regions in the deduced amino acid sequences of TPH1 and TPH2 as antigens to develop antibodies that are highly specific for each enzyme. These antibodies retain specificity against the TPH isoforms in immunoblotting, immunoprecipitation, and immunocytochemical applications, and they have broad cross-species reactivity, indicating their usefulness in studies examining differential expression of TPH in the brain or periphery.

2. Results

2.1. Immunoblotting

PH8, a pan-TPH monoclonal antibody, and affinity-purified polyclonal antibodies against TPH1 and TPH2 were tested for reactivity and specificity by probing blots containing recombinant forms of each enzyme isoform. Fig. 1 shows that PH8 recognizes both TPH1 and TPH2, with immunoreactivity observed at 51 kDa and 56 kDa, respectively. The antibody against TPH1 reacted with TPH1 protein and showed no cross-reactivity with TPH2. Similarly, the antibody against TPH2 reacted with TPH2 protein and showed no cross-reactivity with TPH1. Neither antibody against TPH reacted with purified tyrosine hydroxylase or phenylalanine hydroxylase (data not shown). The specificity of the TPH antibodies in reacting with the isoforms in different tissues was tested, and the results are presented in Fig. 2. Extracts from tissues expected to express the TPH1 isoform predominantly (i.e., pineal gland, P815 mastocytoma cells) or the TPH2 isoform predominantly (all brain tissues) were resolved by SDS-PAGE and blotted to nitrocellulose. It can be seen in Fig. 2A that αTPH1 reacted with a 51-kDa protein in pineal gland and P815 cells, consistent with TPH1, while showing no reactivity with either recombinant TPH2 or other proteins in brain tissue extracts. On the other hand, αTPH2 reacted with a 56-kDa protein in extracts from mesencephalon, striatum, and hippocampus, consistent with TPH2, while showing no reactivity with recombinant TPH1, or with pineal gland or P815 cell extracts, as shown in Fig. 2B. PH8 reacts with each form of TPH in tissue extracts as shown in Fig. 2C, as expected. Finally, the isoform-specific TPH antibodies were tested for cross-species reactivity and the results are presented in Fig. 3. αTPH1 recognized TPH1 protein from mouse, rat, and rabbit pineal gland (Fig. 3A), while αTPH2 did not show reactivity with pineal tissues (data not shown). Fig. 3B shows that αTPH2 recognized TPH2 from mouse, rat, rabbit, primate, and human mesencephalic tegmentum, while αTPH1 did not show reactivity with brain tissue (data not shown). These results establish that the antibodies against TPH1 and TPH2 are highly specific in their respective reactivity with the TPH protein isoforms. If immunoblots were processed in the presence of the TPH1 or TPH2 peptide antigens,

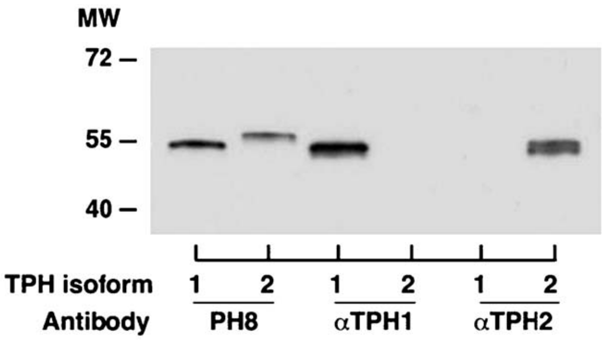

Fig. 1 – Immunoblotting of recombinant TPH1 and TPH2 with isoform-specific antibodies. Recombinant forms of TPH1 and TPH2 (10 ng each, per lane) were exposed to SDS-PAGE and blotted to nitrocellulose for immunoblotting. Blots were probed with PH8 (1:20,000), αTPH1 (1:20,000), or αTPH2 (1:20,000) for 1 h at room temperature. After exposure to horseradish peroxidase-conjugated secondary antibodies (1:4000 for each) for 1 h at room temperature, blots were washed, and immunoreactivity was visualized by enhanced chemiluminescence. MW standards are indicated in kilodalton.

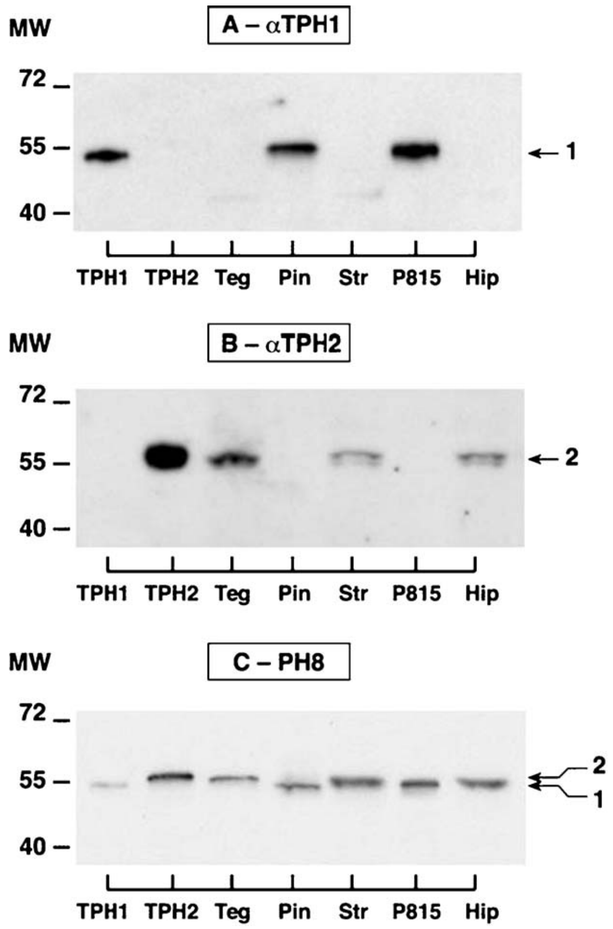

Fig. 2 – Immunoblotting of TPH1 and TPH2 in mouse tissues. Soluble extracts from mouse mesencephalic tegmentum (Teg, 30 μg), pineal (Pin, 20 μg), striatum (Str, 30 μg), P815 mastocytoma cells (P815, 5 μg), or hippocampus (Hip, 30 μg) were resolved by SDS-PAGE and blotted to nitrocellulose. Blots were probed with αTPH1 (A, 1:1000), αTPH2 (B, 1:1000) or PH8 (C, 1:20,000) overnight at 4 °C. After exposure to horseradish peroxidase-conjugated secondary antibodies (1:4000) for 1 h at room temperature, blots were washed, and TPH immunoreactivity was visualized by enhanced chemiluminescence. Lanes 1 and 2 of each panel contain recombinant TPH1 and TPH2 (10 ng each lane) as reference standards. Arrows on right side of each panel point to TPH1 (51 kDa) or TPH2 (56 kDa) immunoreactive bands. MW standards are indicated in kilodalton.

the reactivity of each antibody for its respective antigen (indicated by arrows in Figs. 2 and 3) was completely prevented (data not shown).

2.2. *Immunoprecipitation*

The antibodies against the TPH isoforms were tested for the ability to immunoprecipitate TPH protein from tissue, and the results are presented in Fig. 4. Antibodies against TPH1 precipitated recombinant TPH1 but not TPH2, and antibodies against TPH2 precipitated recombinant TPH2 but not TPH1 (data not shown). Treatment of extracts from P815 mastocytoma cells with antibodies against TPH1 or TPH2 revealed that αTPH1 precipitated TPH protein (51 kDa) in a concentration-dependent manner (Fig. 4A), whereas αTPH2 did not precipitate TPH1 (Fig. 4B). Similarly, when extracts from mouse mesencephalic tegmentum were treated with antibodies against TPH1 or TPH2, αTPH1 did not precipitate proteins (Fig. 4C), while αTPH2 captured a protein of 56 kDa, consistent with TPH2 in a concentration-dependent manner (Fig. 4D). Omission of primary antibodies from immunoprecipitate reactions demonstrated that neither TPH1 nor TPH2 proteins was binding directly to protein A beads.

If immunoprecipitation was carried out in the presence of the TPH1 or TPH2 peptide antigens, the reactivity of each antibody for its respective antigen (Figs. 4A and D) was completely prevented (data not shown). These results establish that the antibodies against TPH1 and TPH2 retain their specificity when used to immunoprecipitate TPH from tissue,

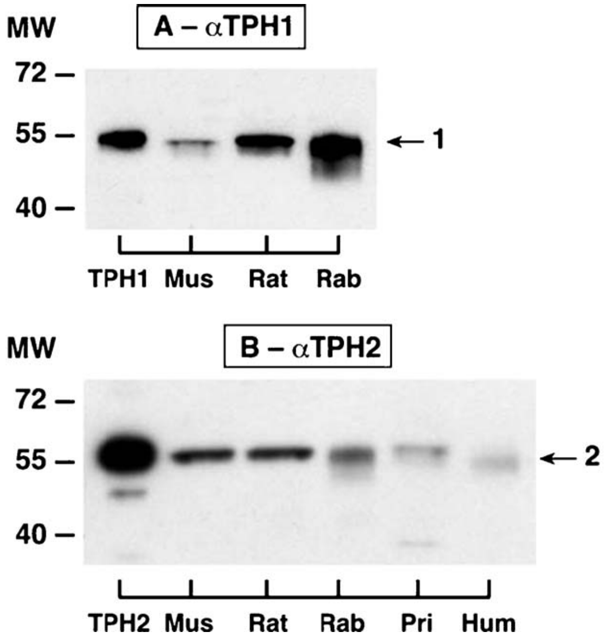

Fig. 3 – Cross-species reactivity of TPH1 and TPH2 isoform-specific antibodies. (A) Soluble extracts from mouse (Mus, 50 μg), rat (Rat, 5 μg), or rabbit (Rab, 1 μg) pineal gland or (B) from mouse (Mus, 45 μg), rat (Rat, 45 μg), rabbit (Rab, 450 ng), primate (Pri, 45 μg), or human (Hum, 10 μg) mesencephalic tegmentum were resolved by SDS-PAGE and blotted to nitrocellulose. Blots were probed with αTPH1 (1:1000) or αTPH2 (1:1000) overnight at 4 °C. After exposure to horseradish peroxidase-conjugated secondary antibodies (1:4000) for 1 h at room temperature, blots were washed, and TPH immunoreactivity was visualized by enhanced chemiluminescence. Lane 1 of each blot contains recombinant TPH1 (A) or TPH2 (B) as a reference standard (10 ng each). Because αTPH2 did not react with proteins in panel A, and αTPH1 did not react with proteins in panel B, these blank blots are not included in this figure. Arrows on right side of each panel point to TPH1 (51 kDa) or TPH2 (56 kDa) immunoreactive bands. MW standards are indicated in kilodalton.

and they agree very well with results from immunoblotting (see Figs. 1–3 above).

2.3. *Immunocytochemistry*

Sections were cut through paraformaldehyde-fixed mouse mesencephalon or duodenum and antibodies against TPH1 or TPH2 were used to probe for their respective antigen by immunocytochemistry. Fig. 5 shows that αTPH1 labeled only a small number of cells in the area of the dorsal raphe nucleus. In contrast, αTPH2 labeled a dense network of cell bodies and axonal processes in the mesencephalon of mice, in a pattern that is highly characteristic of 5HT-containing neurons (Steinbusch, 1981). Staining of TPH2-positive cell bodies was entirely cytoplasmic. Sections through the duodenum were extensively labeled with αTPH1 as shown in Fig. 5. TPH1 immunoreactivity was restricted to the basolateral side of the mucosa, consistent with the intracellular distribution of 5HT-containing enterochrommafin cells in the gut (Gershon, 2005; Nilsson et al., 1985). The detection of TPH1 in the gut using fluorescence microscopy did not allow a clear demonstration of the cellular location of the enzyme, so sections were stained with alkaline phosphatase-conjugated secondary antibodies and viewed under the light microscope. The inset to Fig. 5 shows TPH1 immunoreactivity (red stain) that is clearly limited to the granular enterochrommafin cells. Nuclei and other glandular cells of the mucosal crypts were not stained (Fig. 5 inset) nor was adjoining muscle tissue (data not shown). Antibodies against TPH2 did not reveal immunoreactivity in these areas of the gut, as expected, since the present studies focused on the lamina where TPH1 was anticipated to be the predominant enzyme form. αTPH2 is predicted to recognize TPH2 in enteric neuronal tissue (Gershon, 2005), and studies are underway to confirm this. If incubations of tissue sections with αTPH1 or αTPH2 were carried out in the presence of the TPH1 or TPH2 peptide antigens, respectively, the reactivity of each antibody for its respective antigen was completely prevented (data not shown).

3. Discussion

The present results describe the production of monospecific polyclonal antibodies against TPH1 and TPH2 through the use of nonoverlapping peptide sequences from the respective TPH isoforms as antigens. When paired with sequential exclusive and inclusive affinity chromatography of hyperimmune serum against their respective peptide antigens, followed by affinity chromatography against full length, recombinant forms of TPH1 or TPH2, the antibodies proved to be highly specific. Antibodies against TPH1 reacted with TPH1 protein and not with TPH2 protein. Similarly, antibodies against TPH2 reacted with TPH2 protein and not with TPH1. The isoform-specific antibodies against TPH did not cross-react with either tyrosine hydroxylase or phenylalanine hydroxylase from mouse. The antibodies against TPH1 and TPH2 retained their reactivity and specificity in immunoblotting, immunoprecipitation, and immunocytochemical applications, indicating their usefulness for studies that seek to examine differential expression of TPH protein in brain and periphery. The TPH1

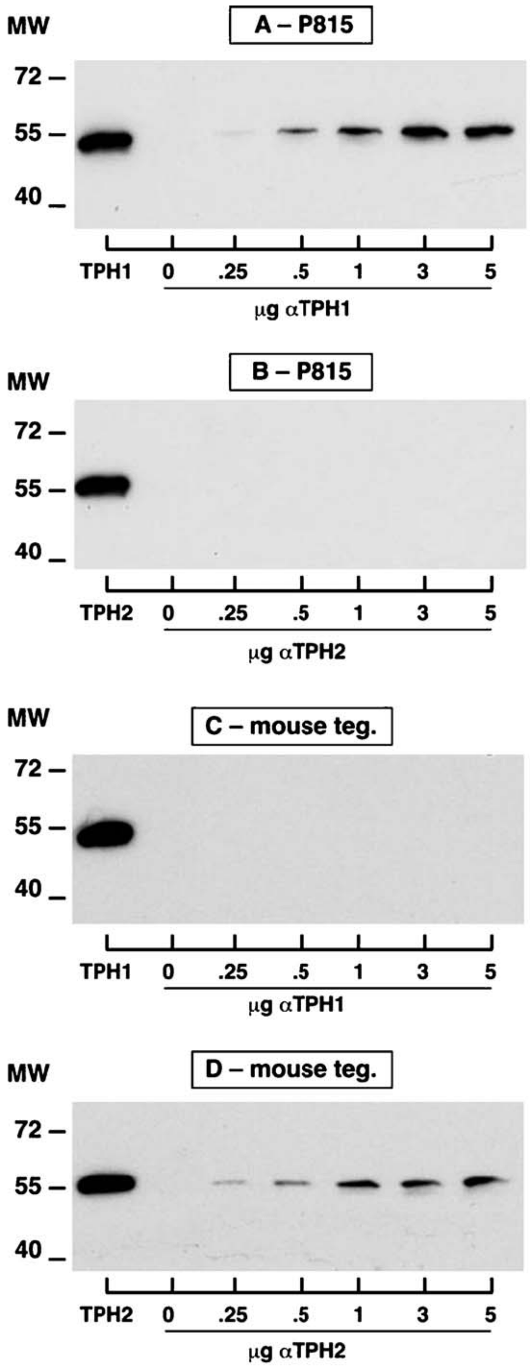

Fig. 4 – Immunoprecipitation of TPH1 and TPH2 from tissue using isoform-specific antibodies against TPH. Soluble extracts from P815 mouse mastocytoma cells (A and B) or mouse mesencephalic tegmentum (C and D) were incubated with the indicated amounts (μg) of either αTPH1 or TPH2 for 2 h at 4 °C. Protein A beads were added, and tubes were incubated for an additional 1 h at room temperature. Protein A beads were collected by centrifugation, washed, and captured TPH proteins were eluted with SDS-PAGE buffer. After resolution of immunoprecipitated proteins with SDS-PAGE, TPH immunoreactivity was detected with PH8 and enhanced chemiluminescence. MW standards are indicated in kilodalton.

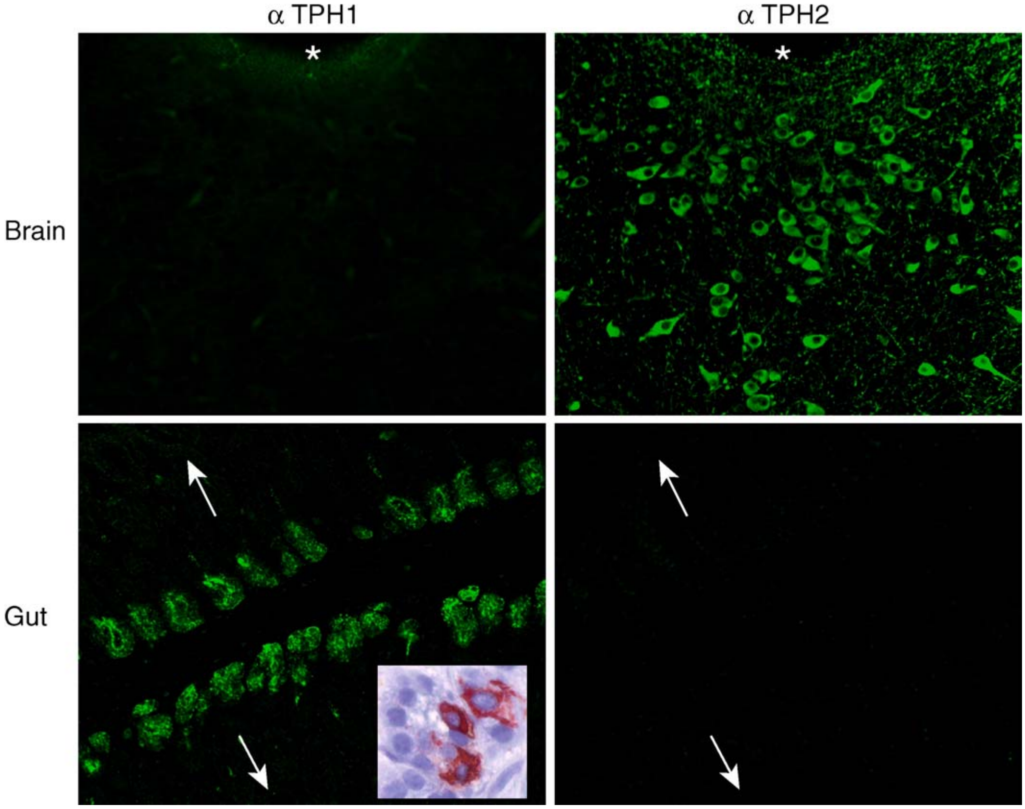

Fig. 5 – Immunocytochemical detection of TPH1 and TPH2 in brain and gut with isoform-specific antibodies. Sections from paraformaldehyde-fixed mouse brain from the area of the dorsal raphe (50 um) or gut (5 um) were cut and reacted with either αTPH1 (1:500) or αTPH2 (1:500) overnight at 4 °C. Sections were washed extensively and then reacted with Alexa Fluor 488 goat anti-rabbit secondary antibody (1:1000) for 30 min at room temperature. TPH immunoreactivity was visualized by fluorescence microscopy. The inset shows TPH1 immunoreactivity (red stain) in the mouse gut as detected by alkaline phosphatase-conjugated secondary antibodies. For orientation, the * symbols indicate the cerebral aqueduct in the brain sections while the arrows point toward the lumen of the duodenum and away from the basolateral area of the mucosa in the sections from gut. (For interpretation of the references to colour in this figure legend, the reader is referred to the web version of this article.)

and TPH2 antibodies also show broad reactivity against their respective antigens across species including mouse, rat, rabbit, primate, and human.

TPH was long considered to be a single-gene product until Walther et al. (2003) described a brain-specific isoform, now termed TPH2. Prior to this interesting discovery, differences in the molecular or catalytic properties of TPH were generally attributed to differential translational (Dumas et al., 1989) or post-translational events (Kim et al., 1991; Kuhn et al., 1979, 1980). Because TPH1 and TPH2 possess such extensive homology across species in their nucleotide and deduced amino acid sequences, most probes (antibodies or mRNA probes) used heretofore could not always distinguish between them. For example, antibodies raised against TPH purified from P815 mastocytoma cells (i.e., TPH1 isoform) react with gut but not brain TPH (Hasegawa et al., 1987), while other antibodies raised against the same antigen show cross-reactivity with both gut and brain TPH (Yu et al., 1999). Antibodies raised against recombinant TPH also recognize a 51-kDa protein (Dumas et al., 1989) or a 56-kDa protein (Haycock et al., 2002) in both pineal and brain. Although the monoclonal antibody MαWH showed preference for brain TPH (i.e., TPH2) and the polyclonal antibody SαWH showed preference for pineal TPH (i.e., TPH1), each retained some reactivity to TPH from pineal and brain, respectively (Haycock et al., 2002). A similar situation exists at the nucleotide level for TPH. For example, a gene containing a portion of the 5′ flanking region of mouse TPH fused to the coding region of lacZ resulted in high level lacZ expression in the pineal of transgenic mice but only moderate expression in the dorsal and median raphe nuclei (Huh et al., 1994). An antisense oligo based on a sequence in the region of the translation start codon of P815 TPH inhibited TPH activity after direct infusion into the brains of mice (McCarthy et al., 1995). Salli et al. (2004) also observed that neurons derived from rhesus monkey embryonic stem cells express both TPH1 and TPH2 transcripts.

The use of nonselective TPH probes to study its transcription and/or translation has not caused extensive confusion because of the rather selective tissue distribution of TPH—TPH1 is the predominant isoform in gut and pineal, and TPH2 is the predominant form in neuronal tissue. However, it is possible that some tissues may express both isoforms. The situation is quite clear in the gut where TPH1 is found in the enterochrommafin cells of the basolateral aspects of the mucosa, and TPH2 is located within the enteric nervous system (Gershon, 2005). The situation in brain is much less clear with

regard to expression of at least TPH1. We did note that a few cells in the area of the dorsal raphe are labeled with antibodies against TPH1 (Fig. 5) but to a much lesser extent than seen with TPH2 antibodies. We cannot rule out the possibility that this result represents slight cross-reactivity of αTPH1 with TPH2 protein, which was not evident to us in other tests of antibody specificity including immunoblotting and immunoprecipitation. On the other hand, the mRNA for TPH1 has been detected in the mesencephalon (Patel et al., 2004; Salli et al., 2004) where TPH2 mRNA predominates, and TPH2 mRNA has been detected in pineal where TPH1 predominates (Sugden, 2003). It is possible that detection of TPH1 or TPH2 in tissues with extremely low levels of expression would be facilitated through the use of immunocytochemistry, to allow the analysis of very discrete cell populations that contain one form or the other. Additional studies will be required to ascertain the extent to which TPH1 protein is translated in brain or the extent to which TPH2 is expressed in pineal, and such studies are now possible with the isoform-specific TPH antibodies.

A number of physiological conditions and pharmacological treatments are known to cause differential regulation of TPH transcription and possibly translation. For example, both TPH1 and TPH2 mRNAs are expressed under diurnal rhythms in pineal gland (Sugden, 2003) and retina (Liang et al., 2004). Glucocorticoids increase TPH mRNA in pineal and decrease its expression in brain (Clark et al., 2005; Clark and Russo, 1997), and ovarian steroids stimulate TPH2 mRNA expression in the raphe region of macaque monkeys (Sanchez et al., 2005). Repeated immobilization stress significantly increases TPH mRNA levels in the raphe nuclei but not in pineal gland (Chamas et al., 1999, 2004). Finally, neurotoxic doses of MDMA increase TPH mRNA levels in brain while causing decreases in TPH protein levels (Austin, 2004). Taken together, considerable evidence points to the possibility that TPH1 and TPH2 are differentially regulated at both transcriptional and translational levels, and the availability of isoform-specific antibodies against TPH1 and TPH2 should make it possible to determine which form of the enzyme is altered in expression in various tissues.

4. Experimental procedures

4.1. Materials

Glutathione, glutathione-agarose, dithiothreitol (DTT), ferrous ammonium sulfate, and tryptophan were obtained from Sigma Aldrich Chemical Company (St. Louis, MO). Tetrahydrobiopterin (BH_4) was obtained from Dr. B. Schircks Laboratories (Jona, Switzerland). The pGEX-4T vectors, sheep anti-mouse IgG and donkey anti-rabbit IgG conjugated to horseradish peroxidase, thrombin protease, and Protein A Sepharose CL-4B were products of Amersham Biosciences (Piscataway, NJ). P815 mastocytoma cells were obtained from ATCC (Manassas, VA). DMEM and all tissue culture related reagents and Alexa Fluor 488 goat anti-rabbit IgG were from Invitrogen (Carlsbad, CA). Western Lightning Chemiluminescence Reagent Plus was purchased from PerkinElmer Life Sciences (Boston, MA), and Biomax MR film was a product of Kodak (Rochester, NY). Vectastain ABC kits and Vectashield were obtained from Vector Laboratories (Burlingame, CA). The monoclonal antibody PH8, which has reactivity toward TPH from brain and pineal (Haan et al., 1987; Haycock et al., 2002) was provided by Dr. Richard G.H. Cotton. Tyrosine hydroxylase was produced in recombinant form and purified as previously described (Kuhn et al., 1999). Phenylalanine hydroxylase was purified from mouse liver by substrate-induced hydrophobic interaction chromatography (Shiman et al., 1979).

4.2. *Preparation of recombinant TPH1 and TPH2 and assays of catalytic activity*

Recombinant TPH1 (D'Sa et al., 1996) and TPH2 (Sakowski et al., 2006) were expressed as glutathione S-transferase fusion proteins and purified by affinity chromatography and thrombin cleavage from the fusion tag. Enzymes were judged to be >95% pure by SDS-PAGE. TPH activity was assayed for 15 min at 37 °C as previously reported (Kuhn and Arthur, 1996). The standard reaction mixture contained 50 mM Tris pH 7.4, 1 mM DTT, 0.05 mg/ml catalase, 200 μM tryptophan, 100 μM ferrous ammonium sulfate, and 200 μM BH_4.

4.3. *Antibody production*

Peptides corresponding to the sequence L^{435}ARVSRWPSV^{444} in the C-terminal region of TPH1 and R^{15}RGLSLDSAV-PEDHQL^{30} in the N-terminal region of TPH2 were conjugated to Keyhole Limpet hemocyanin and used as immunogens in rabbits by Bethyl Laboratories (Montgomery, TX). These regions of TPH1 and TPH2 do not share any sequence homology with each other. Hyperimmune serum from rabbits immunized with the TPH1 peptide sequence was chromatographed over a column of immobilized TPH2 peptide, and antibodies in the effluent were captured on a column of immobilized TPH1 peptide. Serum from rabbits immunized with the TPH2 peptide was similarly cross-affinity purified. Antibodies against TPH1 and TPH2 were subjected to additional purification by affinity chromatography against full-length recombinant TPH1 or TPH2 protein, respectively. Just prior to use, each antibody was precleared with soluble mouse extract from brain areas not expressing TPH1 (e.g., striatum in the case of αTPH1) or TPH2 (e.g., cerebellum in the case of αTPH2). Preimmune serum was collected from subjects prior to immunization. Antibodies were stored at 4 °C in 0.1% sodium azide.

4.4. *Tissue preparation*

Brain and duodenum were obtained from C57BL/6 mice. After sacrifice, pineal glands were harvested, and the mesencephalic tegmentum (containing the densest concentration of 5HT-producing cell bodies in brain), striatum, and hippocampus were dissected from brain and stored frozen at −80 °C. When fixation of brain was required, mice were deeply anesthetized with pentobarbital (120 mg/kg) and perfused transcardially with ice-cold 4% paraformaldehyde. Brains were removed and stored overnight in fixative at 4 °C. For preparation of duodenum, fresh tissue was dissected and

washed repeatedly with phosphate-buffered saline (PBS). Duodenum was cut longitudinally into strips and placed into ice-cold 4% paraformaldehyde for 4 h. Thereafter, duodenal tissue was dehydrated through a series of graded ethanol washes and embedded in paraffin. Unfixed primate brain (*Macaca fascicularis*, female) was provided by the National Primate Research Center at the University of Washington. Unfixed human brain was generously donated by Dr. Alan Hudson (Department of Immunology and Microbiology, Wayne State University School of Medicine). Primate and human mesencephalic tegmentum were prepared for use in the same manner as unfixed mouse brain tissue. P815 mouse mastocytoma cells were maintained in culture using DMEM containing 10% fetal bovine serum and 100 units/ml penicillin–streptomycin as growth media. Cells were harvested when they reached about 80% confluence, washed to remove media, and then stored frozen at −80 °C. Antibody dilutions for use in different techniques were determined empirically (see below). All experiments using mice conformed to NIH and international guidelines on the ethical use of animals and were designed to minimize both the number of animals used and any pain experienced. Specific animal protocols were approved by the Animal Investigation Committee and Division of Laboratory Animal Resources at Wayne State University.

4.5. *Immunodetection of TPH isoforms*

For immunoblotting, purified recombinant TPH1 or TPH2 (10–100 ng protein) or soluble extracts from brain or P815 mastocytoma cells were exposed to SDS-PAGE using 10% acrylamide and blotted to nitrocellulose. Prior to SDS-PAGE and blotting of mouse brain extracts, tubulin was removed by differential ion-exchange chromatography because it obscures TPH immunoreactivity, as previously described (Johansen et al., 1996). After blocking in 5% nonfat dry milk in Tris-buffered saline containing 0.01% Triton X-100 (TBST) overnight at 4 °C, blots were incubated with affinity purified antibodies against TPH1, TPH2, or to the pan-TPH antibody PH8 as specified below. Unbound primary antibodies were removed by washing in TBST (3×, 10 min per wash at room temperature), and donkey anti-rabbit IgG conjugated to horseradish peroxidase was incubated with blots at a dilution of 1:4000 for 1 h at room temperature. TPH1 and TPH2 immunoreactivity was subsequently visualized with enhanced chemiluminescence. For immunoprecipitation, recombinant TPH1 or TPH2 was incubated with either isoform-specific antibody for 2 h at 4 °C. Protein A beads, previously blocked with 5% nonfat dry milk in TBST, were added and incubated for 1 h at room temperature. The Protein A beads were pelleted by centrifugation, washed, and captured TPH protein was eluted with SDS-PAGE buffer followed by immunoblotting for TPH with PH8. Isoform-specific antibodies were also used to immunoprecipitate TPH from extracts of brain and P815 mastocytoma cells as described above for recombinant proteins. Brain extracts and P815 cells were prepared by homogenization in PBS containing 1 mM DTT and insoluble material was removed by centrifugation (30,000 × *g*, 10 min at 4 °C). TPH immunoprecipitated from tissues was detected by PH8 as described above. Immunoblotting was used to monitor the effectiveness of immunoprecipitation, in place of enzyme activity assay, because the TPH1 and TPH2 antibodies inhibited TPH catalysis (data not shown). Dilutions for antibodies used in immunoblotting and immunoprecipitation are specified in the figure legends. For immunocytochemistry, sections of 50 μm were cut through the dorsal raphe area of mouse brain using a Vibratome and floated into PBS. Sections were incubated in blocking buffer (PBS containing 0.3% Triton X-100, 1% bovine serum albumin, and 5% normal goat serum) for 60 min at room temperature prior to incubation with αTPH1 or αTPH2 (each diluted 1:500 in blocking buffer) overnight at 4 °C. Following repeated washes with PBS containing 0.3% Triton X-100, Alexa Fluor 488 goat anti-rabbit secondary antibody (diluted 1:1000 in blocking buffer) was applied for 30 min at room temperature, then sections were washed three times with PBS containing 0.3% Triton X-100 and once with PBS. Sections were transferred to slides and cover slipped under Vectashield. Duodenal tissue sections of 5 μm thickness were cut after removal of paraffin, and sections were probed with TPH antibodies in the same manner described above for brain sections. TPH1 and TPH2 immunoreactivity was visualized by fluorescence microscopy using an Olympus IX81 microscope equipped with a Retiga 1300 CCD camera. In some cases, duodenal sections were incubated with Vectastain ABC kits with alkaline peroxidase after exposure to primary antibodies and TPH immunoreactivity was viewed using an Olympus BX40 light microscope. Preimmune serum did not bind to recombinant TPH1 or TPH2 on immunoblots nor did it bind to tissue in immunoblot, immunoprecipitation, or immunocytochemical studies (data not shown).

Acknowledgments

Supported by National Institute on Drug Abuse grants DA10756, DA014392, and a VA Merit Award. SAS was supported by NIDA grant T32 DA07310. This research was also supported in part by NIH grant RR00166 to the National Primate Research Center at the University of Washington. The authors thank Dr. R.G.H. Cotton for the generous gift of PH8, Carole Elliott for providing the primate brain, Dr. Alan Hudson for the sample of human brain tissue, Dr. Jim Granneman for his assistance with the Olympus IX81 fluorescence microscope, and Dr. Xiaotan Qiao (University of Michigan) for her assistance with tissue preparation from gut.

REFERENCES

Austin, M.C., 2004. Ecstasy (MDMA)-induced alterations in tryptophan hydroxylase protein and mRNA expression in the dorsal and median raphe nuclei. Abstr.-Soc. Neurosci. 689, 688.

Brawman-Mintzer, O., Yonkers, K.A., 2004. New trends in the treatment of anxiety disorders. CNS Spectr. 9, 19–27.

Chamas, F., Serova, L., Sabban, E.L., 1999. Tryptophan hydroxylase mRNA levels are elevated by repeated immobilization stress in

rat raphe nuclei but not in pineal gland. Neurosci. Lett. 267, 157–160.
Chamas, F.M., Underwood, M.D., Arango, V., Serova, L., Kassir, S.A., Mann, J.J., Sabban, E.L., 2004. Immobilization stress elevates tryptophan hydroxylase mRNA and protein in the rat raphe nuclei. Biol. Psychiatry 55, 278–283.
Clark, M.S., Russo, A.F., 1997. Tissue-specific glucocorticoid regulation of tryptophan hydroxylase mRNA levels. Brain Res. Mol. Brain Res. 48, 346–354.
Clark, J.A., Pai, L.Y., Flick, R.B., Rohrer, S.P., 2005. Differential hormonal regulation of tryptophan hydroxylase-2 mRNA in the murine dorsal raphe nucleus. Biol. Psychiatry 57, 943–946.
D'Sa, C.M., Arthur Jr., R.E., Kuhn, D.M., 1996. Expression and deletion mutagenesis of tryptophan hydroxylase fusion proteins: delineation of the enzyme catalytic core. J. Neurochem. 67, 917–926.
De Luca, V., Likhodi, O., Van Tol, H.H., Kennedy, J.L., Wong, A.H., 2005. Tryptophan hydroxylase 2 gene expression and promoter polymorphisms in bipolar disorder and schizophrenia. Psychopharmacology (Berlin) 183, 378–382.
Dumas, S., Darmon, M.C., Delort, J., Mallet, J., 1989. Differential control of tryptophan hydroxylase expression in raphe and in pineal gland: evidence for a role of translation efficiency. J. Neurosci. Res. 24, 537–547.
Gershon, M.D., 2003. Plasticity in serotonin control mechanisms in the gut. Curr. Opin. Pharmacol. 3, 600–607.
Gershon, M.D., 2004. Review article: serotonin receptors and transporters—roles in normal and abnormal gastrointestinal motility. Aliment. Pharmacol. Ther. 20 (Suppl. 7), 3–14.
Gershon, M.D., 2005. Nerves, reflexes, and the enteric nervous system: pathogenesis of the irritable bowel syndrome. J. Clin. Gastroenterol. 39, S184–S193.
Grenett, H.E., Ledley, F.D., Reed, L.L., Woo, S.L., 1987. Full-length cDNA for rabbit tryptophan hydroxylase: functional domains and evolution of aromatic amino acid hydroxylases. Proc. Natl. Acad. Sci. U. S. A. 84, 5530–5534.
Haan, E.A., Jennings, I.G., Cuello, A.C., Nakata, H., Fujisawa, H., Chow, C.W., Kushinsky, R., Brittingham, J., Cotton, R.G., 1987. Identification of serotonergic neurons in human brain by a monoclonal antibody binding to all three aromatic amino acid hydroxylases. Brain Res. 426, 19–27.
Hasegawa, H., Yanagisawa, M., Inoue, F., Yanaihara, N., Ichiyama, A., 1987. Demonstration of non-neural tryptophan 5-mono-oxygenase in mouse intestinal mucosa. Biochem. J. 248, 501–509.
Haycock, J.W., Kumer, S.C., Lewis, D.A., Vrana, K.E., Stockmeier, C.A., 2002. A monoclonal antibody to tryptophan hydroxylase: applications and identification of the epitope. J. Neurosci. Methods 114, 205–212.
Huh, S.O., Park, D.H., Cho, J.Y., Joh, T.H., Son, J.H., 1994. A 6.1 kb 5′ upstream region of the mouse tryptophan hydroxylase gene directs expression of E. coli lacZ to major serotonergic brain regions and pineal gland in transgenic mice. Brain Res. Mol. Brain Res. 24, 145–152.
Johansen, P.A., Jennings, I., Cotton, R.G., Kuhn, D.M., 1996. Phosphorylation and activation of tryptophan hydroxylase by exogenous protein kinase A. J. Neurochem. 66, 817–823.
Johnson, B.A., 2004. Role of the serotonergic system in the neurobiology of alcoholism: implications for treatment. CNS Drugs 18, 1105–1118.
Joiner Jr., T.E., Brown, J.S., Wingate, L.R., 2005. The psychology and neurobiology of suicidal behavior. Annu. Rev. Psychol. 56, 287–314.
Kim, K.S., Wessel, T.C., Stone, D.M., Carver, C.H., Joh, T.H., Park, D.H., 1991. Molecular cloning and characterization of cDNA encoding tryptophan hydroxylase from rat central serotonergic neurons. Brain Res. Mol. Brain Res. 9, 277–283.
Kuhn, D.M., Arthur Jr., R.E., 1996. Inactivation of brain tryptophan hydroxylase by nitric oxide. J. Neurochem. 67, 1072–1077.
Kuhn, M.D., Rosenberg, R.C., Lovenberg, W., 1979. Determination of some molecular parameters of tryptophan hydroxylase from rat midbrain and murine mast cell. J. Neurochem. 33, 15–21.
Kuhn, D.M., Meyer, M.A., Lovenberg, W., 1980. Comparisons of tryptophan hydroxylase from a malignant murine mast cell tumor and rat mesencephalic tegmentum. Arch. Biochem. Biophys. 199, 355–361.
Kuhn, D.M., Arthur Jr., R.E., Thomas, D.M., Elferink, L.A., 1999. Tyrosine hydroxylase is inactivated by catechol-quinones and converted to a redox-cycling quinoprotein: possible relevance to Parkinson's disease. J. Neurochem. 73, 1309–1317.
Liang, J., Wessel III, J.H., Iuvone, P.M., Tosini, G., Fukuhara, C., 2004. Diurnal rhythms of tryptophan hydroxylase 1 and 2 mRNA expression in the rat retina. NeuroReport 15, 1497–1500.
Lucki, I., 1998. The spectrum of behaviors influenced by serotonin. Biol. Psychiatry 44, 151–162.
McCarthy, M.M., Nielsen, D.A., Goldman, D., 1995. Antisense oligonucleotide inhibition of tryptophan hydroxylase activity in mouse brain. Regul. Pept. 59, 163–170.
Nilsson, O., Ericson, L.E., Dahlstrom, A., Ekholm, R., Steinbusch, H.W., Ahlman, H., 1985. Subcellular localization of serotonin immunoreactivity in rat enterochromaffin cells. Histochemistry 82, 351–355.
Patel, P.D., Pontrello, C., Burke, S., 2004. Robust and tissue-specific expression of TPH2 versus TPH1 in rat raphe and pineal gland. Biol. Psychiatry 55, 428–433.
Sakowski, S.A., Geddes, T.J., Kuhn, D.M., 2006. Mouse tryptophan hydroxylase isoform 2 and the role of proline 447 in enzyme function. J. Neurochem. 96, 758–765.
Salli, U., Reddy, A.P., Salli, N., Lu, N.Z., Kuo, H.C., Pau, F.K., Wolf, D.P., Bethea, C.L., 2004. Serotonin neurons derived from rhesus monkey embryonic stem cells: similarities to CNS serotonin neurons. Exp. Neurol. 188, 351–364.
Sanchez, R.L., Reddy, A.P., Centeno, M.L., Henderson, J.A., Bethea, C.L., 2005. A second tryptophan hydroxylase isoform, TPH-2 mRNA, is increased by ovarian steroids in the raphe region of macaques. Brain Res. Mol. Brain Res. 135, 194–203.
Shiman, R., Gray, D.W., Pater, A., 1979. A simple purification of phenylalanine hydroxylase by substrate-induced hydrophobic chromatography. J. Biol. Chem. 254, 11300–11306.
Steinbusch, H.W., 1981. Distribution of serotonin-immunoreactivity in the central nervous system of the rat-cell bodies and terminals. Neuroscience 6, 557–618.
Sugden, D., 2003. Comparison of circadian expression of tryptophan hydroxylase isoform mRNAs in the rat pineal gland using real-time PCR. J. Neurochem. 86, 1308–1311.
Walther, D.J., Bader, M., 2003. A unique central tryptophan hydroxylase isoform. Biochem. Pharmacol. 66, 1673–1680.
Walther, D.J., Peter, J.U., Bashammakh, S., Hortnagl, H., Voits, M., Fink, H., Bader, M., 2003. Synthesis of serotonin by a second tryptophan hydroxylase isoform. Science 299, 76.
Yu, P.L., Fujimura, M., Okumiya, K., Kinoshita, M., Hasegawa, H., Fujimiya, M., 1999. Immunohistochemical localization of tryptophan hydroxylase in the human and rat gastrointestinal tracts. J. Comp. Neurol. 411, 654–665.
Zhang, X., Beaulieu, J.M., Sotnikova, T.D., R.R. Gainetdinov, M.G., 2004. Caron Tryptophan hydroxylase-2 controls brain serotonin synthesis. Science 305, 217.
