# Supplementary material for: 5-Hydroxytryptophan (5-HTP): Natural Occurrence, Analysis, Biosynthesis, Biotechnology, Physiology and Toxicology
Source: Int J Mol Sci. 2020 Dec 26;22(1):181. doi: 10.3390/ijms22010181 (PMC7796270; doi:10.3390/ijms22010181)
Supplement: Supplementary file 1 [file ijms-22-00181-s001.zip › 5-HTP.Data/PDF/2418627606/v59-200.pdf]

of reagent grade benzene. The flask was kept at 0° C by means of an ice–salt bath. After 5 minutes, the solution had turned dark. The addition of hydrogen iodide was continued for 1 hour. After the mixture had been left to stand for 30 minutes at 0° C, it was freed of excess hydrogen iodide by vigorous gassing with dry nitrogen. The mixture was kept overnight at 0° C after which the titanium iodide was filtered off. The product was recrystallized, then dried under reduced pressure of 25 mm Hg, and sealed in a bent glass tube about 30 cm in length. Finally, the product was sublimed by immersing one end of the tube in an ice–salt bath while gently heating the other end. The purple solid collected in the cold part of the tube. A 70% yield, based on $TiCl_4$ was obtained. Analysis: The titanium was precipitated as the oxide, ignited, and weighed. The iodide was determined by preliminary oxidation with KIO_3, followed by titration with standard $Na_2S_2O_3$ solution. The following are mean values of three determinations:

Calculated values for	Ti = 8.62%	I = 91.38%
Experimental values for	Ti = 8.49%	I = 91.46%

The product is extremely hygroscopic, fuming strongly in air. It is readily soluble in water, forming a brown-colored solution. On standing for several weeks out of contact with the air, the brown solution becomes colorless. The product is soluble without reaction in benzene and carbon tetrachloride.

1. INORGANIC SYNTHESES. *Edited by* W. C. Fernelius. Vol. II. McGraw-Hill Book Co., Inc., New York. 1946. (*a*) p. 114, (*b*) p. 115.
2. SCOTT, W. W. Standard methods of chemical analysis. Vol. I. 5th ed. D. Van Nostrand Company, Inc., 1939. (*a*) p. 191, (*b*) p. 454, (*c*) p. 981.

RECEIVED MARCH 5, 1959.
DEPARTMENT OF CHEMISTRY,
WAYNE STATE UNIVERSITY,
DETROIT 2, MICHIGAN.

Can. J. Chem. Downloaded from cdnsciencepub.com by 2.235.186.236 on 11/16/20
For personal use only.

A NEW SYNTHESIS OF 5-HYDROXYTRYPTOPHAN*

GERASSIMOS FRANGATOS AND FRANCIS L. CHUBB

The biological significance of 5-hydroxytryptophan has been established. It is an important metabolite of tryptophan and is a precursor of serotonin (5-hydroxytryptamine) (1). In recent years serotonin has been the subject of an extensive study by many workers due to its potential importance in the function of the autonomic and central nervous system (2).

The first synthesis of 5-hydroxytryptophan was reported by Ek and Witkop (3), and is an application of the gramine synthesis developed by Snyder and Smith (4). A second synthesis of 5-hydroxytryptophan, which is also an application of the gramine synthesis, has recently been reported by Koo, Avakian, and Martin (5). The synthesis herein reported is an application of the convenient tryptophan synthesis of Warner and Moe (6). This method eliminates the difficult and tedious preparation of 5-benzyloxyindole.

The p-benzyloxyphenylhydrazone of γ,γ-dicarbethoxy-γ-acetamido-butyraldehyde (I) was prepared, and cyclized, without isolation, to form ethyl β-(5-benzyloxyindolyl-3)-α-carbethoxy-α-acetamidopropionate (II). This was identical with material prepared

**Based on a paper presented at the 41st Annual Conference of the Chemical Institute of Canada, Toronto, May 26–28, 1958.*

from the reaction of ethyl acetamidomalonate with 5-benzyloxygramine. Saponification and partial decarboxylation of II, followed by hydrolysis of the acetamido group, gave 5-benzyloxytryptophan (III). 5-Hydroxytryptophan (IV) was obtained by hydrogenolysis of III.

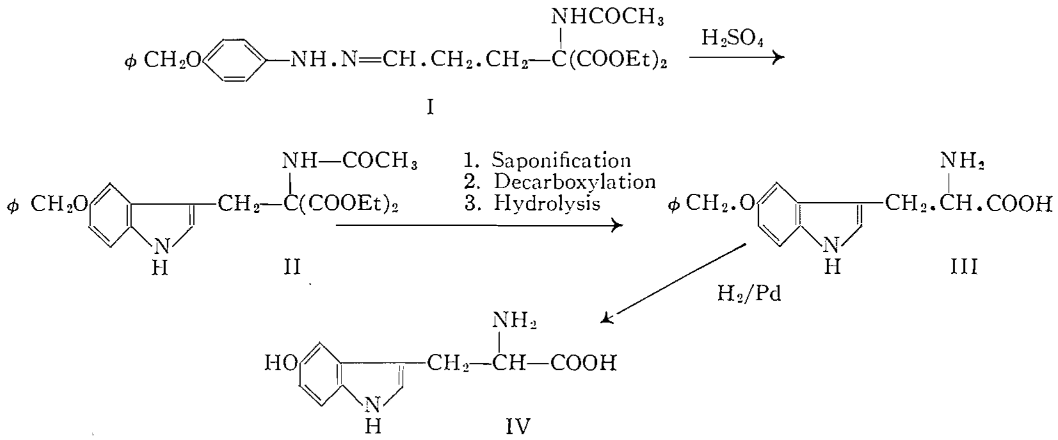

EXPERIMENTAL

Ethyl β-(5-Benzyloxyindolyl-3)-α-carbethoxy-α-acetamidopropionate (II)

A mixture of 85 g of ethyl acetamidomalonate (4), 0.4 g of sodium methoxide, and 200 ml of absolute ethanol was cooled to 0° with stirring. A thin slurry formed and then 31 ml of acrolein was added during 1.5 hours, keeping the temperature at 0–3°. The stirring was continued for 2 hours at 0–3°. Then 85 g of *p*-benzyloxyphenylhydrazine (7) and 10 ml of glacial acetic acid were added with vigorous stirring. The reaction mixture was heated to 50°, with stirring, and maintained at that temperature until the hydrazine went into solution. The resulting dark solution of II was kept at room temperature overnight. It was then transferred to a three-necked flask and water was added to bring the total volume to 1.5 liters. After 70 ml of concentrated sulphuric acid was added, the mixture was stirred vigorously and refluxed for 3 hours. The ethyl β-(5-benzyloxyindolyl-3)-α-carbethoxy-α-acetamidopropionate soon crystallized. The reaction mixture was cooled and filtered to yield 160 g of crude product. Three recrystallizations from toluene followed by one from aqueous ethanol gave 135 g (75.5%) of pure II, m.p. 169°, undepressed on admixture with an authentic sample prepared according to ref. 5. Anal. Calculated for $C_{25}H_{28}N_2O_6$: C, 66.36; H, 6.24; N, 6.19. Found: C, 66.60; H, 6.28; N, 6.08.

5-Benzyloxytryptophan (III)

The ester II (50 g) was saponified, decarboxylated, and deacetylated according to the procedure of Koo, Avakian, and Martin (5). The intermediate dicarboxylic acid was not purified before decarboxylation and deacetylation to form 5-benzyloxytryptophan (III).

The crude III was purified according to the following procedure. 5-Benzyloxytryptophan (20 g) was warmed with 100 ml of water and 1 liter of ethanol. If solution was not complete then 1% sodium hydroxide was added dropwise until the material all dissolved. After treatment with charcoal and filtration, the solution was cooled and adjusted to pH 6.6 with acetic acid. The 5-benzyloxytryptophan began to crystallize

Can. J. Chem. Downloaded from cdnsciencepub.com by 2.235.186.236 on 11/16/20
For personal use only.

immediately. In case it separated as a gel, warming and stirring converted it to the crystalline form. A total of 29 g (88.5%) of III, m.p. 276–280° was obtained.

5-Hydroxytryptophan (IV)

5-Hydroxytryptophan was prepared by catalytic hydrogenolysis of III according to the procedure of Koo, Avakian, and Martin (5).

1. Udenfriend, S., Titus, E., Weissbach, H., and Peterson, R. E. J. Biol. Chem. **219**, 335 (1956).
2. Page, I. H. Physiol. Rev. **38**, 277 (1958).
3. Ek, A. and Witkop, B. J. Am. Chem. Soc. **76**, 5579 (1954).
4. Snyder, H. R. and Smith, C. W. J. Am. Chem. Soc. **66**, 350 (1944).
5. Koo, J., Avakian, S., and Martin, G. J. J. Org. Chem. **24**, 179 (1959).
6. Warner, D. T. and Moe, O. A. J. Am. Chem. Soc. **70**, 2763 (1948).
7. Mentzer, C., Beaudet, C., and Bory, M. Bull. soc. chim. France, 421 (1953).

Received April 24, 1959.
Research Laboratories,
Frank W. Horner Limited,
Montreal, Quebec.

Can. J. Chem. Downloaded from cdnsciencepub.com by 2.235.186.236 on 11/16/20
For personal use only.

THE EFFECT OF AMINES AND PHENOLS ON THE GAS-PHASE OXIDATION OF *n*-BUTANE AND ISOBUTANE*

K. U. Ingold and I. E. Puddington

In recent years the antiknock efficiency of a large number of fuel additives has been measured. Among the more powerful fuel additives are amines both aliphatic and aromatic (1, 2, 3, 4), and aromatic compounds in general (2, 5).

From an examination of the high and low temperature oxidation of diisopropyl ether, Chamberlain and Walsh (6, 7) concluded that inhibition of this reaction by aromatic compounds and, therefore, the antiknock ability of aromatic compounds, was due primarily to the electronic properties of the ring, nitrogen, and other side groups merely modifying the properties of the ring. However, it has since been shown by using deuterated amine antiknocks that, in the internal combustion engine, aromatic amines owe their antiknock efficiency to their ability to destroy chain-propagating radicals ($X^{\cdot}$) by the reaction (3)

$$X^{\cdot} + ArNHR \rightarrow XH + Ar\dot{N}R \qquad [1]$$

where $Ar\dot{N}R$ is a less reactive radical than $X^{\cdot}$. The effect of deuterated antiknocks on the low temperature oxidation of hydrocarbons has, however, not been previously studied.

The present work involved a brief investigation of the effect of aniline and aniline N—D_2 on the low temperature oxidation of two simple paraffins, viz. *n*-butane and isobutane. The effect of a few other amines and phenols was also examined with a view to comparing inhibitor efficiencies in gas- and liquid-phase (8) hydrocarbon oxidations.

The oxidation was studied in a conventional apparatus, except that the progress of the reaction was measured by means of a diaphragm manometer to avoid contamination of the reaction vessel with mercury. The butane (70 mm) and oxygen (280 mm) were premixed before being admitted to the cylindrical Pyrex reaction vessel (15 cm long, 4 cm diameter). The oxidation of *n*-butane was studied at 275° C and isobutane in the range 275°–310° C. The pressure–time curves for both butanes are S-shaped, and although the

**Issued as N.R.C. No. 5266.*
