# Supplementary material for: 5-Hydroxytryptophan (5-HTP): Natural Occurrence, Analysis, Biosynthesis, Biotechnology, Physiology and Toxicology
Source: Int J Mol Sci. 2020 Dec 26;22(1):181. doi: 10.3390/ijms22010181 (PMC7796270; doi:10.3390/ijms22010181)
Supplement: Supplementary file 1 [file ijms-22-00181-s001.zip › 5-HTP.Data/PDF/2622234397/Lin-2014-Engineering-bacterial-phenylalanine.pdf]

Downloaded via UNIV DEGLI STUDI DI TORINO on November 17, 2020 at 16:54:52 (UTC).
See https://pubs.acs.org/sharingguidelines for options on how to legitimately share published articles.

ACS Synthetic Biology
Research Article
pubs.acs.org/synthbio

# Engineering Bacterial Phenylalanine 4-Hydroxylase for Microbial Synthesis of Human Neurotransmitter Precursor 5-Hydroxytryptophan

Yuheng Lin,[†] Xinxiao Sun,[‡] Qipeng Yuan,[‡] and Yajun Yan*[,§]

[†]College of Engineering, University of Georgia, Athens, Georgia 30602, United States
[‡]State Key Laboratory of Chemical Resource Engineering, Beijing University of Chemical Technology, Beijing 100029, China
[§]BioChemical Engineering Program, College of Engineering, University of Georgia, Athens, Georgia 30602, United States

*S* *Supporting Information*

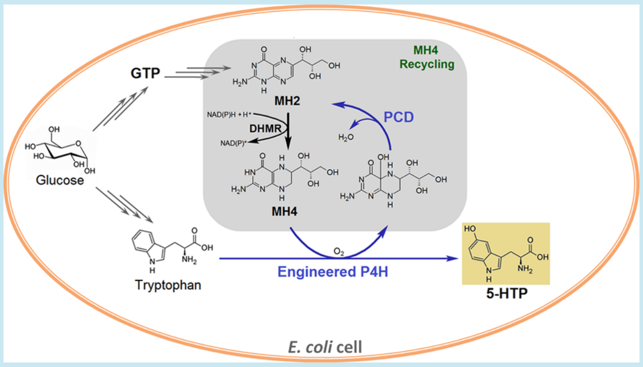


**ABSTRACT:** 5-Hydroxytryptophan (5-HTP) is a drug that is clinically effective against depression, insomnia, obesity, chronic headaches, etc. It is only commercially produced by the extraction from the seeds of *Griffonia simplicifolia* because of a lack of synthetic methods. Here, we report the efficient microbial production of 5-HTP via combinatorial protein and metabolic engineering approaches. First, we reconstituted and screened prokaryotic phenylalanine 4-hydroxylase activity in *Escherichia coli*. Then, sequence- and structure-based protein engineering dramatically shifted its substrate preference, allowing for efficient conversion of tryptophan to 5-HTP. Importantly, *E. coli* endogenous tetrahydromonapterin (MH4) could be utilized as the coenzyme, when a foreign MH4 recycling mechanism was introduced. Whole-cell bioconversion allowed the high-level production of 5-HTP (1.1−1.2 g/L) from tryptophan in shake flasks. On this basis, metabolic engineering efforts were further made to achieve the *de novo* 5-HTP biosynthesis from glucose. This work not only holds great scale-up potential but also demonstrates a strategy for expanding the native metabolism of microorganisms.

**KEYWORDS:** *antidepressant, 5-hydroxytryptophan, phenylalanine 4-hydroxylase, protein engineering, metabolic engineering, microbial production*

The World Health Organization (WHO) has reported that depression is a common mental disorder affecting more than 350 million people globally. It results in ∼1 million suicides per year. Unfortunately, more than 50% of sufferers around the world (>90% in some regions) have never received medical treatment.[1] Alterations in serotonin (5-hydroxytryptamine) metabolism were thought to be an important physiological factor for depression.[2] Dysfunction of the serotonergic mechanism in the central nervous system (CNS) has been implicated in the etiology of depression.[3] However, the supply of serotonin via oral administration is not clinically effective against depression because it cannot pass through the brain−blood barrier. Unlike the conventional antidepressants (e.g., selective serotonin re-uptake inhibitors) acting on minimizing serotonin loss, 5-hydroxytryptophan (5-HTP) functions as the direct precursor to increase serotonin supply. Orally administered 5-HTP can easily pass through the blood−brain barrier without requiring transport molecules. Then it can be efficiently converted into serotonin in the CNS by endogenous decarboxylase.[3] Detailed clinical trials have demonstrated its efficacy in alleviating depression symptoms. Meanwhile, the therapeutic administration of 5-HTP has been shown to be effective in treating insomnia, fibromyalgia, obesity, cerebellar ataxia, and chronic headaches.[4] Importantly, relatively few adverse effects are associated with its use in treatment.[2] In most European countries, 5-HTP is a commonly prescribed drug for multiple treatment purposes, while in North America, it is sold as an "over-the-counter" dietary supplement.

Because of the difficulty involved in regioselective hydroxylation of tryptophan via chemical approaches, the commercial production of 5-HTP relies on its isolation from the seeds of *Griffonia simplicifolia*, a woody climbing shrub grown in West and Central Africa.[3,4] The season- and region-dependent supply of the raw materials has been largely limiting its cost-effective production and broad clinical applications. In the past few decades, the development of metabolic engineering and protein engineering in combination with fundamental genetics, biochemistry, and bioinformatics provides new strategies for synthesizing natural and non-natural molecules using microbial systems. On the basis of the accumulated knowledge about natural biosynthetic mechanisms of target products, especially

**Received:** May 16, 2014
**Published:** June 17, 2014

 © 2014 American Chemical Society 

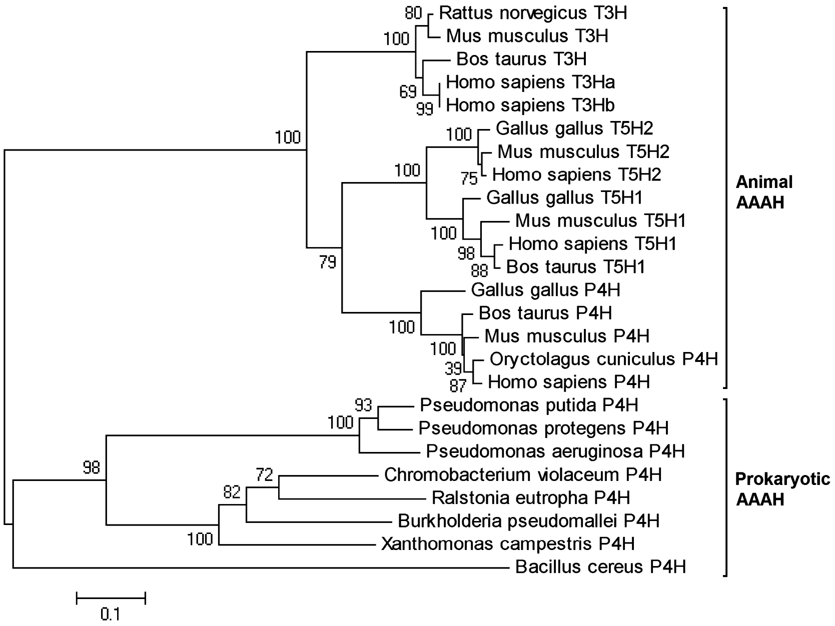


**Figure 1.** Phylogenetic relationship between prokaryotic P4Hs and animal AAAHs. The protein sequence alignment was performed using ClustalX version 2.1. The phylogenetic tree was constructed with MEGA version 5.02 by using the neighbor-joining method. The bootstrapping method was used for the phylogeny test (1000 replications). The numbers associated with the branches refer to the bootstrap values representing the substitution frequencies per amino acid residue.

the genetic and biochemical information about the involved enzymes, heterologous enzymatic reactions can be reconstituted, modified, and optimized in genetically superior microbial hosts to achieve the efficient production of pharmaceutically important compounds that are scarce in nature.[5−11]

5-HTP is natively produced in humans and animals from L-tryptophan by the action of tryptophan 5-hydroxylase (T5H) and then converted to the neurotransmitter serotonin under normal physiological conditions.[4] T5Hs belong to the class of pterin-dependent aromatic amino acid hydroxylases (AAAHs) that also include two other subgroups: phenylalanine 4-hydroxylases (P4Hs) and tyrosine 3-hydroxylases (T3Hs). AAAHs were broadly identified and extensively studied in animals because of their close relationship with human diseases such as phenylkentonuria, Parkinson's disease, and neuropsychiatric disorders.[12] These enzymes consist of three domains that are the N-terminal regulatory domain, the central catalytic domain, and the C-terminal domain involved in tetramer formation and usually utilize tetrahydrobiopterin (BH4) as the coenzyme (or cosubstrate).[13] Animal T5Hs proved to be unstable and hard to express functionally in a microbial host.[14,15] A very recent patent reported the use of truncated T5H1 from *Oryctolagus cuniculus*, which produced up to 0.9 mM (equivalent to 198 mg/L) 5-HTP from tryptophan in *Escherichia coli*. To supply the pterin coenzyme, the animal BH4 biosynthetic pathway coupled with a regeneration system including a total of five enzymes was required for co-expression in *E. coli*.[16] However, the production efficiency is still not satisfying for scale-up production.

A few AAAHs were also found in bacteria such as *Pseudomonas* and *Chromobacterium* species.[17,18] So far, all of them were identified as P4Hs with little activity for tryptophan hydroxylation, but such activity was reported to be improved *in vitro* when mutations were introduced into the P4H from *Chromobacterium violaceum*.[19] Prokaryotic P4Hs consist of only one domain corresponding to the catalytic domain of animal AAAHs.[13] Recent experimental evidence indicated that bacterial P4Hs may utilize tetrahydromonapterin (MH4) instead of BH4 as the native pterin coenzyme,[20] because BH4 does not naturally occur in most bacteria. Interestingly, MH4 is the major form of pterin in *E. coli*, although its function is still unknown. In this work, we report the reconstitution of bacterial P4H activity in *E. coli* through utilization and recycling of its endogenous MH4. Combined bioprospecting and protein engineering approaches allowed the development of the P4H mutants that are highly active in converting tryptophan to 5-HTP, which allowed the establishment of an efficient 5-HTP production platform via further metabolic engineering efforts. This *de novo* process does not require supplementation of expensive pterin cofactors or precursors but utilizes only renewable simple carbon sources, which holds great potential for scale-up production of 5-HTP in microorganisms.

## ■ RESULTS AND DISCUSSION

**Phylogenetic Analysis of AAAHs.** Compared with animal AAAHs that include three subgroups, their prokaryotic counterparts were all identified or annotated as P4H only. Previous biochemical and structural studies revealed that in addition to the central catalytic domain, animal AAAHs usually consist of two additional domains that are the N-terminal regulatory domain and the C-terminal domain involved in tetramer formation, while prokaryotic AAAHs (e.g., P4H from *C. violaceum*) are monomers with only one single domain that shares moderate sequence similarity (∼30%) with the catalytic domains of animal AAAHs.[21] To explore the evolutionary relationship among AAAHs, 25 amino acid sequences from both prokaryotes and animals were randomly selected and a phylogenetic tree was constructed using MEGA version 5.02 based on the neighbor-joining method (Figure 1).[22] The tree reflects a considerable evolutionary separation between prokaryotic and animal AAAHs. The three subfamilies (P4Hs, T5Hs, and T3Hs) of animal AAAHs are distinctly separated, as

well, among which P4Hs show closer a phylogenetic relationship with T5Hs than with T3Hs. These results are consistent with a previous phylogenetic study of AAAHs.[23]

Considering the phylogenetic evidence in combination with the development of functional diversity, we inferred that the animal AAAHs evolved from prokaryotic P4Hs through duplication and divergence. Therefore, we hypothesized that even after a long-term evolution process, prokaryotic and animal P4Hs may still share some conserved amino acid residues that determine their substrate preference toward phenylalanine. Meanwhile, animal P4Hs and T5Hs share a high degree of sequence similarity, suggesting that the interchange of substrate preference from phenylalanine to tryptophan may only involve the substitution of a small number of residues. On the basis of these hypotheses, we speculated that by performing a comprehensive alignment analysis of the sequences of animal AAAHs and prokaryotic P4Hs, we may be able to identify the substrate-determining residues from the latter group and artificially force them to evolve into T5Hs.

**Bioprospection and Reconstitution of Prokaryotic P4Hs in *E. coli*.** Before exploring the substrate-determining amino acid residues, we picked five P4Hs from different microorganisms (*Pseudomonas aeruginosa*, *Pseudomonas putida*, *Pseudomonas fluorescens*, *Ralstonia eutropha*, and *Xanthomonas campestris*) to verify and compare their activities and substrate preferences, because most of the prokaryotic P4Hs are still putative enzymes without experimental confirmation of their function. The P4H from *P. aeruginosa* (PaP4H) was previously identified *in vitro*, and its crystal structure has been determined.[24] Some genetic and biochemical evidence suggested that PaP4H utilizes MH4 instead of BH4 as the native pterin coenzyme.[20] Thus, we first selected it as a prototype to establish its *in vivo* activity in *E. coli*, because MH4 is the major pterin produced by *E. coli* (Figure 2). To achieve the expression of PaP4H, its *phhA* gene was amplified from the genomic DNA of *P. aeruginosa* and cloned into a high-copy number plasmid under the control of an isopropyl β-D-1-thiogalactopyranoside (IPTG)-inducible promoter $P_L$*lacO1*. The resulting expression vector pZE-PaphhA was introduced into *E. coli* strain BW25113Δ*tnaA* (abbreviated as BWΔ*tnaA*). Because tryptophanase encoded by *tnaA* was reported to catalyze the degradation of tryptophan and 5-HTP,[25] the gene was knocked out from all the strains used in this study. We observed that the cell growth of BWΔ*tnaA* carrying pZE-PaphhA was significantly retarded. Its $OD_{600}$ values reached only 0.8−1.0 after cultivation for 8 h, dramatically lower than those of the control strain (BWΔ*tnaA* carrying an empty vector) with $OD_{600}$ values at 5.5−6.0. A similar effect was also observed in a previous study.[26] When the cells were incubated with phenylalanine (500 mg/L), almost no hydroxylated product (tyrosine) was detected. Indeed, *P. aeruginosa* possesses a pterin 4a-carbinolamine dehydratase (PCD, encoded by *phhB*) responsible for the regeneration of dihydromonapterin (MH2), which can be further reduced to MH4, but *E. coli* does not have such a mechanism natively. To establish an artificial MH4 recycling system (Figure 2), *phhB* from *P. aeruginosa* and *folM* from *E. coli* [encoding dihydromonapterin reductase (DHMR)] were co-expressed along with the *phhA* using vector pZE-PaABM. Interestingly, the *E. coli* strain harboring this vector dramatically improved cell viability, which was comparable to that of the control strain. Its $OD_{600}$ values reached 4.5−5.5 after cultivation for 8 h. When these cells were collected and incubated with phenylalanine, a large amount of tyrosine was produced at a rate of 83.50 μM $h^{-1}$ $OD_{600}{}^{-1}$, as was shown in the *in vivo* assays (Table 1).

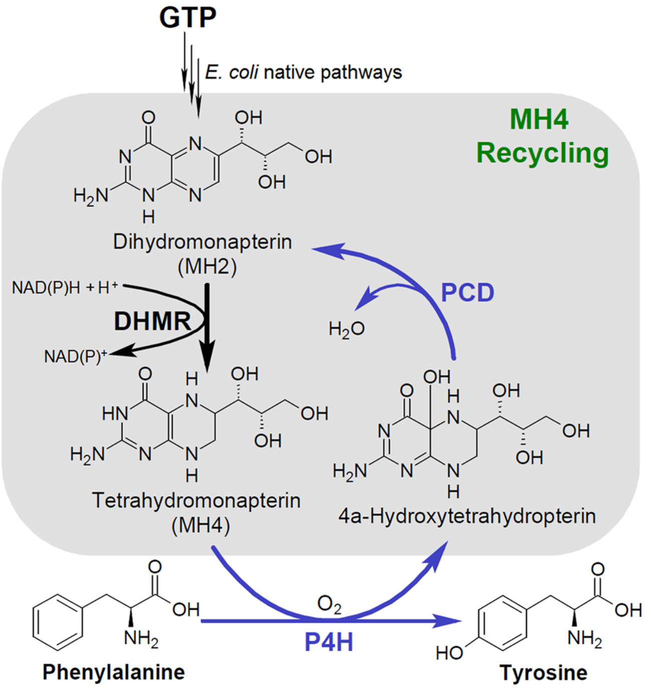


**Figure 2.** Reconstitution of prokaryotic P4H activity in *E. coli.* The black- and blue-colored arrows indicate the *E. coli* native pathways and heterologous reactions, respectively. Bold arrows refer to the overexpressed steps. The introduced MH4 recycling system is indicated by the gray-colored box. Abbreviations: GTP, guanosine 5′-triphosphate; PCD, pterin-4α-carbinolamine dehydratase; DHMR, dihydromonapterin reductase; P4H, phenylalanine 4-hydroxylase.

**Table 1. *In Vivo* Activities of P4Hs from Different Microorganisms**

| | *in vivo* activity[a] (μM $h^{-1}$ $OD_{600}{}^{-1}$) | | |
|---|---|---|---|
| source of P4H | phenylalanine | tryptophan | preference (Phe:Trp) |
| *P. aeruginosa* | 83.50 ± 16.00 | 0.19 ± 0.02 | 439.5 |
| *P. putida* | 76.32 ± 10.02 | 0.12 ± 0.03 | 636.0 |
| *P. fluorescens* | 82.47 ± 12.05 | 0.20 ± 0.05 | 412.4 |
| *R. eutropha H16* | 73.33 ± 4.63 | 1.22 ± 0.04 | 60.1 |
| *X. campestris* | 97.40 ± 4.42 | 2.91 ± 0.21 | 33.5 |

[a]All data are reported as means ± the standard deviation from three independent experiments.

These results indicated that introduction of the MH4 recycling system not only restored the cell growth but also allowed the *E. coli* strain to convert phenylalanine to tyrosine. Moreover, this strain was also capable of converting tryptophan to 5-HTP (Table 1), although the production rate (0.19 μM $h^{-1}$ $OD_{600}{}^{-1}$) was much lower, only equivalent to 0.23% of that toward phenylalanine.

On this basis, another four P4Hs were also tested by replacing the PaP4H gene on pZE-PaABM with their respective genes. As shown in Table 1, all the identified P4Hs showed high activity and a strong substrate preference for phenylalanine in *E. coli*. Among them, the P4H from *X. campestris* (XcP4H) exhibited the highest activity toward both phenylalanine and tryptophan. The three from *Pseudomonas* species showed the most similar catalytic properties, which is consistent with their

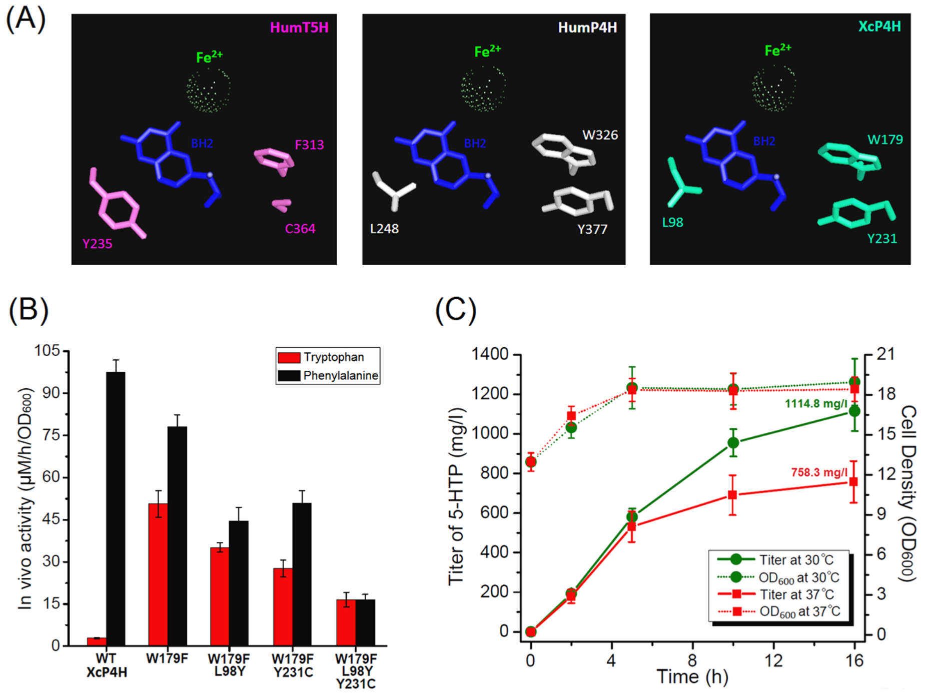


**Figure 3.** Modification of XcP4H via protein engineering. (A) Comparative illustration of the positions of the three critical residues (L98, W179, and Y231) in the structures of XcP4H, the P4H from human (HumP4H), and the T5H1 from human (HumT5H). (B) *In vivo* activities of wild-type XcP4H and its mutants. Red- and black-colored bars indicate the activities toward tryptophan and phenylalanine, respectively. (C) Whole-cell bioconversion of tryptophan into 5-HTP using XcP4H mutant W179F. Solid and dotted lines indicate the time courses of 5-HTP production and cell density, respectively. Green- and red-colored lines indicate the profiles at 30 and 37 °C, respectively. All data are reported as means ± the standard deviation from three independent experiments. Error bars are defined as the standard deviation.

**Table 2. *In Vivo* Activities and Substrate Preferences of XcP4H Mutants**

| | substrate | | | | |
|---|---|---|---|---|---|
| | phenylalanine | | tryptophan | | |
| | *in vivo* activity[a] ($\mu$M $h^{-1}$ $OD_{600}^{-1}$) | RA[b] (%) | *in vivo* activity[a] ($\mu$M $h^{-1}$ $OD_{600}^{-1}$) | RA[b] (%) | preference (Phe:Trp) |
| WT | 97.40 ± 4.42 | 100 | 2.91 ± 0.21 | 100 | 33.5 |
| W179F | 78.05 ± 4.34 | 80 | 50.60 ± 4.72 | 1739 | 1.5 |
| W179F/L98Y | 44.49 ± 4.95 | 46 | 35.13 ± 1.67 | 1207 | 1.3 |
| W179F/Y231C | 50.92 ± 4.36 | 52 | 27.71 ± 2.99 | 952 | 1.8 |
| W179F/L98Y/Y231C | 16.56 ± 1.86 | 17 | 16.58 ± 2.59 | 570 | 1.0 |

[a]All data are reported as means ± the standard deviation from three independent experiments. [b]RA, relative activity, setting the RA of WT XcP4H to 100%.

close phylogenetic relationship. Therefore, we confirmed that all the tested P4Hs can function well by utilizing the *E. coli* endogenous pterin coenzyme MH4 in the presence of a recycling system.

**Modification of the XcP4H Substrate Preference through Protein Engineering.** XcP4H was selected for protein engineering because of its superior catalytic potential. To investigate the substrate-determining amino acid residues, its sequence was aligned with animal P4Hs and T5Hs. Comparison of the sequences of only animal P4Hs and T5Hs led to the identification of a number of residues that are conserved within each group but varied between groups. However, when these residues were aligned with the XcP4H sequence, only six of them were found to be conserved in P4Hs and probably critical to the substrate selectivity, which are Q85, L98, W179, L223, Y231, and L282 (Figure S1 of the Supporting Information). To further investigate their locations in the enzyme structure, a homology model was built using the crystal structure of the P4H from *C. violaceum* [Protein Data Bank (PDB) entry 3TK2] as a template. The conserved residues were well-aligned with those in the crystal structures of human P4H (PDB entry 1MMK) and T5H1 (PDB entry 3HF6), indicating the reliability of the model. In this structure, W179 is located inside the catalytic pocket just at the predicted phenylalanine binding site, while L98 and Y231 are near the entrance to the pocket, which are closer to the coenzyme MH4 binding site (Figure 3A). However, Q85, L223, and L282 are not located near the catalytic pocket, suggesting these residues are less relevant to the enzyme's substrate selection. Therefore, we selected W179, L98, and Y231 as the targets for further mutation analysis. We hypothesized that if these residues were replaced with their respective residues in T5Hs that are F, Y, and C, respectively, the mutants might exhibit a stronger preference for tryptophan. As a result, the W179F mutant of XcP4H exhibited a 17.4-fold increase in tryptophan hydroxylation activity compared with that of the wild-type (WT) enzyme; meanwhile, its activity toward phenylalanine decreased by ~20%. The substrate preference for phenylalanine over

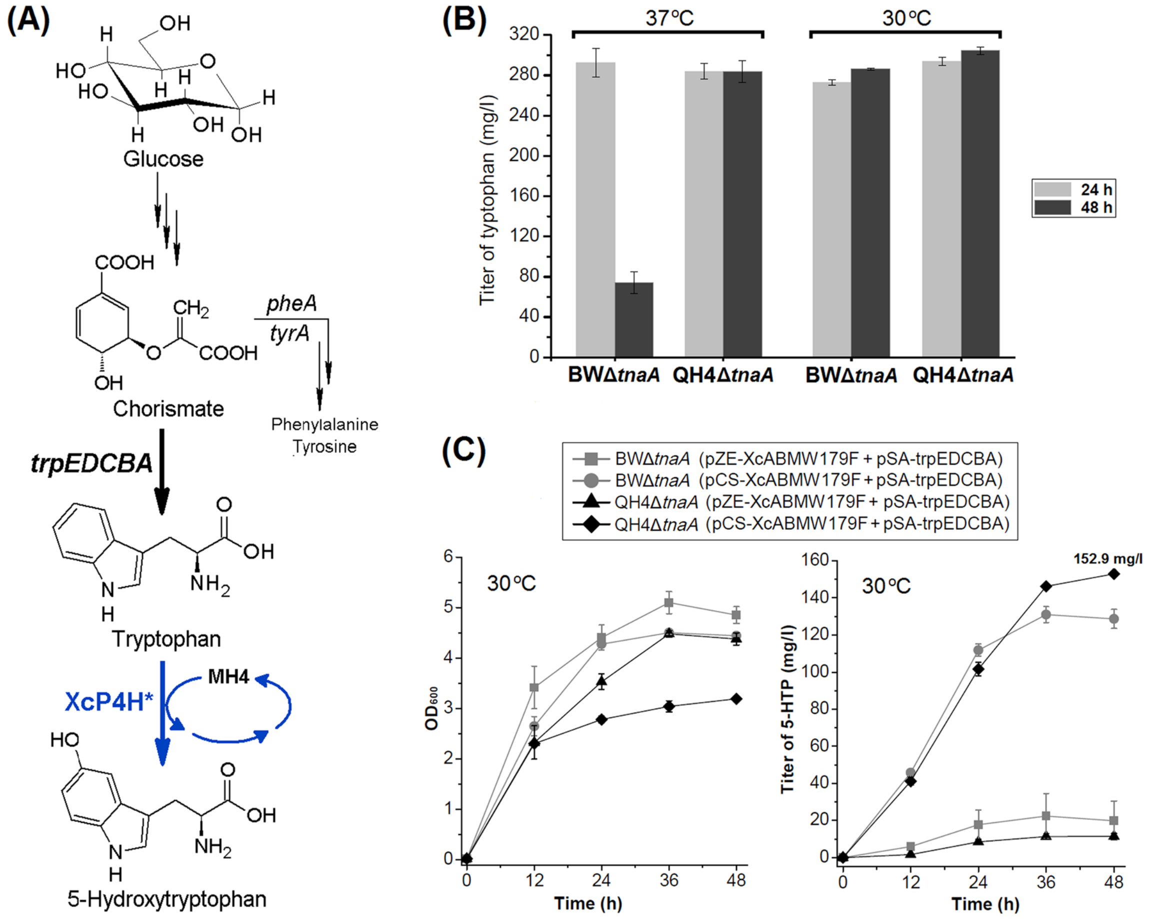


**Figure 4.** *De novo* production of 5-HTP from glucose. (A) Schematic presentation of the complete 5-HTP biosynthetic pathway. The black- and blue-colored arrows indicate the *E. coli* native pathways and heterologous reactions, respectively. (B) Production of tryptophan from glucose at 30 and 37 °C. Data are reported as means ± the standard deviation from two independent experiments. (C) Profiles of cell growth and 5-HTP production from glucose of two host strains, BWΔ*tnaA* (gray-colored) and QH4Δ*tnaA* (black-colored). All data are reported as means ± the standard deviation from three independent experiments. Error bars are defined as the standard deviation.

tryptophan was shifted from 33.5 to 1.5 (Table 2). When the L98Y or Y231C mutation was combined with the W179F mutation, the substrate preference further shifted toward tryptophan, although their activities toward tryptophan were not as high as that of the W179F mutation alone. The triple mutant showed almost the same preference for the two substrates (Figure 3B). As we mentioned, L98 and Y231 are closer to the MH4 binding site, suggesting that these two residues might not contribute to aromatic amino acid substrate selection.

To further explore the potential of XcP4H mutant W179F for whole-cell biocatalysis, feeding experiments were conducted by incubating precultured *E. coli* cells harboring pZE-XcABMW179F (initial $OD_{600}$ of 12−13) with 2.0 g/L tryptophan. As shown in Figure 3C, the initial conversion rates were similar at 30 and 37 °C, although the cells grew slightly faster at 37 °C. However, the production efficiency at 30 °C became obviously higher after 5 h. By the end of 16 h, the cultures at 30 and 37 °C accumulated 1114.8 and 758.3 mg of 5-HTP per liter at the expense of 1503.2 and 1417.1 mg/L tryptophan, respectively. Meanwhile, we observed that the color of the cultures gradually turned dark after 5 h, especially at 37 °C, probably because of the oxidation of 5-HTP and tryptophan under aerobic conditions.

***De Novo* Microbial Synthesis of 5-HTP via Metabolic Engineering.** After achieving the efficient bioconversion of tryptophan to 5-HTP, we proceeded with the construction of a 5-HTP-producing strain that allows the utilization of endogenous tryptophan generated from simple carbon sources (Figure 4A). Our first attempt was focused on the construction of a tryptophan overproducer. In *E. coli*, tryptophan biosynthesis is branched from the shikimate pathway at chorismate by the action of the *trp* regulon (Figure 4A) and negatively regulated by the tryptophan transcriptional repressor (TrpR) in response to intracellular tryptophan levels. To circumvent the intrinsic regulation at the transcription level, the complete *trp* operon, including *trpEDCBA*, was cloned into a low-copy number plasmid under the control of an IPTG-inducible promoter. Meanwhile, to eliminate the feedback inhibition effect, an S40F mutation was incorporated into TrpE according to a previous study,[27] resulting in plasmid pSA-TrpEDCBA. When the plasmid was introduced into *E. coli* BWΔ*tnaA*, the resulting strain produced 292.2 mg/L tryptophan at 37 °C after a 24 h cultivation; however, the titers dramatically decreased after 48 h (74.4 mg/L) probably because of oxidative degradation.[28] This problem was solved when the growth temperature was changed to 30 °C (Figure 4B). In addition to BWΔ*tnaA*, we attempted to use QH4Δ*tnaA* as the host for boosting carbon flux through the shikimate pathway, because

QH4 is a derivative of the well-developed phenylalanine overproducer ATCC31884 with *pheLA* and *tyrA* disrupted and has been successfully engineered for the enhanced production of caffeic acid, salicylic acid, and muconic acid in our previous studies.[29,30] However, in this study, QH4Δ*tnaA* harboring pSA-TrpEDCBA did not significantly improve the production of tryptophan but showed slightly improved titers at 30 °C compared with that of the BWΔ*tnaA* host. By the end of 48 h, up to 304.4 mg/L tryptophan was produced (Figure 4B). The control strain QH4Δ*tnaA* without the overexpression of the *trp* operon did not accumulate tryptophan at either temperature.

As the tryptophan production and the bioconversion of tryptophan to 5-HTP were achieved and 30 °C worked better for both cases, our further efforts were directed at establishing the *de novo* biosynthesis of 5-HTP at this temperature by integrating the two modules. When pZE-XcABMW179F was cotransferred together with pSA-TrpEDCBA into *E. coli* BWΔ*tnaA* and QH4Δ*tnaA*, the generated strains produced only 19.9 and 11.5 mg/L 5-HTP, respectively, without tryptophan accumulating in the cultures. Apparently, the introduction of the 5-hydroxylation reaction using a high-copy number plasmid exerted a negative influence on the flow of carbon through tryptophan compared with those of their parent strains. We speculated that the excessive expression of the XcP4H mutant with the MH4 recycling system might have resulted in metabolic imbalance and disturbed carbon flux toward tryptophan. To test this hypothesis, we cloned the coding sequences of XcP4H mutant W179F, PCD, and DHMR into a medium-copy number plasmid instead of the high-copy number one, yielding plasmid pCS-XcABMW179F. Interestingly, we observed dramatic improvement in 5-HTP production for both BWΔ*tnaA* and QH4Δ*tnaA* harboring pCS-XcABMW179F; by the end of 48 h, the two strains produced 128.6 and 152.9 mg/L 5-HTP, respectively (Figure 4C), at the expense of 8.5 and 9.7 g/L glucose consumption, respectively. Meanwhile, we detected the accumulation of tryptophan at concentrations of 166.3 and 339.7 mg/L for the two strains, indicating that the carbon flux toward tryptophan was fully recovered. Other byproducts were also detected as depicted in Table S1 of the Supporting Information. The 5-HTP-producing strains followed growth-dependent production patterns (Figure 4C).

**Discussion.** The expense of medication has become a major burden of families around the world. High price indeed deprives the low-income population of access to the use of some drugs that are hard to obtain and expensive. The causes of this issue are either the low efficiency in isolating these pharmaceuticals from natural sources or the high cost of their chemical synthesis. Microbial biosynthesis and biocatalysis provide a facile and eco-friendly way to produce pharmaceutically valuable compounds. The development of metabolic engineering and synthetic biology tools allows the tailored assembly of heterologous and artificial pathways in desirable host strains for the biosynthesis of target products.[31]

A lack of suitable enzymes is one of the most frequently encountered problems in pathway engineering in microbes. Functional expression of eukaryotic enzymes is often problematic because of their low solubility, low stability, and/or requirements for post-translational modification. For example, the tryptophan 5-hydroxylation reaction has been understood for a long time, and many T5Hs from human and animals have been identified and characterized. However, animal AAAHs were hard to express in *E. coli* in a soluble and stable form.[14,32] In addition, their activities are usually regulated by phosphorylation as well as their products.[33] Although the use of truncated or fusion proteins can help yield soluble and active enzymes,[32] the catalytic efficiency seems to remain low in the production of 5-HTP in *E. coli* using truncated animal T5Hs.[16] In recent years, protein engineering has become a potent tool for enzyme modification for obtaining the desired catalytic properties.[34] In our study, a highly active P4H that utilizes MH4 was successfully engineered to catalyze the tryptophan 5-hydroxylation reaction. As we were writing this article, another study also reported the 5-HTP production in *E. coli* with a mutant P4H from *C. violaceum*.[35] However, its function had to completely rely on the supplementation of exogenous pterin coenzyme 6,7-dimethyl-5,6,7,8-tetrahydropterine hydrochloride,[35] which is disadvantageous in terms of economic viability as we discuss below.

Self-supply or regeneration of cofactors (including coenzymes and cosubstrates) is one of the greatest advantages for whole-cell biosynthesis and biocatalysis. In the *E. coli* host, many such molecules can be natively generated along with cell growth, such as FMN/$FMNH_2$, FAD/$FADH_2$, $NAD(P)^+$/NAD(P)H, coenzyme A, acetyl-CoA, malonyl-CoA, MH4, etc. Although it is more convenient and economical to utilize these endogenous cofactors, sometimes heterologous enzymes require the cofactor(s) not produced by the host strain. To solve such a problem, one approach is to supplement the culture medium with exogenous cofactors, but it should be noted that most of the cofactors such as tetrahydropterine are so expensive that the supplementation of them is not economically viable for commercial production. Another approach is to introduce the cofactor biosynthetic and/or regeneration mechanism(s) into the host strain. As in the study using animal T5H to produce 5-HTP, a BH4 biosynthetic pathway starting from GTP was introduced into *E. coli*.[16] Meanwhile, a BH4 regeneration system was necessary to achieve continuous production. Recently, an interesting study reported that mouse T3H can also utilize *E. coli* MH4 in the presence of a BH4 regeneration system, although the efficiency proved to be low.[36] In this work, with minimal modifications of the host strains' metabolism, the prokaryotic P4Hs and the mutants were able to utilize and recycle *E. coli* endogenous MH4 and NAD(P)H (Figure 2).

**Conclusion.** This work simultaneously solved two problems in the biological production of 5-HTP, which are related to enzyme compatibility and cofactor self-supply. To the best of our knowledge, the titer of 5-HTP (1.1−1.2 g/L) generated from tryptophan in this work is significantly higher than those in previous studies, showing great scale-up potential. Moreover, this work also demonstrates the *de novo* production of 5-HTP without needing to supplement precursors and coenzymes. Because the high-level production of tryptophan (up to 48.7 g/L) has already been achieved in *E. coli*,[37] introduction and optimization of the 5-hydroxylation reaction into tryptophan overproducers is expected to result in efficient and low-cost production of 5-HTP.

## ■ MATERIALS AND METHODS

**Experimental Materials.** *E. coli* XL1-Blue was employed as the host strain for cloning and plasmid propagation; *E. coli* BW25113Δ*tnaA* was used as the host strain for *in vivo* enzyme assays, feeding experiments, and *de novo* production of tryptophan and 5-HTP. QH4 was previously constructed with the disruption of *pheLA* and *tyrA* from a phenylalanine

producer, *E. coli* ATCC31884.[29] Luria-Bertani (LB) medium containing 10 g/L tryptone, 5 g/L yeast extract, and 10 g/L NaCl was used for cell cultivation and enzyme expression. M9 minimal medium containing 5 g/L glycerol, 6 g/L $Na_2HPO_4$, 0.5 g/L NaCl, 3 g/L $KH_2PO_4$, 1 g/L $NH_4Cl$, 246.5 mg/L $MgSO_4·7H_2O$, 14.7 mg/L $CaCl_2$, and 27.8 mg/L $FeSO_4·7H_2O$ was used for *in vivo* assays of P4Hs. Modified M9 (M9Y) medium was used for *de novo* production of tryptophan and 5-HTP. M9Y medium contains 10 g/L glucose, 6 g/L $Na_2HPO_4$, 0.5 g/L NaCl, 3 g/L $KH_2PO_4$, 1 g/L $NH_4Cl$, 246.5 mg/L $MgSO_4·7H_2O$, 14.7 mg/L $CaCl_2·2H_2O$, 27.8 mg/L $FeSO_4·7H_2O$, 2 g/L yeast extract, and 2 g/L sodium citrate dihydrate. When necessary, the medium was supplemented with kanamycin, ampicillin, and chloramphenicol at final concentrations of 50, 100, and 34 mg/L, respectively. pZE12-luc, pCS27, and pSA74 are high-, medium-, and low-copy number plasmids,[30] respectively, which were used for expressing enzymes in *E. coli*. Details of the strains and plasmids used in this study are listed in Table S2 of the Supporting Information.

**DNA Manipulation.** *E. coli* strain BW25113 Δ*tnaA::kan* (JW3686-7) was purchased from the Coli Genetic Stock Center. The kanamycin resistant marker was deleted according to the reported protocol.[38] Deletion of the *tnaA* gene from QH4 was performed using the reported Red disruption method.[38] Plasmid pZE-PaphhA was constructed by inserting the amplified *phhA* gene from *P. aeruginosa* into pZE12-luc using restriction sites Acc65I and XbaI. pZE-PaABM was constructed by inserting *phhA* and *phhB* from *P. aeruginosa* and *folM* from *E. coli* into pZE12-luc via multipiece ligation using Acc65I/NdeI, NdeI/HindIII, and HindIII/XbaI. pZE-PpABM, pZE-PfABM, pZE-ReABM, and pZE-XcABM were constructed using the same approach with the respective *phhA* genes in place of the *phhA* gene from *P. aeruginosa*. pSA-trpEDCBA was constructed by inserting the DNA fragment of *trpEDCBA* from *E. coli* into pSA74 using Acc65I and BamHI. Site-directed mutagenesis was conducted by overlap polymerase chain reaction. Plasmids pZE-XcABMW179F, pZE-XcABM2Ma, pZE-XcABM2Mb, and pZE-XcABM3M were constructed by replacing the wild-type *phhA* gene from *X. campestris* with the respective mutant genes (Table S2 of the Supporting Information).

**Construction of the Phylogenetic Tree and Homology Modeling.** The AAAH sequences were randomly selected from GenBank using "phenylalanine 4-hydroxylase", "tyrosine 3-hydroxylase", and "tryptophan 5-hydroxylase" as the searching keywords. The alignment of the AAAH amino acid sequences was conducted using ClustalX version 2.1. The phylogenetic tree was constructed by Molecular Evolutionary Genetics Analysis (MEGA) version 5.02 using the neighbor-joining method.[22] The bootstrapping test was performed to evaluate the reliability (1000 replicates). All other used parameters were the default of the software. The homology model of XcP4H was built with the SWISS-MODEL online server by using the crystal structure of the P4H from *C. violaceum* (PDB entry 3TK2) as a template.

***In Vivo* Assays of Wild-Type and Mutant P4Hs.** *E. coli* BW25113Δ*tnaA* carrying pZE-PaABM was inoculated into 50 mL of LB liquid medium containing 0.5 mM IPTG and 100 μg/mL ampicillin and grown aerobically at 37 °C for ~8 h until the $OD_{600}$ reached 4.5−5.5. Then the cells were harvested and resuspended in M9 minimal medium ($OD_{600}$ = 4.5−5.5). After adaptation for 20 min, phenylalanine or tryptophan was added to the cell suspension to a final concentration of 500 mg/L. At the same time, 1 mM ascorbic acid was added to prevent product oxidation. The flasks were incubated with shaking (300 rpm) at 37 °C for 1 h. Subsequently, samples were taken by removing cell pellets, and the products (tyrosine and tryptophan) were quantitatively measured via high-performance liquid chromatography (HPLC). The same method was used to measure the *in vivo* activities of other P4Hs and XcP4H mutants. The *in vivo* activities of P4Hs were expressed in units of micromolar per minute per $OD_{600}$.

**Bioconversion of Tryptophan to 5-HTP.** *E. coli* strain BW25113Δ*tnaA* was transformed with plasmid pZE-XcABMW179F. Single colonies were inoculated into 50 mL of LB medium containing 0.5 mM IPTG and grown aerobically at 37 °C for ~8 h until the $OD_{600}$ reached ~5.0. Then cells were harvested, resuspended in 20 mL of M9Y medium (at $OD_{600}$ = 12−13) containing 2 g/L tryptophan, and left to grow at 30 and 37 °C. Samples were taken at 2, 5, 10, and 16 h. The concentrations of produced 5-HTP were analyzed by HPLC.

***De Novo* Production of Tryptophan and 5-HTP.** Overnight LB cultures of the producing strains were inoculated at 2% into the M9Y medium containing appropriate antibiotics and cultivated at 30 and 37 °C while being shaken at 300 rpm. IPTG was added to the cultures to a final concentration of 0.5 mM at 0 h. Samples were taken every 12 h. The $OD_{600}$ values were measured, and the concentrations of the products, intermediates, and byproducts were analyzed by HPLC.

**HPLC Analysis.** L-Tyrosine (from Sigma-Aldrich), L-tryptophan (from Sigma-Aldrich), and 5-HTP (from Acros Organics) were used as standards. Both the standards and samples were quantified by HPLC (Dionex Ultimate 3000 installed with an Ultimate 3000 photodiode array detector and a reverse phase ZORBAX SB-C18 column). A gradient elution method described in our previous study[39] was used. Quantification of tryptophan, tyrosine, and 5-HTP was based on the peak areas at a specific wavelength (276 nm). Glucose, acetate, and pyruvate were quantified using a previously described method.[40]

## ■ ASSOCIATED CONTENT

### Supporting Information

This material is available free of charge via the Internet at http://pubs.acs.org.

## ■ AUTHOR INFORMATION

### Corresponding Author

*Address: 601B Driftmier Engineering Center, University of Georgia, Athens, GA 30602. Telephone: (706) 542-8293. Fax: (706) 542-8806. E-mail: yajunyan@uga.edu.

### Author Contributions

Y.Y. and Y.L. conceived this study. Y.L. and X.S. designed and performed the experiments. Y.Y. and Q.Y. supervised and directed the project. Y.L. analyzed the data and wrote the manuscript. Y.Y. revised the manuscript.

### Notes

The authors declare the following competing financial interest(s): A patent application about this technology has been filed by the University of Georgia.

## ■ ACKNOWLEDGMENTS

This work was supported by start-up funds from the College of Engineering of the University of Georgia. We also acknowledge

the support from the National Science Foundation of China (21176018).

## ■ REFERENCES

(1) World Health Organization (2012) World Health Organization Fact Sheet on Depression (http://www.who.int/mediacentre/factsheets/fs369/en/index.html).

(2) Byerley, W. F., Judd, L. L., Reimherr, F. W., and Grosser, B. I. (1987) 5-Hydroxytryptophan: A review of its antidepressant efficacy and adverse effects. *J. Clin. Psychopharmacol.* *7*, 127−137.

(3) Turner, E. H., Loftis, J. M., and Blackwell, A. D. (2006) Serotonin a la carte: Supplementation with the serotonin precursor 5-hydroxytryptophan. *Pharmacol. Ther.* *109*, 325−338.

(4) Birdsall, T. C. (1998) 5-Hydroxytryptophan: A clinically-effective serotonin precursor. *Alternative Medicine Review* *3*, 271−280.

(5) Ajikumar, P. K., Xiao, W. H., Tyo, K. E. J., Wang, Y., Simeon, F., Leonard, E., Mucha, O., Phon, T. H., Pfeifer, B., and Stephanopoulos, G. (2010) Isoprenoid Pathway Optimization for Taxol Precursor Overproduction in *Escherichia coli*. *Science* *330*, 70−74.

(6) Leonard, E., Ajikumar, P. K., Thayer, K., Xiao, W. H., Mo, J. D., Tidor, B., Stephanopoulos, G., and Prather, K. L. J. (2010) Combining metabolic and protein engineering of a terpenoid biosynthetic pathway for overproduction and selectivity control. *Proc. Natl. Acad. Sci. U.S.A.* *107*, 13654−13659.

(7) Anthony, J. R., Anthony, L. C., Nowroozi, F., Kwon, G., Newman, J. D., and Keasling, J. D. (2009) Optimization of the mevalonate-based isoprenoid biosynthetic pathway in *Escherichia coli* for production of the anti-malarial drug precursor amorpha-4,11-diene. *Metab. Eng.* *11*, 13−19.

(8) Ro, D. K., Paradise, E. M., Ouellet, M., Fisher, K. J., Newman, K. L., Ndungu, J. M., Ho, K. A., Eachus, R. A., Ham, T. S., Kirby, J., Chang, M. C., Withers, S. T., Shiba, Y., Sarpong, R., and Keasling, J. D. (2006) Production of the antimalarial drug precursor artemisinic acid in engineered yeast. *Nature* *440*, 940−943.

(9) Zhang, K., Li, H., Cho, K. M., and Liao, J. C. (2010) Expanding metabolism for total biosynthesis of the nonnatural amino acid L-homoalanine. *Proc. Natl. Acad. Sci. U.S.A.* *107*, 6234−6239.

(10) Lim, C. G., Fowler, Z. L., Hueller, T., Schaffer, S., and Koffas, M. A. G. (2011) High-Yield Resveratrol Production in Engineered *Escherichia coli*. *Appl. Environ. Microbiol.* *77*, 3451−3460.

(11) Lin, Y., Shen, X., Yuan, Q., and Yan, Y. (2013) Microbial biosynthesis of the anticoagulant precursor 4-hydroxycoumarin. *Nat. Commun.* *4*, 2603.

(12) Teigen, K., McKinney, J. A., Haavik, J., and Martinez, A. (2007) Selectivity and affinity determinants for ligand binding to the aromatic amino acid hydroxylases. *Curr. Med. Chem.* *14*, 455−467.

(13) Fitzpatrick, P. F. (2003) Mechanism of aromatic amino acid hydroxylation. *Biochemistry* *42*, 14083−14091.

(14) McKinney, J., Knappskog, P. M., Pereira, J., Ekern, T., Toska, K., Kuitert, B. B., Levine, D., Gronenborn, A. M., Martinez, A., and Haavik, J. (2004) Expression and purification of human tryptophan hydroxylase from *Escherichia coli* and *Pichia pastoris*. *Protein Expression Purif.* *33*, 185−194.

(15) Martinez, A., Knappskog, P. M., and Haavik, J. (2001) A structural approach into human tryptophan hydroxylase and its implications for the regulation of serotonin biosynthesis. *Curr. Med. Chem.* *8*, 1077−1091.

(16) Knight, E. M., Zhu, J., Förster, J., and Luo, H. (2013) Microorganisms for the production of 5-hydroxytryptophan. WO2013127914.

(17) Nakata, H., Yamauchi, T., and Fujisawa, H. (1979) Phenylalanine hydroxylase from *Chromobacterium violaceum*. Purification and characterization. *J. Biol. Chem.* *254*, 1829−1833.

(18) Zhao, G., Xia, T., Song, J., and Jensen, R. A. (1994) *Pseudomonas aeruginosa* possesses homologues of mammalian phenylalanine hydroxylase and 4$\alpha$-carbinolamine dehydratase/DCoH as part of a three-component gene cluster. *Proc. Natl. Acad. Sci. U.S.A.* *91*, 1366−1370.

(19) Kino, K., Hara, R., and Nozawa, A. (2009) Enhancement of L-tryptophan 5-hydroxylation activity by structure-based modification of L-phenylalanine 4-hydroxylase from *Chromobacterium violaceum*. *J. Biosci. Bioeng.* *108*, 184−189.

(20) Pribat, A., Blaby, I. K., Lara-Nunez, A., Gregory, J. F., III, de Crecy-Lagard, V., and Hanson, A. D. (2010) FolX and FolM are essential for tetrahydromonapterin synthesis in *Escherichia coli* and *Pseudomonas aeruginosa*. *J. Bacteriol.* *192*, 475−482.

(21) Erlandsen, H., Kim, J. Y., Patch, M. G., Han, A., Volner, A., Abu-Omar, M. M., and Stevens, R. C. (2002) Structural comparison of bacterial and human iron-dependent phenylalanine hydroxylases: Similar fold, different stability and reaction rates. *J. Mol. Biol.* *320*, 645−661.

(22) Tamura, K., Peterson, D., Peterson, N., Stecher, G., Nei, M., and Kumar, S. (2011) MEGA5: Molecular evolutionary genetics analysis using maximum likelihood, evolutionary distance, and maximum parsimony methods. *Mol. Biol. Evol.* *28*, 2731−2739.

(23) Cao, J., Shi, F., Liu, X., Huang, G., and Zhou, M. (2010) Phylogenetic analysis and evolution of aromatic amino acid hydroxylase. *FEBS Lett.* *584*, 4775−4782.

(24) Ekstrom, F., Stier, G., Eaton, J. T., and Sauer, U. H. (2003) Crystallization and X-ray analysis of a bacterial non-haem iron-containing phenylalanine hydroxylase from the Gram-negative opportunistic pathogen *Pseudomonas aeruginosa*. *Acta Crystallogr.* *D59*, 1310−1312.

(25) Gong, F., Ito, K., Nakamura, Y., and Yanofsky, C. (2001) The mechanism of tryptophan induction of tryptophanase operon expression: Tryptophan inhibits release factor-mediated cleavage of TnaC-peptidyl-tRNA(Pro). *Proc. Natl. Acad. Sci. U.S.A.* *98*, 8997−9001.

(26) Song, J., Xia, T., and Jensen, R. A. (1999) PhhB, a *Pseudomonas aeruginosa* homolog of mammalian pterin 4a-carbinolamine dehydratase/DCoH, does not regulate expression of phenylalanine hydroxylase at the transcriptional level. *J. Bacteriol.* *181*, 2789−2796.

(27) Zhao, Z. J., Zou, C., Zhu, Y. X., Dai, J., Chen, S., Wu, D., Wu, J., and Chen, J. (2011) Development of L-tryptophan production strains by defined genetic modification in *Escherichia coli*. *J. Ind. Microbiol. Biotechnol.* *38*, 1921−1929.

(28) Simat, T. J., and Steinhart, H. (1998) Oxidation of Free Tryptophan and Tryptophan Residues in Peptides and Proteins. *J. Agric. Food Chem.* *46*, 490−498.

(29) Huang, Q., Lin, Y., and Yan, Y. (2013) Caffeic acid production enhancement by engineering a phenylalanine over-producing *Escherichia coli* strain. *Biotechnol. Bioeng.* *110*, 3188−3196.

(30) Lin, Y., Sun, X., Yuan, Q., and Yan, Y. (2014) Extending shikimate pathway for the production of muconic acid and its precursor salicylic acid in *Escherichia coli*. *Metab. Eng.* *23C*, 62−69.

(31) Mora-Pale, M., Sanchez-Rodriguez, S. P., Linhardt, R. J., Dordick, J. S., and Koffas, M. A. (2014) Biochemical strategies for enhancing the in vivo production of natural products with pharmaceutical potential. *Curr. Opin. Biotechnol.* *25C*, 86−94.

(32) Higgins, C. A., Vermeer, L. M., Doorn, J. A., and Roman, D. L. (2012) Expression and purification of recombinant human tyrosine hydroxylase as a fusion protein in *Escherichia coli*. *Protein Expression Purif.* *84*, 219−223.

(33) Daubner, S. C., Lauriano, C., Haycock, J. W., and Fitzpatrick, P. F. (1992) Site-directed mutagenesis of serine 40 of rat tyrosine hydroxylase. Effects of dopamine and cAMP-dependent phosphorylation on enzyme activity. *J. Biol. Chem.* *267*, 12639−12646.

(34) Marcheschi, R. J., Gronenberg, L. S., and Liao, J. C. (2013) Protein engineering for metabolic engineering: Current and next-generation tools. *Biotechnol. J.* *8*, 545−555.

(35) Hara, R., and Kino, K. (2013) Enhanced synthesis of 5-hydroxy-L-tryptophan through tetrahydropterin regeneration. *AMB Express* *3*, 70.

(36) Satoh, Y., Tajima, K., Munekata, M., Keasling, J. D., and Lee, T. S. (2012) Engineering of L-tyrosine oxidation in *Escherichia coli* and microbial production of hydroxytyrosol. *Metab. Eng.* *14*, 603−610.

(37) Wang, J., Cheng, L. K., Wang, J., Liu, Q., Shen, T., and Chen, N. (2013) Genetic engineering of *Escherichia coli* to enhance production of L-tryptophan. *Appl. Microbiol. Biotechnol. 97*, 7587−7596.
(38) Datsenko, K. A., and Wanner, B. L. (2000) One-step inactivation of chromosomal genes in *Escherichia coli* K-12 using PCR products. *Proc. Natl. Acad. Sci. U.S.A. 97*, 6640−6645.
(39) Lin, Y., and Yan, Y. (2012) Biosynthesis of caffeic acid in *Escherichia coli* using its endogenous hydroxylase complex. *Microb. Cell Fact. 11*, 42.
(40) Shen, X., Lin, Y., Jain, R., Yuan, Q., and Yan, Y. (2012) Inhibition of acetate accumulation leads to enhanced production of (*R*,*R*)-2,3-butanediol from glycerol in *Escherichia coli*. *J. Ind. Microbiol. Biotechnol. 39*, 1725−1729.
