# Supplementary material for: 5-Hydroxytryptophan (5-HTP): Natural Occurrence, Analysis, Biosynthesis, Biotechnology, Physiology and Toxicology
Source: Int J Mol Sci. 2020 Dec 26;22(1):181. doi: 10.3390/ijms22010181 (PMC7796270; doi:10.3390/ijms22010181)
Supplement: Supplementary file 1 [file ijms-22-00181-s001.zip › 5-HTP.Data/PDF/2974329600/J. Biol. Chem.-1974-Letendre-7186-91.pdf]

THE JOURNAL OF BIOLOGICAL CHEMISTRY
Vol. 249, No. 22, Issue of November 25, pp. 7186-7191, 1974
Printed in U.S.A.

The Tryptophan Hydroxylase of *Chromobacterium violaceum*

(Received for publication, April 8, 1974)

CAROL H. LETENDRE, GENEVA DICKENS, AND GORDON GUROFF

From the Section on Intermediary Metabolism, Laboratory of Biomedical Sciences, National Institute of Child Health and Human Development, National Institutes of Health, Bethesda, Maryland 20014

SUMMARY

A tryptophan hydroxylase has been purified approximately 20-fold from *Chromobacterium violaceum*. The enzyme is inducible and it is induced to a greater extent by phenylalanine than by tryptophan. The hydroxylation requires molecular oxygen and a reduced pteridine, and has a further requirement for the addition of a sulfhydryl compound. No evidence for the further metabolism of the product, 5-hydroxytryptophan, could be obtained. The enzyme is inhibited by phenylalanine and *p*-chlorophenylalanine. It acts on D-tryptophan to some extent. The enzyme is different from the phenylalanine hydroxylase also produced by the organism.

The hydroxylation of tryptophan has been intensively studied in mammalian systems because it is the first and rate-limiting step in the biosynthesis of serotonin. The enzyme which catalyzes the hydroxylation, tryptophan 5-hydroxylase (EC 1.99.1.4), has been studied with cell-free preparations from brain (1-5), from pineal gland (4), from intestine (6), from mast cells (7, 8), and from carcinoid tumor (9, 10). It appears to be a typical mixed-function oxidase using tetrahydropteridine as a reducing agent and molecular oxygen as an oxidizing agent. In this regard, the enzyme seems to be similar in its mode of action to the more thoroughly studied phenylalanine hydroxylase (11-13) and to tyrosine hydroxylase (14, 15).

Although tryptophan hydroxylase is thought of as a mammalian enzyme, the first reports of tryptophan hydroxylation described studies in whole cells of *Chromobacterium violaceum* (16, 17). The function of tryptophan hydroxylation in this organism presumably is to provide precursor for the characteristic pigment produced by this species, violacein. These early investigations yielded information on the conversion, but did not succeed in obtaining a cell-free preparation which could be used to examine the reaction in detail. In fact, no cell-free bacterial system has been available with which to study this reaction.

Recent studies in this laboratory have shown that *Chromobacterium violaceum* contains a phenylalanine hydroxylase, similar to the phenylalanine hydroxylase from *Pseudomonas* sp. (ATCC 11299a) (18-20). Since a cell-free phenylalanine hydroxylase was easily obtained from this organism, an attempt was made to prepare a cell-free preparation which would hydroxylate tryptophan. This report describes the characteristics of a partially purified tryptophan hydroxylase from *Chromobacterium violaceum*.

EXPERIMENTAL PROCEDURES

Chromobacterium violaceum (ATCC 12540) was obtained from the American Type Culture Collection as a lyophilized powder. The cultures were revived in standard media and maintained on nutrient agar at room temperature. For induction studies and preparation of extracts used for product identification, cells were grown overnight at room temperature, with shaking, in 2-liter Erlenmeyer flasks. The growth medium contained K_2HPO_4, 0.2%; KH_2PO_4, 0.1%; $MgSO_4$, 0.02%; NH_4Cl, 0.2%; yeast extract, 0.02%; and L-phenylalanine, 0.2%. For some experiments the phenylalanine was replaced by either 0.2% L-tryptophan, 0.2% L-asparagine, or 0.2% L-tyrosine. The flasks contained 500 ml of the appropriate medium and were autoclaved at 120° and 120 pounds of pressure for 20 min before inoculation. For some enzyme preparation cells were grown in the above medium in a 50-liter fermentor.[1]

After overnight growth at room temperature in the case of 2-liter flasks and at 25° in the case of the fermentor, the cultures had usually reached an A_{540} of between 0.3 and 0.4. The contents of eight 2-liter flasks were combined, the cells collected by centrifugation, washed with 20 ml of 0.05 M acetate buffer, pH 6.0, and again collected. Following suspension in 8 ml of 0.05 M acetate, cells were passed through a 3-ml French pressure cell.

Cells from 50-liter cultures were centrifuged and washed as above, and finally suspended in 20 ml of 0.05 M acetate buffer, pH 6.0, for lysis.

Lysates were centrifuged at 12,000 × *g* for 10 min, and the supernatant portion was fractionated between 45 and 60% saturation with solid ammonium sulfate. Each precipitation was done for 20 min at 0° with stirring, and the precipitates were collected by centrifugation at 12,000 × *g*. The precipitate obtained by such fractionation was taken up in 2 ml (6 ml in the case of the fermentor-grown cells) of 0.05 M acetate, pH 6.0, and dialyzed against 2 liters of the same buffer. The dialyzed preparation was stored at −20° until used.

One such preparation resulting from a 50-liter growth or four preparations from growth in 2-liter flasks were combined, centrifuged, and applied to a column (1.2 × 11 cm) of DEAE-cellulose, previously equilibrated with 0.05 M acetate, pH 6.0. The column was washed with the same buffer and eluted with a gradient composed of 50 ml of 0.05 M acetate and 50 ml of 0.05 M acetate containing 0.5 M NaCl. The eluate was collected in 5-ml fractions, and the A_{280} and the activity of each fraction were measured. Usually about 20 fractions were collected with the peak activity occurring in tubes 13 or 14. The active fractions were individually brought to 60% saturation with solid ammonium sulfate and kept on ice for 20 min after the ammonium sulfate was dissolved by gentle stirring. The precipitates were collected by centrifugation, the tubes carefully drained, and the pellets dissolved in a

[1] We are indebted to Mr. David Rogerson of the Pilot Plant Area maintained by the National Institute of Arthritis, Metabolism, and Digestive Diseases for the use of their facilities.

Downloaded from http://www.jbc.org/ by guest on November 17, 2020

5-ml portion of the acetate buffer. This preparation was divided into 1-ml portions and frozen at −20°. Any attempt to dialyze the sample at this stage led to large losses of activity. The frozen samples were stable for several days but the enzyme seemed to lose activity after a few hours at 0°.

Hydroxylase activity was assayed by measuring the fluorescence of the 5-hydroxytryptophan produced, as described by Friedman, Kappelman, and Kaufman (5) and modified by Baumgarten, Victor, and Lovenberg (21). The incubation mixtures, unless otherwise indicated, contained Tris·HCl buffer, pH 7.4, 60 μmoles; Cleland's reagent, 1 μmole; 2-amino-6,7-dimethyl-4-hydroxy-5,6,7,8-tetrahydropteridine, 0.1 μmole; L-tryptophan, 0.5 μmole; and enzyme in a total volume of 280 μl. All the components of the mixture, except the tryptophan, were preincubated at room temperature for 10 min, and then the tryptophan was added and the incubation continued for 60 min, also at room temperature. The reaction was terminated by the addition of 25 μl of 6 N perchloric acid, and the tubes were centrifuged at 12,000 × g for 5 min. To 1.1 ml of 3 N HCl, 200 μl of the sample were added and the fluorescence was measured in an Aminco-Bowman spectrophotofluorimeter at 290 nm activation and 540 nm emission. Phenylalanine hydroxylase was measured by the isotopic method of Guroff and Abramowitz (22). Protein was estimated using the method of Lowry *et al.* (23).

Paper chromatograms involving radioactive compounds were scanned in a Packard model 7201 radiochromatogram scanner.

2-Amino-6,7-dimethyl-4-hydroxy-5,6,7,8-tetrahydropteridine was obtained from Aldrich Chemical Company. Dihydropteridines were the gift of Dr. Ted Shiota, University of Alabama, Birmingham. These compounds were reduced to the tetrahydro form in the presence of hydrogen and platinum oxide prior to use. All other chemicals were from standard commercial sources.

RESULTS

Growth of Cells and Induction of Tryptophan Hydroxylase—Cells grown overnight on 0.2% L-phenylalanine, 0.2% L-tryptophan, 0.2% L-asparagine, or 0.2% L-tyrosine as carbon source grew to optical densities at 540 nm of 0.32, 0.16, 1.35, and 0.30, respectively. When extracts from these cells were assayed for tryptophan hydroxylase by the standard assay and compared with a zero time control, *i.e.* a complete incubation to which perchloric acid was added before the substrate, they had activities of 1.3 nmoles, 0.5 nmole, 0.1 nmole, and 0.1 nmole of 5-hydroxytryptophan produced per mg of protein per hour, respectively. Thus, although the growth on asparagine was a great deal higher and that on phenylalanine and tyrosine was about equal, enzyme activity was seen only in the case of phenylalanine- and tryptophan-grown cells. Enzyme activity following growth on phenylalanine was at least twice as great as that on tryptophan. In addition, the substrate blanks obtained with crude extracts from phenylalanine-grown cells were much lower than those with comparable extracts from tryptophan cells; this most probably was due to the presence of endogenous 5-hydroxytryptophan in the cells. For these several reasons the following studies were done with extracts from phenylalanine-grown cells.

Identification of Product—The dialyzed crude extract was used to identify the product of the reaction. The incubations were set up basically as described under "Experimental Procedures." When radioactive tryptophan was used as substrate, the carrier was omitted from the incubations and the reactions were stopped by placing them for 1 min at 100°. The tubes were centrifuged and the supernatant portions chromatographed. No perchloric acid or HCl was used.

When extracts were incubated with L-[^{14}C]tryptophan, boiled, centrifuged, and the supernatant portions chromatographed with added internal standards, a radioactive component appeared at exactly the R_F of carrier 5-hydroxytryptophan (Fig. 1). The radioactivity in this component increased with time of incubation, and was not present in samples boiled for 1 min before the addition of the isotope, nor in samples from incubations lacking the tetrahydropteridine cofactor.

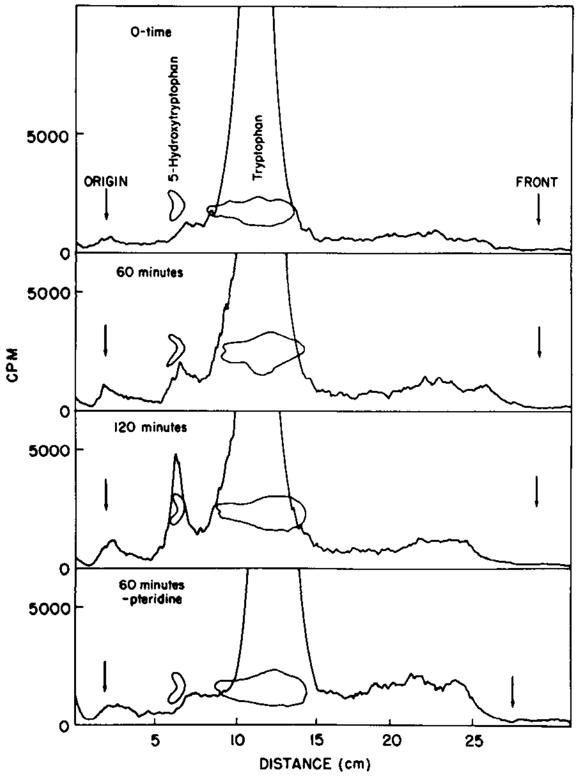

FIG. 1. Formation of 5-hydroxytryptophan by crude dialyzed extracts of *Chromobacterium violaceum*. The incubations were carried out as described under "Experimental Procedures." They contained 1.6 mg of extract protein. The carrier tryptophan was omitted and L-[3-^{14}C]tryptophan (New England Nuclear), 1 μCi, was added to each incubation (specific activity 100 μCi/900 mg). The tubes were boiled for 1 min at the end of the incubation time, the denatured protein was centrifuged out, and 150 μl of the supernatant portion were chromatographed. Tryptophan and 5-hydroxytryptophan were added as internal standards. The samples were applied to Whatman No. 3MM paper and the papers chromatographed in ascending fashion in butanol-acetic acid-H_2O (120:30:50) as solvent. The R_Fs of standards were: tryptophan, 0.43; 5-hydroxytryptophan, 0.14; 5-hydroxytryptamine, 0.41. The 5-hydroxytryptophan standards were observed after passing the dried papers through HCl vapors and inspecting for fluorescent spots. Radioactive areas were located using a Radiochromatogram scanner in which full scale deflection (the *ordinate*) was equivalent to 10,000 cpm.

Similar experiments were done with the same extracts, using hydroxy-[5-^{14}C]tryptophan instead of tryptophan as substrate. In these experiments no new radioactive products appeared, and specifically, no radioactivity could be detected at the R_F of 5-hydroxytryptamine (serotonin). These data suggest that, under these conditions, no decarboxylation of the 5-hydroxytryptophan occurred.

When crude dialyzed extracts were incubated with substrate levels of tryptophan and the reaction stopped with perchloric acid as usual, the product could also be identified as 5-hydroxytryptophan by its characteristic fluorescence (Fig. 2). The activation and emission characteristics of the product corresponded very well to those of standard 5-hydroxytryptophan. Any such products were lacking in samples which had been acidified before the additon of the substrate.

Purification of Enzyme and Dependences of Its Action—The simple purification steps applied here led to approximately a

Downloaded from http://www.jbc.org/ by guest on November 17, 2020

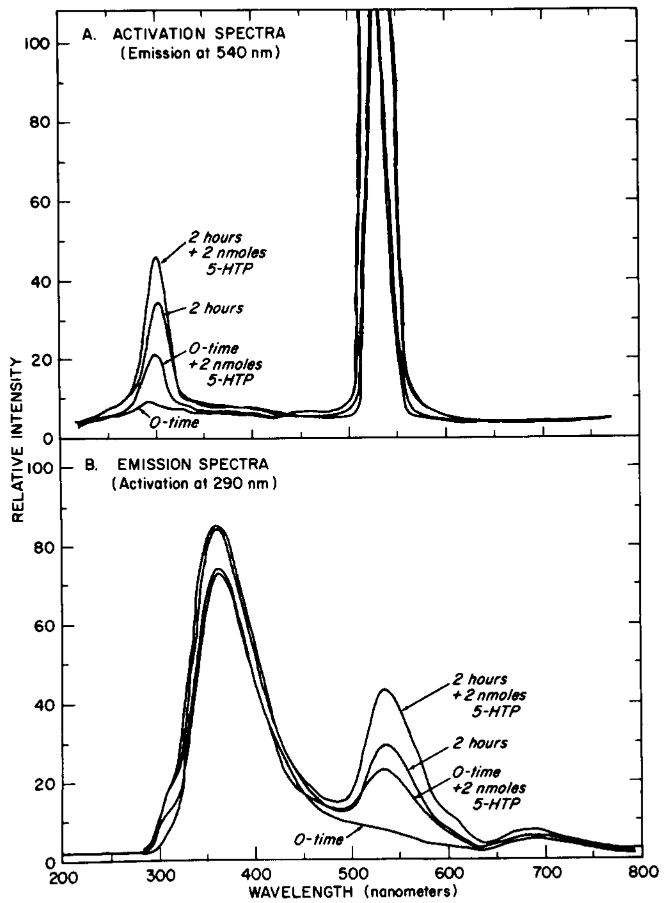

Fig. 2. Fluorescent spectra of the product as compared with 5-hydroxytryptophan (*5-HTP*). Incubation mixtures were carried out as described under "Experimental Procedures" with 1.6 mg of extract protein. After the spectra had been determined, 2 nmoles of 5-hydroxytryptophan were added to both zero time and 2-hour samples and the spectra re-examined.

Table I

Purification of tryptophan hydroxylase from Chromobacterium violaceum

Step	Volume	Total units[a]	Protein	Specific activity	Yield
	ml		*mg*	*units/mg protein*	%
Crude extract[b]	32	297	371.5	0.8	(100)
45-60% ammonium sulfate fraction[b]	8	97	42	2.3	33
DEAE-cellulose eluate	5	149	8.5	17.5	52

[a] One unit of enzyme is that amount which will produce 1 nmole of 5-hydroxytryptophan in 1 hour under standard conditions.

[b] Calculated for four preparations before collection at the final step.

20-fold purification and a 50% yield of activity (Table I). The increase in total activity following the DEAE-cellulose step cannot be explained at the present time. Obviously, it may involve the removal of some inhibitory protein or the activation of the enzyme by some constituent of the ammonium sulfate which could not be dialyzed away without marked losses in activity (see "Experimental Procedures"). It is interesting to note here that dialysis of the crude lysates also frequently led to increases in total activity.

Table II

Dependence of purified tryptophan hydroxylase activity on various components of reaction mixture

In Experiment A, the incubations contained 260 μg of purified enzyme. In Experiment B, 170 μg of a different preparation were used. The other components are as described under "Experimental Procedures."

Assay conditions	Activity
	nmoles 5-HTP[a] produced
Experiment A	
Control	1.6
— Enzyme	0.4
— Tryptophan	0.2
— $DMPH_4$	0.5
— Cleland's reagent	0.4
Without preincubation	1.5
Experiment B	
Control	3.0
— Enzyme	0.4
— O_2[b]	1.2

[a] 5-HTP, 5-hydroxytryptophan; $DMPH_4$, 2-amino-6,7-dimethyl-4-hydroxy-5,6,7,8-tetrahydropteridine.

[b] Preincubation and incubation carried out under N_2.

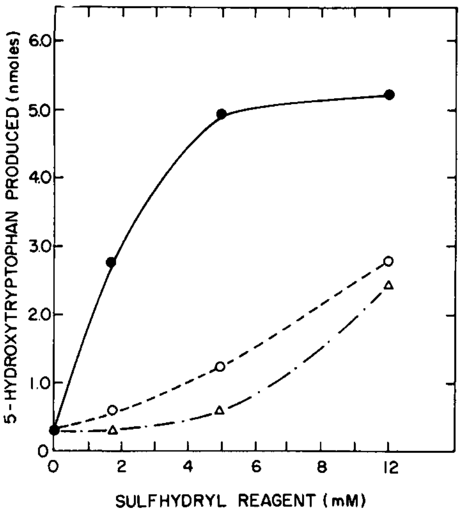

Fig. 3. Comparison of sulfhydryl reagents in action of partially purified *Chromobacterium* tryptophan hydroxylase. Incubations were carried out as described under "Experimental Procedures" except that sulfhydryl reagents in the amounts indicated replaced the 1 μmole of Cleland's reagent in the standard reaction mixture. Incubations were carried out with 46 μg of partially purified enzyme protein. ○, Cleland's reagent; ●, glutathione; △, β-mercaptoethanol.

The activity of the purified enzyme was dependent on the addition of a number of components (Table II). These included, of course, enzyme and substrate, and also reduced pteridine cofactor, Cleland's reagent, and oxygen. The oxygen requirement was not complete, but this probably reflects the lack of rigorous anaerobiasis in our experiments rather than a true lack of dependence. If the incubation is done under N_2 but the preincubation is not, then the dependence on oxygen is even less obvious, although still discernible.

The requirement for a sulfhydryl reagent can be satisfied by either Cleland's reagent, glutathione, or β-mercaptoethanol as seen in Fig. 3. Maximum activity was attained at about 5 mM

Downloaded from http://www.jbc.org/ by guest on November 17, 2020

TABLE III

Comparison of reducing agents on 5-hydroxytryptophan production by partially purified tryptophan hydroxylase

Incubations containing 95 μg of partially purified enzyme protein were carried out as described under "Experimental Procedures" except that volumes were doubled and Cleland's reagent was omitted except where indicated. Total volume in each case was 0.56 ml.

Incubation conditions	Activity	
	45 min	90 min
	nmoles 5-HTP produced	
Complete system	0	0
+ Ascorbic acid (5×10^{-3} M)	0.03	0
+ NADH (4.5×10^{-3} M)	0	0
+ Additional DMPH$_4$,[a] 0.01 mg at 45 min	0	0
+ Cleland's reagent (5×10^{-3} M)	0.77	1.01

[a] 2-Amino-6,7-dimethyl-4-hydroxy-5,6,7,8-tetrahydropteridine.

of Cleland's reagent which was the most efficient of those compounds tested.

Cleland's reagent has been shown to be effective in regeneration of tetrahydropterin in at least two related reactions, tryptophan hydroxylase of rabbit hindbrain (5) and *Pseudomonas* sp. phenylalanine hydroxylase.[2] The experiment presented in Table III was carried out to determine if a reducing agent was required for enzyme activity or for the maintenance of pteridine cofactor in the reduced form. The enzymatic production of 5-hydroxytryptophan was determined after 45 and 90 min in the presence of ascorbic acid, NADH, or Cleland's reagent, compounds which have been shown to regenerate tetrahydropterins in other systems[2] (5, 24). Measurable amounts of 5-hydroxytryptophan were produced only in the presence of Cleland's reagent. Although reduced tetrahydropterin was presumably maintained by ascorbic acid and NADH and was certainly present after a second addition of 6,7-dimethyltetrahydropteridine, the enzyme was inactive except in the presence of a sulfhydryl reagent.

The activity of the purified enzyme was fairly linear with protein concentration in the range of 2 to 4 nmoles of product formed but frequently gave less than proportional response below 1 to 2 nmoles (Fig. 4*A*). The reaction was linear with time up to 60 min (Fig. 4*B*). The enzyme exhibited a rather broad pH curve under these conditions with an optimum at about pH 7.0 (Fig. 5).

Kinetics and Specificity of Purified Enzyme—The purified enzyme exhibited fairly straightforward Michaelis-Menten kinetics for both substrate and 6,7-dimethyltetrahydropteridine (Figs. 6 and 7). The K_m values for tryptophan and for reduced pteridine were 2.2×10^{-3} M and 1.3×10^{-4} M, respectively. Again, there was less than proportional response at low levels of product formed.

The action of the enzyme was about 50% inhibited by either α,α-dipyridyl or EDTA at concentrations in the order of 1 to 2 $\times 10^{-5}$ M (Table IV, Experiments A and B). The requirement for tetrahydropteridine could not be satisfied by either ascorbic acid or NADH (Table IV, Experiment C).

The enzyme used D-tryptophan about one-third as well as it

[2] Letendre, C. H., Dickens, G., and Guroff, G., manuscript in preparation.

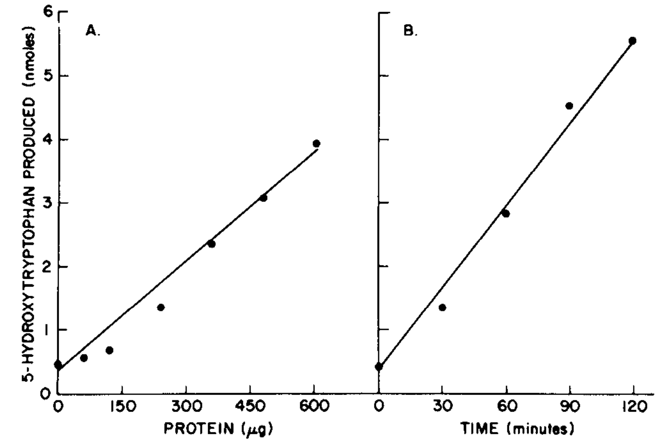

FIG. 4. *A*, enzyme curve of partially purified *Chromobacterium* tryptophan hydroxylase. *B*, time curve of the action of partially purified *Chromobacterium* tryptophan hydroxylase. Incubations were run as described under "Experimental Procedures" with 100 μg of partially purified enzyme protein.

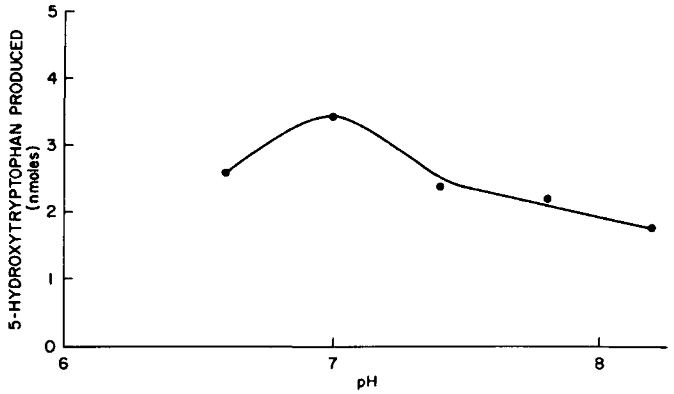

FIG. 5. Influence of pH on the action of partially purified *Chromobacterium* tryptophan hydroxylase. Reaction mixtures were incubated as described under "Experimental Procedures" with 270 μg of partially purified enzyme protein. Tris buffers were used for all pH points.

used L-tryptophan (Table IV, Experiment D). This experiment confirms earlier work which showed that whole cells would convert D-tryptophan to hydroxyindoles (17). L-Phenylalanine, L-tyrosine, and *p*-chloro-DL-phenylalanine inhibited the hydroxylation of L-tryptophan, with phenylalanine being the most effective. When the nature of the inhibition by L-phenylalanine was investigated, it appeared to be a competitive inhibitor, but the lack of linear response at very low levels of product formation made presentable data difficult to get.

The bacterial tryptophan hydroxylase was able to utilize the synthetic tetrahydropteridine, 6,7-dimethyltetrahydropteridine, as well as a number of naturally occurring tetrahydropteridines. As seen in Fig. 8, both L-*threo*-neopterin, the chief pteridine in *Escherichia coli* (25) and *Pseudomonas* sp. (20) and biopterin, the natural cofactor for the rat liver phenylalanine hydroxylase (26) were the most active cofactors in 5-hydroxytryptophan production. Both D- and L-*erythro*-neopterins were less active as cofactors. Neither L-*threo*-neopterin nor L-*erythro*-biopterin permitted more than 31% of the activity seen with the synthetic tetrahydropteridine which was by far the most active of those tried.

Since the phenylalanine hydroxylase of rat liver will also hydroxylate tryptophan (27) and since the *Chromobacterium* has an active phenylalanine hydroxylase, it was important to show that

Downloaded from http://www.jbc.org/ by guest on November 17, 2020

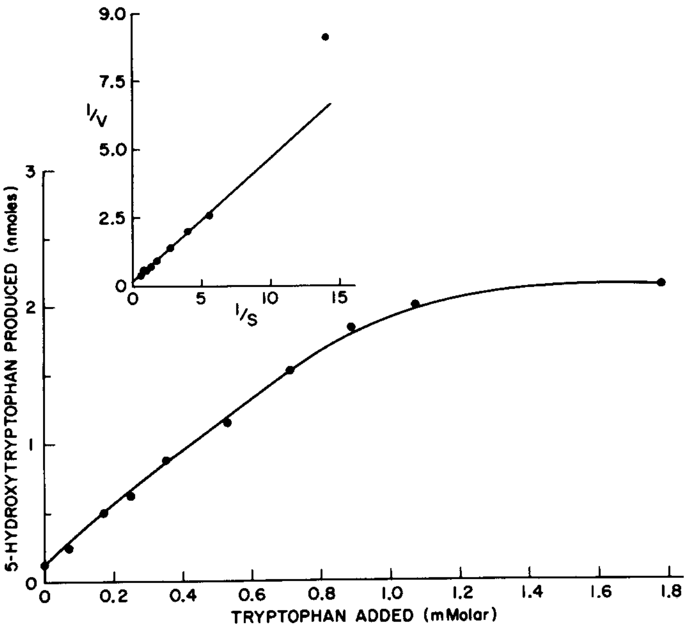

FIG. 6. Influence of substrate concentration on the action of partially purified *Chromobacterium* tryptophan hydroxylase. Reaction mixtures were incubated as described under "Experimental Procedures" with 430 μg of partially purified enzyme protein.

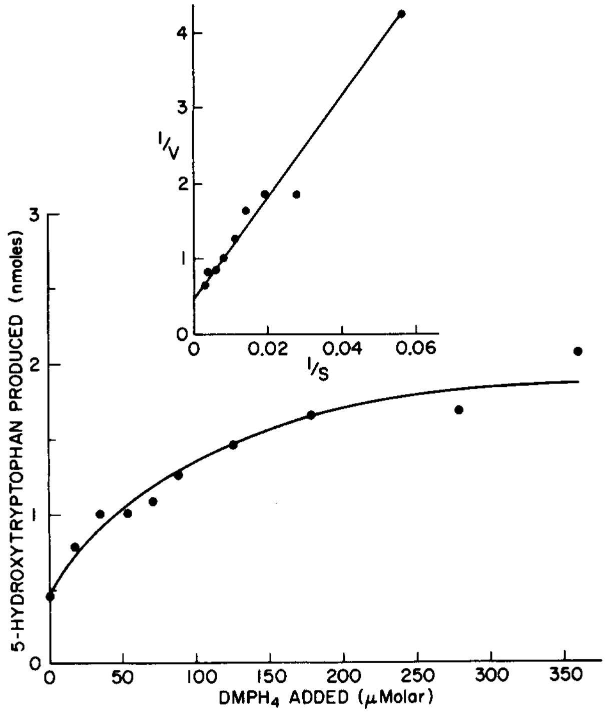

FIG. 7. Influence of 2-amino-6,7-dimethyl-4-hydroxy-5,6,7,8-tetrahydropteridine (*DMPH*$_4$) concentration on the action of partially purified *Chromobacterium* tryptophan hydroxylase. Reaction mixtures were incubated as described under "Experimental Procedures" with 430 μg of partially purified enzyme protein.

the tryptophan hydroxylating activity studied here was not merely a reflection of the phenylalanine hydroxylase enzyme in this organism. Purification of the tryptophan hydroxylase activity was not accompanied by purification of the phenylalanine hydroxylase (Table V). Other experiments showed that

TABLE IV

Influence of other compounds on action of purified tryptophan hydroxylase from Chromobacterium violaceum

Incubations carried out as described under "Experimental Procedures" using 470 μg of partially purified enzyme protein.

Incubation conditions	Activity
	nmoles 5-HTP produced
Experiment A	
Complete	4.8
+ α,α-Dipyridyl (1.8 × 10^{-5} M)	2.8
Experiment B	
Complete	2.2
+ EDTA (1.8 × 10^{-5} M)	0.8
Experiment C	
Complete	3.2
− Tetrahydropteridine + ascorbic acid (1 × 10^{-3} M)	0.4
− Tetrahydropteridine + NADH (1 × 10^{-3} M)	0.2
− Tetrahydropteridine	0.4
Experiment D	
Complete	1.9
− L-Tryptophan	0.1
− L-Tryptophan + D-tryptophan (0.5 μmole)	0.7
Experiment E	
Complete	3.7
+ L-Phenylalanine (0.5 μmole)	1.1
+ L-Tyrosine (0.5 μmole)	2.0
+ *p*-Chloro-DL-phenylalanine (0.5 μmole)	1.3

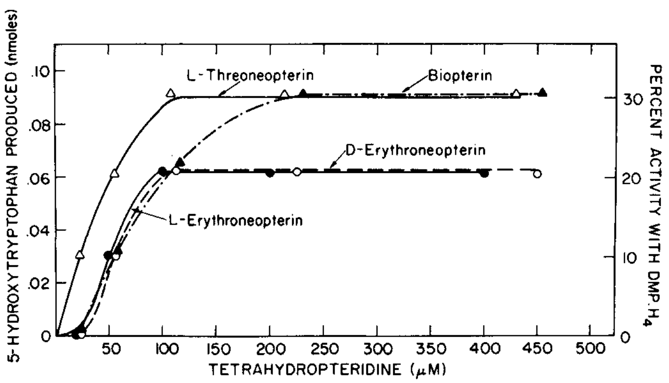

FIG. 8. Pteridine specificity of partially purified *Chromobacterium* tryptophan hydroxylase. Incubations with 40 μg of partially purified enzyme protein were carried out as described under "Experimental Procedures" except that Cleland's reagent was present at 10 mM and 2-amino-6,7-dimethyl-4-hydroxy-5,6,7,8-tetrahydropteridine was replaced by the various tetrahydropteridines as indicated. Concentrations of these compounds were determined spectrophotometrically immediately prior to use.

TABLE V

Comparison of tryptophan and phenylalanine hydroxylating actvities in crude and purified enzyme

Enzyme	Tryptophan hydroxylase	Phenylalanine hydroxylase
	nmoles/mg protein/hr	*nmoles/mg protein/10 min*
Crude extract	0.6	26.4
Purified tryptophan hydroxylase	10.0	32.5
Purification	16.7	1.2

Downloaded from http://www.jbc.org/ by guest on November 17, 2020

the two activities did not have the same temperature optimum, the same pH optimum, nor the same over-all stability. Thus, the two enzymes are clearly quite distinct.

DISCUSSION

Although several groups have reported on tryptophan hydroxylases from mammalian sources, this appears to be the first report of a cell-free preparation from a microorganism. The tryptophan hydroxylase from *Chromobacterium* has, at least superficially, the characteristics of the tryptophan hydroxylases found in various mammalian tissues. That is, it appears to be a mixed function oxidase using tetrahydropteridine as the reducing agent. The *Chromobacterium* enzyme also has a stringent requirement for sulfhydryl, and Cleland's reagent was found to be the most effective of the compounds tested. Our experiments strongly suggest that the sulfhydryl compound is essential to enzyme activity rather than in the maintenance of a reduced pteridine cofactor as is the case in rabbit hindbrain tryptophan hydroxylase (5). An iron requirement could not be shown clearly since the enzyme was inhibited by EDTA and by α,α-dipyridyl to about the same extent. Other experiments have shown that the addition of Fe^{++} does not stimulate the reaction. Another property of the bacterial enzyme which resembles reported observations with the mammalian enzyme is its inhibition by phenylalanine and *p*-chlorophenylalanine (8, 10). The K_m values determined here for tetrahydropteridine and for substrate are on the order of 10-fold higher than those reported for pineal or brainstem enzyme (4).

The nonspecificity of bacterial tryptophan hydroxylase is a property in common with many of the other mammalian and bacterial hydroxylases[2, 3] (28). Further investigation may reveal that the properties of this enzyme, like those of the mammalian tryptophan (5) and phenylalanine hydroxylases (5, 29) may be altered in the presence of the natural cofactor.

The relationship between the tryptophan and phenylalanine hydroxylases in various sources is an interesting one. The liver phenylalanine hydroxylase will hydroxylate tryptophan (27). The mast cell and the pineal tryptophan hydroxylases will hydroxylate phenylalanine (8, 10). In the present work it has been shown clearly that the two enzymes are distinct in *Chromobacterium*. But the preparation studied here retained some ability to hydroxylate phenylalanine. Since the enzyme is only partially purified it is not possible to decide if it has phenylalanine hydroxylase activity of its own or if the activity is merely a reflection of residual phenylalanine hydroxylase. The fact that phenylalanine inhibits the formation of 5-hydroxytryptophan, probably in a competitive manner, suggests the former possibility.

The function of the tryptophan hydroxylase in *Chromobacterium* is, of course, quite different than that of animals in which it certainly serves in the pathway to serotonin. In *Chromobacterium* there is no evidence for the formation of serotonin, either in whole cells, or in the present studies with crude extracts. Presumably the hydroxytryptophan produced by *Chromobacterium* is precursor to the characteristic pigment of these cells, violacein.

Finally, the induction of the enzyme by phenylalanine requires some comment. The studies presented here are preliminary in the sense that we do not know at what stage of growth the enzyme is induced, nor if it is induced by tryptophan even in the presence of an unrelated source of carbon and nitrogen. What we do know is that it is induced better by phenylalanine than by tryptophan, its substrate. It must be remembered, however, that tryptophan is a very poor substrate for the growth of the organism and so measurement of hydroxylase and evaluation of stages of growth is very difficult. It is of interest that two pteridine-requiring enzymes, tryptophan hydroxylase and phenylalanine hydroxylase, appear to be linked together as regards their induction by phenylalanine. Such findings pose a further question as to the inducibility of the pteridine-synthesizing or -metabolizing enzymes in this organism by phenylalanine and tryptophan since it has recently been shown that phenylalanine induces the dihydropteridine and dihydrofolate reductases, as well as phenylalanine hydroxylase, in *Pseudomonas* sp.[4]

[3] Guroff, G., Dickens, G., Bensinger, R., Yamabe, H., and Lovenberg, W., unpublished experiments.

[4] Williams, C. D., Letendre, C. H., Guroff, G., Hains, C., and Shiota, T., manuscript in preparation.

REFERENCES

1. Grahame-Smith, D. G. (1964) *Biochem. Biophys. Res. Commun.* **16**, 586
2. Grahame-Smith, D. G. (1967) *Biochem. J.* **105**, 351
3. Ichiyama, A., Nakamura, S., Nishizuka, Y., and Hayaishi, O. (1970) *J. Biol. Chem.* **245**, 1699
4. Jequier, E., Robinson, D. S., Lovenberg, W., and Sjoerdsma, A. (1969) *Biochem. Pharmacol.* **18**, 1071
5. Friedman, P. A., Kappelman, A. H., and Kaufman, S. (1972) *J. Biol. Chem.* **247**, 4165
6. Noguchi, T., Nishino, M., and Kido, R. (1973) *Biochem. J.* **131**, 375
7. Hosoda, S., and Glick, D. (1966) *J. Biol. Chem.* **241**, 192
8. Sato, T. L., Jequier, E., Lovenberg, W., and Sjoerdsma, A. (1967) *Eur. J. Pharmacol.* **1**, 18
9. Grahame-Smith, D. G. (1967) *Clin. Sci.* **33**, 147
10. Lovenberg, W., Jequier, E., and Sjoerdsma, A. (1967) *Science* **155**, 217
11. Kaufman, S. (1970) *Methods Enzymol.* **17A**, 603
12. Kaufman, S., and Fisher, D. B. (1970) *J. Biol. Chem.* **245**, 4745
13. Fisher, D. B., Kirkwood, R., and Kaufman, S. (1972) *J. Biol. Chem.* **247**, 5161
14. Nagatsu, T., Levitt, M., and Udenfriend, S. (1964) *J. Biol. Chem.* **239**, 2910
15. Ikeda, M., Fahien, L. A., and Udenfriend, S. (1966) *J. Biol. Chem.* **241**, 4452
16. Mitoma, C., Weissbach, H., and Udenfriend, S. (1955) *Nature* **175**, 994
17. Mitoma, C., Weissbach, H., and Udenfriend, S. (1956) *Arch. Biochem. Biophys.* **63**, 122
18. Guroff, G., and Ito, T. (1965) *J. Biol. Chem.* **240**, 1175
19. Guroff, G., and Rhoads, C. A. (1967) *J. Biol. Chem.* **242**, 3641
20. Guroff, G., and Rhoads, C. A. (1969) *J. Biol. Chem.* **244**, 142
21. Baumgarten, H. G., Victor, S. J., and Lovenberg, W. (1974) *J. Neurochem.*, in press
22. Guroff, G., and Abramowitz, A. (1967) *Anal. Biochem.* **19**, 548
23. Lowry, O. H., Rosebrough, N. J., Farr, A. L., and Randall, R. J. (1951) *J. Biol. Chem.* **193**, 265
24. Stone, K. J., and Townsley, B. H. (1973) *Biochem. J.* **131**, 611
25. Rembold, H., and Heinz, G. (1971) *Hoppe-Seyler's Z. Physiol. Chem.* **352**, 1271
26. Kaufman, S. (1963) *Proc. Natl. Acad. Sci. U. S. A.* **50**, 1085
27. Renson, J., Weissbach, H., and Udenfriend, S. (1962) *J. Biol. Chem.* **237**, 2261
28. Friedman, P. A., and Kaufman, S. (1973) *Biochim. Biophys. Acta* **293**, 56
29. Kaufman, S. (1971) *Advan. Enzymol.* **35**, 245

Downloaded from http://www.jbc.org/ by guest on November 17, 2020

The Tryptophan Hydroxylase of *Chromobacterium violaceum*

Carol H. Letendre, Geneva Dickens and Gordon Guroff

J. Biol. Chem. 1974, 249:7186-7191.

Access the most updated version of this article at http://www.jbc.org/content/249/22/7186

Alerts:

- When this article is cited
- When a correction for this article is posted

Click here to choose from all of JBC's e-mail alerts

This article cites 0 references, 0 of which can be accessed free at
http://www.jbc.org/content/249/22/7186.full.html#ref-list-1

Downloaded from http://www.jbc.org/ by guest on November 17, 2020
