# Supplementary material for: 5-Hydroxytryptophan (5-HTP): Natural Occurrence, Analysis, Biosynthesis, Biotechnology, Physiology and Toxicology
Source: Int J Mol Sci. 2020 Dec 26;22(1):181. doi: 10.3390/ijms22010181 (PMC7796270; doi:10.3390/ijms22010181)
Supplement: Supplementary file 1 [file ijms-22-00181-s001.zip › 5-HTP.Data/PDF/3180434149/Moran-1999-A-continuous-fluorescence-assay-for.pdf]

Analytical Biochemistry **266,** 148–152 (1999)
Article ID abio.1998.2956, available online at http://www.idealibrary.com on IDEAL®

A Continuous Fluorescence Assay for Tryptophan Hydroxylase

Graham R. Moran* and Paul F. Fitzpatrick*,†,[1]
*Department of Biochemistry and Biophysics and †Department of Chemistry, Texas A & M University, College Station, Texas 77843

Received August 7, 1998

A continuous fluorometric assay for tryptophan hydroxylase activity based on the different spectral characteristics of tryptophan and 5-hydroxytryptophan is presented. Hydroxylation of tryptophan at the 5-position results in a large increase in the fluorescence of the molecule. The assay selectively monitors the fluorescence yield of 5-hydroxytryptophan by exciting the reaction mix at 300 nm. The rate of increase of the emission signal was found to be directly proportional to the enzyme concentration. Inner filter effects due to quinonoid dihydropterin accumulation were eliminated by the inclusion of a thiol reductant. Activity measured using this assay method was found to be the same as that determined by established discontinuous HPLC assay methods. The application of the assay to routine activity measurements and to steady-state determinations with the substrates tryptophan and tetrahydropterin is described. © 1999 Academic Press

Tryptophan hydroxylase (TRH;[2] EC 1.14.16.4) is a member of the family of pterin-dependent aromatic amino acid hydroxylases, along with phenylalanine hydroxylase (PAH) and tyrosine hydroxylase (TYH). Each catalyzes the hydroxylation of the ring of a specific aromatic amino acid substrate. The electrons required for the reductive activation of molecular oxygen are from tetrahydrobiopterin (BH_4).

[1] To whom correspondence should be addressed at Department of Biochemistry and Biophysics, Texas A & M University, College Station, TX 77843-2128.

[2] Abbreviations used: TRH, tryptophan hydroxylase; PAH, phenylalanine hydroxylase; TYH, tyrosine hydroxylase; $TRH_{102-416}$, tryptophan hydroxylase mutant protein lacking 101 residues from the amino terminus and 28 residues from the carboxyl terminus; DTT, dithiothreitol; 6-$MePH_4$, 6-methyltetrahydropterin; BH_4, tetrahydrobiopterin.

Characterization of TRH has not kept pace with that of PAH and TYH due to the low yield, instability, and low specific activity of the wild-type enzyme from native or recombinant sources (1–9). Enzymological investigations of TRH have used a variety of discontinuous radioenzymatic or colorimetric assays (1, 10, 11), the most recent of which rely on direct fluorescence detection of the substrate and product after separation by HPLC (12, 13). These assays have proven appropriate for wild-type TRH as they offer high sensitivity and permit the assessment of the activity of numerous fractions within a relatively short period. However, the general limitation of discontinuous assays is that they provide insufficient resolution in the time axis to accurately describe a rate without exhaustive application of the method. An alternative for TRH is to continuously monitor the fluorescence due to the product 5-hydroxytryptophan as it accumulates during turnover.

A continuous spectrophotometric assay for TRH activity that takes advantage of the different spectral properties of tryptophan and 5-hydroxytryptophan is described here. Methods for the application of the assay both to routine activity determinations and to kinetic analyses are presented.

EXPERIMENTAL PROCEDURES

Materials

6-Methyltetrahydropterin was purchased from Shircks Laboratories, while tetrahydrobiopterin was from Calbiochem. Tryptophan, 5-hydroxytryptophan, and ferrous ammonium sulfate were obtained from Sigma Chemical Co. Mes and DTT were purchased from Research Organics, Inc. Catalase was from Boehringer-Mannheim.

$TRH_{102-416}$ was prepared as previously described, excluding the ceramic hydroxyapatite chromatographic step (14). The specific activity of the protein prepared in this manner was typically 1.6 μmol/mg min, and it

0003-2697/99 $30.00
Copyright © 1999 by Academic Press
All rights of reproduction in any form reserved.

was judged by SDS–PAGE to be greater than 85% pure.

The concentrations of tryptophan and 5-hydroxytryptophan were determined using an ϵ_{278} value of 5500 M^{-1} cm^{-1}. The concentrations of both 6-MePH_4 and BH_4 were determined in 2 M HCl using ϵ_{266} values of 17,800 and 18,000 M^{-1} cm^{-1}, respectively. The concentration of $TRH_{102-416}$ was determined using an ϵ_{280} value of 35,200 M^{-1} cm^{-1}. Stock solutions of tryptophan and tetrahydropterin were made in 10 mM HCl.

Fluorometric Assays for Tryptophan Hydroxylase

Routine activity measurements. Routine assays of activity in crude or purified TRH samples were made using an SLM Aminco 8000 spectrofluorometer. The standard reaction conditions were 60 μM tryptophan, 300 μM 6-methyltetrahydropterin, 200 mM ammonium sulfate, 7 mM DTT, 25 μg/ml catalase, 25 μM ferrous ammonium sulfate, 50 mM Mes, pH 7.0, with atmospheric oxygen. A stock reaction mix containing all of the above substituents excluding 6-MePH_4 was prepared and kept on ice. For each assay 970 μl of this reaction mix was equilibrated to 15°C for 2 min in a 1-ml fluorescence cuvette. The enzyme sample (10–20 μl) was then added, and the solution was allowed to equilibrate for a further minute. The assay was then initiated by the addition of 10 μl of a 30 mM stock of 6-MePH_4. Activity was measured by exciting the sample at 300 nm across the 4-mm axis of the cuvette and monitoring the emission at 330 nm using an 8-nm band pass.

Kinetic Analysis

Studies in which one of the three substrates were varied in successive assays for the determination of kinetic parameters were undertaken with either an SLM Aminco 8000 fluorometer or an Applied Photophysics stopped-flow spectrophotometer operating in fluorescence mode. The assay conditions of these studies were 200 mM ammonium sulfate, 7 mM DTT, 25 μg/ml catalase, 25 μM ferrous ammonium sulfate, 50 mM Mes, pH 7.0, with atmospheric oxygen at 15°C. These conditions were obtained by mixing equal volumes of two solutions preequilibrated to 15°C. Solution A, an enzyme solution, contained 50–100 nM TRH, 100 mM Mes, pH 7.0, 400 mM ammonium sulfate, 2 mM DTT, 50 μg/ml catalase, 50 μM ferrous ammonium sulfate. Solution B, a substrate solution, contained tryptophan, 6-MePH_4, 10 mM HCl, and 12 mM DTT. When using the conventional Aminco fluorometer, activity was measured by monitoring the emission at 330 nm when the sample was excited at 300 nm across the cuvette's 4-mm axis using an 8-nm emission band pass. When using the stopped-flow spectrophotometer the sample was excited at 300 nm along a 2-mm path and all emission perpendicular to the light source which passed a 305-nm short-wavelength cutoff filter was monitored. In either case the emission signal due to 5-hydroxytryptophan was calibrated by preparing a standard curve based on the change in fluorescence yield from complete enzymatic turnover of standardized solutions of tryptophan measured on the same instrument in the presence of the same concentration of 6-MePH_4.

Discontinuous Chromatographic Assay

The reaction conditions and protocol of initiation of the discontinuous HPLC assay were the same as those used in the routine assay (50–100 nM TRH, tryptophan, 6-MePH_4 in 50 mM Mes, pH 7.0, 200 mM ammonium sulfate, 7 mM DTT, 25 μg/ml catalase at 15°C). After initiation with tetrahydropterin, 100-μl samples were withdrawn from the reaction at 20 and 40 s and quenched with 10 μl of 40% trichloroacetic acid. The samples were then centrifuged at 12,000*g* for 10 min to pellet precipitated protein. The supernatant was injected onto a Rainin microsorb MV reverse-phase C18 HPLC column (50 mm × 4.6 mm i.d.) using a 10-μl injection loop. The mobile phase was 40 mM sodium acetate, pH 3.5, at a flow rate of 1 ml/min. A Waters 470 fluorescence detector with an excitation wavelength of 280 nm and an emission wavelength of 340 nm was used for detection. Under these conditions the retention times of tryptophan and 5-hydroxytryptophan were 3.6 and 1.4 min, respectively. Standard curves were generated based on the areas of peaks produced from standardized solutions of tryptophan and 5-hydroxytryptophan.

RESULTS AND DISCUSSION

Standard Assay

The tryptophan hydroxylase assays described here are based on the different spectral characteristics of tryptophan and 5-hydroxytryptophan. The addition of a hydroxyl moiety to tryptophan at the 5-position has the effect of broadening and shifting the UV absorbance transitions to longer wavelengths and markedly increasing the fluorescence (Fig. 1A). Maximum fluorescence signal from tryptophan or 5-hydroxytryptophan is obtained when either is excited at 278 nm. However, it is evident when comparing the absorbance spectrum of each compound that, in the presence of tryptophan, 5-hydroxytryptophan can be selectively excited between 300 and 320 nm (Fig. 1A). For example, upon excitation at 300 nm the total fluorescence yield of 5-hydroxytryptophan is 11.9-fold greater than that of tryptophan (Fig. 1A). This difference serves as the basis for the assay.

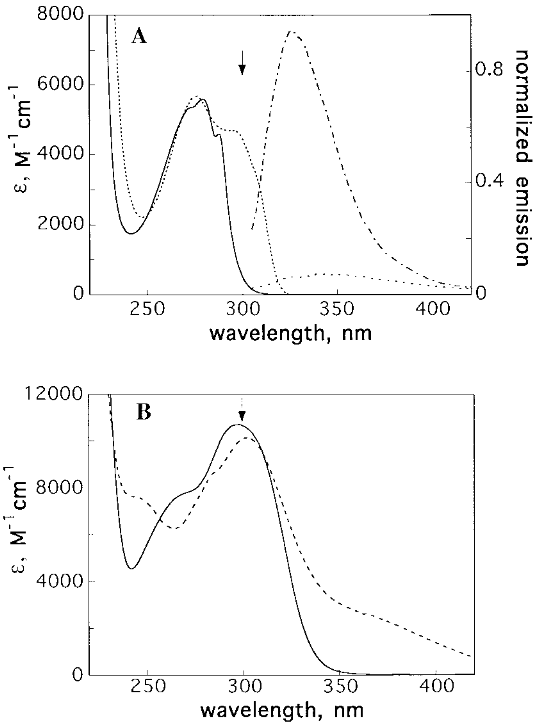

FIG. 1. Absorbance and emission spectra of assay substituents. (A) (—) Absorbance spectrum of tryptophan; (- - -) absorbance spectrum of 5-hydroxytryptophan; (– – –) normalized emission spectrum of tryptophan when excited at 300 nm; (– - –) normalized emission spectrum of 5-hydroxytryptophan when excited at 300 nm. (B) Pterin spectra: (—) tetrahydropterin and (- - -) quinonoid dihydropterin. Arrows in A and B indicate the 300-nm excitation wavelengths of the assay.

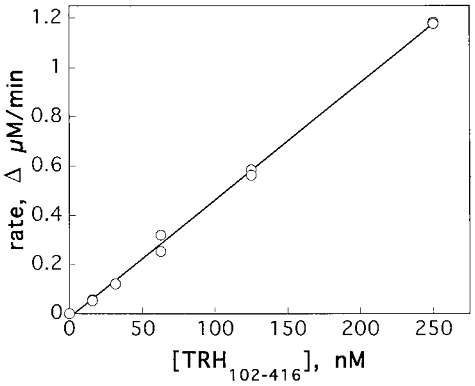

FIG. 2. Rate of 5-hydroxytryptophan formation as a function of protein concentration in the assay. The rates were calculated from linear fits to the data obtained in the first 60 s of turnover.

The assay is carried out at 15°C as TRH is stable at this temperature for extended periods. Stocks of tetrahydropterin are kept in 10 mM HCl until they are used to initiate the assay. This is done to protonate N5 and prevent autooxidation of the tetrahydropterin. Upon initiation of the reaction with tetrahydropterin, a linear increase in emission is observed for 60–100 s. The data obtained from fits to the first 60 s of turnover over a range of TRH concentrations (15–250 nM) indicate that the rate of the observed emission increase is directly proportional to the enzyme concentration (Fig. 2).

Each assay component, other than the enzyme and substrates, has a function intended to stabilize the enzyme and/or maximize the duration of product formation. Ferrous iron is required by TRH for activity. Ammonium sulfate is added to stabilize TRH during the assay. Catalase is added to consume any hydrogen peroxide formed upon autooxidation of the tetrahydropterin. A reductant, in this case DTT, is integral to the assay. The reductant maintains both the iron and the pterin in their reduced forms (Scheme 1). The quinonoid dihydropterin is the product of both tetrahydropterin autooxidation and hydrolysis of the C4a-hydroxypterin formed during TRH turnover. The reduction of this species by DTT serves a number of functions in the assay. First, reduction of the quinonoid dihydropterin ensures that the concentration of tetrahydropterin does not change significantly during enzyme turnover, allowing initial rate conditions to be maintained. Second, changes in the pterin spectra at the excitation wavelength are prevented, and the inner filter effect caused by the significant absorbance of the quinonoid across the emission wavelengths for 5-hydroxytryptophan is eliminated (Fig. 1B).

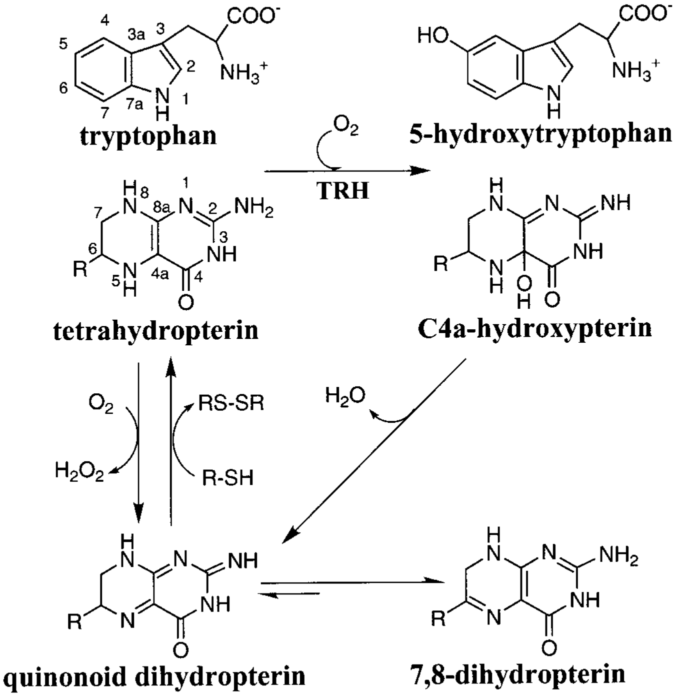

SCHEME 1

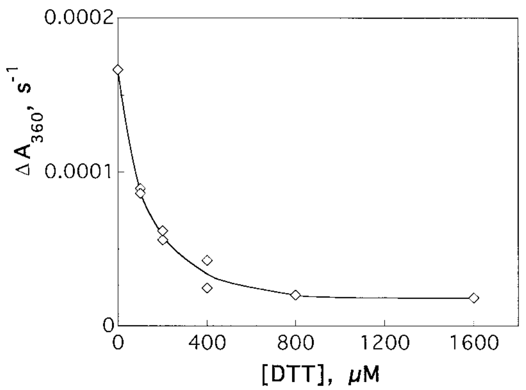

FIG. 3. Suppression of the rate of accumulation of quinonoid dihydropterin by dithiothreitol under the conditions of the continuous assay. Ammonium sulfate (200 mM), 7 mM DTT, 25 μg/ml catalase, 25 μM ferrous ammonium sulfate, 50 mM Mes, pH 7.0, with 320 μM oxygen was mixed in a 1:1 ratio with 6-methyltetrahydropterin (300 μM) and a specific concentration of DTT in 10 mM HCl. The change in absorbance at 360 nm describes the relative rate of autooxidation of 150 μM 6-methyltetrahydropterin at the DTT concentrations used. Rates were calculated from linear fits to the 360-nm absorbance increase observed in the first 120 s after mixing.

To establish the concentration of DTT required to suppress the accumulation of the quinonoid dihydropterin, 6-MePH_4 was mixed with various concentrations of DTT under the conditions of the assay. The rate of increase in absorbance at 360 nm was used as a measure of quinonoid dihydropterin accumulation. At the pH of the assay this wavelength is isosbestic for the conversion of the quinonoid dihydropterin to the 7,8-dihydropterin. The results are shown in Fig. 3. Essentially complete suppression of the quinonoid dihydropterin occurs at 1.5 mM DTT. A higher DTT concentration (7 mM) is recommended as a general condition since this concentration will remain in excess when using high pterin, oxygen, or enzyme concentrations.

Application to Steady-State Kinetic Analyses

For the determination of kinetic constants, turnover is initiated by mixing two solutions in a 1:1 ratio. This approach was chosen as it provides the most accurate means of obtaining the desired substrate concentrations. Solution A contains TRH, buffer, iron, and salt concentrations twice that of the desired reaction conditions (this has no adverse effect on TRH stability); solution B has the substrates tryptophan and tetrahydropterin at twice the final concentrations in dilute acid with DTT. The concentration of buffer in solution A is sufficient to ensure that, when mixed with solution B, the pH under which turnover occurs is not significantly different from that of solution A.

To establish the validity of the continuous assays, a Michaelis–Menten analysis was carried out using both a discontinuous HPLC assay and the continuous assay described here. The latter was done using both an Aminco fluorometer and an Applied Photophysics stopped-flow fluorometer. The data obtained from the standardized discontinuous HPLC assay are in complete agreement with those from the continuous fluorometric assays (Fig. 4).

When only the concentration of tryptophan is varied in a series of assays, the fixed tetrahydropterin absorbance reduces the fluorescence signal by attenuation of the incident light. This does not affect the measured rates provided the emission signal is standardized for 5-hydroxytryptophan at the tetrahydropterin concentration used. If, on the other hand, the concentration of tetrahydropterin is to be varied, the significant absorbance at 300 nm by the tetrahydropterin requires that the extent of attenuation of incident light be determined for each concentration of tetrahydropterin. The weak fluorescence of tryptophan when excited at 300 nm was used to quantitatively assess the attenuation. A linear relationship was found to exist between the tetrahydropterin concentration and the attenuation of the fluorescence signal ($R^2 = 0.988$). From this relationship a molar correction factor could be determined. Comparison of the data obtained at different excitation pathlengths on different instruments revealed that there is also a linear relationship ($R^2 = 0.988$) between the pathlength and the molar correction factor that is independent of the instrument used. Equation [1] was derived from these results. This equation can be used to correct initial rates measured on any fluorometer for the amount of incident light attenuation at any concentration of tetrahydropterin. Here, v_{cor} is the corrected activity, v_{obs} is the observed activity, l is the excitation pathlength in centimeters, and $[PH_4]$ is the molar concentration of 6-methyltetrahydropterin or tetrahydrobiopterin:

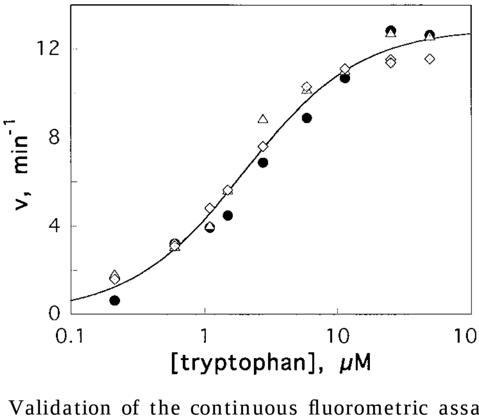

FIG. 4. Validation of the continuous fluorometric assay. Steady-state data obtained from a conventional fluorometer (◇) and that from the stopped-flow spectrofluorometer (△) compared to data obtained from a discontinuous fluorescence HPLC assay (●). The line is a fit of all the data to $v = V\,[TRP]/(K_{TRP} + [TRP])$.

$$v_{cor} = v_{obs}(1 + l(34{,}500\text{cm}^{-1}\text{M}^{-1}[PH_4])). \quad [1]$$

The assay as presented is readily adapted to a stopped-flow fluorometer. The use of stopped-flow methods for the kinetic analysis of TRH has a number of advantages. First, the dimensions of the flow cell are generally small enough to ensure economical use of enzyme stocks. Second, the mixing housing of the stopped-flow instrument can be equilibrated to a temperature at which the enzyme is stable for many hours, and the implementation of an anaerobic technique provides the means to easily and accurately control the concentration of oxygen in the assay.

The assay described here is the first continuous assay for tryptophan hydroxylase. The assay does not require specialized equipment or procedures and takes full account of the spectral characteristics of each assay component. In addition, the assay is sufficiently sensitive to permit the use of relatively low enzyme concentrations or the assessment of activity in enzyme fractions of low specific activity. Moreover, the assay can be applied to the determination of steady-state kinetic parameters for each of the three substrates of the enzyme.

ACKNOWLEDGMENTS

This work was supported in part by NIH Grant GM47291 and Robert A. Welch Foundation Grant A-1245.

REFERENCES

1. Friedman, P. A., Kappelman, H., and Kaufman, S. (1972) *J. Biol. Chem.* **247,** 4165–4173.
2. Tong, J. H., and Kaufman, S. (1975) *J. Biol. Chem.* **250,** 4152–4158.
3. Kuhn, D. M., Ruskin, B., and Lovenberg, W. (1980) *J. Biol. Chem.* **255,** 4137–4143.
4. Nakata, H., and Fujisawa, H. (1982) *Eur. J. Biochem.* **124,** 595–601.
5. Cash, C. D., Vayer, P., Mandel, P., and Maitre, M. (1985) *Eur. J. Biochem.* **149,** 239–245.
6. Vrana, K. E., Rucker, P. J., and Kumer, S. C. (1994) *Life Sci.* **55,** 1045–1052.
7. Yang, X.-J., and Kaufman, S. (1994) *Proc. Natl. Acad. Sci. USA* **91,** 6659–6663.
8. D'Sa, C. M., Arthur, R. E., States, J. C., and Kuhn, D. M. (1996) *J. Neurochem.* **67,** 900–906.
9. Mockus, S. M., Kumer, S. C., and Vrana, K. E. (1997) *J. Mol. Neurosci.* **9,** 35–48.
10. Haesegawa, H., and Ichiyama, A. (1987) *Methods Enzymol.* **142,** 88–92.
11. Vrana, S. L., Dworkin, S. I., and Vrana, K. E. (1993) *J. Neurosci. Methods* **48,** 123–129.
12. Johansen, P. A., Jennings, I., Cotton, R. G. H., and Kuhn, D. M. (1995) *J. Neurochem.* **65,** 882–888.
13. Iizuka, R., Ishida, J., Yoshitake, T., Nakamura, M., and Yamaguchi, M. (1996) *Biol. Pharm. Bull.* **19,** 762–764.
14. Moran, G. R., Daubner, S. C., and Fitzpatrick (1998) *J. Biol. Chem.* **273,** 12259–12266.
