# Supplementary material for: 5-Hydroxytryptophan (5-HTP): Natural Occurrence, Analysis, Biosynthesis, Biotechnology, Physiology and Toxicology
Source: Int J Mol Sci. 2020 Dec 26;22(1):181. doi: 10.3390/ijms22010181 (PMC7796270; doi:10.3390/ijms22010181)
Supplement: Supplementary file 1 [file ijms-22-00181-s001.zip › 5-HTP.Data/PDF/3249177030/Li-2020-Engineering-escherichia-coli-to-imp.pdf]

Synthetic and Systems Biotechnology 5 (2020) 200–205

Contents lists available at ScienceDirect

Synthetic and Systems Biotechnology

journal homepage: http://www.keaipublishing.com/synbio

Engineering *Escherichia coli* to improve tryptophan production via genetic manipulation of precursor and cofactor pathways

Zhu Li[a,b,c], Dongqin Ding[b,c,d], Huiying Wang[b,c], Linxia Liu[b,c], Huan Fang[b,c], Tao Chen[a], Dawei Zhang[b,c,d,*]

[a] *Key Laboratory of Systems Bioengineering of the Ministry of Education, School of Chemical Engineering and Technology, SynBio Research Platform, Collaborative Innovation Centre of Chemical Science and Engineering, Tianjin University, Tianjin, 300072, China*
[b] *Key Laboratory of Systems Microbial Biotechnology, Chinese Academy of Sciences, Tianjin, 300308, China*
[c] *Tianjin Institute of Industrial Biotechnology, Chinese Academy of Sciences, Tianjin, 300308, China*
[d] *University of Chinese Academy of Sciences, Beijing, 100049, China*

ARTICLE INFO

Keywords:
Escherichia coli
Tryptophan
Metabolic precursors
Cofactor supply

ABSTRACT

Optimizing the supply of biosynthetic precursors and cofactors is usually an effective metabolic strategy to improve the production of target compounds. Here, the combination of optimizing precursor synthesis and balancing cofactor metabolism was adopted to improve tryptophan production in *Escherichia coli*. First, glutamine synthesis was improved by expressing heterologous glutamine synthetase from *Bacillus subtilis* and *Bacillus megaterium* in the engineered *Escherichia coli* strain KW001, resulting in the best candidate strain TS-1. Then *icd* and *gdhA* were overexpressed in TS-1, which led to the accumulation of 1.060 g/L tryptophan. Subsequently, one more copy of *prs* was introduced on the chromosome to increase the flux of 5-phospho-α-D-ribose 1-diphosphate followed by the expression of mutated *serA* and *thrA* to increase the precursor supply of serine, resulting in the accumulation of 1.380 g/L tryptophan. Finally, to maintain cofactor balance, *sthA* and *pntAB,* encoding transhydrogenase, were overexpressed. With sufficient amounts of precursors and balanced cofactors, the engineered strain could produce 1.710 g/L tryptophan after 48 h of shake-flask fermentation, which was 2.76-times higher than the titer of the parent strain. Taken together, our results demonstrate that the combination of optimizing precursor supply and regulating cofactor metabolism is an effective approach for high-level production of tryptophan. Similar strategies could be applied to the production of other amino acids or related derivatives.

Introduction

Tryptophan, which is essential for mammals, is a critical aromatic amino acid that has been widely applied in food, pharmaceuticals, animal husbandry and other fields [1,2]. Consequently, there is a market in excess of 14,000 ton/year for L-tryptophan [3] and there is still a need for increased production. However, tryptophan extracted from capillus or synthesized from petrochemicals no longer satisfies the requirements of contemporary industries due to restrictions of environmental impact and raw materials [3]. As an alternative, microbial fermentation is becoming more prevalent because it is potentially eco-friendly and sustainable. In the past decades, *Corynebacterium glutamicum* [4,5] or yeast [6] were engineered by various biological strategies to produce tryptophan, but the engineered strains did not satisfy the industrial requirements due to the need for additives (such as indole and amino acids) during the fermentation and long fermentation cycle, which led to high cost. In later studies, researchers turned to *E. coli* due to its genetic tractability, robustness and fast reproduction. For instance, phosphate acetyltransferase (*pta*), a high affinity tryptophan transporter (*mtr*) and aromatic amino acid exporter (*yddG*) of *E. coli* were mutated, deleted and overexpressed, respectively, which resulted in 48.68 g/L tryptophan production after 48 h in 30 L fermenter [7]. Similarly, Gu et al. constructed recombinant *E. coli* by the separate or simultaneous knockout of *mtr, tnaB* and *aroP*. Compared to the parental strain, the recombinant strain's tryptophan production increased by 51.6% [8]. Although these engineered *E. coli* strains have been successfully constructed to produce tryptophan, the process of tryptophan biosynthesis is more complicated than that of other amino acids and is

Peer review under responsibility of KeAi Communications Co., Ltd.
* Corresponding author. Key Laboratory of Systems Microbial Biotechnology, Chinese Academy of Sciences, Tianjin, China.
E-mail addresses: lizh@tib.cas.cn (Z. Li), ding_dq@tib.cas.cn (D. Ding), wanghuiying@tib.cas.cn (H. Wang), liulinxia@tib.cas.cn (L. Liu), fang_h@tib.cas.cn (H. Fang), chentao@tju.edu.cn (T. Chen), zhang_dw@tib.cas.cn (D. Zhang).

https://doi.org/10.1016/j.synbio.2020.06.009
Received 27 April 2020; Received in revised form 23 June 2020; Accepted 23 June 2020
2405-805X/ © 2020 The Author(s). Production and hosting by Elsevier B.V. on behalf of KeAi Communications Co., Ltd. This is an open access article under the CC BY license (http://creativecommons.org/licenses/BY/4.0/).

subject to diverse regulation mechanisms including repression, attenuation, feedback inhibition and feed-forward regulation [9]. Therefore, engineering *E. coli* to construct industrial strains remains a difficult challenge.

In previous studies, optimizing precursor synthesis was considered as an important strategy for improving the yield of target metabolites. For example, phosphoenolpyruvate (PEP) and erythrose 4-phosphate (E4P), respectively derived from the glycolysis pathway (EMP) and the pentose phosphate (PP) pathway, are two essential precursors for tryptophan synthesis. There are two strategies for augmenting the PEP pool. One strategy relies on the overexpression of *ppsA* [10,11], knockdown of *pykAF* [12] and knockout of *ppc* [13]. This can increase the intracellular pool of PEP and strengthen the metabolic flux through the shikimate pathway, which is essential for aromatic amino acid synthesis. The other strategy is the modification of the phosphotransferase system (PTS). According to prior investigations, PTS modification, including attenuation, replacement and knockout, is also an important means to improve the intracellular PEP levels [12,14,15]. For instance, Chen et al. engineered *E. coli* to produce tryptophan via PTS modification that contributed to the tryptophan production of 39.7 g/L [15]. Furthermore, the increase of E4P levels can significantly improve the flux through the shikimate pathway. A common strategy for increasing the E4P content is PP pathway modification, especially the *tktA* trim. For example, overexpression of *tktA* was usually conducted in *E. coli* to improve the yield of tryptophan [11,16]. In a recent study, Liu et al. introduced a heterologous phosphoketolase pathway into yeast to enhance E4P synthesis [17], a strategy that may be suited for *E. coli* as well. It has therefore been established in the literature that the optimization of PEP and E4P synthesis can improve tryptophan production [18], but the synthesis of another three precursors, glutamine, serine and 5-phospho-α-D-ribose 1-diphosphate (PRPP), was rarely optimized. These three precursors are also necessary for tryptophan synthesis in *E. coli*. In this work, we systematically optimized the biosynthesis of serine, glutamine and PRPP to further increase tryptophan production.

Cofactor regulation also plays a vital role in constructing a highly efficient cell factory. In the past decades, a number of studies focused on NADH and NADPH. Their interconversion is regulated by soluble pyridine nucleotide transhydrogenase (SthA) and membrane-bound transhydrogenase (PntAB). Many studies have demonstrated that imbalance of reducing equivalent is usually the limiting factor for obtaining the maximum yield of target fermentation products. For example, activating a soluble form of transhydrogenase SthA in *E. coli* enable the efficient synthesis of succinate [19]. Similarly, Luo et al. engineered *sthA* by regulating its expression level for the efficient synthesis of 2-pyrone-4,6-dicarboxylic acid (a dicarboxylic acid with a polar pseudo-aromatic moiety) in *E. coli* [20]. Additionally, the overexpression of *pntAB* improved the production of polyhydroxyalkanoates [21]. Wu et al. engineered *Corynebacterium glutamicum* to produce lysine (27.1 g/L) via a combination of engineering cofactor metabolism and optimizing the lysine synthesis pathway [22]. The precursor pathways relevant to tryptophan synthesis also require NADH and NADPH (Fig. 1). When these precursor pathways are modified, it may cause a redox imbalance and impact tryptophan production. However, the regulation of cofactors to increase the production of tryptophan in *E. coli* was rarely studied.

In this study, a combination of precursor pathway modification and cofactor regulation was applied to improve tryptophan production in *E. coli*. We selected the engineered *E. coli* KW001 as a chassis cell [15], since this strain could better produce tryptophan. Firstly, heterologous glutamine synthetase genes from *Bacillus subtilis* and *Bacillus megaterium* were separately introduced into the KW001 strain to optimize glutamine synthesis. Then *icd* and *gdhA* genes were overexpressed via the insertion at the *poxB* and *ackA* loci on the chromosome to further enhance glutamine synthesis. Secondly, an additional copy of *prs* was introduced on the chromosome to increase the flux of PRPP. Subsequently, mutated *serA* and *thrA* were expressed to enhance the serine supply. Finally, *sthA* and *pntAB* were co-overexpressed using strong promoters to maintain cofactor balance. Compared with the KW001 strain, the final strain's tryptophan production increased 2.76-fold (Fig. 1). The integrated strategy presented here can offer a reference for constructing efficient strains for the overproduction of tryptophan meeting the needs of industry.

Materials and methods

Reagents

Taq PCR Master Mix and DNA ladder were purchased from Tiangen (China). DNA was purified using a Universal DNA Purification Kit (Tiangen, China). Plasmids were extracted using the Mini Plasmid Kit (Tiangen, China). All primers (Supplemental Table S1) were synthesized by QINGKE (Beijing, China). O-phthalaldehyde (OPA) was purchased from Agilent (China). Other chemical reagents were purchased from Yuanye Biotech (China).

Genetic methods

The pRed_Cas9_recA [23] based on the CRISPR-CAS system [24] is a plasmid tool for scarless genomic editing. In this study, all genomic editing, including deletion, insertion and mutation, used this plasmid.

Construction of plasmids and strains

All strains and plasmids used in this study are listed in Table 1. *E. coli* KW001 [15] was used as the starting strain for engineering. *E. coli* DH5α was used for cloning and plasmid propagation.

Culture conditions

In this study, lysogeny broth (LB) and fermentation medium were used. Liquid LB contained 1% (w/v) tryptone, 0.5% (w/v) yeast extract, and 1% (w/v) NaCl. If solid LB plates were needed, XY % (w/v) agar was added. The shake-flask fermentation medium was composed of glucose (30 g/L), $MgSO_4{\cdot}7H_2O$ (0.5 g/L), KH_2PO_4 (3 g/L), K_2HPO_4 (12 g/L), $(NH_4)_2SO_4$ (4 g/L), yeast extract (1 g/L), monosodium citrate (2 g/L), and $FeSO_4{\cdot}7H_2O$ (0.1 mg/mL). Ampicillin and tetracycline were used at a concentration of 100 mg/L where indicated.

DH5α cells were grown at 30 or 37 °C and 220 rpm in test-tubes containing 5 mL of LB. When the pXlltS plasmid (Table 1) was constructed, DH5α was incubated at 37 °C in LB with tetracycline. When the Cas9 series plasmids (Table 1) were constructed, DH5α was incubated at 30 °C in LB with ampicillin. The process of Cas9 genome editing in *E. coli* was done as described before [23].

For the fermentations, an aliquot comprising 5 μl of cryopreserved *E. coli* cells was used to inoculate a test-tube with 5 ml LB medium, which was shaken at 220 rpm for 12 h. Then, the resulting seed culture was used to inoculate a 250 ml shake flask with 20 ml of fermentation medium to an OD_{600} of 0.1, and cultivated for 48 h.

Analytical techniques

The concentrations of the amino acids tryptophan, serine, glutamate and glutamine were determined using high-performance liquid chromatography (HPLC) with UV detection. Samples collected from shake flasks were centrifuged. Amino acid concentrations were determined via HPLC according to the instruction of Zorbax Eclipse-AAA columns on an Agilent 1100 HPLC. This column could achieve the resolution ratio at approximately 10 pmols. Derivatization was performed according to the manufacturer's protocol. Tryptophan, serine, glutamate and glutamine were separated by using mobile phase A (40 mM Na_2HPO_4 pH 7.8) and phase B (Acetonitrile (ACN):

MeOH: H_2O = 45:45:10) with gradient elution (The gradient

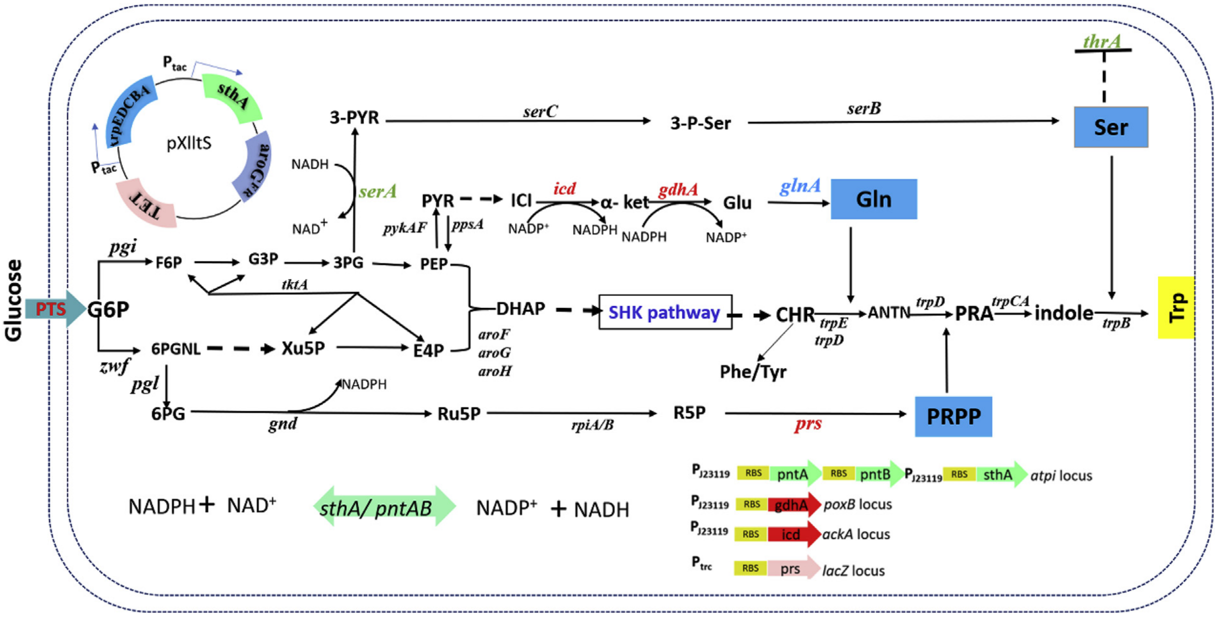

Fig. 1. Biosynthetic pathway of tryptophan from glucose and the metabolic engineering strategies used in this study. Abbreviations: *pgi*, glucose-6-phosphate isomerase; *zwf*, glucose-6-phosphate 1-dehydrogenase; *pgl*, 6-phosphogluconolactonase; *tktA*, transketolase I; *gnd*: 6-phosphogluconate dehydrogenase; *rpiA*, ribose-5-phosphate isomerase A; *prs*, ribose-phosphate diphosphokinase; *aroF*, *aroG* and *aroH*: 3-deoxy-7-phosphoheptulonate synthase; *pykAF:* pyruvate kinases I and II; *ppsA*, PEP synthetase; *serA*, phosphoglycerate dehydrogenase; *serC*, phosphoserine/phosphohydroxythreonine aminotransferase; *serB*, phosphoserine phosphatase; *icd*, isocitrate dehydrogenase; *gdhA*, glutamate dehydrogenase; *glnA*; glutamine synthetase; *sthA*, soluble pyridine nucleotide transhydrogenase; *pntAB*, pyridine nucleotide transhydrogenase; *thrA*, fused aspartate kinase/homoserine dehydrogenase 1; *trpD* and *trpE*, anthranilate synthase; *trpC*, fused indole-3-glycerol phosphate synthase/phosphoribosyl anthranilate isomerase; *trpB*, tryptophan synthase subunit beta; *trpA*, tryptophan synthase subunit alpha. G6P, glucose-6-phosphate; F6P, fructose-6-phosphate; 6PGNL, 6-phosphoglucono-lactone, G3P, glyceraldehyde-3-phosphate; 3 PG, glycerate-3-phosphate; 3-PYR, 3-phosphonooxypruvate; PEP, phosphoenolpyruvate; Xu5P, xylulose-5-phosphat;E4P, erythrose-4-phosphate; Ru5P, ribulose-5-phosphate; R5P, ribose-5-phosphate; PRPP, phosphoribosyl pyrophosphate; PYR, pyruvate; ICI, isocitrate; α-ket; α-ketoglutarate; Glu, glutamate; Gln, glutamine; 3-P-Ser, 3-phosphoserine; Ser, serine; DHAP, 3-deoxy-d-arabinoheptulosonate-7-phosphate; CHR, chorismate; ANTN, anthranilate; PRA, N-(5′-phosphoribosyl)-anthranilate; Trp, tryptophan. Overexpressed genes are marked in red, expressed heterologous genes are marked in blue and mutated genes are marked in green. RBS: ribosome bind site, whose sequence is AAGGAGATATA. P_{J23119}: J23119 promoter (http://parts.igem.org/Part:BBa_J23119), whose sequence is TTGACAGCTAGCTCAGTCCTAGGTATAATGCTAGC.

Table 1
Plasmids and strains used in this study.

Name	Description	Reference
pCas9-Red	*Exo*, *bet*, *gam*, arabinose operon, Cas9, sgRNA, Amp^r	[23]
PH5a1	Derived from p15A, incomplete *lacI*, tet^r, $aroG^{fr}$, $trpE^{fr}DCBA$	[15]
pXIltS	Derived from PH5a1, incomplete *lacI*, tet^r, $aroG^{fr}$, $trpE^{fr}DCBA$, *sthA*	this study
pCas9-bsu-*glnA*	Derived from pCas9-Red, *BSglnA* with about 300bp homologous arms, sgRNA with specific N20 sequence (AGAAGCGAAAGAGATCCCAC).	this study
pCas9-bmq-*glnA*	Derived from pCas9-Red, *BMglnA* with about 300bp homologous arms, sgRNA specific with N20 sequence (AGAAGCGAAAGAGATCCCAC).	this study
pCas9-bsu-*glnA-159*	Derived from pCas9-bsu-*glnA*,	this study
pCas9-bsu-*glnA-304*	Derived from pCas9-bsu-*glnA*	this study
pCas9-*gdhA*	Derived from pCas9-Red, *gdhA* (*poxB* locus) with about 300bp homologous arms, sgRNA with specific specific N20 sequence (CCTATTGTTCATGCCCTGCG).	this study
pCas9-*icd*	Derived from pCas9-Red, *icd* (*ackA* locus) with about 300bp homologous arms, sgRNA with N20 sequence (CAGCCACTTCTATGTAACCC).	this study
pCas9-$serA^{mut}$	Derived from pCas9-Red, *serAmut* with about 300bp homologous arms, sgRNA with specific N20 sequence (TCCGTCCACCAAAAATATGA).	this study
pCas9-$thrA^{mut}$	Derived from pCas9-Red, $thrA^{mut}$ with about 300bp homologous arms, sgRNA with specific N20 sequence (GTCACGCGCCCGTATTTCCG).	this study
pCas9-*prs*	Derived from pCas9-Red, *prs* (*lacz* locus) with about 300bp homologous arms, sgRNA with specific N20 sequence (ATTATCCGAACCATCCGCTG).	this study
pCas9-*sthA-pntAB*	Derived from pCas9-Red, *sthA-pntAB* (*atpi* locus) with about 300bp homologous arms, sgRNA with specific N20 sequence (CAGGCGCATACACCAGCGAA).	this study
DH5α	Host for cloning and plasmid construction	lab stock
KW001	Starting strain for engineering, Tet^r,with PH5a1 .	[15]
TS-1	Derived from KW001, *BSglnA* replacing the native *glnA*	this study
TS-2	Derived from KW001, *BMglnA* replacing the native *glnA*	this study
TS-3	Derived from KW001, *BSglnA*-L159I replacing the native *glnA*	this study
TS-4	Derived from KW001, *BSglnA*-E304A replacing the native *glnA*	this study
TS-5	Derived from TS-1,::*icd*-::*gdhA* with J23119 promoter	this study
TS-51	Derived from TS-1.::*icd* with J23119 promoter	this study
TS-52	Derived from TS-1.::*gdhA* with J23119 promoter	this study
TS-6	Derived from TS-5,::*prs* with trc promoter	this study
TS-7	Derived from TS-6, $serA^{mut}$	this study
TS-8	Derived from TS-7, $serA^{mut}$-$thrA^{mut}$	this study
TS-9	Derived from TS-8,::*sthA-pntAB* with J23119 promoter	this study
TS-10	Derived from TS-9, pXIltS	this study

variation ratio of phases A:B with time was shown in Supplemental Table S2). Column temperature was set to 30 °C, the flow rate was 2 mL/min and the measure wavelength was 338 nm. One sample was quantitatively analyzed within 30 min. All experiments were conducted in triplicate.

Glucose, acetate and α-ketoglutarate were measured using an Aminex HPX-87H column (300 × 7.8 mm column) (Bio-Rad, Hercules, USA) which was maintained at 50 °C and the elution was monitored at 210 nm. The mobile phase was composed of 5 mM H_2SO_4, with a flow rate of 0.5 ml/min.

Results and discussion

Engineering the glutamine synthesis pathway

Glutamine is one of the important precursors for tryptophan synthesis (Fig. 1). In *E. coli*, anthranilate synthase encoded by *trpE* and *trpD* transforms chorismate into anthranilate, which requires glutamine as an amine donor for the reaction. In this study, the glutamine biosynthesis pathway was optimized by overexpressing *icd* and *gdhA*, as well as a heterologous *Bacillus subtilis glnA* (*BSglnA*) in *E. coli* KW001.

Glutamine synthase (GlnAs) is usually susceptible to the concentration of ammonium ions in *E. coli*. At high ammonium concentrations, GlnA adenylation leads to a loss of activity [25]. However, since homologous enzymes from Gram-positive bacteria are insensitive to ammonium ions [26], GlnAs from *Bacillus subtilis* (BS) and *Bacillus megaterium* (BM) was separately introduced into KW001 to replace the native enzyme, resulting in the strains TS-1 (*BSglnA*) and TS-2 (*BMglnA*). As shown in Fig. 2A, the heterologous genes could be successfully expressed in *E. coli*. The TS-1 strain produced 0.810 g/L tryptophan, while there was no change of production in TS-2 compared with the KW001 strain (0.620 g/L). Unexpectedly, approximately 0.798 g/L acetate (Table 2) was also produced by TS-1 strain, which resulted in waste of carbon. In addition, the glutamine and glutamate concentrations were examined. However, the two compounds were not detected in the fermentation broth of TS-1 and KW001, indicating that glutamine was consumed at the same rate as it was generated. Subsequently, two improved mutants of *BSglnA*, L159I and E304A [27], which are insensitive to end-product inhibition, were respectively introduced into TS-1 to construct the strains TS-3(L159I) and TS-4 (E304A). Regrettably, the product titers of the strains TS-3 and TS-4 were practically unchanged compared with TS-1 (p-value > 0.05), as shown in Fig. 2A. In *E. coli*, GlnAs is regulated by several mechanisms such as cumulative feedback inhibition by multiple end-products of glutamine metabolism, and dynamic interconversion of the enzyme between adenylation and de-adenylation [28]. Thus, we speculated that the introduction of BSGlnAs into *E. coli* might partially release cumulative feedback inhibition and increase the production of tryptophan in

Table 2
Analysis of key metabolites in the engineered strains constructed in this study.

Strain	Metabolite titer at 48 h		
	α-ket (g/L)	Acetate (g/L)	Tryptophan (g/L)
KW001	0.261±0.080	0.835±0.070	0.620± 0.070
TS-1	0.362± 0.060	0.798±0.030	0.810± 0.060
TS-5	1.492±0.210	0.185±0.050	1.060±0.100
TS-51	0.671±0.120	0.340±0.060	0.760 ± 0.120
TS-52	0.281±0.070	0.786±0.040	0.750 ± 0.090
TS-6	1.242±0.060	0.138±0.050	1.220 ± 0.120
TS-7	1.241±0.070	0.267±0.060	1.040 ± 0.120
TS-8	1.090±0.170	0.138±0.050	1.380±0.100
TS-9	1.248±0.770	0.124±0.030	1.520±0.080
TS-10	1.104±0.070	0.076± 0.060	1.710±0.100

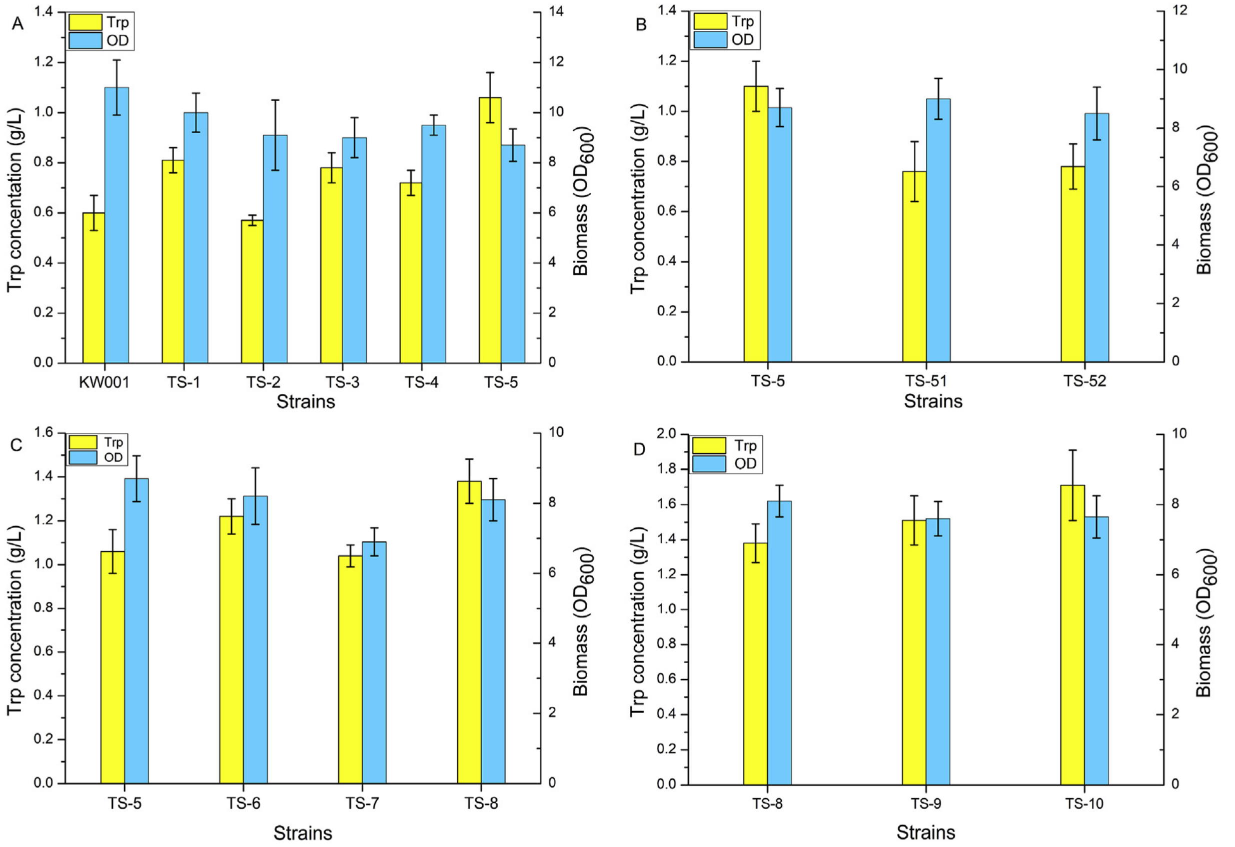

Fig. 2. Fermentation results of the strains. (A) KW001, TS-1, TS-2, TS-3, TS-4 and TS-5. (B) TS-5, TS-51 and TS-52. (C) TS-5, TS-6, TS-7and TS-8. (D) TS-8, TS-9 and TS-10. Yellow box, Trp concentration (g/L); blue box, biomass (OD_{600}). Error bars indicate the standard deviations from three independent cultures.

the TS-1 strain.

Additionally, the co-overexpression of *icd* (encoding *isocitrate dehydrogenase*) and *gdhA* (encoding *glutamate dehydrogenase*) from the chromosome was conducted to further enhance the synthesis of glutamine. The *poxB* locus (*gdhA*) and *ackA* locus (*icd*) were selected as insertion site. The enzymes encoded by *gdhA* and *icd* constitute a bridge between carbon metabolism and nitrogen metabolism. Moreover, *poxB* and *ackA* were selected as insertion sites because they are related to acetic acid metabolism, and previous investigations have demonstrated that *ackA* inactivation was favorable to tryptophan biosynthesis [29]. Consequently, *gdhA* and *icd* were co-overexpressed in the TS-1 strain to construct the TS-5 strain. As shown in Table 2 and Supplementary Fig. 2, TS-5 could produce 1.060 g/L tryptophan, and its conversion rate increased to 4.1%, illustrating that the combination of reinforcing nitrogen metabolism and expressing *BSglnA* was an effective strategy. A reason for the improvement of tryptophan production might be that the intracellular ratio of α-ketoglutarate/glutamine (K/G) increases. According to a previous study, high activity of GlnAs led to a high ratio of K/G [30]. As shown in Table 2, the TS-5 strain could accumulate 1.492 g/L α-ketoglutarate, representing a six-fold increase over the parental strain, indicating that the rise of K/G was indeed favorable to glutamine synthesis and increased tryptophan production. Moreover, the acetate titer of strain TS-5 (0.185 g/L) decreased by 4.3 times compared to strain TS-1 (0.798 g/L), likely because of the *ackA* deletion. To investigate this, we individually overexpressed *icd* and *gdhA* in the TS-1 strain, resulting in the strains TS-51 (the deletion of *icd*) and TS-52 (the deletion of *gdhA*). Their fermentation results are shown in Fig. 2B. The acetate content in the fermentation broth of TS- 51 (0.340 g/L) decreased compared to the TS-1strain (0.798 g/L) (Table 2), which also illustrated that *ackA* inactivation made a difference. This was consistent with a previous report by Liu et al. [29]. However, *poxB* was reported to be dispensable for acetate synthesis under anaerobic fermentation conditions [31]. Consequently, the deletion of *poxB* had little impact on acetate synthesis. As shown in Table 2, the acetate titer of the strain TS-52 was the same as that of TS-1. The individual overexpression of *icd* and *gdhA* had no effect on tryptophan production (Fig. 2B). As shown in Supplementary Fig. 1 A and B, the growth of strain TS-5 was faster than that of KW001 in the early stages of the fermentation, but the biomass of KW001 was higher than that of TS-5 at the end of fermentation. The enhancement of nitrogen metabolism may facilitate cell growth and contribute to tryptophan synthesis.

Optimizing the synthetic pathways of PRPP and serine

PRPP and serine are also two essential precursors for tryptophan synthesis (Fig. 1). PRPP and anthranilate are condensed to N-(5′-phosphoribosyl)-anthranilate (PRA) by anthranilate synthase (encoded by *trpD*), while serine and indole can be condensed to form tryptophan by tryptophan synthase subunit beta (encoded by *trpB*). As a result, the optimization of PRPP and serine biosynthesis may be advantageous for tryptophan synthesis. For example, Fan et al. showed that co-expression of *zwf, gnd* and *prs* in *E. coli* CYT15 could optimize PRPP biosynthesis and improve cytidine production [32] due to PRPP being a precursor for cytidine synthesis. More importantly, the increase of tryptophan production via the overexpression of *prs* has been demonstrated by Trondle et al. using metabolic omics methods [33]. Furthermore, a strain with an engineered serine biosynthesis pathway was able to effectively synthesize the precursor serine for tryptophan production based on metabolic analysis [34]. To confirm that the optimization of PRPP and serine biosynthesis would lead to an improvement of tryptophan production, we constructed three strains as shown below.

Firstly, to optimize PRPP biosynthesis, *prs* was overexpressed from the chromosome of strain TS-5 (*atpi* locus) using the strong Ptrc promoter, resulting in strain TS-6. As shown in Fig. 2 C, the tryptophan yield of TS-6 was 1.220 g/L after 48 h of shake flask fermentation. Compared with the TS-5 strain, its production increased by 15%. This result indicated that the overexpression of *prs* was an effective strategy for increasing tryptophan synthesis, which was consistent with a previous report by Trondle et al. [33]. Secondly, serine biosynthesis was optimized via the modification of *serA* and *thrA*. A *serA* mutant (H344A, N346A, N364A) [35],which released feedback inhibition from serine, was introduced into the TS-6 strain, forming the strain TS-7. Then, a *thrA* variant with two known site-directed mutations (S357R and Y356C) [36], which could release the inhibition of the threonine synthesis pathway from serine and increase the strain's tolerance toward serine, was introduced into the TS-7 strain, generating strain TS-8. As shown in Fig. 2 C, we found that the biomass of TS-7 was slightly decreased compared to that of TS-6 (p-value = 0.065 > 0.05). However, when the mutant *thrA* was introduced into strain TS-7, its growth was recovered. The strain TS-8 could produce 1.380 g/L tryptophan, representing a 5.4% conversion rate (Supplementary Fig. 2). Additionally, 0.050 g/L serine was detected in the fermentation broth of strain TS-8, while none was detected in that of strain TS-6, indicating that only the combined modification of *thrA* and *serA* was effective. As shown in Supplementary Fig. 1 A and B, the cell growth rate of strains TS-8 and TS-5 was similar, but the biomass of strain TS-5 was higher than that of TS-8, possibly due to an alleviation of the metabolic burden. According to these results, the optimization of serine and PRPP biosynthesis was an effective strategy for the improvement of tryptophan production.

The metabolic regulation of NADH and NADPH

In order to further optimize tryptophan synthesis, it is necessary to regard the cofactor regulation. As shown in Fig. 1, the biosynthesis of PRPP, glutamine and serine requires cofactor participation. When these precursor pathways were modified, the changes will also have an unavoidable influence on the cofactor metabolism. Thus, to balance cofactor metabolism, we selected the two transhydrogenases SthA and PntAB that are known to interconvert NADH and NADPH [37,38].

To balance the metabolism between NADH and NADPH, we decided to overexpress *pntAB* and *sthA* in strain TS -8 by inserting them into lacZ locus on the chromosome, generating the strain TS-9. When the TS-9 strain was cultured for 48 h in shake flasks, 1.510 g/L tryptophan was detected, as shown in Fig. 2 D. When we optimized PRPP biosynthesis, the PP pathway was enhanced, which lead to increased NADPH production. It should be noted that SthA preferentially transforms NADPH to NADH under metabolic conditions with excess NADPH [38]. We hypothesized that tryptophan production could be further improved if the expression of *sthA* was further enhanced. To prove this conjecture, the plasmid pXlltS derived from pHa1 (Table 1) was introduced into TS-9, generating the strain TS-10. Remarkably, the strain TS-10 produced 1.710 g/L tryptophan with only 0.076 g/L acetate, as shown in Fig. 2 D and Table 2. The TS-10 strain was further characterized, as shown in Supplementary Fig. 1 A. The biomass of strain TS-10 was apparently decreased, but its conversion rate increased by 4.7% compared to the starting strain KW001 (Supplementary Fig. 2).

Conclusions

In the previous work, Chen et al. engineered KW001 strain via the increase of PEP supply by replacing PTS system and the decrease of acetate accumulation to improve the production of tryptophan. Their work, however, did not devote to the optimization of several other crucial precursors, such as glutamine, serine and PRPP. In this study, we developed a strategy based on the combined optimization of these three precursors biosynthesis and cofactor metabolism, which effectively improved tryptophan production in the engineered strains derived from KW001. On the one hand, glutamine, serine and PRPP biosynthesis was optimized by key genetic manipulations to improve tryptophan production. On the other hand, the overexpression of two transhydrogenases balanced the cofactor metabolism, which further increased

tryptophan production. The final engineered strain could produce 1.710 g/L tryptophan with a conversion rate of 6.7% after shake-flask fermentation for 48 h. Compared to it of KW001 strain, tryptophan production of TS-10 strain increased 2.76 times, representing that tryptophan conversion rate increased by 4.7%. Overall, the combined strategy based on optimization of precursor synthesis and regulation of cofactor metabolism is a good way to promote tryptophan synthesis, and can offer a viable approach for the construction of industrial strains. In future study, we could consider the combined utilization of our modified strategies and Chen et al.'s them, expecting the further improvement of production of tryptophan.

Declaration of competing interestCOI

The authors declare that they have no conflicts of interest.

CRediT authorship contribution statement

Zhu Li: Writing - original draft, Data curation. **Dongqin Ding:** Writing - review & editing. **Huiying Wang:** Writing - review & editing. **Linxia Liu:** Writing - review & editing. **Huan Fang:** Writing - review & editing. **Tao Chen:** Supervision, Methodology. **Dawei Zhang:** Supervision, Methodology.

Acknowledgements

This work was supported by the National Key R&D Program of China (2018YFA0900300), the Tianjin Science Fund for Distinguished Young Scholars (17JCJQJC45300), and the Science and Technology Service Network (STS) Initiative of the Chinese Academy of Sciences (CAS) (KFJ-STS-ZDTP-065).

Appendix A. Supplementary data

Supplementary data to this article can be found online at https://doi.org/10.1016/j.synbio.2020.06.009.

References

[1] Won-Gi Bang SL, Hermann Sahm T, Fritz Wagner. Production of L-tryptophan by Escherichia coli cells. Cell 1983;XXV:999–1011.

[2] Zhao ZJ, Zou C, Zhu YX, Dai J, Chen S, Wu D, et al. Development of L-tryptophan production strains by defined genetic modification in Escherichia coli. J Ind Microbiol Biotechnol 2011;38:1921–9.

[3] Alberto Rodriguez JAM, Flores Noemí, Escalante Adelfo, Gosset Guillermo, Bolivar Francisco. Engineering Escherichia coli to overproduce aromatic amino acids and derived compounds. Microb Cell Factories 2014;13.

[4] Ikeda M, Katsumata R. Tryptophan production by transport mutants of Corynebacterium glutamicum. Biosci Biotechnol Biochem 1995;59:1600–2.

[5] Katsumata2 MIAR. Hyperproduction of tryptophan by Corynebacterium glutamicum with the modified pentose phosphate pathway. Appl Environ Microbiol 1999:2497–502.

[6] Suastegui M, Shao Z. Yeast factories for the production of aromatic compounds: from building blocks to plant secondary metabolites. J Ind Microbiol Biotechnol 2016;43:1611–24.

[7] Wang J, Cheng LK, Wang J, Liu Q, Shen T, Chen N. Genetic engineering of Escherichia coli to enhance production of L-tryptophan. Appl Microbiol Biotechnol 2013;97:7587–96.

[8] Gu P, Yang F, Li F, Liang Q, Qi Q. Knocking out analysis of tryptophan permeases in Escherichia coli for improving L-tryptophan production. Appl Microbiol Biotechnol 2013;97:6677–83.

[9] Chen L, Chen M, Ma C, Zeng A-P. Discovery of feed-forward regulation in L-tryptophan biosynthesis and its use in metabolic engineering of E. coli for efficient tryptophan bioproduction. Metab Eng 2018;47:434–44.

[10] Liu X, Lin J, Hu H, Zhou B, Zhu B. Site-specific integration and constitutive expression of key genes into Escherichia coli chromosome increases shikimic acid yields. Enzym Microb Technol 2016;82:96–104.

[11] Shen T, Liu Q, Xie X, Xu Q, Chen N. Improved production of tryptophan in genetically engineered Escherichia coli with TktA and PpsA overexpression. J Biomed Biotechnol 2012:605219. 2012.

[12] Liu L, Chen S, Wu J. Phosphoenolpyruvate:glucose phosphotransferase system modification increases the conversion rate during L-tryptophan production in Escherichia coli. J Ind Microbiol Biotechnol 2017;44:1385–95.

[13] Lee MY, Hung WP, Tsai SH. Improvement of shikimic acid production in Escherichia coli with growth phase-dependent regulation in the biosynthetic pathway from glycerol. World J Microbiol Biotechnol 2017;33:25.

[14] Liu Y, Xu Y, Ding D, Wen J, Zhu B, Zhang D. Genetic engineering of Escherichia coli to improve L-phenylalanine production. BMC Biotechnol 2018;18:5.

[15] Chen Y, Liu Y, Ding D, Cong L, Zhang D. Rational design and analysis of an Escherichia coli strain for high-efficiency tryptophan production. J Ind Microbiol Biotechnol 2018;45:357–67.

[16] Gu P, Yang F, Kang J, Wang Q, Qi Q. One-step of tryptophan attenuator inactivation and promoter swapping to improve the production of L-tryptophan in Escherichia coli. Microb Cell Factories 2012;11:30.

[17] Liu Q, Yu T, Li X, Chen Y, Campbell K, Nielsen J, et al. Rewiring carbon metabolism in yeast for high level production of aromatic chemicals. Nat Commun 2019;10:4976.

[18] Niu H, Li R, Liang Q, Qi Q, Li Q, Gu P. Metabolic engineering for improving L-tryptophan production in Escherichia coli. J Ind Microbiol Biotechnol 2019;46:55–65.

[19] Zhu X, Tan Z, Xu H, Chen J, Tang J, Zhang X. Metabolic evolution of two reducing equivalent-conserving pathways for high-yield succinate production in Escherichia coli. Metab Eng 2014;24:87–96.

[20] Luo ZW, Kim WJ, Lee SY. Metabolic engineering of Escherichia coli for efficient production of 2-Pyrone-4,6-dicarboxylic acid from glucose. ACS Synth Biol 2018;7:2296–307.

[21] Jung HR, Yang SY, Moon YM, Choi TR, Song HS, Bhatia SK, et al. Construction of efficient platform Escherichia coli strains for polyhydroxyalkanoate production by engineering branched pathway. Polymers (Basel) 2019;11.

[22] Wu W, Zhang Y, Liu D, Chen Z. Efficient mining of natural NADH-utilizing dehydrogenases enables systematic cofactor engineering of lysine synthesis pathway of. Metab Eng 2019;52:77–86.

[23] Zhao D, Yuan S, Xiong B, Sun H, Ye L, Li J, et al. Development of a fast and easy method for Escherichia coli genome editing with CRISPR/Cas9. Microb Cell Factories 2016;15:205.

[24] 1,1,4,2,3,2,3,1 Jiang Wenyan, Bikard David, Cox David, Feng Zhang, Marraffini Luciano A. RNA-guided editing of bacterial genomes using crisPr-cas systemsrNA-guided editing of bacterial genomes using crisPr-cas systems. Nat Biotechnol 2013;31:232–41.

[25] Mangum JH, Magni G, Stadtman ER. Regulation of glutamine synthetase adenylylation and deadenylylation by the enzymatic uridylylation and deuridylylation of the PII regulatory protein. Arch Biochem Biophys 1973;158(2):514–25.

[26] Susan H, Fisher JLBa, Lewis V, Wray J. Mutations in Bacillus subtilis glutamine synthetase that block its interaction with transcription factor TnrA. Mol Microbiol 2002;45(3):627–35.

[27] Wray Jr. LV, Fisher SH. Functional roles of the conserved Glu304 loop of Bacillus subtilis glutamine synthetase. J Bacteriol 2010;192:5018–25.

[28] Stadtman ER. Regulation of glutamine synthetase activity. EcoSal Plus 2004;1.

[29] Liu L, Duan X, Wu J. L-tryptophan production in Escherichia coli improved by weakening the pta-AckA pathway. PLoS One 2016;11:e0158200.

[30] Hart Y, Madar D, Yuan J, Bren A, Mayo AE, Rabinowitz JD, et al. Robust control of nitrogen assimilation by a bifunctional enzyme in E. coli. Mol Cell 2011;41:117–27.

[31] *,†,§.,‡ Cheryl R, Dittrich GNB, San Ka-Yiu. Characterization of the acetate-producing pathways in Escherichia coli. Biotechnol Prog 2005;21:1062–7.

[32] Fang H, Xie X, Xu Q, Zhang C, Chen N. Enhancement of cytidine production by coexpression of gnd, zwf, and prs genes in recombinant Escherichia coli CYT15. Biotechnol Lett 2013;35:245–51.

[33] Trondle J, Schoppel K, Bleidt A, Trachtmann N, Sprenger GA, Weuster-Botz D. Metabolic control analysis of L-tryptophan production with Escherichia coli based on data from short-term perturbation experiments. J Biotechnol 2019;307:15–28.

[34] Chen L, Zeng AP. Rational design and metabolic analysis of Escherichia coli for effective production of L-tryptophan at high concentration. Appl Microbiol Biotechnol 2017;101:559–68.

[35] Al-Rabiee R, Zhang Y, Grant GA. The mechanism of velocity modulated allosteric regulation in D-3-phosphoglycerate dehydrogenase. Site-directed mutagenesis of effector binding site residues. J Biol Chem 1996;271:23235–8.

[36] Mundhada H, Seoane JM, Schneider K, Koza A, Christensen HB, Klein T, et al. Increased production of L-serine in Escherichia coli through adaptive laboratory evolution. Metab Eng 2017;39:141–50.

[37] Yan-Yan Cui1 CL, Zhang1 Yuan-Yuan, Huang1 Jian. Jian-Zhong Liu1*. Production of shikimic acid from Escherichia coli through chemically inducible chromosomal evolution and cofactor metabolic engineering. microb Cell Fact 2014;13:21.

[38] Sauer U, Canonaco F, Heri S, Perrenoud A, Fischer E. The soluble and membrane-bound transhydrogenases UdhA and PntAB have divergent functions in NADPH metabolism ofEscherichia coli. J Biol Chem 2004;279:6613–9.
