# Supplementary material for: 5-Hydroxytryptophan (5-HTP): Natural Occurrence, Analysis, Biosynthesis, Biotechnology, Physiology and Toxicology
Source: Int J Mol Sci. 2020 Dec 26;22(1):181. doi: 10.3390/ijms22010181 (PMC7796270; doi:10.3390/ijms22010181)
Supplement: Supplementary file 1 [file ijms-22-00181-s001.zip › 5-HTP.Data/PDF/3517053581/Journal of Bacteriology-2009-Pribat-475.full.pdf]

JOURNAL OF BACTERIOLOGY, Jan. 2010, p. 475–482 Vol. 192, No. 2
0021-9193/10/$12.00 doi:10.1128/JB.01198-09
Copyright © 2010, American Society for Microbiology. All Rights Reserved.

FolX and FolM Are Essential for Tetrahydromonapterin Synthesis in *Escherichia coli* and *Pseudomonas aeruginosa*▽†

Anne Pribat,[1]‡ Ian K. Blaby,[2]‡ Aurora Lara-Núñez,[3] Jesse F. Gregory III,[3] Valérie de Crécy-Lagard,[2]* and Andrew D. Hanson[1]*

Horticultural Sciences Department,[1] Microbiology and Cell Science Department,[2] and Food Science and Human Nutrition Department,[3] University of Florida, Gainesville, Florida 32611

Received 4 September 2009/Accepted 27 October 2009

Tetrahydromonapterin is a major pterin in *Escherichia coli* and is hypothesized to be the cofactor for phenylalanine hydroxylase (PhhA) in *Pseudomonas aeruginosa*, but neither its biosynthetic origin nor its cofactor role has been clearly demonstrated. A comparative genomics analysis implicated the enigmatic *folX* and *folM* genes in tetrahydromonapterin synthesis via their phyletic distribution and chromosomal clustering patterns. *folX* encodes dihydroneopterin triphosphate epimerase, which interconverts dihydroneopterin triphosphate and dihydromonapterin triphosphate. *folM* encodes an unusual short-chain dehydrogenase/reductase known to have dihydrofolate and dihydrobiopterin reductase activity. The roles of FolX and FolM were tested experimentally first in *E. coli*, which lacks PhhA and in which the expression of *P. aeruginosa* PhhA plus the recycling enzyme pterin 4a-carbinolamine dehydratase, PhhB, rescues tyrosine auxotrophy. This rescue was abrogated by deleting *folX* or *folM* and restored by expressing the deleted gene from a plasmid. The *folX* deletion selectively eliminated tetrahydromonapterin production, which far exceeded folate production. Purified FolM showed high, NADPH-dependent dihydromonapterin reductase activity. These results were substantiated in *P. aeruginosa* by deleting *tyrA* (making PhhA the sole source of tyrosine) and *folX*. The Δ*tyrA* strain was, as expected, prototrophic for tyrosine, whereas the Δ*tyrA* Δ*folX* strain was auxotrophic. As in *E. coli*, the *folX* deletant lacked tetrahydromonapterin. Collectively, these data establish that tetrahydromonapterin formation requires both FolX and FolM, that tetrahydromonapterin is the physiological cofactor for PhhA, and that tetrahydromonapterin can outrank folate as an end product of pterin biosynthesis.

Pterins contain the bicyclic pteridine ring with an amino group in the 2-position and an oxo group in the 4-position; they can be reduced through the dihydro forms to the tetrahydro forms, which are active as cofactors (Fig. 1A). Tetrahydropterins are known to be the cofactors for phenylalanine hydroxylases from *Pseudomonas* and *Chromatium* species as well as for mammalian aromatic amino acid hydroxylases and other mammalian enzymes (13, 17, 38, 41) (Fig. 1B). Although the identity of the mammalian tetrahydropterin cofactor, tetrahydrobiopterin (H_4-BPt), is firmly established (38), the same is not true for bacteria, and the biosynthesis of bacterial tetrahydropterins is not well understood.

While a few bacterial taxa, such as *Cyanobacteria* and *Chlorobia*, produce H_4-BPt, most do not, as judged directly from pterin analysis and indirectly from the rarity of H_4-BPt biosynthesis genes 6-pyruvoyltetrahydropterin synthase II (PTPS-II) and sepiapterin reductase (SR) (Fig. 1C) among sequenced genomes (12, 25). As bacteria lacking H_4-BPt include *Pseudomonas* and many others with phenylalanine hydroxylase genes, it is clear that bacterial phenylalanine hydroxylases generally must use a cofactor other than H_4-BPt. The most prominent candidate is tetrahydromonapterin (H_4-MPt), which occurs in *Escherichia coli* (21) and almost certainly also in *Pseudomonas* species (11, 17). H_4-MPt could be derived from the dihydropterin intermediates of folate biosynthesis via two different routes (Fig. 1C). These are (i) the conversion of dihydroneopterin triphosphate (H_2-NPt-P_3) to dihydromonapterin triphosphate (H_2-MPt-P_3) by H_2-NPt-P_3 epimerase (FolX) followed by dephosphorylation and reduction to the tetrahydro level, and (ii) the conversion of dihydroneopterin (H_2-NPt) to dihydromonapterin (H_2-MPt) by the epimerase action of dihydroneopterin aldolase (FolB) and then reduction. FolB is a fairly well-understood enzyme of folate synthesis (9), but FolX has no known biological role and a *folX* deletant has no obvious phenotype (19). *folX* genes apparently are confined to *Gammaproteobacteria* (9).

Although the epimerase activities of FolX and FolB have been demonstrated amply *in vitro* (1, 5, 19), no genetic evidence links either enzyme to H_4-MPt formation *in vivo*. The situation with the reduction of H_2-MPt to H_4-MPt is even less clear, because this activity has not been investigated experimentally. A candidate enzyme for this step nevertheless can be proposed on bioinformatic grounds: the somewhat mysterious FolM protein (9). FolM belongs to a subset of the short-chain dehydrogenase/reductase (SDR) family having the characteristic motif TGX_3RXG (in place of TGX_3GXG, which typifies other SDRs). The archetype of this subset is *Leishmania* pteridine reductase 1 (PTR1), which reduces various dihydropterins to the tetrahydro state (15). *E. coli* FolM has low

* Corresponding author. Mailing address for A. D. Hanson: University of Florida, Horticultural Sciences Department, P.O. Box 110690, Gainesville, FL 32611. Phone: (352) 392-1928. Fax: (352) 392-5653. E-mail: adha@ufl.edu. Mailing address for V. de Crécy-Lagard: University of Florida, Microbiology and Cell Science Department, P.O. Box 110700, Gainesville, FL 32611. Phone: (352) 392-9416. Fax: (352) 392-5922. E-mail: vcrecy@ufl.edu.

† Supplemental material for this article may be found at http://jb.asm.org/.

‡ These authors contributed equally to this paper.

▽ Published ahead of print on 6 November 2009.

Downloaded from http://jb.asm.org/ on December 1, 2020 by guest

dihydrofolate (H_2-folate) and dihydrobiopterin (H_2-BPt) reductase activities *in vitro* (14), but neither of these is likely to be its physiological function, since H_2-folate reduction normally is mediated by FolA and *E. coli* lacks H_4-BPt. *folM* genes occur in many Gram-negative organisms, including *Chlamdiae*, *Chloroflexi*, *Cyanobacteria*, *Acidobacteria*, *Planctomycetes*, *Alphaproteobacteria*, *Betaproteobacteria*, *Gammaproteobacteria*, and *Deltaproteobacteria* (9).

We report here comparative genomic and genetic evidence that FolX and FolM are required for H_4-MPt synthesis in *E. coli* and *P. aeruginosa*, the bacteria in which H_4-MPt has been most studied, and biochemical evidence that FolM has high H_2-MPt reductase activity. We also point out gaps in the understanding of pterin metabolism that our data bring sharply into focus.

MATERIALS AND METHODS

Bioinformatics. Bacterial genomes were analyzed using the SEED database and its tools (31). Full results of the analysis are available at the public SEED server (theseed.uchicago.edu) in the subsystem pterin metabolism 3.

Pterins. 7,8-Dihydromonapterin, 7,8-dihydroneopterin, 6-hydroxymethyl-7,8-dihydropterin hydrochloride, L-monapterin, 5,6,7,8-tetrahydromonapterin, and 7,8-dihydrofolate were obtained from Schircks Laboratories (www.schircks.com). Near-saturated solutions were freshly prepared in N_2-sparged potassium phosphate (10 mM, pH 7.5), excluding light, and titers were determined spectrophotometrically using published extinction coefficients (4, 32). Quinonoid dihydromonapterin was prepared *in situ* in enzyme assays using H_2O_2 and horseradish peroxidase (26). Assay mixtures (100 μl) contained 100 mM potassium phosphate (pH 6.0), 1 μg of horseradish peroxidase, 0.5 mM H_2O_2, 50 μM H_4-MPt, 100 μM NADPH, and 5 μg of purified FolM.

Bacterial strains, plasmids, and media. *E. coli* K-12 MG1655 and *P. aeruginosa* PAO1 were grown at 37°C on Luria-Bertani medium (LB) or minimal medium containing M9 salts (35), 0.1 mM $CaCl_2$, 0.5 mM $MgSO_4$, and 0.4% (wt/vol) glucose as the carbon source unless otherwise noted. Media were solidified with 15 g of agar/liter. Kanamycin (50 μg/ml), ampicillin (100 μg/ml), spectinomycin (100 μg/ml), thiamine (17 μg/ml), L-phenylalanine (50 μg/ml), L-tyrosine (54 μg/ml), L-tryptophan (50 μg/ml), isopropyl-β-D-thiogalactopyranoside (IPTG; 1 mM), *p*-aminobenzoic acid (10 μM), 4-hydroxybenzoate (10 μM), and 2,3-dihydroxybenzoate (10 μM) were added as required. Plasmids used in this study are listed in Table S1 in the supplemental material.

***E. coli* deletions and functional complementation tests.** *E. coli* strains MG1655 *folX*::Kn, MG1655 *folM*::Kn, JP2255 *folX*::Kn, and JP2255 *folM*::Kn were created by the P1 transduction of the respective mutant allele from the Keio collection into strain MG1655 or JP2255 (2). Where necessary, the kanamycin cassette was recombined out using pCP20 (8). Deletions were confirmed by PCR and sequencing (see Table S2 in the supplemental material). The plasmids pJS11 (37), which carries *P. aeruginosa phhA*, and pBluescript, carrying *P. aeruginosa phhB* (27), were transformed simultaneously by electroporation into strains JP2255, JP2255 *folM*, and JP2255 *folX*. Complementation was assessed by plating trans-

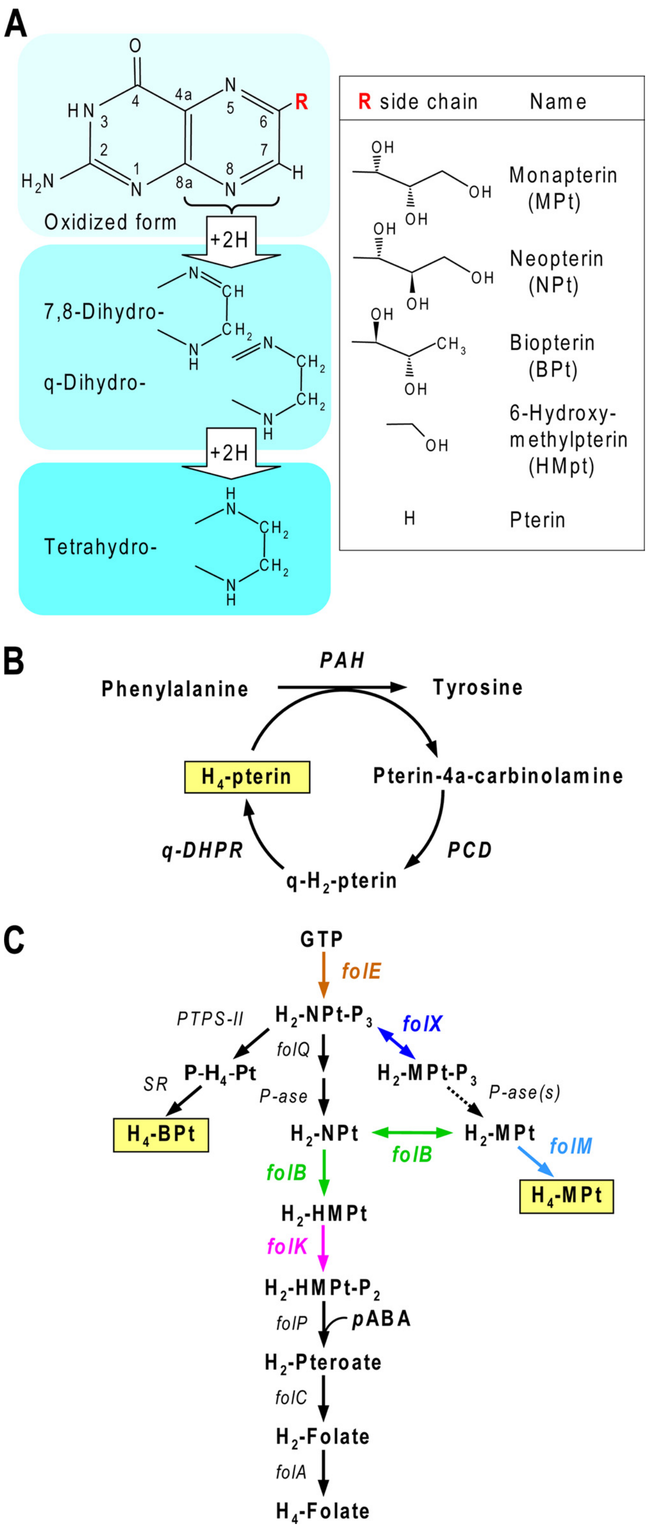

FIG. 1. Tetrahydropterin structure, cofactor role, and biosynthesis. (A) The pterin nucleus, its levels of reduction, and the structures of compounds relevant to this study. (B) The requirement for a tetrahydropterin (H_4-pterin) cofactor for phenylalanine hydroxylase (PAH) and the cofactor regeneration cycle involving pterin-4a-carbinolamine dehydratase (PCD) and quinonoid dihydropterin (q-H_2-pterin) reductase (q-DHPR; EC 1.5.1.34). (C) The established steps in tetrahydrobiopterin (H_4-BPt) biosynthesis and possible routes for tetrahydromonapterin (H_4-MPt) biosynthesis in relation to the folate pathway. H_4-BPt is formed by the sequential action of 6-pyruvoyltetrahydropterin (P-H_4-Pt) synthase (PTPS-II) and sepiapterin reductase (SR). H_4-MPt could originate via the FolX-catalyzed epimerization of dihydroneopterin triphosphate (H_2-NPt-P_3) to dihydromonapterin triphosphate (H_2-MPt-P_3), followed by dephosphorylation to dihydromonapterin (H_2-MPt) and reduction by a dihydropterin reductase (EC 1.5.1.33), putatively FolM. H_4-MPt also could come from the FolB-mediated epimerization of dihydroneopterin (H_2-NPt) followed by reduction. FolB also mediates the side chain cleavage of H_2-NPt or H_2-MPt to give 6-hydroxymethyldihydropterin (H_2-HMPt); the H_2-MPt cleavage is omitted for simplicity. Other abbreviations: P-ase, phosphatase; H_2-HMPt-P_2, 6-hydroxymethyldihydropterin diphosphate; *p*ABA, *p*-aminobenzoate; H_2-pteroate, dihydropteroate; H_2-folate, dihydrofolate; H_4-folate, tetrahydrofolate.

Downloaded from http://jb.asm.org/ on December 1, 2020 by guest

formants onto minimal medium with 0.4% (vol/vol) glycerol as the carbon source, plus appropriate antibiotics, thiamine, and L-phenylalanine, with or without L-tyrosine. Plates were incubated at 37°C for 2 to 3 days. To restore the phenotype by complementation, *folM* and *folX* were PCR amplified and cloned into pEXT21 (10). These plasmids and the empty vector then were transformed by electroporation into their respective host strains expressing *phhA* and *phhB* and were plated on LB containing ampicillin, kanamycin, and spectinomycin. Transformants then were plated on minimal medium as described above.

***P. aeruginosa* deletions and functional complementation tests.** Genes were deleted in *P. aeruginosa* using pEX18Gm and pEX18Tet (20), both of which contain the *sacB* gene for the negative selection of the suicide marker. Approximately 1-kbp regions flanking each target gene were amplified by PCR. To delete *folX*, the *gent* gene from pEX18Gm was amplified by PCR. In a three-way ligation, the resistance gene was cloned between the flanking regions into pEX18Tet. In a similar manner, to generate the deletion construct for *tyrA*, the *tet* gene from pEX18Tet was amplified and cloned between the flanking regions into pEX18Gm. The deletion constructs were transformed by electroporation as described previously (7), and after being plated on LB containing the appropriate antibiotic, resistant colonies capable of growth on LB containing 6% (wt/vol) sucrose were identified and verified by PCR. For deletions in *tyrA*, the following also were added to media: *p*-aminobenzoic acid, 4-hydroxybenzoate, 2,3-dihydroxybenzoate, L-tryptophan, L-phenylalanine, and L-tyrosine at the concentrations specified above. Deletants were confirmed by PCR and sequencing (see Table S2 in the supplemental material). To negate the potentially lethal polar effects of deleting *tyrA* on the immediately downstream gene *cmk* (encoding cytidylate kinase), *cmk* was cloned by PCR into pHERD20T (33) and transformed into the Δ*tyrA* and Δ*folX* Δ*tyrA* strains. PhhA function was assessed by being streaked on minimal medium plates (as described above) containing appropriate antibiotics and *p*-aminobenzoic acid, 4-hydroxybenzoate, 2,3-dihydroxybenzoate, and L-tryptophan, with or without L-phenylalanine and L-tyrosine. The plates were incubated at 37°C for 2 to 3 days.

Pterin and folate analysis. Wild-type and Δ*folX E. coli* strains were grown at 37°C in 50 ml of minimal medium until the A_{600} reached 1.0 ± 0.1. Cells were harvested by centrifugation (6,000 × *g*, 20 min, 4°C) and washed three times with cold 25 mM potassium phosphate, pH 7.5 (wash buffer). Washed pellets and the corresponding culture medium were stored at −80°C until use. For pterin analysis, pellets were resuspended in 1 ml of wash buffer and broken in a Mini-Bead-Beater using 0.1-mm zirconia-silica beads. The extract was removed, and the beads were washed twice with 0.75 ml of wash buffer. The extract and washes were pooled and centrifuged (10,000 × *g*, 10 min, 4°C). Aliquots (100 μl) of supernatants (intracellular fraction) and culture media (extracellular fraction) were oxidized for 1 h in the dark at 4°C by adding 10 μl of 1% (wt/vol) I_2–2% (wt/vol) KI in 0.1 or 1 M HCl, after which the excess iodine was oxidized by adding 10 μl of 10% (wt/vol) sodium ascorbate. Pterins (50- or 100-μl injections) were separated on a 4-μm, 250- by 4.6-mm Synergi Fusion-RP 80 column (Phenomenex) that was eluted isocratically with 10 mM sodium phosphate (pH 6.0) at 1.5 ml/min. Peaks were detected by fluorescence (350 nm excitation, 450 nm emission) and identified relative to standards. Pterins in *P. aeruginosa* wild-type and Δ*folX* strains were analyzed the same way, except that the cells were harvested when the A_{600} reached 0.6 ± 0.1. For folate analysis, cell pellets were suspended in 50 mM HEPES–CHES [2-(*N*-cyclohexylamino)ethanesulfonic acid] (pH 7.85, 10 ml final volume) containing 2% (wt/vol) ascorbic acid and 10 mM β-mercaptoethanol (extraction buffer), sonicated, boiled for 10 min, and centrifuged (13,000 × *g*, 10 min). The samples were reextracted the same way, and the combined extracts were treated for 2 h at 37°C with 2 ml of dialyzed rat plasma to deglutamylate folates to the monoglutamate level. Samples then were boiled for 10 min, centrifuged as described above, and filtered. Culture medium samples (4 ml) were processed similarly after being mixed with 6 ml of ×1.67 extraction buffer. Folates were isolated using 2-ml folate affinity columns (16). Samples of the eluate were analyzed by high-performance liquid chromatography (HPLC) with electrochemical detection (3). The detector response was calibrated with folate standards from Merck Eprova (www.merckeprova.com).

Expression and purification of recombinant FolM. *E. coli* BL21-CodonPlus (DE3)-RIPL cells (Stratagene) were transformed with pFolM (14), which encodes *E. coli* FolM with an N-terminal His tag. Cells were grown at 37°C in LB containing appropriate antibiotics. When the A_{600} reached 0.6, IPTG was added to a final concentration of 1 mM and growth was continued for 4 h. Subsequent steps were carried out at 4°C. Cells from a 200-ml culture were harvested by centrifugation, resuspended in 50 mM sodium phosphate (pH 8.0), 300 mM NaCl, 10% (vol/vol) glycerol, 10 mM imidazole, and disrupted by sonication. The cleared supernatant was loaded onto a 0.35-ml Ni^{2+}-nitrilotriacetic acid agarose resin column (Qiagen) equilibrated with the sonication buffer. After the column was washed with 16 ml of 50 mM sodium phosphate, 300 mM NaCl, 10% (vol/vol) glycerol, 20 mM imidazole (pH 8.0), the recombinant protein was eluted with 3 ml of the same buffer containing 250 mM imidazole and was desalted on PD-10 columns equilibrated in 50 mM sodium phosphate (pH 8.0), 100 mM NaCl, 10% (vol/vol) glycerol. The purified protein was stable to freezing in liquid N_2 and storage at −80°C. The protein concentration was estimated by the Bradford method (6) using bovine serum albumin as a standard.

Biochemical analysis of FolM. FolM activity was measured spectrophotometrically. Assays (100 μl) were run at 22 to 23°C in 100 mM potassium phosphate (pH 6.0) and contained 100 μM NADPH, purified enzyme (2 to 20 μg), a pterin, and (except when quinonoid dihydromonapterin was the substrate) 10 mM β-mercaptoethanol. All components except NADPH were incubated together for 30 s before starting the reaction by adding NADPH. Reductase activities using various substrates (50 μM) were measured by continuous assay, obtaining initial velocities from the rate of decrease of absorbance at 340 nm. Blank rates measured in the absence of enzyme were used to correct for the nonenzymatic breakdown of the substrate and the oxidation of NADPH. As pterins exhibit absorbance changes when reduced, the following molar extinction coefficients ($\varepsilon_{340,}$ M^{-1} cm^{-1}; from the literature or determined empirically) were used for the coupled oxidation/reduction of NADPH-pterin at pH 6.0: H_2-MPt, 6,028; H_2-folate, 12,300 (14); and H_2-HMPt, 10,295. The K_m and V_{max} values for H_2-MPt were measured in a discontinuous assay because of the high absorbance of H_2-MPt at 340 nm. Reactions were stopped after 30 s by adding 400 μl of 0.5 M Tris-HCl (pH 8.0) (FolM is inactive at this pH), and rates were determined as described above (ε_{340} for NADPH/H_2-MPt at pH 8.0 is 11,040 M^{-1} cm^{-1}). Data were fitted to a hyperbolic curve with the MicroCal Origin 3.0 software (Microcal Software, Inc., Northampton, MA).

RESULTS

Genomic context connects *folX* and *folM* with tetrahydropterin production. The analysis of 724 bacterial genomes using the SEED database and its tools (31) revealed 184 that encode phenylalanine hydroxylase (PhhA), only 19 of which also encode the characteristic H_4-BPt synthesis enzyme PTPS-II (25). Of the remaining 165 genomes with PhhA, the majority (108 genomes) encode FolM and, in *Gammaproteobacteria*, frequently also FolX. Conversely, genomes with PTPS-II very rarely encode FolM and never encode FolX. Thus, there is a certain reciprocity between the phyletic distribution of PTPS-II and that of FolM and FolX (Fig. 2A), suggesting that the latter produces an alternative to H_4-BPt. Gene-clustering evidence (Fig. 2B) supports this possibility, for it shows that *folX* and *folM* often are associated with each other and with *folE* (the first gene of pterin synthesis), and sometimes also with *folK* (encoding hydroxymethyldihydropterin pyrophosphokinase, which converts H_2-HMPt to its pyrophosphate) (Fig. 1C). The *folM-folE* association was noted previously (9). Taken together, the comparative genomic data suggest that FolX and FolM have related functions in tetrahydropterin biosynthesis. A simple hypothesis is that they mediate the epimerization and reduction steps in H_4-MPt synthesis (Fig. 1C).

Deleting *E. coli folX* selectively eliminates monapterin production and secretion. The hypothesis described above was tested first in *E. coli*, because *E. coli* has H_4-MPt as a prominent intracellular pterin (21) and secretes large amounts of monapterin in some form (40). The reduction state of the secreted form is uncertain, because H_4-MPt and H_2-MPt can spontaneously oxidize to MPt and are expected to do so once released to the medium (29, 34). A *folX* deletant was constructed, and its intra- and extracellular pterin and folate profiles were compared to those of the parent wild-type strain. Pterins were analyzed by fluorometric HPLC after acidic iodine oxidation to their fluorescent aromatic forms. The Δ*folX* strain showed the selective loss of the MPt peak from the

Downloaded from http://jb.asm.org/ on December 1, 2020 by guest

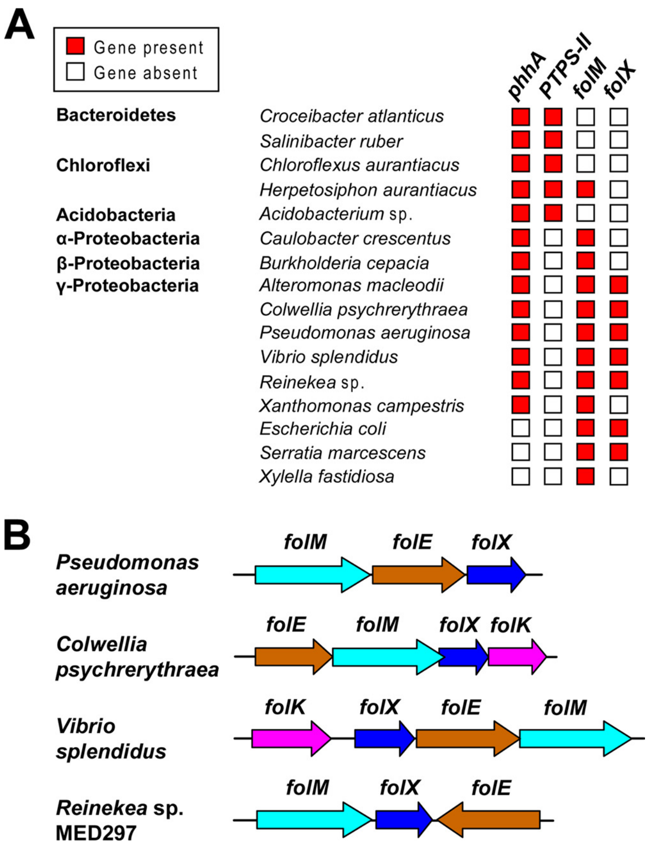

FIG. 2. Comparative genomic evidence implicating *folX* and *folM* in tetrahydromonapterin synthesis. (A) Phyletic distribution of phenylalanine hydroxylase (*phhA*), PTPS-II, *folM*, and *folX* genes among representative genomes. Note the anticorrelation between PTPS-II and *folM* or *folX*. Note also that *folM* sometimes is unaccompanied by *folX*, and that *folM* and *folX* sometimes occur without *phhA*. (B) Clustering of *folX* and *folM* with each other and with *folE* in diverse *Gammaproteobacteria*. In two genomes these genes also cluster with *folK*. Genes are colored to match Fig. 1C. Arrows indicate the transcriptional direction; overlaps between arrows indicate translational coupling.

intracellular profile (Fig. 3A) and the complete loss of extracellular MPt and most other extracellular pterins (which appear to be MPt breakdown products) (Fig. 3B). The intra- and extracellular folate profiles of the Δ*folX* strain were, however, essentially identical to those of the wild type (Fig. 3C). These results establish that FolX is the only source of MPt in *E. coli*, and it has no role in folate synthesis. The combined intra- plus extracellular pterin production of wild-type cells was 9.1 nmol/mg protein; it was 1 nmol/mg protein in the deletant. Since folates contain a pterin moiety, both values should be expanded by 1.3 nmol/mg protein (the approximate sum of intra- plus extracellular folate production) to obtain total pterin production. Total pterin values thus are 10.4 and 2.3 nmol/mg protein for the wild type and deletant, respectively. The difference between these values (8.1 nmol/mg protein) provides an estimate of the normal FolX-mediated flux to MPt. This flux is sixfold larger than the flux to folates (1.3 nmol/mg protein). It is noteworthy that 99% of the MPt produced by wild-type *E. coli* cells was extracellular, as was 98% of total pterin (Fig. 3B).

***folX* and *folM* are essential for *P. aeruginosa* phenylalanine hydroxylase function in *E. coli*.** To further explore the roles of *folX* and *folM* in H_4-MPt formation and function, we turned to a convenient heterologous expression system in which *P. aeruginosa* PhhA is expressed in *E. coli* together with the pterin recycling enzyme pterin-4a-carbinolamine dehydratase (PhhB) (27, 37, 41). As *E. coli* has neither PhhA nor PhhB, their expression confers the ability to form tyrosine from phenylalanine, thereby allowing the rescue of the tyrosine auxotrophy caused by a *tyrA* mutation. This rescue was abrogated by deleting either *folX* or *folM* and restored by expressing the deleted gene from a plasmid (Fig. 3D). Because the Δ*folX* strain lacks MPt and only MPt (Fig. 3A), these results establish that H_4-MPt is the physiological cofactor for PhhA, at least in *E. coli*. They also show that FolM is required for PhhA function, most probably to reduce H_2-MPt to H_4-MPt. An alternative possibility, that FolM is the quinonoid dihydropterin reductase that recycles oxidized H_4-MPt (Fig. 1B), is unlikely, because this activity is reported to reside in the *E. coli* NfnB protein (39) and was not detected in recombinant FolM (see below).

Recombinant FolM has high H_2-MPt reductase activity. To corroborate the inference that FolM is the source of H_2-MPt reductase activity, recombinant FolM was purified (Fig. 4A) and assayed using H_2-MPt or other pterins (50 μM) as substrates and NADPH as a cofactor (Fig. 4B). Assays were made at pH 6.0 because we found that FolM is most active at acidic pH, as previously reported (14). FolM activity with H_2-MPt was 16-fold higher than that with H_2-folate, previously the best known substrate (14). FolM also showed some activity with the folate pathway intermediate 6-hydroxymethyldihydropterin (H_2-HMPt) but not with dihydroneopterin (H_2-NPt), the epimer of H_2-MPt. Monapterin was not a substrate, and neither, as far as could be judged, was the quinonoid form of dihydromonapterin. This form (prepared *in situ* by oxidizing H_4-MPt) rearranges very fast to H_2-MPt (23), and no activity beyond that attributable to H_2-MPt thus formed was detected. NADH could not replace NADPH as the cofactor. The kinetic characterization of the H_2-MPt reductase activity (Fig. 4C) indicated a higher V_{max} than that reported for H_2-folate (5.99 versus 0.083 μmol min^{-1} mg^{-1}) and also a higher K_m (147 versus 9.5 μM) (14).

FolX is essential for PhhA function in *P. aeruginosa*. To extend the findings described above to *P. aeruginosa*, we first constructed a Δ*folX* strain and demonstrated that the deletion abolished intra- and extracellular MPt production (Fig. 5A and B). As in *E. coli*, >95% of the MPt made by wild-type cells was extracellular (Fig. 5B). A *tyrA* deletant then was constructed as well as a Δ*tyrA* Δ*folX* strain. The Δ*tyrA* strain was, as expected, prototrophic for tyrosine, since PhhA allows tyrosine formation from phenylalanine (Fig. 4C). The Δ*tyrA* Δ*folX* strain was auxotrophic for tyrosine (Fig. 4C), showing that *folX* is required for PhhA function *in situ* as well as when expressed heterologously in *E. coli*.

DISCUSSION

Our data confirm the hypothesis (1) that FolX is the sole source of MPt in *E. coli*, and they show that this is the case in *P. aeruginosa* as well. They also establish a physiological function for FolM as an H_2-MPt reductase, again confirming an

Downloaded from http://jb.asm.org/ on December 1, 2020 by guest

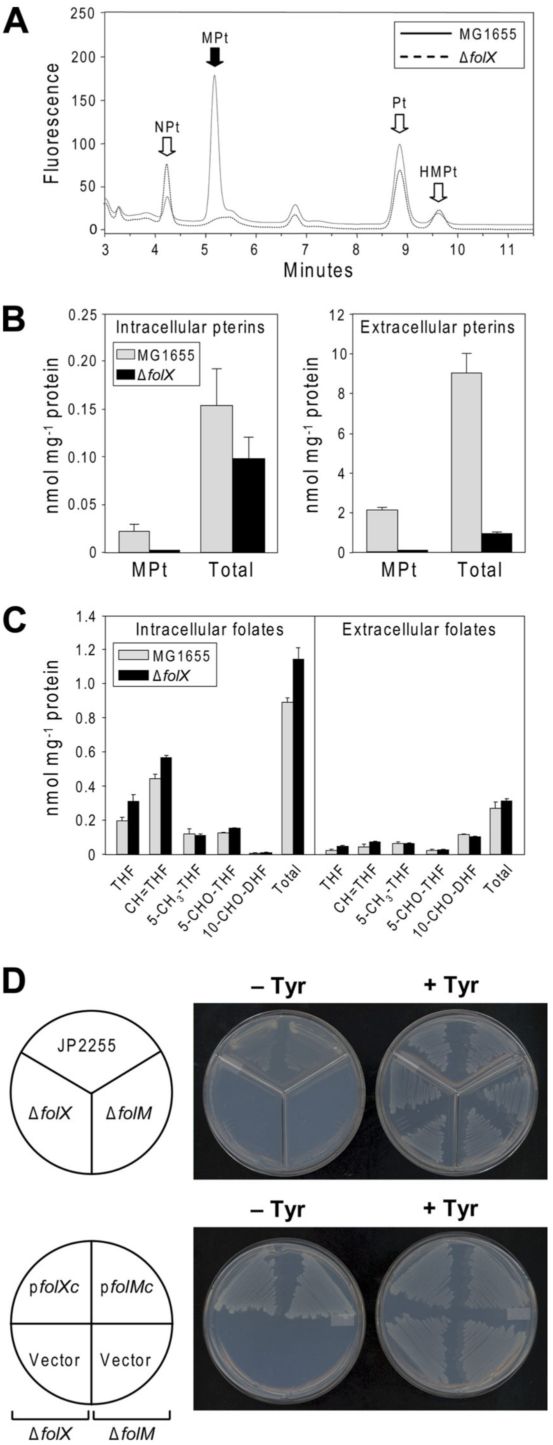

FIG. 3. Genetic evidence implicating *folX* and *folM* in tetrahydromonapterin synthesis in *E. coli* and tetrahydromonapterin as the cofactor for *P. aeruginosa* PhhA. (A) Fluorometric HPLC profile of pterins extracted from wild-type (MG1655) and Δ*folX E. coli* strains grown in liquid M9 medium plus 0.4% glucose to an A_{600} of 1.5. Samples were oxidized before analysis to convert di- and tetrahydropterins to their fluorescent aromatic forms. Note the selective loss of monapterin (MPt) from the *folX* deletant. NPt, neopterin; Pt, pterin; HMPt, 6-hydroxymethylpterin. (B) Quantitation of intra- and extracellular pterins (as MPt equivalents per mg cellular protein) in MG1655 and Δ*folX* cultures grown to an A_{600} of 1.0 in the medium described for panel A. Data are means and standard errors from three replicates. Besides MPt, the most prominent extracellular species was pterin, a breakdown product of MPt (34). (C) Quantitation of intra- and extracellular folates (per mg cellular protein) from MG1655 and Δ*folX* strains grown as described for panel B. Data are means and standard errors from three replicates. THF, tetrahydrofolate; CH = THF, 5,10-methenyltetrahydrofolate; 5-CH_3-THF, 5-methyltetrahydrofolate; 5-CHO-THF, 5-formyltetrahydrofolate; 10-CHO-DHF, 10-formydihydrofolate. Note that the analytical procedures convert 5,10-methylenetetrahydrofolate to THF and 10-formyltetrahydrofolate to CH = THF. 10-CHO-DHF is an oxidation product of 10-formyltetrahydrofolate. (D) The upper frames show the abrogation of tyrosine prototrophy by deleting *folX* or *folM* from the *tyrA* strain JP2255 harboring plasmids carrying *P. aeruginosa phhA* and *phhB*. Duplicate strains were tested. The lower frames show the restoration of tyrosine prototrophy to *folX* or *folM* deletants in the system described in the upper frames by introducing plasmid-borne *folX* (p*folXc*) or *folM* (p*folMc*), respectively. Controls with the plasmid alone (Vector) were included. Plates contained M9 minimal medium supplemented with 0.4% glycerol, 17 μg/ml thiamine, 50 μg/ml L-phenylalanine, 1 mM IPTG, and appropriate antibiotics, with or without 54 μg/ml L-tyrosine.

earlier hypothesis (9). In this connection, the lack of activity against H_2-NPt is significant, because, as an intermediate of folate synthesis (Fig. 1C), this pterin must remain in the dihydro form. Although the K_m for H_2-MPt is higher than that for H_2-folate, the latter would not be expected to displace H_2-MPt *in vivo*, because H_2-folate concentrations are kept extremely low by FolA, whose K_m for H_2-folate is close to 1 μM (30, 36).

Our results also raise three questions that reveal gaps in the present understanding of bacterial pterin metabolism. First, since FolB has high H_2-NPt epimerase activity *in vitro* (16% of its H_2-NPt→H_2-HMPt cleavage activity) (19), why can FolB not give rise to H_2-MPt when FolX is ablated? The answer may lie in the occurrence in diverse bacteria of genes encoding fusions of FolB with FolK, the next enzyme in the folate synthesis pathway (Fig. 6A). Such a fusion implies the possibility of the metabolic channeling of H_2-NPt straight through to H_2-HMPt diphosphate (Fig. 1C), with any H_2-MPt formed via the epimerase activity of FolB being inaccessible to other enzymes. It therefore is conceivable that FolB-FolK fusions are covalently linked versions of a noncovalent FolB-FolK complex in which channeling also occurs. A related possibility is that the FolB-FolK complex lacks epimerase activity.

The second question is about the role of FolM in bacteria whose genomes lack a *folX* gene. Such bacteria are numerous, and some of them (e.g., *Burkholderia* and *Xanthomonas*) contain a *phhA* gene and, hence, almost surely produce a tetrahydropterin (Fig. 2A). Perhaps in such cases, FolM has access to H_2-MPt produced by FolB because FolB is not tightly complexed with FolK. In this connection, it is interesting that, in *Gammaproteobacteria* that lack *folX*, *folM* clusters on the chromosome with *folB* instead of *folX* (Fig. 6B). Alternatively, FolM might reduce the folate synthesis intermediate H_2-HMPt, which is a substrate for *E. coli* FolM (Fig. 4B), but this would set up competition between tetrahydropterin and folate synthesis.

The third question concerns the biological role of H_4-MPt in *E. coli* and other bacteria (e.g., *Serratia* and *Shigella*) that have *folX* and *folM* but no phenylalanine hydroxylase gene (Fig. 2A). It is probable that, like *E. coli*, the others produce and secrete H_4-MPt, because high levels of MPt were found in the

Downloaded from http://jb.asm.org/ on December 1, 2020 by guest

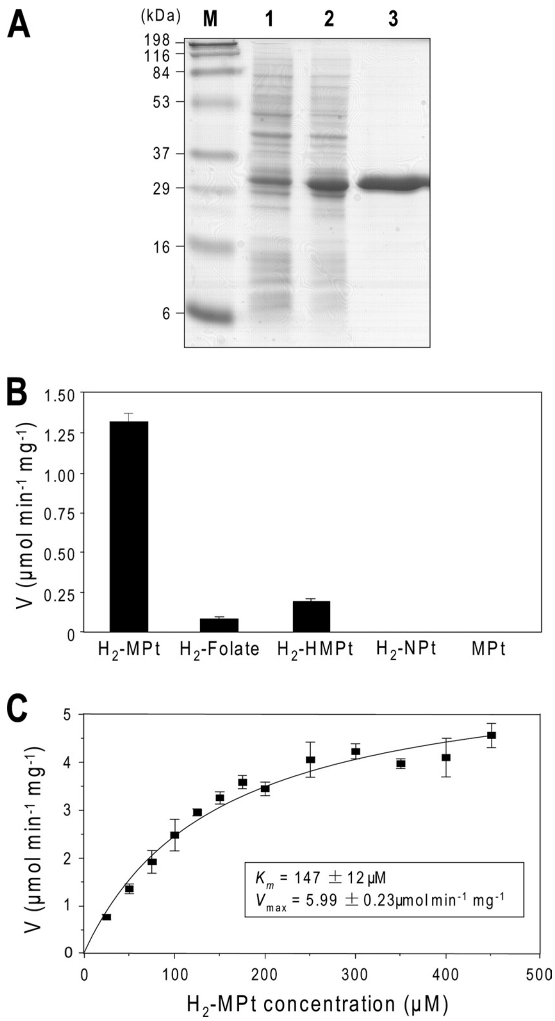

FIG. 4. Purification and characterization of *E. coli* FolM. (A) SDS-polyacrylamide gel electrophoresis of extracts of cells (~17 µg protein) harboring pFolM before and after 4 h IPTG induction (lanes 1 and 2, respectively) and 10 µg of Ni^{2+} affinity-purified FolM protein (lane 3). Staining was done with Coomassie blue. The positions of molecular markers (M; in kDa) are indicated. (B) Reductase activities measured at pH 6.0 using various substrates (50 µM) and 100 µM NADPH. Values are means and standard errors from three replicates. Abbreviations are defined in the legend to Fig. 1. (C) Dihydromonapterin reductase activity as a function of dihydromonapterin concentration. Assays were made at pH 6.0 and contained 100 µM NADPH. The K_m and V_{max} values estimated by hyperbolic curve fitting are shown in the inset.

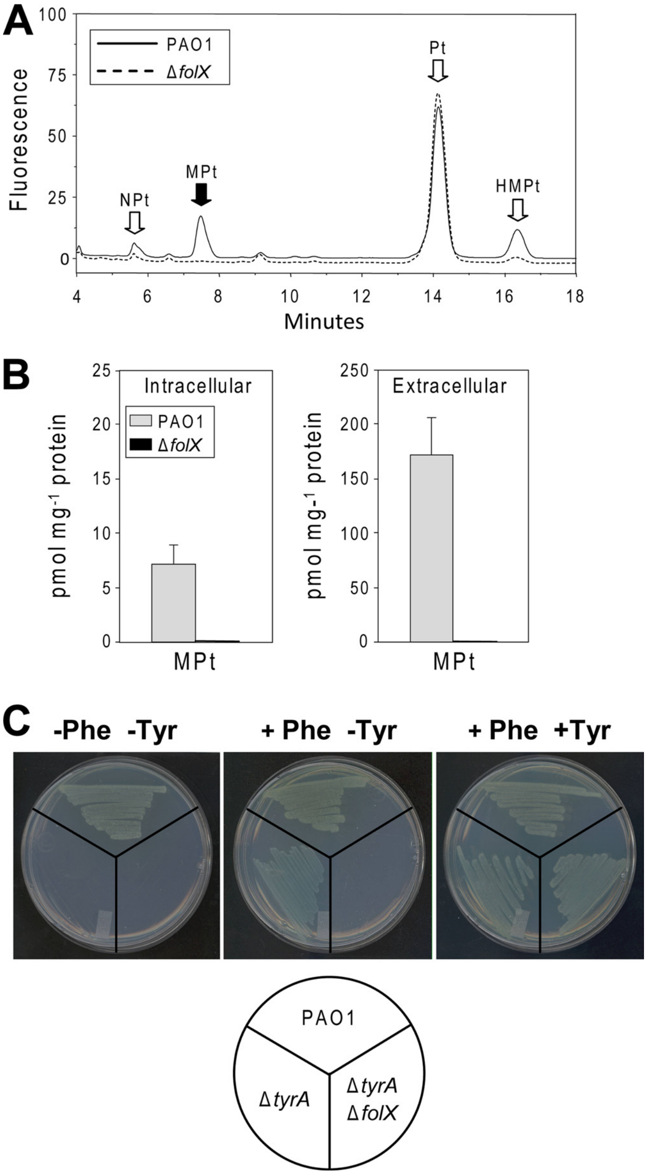

FIG. 5. Genetic evidence implicating *folX* in tetrahydromonapterin synthesis in *P. aeruginosa* and supporting the tetrahydromonapterin requirement of *P. aeruginosa* PhhA *in situ*. (A) Fluorometric HPLC analysis of pterins extracted from wild-type (PAO1) and Δ*folX* strains grown to an A_{600} of 0.6 in liquid M9 medium supplemented with 0.4% glucose. Note the loss of the monapterin peak in the Δ*folX* strain. Abbreviations are the same as those in the legend to Fig. 3A. (B) Quantitation of intra- and extracellular monapterin (per mg cellular protein) in wild-type and Δ*folX* strains grown to an A_{600} of 0.6 in the medium described for panel A. Data are means and standard errors from three replicates. (C) Abolition of the tyrosine prototrophy of a Δ*tyr*A strain by the deletion of *folX*. Wild-type *P. aeruginosa* was included as a control. Plates contained M9 medium supplemented with 0.4% glucose, 0.1% arabinose, 50 µg/ml L-tryptophan, 10 µM *p*-aminobenzoate, 10 µM 4-hydroxybenzoate, and 10 µM 2,3-dihydroxybenzoate, with or without 50 µg/ml L-phenylalanine and 54 µg/ml L-tyrosine. Note that the Δ*tyr*A strain requires L-phenylalanine as well as L-tryptophan and *p*-aminobenzoate, because *P. aeruginosa* TyrA is a bifunctional cyclohexadienyl dehydrogenase/5-enolpyruvylshikimate-3-phosphate synthase whose ablation eliminates the synthesis of chorismate as well as that of tyrosine.

culture medium of *Serratia marcescens* (synonym, *S. indica*)(22, 24). The scale of pterin production in *E. coli* and *S. marcescens* far exceeds that of folates, although the latter usually are considered the main end products of pterin biosynthesis (Fig. 3B) (22). In *E. coli* at least, pterin production peaks as log-phase growth ends (40), and *folX* expression is 39-fold higher in stationary-phase cells than in mid-log-phase cells (18). It therefore seems likely that a major tetrahydropterin-dependent process remains to be discovered in these bacteria, that this process is extracellular, and that it is most active when growth ceases. In this connection, it may be relevant that the FolM homolog PTR1 of the parasitic protist *Leishmania major*

Downloaded from http://jb.asm.org/ on December 1, 2020 by guest

A

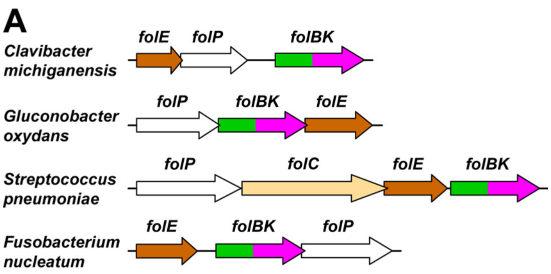

B

Gene absent | + Gene present | + Gene present and clustered

Species	folM	folB	folX
Colwellia psychrerythraea	+	+	+
Saccharophagus degradans	+	+	
Pseudoalteromonas haloplanktis	+	+	+
Shewanella frigidimarina	+	+	+
Methylococcus capsulatus	+	+	
Alcanivorax borkumensis	+	+	
Azotobacter vinelandii	+	+	+
Cellvibrio japonicus	+	+	
Pseudomonas aeruginosa	+	+	+
Pseudomonas mendocina	+	+	+
Pseudomonas fluorescens	+	+	+
Pseudomonas syringae	+	+	+
Vibrio sp. MED222	+	+	+
Vibrio splendidus	+	+	+
Kangiella koreensis	+	+	
Reinekea sp. MED297	+	+	+

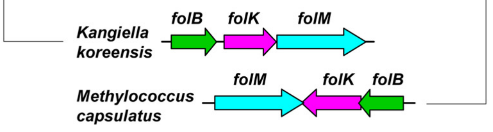

FIG. 6. Comparative genomic evidence bearing on possible metabolic channeling between FolB and FolK and on the possible substrate for FolM in bacteria without FolX. (A) The widespread occurrence in folate synthesis gene clusters of *folB-folK* fusion genes. The species shown represent four diverse phyla (*Actinobacteria*, *Alphaproteobacteria*, *Firmicutes*, and *Fusobacteria*). (B) Clustering patterns of *folM* genes in *Gammaproteobacteria* with or without *folX* genes. Note that *folM-folB* clusters occur only if *folX* is absent. The *folM-folB* clusters also include *folK*, as illustrated by two representative examples (*Kangiella koreensis* and *Methylococcus capsulatus*).

has an important (but biochemically undefined) role in oxidative stress resistance that depends upon the reduction of H_2-BPt to H_4-BPt (28).

ACKNOWLEDGMENTS

This work was supported by the National Research Initiative of the U.S. Department of Agriculture Cooperative State Research, Education, and Extension Service (grant no. 2008-35318-04589 to A.D.H.), by the National Institutes of Health (grant no. R01 GM70641-01 to V.C.-L.), and by an endowment from the C. V. Griffin Sr. Foundation.

We thank M. Mevarech (Tel Aviv University, Israel) for pFolM, S. Jin (University of Florida) for *P. aeruginosa* PAO1 and the pEX18 vectors, and H. Yu (Marshall University, Huntington, WV) for pHERD20T.

REFERENCES

1. **Ahn, C., J. Byun, and J. Yim.** 1997. Purification, cloning, and functional expression of dihydroneopterin triphosphate 2′-epimerase from *Escherichia coli*. J. Biol. Chem. **272:**15323–15328.
2. **Baba, T., T. Ara, M. Hasegawa, Y. Takai, Y. Okumura, M. Baba, K. A. Datsenko, M. Tomita, B. L. Wanner, and H. Mori.** 2006. Construction of *Escherichia coli* K-12 in-frame, single-gene knockout mutants: the Keio collection. Mol. Syst. Biol. **2:**2006.0008.
3. **Bagley, P. J., and J. Selhub.** 2000. Analysis of folate form distribution by affinity followed by reversed-phase chromatography with electrochemical detection. Clin. Chem. **46:**404–411.
4. **Blakley, R. L.** 1969. Chemical and physical properties of pterins and folate derivatives, p. 58–105. *In* R. L. Blakley (ed.), The biochemistry of folic acid and related pteridines. Wiley, New York, NY.
5. **Blaszczyk, J., Y. Li, J. Gan, H. Yan, and X. Ji.** 2007. Structural basis for the aldolase and epimerase activities of *Staphylococcus aureus* dihydroneopterin aldolase. J. Mol. Biol. **368:**161–169.
6. **Bradford, M. M.** 1976. A rapid and sensitive method for the quantitation of microgram quantities of protein utilizing the principle of protein-dye binding. Anal. Biochem. **72:**248–254.
7. **Choi, K.-H., A. Kumar, and H. P. Schweizer.** 2006. A 10 min method for preparation of highly electrocompetent *Pseudomonas aeruginosa* cells: application for DNA fragment transfer between chromosomes and plasmid transformation. J. Microbiol. Methods **64:**391–397.
8. **Datsenko, K. A., and B. L. Wanner.** 2000. One-step inactivation of chromosomal genes in *Escherichia coli* K-12 using PCR products. Proc. Natl. Acad. Sci. USA **97:**6640–6645.
9. **de Crécy-Lagard, V., B. El Yacoubi, R. D. de la Garza, A. Noiriel, and A. D. Hanson.** 2007. Comparative genomics of bacterial and plant folate synthesis and salvage: predictions and validations. BMC Genomics **8:**245.
10. **Dykxhoorn, D. M., R. St. Pierre, and T. Linn.** 1996. A set of compatible *tac* promoter expression vectors. Gene **177:**133–136.
11. **Fernandez, R. F., E. Dolghih, and D. A. Kunz.** 2004. Enzymatic assimilation of cyanide via pterin-dependent oxygenolytic cleavage to ammonia and formate in *Pseudomonas fluorescens* NCIMB 11764. Appl. Environ. Microbiol. **70:**121–128.
12. **Forrest, H. S., and C. Van Baalen.** 1970. Microbiology of unconjugated pteridines. Annu. Rev. Microbiol. **24:**91–108.
13. **Fujisawa, H., and H. Nakata.** 1987. Phenylalanine 4-monooxygenase from *Chromobacterium violaceum*. Methods Enzymol. **142:**44–49.
14. **Giladi, M., N. Altman-Price, I. Levin, L. Levy, and M. Mevarech.** 2003. FolM, a new chromosomally encoded dihydrofolate reductase in *Escherichia coli*. J. Bacteriol. **185:**7015–7018.
15. **Gourley, D. G., A. W. Schüttelkopf, G. A. Leonard, J. Luba, L. W. Hardy, S. M. Beverley, and W. N. Hunter.** 2001. Pteridine reductase mechanism correlates pterin metabolism with drug resistance in trypanosomatid parasites. Nat. Struct. Biol. **8:**521–525.
16. **Gregory, J. F., and J. P. Toth.** 1988. Chemical synthesis of deuterated folate monoglutamate and in vivo assessment of urinary excretion of deuterated folates in man. Anal. Biochem. **170:**94–104.
17. **Guroff, G., and C. A. Rhoads.** 1969. Phenylalanine hydroxylation by *Pseudomonas* species (ATCC 11299a). Nature of the cofactor. J. Biol. Chem. **244:** 142–146.
18. **Gutiérrez-Ríos, R. M., D. A. Rosenblueth, J. A. Loza, A. M. Huerta, J. D. Glasner, F. R. Blattner, and J. Collado-Vides.** 2003. Regulatory network of *Escherichia coli*: consistency between literature knowledge and microarray profiles. Genome Res. **13:**2435–2443.
19. **Haussmann, C., F. Rohdich, E. Schmidt, A. Bacher, and G. Richter.** 1998. Biosynthesis of pteridines in *Escherichia coli*. Structural and mechanistic similarity of dihydroneopterin-triphosphate epimerase and dihydroneopterin aldolase. J. Biol. Chem. **273:**17418–17424.
20. **Hoang, T. T., R. R. Karkhoff-Schweizer, A. J. Kutchma, and H. P. Schweizer.** 1998. A broad-host-range Flp-*FRT* recombination system for site-specific excision of chromosomally-located DNA sequences: application for isolation of unmarked *Pseudomonas aeruginosa* mutants. Gene **212:**77–86.
21. **Ikemoto, K., T. Sugimoto, S. Murata, M. Tazawa, T. Nomura, H. Ichinose, and T. Nagatsu.** 2002. (6R)-5,6,7,8-Tetrahydro-L-monapterin from *Escherichia coli*, a novel natural unconjugated tetrahydropterin. Biol. Chem. **383:** 325–330.
22. **Iwai, K., M. Kobashi, and H. Fujisawa.** 1970. Occurrence of *Crithidia* factors and folic acid in various bacteria. J. Bacteriol. **104:**197–201.
23. **Kaufman, S.** 1961. The nature of the primary oxidation product formed from tetrahydropteridines during phenylalanine hydroxylation. J. Biol. Chem. **236:** 804–810.
24. **Kobashi, M., and K. Iwai.** 1971. Biochemical studies on naturally occurring pteridines. II. Isolation and characterization of L-threoneopterin as the *Crithidia* factor, and on isoxanthopterin produced by *Serratia indica*. Agric. Biol. Chem. **35:**47–52.
25. **Kong, J. S., J. Y. Kang, H. L. Kim, O. S. Kwon, K. H. Lee, and Y. S. Park.** 2006. 6-Pyruvoyltetrahydropterin synthase orthologs of either a single or dual domain structure are responsible for tetrahydrobiopterin synthesis in bacteria. FEBS Lett. **580:**4900–4904.
26. **Lye, L. F., M. L. Cunningham, and S. M. Beverley.** 2002. Characterization of quinonoid-dihydropteridine reductase (QDPR) from the lower eukaryote *Leishmania major*. J. Biol. Chem. **277:**38245–38253.
27. **Naponelli, V., A. Noiriel, M. J. Ziemak, S. M. Beverley, L. F. Lye, A. M.**

Downloaded from http://jb.asm.org/ on December 1, 2020 by guest

Plume, J. R. Botella, K. Loizeau, S. Ravanel, F. Rébeillé, V. de Crécy-Lagard, and A. D. Hanson. 2008. Phylogenomic and functional analysis of pterin-4a-carbinolamine dehydratase family (COG2154) proteins in plants and microorganisms. Plant Physiol. **146:**1515–1527.

28. **Nare, B., L. A. Garraway, T. J. Vickers, and S. M. Beverley.** 2009. *PTR1*-dependent synthesis of tetrahydrobiopterin contributes to oxidant susceptibility in the trypanosomatid protozoan parasite *Leishmania major*. Curr. Genet. **55:**287–299.
29. **Noiriel, A., V. Naponelli, J. F. Gregory III, and A. D. Hanson.** 2007. Pterin and folate salvage. Plants and *Escherichia coli* lack capacity to reduce oxidized pterins. Plant Physiol. **143:**1101–1109.
30. **Ohmae, E., Y. Fukumizu, M. Iwakura, and K. Gekko.** 2005. Effects of mutation at methionine-42 of *Escherichia coli* dihydrofolate reductase on stability and function: implication of hydrophobic interactions. J. Biochem. **137:**643–652.
31. **Overbeek, R., T. Begley, R. M. Butler, J. V. Choudhuri, H. Y. Chuang, M. Cohoon, V. de Crécy-Lagard, N. Diaz, T. Disz, R. Edwards, M. Fonstein, E. D. Frank, S. Gerdes, E. M. Glass, A. Goesmann, A. Hanson, D. Iwata-Reuyl, R. Jensen, N. Jamshidi, L. Krause, M. Kubal, N. Larsen, B. Linke, A. C. McHardy, F. Meyer, H. Neuweger, G. Olsen, R. Olson, A. Osterman, V. Portnoy, G. D. Pusch, D. A. Rodionov, C. Ruckert, J. Steiner, R. Stevens, I. Thiele, O. Vassieva, Y. Ye, O. Zagnitko, and V. Vonstein.** 2005. The subsystems approach to genome annotation and its use in the project to annotate 1,000 genomes. Nucleic Acids Res. **33:**5691–5702.
32. **Pfleiderer, W.** 1985. Chemistry of naturally occurring pteridines, p. 43–114. *In* R. L. Blakley, and S. J. Benkovic (ed.), Folates and pterins, vol. 2. Wiley, New York, NY.
33. **Qiu, D., F. H. Damron, T. Mima, H. P. Schweizer, and H. D. Yu.** 2008. P_{BAD}-based shuttle vectors for functional analysis of toxic and highly regulated genes in *Pseudomonas* and *Burkholderia* spp. and other bacteria. Appl. Environ. Microbiol. **74:**7422–7426.
34. **Rembold, H., and R. Gyure.** 1972. Biochemistry of the pteridines. Angew. Chem. Int. Ed. Engl. **11:**1061–1072.
35. **Sambrook, J., E. F. Fritsch, and T. Maniatis.** 1982. Molecular cloning: a laboratory manual. Cold Spring Harbor Laboratory, Cold Spring Harbor, NY.
36. **Shaw, D., J. D. Odom, and R. B. Dunlap.** 1999. High expression and steady-state kinetic characterization of methionine site-directed mutants of *Escherichia coli* methionyl- and selenomethionyl-dihydrofolate reductase. Biochim. Biophys. Acta **1429:**401–410.
37. **Song, J., T. Xia, and R. A. Jensen.** 1999. PhhB, a *Pseudomonas aeruginosa* homolog of mammalian pterin 4a-carbinolamine dehydratase/DCoH, does not regulate expression of phenylalanine hydroxylase at the transcriptional level. J. Bacteriol. **181:**2789–2796.
38. **Thöny, B., G. Auerbach, and N. Blau.** 2000. Tetrahydrobiopterin biosynthesis, regeneration and functions. Biochem. J. **347:**1–16.
39. **Vasudevan, S. G., D. C. Shaw, and W. L. Armarego.** 1988. Dihydropteridine reductase from *Escherichia coli*. Biochem. J. **255:**581–588.
40. **Wachter, H., A. Hausen, E. Reider, and M. Schweiger.** 1980. Pteridine excretion from cells as indicator of cell proliferation. Naturwissenschaften **67:**610–611.
41. **Zhao, G., T. Xia, J. Song, and R. A. Jensen.** 1994. *Pseudomonas aeruginosa* possesses homologues of mammalian phenylalanine hydroxylase and 4 alpha-carbinolamine dehydratase/DCoH as part of a three-component gene cluster. Proc. Natl. Acad. Sci. USA **91:**1366–1370.

Downloaded from http://jb.asm.org/ on December 1, 2020 by guest
