# Supplementary material for: 5-Hydroxytryptophan (5-HTP): Natural Occurrence, Analysis, Biosynthesis, Biotechnology, Physiology and Toxicology
Source: Int J Mol Sci. 2020 Dec 26;22(1):181. doi: 10.3390/ijms22010181 (PMC7796270; doi:10.3390/ijms22010181)
Supplement: Supplementary file 1 [file ijms-22-00181-s001.zip › 5-HTP.Data/PDF/3560911546/Hanna-2007-Synthesis-of-some-tropane-derivativ.pdf]

Available online at www.sciencedirect.com
ScienceDirect
Bioorganic & Medicinal Chemistry 15 (2007) 7765–7772
Bioorganic & Medicinal Chemistry

Synthesis of some tropane derivatives of anticipated activity on the reuptake of norepinephrine and/or serotonin

Mona M. Hanna,* Nahed M. Eid, Riham F. George and Hani M. Safwat

Pharmaceutical Chemistry Department, Faculty of Pharmacy, Cairo University, Cairo, Egypt

Received 4 April 2007; revised 21 August 2007; accepted 28 August 2007
Available online 1 September 2007

Abstract—A variety of tropane derivatives **14a–g** were prepared via the reaction of the alcohol analogs **12a** and **12b** with substituted fluorobenzenes **13a–f**. The prepared compounds were tested for their activity and selectivity toward the norepinephrine transporter (NET) and serotonin transporter (SERT) using yohimbine-induced mortality and 5-hydroxytryptophan-induced neurotoxicity in mice, respectively. All the tested compounds were found to be NE and 5-HT reuptake inhibitors except **14d** which exhibited selective 5-HT reuptake inhibition activity.
© 2007 Elsevier Ltd. All rights reserved.

1. Introduction

Major depression is one of the most common psychiatric disorders. By 2020 it will be expected that depression will be the second most serious medical condition with respect to global disease burden.[1] Over the last two decades, a new generation of antidepressant resulted from the discovery of selective serotonin-reuptake inhibitors (SSRIs) such as fluoxetine (Prozac®) **1** and paroxetine (Paxil®) **2**.[2] Although SSRIs are very effective antidepressants with substantially fewer side effects than the older tricyclic antidepressants (TCAs), they are not universally effective and can also have bothersome side effects of their own, such as anxiety, sleep disturbance, weight gain, sexual dysfunction, and gastrointestinal disturbances.[3] In addition, they have a delayed onset of antidepressant effect that is attributed to the action of released serotonin (5-HT) on the presynaptic receptor 5-HT_{1A} leading to feed back inhibition of 5-HT release, then by time the adaptive changes and desensitization of this receptor result in the antidepressant effect.[2,4] It is reported that potent and selective 5-HT and norepinephrine (NE) reuptake inhibitors desensitize, respectively, terminal autoreceptors and α_2 adrenergic heteroreceptors on 5-HT fibers, that it can be argued that the combination of these two treatments might result in a greater increase in the 5-HT neurotransmission than either treatment alone, and thereby, achieve a greater antidepressant efficacy.[5] Therefore, the dual NET/SERT reuptake inhibitors, venlafaxine (Effexor®) **3**[6,7] and duloxetine (Cymbalta®) **4**,[8–10] have been introduced, representing a new class of antidepressants with a higher rate of efficacy and fewer side effects than TCAs and SSRIs. Furthermore, the venlafaxine analogs PRC025 and PRC050 **5a** and **5b** have demonstrated a higher affinity than venlafaxine itself for all human monoamine transporters (norepinephrine, serotonin, and dopamine transporters).[11] Moreover, different non-selective constrained amines have been prepared aiming to increase their onset of antidepressant effect such as DOV 21,947 **6** which is found to have a high affinity to SERT and NET.[12] In addition, compound **7** has a potent and rapid antidepressant activity as it acts as non-selective monoamine reuptake inhibitor.[13] In the literature, several tropane derivatives have been prepared and tested for their selective binding to SERT and/or NET. The substituted 3-phenoxynortropane **8** has been prepared and tested for its antidepressant activity by the L-5-hydroxytryptophan potentiation assay, it has ED_{50} 8.4 mg/kg.[14] Structural modifications result in increased affinity to both SERT and NET as represented by **9a–c**.[15,16] Recently, the conformationally constrained tricyclic tropane analogs **10a–d** represent a new class of selective drugs at SERT and NET;[17–20] it is important to note that **10d** is a potent and a dual SERT/NET inhibitor with K_i values around 1 nM

Keywords: Tropinone; 3-Phenyltropines; Antidepressant; Serotonin; Norepinephrine.
* Corresponding author. Tel.: +20 202 6242769; e-mail: mona_maurice@yahoo.com

0968-0896/$ - see front matter © 2007 Elsevier Ltd. All rights reserved.
doi:10.1016/j.bmc.2007.08.055

having a selectivity profile like venlafaxine **3** and duloxetine **4**.[20] In the light of these facts, it deemed of interest to rigidify the skeleton of fluoxetine into a tropane structure retaining the phenoxy and phenyl moieties of fluoxetine, to study the effect of this conformation-lock on the activity and selectivity toward norepinephrine and serotonin-reuptake blockade. Furthermore, in fluoxetine, the *para* CF_3 group is essential for the activity and selectivity,[21] therefore, in the present work, it is intended to investigate synthesis of novel tropane containing compounds with different electron deficient groups attached on the phenoxy moiety and evaluate their NE and 5-HT reuptake inhibition activity, aiming to produce safer, faster acting, and better tolerated antidepressants that overcome the problem of suicidality in patients being treated with other antidepressants.

tion of tropinone **11** to the ethereal solution of phenyllithium as previously reported.[22] However, compound **12b** was prepared by the addition of tropinone **11** to ethereal solution of *n*-butyllithium treated with 4-fluorobromobenzene **13e** according to the reported procedure.[23] Compounds **14a–g** were prepared from their precursor alcohols **12a** and **12b** by the action of sodium hydride in dry DMF to form the corresponding sodium salt.[16] The substituted fluorobenzene derivatives **13a–f** were then added to obtain the required compounds through aromatic nucleophilic substitution reaction.[24] The temperature of the reaction was varied according to the electro-negativity of the substituent at the fluorobenzene. Thus, for the preparation of compound **14a** the reaction was conducted at −70 °C,[25] as the 4-fluoronitrobenzene **13a** is the most reactive reagent. Trials to

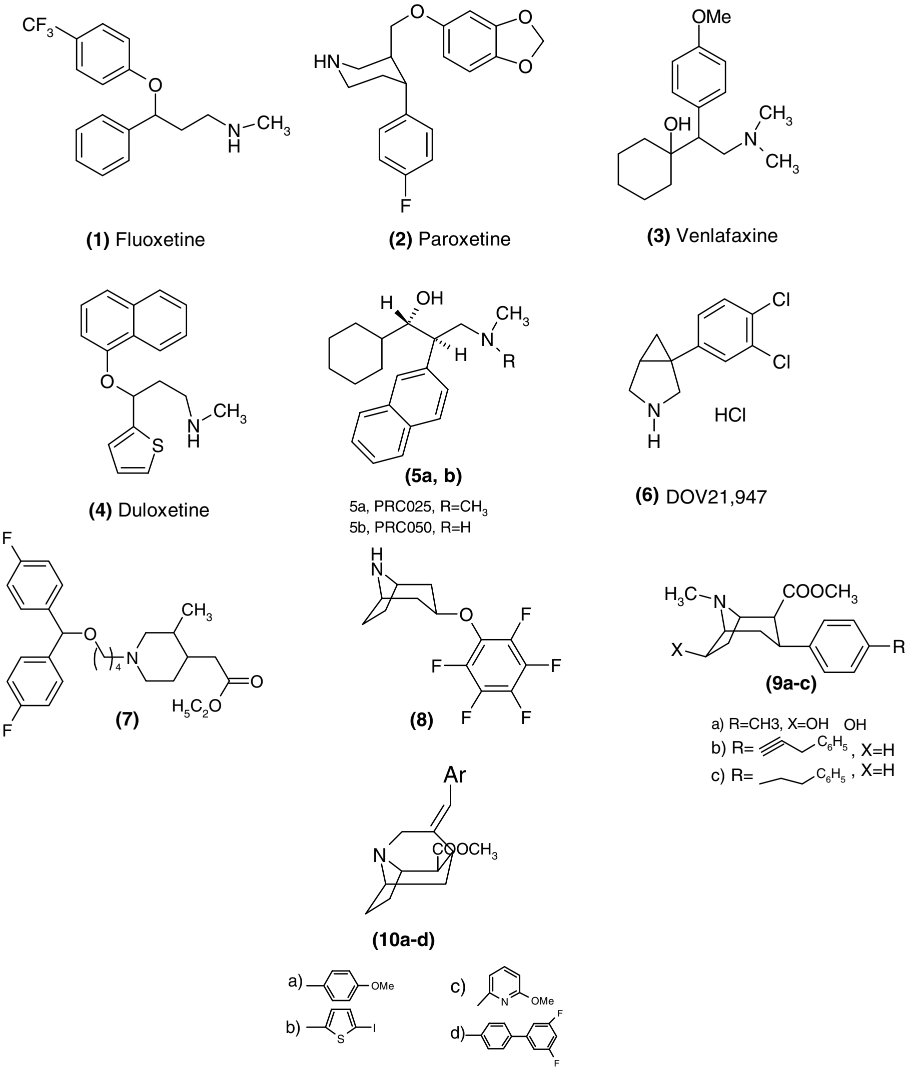

2. Results and discussion

2.1. Chemistry

The target compounds were prepared according to Scheme 1, where the alcohol **12a** was obtained by addi-

achieve the reaction at higher temperatures −15 °C and room temperature[26] failed to give the target compound. Likewise, the reaction temperature and reaction time adopted for the preparation of compounds **14b–g** were determined according to the reactivity of the reagent. IR spectra of compounds **14a–g** showed the

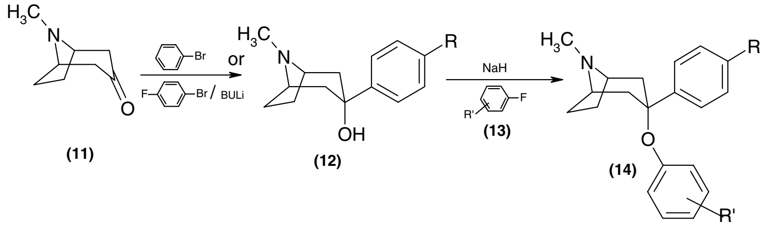

13a, R = 4-NO_2
13b, R = 4-CN
13c, R = 4-CF_3
13d, R = 3-CF_3
13e, R = 4-Br
13f, R = 4-Cl

14a, R = H, R' = 4-NO_2
14b, R = H, R' = 4-CN
14c, R = H, R' = 4-CF_3
14d, R = H, R' = 3-CF_3
14e, R = H, R' = 4-Br
14f, R = H, R' = 4-Cl
14g, R = F, R' = 4-CN

Scheme 1.

disappearance of the OH band of the corresponding parent alcohols, and the presence of a nitrile stretching vibrational band at 2226–2223 cm^{-1} in case of **14b** and **14g**. Additionally, all compounds were confirmed by ^{1}H NMR spectra that revealed the presence of additional aromatic protons with respect to the alcohol precursors **12a** and **12b** besides the expected tropane protons. Single crystal X-ray diffraction studies of **14a** (Fig. 1) revealed the appearance of *endo* configuration. In addition the piperidinyl function of the tropane moiety adopted a distorted chair form configuration.

2.2. Pharmacological screening

All compounds were tested for their serotonin-reuptake inhibition by measuring potentiation of 5-hydroxytryptophan (5-HTP)-induced neurotoxicity in mice. They also were tested for their norepinephrine reuptake inhibition by measuring yohimbine-induced mortality. Fluoxetine and citalopram were used as standard drugs with selective serotonin reuptake inhibitory effect, clomipramine was used as non-selective drug with 5-HT and NE reuptake inhibitory effects. The observed data were summarized in Table 1 which show the response% of the tested animals showing positive response (mice head twitches) to the total number of mice in each group and Table 2 which shows the response% of the tested animals showing positive response (mice death) to the total number of mice in each group. The doses were calculated in mM/kg and administered intraperitoneally (ip). ED_{50} 5-HT/ED_{50} NE ratio was calculated (Table 3) as a parameter for selectivity. Therefore, if the value of this ratio is significantly less than one, the compound will be SSRI, and if it is non-significantly different from one, the compound will be non-selective 5-HT/NE reuptake inhibitor.

From the obtained data (Tables 1–3 and Fig. 2), all compounds were found to inhibit the reuptake of 5-HT and NE except **14d**, that showed selectivity toward

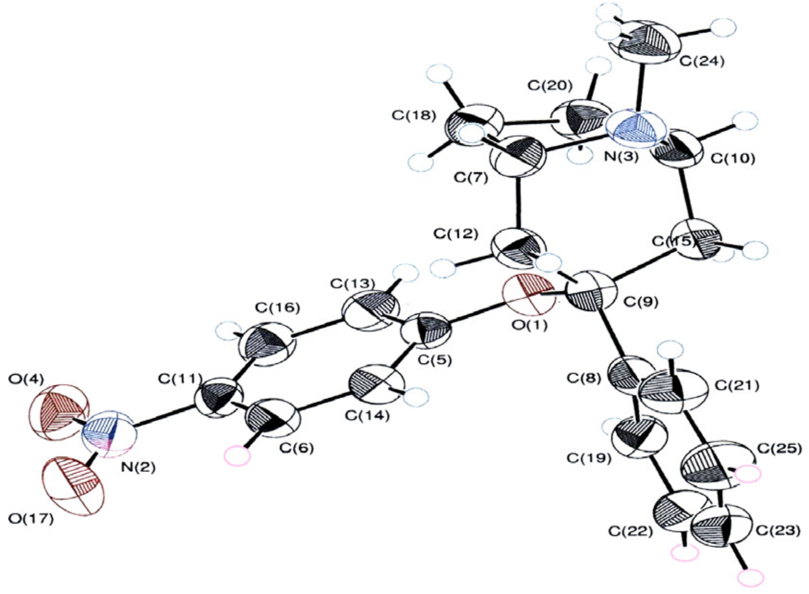

Figure 1. Ortep preview of compound **14a**.

Table 1. Response% of the tested compounds (mM/kg) toward potentiation of 5-HTP induced neurotoxicity

Compound	Response%[a] of the tested animals towards dose (mM/kg)					
	1.62	3.24	4.86	6.47	12.94	25.89
Fluoxetine	NT[b]	16.67%	NT	50%	100%	NT
Clomipramine	50%	50%	NT	83.33%	NT	NT
Citalopram	50%	83.33%	NT	100%	NT	NT
**14a**	33.33%	50%	66.67%	Toxic	NT	NT
**14b**	16.67%	33.33%	NT	66.67%	NT	NT
**14c**	NT	50%	NT	66.67%	83.33%	NT
**14d**	NT	33.33%	NT	50%	83.33%	NT
**14e**	NT	33.33%	NT	50%	66.67%	NT
**14f**	NT	33.33%	NT	66.67%	83.33%	NT
**14g**	33.33%	50%	66.67%	Toxic	NT	NT

[a] Response% of the tested animals showing positive response (mice head twitches) to the total number of mice in each group.
[b] NT, not tested.

Table 2. Response% of the tested compounds (mM/kg) toward potentiation of yohimbine induced mortality

Compound	Response%[a] of the tested animals towards dose (mM/kg)							
	1.62	3.24	4.86	6.47	9.71	12.94	19.42	25.89
Fluoxetine	NT[b]	0%	NT	0%	NT	0%	NT	NT
Clomipramine	NT	16.67%	NT	50%	66.67%	NT	NT	NT
Citalopram	NT	NT	NT	0%	NT	16.67%	NT	83.33%
**14a**	NT	0%	NT	50%	NT	50%	NT	Toxic
**14b**	NT	50%	NT	50%	NT	83.33%	NT	NT
**14c**	NT	NT	NT	0%	NT	66.67%	66.67%	100%
**14d**	NT	0%	NT	16.67%	NT	33.33%	Toxic	Toxic
**14e**	NT	NT	NT	16.67%	NT	50%	NT	100%
**14f**	NT	NT	NT	33.33%	NT	33.33%	NT	66.67%
**14g**	NT	16.67%	NT	66.67%	NT	100%	NT	NT

[a] Response% of the tested animals showing positive response (mice death) to the total number of mice in each group.
[b] NT, not tested.

Table 3. Potency (ED_{50}) in potentiation of 5-HTP induced neurotoxicity, yohimbine induced mortality and the 5-HT/NE selectivity ratio of the tested compounds

Compound	ED_{50} (mM/kg) 5-HTP	ED_{50} (mM/kg) NE	5-HT/NE selectivity
Fluoxetine	5.09	>12.90	<0.39
Clomipramine	2.00	5.78	0.35
Citalopram	1.58	13.73	0.12[a]
**14a**	2.94	10.27	0.29
**14b**	4.44	4.08	1.08
**14c**	3.30	11.27	0.29
**14d**	5.46	53.13	0.10[a]
**14e**	6.47	11.41	0.56
**14f**	4.72	16.06	0.29
**14g**	2.94	5.02	0.59

[a] Significantly different from one at $p < 0.05$.

5-HT reuptake inhibition. This suggests that the rigidification of fluoxetine structure resulted in loss of selectivity toward SERT as compound **14c** showed both NE and 5-HT reuptake inhibitory activity. A hypothetical explanation for this shift in activity is that the rigidification of fluoxetine structure into tropane nucleus resulted in reduction of the distance between the *para* substitution and the nitrogen of the bridge which may facilitate the binding of the derivatives to both transporters. On the other hand, the presence of CF_3 group at *meta*- rather than *para*-position **14d** restored the selectivity towards SERT. Also, there was no significant difference between **14e** and **14f** in potency and selectivity, indicating that the *para*-position can accommodate a bulky moiety without alteration in activity or selectivity. Furthermore, the electronegativity did not affect the activity or selectivity as all *para*-substituted derivatives showed comparable activity to clomipramine. Moreover, introduction of the *para* fluorine moiety at the phenyl ring of **14b** to produce **14g** did not affect neither selectivity nor activity. Additionally, although compound **14g** was non-selective, its effect on 5-HT reuptake inhibition did not differ significantly from that of citalopram.

In conclusion, it could be assumed that, all the *para*-substituted derivatives adopted in the present study exerted non-selective 5-HT and NE reuptake inhibition regardless of the electronegativity or the size of the *para* substituent. The *meta* substituted derivative **14d** had selective 5-HT reuptake inhibition activity. Introduction of the *para* fluorine moiety at the phenyl ring of **14b** to produce **14g** did not affect the selectivity or activity.

3. Experimental

Melting points were determined on Electrothermal 9100 digital melting point apparatus and were uncorrected. Elemental microanalyses were performed at the microanalytical center, Faculty of Science, Cairo University.

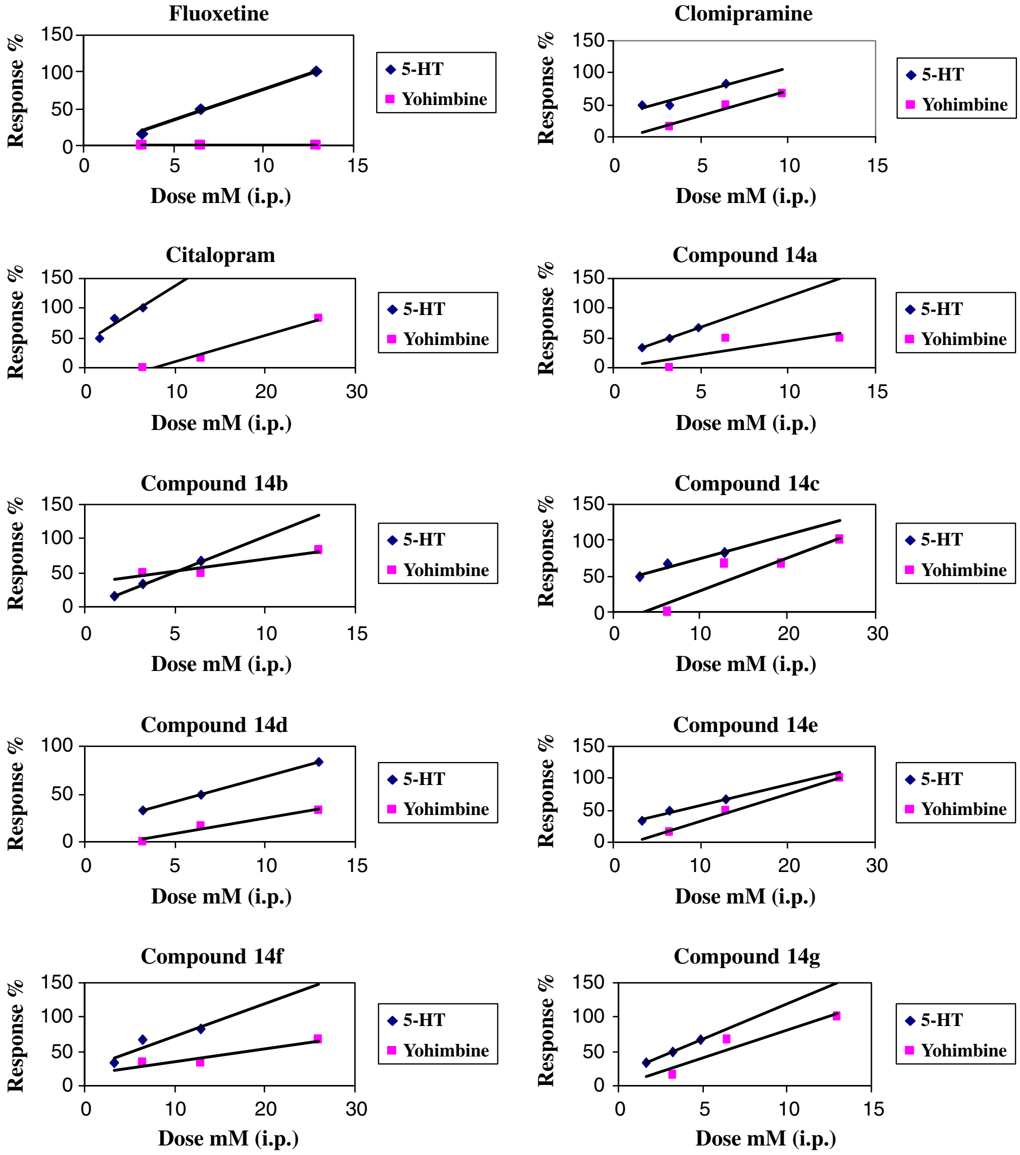

Figure 2. Dose–response% curves showing pharmacological activity of the tested compounds in potentiation of 5-HTP induced neurotoxicity and yohimbine induced mortality.

The IR spectra were recorded on a Bruker FT-IR spectrophotometer as potassium bromide discs in case of solids and as thin films in case of oils. The ^1H NMR spectra were recorded in $CDCl_3$ on a Varian Mercury spectrometer (300 MHz). Mass spectra were performed on HP MODEL: MS_5988 mass spectrometer (EI, 70 eV). All compounds **14a–g** were purified by column chromatography using silica gel 60 (Fluka, 70–230 mesh) as stationary phase and chloroform/methanol (10:1 v/v) as mobile phase. Thin layer chromatography was carried out on silica gel TLC plates with fluorescence indicator (F_{254}). Tropinone **11**,[27,28] 3-phenyltropine **12a**,[22] and 3-(*p*-fluorophenyl)tropine **12b**[23] were prepared according to the previously reported procedures.

3.1. General procedure for preparation of 14a–g

To a suspension of sodium hydride (0.14 g; 60% in mineral oil, washed with *n*-hexane) in dry DMF (1.5 ml), a suspension of the corresponding alcohol **12a** or **12b** (2.3 mmol) in dry DMF (5 ml) was added. The mixture was stirred at 80 °C for 1 h. Then the reaction mixture was cooled to the specified temperature where a solution of the appropriate substituted fluorobenzene **13a–f** (2.88 mmol) in dry DMF (2 ml) was added dropwise. After stirring for the specified period of time, the reaction was allowed to reach the room temperature and poured on ice water (25 ml), stirred for 5 min, and extracted with chloroform (3× 30 ml). The extracts were dried over anhydrous magnesium sulphate, filtered and evaporated under vacuum leaving an oily residue. Purification by column chromatography and evaporation of the eluate under vacuum resulted in separation of **14a–g** in pure form.

3.1.1. 8-Methyl-3-(4′-nitrophenoxy)-3-phenyl-8-azabicyclo[3.2.1]octane (14a).

Compound **14a** was prepared from **12a** and 4-fluoronitrobenzene **13a** at −70 °C for

2 h. Colorless crystals, from methanol mp 128 °C, yield 40%. IR: υ_{max}/cm^{-1} 3025 (CH aromatic), 2995–2800 (CH aliphatic), 1591, 1342 (NO_2 group). ^1H NMR: δ 2.11–2.21 (m, 4H, tropane), 2.41–2.47 (m, 2H, tropane), 2.49 (s, N-CH_3), 2.72–2.78 (d, $J = 16.5$ Hz, 2H, tropane), 3.42 (s, 2H, tropane at *C*-1 and *C*-5), 6.61–6.64 (d, $J = 9$ Hz, 2H, aromatic protons C′-2, C′-6), 7.26–7.45 (m, 5H of the phenyl ring), 8.00–8.03 (d, $J = 9$ Hz, 2H, aromatic protons C′-3, C′-5) MS: *m*/*z* (%) 338 (M^+, 3), 201 (16), 200 (100), 170 (8), 94 (2), 82 (28), 57 (1). Anal. Calcd for $C_{20}H_{22}N_2O_3$ (338.41): C, 70.98; H, 6.55; N, 8.28. Found: C, 71; H, 6.66; N, 8.26.

3.1.2. 3-(4′-Cyanophenoxy)-8-methyl-3-phenyl-8-azabicyclo[3.2.1]octane (14b). Compound **14b** was prepared from **12a** and 4-fluorobenzonitrile **13b** at room temperature and stirring overnight. Brown crystals purified by column chromatography mp 91–93 °C, yield 30%. IR: υ_{max}/cm^{-1} 3059 (CH aromatic), 2928–2855 (CH aliphatic), 2223 (CN group). ^1H NMR: δ 1.99–2.07 (m, 4H, tropane), 2.31–2.46 (m, 7H, tropane protons including N-CH_3), 3.23 (s, 2H, tropane at *C*-1 and *C*-5), 6.58–6.61 (d, $J = 9$ Hz, 2H, aromatic protons C′-2, C′-6), 7.24–7.39 (m, 5H, phenyl ring and C′-3, 5); MS: *m*/*z* (%) 318 (M, 1), 201 (16), 200 (100), 170 (10), 118 (3), 94 (4), 82 (32), 57 (13). Anal. Calcd for $C_{21}H_{22}N_2O$ (318.42): C, 79.21; H, 6.96; N, 8.8. Found: C, 79.14; H, 6.94; N, 8.52.

3.1.3. 8-Methyl-3-(4′-trifluoromethylphenoxy)-3-phenyl-8-azabicyclo[3.2.1]octane (14c). Compound **14c** was prepared from **12a** and 4-fluorobenzotrifluoride **13c** at 70 °C and stirring for 5 h. Colorless crystals purified by column chromatography mp 103–105 °C, yield 25%. IR: υ_{max}/cm^{-1} 3040 (CH aromatic), 2971–2851 (CH aliphatic). ^1H NMR: δ 2.05–2.08 (m, 2H, tropane), 2.21–2.23 (d, $J = 8$ Hz, 2 H, tropane), 2.41–2.47 (m, 5H, tropane protons including N-CH_3), 2.64–2.69 (d, $J = 15$ Hz, 2H, tropane protons), 3.37 (s, 2H, tropane at *C*-1 and *C*-5), 6.61–6.64 (d, $J = 9$ Hz, 2H, aromatic protons C′-2, C′-6), 7.25–7.47 (m, 7H, aromatic protons). MS: *m*/*z* (%) 361 (M, 2), 201 (17), 200 (100), 170 (11), 161 (2), 94 (4), 82(45), 57(5). Anal. Calcd for $C_{21}H_{22}F_3NO$ (361.41): C, 69.79; H, 6.14; N, 3.88. Found: C, 69.52; H, 6.38; N, 3.71.

3.1.4. 8-Methyl-3-(3′-trifluoromethylphenoxy)-3-phenyl-8-azabicyclo[3.2.1]octane (14d). Compound **14d** was prepared from **12a** and 3-fluorobenzotrifluoride **13d** at 70 °C and stirring for 12 h. Yellow crystals purified by column chromatography mp 80–83 °C, yield 20%. IR: υ_{max}/cm^{-1} 3061–3030 (CH aromatic), 2924–2852 (CH aliphatic). ^1H NMR: δ 2.03–2.09 (m, 2H, tropane protons), 2.14–2.19 (m, 2H, tropane protons), 2.37–2.41 (m, 5H, tropane protons including N-CH_3), 2.50–2.56 (m, 2H, tropane protons), 3.30 (s, 2H, tropane at *C*-1 and *C*-5), 6.60–6.62 (d, $J = 8$ Hz,1H, aromatic proton *C*-′6), 6.88 (s, 1H, aromatic proton *C*-′2), 7.06–7.09 (d, $J = 8$ Hz, 1H, aromatic proton *C*-′4), 7.13–7.18 (t, $J = 8$ Hz, 1H, *C*-′5), 7.29–7.44 (m, 5H, phenyl protons). MS: *m*/*z* (%) 361 (M, 2), 201 (18), 200 (100), 170 (9), 161 (1), 94 (3), 82 (18), 57 (2). Anal. Calcd for $C_{21}H_{22}F_3NO$ (361.41): C, 69.79; H, 6.14; N, 3.88. Found: C, 70.11; H, 5.8; N, 3.78.

3.1.5. 3-(4′-Bromophenoxy)-8-methyl-3-phenyl-8-azabicyclo[3.2.1]octane (14e). Compound **14e** was prepared from **12a** and 4-fluorobromobenzene **13e** at 70 °C and stirring for 48 h. Brown oil purified by column chromatography, yield 10%. IR: υ_{max}/cm^{-1} 3060–3030 (CH aromatic), 2925–2856 (CH aliphatic). ^1H NMR: δ 2.00–2.05 (m, 2H, tropane protons), 2.20–2.22 (m, 2H, tropane protons), 2.36–2.48 (m, 5H, tropane protons including N-CH_3), 2.55–2.60 (m,2H, tropane protons), 3.37 (s, 2H, tropane at *C*-1 and *C*-5), 6.40–6.43 (d, $J = 9$ Hz, 2H, aromatic protons C′-2, C′-6), 7.17–7.42 (m, 7H, aromatic protons). Anal. Calcd for $C_{20}H_{22}BrNO$ (372.31): C, 64.52; H, 5.95; N, 3.76; Br, 21.46. Found: C, 65.19; H, 5.8; N, 3.73; Br, 21.85.

3.1.6. 3-(4′-Chlorophenoxy)-8-methyl-3-phenyl-8-azabicyclo[3.2.1]octane (14f). Compound **14f** was prepared from **12a** and 4-fluorochlorobenzene **13f** at 70 °C and stirring for 48 h. Brown oil purified by column chromatography, yield 12%. IR: υ_{max}/cm^{-1} 3088–3022 (CH aromatic), 2927–2817 (CH aliphatic). ^1H NMR: δ 2.00–2.04 (m, 2H, tropane protons), 2.15–2.20 (m, 2H, tropane protons), 2.35–2.42 (m, 5H, tropane protons including N-CH_3), 2.49–2.55 (m, 2H, tropane protons), 3.31 (s, 2H, tropane at *C*-1 and *C*-5), 6.45–6.47 (d, $J = 7$ Hz, 2H, aromatic protons C′-2, C′-6), 7.03–7.05 (d, $J = 7$ Hz, 2H, aromatic protons C′-3, 5), 7.22–7.42 (m, 5H, phenyl protons). Anal. Calcd for $C_{20}H_{22}ClNO$ (327.86): C, 73.27; H, 6.76; N, 4.27; Cl, 10.81. Found: C, 73.29; H, 7.01; N, 4.29; Cl, 10.92.

3.1.7. 3-(4′-Cyanophenoxy)-3-(4″-fluorophenyl)-8-methylazabicyclo[3.2.1]octane (14g). Compound **14g** was prepared from **12b** and 4-fluorobenzonitrile **13b** at room temperature and stirring overnight. Yellow crystals purified by column chromatography mp 210–214 °C, yield 25%. IR: υ_{max}/cm^{-1} 3031 (CH aromatic), 2850 (CH aliphatic), 2226 (CN group). ^1H NMR: δ 2.07–2.09 (m,4H, tropane protons), 2.40–2.50 (m, 5H, tropane protons including N-CH_3), 2.68 (d, 2H, tropane protons), 3.43 (s, 2H, tropane at *C*-1 and *C*-5), 6.59–6.62 (d, $J = 9$ Hz, 2H, aromatic protons C′-2, C′-6), 6.98–7.04 (t, 2H*C*″-3, *C*″-5), 7.38–7.44 (m, 4H, aromatic protons). MS: *m*/*z* (%) 336 (M, 1), 219 (17), 218 (100), 188 (10), 118 (1), 94 (4), 82 (27), 57 (6). Anal. Calcd, for $C_{21}H_{21}FN_2O.2H_2O$: C, 67.72; H, 6.77; N, 7.52. Found: C, 68.19; H, 6.98; N, 7.46.

3.2. Single crystal X-ray crystallographic data of (14a)

Compound **14a** was recrystallized as prismatic colorless crystals from methanol. The crystallographic data were collected at $T = 298$ K on a Kappa CCD Enraf Nonius FR 590 diffractometer using a graphite monochromator with Mo-K_α radiation ($\lambda = 0.71073$ Å). The crystal structure was determined by SIR92[29] and refined by maXus[30] (Bruker Nonius, Delft and MacScience, Japan). Chemical formula $C_{20}H_{22}N_2O_3$, $M_r = 338.407$, monoclinic, crystallizes in space group $P2_1/C$, cell lengths ‘$a = 7.4365(3)$, $b = 14.0326(6)$, $c = 16.8885(9)$

Å', cell angles '$\alpha = 90.00$, $\beta = 99.271(2)$, $\gamma = 90.00°$', $V = 1739.34(14)$ Å^3, $Z = 4$, $D_c = 1.292$ mg/m^3, θ values 2.910–25.682°, absorption coefficient μ (Mo-K_α) = 0.09 mm^{-1}, $F(000)=720$. The unique reflections measured 5689 of which 1765 reflections with threshold expression $I > 3\sigma(I)$ were used in the structural analysis. Convergence for 226 variable parameters by least-squares refinement on F^2 with $w = 1/[\sigma^2(F_o^2) + 0.10000F_o^2]$. The final agreement factors were $R = 0.044$ and $wR = 0.105$ with a goodness-of-fit of 1.300.

3.3. Pharmacological studies

3.3.1. Potentiation of 5-HTP-induced neurotoxicity in mice.[31] The 5-HT reuptake inhibition activity was determined in vivo by potentiation of 5-HTP induced neurotoxicity in mice.[31] The animals were divided into ten groups each of six male mice (18–22 g) to test compounds **14a–g** and fluoxetine, citalopram, and clomipramine as standard drugs (Table 1). They were treated with the test drug ip in the dose of 6.47 mM/kg as a suspension in saline and Tween 80. Thirty minutes later, the mice received 75 mg/kg pargyline HCl subcutaneously (sc). Ninety minutes after pargyline, the animals were injected with 10 mg/kg DL-5-HTP ip. An additional group of six animals was used as a control group which was injected with water–Tween 80 as a suspending agent. An animal was considered to give positive response if it showed head twitches 20 min after 5-HTP injection. According to the observed response%, the experiment was repeated similarly using either higher or lower doses. Enhancement was observed after treatment with serotonin uptake blockers relative to animals pretreated with pargyline only (control).

3.3.2. Potentiation of yohimbine-induced mortality in mice.[31] The NE reuptake inhibition activity was determined in vivo by potentiation of yohimbine-induced mortality in mice.[31] The animals were divided into ten groups each of six male mice (18–22 g) to test compounds **14a–g** and fluoxetine, citalopram and clomipramine as standard drugs (Table 2). They were treated with the test drug ip in the dose of 6.47 mM/kg as a suspension in saline and Tween 80. The animals received the test compound or water–Tween 80 as a suspending agent (control) by ip administration. Thirty min later, a dose of 25 mg/kg yohimbine (a sublethal dose) was given sc. The mortality rate was assessed 24 h after dosing. According to the observed response%, the experiment was repeated similarly using either higher or lower doses.

Using yohimbine-induced mortality, the effects of the treatment with fluoxetine, citalopram, clomipramine and new tropane derivatives on dose–response% were graphically illustrated in Figure 2 in comparison with their effect on 5-HTP-induced neurotoxicity.

Potency (ED_{50}) of these compounds were calculated by InStat 3 and were given in Table 3 and statistical comparisons between effect on 5-HTP treatment and yohimbine treatment were carried out by using Litchfield and Wilcoxon method (1949)[32] except for fluoxetine tested by Fischer's exact test.

References and notes

1. *The Global Burder of Disease*; Murray, C. J. L., Lopez, A. D., Eds.; Harvard University Press: Cambridge, MA, 1996.
2. Iversen, L.; Glennon, R. A. In *Burger's Medicinal Chemistry and Drug discovery*; 6th ed.; Antidepressants; Abraham, D. J., Ed.; John Wiley and Sons, Inc.: Canada, 2003; Vol. 6.
3. Stahl, S. M. *J. Clin. Psychiatry* **1998**, *59*(4), 5.
4. Perez, V.; Puigdemont, D.; Gilaberte, I.; Alvarez, E.; Artigas, F. *J. Clin. Psychopharmacol.* **2001**, *21*(1), 36.
5. Mongeau, R.; Blier, P.; de Montigny, C. *Brain Res. Rev.* **1997**, *23*, 145.
6. Einarson, T. R.; Addis, A.; Mittmann, N.; Iskedjian, M. *Can. J. Clin. Pharmacol.* **1998**, *5*, 205.
7. Rickels, K. *Biol. Psychiatry* **1991**, *1*, 345.
8. Hemrick-Luecke, S. K.; Fuller, R. W.; Snoddy, H. D. *J. Pharmacol. Exp. Ther.* **1994**, *269*, 132.
9. Tollefson, G. D.; Thompson, R. G.; Heiligenstein, J. H.; James, S.; Wong, D. T.; Faries, D. L. *Am. Coll. Neuropsychopharmacol.* **1993**.
10. Anttila, S.; Leinonen, E. *Curr. Opin. Investig. Drugs* **2002**, *3*, 1217.
11. Shaw, A. M.; Boules, M.; Zhang, Y.; Williams, K.; Robinson, J.; Carlier, P. R.; Richelson, E. *Eur. J. Pharmacol.* **2007**, *555*, 30.
12. Skolnick, P.; Popik, P.; Janowsky, A.; Beer, B.; Lippa, A. S. *Eur. J. Pharmacol.* **2003**, *461*, 99.
13. El Ahmed, Y.; Maillet, P.; Laurent, E.; Talab, A.; Teste, J. F.; Cedat, M. J. *Eur. J. Med. Chem.* **1997**, *32*, 205.
14. Helsley, G. C.; Davis, L.; Olsen; G. E. EP0333026, 1989.
15. Zhao, L.; Johnson, K. M.; Zhang, M.; Flippen-Anderson, J.; Kozikowski, A. P. *J. Med. Chem.* **2000**, *43*, 3283.
16. Blough, B. E.; Keverline, K. I.; Nie, Z.; Navarro, H.; Kuhar, M. J.; Carroll, F. I. *J. Med. Chem.* **2002**, *45*, 4029.
17. Hoepping, A.; Johnson, K. M.; George, C.; Flippen-Anderson, J.; Kozikowski, A. P. *J. Med. Chem.* **2000**, *43*(10), 2064.
18. Zhang, A.; Zhou, G.; Rong, S.-B.; Johnson, K. M.; Zhang, M.; Kozikowski, A. P. *Bioorg. Med. Chem. Lett.* **2002**, *12*, 993.
19. Zhou, J.; Kläß, T.; Zhang, A.; Johnson, K. M.; Wang, C. Z.; Ye, Y.; Kozikowski, A. P. *Bioorg. Med. Chem. Lett.* **2003**, *13*, 3565.
20. Zhou, J.; Kläß, T.; Johnson, K. M.; Gliberson, K. M.; Kozikowski, A. P. *Bioorg. Med. Chem. Lett.* **2005**, *15*, 2461.
21. Fuller, R. W.; Wong, D. T.; Robertson, D. W. *Med. Res. Rev.* **1991**, *11*(1), 17.
22. Cope, A. C.; D'Addieco, A. A. *J. Am. Chem. Soc.* **1951**, *73*, 3419.
23. Perregaard, J.; Mollzen, E. K.; Meier, E.; Sanchez, C. *J. Med. Chem.* **1995**, *38*(11), 1998.
24. March, J. Advanced organic chemistry, Reactions, Mechanisms and Structures. *Fourth ed*; John Wiley & Sons Inc: New York, 1992, p.641.
25. Spiniello, M.; White, J. M. *Org. Bimol. Chem.* **2003**, *1*, 3094.
26. Klaus, E.; Hans-Peter, K.; Heinz-Guenter, M.; Hartmann, S. US4333942, 1982.
27. Jain, M. P.; Thakur, R. S.; Rao, P. R. *Res. & Ind.* **1975**, *20*, 3.
28. Chou, T.-S.; Cheng, W.-J. *Bull. Inst. Chem. Acad. Sin.* **1982**, *29*, 9.
29. Altomare, A.; Cascarano, G.; Giacovazzo, C.; Guagliardi, A.; Burla, M. C.; Polidori, G.; Camalli, M. *J. Appl. Crystallogr.* **1994**, *27*, 435.

30. Mackay, S.; Gilmore, C. J.; Edwards, C.; Stewart, N.; Shankland, K. maXus Computer Program for the Solution and Refinement of Crystal Structures. Bruker Nonius, The Netherlands; MacScience, Japan & The University of Glasgow, 1999.
31. Vogel, H. G. *Drug discovery and evaluation, pharmacological assays; antidepressant activity*, second ed.; Springer-Verlag: Berlin Heidelberg, 2002, p 570, 571.
32. Litchfield, J. T.; Wilcoxon, F. *J. Pharmacol. Exp. Therap.* **1949**, *96*, 99.
