# Supplementary material for: 5-Hydroxytryptophan (5-HTP): Natural Occurrence, Analysis, Biosynthesis, Biotechnology, Physiology and Toxicology
Source: Int J Mol Sci. 2020 Dec 26;22(1):181. doi: 10.3390/ijms22010181 (PMC7796270; doi:10.3390/ijms22010181)
Supplement: Supplementary file 1 [file ijms-22-00181-s001.zip › 5-HTP.Data/PDF/3568321547/fendo-10-00158.pdf]

frontiers in Endocrinology

REVIEW
published: 08 April 2019
doi: 10.3389/fendo.2019.00158

Check for updates

Tryptophan Metabolic Pathways and Brain Serotonergic Activity: A Comparative Review

*Erik Höglund[1,2], Øyvind Øverli[3] and Svante Winberg[4]**

[1] Norwegian Institute of Water Research, Oslo, Norway, [2] Centre of Coastal Research, University of Agder, Kristiansand, Norway, [3] Department of Food Safety and Infection Biology, Faculty of Veterinary Medicine, Norwegian University of Life Sciences, Oslo, Norway, [4] Behavioural Neuroendocrinology Group, Department of Neuroscience, Uppsala University, Uppsala, Sweden

The essential amino acid L-tryptophan (Trp) is the precursor of the monoaminergic neurotransmitter serotonin (5-hydroxytryptamine, 5-HT). Numerous studies have shown that elevated dietary Trp has a suppressive effect on aggressive behavior and post-stress plasma cortisol concentrations in vertebrates, including teleosts. These effects are believed to be mediated by the brain serotonergic system, even though all mechanisms involved are not well understood. The rate of 5-HT biosynthesis is limited by Trp availability, but only in neurons of the hindbrain raphe area predominantly expressing the isoform TPH2 of the enzyme tryptophan hydroxylase (TPH). In the periphery as well as in brain areas expressing TPH1, 5-HT synthesis is probably not restricted by Trp availability. Moreover, there are factors affecting Trp influx to the brain. Among those are acute stress, which, in contrast to long-term stress, may result in an increase in brain Trp availability. The mechanisms behind this stress induced increase in brain Trp concentration are not fully understood but sympathetic activation is likely to play an important role. Studies in mammals show that only a minor fraction of Trp is utilized for 5-HT synthesis whereas a larger fraction of the Trp pool enters the kynurenic pathway. The first stage of this pathway is catalyzed by the hepatic enzyme tryptophan 2,3-dioxygenase (TDO) and the extrahepatic enzyme indoleamine 2,3-dioxygenase (IDO), enzymes that are induced by glucocorticoids and pro-inflammatory cytokines, respectively. Thus, chronic stress and infections can shunt available Trp toward the kynurenic pathway and thereby lower 5-HT synthesis. In accordance with this, dietary fatty acids affecting the pro-inflammatory cytokines has been suggested to affect metabolic fate of Trp. While TDO seems to be conserved by evolution in the vertebrate linage, earlier studies suggested that IDO was only present mammals. However, recent phylogenic studies show that IDO paralogues are present within the whole vertebrate linage, however, their involvement in the immune and stress reaction in teleost fishes remains to be investigated. In this review we summarize the results from previous studies on the effects of dietary Trp supplementation on behavior and neuroendocrinology, focusing on possible mechanisms involved in mediating these effects.

Keywords: serotonin, stress, aggression, immune response, fatty acids, dietary supplementation

OPEN ACCESS

Edited by:
Lluis Tort,
Autonomous University of Barcelona, Spain

Reviewed by:
Syaghalirwa N. M. Mandiki,
University of Namur, Belgium
Benjamin Costas,
Centro Interdisciplinar de Investigação Marinha e Ambiental (CIIMAR), Portugal

****Correspondence:***
Svante Winberg
svante.winberg@neuro.uu.se

Specialty section:
This article was submitted to Experimental Endocrinology, a section of the journal Frontiers in Endocrinology

Received: *06 November 2018*
Accepted: *22 February 2019*
Published: *08 April 2019*

Citation:
Höglund E, Øverli Ø and Winberg S (2019) Tryptophan Metabolic Pathways and Brain Serotonergic Activity: A Comparative Review. Front. Endocrinol. 10:158. doi: 10.3389/fendo.2019.00158

INTRODUCTION

Tryptophan (Trp) is an essential amino acid in all animals, which is synthesized and provided to higher trophic levels by bacteria, fungi and plants. In addition to being a component for protein synthesis, Trp is also the obligatory substrate for the production of several important bioactive substances. For example, tryptophan is a substrate for the synthesis of serotonin (5-hydroxytryptpamine, 5-HT) in the brain and gut, and melatonin in the pineal gland. In vertebrates, central 5-HT plays an integrative role in the behavioral and neuroendocrine stress response (1–3). Accordingly, effects of dietary Trp on the neuroendocrine stress response have been reported in a variety of species, spanning from teleosts to humans (4–10). However, the mechanisms underlying this link between Trp metabolism and the stress response are not fully understood.

In mammals, the majority of Trp is catabolized and transformed through the kynurenic pathway to bioactive substances which potentially can interact with the stress response (11). Moreover, infections, stress, and changes in the gut microbiome have all been shown to shunt Trp metabolism from 5-HT production toward this pathway (12, 13). Consequently, pathological changes in stress responsiveness, as in depression, have been related to nutritional factors, stress and immune function in humans (14, 15). However, in non-mammals, information on the kynurenic pathway and its interactions with central 5-HT signaling and the stress response is scattered and/or limited.

Dietary manipulations affecting Trp availability to the brain have been used as a tool to investigate involvement of the 5-HT system in behavior, mood and cognition in humans (16–18). Likewise, the dietary Trp content have been shown to affect endocrine and behavioral responses to stress in teleost fishes (10, 19, 20). This review summarizes the results from previous studies on the effects of dietary Trp supplementation on the behavioral and neuroendocrine stress response, focusing on possible mechanisms involved in mediating these effects. We also present a hypothesis on how the diet could be used to improve fish stress tolerance through interactions with the Trp metabolic pathways.

L-TRYPTOPHAN AVAILABILITY AND BRAIN SEROTONERGIC ACTIVITY

In serotonergic neurons Trp serves as the precursor for 5-HT. The 5-HT metabolic pathway is initiated by Trp being hydroxylated to the intermediate 5-hydroxytryptophan (5-HTP), which is subsequently decarboxylated to become 5-HT. Tissue levels of 5-HTP are usually low since this substance is rapidly decaroxylated by the enzyme aromatic amino acid decarboxylase [for review see (21)]. Thus, the rate limiting step in the biosynthesis of 5-HT is the hydroxylation of Trp which is catalyzed by the enzyme tryptophan hydroxylase (TPH) (**Figure 1**). This enzyme is specific for 5-HT producing cells, however, it is present in two different isoforms, TPH1 and TPH2 [reviewed in (22, 23)].

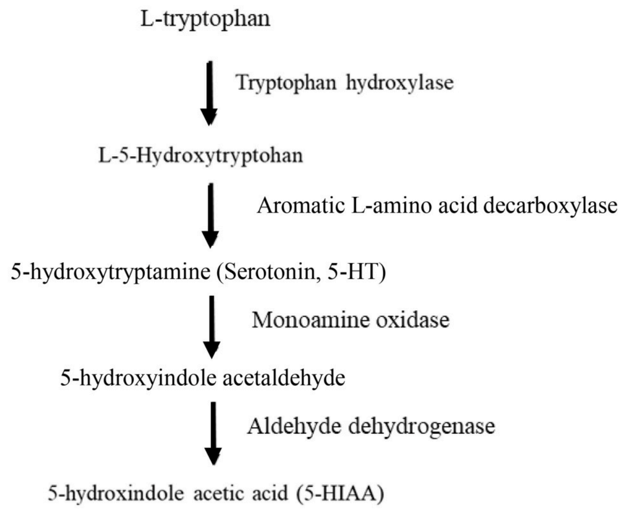

FIGURE 1 | Biosynthetic pathway of serotonin.

In amniotes 5-HT neurons are only present in the raphe area of the hind brain whereas in anamniotes, including teleosts, 5-HT cell bodies are also located in pretectal areas and basal forebrain. In zebrafish (*Danio rerio*) raphe and pretectal 5-HT cells express TPH2, whereas diencephalic and hypothalamic 5-HT cells express TPH1 (TPH1a and TPH1b) and TPH3, respectively (23). Interestingly, TPH2 show a Km for its substrate which is in the range of *in vivo* brain levels of Trp (24). Consequently, the rate of 5-HT synthesis in cells expressing TPH2 is drastically affected by changes in Trp availability, an effect which is probably not seen in 5-HTergic cells expressing other TPH isoforms (22). Moreover, the rate of 5-HT synthesis is believed to be reflected in the release of 5-HT, often quantified as the concentration of the catabolite 5-hydroxyindole acetic acid (5-HIAA), or the 5-HIAA/5-HT ratio. Thus, changes in Trp availability may have direct effects on 5-HTergic tone. Coherent to this, Russo et al. (25) made the interesting suggestion that Trp may act as signal to the brain, transferring information on peripheral homeostasic challenges to the 5-HT system which in turn could act to defend homeostasis. Dietary composition as well as stress, physical activity and immune system activation will all have effects on plasma Trp concentrations, and thus on brain Trp availability and raphe 5-HTergic activity (25). Such Trp related changes in 5-HTergic activity could have direct effects on behavior as well as endocrine status through 5-HT projections to telencephalic and hypothalamic areas. It could be argued that such effects may be less important in teleost fish since they have extra-raphe located 5-HT cell populations expressing the TPH1 isoform, making them less responsive to changes in Trp availability. However, in teleosts, as well as in other vertebrates, the raphe 5-HTergic cells have a wide projection pattern innervating most brain regions (23). Still, it has to be acknowledged that very little is known about the role of telesost forebrain 5-HT cell population in the control of behavior and endocrine functions (23).

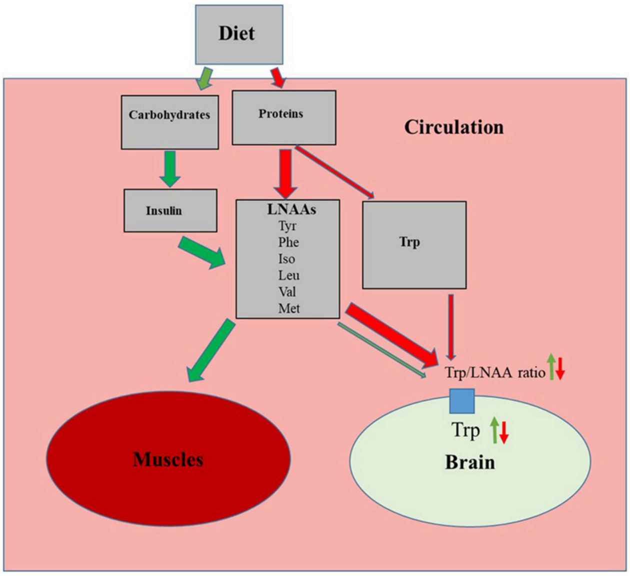

FIGURE 2 | Effects of the proteins and carbohydrates on influx of tryptophan (Trp) to the brain. Green arrows indicate activation of carbohydrate induced pathway, resulting increased muscle uptake of large neutral amino acids (LNAAs; Tyr, tyrosine; Phe, phenylalanine; Iso, isoleucine; Leu, leucine; Val, valine and Met, methionine) which in turn increases plasma Trp/LNAA ratio and brain Trp levels. Red arrows indicate how a normal dietary protein source, with relatively low Trp content, decreases the plasma Trp/LNAA ratio and brain Trp levels.

FACTORS AFFECTING TRP UPTAKE TO THE BRAIN

Dietary Effects on Trp Availability

The essential amino acid Trp enters the brain in competition with other large neutral amino acids (LNNAs; i.e., valine, isoleucine, leucine, tyrosine, phenylalanine and methionine) through a common transporter protein. Thus, the amount of Trp entering the brain depends on the plasma concentrations of Trp in relation to the other LNAAs [for references see reviews (26, 27)]. Hence, ingestion of a normal protein source, usually containing 0.5–1% Trp, results in a relatively small increase in Trp but a larger elevation of plasma concentrations of other LNNAs (28). This results in a decrease in the plasma Trp/LNAA ratio and thus reduced Trp influx to the brain (**Figure 2**). Dietary carbohydrates, on the contrary, increase brain Trp levels. This is due to elevated insulin which in turn promote uptake of LNAAs except Trp to the skeletal muscles, thereby increasing plasma Trp/LNAA ratio and Trp influx to the brain (**Figure 2**) (26, 27). This differential amino acid uptake to skeletal muscles is caused by the fact that Trp in blood plasma is bound to albumin whereas other LNAA are not. Trp influx to the brain is then promoted by the common LNAA transporter protein in the blood brain barrier having a much higher affinity for Trp compared to albumin (27).

Studies in rainbow trout (*Oncorhynchus myliss*) show that the amino acid composition of trout albumin differs from that of mammalians and lacks the binding site for indoles (29, 30). Thus, in rainbow trout, the majority of plasma Trp is in its free non-protein bound state (31, 32). This assumption is further strengthened by a study by Ruibal et al. (33) showing that hyperglycemia induced elevation of plasma insulin levels did not affect brain 5-HT activity in rainbow trout. It is not known if the lack of Trp binding by albumin is specific for rainbow trout or if it represents a more general trait of teleost albumin. However, it is possible that in teleost fishes brain influx of Trp could be more dependent of the dietary amino acid composition than on carbohydrates.

The Kynurenic Pathway

In fact, only a minor fraction of the Trp pool is utilized for 5-HT biosynthesis. In mammals, the majority of Trp enters the kynurenic pathway and is converted to other bioactive substances than 5-HT, such as kynurenic acid and quinolinic acid (**Figure 3**) [for references see review (11)]. The first stage of this pathway is catalyzed by the hepatic enzyme tryptophan 2,3-dioxygenase (TDO) and the extrahepatic enzyme indoleamine 2,3-dioxygenase (IDO), enzymes that are induced by glucocorticoids and pro-inflammatory cytokines, respectively (34). Thus, chronic stress and infections can shunt available Trp toward the kynurenic pathway and thereby lowering brain 5-HT synthesis while simultaneously increasing the production of other Trp based bioactive substances. Moreover, since a majority of Trp follows the kynurenic pathway (<95%, **Figure 3**) relative small changes in the activity of this pathway can have rather big impact on the Trp influx to the brain (35). Accordingly, decreased Trp influx to the brain as a result of stress or inflammation/infection induced activation of the kynurenic pathway have been suggested to be an underlying factor for mental illnesses and dysregulation of the neuroendocrine stress axis (12, 14, 15).

Generally, IDO is more nonspecific than TDO, and catabolizes other indoleamines than Trp. Moreover, two distinct IDO genes, IDO1 and IDO2, have been identified in vertebrates. Earlier studies suggested that IDO1 arose by a gene duplication in mammals (36). However, recent phylogenetic analyses show that IDO1 are present in reptiles and in teleosts, indicating that the gene duplication occurred in the common ancestor of vertebrates (37). In mammals, the activation of dendritic cells results in IDO1 induction with the depletion of Trp levels locally or systemically, a mechanism by which interferons inhibit the growth of certain bacteria, intracellular parasites, and viruses (34). Moreover, an elevation of the activity of the kynurenic pathway also inhibits T lymphocyte replication which results in immunosuppression and tolerogenicity. In line with this, IDO1 have been suggested to play an important role in preventing fetal rejection and in facilitating immune escape of tumor cells (34). In addition, some products of the kynrunic pathway may act anti-inflammatory (38, 39). However, to which extent these anti-inflammatory Trp catabolites acts back on the activity kynurenic pathway and thereby affecting Trp influx to the brain and/or central 5-HT signaling is to our knowledge unknown.

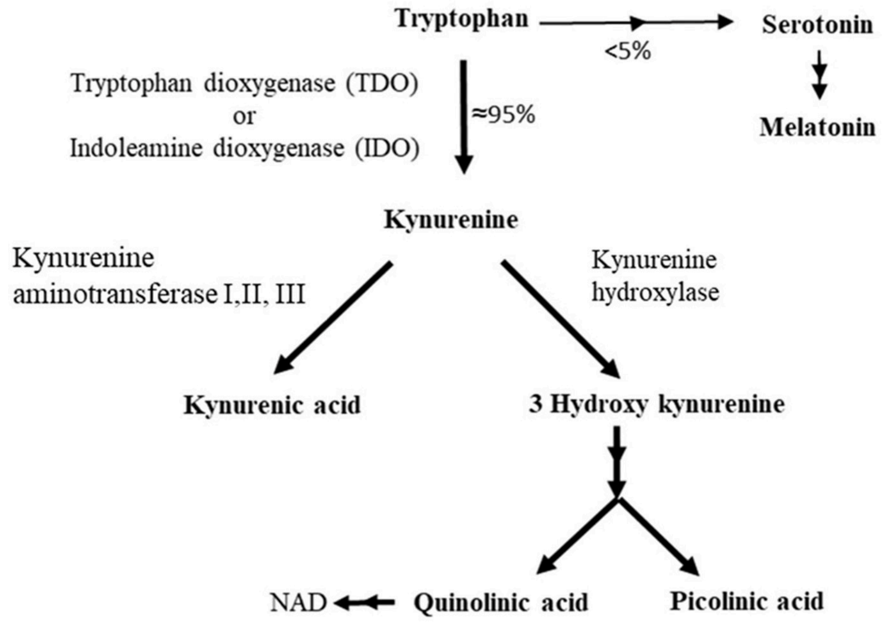

FIGURE 3 | Major metabolic pathway of tryptophan in mammals.

The Trp catabolizing efficiency of IDO2 and non-mammalian IDO1 seems to be lower than mammalian IDO1, and their function and involvement in the immune response in comparative model species is far less understood (37). However, recently, it has been demonstrated that treatment with bacterial lipopolysaccharide (LPS) induces and upregulation of IDO expression in rainbow trout, suggesting that this enzyme is involved in the immune response in non-mammalian vertebrates (40). Moreover, in the aforementioned study, expression of IDO was induced by the pro-inflammatory cytokine interferon gamma (IFNγ) in an *in vitro* cell model, indicating similar induction mechanisms as those in mammalian IDO1 (40). This suggests that systemic infection may decrease Trp influx to the brain of teleost fishes in the same way as in mammals, and result in behavioral and physiological changes (see section Kynurenine pathway).

Acute Stress

As discussed above chronic stress may result in lowered brain Trp availability as a consequence of a stress-induced activation of the kynurenine pathway. However, acute stress has been reported to have the opposite effect elevating brain Trp levels in both mammals (41, 42) and teleost fish (3, 10). This stress-induced increase in brain Trp concentrations appears at least in part related to a sympathetic activation and elevated levels of circulating plasma catecholamines (43). Plasma catecholamines stimulate lipolysis, resulting in elevated plasma levels of non-esterified fatty acids, which in turn could compete with Trp for binding to albumin and thus elevate the plasma pool of free Trp available for uptake into the brain [reviewed by (44)]. However, as discussed above, rainbow trout albumin appears to lack the Trp binding site, suggesting that mechanisms based on competition between Trp and non-esterified fatty acids are not involved in stress-induced increase in brain Trp in teleosts, at least not in rainbow trout. It has also been suggested that sympathetic activation results in increased permeability of the blood-brain barrier, another mechanism that could increase brain Trp influx (44).

TRP AND THE NEUROENDOCRINE STRESS RESPONSE

Stress Responses Are Modified by Trp Availability and Brain 5-HT Functions

As mentioned earlier in this review, the positive relationship between Trp availability and brain 5-HT production is well conserved within the vertebrate linage. Coherent to this, the involvement of 5-HT in the neuroendocrine regulation of the stress response seems to be similar within this linage. 5-HT plays a central role in control of the hypothalamus–pituitary–adrenal axis (HPA axis) in mammals, and the hypothalamic–pituitary–interrenal axis (HPI axis) in fish. This, mainly through its effects on the release of corticotropin-releasing factor (CRF) from the hypothalamus (45, 46). In addition, extra hypothalamic 5-HT appears be involved in appraisal and stress coping mechanisms, modulating behavioral and neuroendocrine responses to stressors (47, 48). Furthermore, as mentioned in section The Kynurenic pathway and Acute stress, stress by itself can influence the Trp influx to the brain, and thereby affect 5-HT signaling and the stress response. Moreover, the HPA/HPI axis are under feedback control on serval levels, including central 5-HT signaling. Thus, the link between Trp and the 5-HT system

and how they control behavioral and neuroendocrine stress responses appears complex with 5-HT having context dependent effects (19, 22, 49).

Effects of Elevated Dietary Trp

Long-term effects of Trp dietary manipulations on the neuroendocrine stress response have been observed in both mammals and teleost fishes [for a review see (49)]. For instance, in pigs, elevated dietary Trp had stress suppressive effects, including elevated hypothalamic 5-HT and lowered post stress plasma cortisol levels, effects that peaked after 5 days of dietary Trp enrichment (50). Similarly, (51) showed that post-stress plasma cortisol levels returned to baseline earlier after social stress in pigs fed Trp enriched feed for 7 days. Interestingly, a similar time frame for the suppressive effects of dietary Trp supplementation on glucocorticoid release has also been demonstrated in fish (for references see **Table 1**). For instance, studies in rainbow trout show that suppression of the neuroendocrine stress response is present after 7, but not after 3 or 28 days of treatment with dietary Trp supplementation (52). Furthermore, in the earlier studies showing a suppressive effect of elevated dietary Trp on the neuroendocrine response to an acute stressor the effects were investigated during or directly following a period of dietary Trp supplementation (10, 52). However, in recent studies in sea water reared Atlantic salmon (*Salmo salar*), the suppressive effect on post-stress plasma cortisol seems to appear between 2 and 8 days after terminating the Trp supplementation. Moreover, in Atlantic salmon, this suppressive effect was still present at 21 days post Trp supplementation (7, 53). Basic et al. (53) suggested that such slow acting Trp-induced alterations of HPI-axis reactivity could be related to smoltification, a process where salmonid fish adapt to sea water. Moreover, these long-term alternations of HPI axis reactivity was not related to changes in hypothalamic 5-HT neurochemistry. Instead they coincided with changes in dopaminergic neurochemistry in this brain part, effects which may be related to elevated activity of the kynruneric pathway, as discussed in section The Kynurenic pathway. Similar results were shown in the study performed by Höglund et al. (7), where 5-HTergic activity in hypothalamus did not follow the long term Trp induced suppressive effect on post stress cortisol levels. The latter study also included telencephalon and 5-HT activity followed the same general pattern as cortisol in this brain part. Höglund et al. (7) suggested that such region specific differences could be related to 5-HT signaling in telencephalon being more dependent on projections from the hindbrain raphe, a nucleus where 5-HT neurons are highly sensitive to available Trp, see section L-tryptophan availability and brain serotonergic activity.

Generally, teleost fishes have a remarkable neurogenic and regenerative capacity throughout ontogeny, and it has been suggested that structural changes may underlie long-lasting effects on telencephalic neurochemistry induced by elevated dietary Trp in teleost fishes (7). This type of brain architectural changes is supported by mammalian studies, showing that the 5-HT system is involved in the organization and development of its own neural projection pattern (65). In addition, a positive relationship between dietary Trp content and neural proliferation markers, such as (exogenous) 5-bromo-2-deoxyuridine and brain derived neurotrophic factor (BDNF) has been demonstrated in rats (66), which lends further support for the suggestion that dietary Trp can induce structural changes in the brain.

There are studies in teleost fishes showing effect of longer Trp treatment periods than 7 days (**Table 1**). For example, Tejpal et al. (60) showed that a 60 days of dietary Trp supplementation decreased baseline plasma cortisol values as well as the cortisol response to 60 days of crowding stress. Moreover, longer Trp treatment periods have also been shown to act stimulatory on plasma cortisol responses. For example, an immune challenge by i.p. injection of inactivated *Photobacterium damselae* suspension resulted in elevated cortisol values in seabass fed Trp supplemented feed for 2 weeks as compared to fish given standard feed fish (67). Furthermore, there is a rather high variability in the effect of elevated dietary Trp on baseline cortisol values (**Table 1**). This variability could reflect interspecific differences in Trp metabolism and neuroendocrine mechanisms (49). Moreover, Höglund et al. (19) suggested that such variation could be related to differences in HPI axis activation due to divergent rearing environments. For example, in the studies performed by Lepage et al. (10, 52, 62), fish were kept socially isolated while in other studies they were group reared (4, 7, 53, 54). Considering the fact that the 5-HT system is affected by social interaction (3, 22, 68), this type of rearing differences may explain some of the variability in the response to elevated dietary Trp. Moreover, studies in humans and rats suggest that individual variation in 5-HT neurotransmission underlies differences in the response to dietary Trp manipulation (27). It has become increasingly clear that individual variation in HPA/I axis reactivity is as widespread phenomena in the vertebrate linage (69). Still, if such individual variation is related to sensibility to dietary manipulations of dietary Trp content in non-mammalian vertebrates remains to be investigated.

Kynurenine Pathway

As mentioned above, in the section about factors affecting Trp uptake to the brain. Trp influx to the brain and brain 5-HT signaling can be modulated by the activation of the kynurenic pathway. In addition, metabolites of this pathway may affect neuronal signaling involved in stress coping processes [reviwed by (14)]. The metabolite in the first step of this pathway, kynurenine, readily passes the blood brain barrier (70). In the brain it is further degraded to kynurenic acid or quinolinic acid. Further down this pathway quinolinic acid produces neurotoxic compounds such as NMDA receptor agonists and oxidative radicals (71) while kynurenic acid is neuroprotective by being an NMDA receptor antagonist [for references see (14)]. In mammals, the neuroprotective kynurenic acid is mainly produced in astrocytes, while neurotoxic compounds are produced in macrophages and microglia (34). It has been suggested that an imbalance between these neurodegenerative and neuroprotective factors are involved in brain dysfunctions, including poor stress coping ability, in depression (72). In

TABLE 1 | Effects of dietary tryptophan supplementation on the behavioral and endocrine stress response in teleost fishes.

Species	Dose (x std feed)	Treatment (days)	Behavior	Plasma cortisol		Stressor		References
				Baseline	Stress	Type	Post Trp terat. (days)	
Oncorhuncus mykiss	2	7	N. i.	–	–	Confinement 2 h	1	(10)
	4	7	N. i.	↑	↓	Confinement 2 h	1	
	8	7	N. i.	↑	↓	Confinement 2 h	1	
Oncorhuncus mykiss	8	3	N. i.	↑	-	Confinement 2 h	1	(52)
	8	7	N. i.	–	↓	Confinement 2 h	1	
	8	28	N. i.	–	–	Confinement 2 h	1	
Gadus morhua	2	7	N. i.	–	–	Confinement (0.5 h)	1	(4)
	2	7	N. i.	–	–	Confinement (0.5 h)	2	
	2	7	N. i.	–	–	Confinement (0.5 h)	4	
	3	7	N. i.	–	–	Confinement (0.5 h)	1	
	3	7	N. i.	–	–	Confinement (0.5 h)	2	
	3	7	N. i.	–	–	Confinement (0.5 h)	4	
	4	7	N. i.	–	↓	Confinement (0.5 h)	1	
	4	7	N. i.	–	–	Confinement (0.5 h)	2	
	4	7	N. i.	–	–	Confinement (0.5 h)	4	
Salmo salar	2	7	N. i.	–	–	Confinement (0.5 h)	1	(53)
	2	7	N. i.	–	–	Confinement (0.5 h)	2	
	2	7	N. i.	↓	↓	Confinement (0.5 h)	10	
	3	7	N. i.	↓	–	Confinement (0.5 h)	1	
	3	7	N. i.	–	–	Confinement (0.5 h)	2	
	3	7	N. i.	↓	↓	Confinement (0.5 h)	10	
	4	7	N. i.	–	–	Confinement (0.5 h)	1	
	4	7	N. i.	–	↑	Confinement (0.5 h)	2	
	4	7	N. i.	↓	↓	Confinement (0.5 h)	10	
Oreochromis niloticus	4	7	N. i.	–	–	Chasing (0.3 h)	0	(54)
	10	7	N. i.	↓	–	Chasing (0.3 h)	0	
Salmo salar	2	7	N. i.	–	–	Crowding (1 h)	8	(7)
	2	7	N. i.	–	↓	Crowding (1 h)	21	
	3	7	N. i.	–	–	Crowding (1 h)	8	
	3	7	N. i.	–	↓	Crowding (1 h)	21	
Dicentrarchus labrax	2	14	N.i	–	↑	24 h post immune challenge	0	
Cyprinus carpio	5	15	N. i.	N. i.	↓	Saltwater (6 h)	0	(55)
Cyprinus carpio		21	N. i.	↓	↓	Cu+ exsposure (7 days)	0	(56)
Cichlasoma dimerus	8	28	N. i.	↓	N. i.			(57)
Labeo rohita	1.7[a]	45	N. i.	↓	N. i.			(58)

(Continued)

TABLE 1 | Continued

Species	Dose (x std feed)	Treatment (days)	Behavior	Plasma cortisol		Stressor		References
				Baseline	Stress	Type	Post Trp terat. (days)	
	2.4[a]	45	N. i.	↓	N. i.			
	2.9[a]	45	N. i.	↓	N. i.			
Labeo rohita	2.8	60	N. i.	N. i.	↓	Temp and/or salt (30 days)	0	(59)
	4.8			N. i.	↓	Temp and/or salt (30 days)	0	
Cirrhinus mrigala	~3[a]	60	N. i.	↓	↓	High rearing density (60 days)	0	(60)
Sander lucioperca	3	7-60	N. i.	N. i.	↓	Emersion	0	(61)
	6	7-60	N. i.	N. i.	↓	Emersion	0	
**AGGRESSION**								
Oncorhyncus mykiss	36	0	–				0	(20)
		3	–				0	
		7	↓				0	
	360	0	–				0	
		3	–				0	
		7	↓				0	
Oncorhuncus mykiss	8	7	↓	N. i	↓	A smaller conspefic (1 h)	1	(62)
Gadus morhua	6[a]	4–10	↓	N. i	N. i	3 × social interact. (0.15 h/day)	0	(63)
Matrinxã Brycon amaz.	2	7	↓	N. i.	↓	Social interaction (0.3 h)	0	(64)
	4	7	↓<br>Anneroxia	N. i.	↓	Social interaction (0.3 h)	0	
Salmo trutta	3.6	7	↓	N. i.	N. i.	Novel environment (3 days)	1–3	(19)

↑, ↓, and – refers to stimulating, suppressive or no effect t compared to standard feed.
N.i. Not investigated.
[a]*Estimated from similar feed recipe.*

addition, studies in rats show that dietary Trp can affect brain levels of kynurenic acid (73), which in turn effects other neurotransmitters, such as dopamine and glutamine through activation of NMDA and/or a7 nicotinic acetylcholine receptor (74, 75). Central effects of Trp metabolites produced by the kynurenic pathway in teleost fishes are, to our knowledge, largely unknown. Still, effects of dietary Trp supplementation on dopaminergic neurochemistry in Atlanitic salmon (53) and Atlanitic cod (*Gadus morhua*) (4) have been suggested to be related to elevated levels of kynurenic acid (53).

BEHAVIORAL EFFECTS OF ELEVATED DIETARY TRP

There is a general consensus that low levels of central 5-HT are associated with high levels of aggression within the vertebrate subphylum (3, 69). In line with this, human studies show that alterations of the dietary Trp content changes irritability and aggressive behavior [for references see review by Young and Leyton (76)]. For example, human lab studies show that dietary Trp induces a dose dependent effect on aggressive responses, where Trp supplementation and depletion induced the lowest highest aggression, respectively (77, 78). This negative relationship between dietary Trp content and aggression is further supported by studies on rats and birds, showing that Trp loading can attenuate aggressiveness (79, 80). Similarly, there are studies in teleost fishes showing a general suppressive effect on aggressive behavior by dietary Trp supplementation (20, 63, 64). Furthermore, in the study performed by Winberg et al. (20) the attenuating effects of dietary Trp on aggressive responses during territorial defense followed the same time-coarse as the effects on the neuroendocrine stress response in rainbow trout (52), with a peak after 7 days of treatment. This together with a study performed by Höglund et al. (19), showing that the same treatment time attenuated the anorexic response to a novel environment, strongly suggest that Trp affects 5-HT signaling and the integrating role of this neurotransmitter in behavioral and neuroendocrine stress responses.

Dietary Trp supplementation have also been shown to reduce cannibalism in juvenile grouper (*Epinephelus coioides*) (81) and pike perch (*Sander lucioperca*) (82). However, the behavioral components of this response were not studied. Differences in body size is a main factor underlying cannibalism in piscivorous fish (83), and one possible explanation to the reduced cannibalism could be a more homogeneous growth due to reduced competition for food in fish given Trp supplemented food. The behavioral effect of dietary Trp manipulations in teleost fishes are summarized in **Table 1**.

CONCLUSIONS AND SUGGESTION FOR DIRECTION OF FURTHER STUDIES

A positive relationship between dietary Trp and brain 5-HT activity seems to be present across the vertebrate linage. However, there appear to be differences between teleost fishes and mammals when it comes to plasma Trp transport since teleost albumin lacks the indole binding site (29, 30). This makes Trp influx to the brain less sensitive to carbohydrates in fish compared to mammals. On the other hand, behavioral and neuroendocrine effects of elevated dietary Trp are similar in all vertebrates. Studies in mammals and teleost fishes show that these effects, including suppression of aggressive behavior, attenuation of stress induced anorexia and lower post stress plasma cortisol, appear after 3–7 days of elevated dietary Trp intake. It has been suggested this slow time-course reflects 5-HT induced structural changes in the brain (7). However, further studies are needed to verify this assumption.

In mammals the majority of Trp enters the kynurenic pathway. The first stage of this pathway is catalyzed by the enzymes TDO and IDO that are induced by glucocorticoids and pro-inflammatory cytokines, respectively. Thus, chronic stress and infections can shunt available Trp toward the kynurenic pathway and thereby lowering the rate of brain 5-HT synthesis while simultaneously increasing the production of other Trp metabolites [for references see (14)], which potentially can affect behavioral and endocrine responses to stress. So far, the kynurenic pathway have been neglected when investigating effects of dietary Trp supplementation in teleost fishes. It has previously been pointed out that effects of dietary Trp is context dependent, where especially the stress status of the animals can affect the outcome of dietary Trp manipulation (19). A recent study demonstrates that the expression of IDO mRNA is upregulated by LPS in rainbow trout (40), suggesting that bacterial infection can affect the catabolic faith of Trp also in fish. Previously dietary Trp supplementation have been suggested as a strategy for reducing unavoidable stress, such as stress related to transport, size grading and vaccination, in aquaculture (84). However, considering that inflammatory processes might affect the catabolic faith of Trp in teleost fish, anti-inflammatory treatments should also be considered.

In humans, low circulating levels of the ω3 fatty acids, eicosapentaenoic acid (EPA) and docosahexaenoic acid (DHA), and a decreased ratio of EPA to the ω6 fatty acid arachidonic acid (ARA) have been associated with psychiatric ailments and poor stress coping ability (15). Moreover, a diet with high DHA and EPA have been shown to affect serotonergic transmission and to prevent such psychiatric ailments [for references see (15)]. The mechanisms for this anti-depressive action of ω3 fatty acids are currently not fully understood. However, it is possible that a diet with high ω3 content results in a suppression of pro-inflammatory eicosanoids, which in turn may reduce the activity of the kynurenic pathway, increasing Trp influx to the brain, and subsequently stimulate brain 5-HT synthesis.

The relative amount of marine ω3 fatty acids has decreased in commercial fish feed. Potentially, this may result in poorer stress coping ability trough dietary effects on central 5-HT signaling. Thus, we hypothesize that it is not only the relative amount of Trp to other LNAAs in the diet that is important for producing stress resilient robust fish. Rather, there is an interplay between dietary amino and fatty acids that decides the effects of Trp supplementation, where ratio ω3 to ω6 fatty acids in the diet influences the catabolic faith of Trp. Studies demonstrating a

negative relationship between HPI-axis reactivity and the ration of ω3 to ω6 fatty acids in the diet (85, 86) lends support to this hypothesis. However, if such effects of dietary fatty acid composition are related to changes in the activity of the kynurenic pathway is currently not known.

AUTHOR CONTRIBUTIONS

EH and SW drafted the manuscript. EH, ØØ, and SW finalized the manuscript.

FUNDING

We declare that all sources of funding received for this research have been acknowledged.

ACKNOWLEDGMENTS

This work was supported by the Swedish Research Council VR (621-2012-4679 to SW), the Norwegian Seafood Research Fund-FHF (901282 to EH and SW).

REFERENCES

1. Puglisi-Allegra S, Andolina D. Serotonin and stress coping. *Behav Brain Res.* (2015) 277:58–67. doi: 10.1016/j.bbr.2014.07.052
2. Sandi C, Haller J. Stress and the social brain: behavioural effects and neurobiological mechanisms. *Nat Rev Neurosci.* (2015) 6:290. doi: 10.1038/nrn3918
3. Winberg S, Nilsson GE. Roles of brain monoamine neurotransmitters in agonistic behaviour and stress reactions, with particular reference to fish. *Comp Biochem Physiol C.* (1993) 106:597–614. doi: 10.1016/0742-8413(93)90216-8
4. Basic D, Schjolden J, Krogdahl Å, von Krogh K, Hillestad M, Winberg S, et al. Changes in regional brain monoaminergic activity and temporary down-regulation in stress response from dietary supplementation with L-tryptophan in Atlantic cod (Gadus morhua). *Brit J Nutr.* (2013b) 109:2166–74. doi: 10.1017/S0007114512004345
5. Carrillo M, Ricci LA, Coppersmith GA, Melloni RH. The effect of increased serotonergic neurotransmission on aggression: a critical meta-analytical review of preclinical studies. *Psychopharmacol.* (2009) 205:349–68. doi: 10.1007/s00213-009-1543-2
6. Firk C, Markus CR. Mood and cortisol responses following tryptophan-rich hydrolyzed protein and acute stress in healthy subjects with high and low cognitive reactivity to depression. *Clin Nutr.* (2009) 28:266–71. doi: 10.1016/j.clnu.2009.03.002
7. Höglund E, Øverli Ø, Andersson MÅ, Silva P, Laursen DC, Moltesen MM, et al. Dietary l-tryptophan leaves a lasting impression on the brain and the stress response. *Brit J Nutr.* (2017) 117:1351–7. doi: 10.1017/S0007114517001428
8. Koopmans S, Guzik A, Van Der Meulen J, Dekker R, Kogut J, Kerr B, et al. Effects of supplemental L-tryptophan on serotonin, cortisol, intestinal integrity, and behavior in weanling piglets. *J Anim Sci.* (2006) 84:963–71. doi: 10.2527/2006.844963x
9. Le Floc'h N, Seve B. Biological roles of tryptophan and its metabolism: Potential implications for pig feeding. *Livest Sci.* (2007) 112:23–32. doi: 10.1016/j.livsci.2007.07.002
10. Lepage O, Tottmar O, Winberg S. Elevated dietary intake of L-tryptophan counteracts the stress-induced elevation of plasma cortisol in rainbow trout (Oncorhynchus mykiss). *J Exp Biol.* (2002) 205:3679–87.
11. Le Floc'h N, Otten W, Merlot E. Tryptophan metabolism, from nutrition to potential therapeutic applications. *Amino Acids.* (2011) 41:1195–205. doi: 10.1007/s00726-010-0752-7
12. O'Farrell K, Harkin A. Stress-related regulation of the kynurenine pathway: relevance to neuropsychiatric and degenerative disorders. *Neuropharmacol.* (2017) 112:307–23. doi: 10.1016/j.neuropharm.2015.12.004
13. O'mahony SM, Clarke G, Borre YE, Dinan TG, Cryan JF. Serotonin, tryptophan metabolism and the brain-gut-microbiome axis. *Behav Brain Res.* (2015) 277:32–48. doi: 10.1016/j.bbr.2014.07.027
14. Dantzer R, O'Connor JC, Freund GG, Johnson RW, Kelley KW. From inflammation to sickness and depression: when the immune system subjugates the brain. *Nat Rev Neurosci.* (2008) 9:46. doi: 10.1038/nrn2297
15. Maes M, Yirmyia R, Noraberg J, Brene S, Hibbeln J, Perini G, et al. The inflammatory & neurodegenerative (I&ND) hypothesis of depression: leads for future research and new drug developments in depression. *Metab Brain Dis.* (2009) 24:27–53. doi: 10.1007/s11011-008-9118-1
16. Markus C, Panhuysen G, Jonkman L, Bachman M. Carbohydrate intake improves cognitive performance of stress-prone individuals under controllable laboratory stress. *Brit J Nutr.* (1999) 82:457–67.
17. Markus R, Panhuysen G, Tuiten A, Koppeschaar H. Effects of food on cortisol and mood in vulnerable subjects under controllable and uncontrollable stress. *Physiol Behav.* (2000) 70:333–42. doi: 10.1016/S0031-9384(00)00265-1
18. Reilly J, McTavish S, Young A. Rapid depletion of plasma tryptophan: a review of studies and experimental methodology. *J Psychopharmacol.* (1997) 11:381–92. doi: 10.1177/026988119701100416
19. Höglund E, Sørensen C, Bakke MJ, Nilsson GE, Øverli Ø. Attenuation of stress-induced anorexia in brown trout (Salmo trutta) by pre-treatment with dietary L-tryptophan. *Brit J Nutr.* (2007) 97:786–9. doi: 10.1017/S0007114507450280
20. Winberg S, Øverli Ø, Lepage O. Suppression of aggression in rainbow trout (Oncorhynchus mykiss) by dietary L-tryptophan. *J Exp Biol.* (2001) 204:3867–76.
21. Boadle-Biber MC. Regulation of serotonin synthesis. *Prog Biophys Mol Biol.* (1993) 60:1–15. doi: 10.1016/0079-6107(93)90009-9
22. Backström T, Winberg S. Serotonin coordinates responses to social stress—What we can learn from fish. *Fronts Neurosci.* (2017) 11:595. doi: 10.3389/fnins.2017.00595
23. Lillesaar C. The serotonergic system in fish. *J Chem Neuroanat.* (2011) 41:294–308. doi: 10.1016/j.jchemneu.2011.05.009
24. McKinney J, Knappskog PM, Haavik J. Different properties of the central and peripheral forms of human tryptophan hydroxylase. *J Neurochem.* (2005) 92:311–20. doi: 10.1111/j.1471-4159.2004.02850.x
25. Russo S, Kema IP, Bosker F, Haavik J, Korf J. Tryptophan as an evolutionarily conserved signal to brain serotonin: molecular evidence and psychiatric implications. *World J Biol Psychiat.* (2009) 10:258–68. doi: 10.3109/15622970701513764
26. Fernstrom JD. Role of precursor availability in control of monoamine biosynthesis in brain. *Physiol Rev.* (1983) 63:484–546. doi: 10.1152/physrev.1983.63.2.484
27. Markus CR. Dietary amino acids and brain serotonin function; implications for stress-related affective changes. *Neuromol Med.* (2008) 10:247. doi: 10.1007/s12017-008-8039-9
28. Fernstrom JD. Aromatic amino acids and monoamine synthesis in the central nervous system: influence of the diet. *J Nutr Biochem.* (1990) 1:508–17. doi: 10.1016/0955-2863(90)90033-H
29. Fuller RW, Roush BW. Binding of tryptophan to plasma proteins in several species. *Comp Biochem Physiology B.* (1973) 46:273–6. doi: 10.1016/0305-0491(73)90318-0
30. McLachlan A, Walker JE. Evolution of serum albumin. *J Mol Biol.* (1977) 112:543–58. doi: 10.1016/S0022-2836(77)80163-0
31. Rozas G, Rey P, Andrés M, Rebolledo E, Aldegunde M. Distribution of 5-hydroxytryptamine and related compounds in various brain regions of rainbow trout (Oncorhynchus mykiss). *Fish Physiol Biochem.* (1990) 8:501–6. doi: 10.1007/BF00003407
32. Walton M, Coloso RM, Cowey C, Adron J, Knox D. The effects of dietary tryptophan levels on growth and metabolism of rainbow trout (Salmo gairdneri). *Brit J Nutr.* (1984) 51:279–87. doi: 10.1079/BJN19840032

33. Ruibal C, Soengas J, Aldegunde M. Brain serotonin and the control of food intake in rainbow trout (Oncorhynchus mykiss): effects of changes in plasma glucose levels. *J Comp Physiol A.* (2002) 188:479–84. doi: 10.1007/s00359-002-0320-z
34. Takikawa O. Biochemical and medical aspects of the indoleamine 2, 3-dioxygenase-initiated L-tryptophan metabolism. *Biochem Biophys Res Com.* (2005) 338:12–9. doi: 10.1016/j.bbrc.2005.09.032
35. Salter M, Knowles RG, Pogson C. How does displacement of albumin-bound tryptophan cause sustained increases in the free tryptophan concentration in plasma and 5-hydroxytryptamine synthesis in brain? *Biochem J.* (1989) 262:365–8.
36. Yuasa HJ, Ball HJ, Ho YF, Austin CJ, Whittington CM, Belov K, et al. Characterization and evolution of vertebrate indoleamine 2, 3-dioxygenases: IDOs from monotremes and marsupials. *Comp Biochem Physiol B.* (2009) 153:137–44. doi: 10.1016/j.cbpb.2009.02.002
37. Yuasa HJ, Mizuno K, Ball HJ. Low efficiency IDO2 enzymes are conserved in lower vertebrates, whereas higher efficiency IDO1 enzymes are dispensable. *FEBS J.* (2015) 282:2735–45. doi: 10.1111/febs.13316
38. Li P, Yin YL, Li D, Kim SW, Wu G. Amino acids and immune function. *Brit J Nutr.* (2007) 98:237–52. doi: 10.1017/S000711450769936X
39. Munn DH, Mellor AL. Indoleamine 2,3 dioxygenase and metabolic control of immune responses. *Trends Immunol.* (2013) 34:137–43. doi: 10.1016/j.it.2012.10.001
40. Cortés J, Alvarez C, Santana P, Torres E, Mercado L. Indoleamine 2, 3-dioxygenase: first evidence of expression in rainbow trout (Oncorhynchus mykiss). *Dev Comp Immunol.* (2016) 65:73–8. doi: 10.1016/j.dci.2016.06.020
41. Curzon G, Joseph M, Knott PJ. Effects of immobilization and food deprivation on rat brain tryptophan metabolism. *J Neurochem.* (1972) 19:1967–74. doi: 10.1111/j.1471-4159.1972.tb01486.x
42. Dunn AJ. Changes in plasma and brain tryptophan and brain serotonin and 5-hydroxyindoleacetic acid after footshock stress. *Life Sci.* (1988) 42:1847–53. doi: 10.1016/0024-3205(88)90023-9
43. Dunn AJ, Welch J. Stress-and endotoxin-induced increases in brain tryptophan and serotonin metabolism depend on sympathetic nervous system activity. *J Neurochem.* (1991) 57:1615–22. doi: 10.1111/j.1471-4159.1991.tb06359.x
44. Chaouloff F. Physiopharmacological interactions between stress hormones and central serotonergic systems. *Brain Res Rev.* (1993) 18:1–32. doi: 10.1016/0165-0173(93)90005-K
45. Dinan TG. Serotonin and the regulation of hypothalamic-pituitary-adrenal axis function. *Life Sci.* (1996) 58:1683–94. doi: 10.1016/0024-3205(96)00066-5
46. Winberg S, Nilsson A, Hylland P, Söderstöm V, Nilsson GE. Serotonin as a regulator of hypothalamic-pituitary-interrenal activity in teleost fish. *Neurosci Lett.* (1997) 230:113–6. doi: 10.1016/S0304-3940(97)00488-6
47. De Kloet ER, Joëls M, Holsboer F. Stress and the brain: from adaptation to disease. *Nature Rev Neurosci.* (2005) 6:463. doi: 10.1038/nrn1683
48. Moltesen M, Laursen DC, Thörnqvist P-O, Andersson MÅ, Winberg S, Höglund E. Effects of acute and chronic stress on telencephalic neurochemistry and gene expression in rainbow trout (Oncorhynchus mykiss). *J Exp Biol.* (2016) 219:3907–14. doi: 10.1242/jeb.139857
49. Hoseini SM, Perez-Jimenez A, Costas B, Azeredo R, Gesto M. Physiological roles of tryptophan in teleosts: current knowledge and perspectives for future studies. *Rev Aquacult*. (2017) 11:3–24. doi: 10.1111/raq.12223
50. Adeola O, Ball R. Hypothalamic neurotransmitter concentrations and meat quality in stressed pigs offered excess dietary tryptophan and tyrosine. *J Anim Sci.* (1992) 70:1888–94. doi: 10.2527/1992.7061888x
51. Koopmans SJ, Ruis M, Dekker R, van Diepen H, Korte M, Mroz Z. Surplus dietary tryptophan reduces plasma cortisol and noradrenaline concentrations and enhances recovery after social stress in pigs. *Physiol Behav.* (2005) 85:469–78. doi: 10.1016/j.physbeh.2005.05.010
52. Lepage O, Vílchez IM, Pottinger TG, Winberg S. Time-course of the effect of dietary L-tryptophan on plasma cortisol levels in rainbow trout Oncorhynchus mykiss. *J Exp Biol.* (2003) 206:3589–99. doi: 10.1242/jeb.00614
53. Basic D, Krogdahl Å, Schjolden J, Winberg S, Vindas MA, Hillestad M, et al. Short-and long-term effects of dietary l-tryptophan supplementation on the neuroendocrine stress response in seawater-reared Atlantic salmon (Salmo salar). *Aquaculture.* (2013a) 388:8–13. doi: 10.1016/j.aquaculture.2013.01.014
54. Martins CI, Silva PI, Costas B, Larsen BK, Santos GA, Conceição LE, et al. The effect of tryptophan supplemented diets on brain serotonergic activity and plasma cortisol under undisturbed and stressed conditions in grouped-housed Nile tilapia Oreochromis niloticus. *Aquaculture.* (2013) 400:129–34. doi: 10.1016/j.aquaculture.2013.02.035
55. Hoseini SM, Hosseini SA. Effect of dietary L-tryptophan on osmotic stress tolerance in common carp, cyprinus carpio, juveniles. *Fish Physiol Biochem*. (2010) 36:1061–7. doi: 10.1007/s10695-010-9383-x
56. Hoseini SM, Hosseini SA, Soudagar M. Dietary tryptophan changes serum stress markers, enzyme activity, and ions concentration of wild common carp cyprinus carpio exposed to ambient copper. *Fish Physiol Biochem.* (2012) 38:1419–26. doi: 10.1007/s10695-012-9629-x
57. Morandini L, Ramallo MR, Moreira RG, Höcht C, Somoza GM, Silva A, et al. Serotonergic outcome, stress and sexual steroid hormones, and growth in a South American cichlid fish fed with an L-tryptophan enriched diet. *Gen Comp Endocrinol.* (2015) 223:27–37. doi: 10.1016/j.ygcen.2015.10.005
58. Kumar P, Saurabh S, Pal A, Sahu N, Arasu A. Stress mitigating and growth enhancing effect of dietary tryptophan in rohu (Labeo rohita, Hamilton, 1822) fingerlings. *Fish Physiol Biochem.* (2014) 40:1325–38. doi: 10.1007/s10695-014-9927-6
59. Akhtar M, Pal A, Sahu N, Ciji A, Meena D, Das P. Physiological responses of dietary tryptophan fed Labeo rohita to temperature and salinity stress. *J Anim Physiol Anim Nutr.* (2013) 97:1075–83. doi: 10.1111/jpn.12017
60. Tejpal C, Pal A, Sahu N, Kumar JA, Muthappa N, Vidya S, et al. Dietary supplementation of L-tryptophan mitigates crowding stress and augments the growth in Cirrhinus mrigala fingerlings. *Aquaculture.* (2009) 293:272–7. doi: 10.1016/j.aquaculture.2008.09.014
61. Mandiki RS, Redivo B, Baekelandt S, Douxfils J, Lund I, Höglund E, et al. Long-term tryptophan supplementation decreased the welfare and innate immune status of pikeperch juveniles. *Fish Shellfish Immunol.* (2016) 53:113–4. doi: 10.1016/j.fsi.2016.04.090
62. Lepage O, Larson ET, Mayer I, Winberg S. Serotonin, but not melatonin, plays a role in shaping dominant–subordinate relationships and aggression in rainbow trout. *Horm Behav.* (2005) 48:233–42. doi: 10.1016/j.yhbeh.2005.02.012
63. Höglund E, Bakke MJ, Øverli Ø, Winberg S, Nilsson GE. Suppression of aggressive behaviour in juvenile Atlantic cod (Gadus morhua) by l-tryptophan supplementation. *Aquaculture.* (2005) 249:525–31. doi: 10.1016/j.aquaculture.2005.04.028
64. Wolkers CPB, Serra M, Hoshiba MA, Urbinati EC. Dietary L-tryptophan alters aggression in juvenile matrinxa Brycon amazonicus. *Fish Physiol Biochem.* (2012) 38:819–27. doi: 10.1007/s10695-011-9569-x
65. Daubert EA, Condron BG. Serotonin: a regulator of neuronal morphology and circuitry. *Trend Neurosci.* (2010) 33:424–34. doi: 10.1016/j.tins.2010.05.005
66. Musumeci G, Castrogiovanni P, Castorina S, Imbesi R, Szychlinska MA, Scuderi S, et al. Changes in serotonin (5-HT) and brain-derived neurotrophic factor (BDFN) expression in frontal cortex and hippocampus of aged rat treated with high tryptophan diet. *Brain Res Bull.* (2015) 119:12–8. doi: 10.1016/j.brainresbull.2015.09.010
67. Azeredo R, Machado M, Afonso A, Fierro-Castro C, Reyes-López FE, Tort L, et al. Neuroendocrine and immune responses undertake different fates following tryptophan or methionine dietary treatment: Tales from a teleost model. *Fronts Immunol.* (2017) 8:1226. doi: 10.3389/fimmu.2017.01226
68. Summers CH, Winberg S. Interactions between the neural regulation of stress and aggression. *J Exp Biol.* (2006) 209:4581–9. doi: 10.1242/jeb.02565
69. Øverli Ø, Sørensen C, Pulman KG, Pottinger TG, Korzan W, Summers CH, et al. Evolutionary background for stress-coping styles: relationships between physiological, behavioral, and cognitive traits in non-mammalian vertebrates. *Neuroscie Biobehav Rev.* (2007) 31:396–412. doi: 10.1016/j.neubiorev.2006.10.006
70. Fukui S, Schwarcz R, Rapoport SI, Takada Y, Smith QR. Blood–brain barrier transport of kynurenines: implications for brain synthesis and metabolism. *J Neurochem.* (1991) 56:2007–17. doi: 10.1111/j.1471-4159.1991.tb03460.x
71. Stone TW, Forrest CM, Darlington LG. Kynurenines and brain development. In: Sandeep M, editor. *Targeting the Broadly Pathogenic Kynurenine Pathway*. Cham: Springer (2015). p. 45–61.

72. Miller AH, Maletic V, Raison CL. Inflammation and its discontents: the role of cytokines in the pathophysiology of major depression. *Biol Psychiat.* (2009) 65:732–41. doi: 10.1016/j.biopsych.2008.11.029
73. Okuno A, Fukuwatari T, Shibata K. High tryptophan diet reduces extracellular dopamine release via kynurenic acid production in rat striatum. *J Neurochem.* (2011) 118:796–805. doi: 10.1111/j.1471-4159.2011.07369.x
74. Carpenedo R, Pittaluga A, Cozzi A, Attucci S, Galli A, Raiteri M, et al. Presynaptic kynurenate-sensitive receptors inhibit glutamate release. *Eur J Neurosci.* (2001) 13:2141–7. doi: 10.1046/j.0953-816x.2001.01592.x
75. Rassoulpour A, Wu HQ, Ferre S, Schwarcz R. Nanomolar concentrations of kynurenic acid reduce extracellular dopamine levels in the striatum. *J Neurochem.* (2005) 93:762–5. doi: 10.1111/j.1471-4159.2005.03134.x
76. Young SN, Leyton M. The role of serotonin in human mood and social interaction: insight from altered tryptophan levels. *Pharmacol Biochem Behav.* (2002) 71:857–65. doi: 10.1016/S0091-3057(01)00670-0
77. Bjork JM, Dougherty DM, Moeller FG, Swann AC. Differential behavioral effects of plasma tryptophan depletion and loading in aggressive and nonaggressive men. *Neuropsychopharmacol.* (2000) 22:357. doi: 10.1016/S0893-133X(99)00136-0
78. Pihl RO, Young SN, Harden P, Plotnick S, Chamberlain B, Ervin FR. Acute effect of altered tryptophan levels and alcohol on aggression in normal human males. *Psychopharmacol.* (1995) 119:353–60. doi: 10.1007/BF02245849
79. Gibbons JL, Barr GA, Bridger WH, Liebowitz SF. Manipulations of dietary tryptophan: effects on mouse killing and brain serotonin in the rat. *Brain Res.* (1979) 169:139–53. doi: 10.1016/0006-8993(79)90380-9
80. van Hierden YM, Koolhaas JM, Korte SM. Chronic increase of dietary L-tryptophan decreases gentle feather pecking behaviour. *Appl Anim Behav Sci.* (2004) 89:71–84. doi: 10.1016/j.applanim.2004.05.004
81. Hseu J, Lu F, Su H, Wang L, Tsai C, Hwang P. Effect of exogenous tryptophan on cannibalism, survival and growth in juvenile grouper, Epinephelus coioides. *Aquaculture.* (2003) 218:251–63. doi: 10.1016/S0044-8486(02)00503-3
82. Król J, Zakeś Z. Effect of dietary l-tryptophan on cannibalism, survival and growth in pikeperch *Sander lucioperca* (L.) post-larvae. *Aquacult Int.* (2016) 24:441–51. doi: 10.1007/s10499-015-9936-1
83. Hecht T, Pienaar AG. A review of cannibalism and its implications in fish larviculture. *J World Aquacult Soc.* (1993) 24:246–61. doi: 10.1111/j.1749-7345.1993.tb00014.x
84. Conceiçao LE, Aragão C, Dias J, Costas B, Terova G, Martins C, et al. Dietary nitrogen and fish welfare. *Fish Physiol Biochem.* (2012) 38:119–41. doi: 10.1007/s10695-011-9592-y
85. Koven W, van Anholt R, Lutzky S, Atia IB, Nixon O, Ron B, et al. The effect of dietary arachidonic acid on growth, survival, and cortisol levels in different-age gilthead seabream larvae (Sparus auratus) exposed to handling or daily salinity change. *Aquaculture.* (2003) 228:307–20. doi: 10.1016/S0044-8486(03)00317-X
86. Montero D, Kalinowski T, Obach A, Robaina L, Tort L, Caballero M, et al. Vegetable lipid sources for gilthead seabream (Sparus aurata): effects on fish health. *Aquaculture.* (2003) 225:353–70. doi: 10.1016/S0044-8486(03)00301-6

Conflict of Interest Statement: The authors declare that the research was conducted in the absence of any commercial or financial relationships that could be construed as a potential conflict of interest.

Copyright © 2019 Höglund, Øverli and Winberg. This is an open-access article distributed under the terms of the Creative Commons Attribution License (CC BY). The use, distribution or reproduction in other forums is permitted, provided the original author(s) and the copyright owner(s) are credited and that the original publication in this journal is cited, in accordance with accepted academic practice. No use, distribution or reproduction is permitted which does not comply with these terms.
