# Supplementary material for: 5-Hydroxytryptophan (5-HTP): Natural Occurrence, Analysis, Biosynthesis, Biotechnology, Physiology and Toxicology
Source: Int J Mol Sci. 2020 Dec 26;22(1):181. doi: 10.3390/ijms22010181 (PMC7796270; doi:10.3390/ijms22010181)
Supplement: Supplementary file 1 [file ijms-22-00181-s001.zip › 5-HTP.Data/PDF/3692642565/c5mb00888c.pdf]

Molecular BioSystems

COMMUNICATION

View Article Online
View Journal | View Issue

Open Access Article. Published on 18 March 2016. Downloaded on 12/22/2020 5:05:36 PM. This article is licensed under a Creative Commons Attribution-NonCommercial 3.0 Unported Licence.

CrossMark click for updates

Cite this: *Mol. BioSyst.*, 2016, **12**, 1432

Received 18th December 2015, Accepted 18th March 2016

DOI: 10.1039/c5mb00888c

www.rsc.org/molecularbiosystems

Molecular basis of 5-hydroxytryptophan synthesis in *Saccharomyces cerevisiae*†

Jiantao Zhang,‡[ab] Chaochen Wu,‡[a] Jiayuan Sheng‡[a] and Xueyang Feng*[a]

We report for the first time that 5-hydroxytryptophan can be synthesized in *Saccharomyces cerevisiae* by heterologously expressing prokaryotic phenylalanine 4-hydroxylase or eukaryotic tryptophan 3/5-hydroxylase, together with enhanced synthesis of MH4 or BH4 cofactors. The innate *DFR1* gene in the folate synthesis pathway was found to play pivotal roles in 5-hydroxytryptophan synthesis.

5-Hydroxytryptophan (5-HTP) is an intermediate in the biosynthesis of the neurotransmitter serotonin[1] and has been used in the clinic for over 30 years. 5-HTP can be easily absorbed by the human body and transported across the blood-brain barrier.[2] The therapeutic administration of 5-HTP has shown to be effective in treating depression as well as a wide variety of medical conditions, including fibromyalgia, insomnia, binge eating associated with obesity, cerebellar ataxia, and chronic headaches.[3] Nowadays, since chemical synthesis of 5-HTP is still economically infeasible, 5-HTP is mainly extracted from the seeds of the African plant *Griffonia simplicifolia*,[4–7] which, however, is largely limited by the season- and region-dependent supply of the raw materials.

Recently, the microbial synthesis of 5-HTP has been achieved successfully in *Escherichia coli*,[6,8] which offers an alternative route for producing this small molecule drug. In brief, it was found that the engineered bacterial phenylalanine 4-hydroxylase (P4H) can effectively convert tryptophan to 5-HTP by using tetrahydromonapterin (MH4) as the cofactor.[6,8] Encouraged by the pioneering studies on *de novo* synthesis of 5-HTP in *E. coli*, a model prokaryotic microorganism, in this study we aim to explore the possibility of using eukaryotic organisms such as *S. cerevisiae* to synthesize 5-HTP and uncover the molecular basis of 5-HTP synthesis in eukaryotic cells.

[a] Department of Biological Systems Engineering, Virginia Polytechnic Institute and State University, Blacksburg, VA 24061, USA. E-mail: xueyang@vt.edu

[b] Department of Pharmacology & Toxicology, University of Arizona, Tucson, AZ 85721, USA

† Electronic supplementary information (ESI) available. See DOI: 10.1039/c5mb00888c

‡ JZ, CW and JS contributed to this work equally.

Compared to *E. coli*, *S. cerevisiae* can tolerate harsher fermentation conditions and does not have a phage contamination issue.[9] Such a study, to the best of our knowledge, has not yet been accomplished and little is known about the pathways and cofactors involved in 5-HTP synthesis in eukaryotic organisms. Knowing the molecular basis of 5-HTP synthesis will not only help us understand the mechanisms of 5-HTP synthesis and regulation, but also provide a promising route for large-scale production of 5-HTP using recombinant *S. cerevisiae* as an industrial workhorse. This paper is a proof-of-concept study to reveal the molecular pathways that could lead to 5-HTP synthesis in *S. cerevisiae*. The significance of this work is that we discovered, to the best of our knowledge for the first time, that two metabolic pathways could lead to 5-HTP synthesis in *S. cerevisiae*.

To start, we surveyed some literatures and found two pathways that could potentially lead to 5-HTP synthesis in *S. cerevisiae*. The first pathway uses bacterial P4Hs for 5-HTP synthesis with MH4 used as the cofactor,[6] while the second pathway uses tryptophan 5-hydroxylase (T5H) or tryptophan 3-hydroxylase (T3H) to catalyze 5-HTP synthesis from L-tryptophan by using tetrahydrobiopterin (BH4) as the cofactor[10] (Fig. 1). Since it has been confirmed in *E. coli* that bacterial P4Hs can use MH4

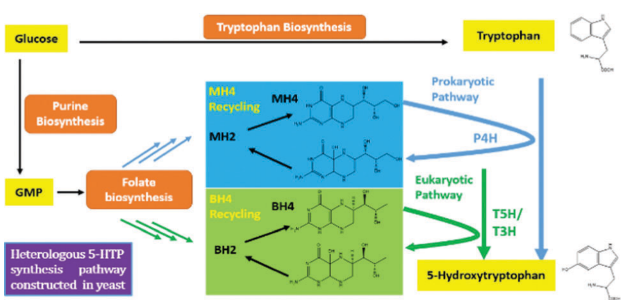

Fig. 1 Molecular basis of 5-HTP biosynthesis revealed in *S. cerevisiae*. Prokaryotic phenylalanine 4-hydroxylase (P4H) or eukaryotic tryptophan 3/5-hydroxylase (T5H/T3H) was found to synthesize 5-HTP by using MH4 or BH4 as a cofactor.

This journal is © The Royal Society of Chemistry 2016

Open Access Article. Published on 18 March 2016. Downloaded on 12/22/2020 5:05:36 PM. This article is licensed under a Creative Commons Attribution-NonCommercial 3.0 Unported Licence.

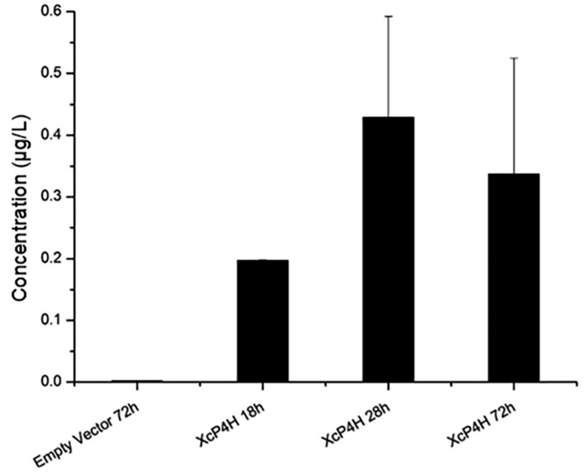

Fig. 2 5-HTP synthesis in *S. cerevisiae* expressing XcP4H from *X. campestris* and MH4 cofactor synthesis pathway. 5-HTP production was detected at 18 h, 28 h and 72 h. No 5-HTP was detected in the control strain harboring an empty vector. All of the samples were measured in triplicate.

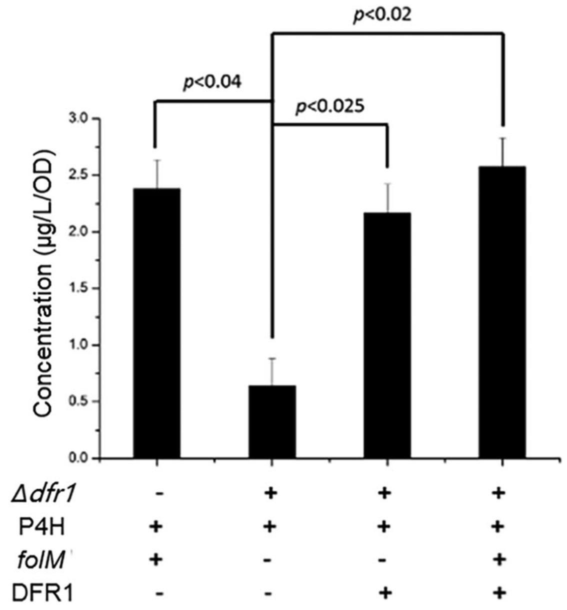

Fig. 3 The role of the *DFR1* gene in 5-HTP synthesis. All of the samples were measured at 72 h in triplicate. Basically, we measured the concentration of 5-HTP produced in yeast as μg L^{-1}. In the meantime, we used the OD600 to measure the cell density. Therefore, the unit of μg per L per OD indicated the cell-based 5-HTP yields in *S. cerevisiae*.

to synthesize 5-HTP, we first constructed the same bacterial pathway in *S. cerevisiae* using the DNA Assembler[4] method (Tables S1 and S2, ESI†). We used strong constitutive promoters to express the XcP4H gene (*i.e.*, encoding an evolved P4H from *X. campestris*) and two genes for MH4 synthesis, *i.e.*, *folM* gene encoding dihydromonapterin reductase (DHMR) from *E. coli* to convert dihydroxymonapetrin (MH2) to MH4; and *phhB* gene encoding pterin-4α-carbinolamine dehydratase from *Pseudomonas aeruginosa* to regenerate MH2.[6] We then transformed the plasmid to the *S. cerevisiae* BY4741 strain *via* LiAc-mediated yeast transformation, and cultured the recombinant yeast cells in SC medium with 2 g L^{-1} tryptophan and 1 mM ascorbic acid in shake flasks. We measured 5-HTP concentrations (Fig. 2) in the medium at 18 h, 28 h, and 72 h using an ELISA kit (Cloud-Clone Corp). As expected, we indeed detected 5-HTP produced by the recombinant *S. cerevisiae* strain, which was synthesized at 0.197 ± 0.001 μg L^{-1}, 0.429 ± 0.163 μg L^{-1}, and 0.337 ± 0.187 μg L^{-1} at 18 h, 28 h, and 72 h, respectively. Compared to the control *S. cerevisiae* strain that only expressed an empty pRS415 plasmid, the 5-HTP concentrations detected at all of the time points were significantly higher ($p < 0.001$). The decreased synthesis of 5-HTP at 72 h was possibly due to the degradation of 5-HTP after the long-time culture in aerobic conditions since 5-HTP is sensitive to oxidation.

Interestingly, we also found that a native *S. cerevisiae* gene, *DFR1*, which encodes dihydrofolate reductase to catalyze tetrahydrofolate synthesis, had a similar role as the *folM* gene in *E. coli*, by surveying the KEGG database and BioCyc database. Considering the bifurcated role of the *folM* gene in recycling MH4 from MH2, we hypothesized that the *DFR1* gene may also have similar function for MH4 synthesis. To test our hypothesis, we constructed a series of recombinant *S. cerevisiae* strains (Table S1, ESI†). As shown in Fig. 3, when we knocked out the *DFR1* gene (Δ*dfr1*) and only expressed XcP4H, the 5-HTP synthesis significantly dropped ($p < 0.04$) from 2.382 ± 0.255 μg per L per OD to 0.520 ± 0.235 μg per L per OD because of the lack of the capability to generate MH4. Then, when we co-expressed the *DFR1* gene along with XcP4H in the context of the forenamed Δ*dfr1* knock-out mutant, the 5-HTP synthesis was fully restored, indicating that the *DFR1* gene could supply MH4 for 5-HTP synthesis. When we further expressed the *DFR1* gene in the Δ*dfr1* mutant with expression of both XcP4H and *folM*, the 5-HTP synthesis was slightly increased, which again suggested a positive role of the *DFR1* gene in 5-HTP synthesis. Taken together, we confirmed that the *DFR1* gene played a pivotal role in 5-HTP synthesis in *S. cerevisiae* by regenerating MH4. It is also worth noting that the removal of the *DFR1* gene did not completely shut down the 5-HTP synthesis, indicating the presence of other innate MH4-regenerating enzymes in *S. cerevisiae*.

In addition to expressing bacterial P4Hs for 5-HTP synthesis with MH4 used as the cofactor, we also explored another possible pathway for 5-HTP synthesis by using eukaryotic genes. 5-HTP is natively produced in humans and animals from L-tryptophan and serves as an important hormone for humans and some animals. The synthesis of 5-HTP was catalysed by tryptophan 5-hydroxylase (T5H) or tryptophan 3-hydroxylase (T3H),[11] both of which use BH4 as the coenzyme. The BH4 recycling pathway was encoded by sepiapterin reductase (SPR) to catalyze the synthesis of BH4 from 6-pyruvoyltetrahydropterin (6PTH),[12] as well as pterin-4-alpha-carbinolamine dehydratase (PCBD) and dihydropteridine reductase (DHPR) to regenerate BH2 and BH4.[13,14] In addition, GTP cyclohydrolase 1 (GCH) and 6-pyruvoyltetrahydropterin synthase (PTS) could produce 6PTH to enhance BH4 supply. To comprehensively explore the potential for 5-HTP synthesis in *S. cerevisiae*, we surveyed various hydroxylases in addition to XcP4H, including P4H from

Open Access Article. Published on 18 March 2016. Downloaded on 12/22/2020 5:05:36 PM.
This article is licensed under a Creative Commons Attribution-NonCommercial 3.0 Unported Licence.

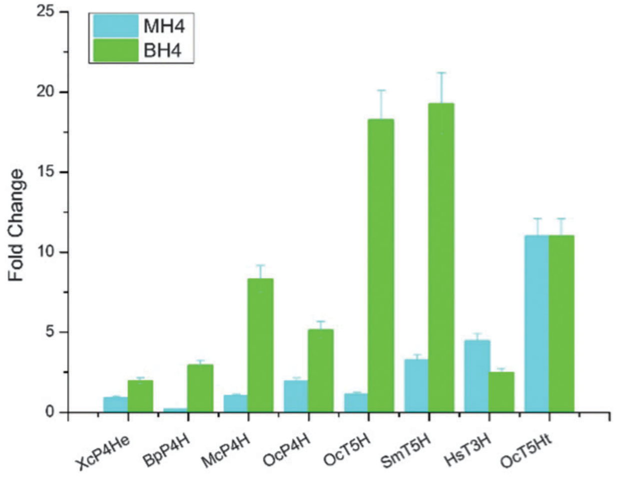

Fig. 4 5-HTP synthesis by expressing different hydroxylases (*i.e.*, P4H, T5H and T3H) and cofactor synthesis pathways (*i.e.*, MH4 pathway and BH4 pathway). All of the samples were measured at 72 h in triplicate, and normalized to the 5-HTP produced by XcP4H using MH4 as the cofactor.

Burkholderia pseudomallei (BpP4H); P4H from *Mesorhizobium ciceri biovar biserrulae* (McP4H); T5H from *Oryctolagus cuniculu* (OcT5H); a truncated version of OcT5H (OcT5Ht); T5H from *Schistosoma mansoni* (SmT5H); P4H from *Oryctolagus cuniculus* (OcP4H); and T3H from *Homo sapiens* (HsT3H). We also constructed a MH4 biosynthesis pathway by expressing *folM* from *E. coli* and *phhb* from *P. aeruginosa*; and a BH4 biosynthesis pathway by expressing PTS and SPR from *Rattus norvegicus*, DHPR and GCH from *E. coli*, and PCBD from *P. aeruginosa* in pRS416 plasmids of *S. cerevisiae*, respectively (Table S1, ESI†). We then combined different hydroxylases with either the MH4 or BH4 biosynthesis pathway, and characterized the 5-HTP synthesis at 72 h by cultivating the recombinant *S. cerevisiae* strains in SC medium with 2 g L^{-1} tryptophan and 1 mM ascorbic acid in shake flasks (Fig. 4). We found that OcT5H and SmT5H using BH4 as cofactors were the top 2 hydroxylases that led to over a 17 fold increase of 5-HTP synthesis compared to that of XcP4H using MH4 as cofactors. Except for HsT3H, BH4 was preferred to being used as the cofactors by all of the hydroxylases for 5-HTP synthesis in *S. cerevisiae*, even for those from bacteria (*i.e.*, XcP4H, BpP4H, McP4H, and OcP4H). The flexibility of using either MH4 or BH4 as the cofactors might due to their similar structures and/or the low substrate specificity of different hydroxylases.

We also applied western blotting to characterize the expression levels of XcP4H, BpP4H, OcT5H, and SmT5H. These four enzymes were chosen since XcP4H and BpP4H led to the lowest production of 5-HTP in yeast while OcT5H and SmT5H led to the highest production. As shown in Fig. S1 (ESI†), we found that the expression levels of different enzymes varied but were not positively correlated with the 5-HTP synthesis. For example, OcT5H had a similar expression level as BpP4H but led to >7 fold higher production of 5-HTP when using BH4 as the cofactor. SmT5H had a higher expression level than OcT5H but led to a similar amount of 5-HTP being produced when using BH4 as the cofactor. In addition, we measured the *in vitro* enzyme activities of XcP4H, BpP4H, OcT5H, and SmT5H. As shown in Fig. S2 (ESI†), we found that all of the enzymes demonstrated higher activities when using BH4 as the cofactor. The activities of OcT5H and SmT5H were 50–75% higher than that of XcP4H and BpP4H when using BH4 as the cofactor. Taken together, the western blotting and the measurement of *in vitro* enzyme activities indicated that T5Hs could be a better candidate when applying metabolic engineering to improve 5-HTP production in yeast.

To conclude, in this study, we discovered that two pathways could lead to 5-HTP synthesis in *S. cerevisiae*, by using either MH4 or BH4 as the cofactor and the innate *DFR1* gene in *S. cerevisiae* was critical in recycling MH4 for 5-HTP synthesis. It is the first time, to the best of our knowledge, that the molecular basis of 5-HTP synthesis was revealed in a eukaryotic organism, which paves the ways for future metabolic engineering (*e.g.*, increasing the supply of tryptophan precursor and cofactors) to promote yeast-based production of 5-HTP.

This work was supported by start-up fund from Virginia Tech (#175323).

Notes and references

1 D. M. Niu, Disorders of BH4 metabolism and the treatment of patients with 6-pyruvoyl-tetrahydropterin synthase deficiency in Taiwan, *Brain Dev.*, 2011, **33**, 847–855, DOI: 10.1016/j.braindev.2011.07.009.

2 E. A. Bell and D. H. Janzen, Medical and ecological considerations of L-dopa and 5-HTP in seeds, *Nature*, 1971, **229**, 136–137.

3 T. C. Birdsall, 5-Hydroxytryptophan: a clinically-effective serotonin precursor, *Alternative medicine review: a journal of clinical therapeutic*, 1998, **3**, 271–280.

4 G. Carnevale, V. Di Viesti, M. Zavatti and P. Zanoli, Anxiolytic-like effect of Griffonia simplicifolia Baill. seed extract in rats, *Phytomedicine*, 2011, **18**, 848–851, DOI: 10.1016/j.phymed.2011.01.016.

5 P. A. Lemaire and R. K. Adosraku, An HPLC method for the direct assay of the serotonin precursor, 5-hydroxytrophan, in seeds of Griffonia simplicifolia, *Phytochem. Anal.*, 2002, **13**, 333–337, DOI: 10.1002/pca.659.

6 Y. Lin, X. Sun, Q. Yuan and Y. Yan, Engineering bacterial phenylalanine 4-hydroxylase for microbial synthesis of human neurotransmitter precursor 5-hydroxytryptophan, *ACS Synth. Biol.*, 2014, **3**, 497–505, DOI: 10.1021/sb5002505.

7 E. H. Turner, J. M. Loftis and A. D. Blackwell, Serotonin a la carte: supplementation with the serotonin precursor 5-hydroxytryptophan, *Pharmacol. Ther.*, 2006, **109**, 325–338, DOI: 10.1016/j.pharmthera.2005.06.004.

8 X. Sun, Y. Lin, Q. Yuan and Y. Yan, Precursor-Directed Biosynthesis of 5-Hydroxytryptophan Using Metabolically Engineered *E. coli*, *ACS Synth. Biol.*, 2015, **4**, 554–558, DOI: 10.1021/sb500303q.

9 K. K. Hong and J. Nielsen, Metabolic engineering of *Saccharomyces cerevisiae*: a key cell factory platform for

Open Access Article. Published on 18 March 2016. Downloaded on 12/22/2020 5:05:36 PM.
This article is licensed under a Creative Commons Attribution-NonCommercial 3.0 Unported Licence.

future biorefineries, *Cell. Mol. Life Sci.*, 2012, **69**, 2671–2690, DOI: 10.1007/s00018-012-0945-1.

10 L. Pan, *et al.*, Erratum: GTP-cyclohydrolase deficiency responsive to sapropterin and 5-HTP supplementation: relief of treatment-refractory depression and suicidal behaviour, *BMJ*, 2011, DOI: 10.1136/bcr.03.2011.3927.corr1.

11 H. Imura, Y. Nakai and T. Yoshimi, Effect of 5-hydroxytryptophan (5-HTP) on growth hormone and ACTH release in man, *J. Clin. Endocrinol. Metab.*, 1973, **36**, 204–206, DOI: 10.1210/jcem-36-1-204.

12 H. Haruki, R. Hovius, M. Gronlund Pedersen and K. Johnsson, Tetrahydrobiopterin Biosynthesis as a Potential Target of the Kynurenine Pathway Metabolite Xanthurenic Acid, *J. Biol. Chem.*, 2016, **291**, 652–657, DOI: 10.1074/jbc.C115.680488.

13 H. Myllykallio, D. Leduc, J. Filee and U. Liebl, Life without dihydrofolate reductase FolA, *Trends Microbiol.*, 2003, **11**, 220–223.

14 J. T. Tsay, *et al.*, Kinetic investigation of the functional role of phenylalanine-31 of recombinant human dihydrofolate reductase, *Biochemistry*, 1990, **29**, 6428–6436.
