# Supplementary material for: 5-Hydroxytryptophan (5-HTP): Natural Occurrence, Analysis, Biosynthesis, Biotechnology, Physiology and Toxicology
Source: Int J Mol Sci. 2020 Dec 26;22(1):181. doi: 10.3390/ijms22010181 (PMC7796270; doi:10.3390/ijms22010181)
Supplement: Supplementary file 1 [file ijms-22-00181-s001.zip › 5-HTP.Data/PDF/3721827945/Rabbani-2017--hydroxytryptophan-a-major-produc.pdf]

Virology 503 (2017) 46–51

Contents lists available at ScienceDirect

Virology

journal homepage: www.elsevier.com/locate/yviro

Brief Communication

5-Hydroxytryptophan, a major product of tryptophan degradation, is essential for optimal replication of human parainfluenza virus

M.A.G Rabbani, Sailen Barik*

Department of Biological, Geological and Environmental Sciences, and Center for Gene Regulation in Health and Disease, Cleveland State University, 2121 Euclid Avenue, Cleveland, OH 44115, United States

ARTICLE INFO

Keywords:
Tryptophan
ISG
Paramyxovirus
Parainfluenza virus
IDO
5-HTP
Serotonin
Melatonin
Kynurenine

ABSTRACT

Interferon (IFN) exerts its antiviral effect by inducing a large family of cellular genes, named interferon (IFN)-stimulated genes (ISGs). An intriguing member of this family is indoleamine 2,3-dioxygenase (IDO), which catalyzes the first and rate-limiting step of the main branch of tryptophan (Trp) degradation, the kynurenine pathway. We recently showed that IDO strongly inhibits human parainfluenza virus type 3 (PIV3), a significant respiratory pathogen. Here, we show that 5-hydoxytryptophan (5-HTP), the first product of an alternative branch of Trp degradation and a serotonin precursor, is essential to protect virus growth against IDO in cell culture. We also show that the apparent antiviral effect of IDO on PIV3 is not due to the generation of the kynurenine pathway metabolites, but rather due to the depletion of intracellular Trp by IDO, as a result of which this rare amino acid becomes unavailable for the alternative, proviral 5-HTP pathway.

1. Introduction

IDO (indolamine 2–3 dioxygenase) is an IFN-stimulated enzyme that catalyzes the first step of the major arm of the tryptophan degradation pathway that leads to the generation of kynurenine and hence commonly called the kynurenine pathway (Fig. 1). Being the rate-limiting enzyme of this pathway, IDO is considered strategically important for tryptophan homeostasis (Mellor and Munn, 2003). Most cells at basal state contain little or no IDO, but the gene is transcriptionally induced by IFN, and hence its designation as an ISG (IFN-stimulated gene) (Obojes et al., 2005; Ozaki et al., 1988). Historically, IFN was discovered as a cytokine that interferes with viral growth, and therefore, the ISGs, the final products of the IFN response pathway, were considered antiviral (Borden et al., 2007). In fact, over the years, several ISGs have been demonstrated to possess antiviral activities (Schoggins and Rice, 2011). However, IDO was originally shown to inhibit the replication of the unicellular protozoan parasite *Toxoplasma gondii* in immune cells (Dai et al., 1994; Mehraj and Routy, 2015; Mellor and Munn, 2003). It is only recently that the relationship of IDO with viral pathogenesis and the antiviral role of IDO have been appreciated, notably with measles virus, herpes simplex virus, hepatitis B virus, influenza virus and respiratory syncytial virus (Adams et al., 2004; Huang et al., 2013; Mao et al., 2011; Obojes et al., 2005; Rabbani et al., 2016; Sage et al., 2014). The majority of these studies were conducted in cells of immune origin, such as macrophages and dendritic cells (DC), or in the live animal, which indicated a role of IDO in regulating adaptive immunity (Mellor and Munn, 2003; Schmidt and Schultze, 2014). Whether IDO or Trp depletion had a direct effect on viral replication in nonimmune cells remained largely unexplored.

Our laboratory has recently shown that the parainfluenza virus type 3 (HPIV3), an agent of acute respiratory disease, is also strongly inhibited by IDO in lung epithelial A549 cells (Rabbani et al., 2016), suggesting a nonimmune mechanism. Here, we pursue the mechanism of this inhibition through the use of various catabolic products of Trp degradation and inhibitors of the degradative enzymes. The catabolism of Trp occurs in two parallel, non-overlapping and mutually exclusive pathways (Fig. 1) (Le Floc'h et al., 2011). The kynurenine pathway, initiated by IDO, generates kynurenine as the second product (Schwarcz et al., 2012). The other pathway is initiated by Trp hydroxylase (TPH), and 5-hydroxytryptophan (5-HTP) is its first product; we will refer it as the 5-HTP pathway (Fig. 1) (Berger et al., 2009). Since the antiviral effect of IDO could be overcome by addition of Trp to the medium (Adams et al., 2004; Mao et al., 2011; Obojes et al., 2005; Rabbani et al., 2016), depletion of intracellular Trp levels has been considered the major reason of the antiviral effect of IDO, although the mechanism has remained unknown. In this communication, we show that the antiviral effect of IDO on PIV3 can be counteracted not only by Trp but also by 5-HTP (Fig. 1). We propose that when IDO is induced, such as upon IFN treatment, it turns the kynurenine

* Corresponding author.
E-mail address: barikfamily@gmail.com (S. Barik).

http://dx.doi.org/10.1016/j.virol.2017.01.007
Received 17 December 2016; Received in revised form 10 January 2017; Accepted 11 January 2017
Available online 20 January 2017
0042-6822/ © 2017 Elsevier Inc. All rights reserved.

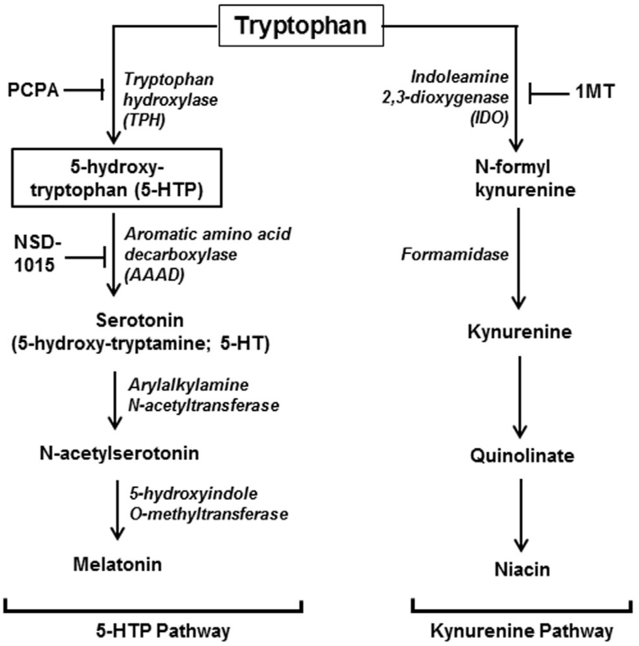

Fig. 1. Two pathways of tryptophan degradation. The pathways have been tentatively named after a major product of each, namely 5-HTP (left) and kynurenine (right). Trp and 5-HTP, the two compounds that exhibited the highest proviral activity in cell culture, are boxed. The enzymes and their inhibitors are shown for the relevant steps of the two pathways. Of note, the first reaction of each pathway, catalyzed by TPH and IDO respectively, is the rate-limiting step of the pathway. All reactions in both pathways are irreversible.

pathway on, leaving little or no Trp to generate 5-HTP, thus restricting viral replication. Our results constitute a novel paradigm for the role of Trp catabolism and establish a cell-intrinsic proviral role of 5-HTP in host-virus interaction.

2. Materials and methods

2.1. Cells, virus, IFN

These have been described previously (Rabbani et al., 2016). In brief, all experiments were performed in A549 cells, which are human alveolar carcinoma type II-like and of epithelial origin. Human parainfluenza virus type 3 JS strain was grown and assayed in monkey kidney epithelial LLC-MK2 cells. Cell-free viral titer was determined by serial dilution and foci/plaque assay. Both cells were grown in monolayer in Dulbecco's minimum essential media (D-MEM) with high-glucose, L-glutamine, and sodium pyruvate (Caisson Laboratories Inc; DML10), heat-inactivated fetal bovine serum (10%) (Fisher Scientific; 03-600-511), penicillin (100 IU/ml) and streptomycin (100 µg/ml) (Mediatech Inc; 30-002-CI). In all infections experiments, PIV3 was added at an m.o.i. of 1.0. Recombinant human IFN-γ (BD Biosciences; cat# 554616) and its use in cell culture (at a final concentration of 60 ng/ml) were described previously (Rabbani et al., 2016).

2.2. Tryptophan metabolites and enzyme inhibitors

The following Trp metabolites and the indicated enzyme inhibitors were all from Sigma-Aldrich (St. Louis, MO): L-tryptophan (cat# T0254), L-kynurenine (cat# K8625), 5-hydroxy-L-tryptophan (cat# H9772), serotonin hydrochloride (cat# H9523), melatonin (cat# M5250), 1-methyl-D-tryptophan (1-MT, inhibitor of IDO; cat# 452483), *p*-chlorophenylalanine (PCPA, inhibitor of Trp hydroxylase; cat# C6506), 3-hydroxybenzylhydrazine dihydrochloride (NSD-1015, inhibitor of aromatic amino acid decarboxylase; cat# 54880). To make stock solutions, all compounds were dissolved in nuclease-free sterile water (Ambion; cat#AM9937), with the exception that melatonin was dissolved in DMSO (Fisher Scientific; cat# BP231) and PCPA in HCl (Sigma; cat# H1758). Appropriate aliquots of the stock solutions were added just prior to infection to ensure that the chemicals were present during the full course of the infection.

2.3. Antibodies and immunoblot

Custom rat antibody was made commercially (Biosynthesis, Inc., Lewisville, TX) against the synthetic peptide KRNQEINQLISPRPSTSLNS of PIV3 nonstructural protein C as described previously (Rabbani et al., 2016). Commercial primary antibodies used in immunoblotting included PIV3 HN antibody (Abcam; cat# ab49756), IDO antibody (Santa Cruz; cat# sc-25809), mouse glyceraldehyde-3-phosphate dehydrogenase (GAPDH) antibody (G-9; Santa Cruz; cat# sc-365062). The corresponding horseradish peroxidase-conjugated secondary antibodies were purchased from Santa Cruz Biotechnology: goat anti-mouse IgG (cat# sc-2031), goat anti-rabbit IgG (cat# sc-2030), and goat anti-rat IgG (cat# sc-2032). Lysis of cells, SDS-PAGE and immunoblotting were also performed as described (Rabbani et al., 2016).

2.4. Data plot

Graphs were plotted in Excel and the bars on each data point represented "Mean +/− S.D." of three values.

3. Results

3.1. Metabolites of the kynurenine pathway of Trp degradation have no effect on PIV3 replication

We have recently shown that exogenously added tryptophan (Trp) counteracted the antiviral effect of indoleamine 2–3 dioxygenase (IDO), produced from either a recombinant IDO transgene or by induction of the endogenous gene by IFN (Rabbani et al., 2016). These results suggested that depletion of intracellular Trp levels may be detrimental to the virus. In the current studies, we further explored the role of IDO and the ensuing kynurenine pathway. First, we asked if direct pharmacological inhibition of IDO would help the virus. We induced IDO by IFN-γ as before (Rabbani et al., 2016), and inhibited its enzymatic activity by adding 1-MT (1-methyl-D-tryptophan) (Uyttenhove et al., 2003). Since the exact inhibitory constants of 1-MT for IDO are not known, we have used relatively high concentrations (50–490 µM), in the same range as Trp, in case it is functioning as a competitive enzyme inhibitor. Results (Fig. 2) show that, as with exogenously added Trp (Fig. 2A and B), increasing amounts of the 1-MT also rescued PIV3 replication (Fig. 2C), measured by the intracellular syntheses of two viral proteins, the nonstructural protein, C, and the structural protein, HN (hemagglutinin). Since IDO is antiviral and the action of IDO on Trp turns on the kynurenine pathway, we postulated that the metabolites of this pathway cannot be proviral. They may also not be antiviral, since if they were, the addition of Trp would have had an antiviral effect by generating more of those metabolites. To test these predictions directly, we added kynurenine to the cell culture and tested its effect on PIV3 growth. Indeed, kynurenine neither stimulated nor inhibited PIV3 replication even at a high concentration of 490 µM (Fig. 2D). We conclude that the antiviral effect of IDO is due to reduced levels of Trp and not from the increased levels of the Trp degradation products of the kynurenine pathway.

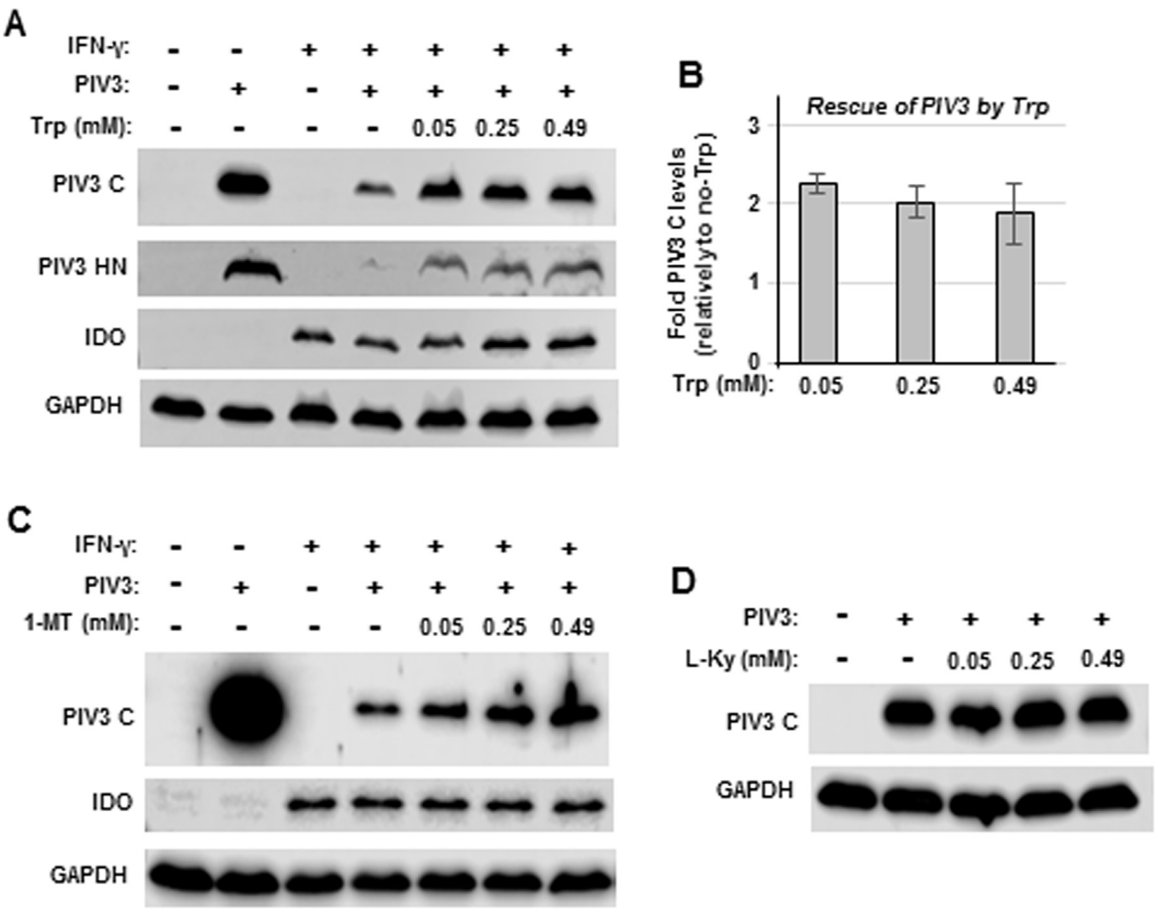

Fig. 2. Lack of a role of the kynurenine pathway in intracellular PIV3 growth. The indicated compounds at a range of final concentrations were added to A549 cells in culture, and PIV3 growth was measured by immunoblot of the viral C protein as described in Materials and Methods. Where shown, IDO was also measured as being induced by IFN-γ treatment. The different panels show the following: Enhancement of PIV3 growth by Trp (A) and by the IDO inhibitor, 1-MT (C), but no effect of kynurenine, a downstream product of the pathway (D). Densitometry (B) of the immunoblot in Panel A clearly reveals the rescuing role of Trp. In panel (A), PIV3 HN (hemagglutinin), a viral structural protein, was also assayed as an additional readout of viral growth, which matched the result of the nonstructural C protein blot.

3.2. Specific metabolites of the 5-HTP pathway of Trp degradation stimulate PIV3 replication

To understand how lower Trp levels caused by IDO may reduce PIV3 replication, we hypothesized that the depletion of Trp abrogates the production of some products of Trp catabolism outside the kynurenine pathway that promote virus growth. As mentioned, the major non-kynurenine pathway of Trp degradation is the 5-HTP pathway, which converts Trp sequentially into 5-HTP, serotonin and melatonin (Fig. 1). We, therefore, tested the ability of these three compounds to facilitate PIV3 growth in IFN-γ-treated A549 cells, in which IDO was induced. The results (Fig. 3) clearly showed that in the IDO-expressing cell, 5-HTP is the major rescuer of PIV3 growth (Fig. 3AB), while serotonin rescued relatively weakly (Fig. 3C). Melatonin was the weakest of the three (Fig. 3D), showing only marginal improvement of viral growth.

3.3. 5-HTP promotes productive PIV3 replication

To test if the intracellular increase in viral protein levels is actually translated into liberated, infectious progeny virus, we assayed viral plaque-forming units released in the culture media at different times post-infection. These results (Fig. 3E) complemented the immunoblot results, showing that the aforementioned metabolites were indeed effective in rescuing the full viral life cycle from IDO-mediated inhibition.

3.4. 5-hydroxytryptophan protects PIV3 growth against IDO

The results presented so far show that Trp, the parent amino acid, and 5-HTP, its immediate degradation product, are the strongest enhancers of PIV3 replication. However, because each compound can be converted to its downstream products, we needed to block the conversion to determine whether the compounds are proviral by themselves. To this end, we again resorted to specific enzyme inhibitors. We used *p*-chlorophenylalanine (PCPA) to inhibit Trp hydroxylase (THP) (Schechter, 1991) that catalyzes the first step of the pathway (Trp→5-HTP), and 3-hydroxybenzylhydrazine dihydrochloride (NSD-1015) to inhibit aromatic amino acid decarboxylase (AAAD) (Honig et al., 2009) that catalyzes the next step (5-HTP→serotonin) (Fig. 1). Thus, PCPA would block the production of all metabolites of this pathway, whereas NSD-1015 would allow the production of 5-HTP but block the production of all other metabolites, since they are downstream of 5-HTP. Results (Fig. 4) show that Trp was unable to rescue PIV3 when its conversion to 5-HTP was blocked (Fig. 4A). In contrast, the ability of 5-HTP to rescue the virus was not affected when its conversion to downstream compounds (such as serotonin and melatonin) was blocked (Fig. 4B). We conclude that 5-HTP is the true proviral factor, and Trp only stimulates virus replication because it is converted to 5-HTP. Thus, conversion to serotonin is not required for the proviral role of 5-HTP, although serotonin itself appears to have some proviral effect of its own.

We then asked whether the amount of 5-HTP is rate-limiting for viral growth in normal cells that has not been treated with IFN. To test

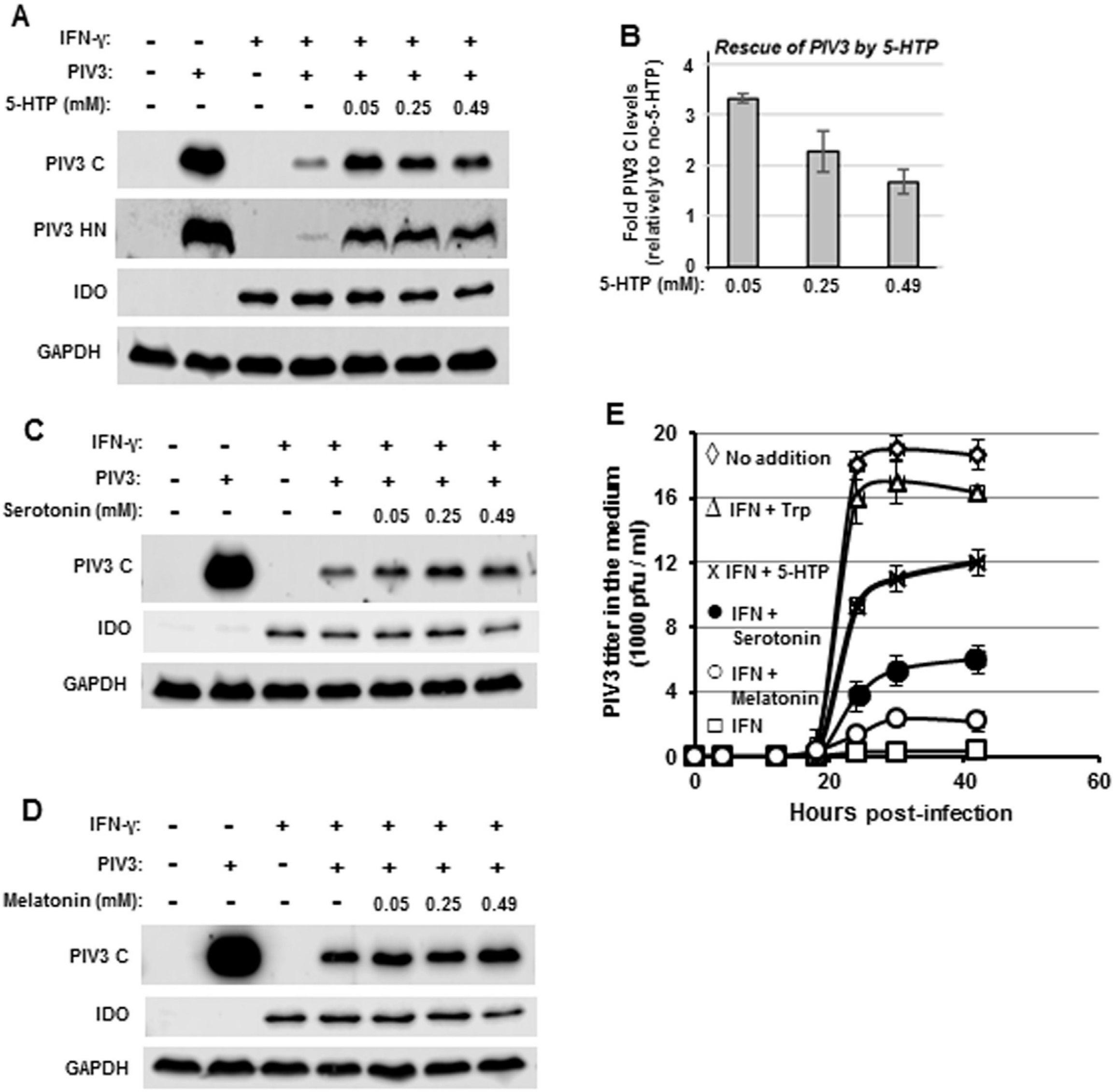

Fig. 3. Assessment of the 5-HTP pathway metabolites in PIV3 growth and release. The ability of three major members of the 5-HTP pathway (Fig. 1) to rescue PIV3 growth in IDO-expressing (induced by IFN-γ) A594 cells was assayed by immunoblot and extracellular infectious virus assay. As seen, (A, B) 5-HTP had the strongest stimulatory effect on the virus, serotonin had a modest effect (C), and melatonin had little or no effect (D). GAPDH serves as the loading control. As in Fig. 2A, PIV3 HN (hemagglutinin) was also assayed in Panel A as an additional readout of viral growth, which paralleled the results of PIV3 C blot. (E) Rescue of progeny PIV3 virus production in the cell culture media. A549 cells were infected with PIV3 in the presence of the indicated compounds of 5-HTP pathway, and media collected at various times post-infection (4 h, 12 h, 18 h, 24 h, 30 h, 42 h) were assayed for infectious virus by serial dilution and plaque assay on LLC-MK2 cells as described previously (Majumdar et al., 2016). Graphing was performed as described in Materials and Methods.

this, we added increasing amounts of 5-HTP to the A549 culture media and measured PIV3 growth by quantitative immunoblot as before. The results (Fig. 4C) showed no increase in virus growth upon 5-HTP addition, thus confirming that the normal cellular content of 5-HTP is sufficient for optimal PIV3 growth. This is in agreement with our hypothesis that the proviral role of 5-HTP becomes apparent only when the Trp levels are reduced by IDO, leaving insufficient amounts of Trp to be converted to 5-HTP.

4. Discussion

In this brief communication, we report that 5-hydoxytryptophan (5-HTP), the very first product of one of the two major tryptophan degradation pathways, is a strong proviral factor for PIV3. Since the discovery of IDO as an antiviral host enzyme, much attention has been paid to the IDO-led kynurenine pathway in search of a mechanism (Mellor and Munn, 2003; Schmidt and Schultze, 2014) focusing almost exclusively on possible immunoregulatory role of kynurenine involving immune cells such as T cells and dendritic cells, while the parallel, 5-HTP pathway escaped attention (Watzlawik et al., 2016). Our studies, however, were conducted in cell culture, whereby the replication of PIV3 in lung epithelial A549 cells was quantified. Thus, the proviral role of 5-HTP that we have discovered is direct and cell-intrinsic, not reliant on immunological or humoral factors. Clearly, the molecular mechanism of this unique proviral role of 5-HTP will require additional studies. It will also be useful to determine whether 5-HTP can rescue other viruses that are inhibited upon IDO induction, which may help identify a common molecular target. For instance, the large subunit (L) of the viral RNA-dependent RNA polymerase is essential for RNA synthesis in all nonsegmented, negative-strand RNA viruses (Order *Mononegavirales*) and is also highly similar in primary structure (Poch et al., 1989); it could, therefore, serve as a metabolite sensor, inhibited by Trp deprivation and activated by 5-HTP through a novel mechanism.

We would like to note that we have arbitrarily chosen the concentrations of the enzyme inhibitors in the same range as the

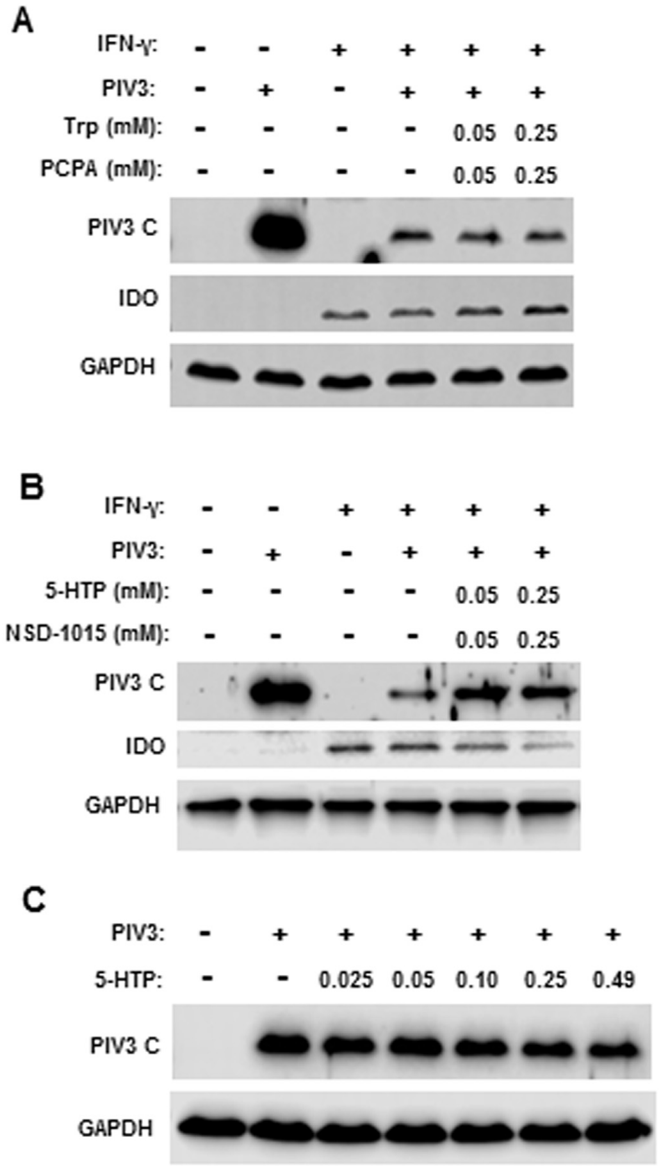

Fig. 4. Dissection of the role of 5-HTP pathway metabolites in PIV3 growth. The ability of (A) Trp and (B) 5-HTP to rescue intracellular PIV3 growth in IDO-expressing (IFN-γ-induced) A594 cells was measured essentially as described for Fig. 3, in the presence of the respective inhibitors of their downstream converting enzymes. As seen, PCPA inhibited the proviral effect of Trp, whereas NSD-1015 did not inhibit the proviral effect of 5-HTP. (C) Addition of 5-HTP to standard, IFN-untreated A549 cells had no enhancing effect on PIV3 growth. In all panels, GAPDH serves as the loading control.

catabolite compounds, since the inhibitory constants are not exactly known. Thus, we cannot be absolutely sure that we achieved 100% inhibition of the enzymes. However, this should not affect our conclusions due to the nature of the results obtained. For example, incomplete inhibition of IDO by 1-MT will allow some kynurenine synthesis, but even high concentrations of kynurenine had no effect on virus growth. Similarly, if there is residual activity of AAAD due to incomplete inhibition by NSD-1015, a small amount of serotonin will be produced, but serotonin has very low proviral activity anyway. On the other hand, inhibition of TPH by PCPA must have been strong enough to inhibit 5-HTP synthesis since PIV3 growth was not enhanced. Use of RNAi to knockdown the relevant enzymes would be an alternative, although it too can be incomplete and can have off-target effects (Svoboda, 2007). Future studies may explore the use of gene knockout mutants to further investigate the role of these enzymatic steps in virus replication.

We have measured the proviral activity of all the products of the 5-HTP pathway, except N-acetyl serotonin, because it is commercially unavailable. The activities seem to follow a decreasing order down the pathway: Trp > 5-HTP > serotonin > > melatonin. We do not know the reason for this order, but would like to draw attention to an important difference between the two Trp degradation pathways. The kynurenine pathway destroys the central aromatic core of Trp in its very first enzymatic step, i.e. in the IDO reaction, causing an oxidative reduction of the indole ring of Trp. The 5-HTP pathway, in sharp contrast, retains the aromatic ring structure of Trp through all the compounds of the pathway. We presume that the structural similarity with Trp allows a certain degree of functional similarity among the 5-HTP catabolites, which may underlie their Trp-like proviral ability. The ability clearly decreases as the compounds gradually veer farther and farther from the structure of Trp. Perhaps Trp, in its proviral role, needs to bind to a protein, and the binding pocket fits the compounds with an efficiency that is proportional to their structural similarity with Trp.

The high proviral activity of 5-HTP also suggests that the steady state level of 5-HTP may be maintained long enough for it to carry out its proviral role. In other words, the proviral activity of 5-HTP may be elicited faster than the action of the AAAD, the enzyme that converts 5-HTP into serotonin (Fig. 1).

Tryptophan holds a unique place in biology. It is the rarest and largest amino acid, nutritionally essential in mammals, and requires the most energy to biosynthesize. Mirroring its rarity, it is also the only amino acid besides methionine that is coded for by a single codon (UGG). Thus, it stands to reason that intracellular Trp levels may act as a sensitive rheostat to regulate biochemical reactions. As a result, several products of the two Trp catabolic pathways described here play important roles in metabolism and medicine (Bai et al., 2016; Morris et al., 2016; Watzlawik et al., 2016). Niacin, the end product of the kynurenine pathway (Fig. 1), is long recognized as an essential vitamin, deficiency of which causes 'pellagra', a form of dermatitis. Kynurenine, serotonin and melatonin (Fig. 1), along with tryptamine, are considered as hormones because of their strategic metabolic roles in several organs. Serotonin, in particular, is a major monoamine neurotransmitter that contributes to hypertension and affects brain functions such as cognitive behavior, happiness, mood, sleep and memory (Monti, 2011; Schechter, 1991; Watts, 2016). It is clinically used to treat conditions such as depression, nausea, public fear and anxiety disorder, and hence, its precursor, 5-HTP, also has similar effect (Turner et al., 2006). Melatonin is used to treat sleep disorders, including jet lag, although its mechanism is unclear. Since the two Trp catabolism pathways are mutually exclusive, they compete for the same intracellular Trp pool; as a result, flow of one pathway would deprive the other. Specifically, an unregulated high level of IFN-γ may lead to chronic induction of IDO, which would direct Trp to the kynurenine pathway, causing a deficiency in 5-HTP, serotonin and melatonin, and promoting neuropsychiatric disorders, which is currently being appreciated in the context of Parkinson's Disease (Anderson et al., 2016). In our case, this would also inhibit PIV3 growth, thus explaining the antiviral property of IDO as an ISG. Our results show that among all the Trp catabolites, 5-HTP is the strongest stimulator of PIV3 replication, and thus, its steady-state level should be a key regulator of virus growth. It is also imperative that clinical administration of serotonin may have some stimulatory effect on PIV3 growth, but that of melatonin will have no effect.

Based on the aforesaid, it is clear that the steady-state level of 5-HTP can be fine-tuned by several factors: whereas dietary intake of Trp and TPH enzyme activity will elevate the level, IDO and AAAD activities will lower it. Correlating these activities and the viral susceptibility of the host cell under various physiological and clinical conditions will constitute stimulating new areas of future research.

5. Conclusions

In this report, using human parainfluenza virus type 3 (PIV3) as our model virus, we have unraveled a potential mechanism for the antiviral effect of indoleamine 2,3-dioxygenase (IDO), a major interferon-stimulated gene (ISG). Our results suggest that IDO activity, which channels tryptophan into the kynurenine pathway, leaves little of this rare amino acid available for the parallel pathway that converts tryptophan sequentially into 5-hydroxytryptophan (5-HTP), serotonin and melatonin. Of these three products, 5-HTP was the strongest stimulator of PIV3 growth, followed by serotonin. These findings, while establishing 5-HTP as a robust proviral host factor for the first time, also indicate that the balance between the two tryptophan degradation pathways may regulate viral susceptibility of the host, which is a new paradigm in innate immunity.

Acknowledgements

This research was supported in part by National Institutes of Health (Grant AI109569).

References

Adams, O., Besken, K., Oberdörfer, C., MacKenzie, C.R., Rüssing, D., Däubener, W., 2004. Inhibition of human herpes simplex virus type 2 by interferon gamma and tumor necrosis factor alpha is mediated by indoleamine 2,3-dioxygenase. Microbes Infect. 6, 806–812.
Anderson, G., Seo, M., Berk, M., Carvalho, A.F., Maes, M., 2016. Gut permeability and microbiota in Parkinson's disease: role of depression, tryptophan catabolites, oxidative and nitrosative stress and melatoninergic pathways. Curr. Pharm. Des. 2016, (Epub ahead of print).
Berger, M., Gray, J.A., Roth, B.L., 2009. The expanded biology of serotonin. Ann. Rev. Med. 60, 355–366.
Bai, M., Liu, H., Xu, K., Oso, A.O., Wu, X., Liu, G., Tossou, M.C., Al-Dhabi, N.A., Duraipandiyan, V., Xi, Q., Yin, Y., 2016. A review of the immunomodulatory role of dietary tryptophan in livestock and poultry. Amino Acids 2016, (Epub ahead of print).
Borden, E.C., Sen, G.C., Uze, G., Silverman, R.H., Ransohoff, R.M., Foster, G.R., Stark, G.R., 2007. Interferons at age 50: past, current and future impact on biomedicine. Nat. Rev. Drug Discov. 6, 975–990.
Dai, W., Pan, H., Kwok, O., Dubey, J.P., 1994. Human indoleamine 2,3-dioxygenase inhibits Toxoplasma gondii growth in fibroblast cells. J. Interferon Res. 14, 313–317.
Honig, G., Jongsma, M.E., van der Hart, M.C., Tecott, L.H., 2009. Chronic citalopram administration causes a sustained suppression of serotonin synthesis in the mouse forebrain. PLoS One 4, e6797.
Huang, L., Li, L., Klonowski, K.D., Tompkins, S.M., Tripp, R.A., Mellor, A.L., 2013. Induction and role of indoleamine 2,3 dioxygenase in mouse models of influenza A virus infection. PLoS One 8, e66546.
Le Floc'h, N., Otten, W., Merlot, E., 2011. Tryptophan metabolism, from nutrition to potential therapeutic applications. Amino Acids 41, 1195–1205.
Majumdar, T., Dhar, J., Patel, S., Kondratov, R., Barik, S., 2016. Circadian transcription factor BMAL1 regulates innate immunity against select RNA viruses. Innate Immun., (pii: 1753425916681075. Epub ahead of print).
Mao, R., Zhang, J., Jiang, D., Cai, D., Levy, J.M., Cuconati, A., Block, T.M., Guo, J.T., Guo, H., 2011. Indoleamine 2,3-dioxygenase mediates the antiviral effect of gamma interferon against hepatitis B virus in human hepatocyte-derived cells. J. Virol. 85, 1048–1057.
Mehraj, V., Routy, J.P., 2015. Tryptophan catabolism in chronic viral infections: handling uninvited guests. Int J. Tryptophan Res. 8, 41–48.
Mellor, A.L., Munn, D.H., 2003. Tryptophan catabolism and regulation of adaptive immunity. J. Immunol. 170, 5809–5813.
Monti, J.M., 2011. Serotonin control of sleep-wake behavior. Sleep. Med. Rev. 15, 269–281.
Morris, G., Berk, M., Carvalho, A., Caso, J.R., Sanz, Y., Walder, K., Maes, M., 2016. The role of the microbial metabolites including tryptophan catabolites and short chain fatty acids in the pathophysiology of immune-inflammatory and neuroimmune disease. Mol. Neurobiol. 2016, (Epub ahead of print).
Obojes, K., Andres, O., Kim, K.S., Däubener, W., Schneider-Schaulies, J., 2005. Indoleamine 2,3-dioxygenase mediates cell type-specific anti-measles virus activity of gamma interferon. J. Virol. 79, 7768–7776.
Ozaki, Y., Edelstein, M.P., Duch, D.S., 1988. Induction of indoleamine 2,3-dioxygenase: a mechanism of the antitumor activity of interferon gamma. Proc. Natl. Acad. Sci. USA 85, 1242–1246.
Poch, O., Sauvaget, I., Delarue, M., Tordo, N., 1989. Identification of four conserved motifs among the RNA-dependent polymerase encoding elements. EMBO J. 8, 3867–3874.
Rabbani, M.A., Ribaudo, M., Guo, J.T., Barik, S., 2016. Identification of interferon-stimulated gene proteins that inhibit human parainfluenza virus type 3. J. Virol. 90, 11145–11156.
Sage, L.K., Fox, J.M., Mellor, A.L., Tompkins, S.M., Tripp, R.A., 2014. Indoleamine 2,3-dioxygenase (IDO) activity during the primary immune response to influenza infection modifies the memory T cell response to influenza challenge. Viral Immunol. 27, 112–123.
Schechter, M.D., 1991. Effect of serotonin depletion by p-chlorophenylalanine upon discriminative behaviours. Gen. Pharm. 22, 889–893.
Schmidt, S.V., Schultze, J.L., 2014. New insights into IDO biology in bacterial and viral infections. Front. Immunol. 5, 384.
Schoggins, J.W., Rice, C.M., 2011. Interferon-stimulated genes and their antiviral effector functions. Curr. Opin. Virol. 1, 519–525.
Schwarcz, R., Bruno, J.P., Muchowski, P.J., Wu, H.Q., 2012. Kynurenines in the mammalian brain: when physiology meets pathology. Nat. Rev. Neurosci. 13, 465–477.
Svoboda, P., 2007. Off-targeting and other non-specific effects of RNAi experiments in mammalian cells. Curr. Opin. Mol. Ther. 9, 248–257.
Turner, E.H., Loftis, J.M., Blackwell, A.D., 2006. Serotonin a la carte: supplementation with the serotonin precursor 5-hydroxytryptophan. Pharm. Ther. 109, 325–338.
Uyttenhove, C., Pilotte, L., Théate, I., Stroobant, V., Colau, D., Parmentier, N., Boon, T., Van, den Eynde, B.J., 2003. Evidence for a tumoral immune resistance mechanism based on tryptophan degradation by indoleamine 2,3-dioxygenase. Nat. Med. 9, 1269–1274.
Watts, S.W., 2016. Oh, the places you'll go! My many colored serotonin (apologies to Dr. Seuss). Am. J. Physiol. Heart Circ. Physiol. 311, H1225–H1233.
Watzlawik, J.O., Wootla, B., Rodriguez, M., 2016. Tryptophan catabolites and their impact on multiple sclerosis progression. Curr. Pharm. Des. 22, 1049–1059.
