# Supplementary material for: 5-Hydroxytryptophan (5-HTP): Natural Occurrence, Analysis, Biosynthesis, Biotechnology, Physiology and Toxicology
Source: Int J Mol Sci. 2020 Dec 26;22(1):181. doi: 10.3390/ijms22010181 (PMC7796270; doi:10.3390/ijms22010181)
Supplement: Supplementary file 1 [file ijms-22-00181-s001.zip › 5-HTP.Data/PDF/3760412536/Sun-2015-Precursor-directed-biosynthesis-of-.pdf]

ACS Synthetic Biology
Letter
pubs.acs.org/synthbio

Precursor-Directed Biosynthesis of 5-Hydroxytryptophan Using Metabolically Engineered *E. coli*

Xinxiao Sun,[†] Yuheng Lin,[‡] Qipeng Yuan,*[,†] and Yajun Yan*[,§]

[†]State Key Laboratory of Chemical Resource Engineering, Beijing University of Chemical Technology, Beijing 100029, China
[‡]College of Engineering, University of Georgia, Athens, Georgia 30602, United States
[§]BioChemical Engineering Program, College of Engineering, University of Georgia, Athens, Georgia 30602, United States

Supporting Information

ABSTRACT: A novel biosynthetic pathway was designed and verified reversely leading to the production of 5-hydroxytryptophan (5-HTP) from glucose. This pathway takes advantage of the relaxed substrate selectivities of relevant enzymes without employing the unstable tryptophan 5-hydroxylase. First, high-titer of 5-HTP was produced from 5-hydroxyanthranilate (5-HI) by the catalysis of *E. coli* TrpDCBA. Then, a novel salicylate 5-hydroxylase was used to convert the non-natural substrate anthranilate to 5-HI. After that, the production of 5-HI from glucose was achieved and optimized with modular optimization. In the end, we combined the full pathway and adopted a two-stage strategy to realize the *de novo* production of 5-HTP. This work demonstrated the application of enzyme promiscuity in non-natural pathway design.

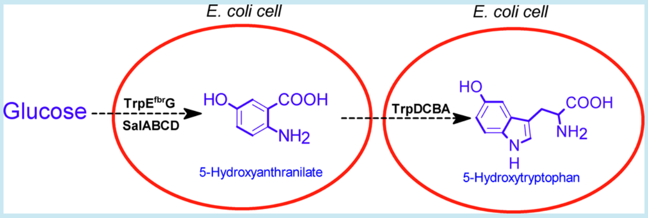

KEYWORDS: *5-hydroxytryptophan, 5-hydroxyanthranilate, salicylate 5-hydroxylase, precursor-directed biosynthesis*

5-Hydroxytryptophan (5-HTP) is a natural nonproteinogenic amino acid and serves as a direct biosynthetic precursor to the neurotransmitter serotonin. 5-HTP has been shown to be effective for the treatment of a variety of conditions, including depression, insomnia, chronic headaches, and binge eating associated with obesity. The therapeutic efficacy of 5-HTP is due to its ability to enhance the synthesis of serotonin in the brain. 5-HTP is well absorbed from an oral dose and can easily cross the blood−brain barrier.[1]

Currently, extraction from the seeds of African plant *Griffonia simplicifolia* is the approach for 5-HTP commercial production. However, the material supply is seasonally and regionally dependent, which limits the output of 5-HTP. Although chemical synthesis of 5-HTP has been reported,[2] it is not economically feasible in large scale. Biosynthesis provides a promising alternative to 5-HTP production. Microorganisms have simpler genetic backgrounds and metabolic networks. They grow faster and the biomass can reach high levels in simple synthetic medium. Many important chemicals have been successfully produced using genetically engineered microorganisms.[3−11]

So far, the only reported 5-HTP biosynthetic pathway is via tryptophan, and the reaction is catalyzed by tryptophan 5-hydroxylase (T5H). However, T5H is unstable when expressed in microorganisms and requires a special cofactor 5,6,7,8-tetrahydrobiopterin (BH4) that should be regenerated by additional enzyme reactions.[12] These problems hampered the application of this pathway to the economical production of 5-HTP. To circumvent these problems, in this study we established a novel artificial pathway for 5-HTP biosynthesis. This pathway takes the advantage of the substrate tolerance of the pathway enzymes and implements the concept of precursor-directed biosynthesis (Figure 1).

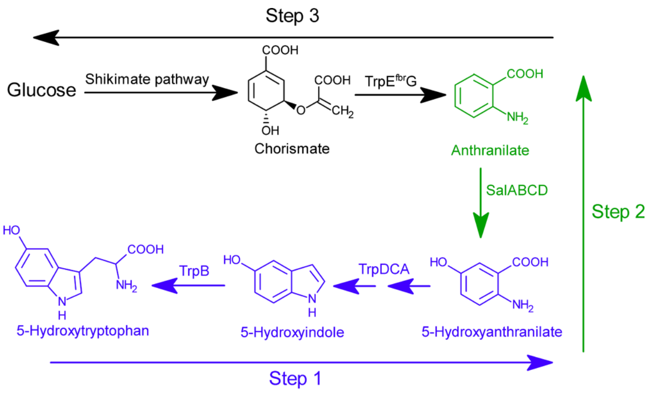

Figure 1. 5-HTP biosynthetic pathway designed in this study. TrpE^{fbr}G, anthranilate synthase (feedback resistance mutant); SalABCD, salicylate 5-hydroxylase; TrpDCA and TrpB, *E. coli* native tryptophan biosynthetic enzymes.

The first step of the pathway design is to find a simple precursor to 5-HTP. In *E. coli* tryptophan biosynthetic pathway, anthranilate (AA) is a precursor of tryptophan, and it is converted into tryptophan via five enzymatic reactions, which are catalyzed by TrpDCBA. 5-HTP is an analogue of tryptophan, and its corresponding precursor should be 5-

Received: August 21, 2014
Published: October 2, 2014

Downloaded via UNIV DEGLI STUDI DI TORINO on November 16, 2020 at 17:07:23 (UTC).
See https://pubs.acs.org/sharingguidelines for options on how to legitimately share published articles.

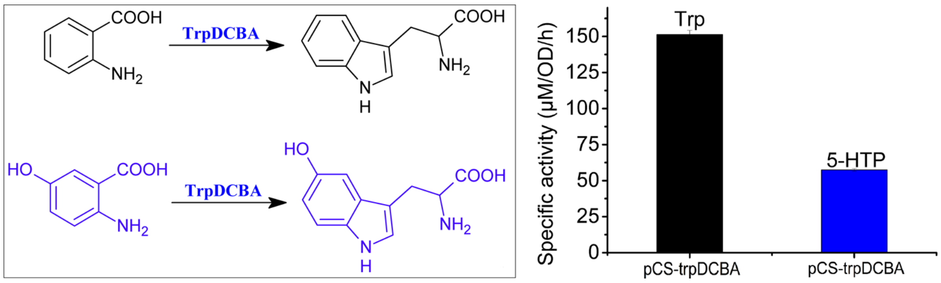

Figure 2. *In vivo* assays of TrpDCBA toward the natural substrate AA (black) and the non-natural substrate 5-HAA (blue). Strain BW2 harboring plasmid pCS-trpDCBA was used for the *in vivo* assays. The product of each reaction was shown on the top of each bar. Trp: tryptophan.

hydroxyanthranilate (5-HAA). It has been reported that substituted AA can also be converted into the corresponding substituted tryptophan through the pathway.[13] To investigate the reaction details, a medium-copy-number plasmid pCS-trpDCBA was constructed. *E. coli* strain BW2 with the knockouts of *tnaA* and *trpE* harboring pCS-trpDCBA was used for the *in vivo* assay. The knockout of *tnaA* can prevent the products tryptophan and 5-HTP from degradation while the knockout of *trpE* can block the native synthesis of AA.

The results showed that AA and 5-HAA can both go through these pathway enzymes, producing tryptophan and 5-HTP, respectively. The *in vivo* specific activity of the bioconversion system toward 5-HAA (57.44 ± 0.94 μM/OD/h) is about 40% of that toward AA (151.37 ± 2.93 μM/OD/h), which indicated that the 5-hydroxyl group affected the enzyme activities to some extent (Figure 2). We also cloned *trpDCBA* into a low-copy-number plasmid yielding pSA-trpDCBA. Interestingly, when strain BW2 harboring pSA-trpDCBA was used for the *in vivo* assays, the intermediate 5-hydroxyindole (5-HI) was accumulated in the cultures, indicating that the reaction catalyzed by TrpB was a rate-limiting step (Figure 3A). To test the capacity of this partial pathway, we continuously fed 5-HAA to the cultures of BW2 harboring pCS-trpDCBA. In 20 h, 1102.43 ± 24.95 mg/L of 5-HTP was produced (Figure 3B).

The second step of 5-HTP biosynthesis is to synthesize 5-HAA from a primary metabolite. AA is a potential precursor of 5-HAA since the 5-hydroxylation of AA will lead to the formation of 5-HAA. However, there were no AA 5-hydroxylases reported so far. Notably, two salicylate 5-hydroxylases (S5Hs) had been characterized from *Ralstonia sp.* Strain U2 and *Pseudomonas aeruginosa* Strain JB2.[14,15] S5H hydroxylates salicylate into gentisate (GA) and is involved in the degradation of aromatic compounds. Since salicylate (2-hydroxybenzoate) and AA (2-aminobenzoate) have very similar molecular structures, we hypothesized that S5H may also hydroxylate AA. To find new S5H candidates, we searched the protein database using the basic local alignment search tool (BLAST) according to the amino acid sequences of the two characterized S5Hs. A gene cluster from *Ralstonia eutropha* H16 encoding a putative S5H was found and designated as *salABCD*. Unlike the former two S5Hs, this cluster locates on the chromosome rather than on a mobile plasmid. While SalABC showed high protein sequence similarity with those from *Ralstonia sp.* Strain U2 (57%−75%), SalD showed only 13% similarity with that from *Ralstonia sp.* Strain U2.

To characterize this gene cluster and test the necessity of SalD, we constructed two high-copy-number plasmids pZE-salABCD and pZE-salABC. *E. coli* strain BW3 with the knockouts of *tnaA* and *trpD* was transformed with these two plasmids, separately. The knockout of *trpD* can block the consumption of AA and 5-HAA by the native TrpDCBA.

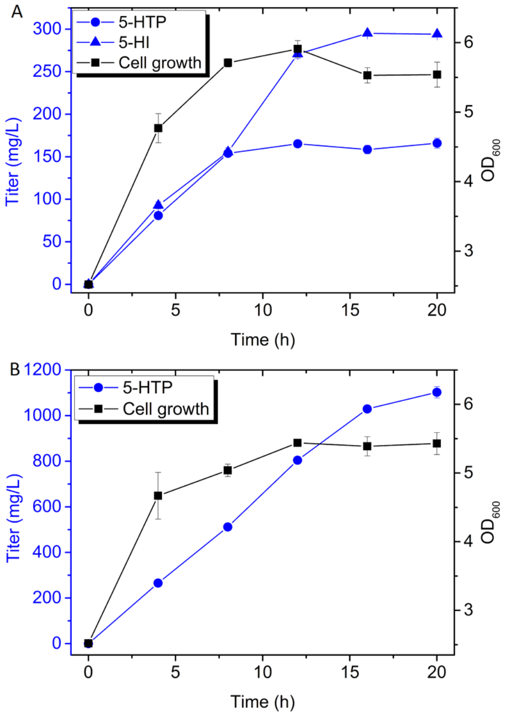

Figure 3. Bioconversion of 5-HAA to 5-HTP. A: Strain BW2 harboring low-copy-number plasmid pSA-trpDCBA. B: Strain BW2 harboring medium-copy-number plasmid pCS-trpDCBA. 5-HAA (600 mg/L) was fed to the cultures at 0 and 10 h. Samples were taken every 4 h and analyzed by HPLC.

The *in vivo* assay showed that SalABCD can convert salicylate into GA with a specific activity of 268.72 ± 7.79 μM/OD/h. We then further tested the enzyme activity toward AA. As expected, the enzyme system could also convert AA into 5-HAA, although with a slightly lower specific activity (216.06 ± 2.02 μM/OD/h). However, the specific activity of SalABC toward AA decreased significantly to 16.75 ± 2.06 μM/OD/h, which proved that SalD is an essential component of the S5H (Figure 4). It is worth mentioning here that SalABCD requires NAD(P)H as the cofactor instead of BH4 and that the former ones are abundant compounds in *E. coli*.

We also tested another enzyme combination to hydroxylate AA, which consisted of an anthraniloyl-CoA synthetase (PqsA) and a salicyloyl-CoA 5-hydroxylase (SdgC). PqsA is involved in the biosynthesis of 2,4-dihydroxyquinoline, an extracellular metabolite produced by *Pseudomonas aeruginosa*.[16] SdgC takes part in salicylate degradation by *Streptomyces* sp. strain WA46.[17]

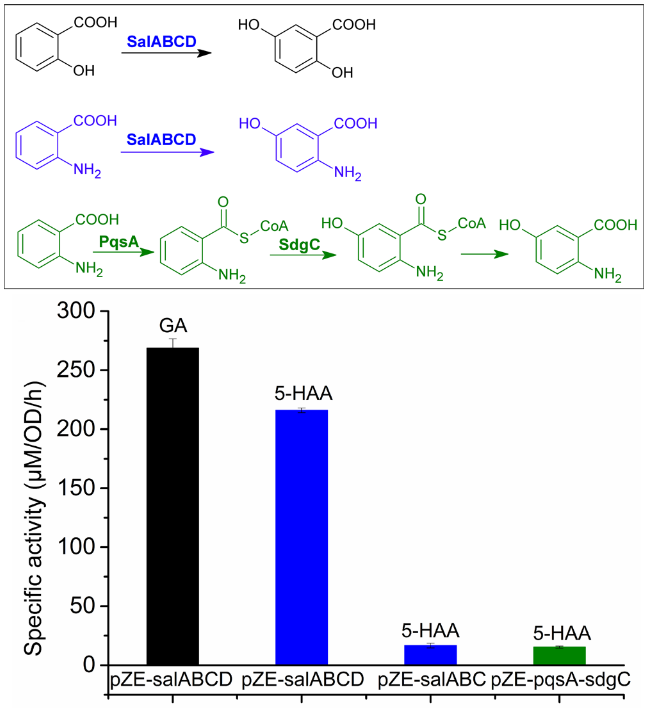

Figure 4. *In vivo* assays of anthranilate 5-hydroxylases. Strain BW3 was used as the host. Plasmids used were shown below each bar. The product of each reaction was shown on the top of each bar.

The *in vivo* assay showed that AA could be converted into 5-HAA by the sequential catalyzation of these two enzymes. However, compared with that of SalABCD, the specific activity of this combination is not desirable (15.44 ± 0.92 μM/OD/h) (Figure 4). We then continuously fed AA to the cultures of strain BW3 harboring plasmid pZE-salABCD. In 12 h, 442.51 ± 6.25 mg/L 5-HAA was produced (Figure S1, Supporting Information).

The last step to establish the 5-HTP biosynthesis is to connect the non-native downstream partial pathway with the *E. coli* native metabolism. As the production of AA in *E. coli* had been reported in our prevous work,[18] here we optimized the production of 5-HAA from glucose by combining the AA production and conversion partial pathways. The 5-HAA biosynthetic pathway was divided into three modules (Figure 1). Module 1 consists of salABCD, which catalyzes the formation of 5-HAA from AA; module 2 is the trpE^{fbr}G module, which converts chorismate to AA; and module 3 is the APTA module, which expresses four key enzymes (AroL, PpsA, TktA, and AroG^{fbr}) in the shikimate pathway to boost the carbon flux toward chorismate synthesis.

Modular optimization results showed that the highest titer (224.31 ± 8.89 mg/L) of 5-HAA was obtained when module 1 was in high-copy-number plasmid (pZE-salABCD) and module 2 in low-copy-number plasmid (pSA-trpE^{fbr}G) (Figure 5). The introduction of module 3 did not further improve the titer. In addition, the intermediate AA was accumulated in all cases, which indicated that module 1 was still a rate-limiting step, even expressed by the high-copy-number plasmid.

After achieving the production of 5-HAA from glucose, we tried to combine the full pathway to realize the *de novo* production of 5-HTP. *E. coli* strain BW1 with the knockout of *tnaA* was transformed with plasmids pSA-trpE^{fbr}G, pZE-salABCD, and pCS-trpDCBA. The positive transformants

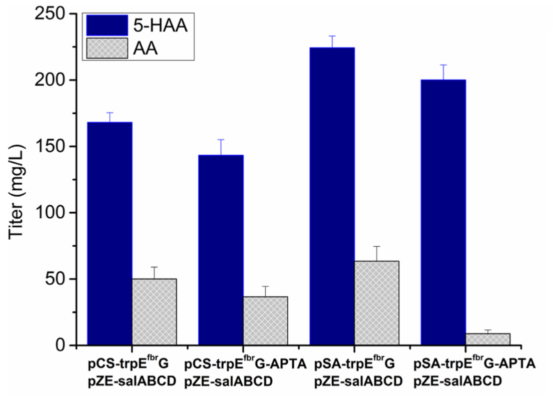

Figure 5. Modular optimization of 5-HAA production from glucose. Strain BW3 was used as the host. Plasmids used were shown below each bar.

were used for shake flask experiments. To our surprise, after 24 h of cultivation no 5-HTP was accumulated in the cultures; while tryptophan was produced at 205.14 ± 6.71 mg/L. One explanation for this phenomenon is that TrpDCBA showed higher substrate affinity and specific activity toward AA than 5-HAA and that AA was directly converted to tryptophan without being hydroxylated. Another possible reason is that TrpE and TrpD have been evolved to form heterotetrameric complex to decrease substrate diffusion.[19] The formation of this enzyme complex may prevent SalABCD from getting access to its substrate AA.

To solve this problem, we attempted to produce 5-HTP by coculturing strain BW3 harboring pSA-trpE^{fbr}G and pZE-salABCD and strain BW2 harboring pSA-trpDCBA and pCA-trpDCBA with two different inoculation ratios. Plasmid pCA-trpDCBA was constructed by replacing the kanamycin resistance gene of plasmid pCS-trpDCBA with ampicillin resistance gene. However, the results were still disappointing, and only about 1 mg/L 5-HTP was produced when the innoculation ratio of the two strains was 1:1; while 5-HTP was not detected with the innoculation ratio of 2:1.

We then developed a two-step strategy for 5-HTP production. First, *E. coli* strain BW3 harboring pSA-trpE^{fbr}G and pZE-salABCD was used for 5-HAA production from glucose. After 24 h, the cells were removed by centrifugation, and the supernatants were mixed with equal volume of the cultures of *E. coli* strain BW2 harboring pSA-trpDCBA and pCA-trpDCBA. The produced 5-HAA was further converted into 5-HTP, and the final titers can reach 98.09 ± 3.24 mg/L (Figure 6). In addition, 23.05 ± 0.83 mg/L tryptophan was also produced as the byproduct, and the accumulation of the intermediate 5-HI was not observed.

In this study, we developed a novel biosynthetic pathway and achieved the production of 5-HTP from glucose using a two-stage system. Currently, the titer is relatively low. To increase its feasibility for industrial application, this pathway needs to be further optimized by systems metabolic engineering strategies.[20] First, the activity of the key enzyme S5H should be improved by either enzyme mining or enzyme evolution. Second, to achieve the production of 5-HTP from the cells expressing the full artificial pathway, we plan to alter the substrate specificity of TrpD by rational protein engineering approaches as well as colocalize TrpE^{fbr}G and S5H by synthetic protein scaffolds to increase the accessibility of AA to S5H.

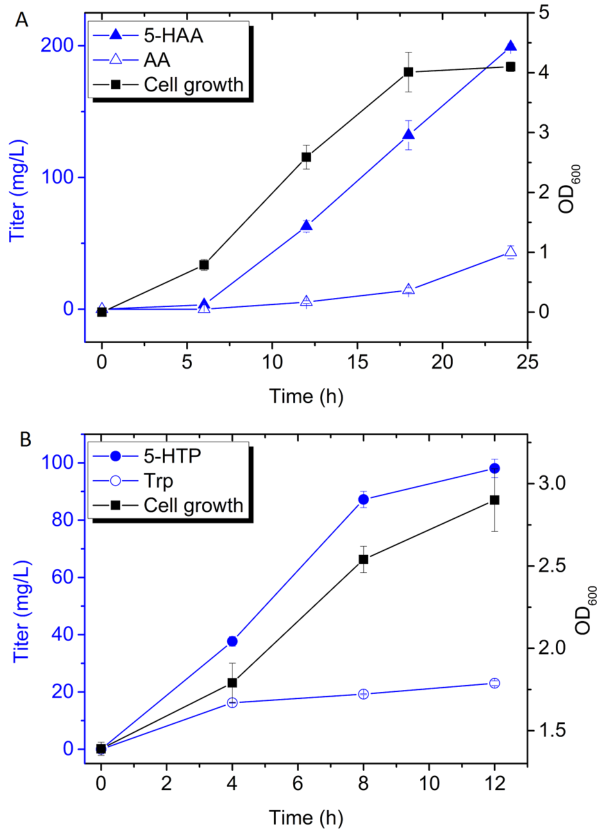

Figure 6. Two-step strategy for 5-HTP production. A: 5-HAA production from glucose using strain BW3 harboring plasmids pZE-salABCD and pSA-trpE^{fbr}G. B: 5-HTP production from 5-HAA using strain BW2 harboring plasmids pCA-trpDCBA and pSA-trpDCBA.

■ METHODS

Strains, Plasmids and Medium. Strains and plasmids used in this study are summarized in Tables S1 and S2, Supporting Information, respectively. *E. coli* strain XL1-Blue was used as the host for standard cloning and plasmid propagation. *E. coli* strains BW1, BW2, and BW3 were used for *in vivo* assays and the production of 5-HAA and 5-HTP. Luria−Bertani (LB) medium was used for inoculant preparation and cell propagation. Modified M9 medium was used for microbial synthesis of 5-HAA and 5-HTP. LB medium contains 10 g/L tryptone, 5 g/L yeast extract, and 10 g/L NaCl. The modified M9 minimal medium contains 10 g/L glucose, 6 g/L Na_2HPO_4, 0.5 g/L NaCl, 3 g/L KH_2PO_4, 1 g/L NH_4Cl, 1 mM $MgSO_4$, 0.1 mM $CaCl_2$, 2 g/L yeast extract,2 g/L sodium citrate, and 100 mg/L serine. When needed, ampicillin, kanamycin, and chloramphenicol were added to the medium at 100, 50, and 34 μg/mL, respectively.

***In Vivo* Enzyme Assays.** *In vivo* assays were carried out to evaluate the activities of TrpDCBA and anthranilate 5-hydroxylases toward their substrates. *E. coli* BW25113 derivative strains were transformed with the corresponding plasmids. Fresh colonies were inoculated into 3 mL of LB medium containing appropriate antibiotics and grown aerobically at 37 °C. Overnight cultures were inoculated into 20 mL of LB medium and left to grow at 37 °C. When OD_{600} reached 0.4, the cultures were induced with 0.25 mM IPTG at 30 °C for 3 h. Cells were then harvested by centrifugation and resuspended in the modified M9 medium. The cell suspensions were fed with the corresponding substrates and incubated at 30 °C with shaking. Samples were taken after 1 h, and the product concentrations were detected by HPLC. The specific activity was expressed as μM/OD/h. For the bioconversion experiments, substrates were added to the cell suspensions at several time points. Samples were taken every few hours and used for the analysis of the cell growth and product accumulation.

Microbial Production of 5-HAA. *E. coli* strain BW3 was transformed with the corresponding plasmids. Overnight cultures were inoculated into the modified M9 medium containing appropriate antibiotics and cultivated at 30 °C with shaking. When OD_{600} reached 0.4, the cultures were induced with 0.25 mM IPTG and continued to grow at 30 °C. Samples were taken every few hours. OD_{600} values were measured, and the product 5-HAA and the intermediates were analyzed by HPLC.

Microbial Production of 5-HTP. We tried two different strategies for the production of 5-HTP from glucose. In the first strategy, *E. coli* strain BW1 was transformed with plasmids pZE-salABCD, pSA-trpE^{fbr}G, and pCS-trpDCBA. Overnight cultures were inoculated into 20 mL of modified M9 medium containing ampicillin, kanamycin, and chloramphenicol and left to grow at 30 °C. When OD_{600} reached 0.4, the cultures were induced with 0.25 mM IPTG. Samples were taken, and the product and the intermediates were analyzed by HPLC. The second strategy was a two-stage process. First, *E. coli* strain BW3 was transformed with plasmids pZE-salABCD and pSA-trpE^{fbr}G. Overnight cultures were inoculated into 20 mL of modified M9 medium containing ampicillin and chloramphenicol and left to grow at 30 °C. When OD_{600} reached 0.4, the cultures were induced with 0.25 mM IPTG. After 24 h of cultivation, the cultures were harvested by centrifugation, and the supernatant was mixed with equal volume of the cultures of strain BW2 harboring pCA-trpDCBA and pSA-trpDCBA, which had already been induced with 0.25 mM IPTG for 3 h. The new cultures continued to be cultivated at 30 °C with shaking. Samples were taken every 4 h, and the substrate consumption and the product and intermediate accumulation were dectected by HPLC.

HPLC Analysis. Salicylate, GA, AA, 5-HAA, tryptophan, 5-HTP, and 5-HI were purchased from Sigma-Aldrich or Acros Organics and were used as the standards. Both the standards and samples were analyzed and quantified by HPLC (Dionex Ultimate 3000) equipped with a reverse phase ZORBAX SB-C18 column and an Ultimate 3000 Photodiode Array Detector. Solvent A was water with 0.2% trifluoroacetic acid, and solvent B was methanol. The column temperature was set to 28 °C. The following gradient was used at a flow rate of 1 mL/min: 5 to 50% solvent B for 15 min, 50 to 5% solvent B for 1 min, and 5% solvent B for an additional 4 min. Quantification of each compound was based on the peak areas at absorbance of specific wavelengths (salicylate 303 nm, GA 331 nm, AA 330 nm, 5-HAA 297 nm, tryptophan 275 nm, 5-HTP 276 nm, and 5-HI 271 nm).

■ ASSOCIATED CONTENT

Ⓢ Supporting Information

This material is available free of charge via the Internet at http://pubs.acs.org.

■ AUTHOR INFORMATION

Corresponding Authors

*(Q.Y.) Tel: +86-13601162675. E-mail: yuanqp@mail.buct.edu.cn.

*(Y.Y.) Tel: 001-706-542-8293. Fax: 001-706-542-8806. E-mail: yajunyan@uga.edu.

Author Contributions

X.S. and Y.Y. conceived this study. X.S. and Y.L. designed and performed the experiments. Y.Y. and Q.Y. supervised and directed the project. X.S. analyzed the data and wrote the manuscript. Y.Y. revised the manuscript.

Notes

The authors declare the following competing financial interest(s): A patent application on this technology has been filed by the University of Georgia..

■ ACKNOWLEDGMENTS

This work was supported by the start-up funds from the College of Engineering, The University of Georgia, Athens.

■ REFERENCES

(1) Birdsall, T. C. (1998) 5-Hydroxytryptophan: a clinically-effective serotonin precursor. *Altern. Med. Rev. 3*, 271−280.

(2) Frangatos, G., and Chubb, F. L. (1959) A new synthesis of 5-hydroxytryptophan. *Can. J. Chem. 37*, 1374−1376.

(3) Lin, Y., Shen, X., Yuan, Q., and Yan, Y. (2013) Microbial biosynthesis of the anticoagulant precursor 4-hydroxycoumarin. *Nat. Commun. 4*, 2603.

(4) Zhang, K., Sawaya, M. R., Eisenberg, D. S., and Liao, J. C. (2008) Expanding metabolism for biosynthesis of nonnatural alcohols. *Proc. Natl. Acad. Sci. U.S.A. 105*, 20653−20658.

(5) Xu, P., Gu, Q., Wang, W., Wong, L., Bower, A. G., Collins, C. H., and Koffas, M. A. (2013) Modular optimization of multi-gene pathways for fatty acids production in *E. coli*. *Nat. Commun. 4*, 1409.

(6) Lin, Y., Sun, X., Yuan, Q., and Yan, Y. (2013) Combinatorial biosynthesis of plant-specific coumarins in bacteria. *Metab. Eng. 18*, 69−77.

(7) Ajikumar, P. K., Xiao, W. H., Tyo, K. E., Wang, Y., Simeon, F., Leonard, E., Mucha, O., Phon, T. H., Pfeifer, B., and Stephanopoulos, G. (2010) Isoprenoid pathway optimization for Taxol precursor overproduction in *Escherichia coli*. *Science 330*, 70−74.

(8) Atsumi, S., Hanai, T., and Liao, J. C. (2008) Non-fermentative pathways for synthesis of branched-chain higher alcohols as biofuels. *Nature 451*, 86−89.

(9) Lin, Y., Sun, X., Yuan, Q., and Yan, Y. (2014) Extending shikimate pathway for the production of muconic acid and its precursor salicylic acid in *Escherichia coli*. *Metab. Eng. 23*, 62−69.

(10) Lin, Y., and Yan, Y. (2014) Biotechnological production of plant-specific hydroxylated phenylpropanoids. *Biotechnol. Bioeng. 111*, 1895−1899.

(11) Yim, H., Haselbeck, R., Niu, W., Pujol-Baxley, C., Burgard, A., Boldt, J., Khandurina, J., Trawick, J. D., Osterhout, R. E., Stephen, R., Estadilla, J., Teisan, S., Schreyer, H. B., Andrae, S., Yang, T. H., Lee, S. Y., Burk, M. J., and Van Dien, S. (2011) Metabolic engineering of *Escherichia coli* for direct production of 1,4-butanediol. *Nat. Chem. Biol. 7*, 445−452.

(12) Hamdan, F. F., and Ribeiro, P. (1999) Characterization of a stable form of tryptophan hydroxylase from the human parasite *Schistosoma mansoni*. *J. Biol. Chem. 274*, 21746−21754.

(13) Chase, M. W., Power, S. D., and Whited, G. M. (1995) Microbial production of 4-, 5-, 6- and 7-substituted indole and tryptophan analogs via fermentation, Google Patents, WO1995034657 A2.

(14) Zhou, N. Y., Al-Dulayymi, J., Baird, M. S., and Williams, P. A. (2002) Salicylate 5-hydroxylase from *Ralstonia* sp. strain U2: a monooxygenase with close relationships to and shared electron transport proteins with naphthalene dioxygenase. *J. Bacteriol. 184*, 1547−1555.

(15) Hickey, W. J., Sabat, G., Yuroff, A. S., Arment, A. R., and Perez-Lesher, J. (2001) Cloning, nucleotide sequencing, and functional analysis of a novel, mobile cluster of biodegradation genes from *Pseudomonas aeruginosa* strain JB2. *Appl. Environ. Microbiol. 67*, 4603−4609.

(16) Zhang, Y. M., Frank, M. W., Zhu, K., Mayasundari, A., and Rock, C. O. (2008) PqsD is responsible for the synthesis of 2,4-dihydroxyquinoline, an extracellular metabolite produced by *Pseudomonas aeruginosa*. *J. Biol. Chem. 283*, 28788−28794.

(17) Ishiyama, D., Vujaklija, D., and Davies, J. (2004) Novel pathway of salicylate degradation by *Streptomyces* sp. strain WA46. *Appl. Environ. Microbiol. 70*, 1297−1306.

(18) Sun, X., Lin, Y., Huang, Q., Yuan, Q., and Yan, Y. (2013) A novel muconic acid biosynthesis approach by shunting tryptophan biosynthesis via anthranilate. *Appl. Environ. Microbiol. 79*, 4024−4030.

(19) Balderas-Hernandez, V. E., Sabido-Ramos, A., Silva, P., Cabrera-Valladares, N., Hernandez-Chavez, G., Baez-Viveros, J. L., Martinez, A., Bolivar, F., and Gosset, G. (2009) Metabolic engineering for improving anthranilate synthesis from glucose in *Escherichia coli*. *Microb. Cell Fact. 8*, 19.

(20) Lee, J. W., Na, D., Park, J. M., Lee, J., Choi, S., and Lee, S. Y. (2012) Systems metabolic engineering of microorganisms for natural and non-natural chemicals. *Nat. Chem. Biol. 8*, 536−546.
