# Supplementary material for: 5-Hydroxytryptophan (5-HTP): Natural Occurrence, Analysis, Biosynthesis, Biotechnology, Physiology and Toxicology
Source: Int J Mol Sci. 2020 Dec 26;22(1):181. doi: 10.3390/ijms22010181 (PMC7796270; doi:10.3390/ijms22010181)
Supplement: Supplementary file 1 [file ijms-22-00181-s001.zip › 5-HTP.Data/PDF/3940976703/Yamamoto-2003-Genetic-engineering-of-escherich.pdf]

Available online at www.sciencedirect.com
SCIENCE DIRECT
ACADEMIC PRESS
Metabolic Engineering 5 (2003) 246–254
METABOLIC ENGINEERING
http://www.elsevier.com/locate/ymben

Genetic engineering of *Escherichia coli* for production of tetrahydrobiopterin

Katsuhiko Yamamoto,* Eri Kataoka, Nobue Miyamoto, Kazuaki Furukawa, Kazuhiro Ohsuye, and Masayuki Yabuta

Bioprocess Development Group, Institute for Medicinal Research and Development, Daiichi Suntory Pharma Co., Ltd. 2716-1 Kurakake, Akaiwa, Chiyoda-machi, Ohra-gun, Gunma 370-0503, Japan

Received 7 October 2002; accepted 7 July 2003

Abstract

Tetrahydrobiopterin (BH4) is an essential cofactor for various enzymes in mammals. In vivo, it is synthesized from GTP via the three-step pathway of GTP cyclohydrolase I (GCHI), 6-pyruvoyl-tetrahydropterin synthase (PTPS) and sepiapterin reductase (SPR). BH4 is a medicine used to treat atypical hyperphenylalaninemia. It is currently synthesized by chemical means, which consists of many steps, and requires costly materials and complicated procedures. To explore an alternative microbial method for BH4 production, we utilized recombinant DNA technology to construct recombinant *Escherichia coli* (*E. coli*) strains carrying genes expressing GCHI, PTPS and SPR enzymes. These strains successfully produced BH4, which was detected as dihydrobiopterin and biopterin, oxidation products of BH4. In order to increase BH4 productivity we made further improvements. First, to increase the de novo GTP supply, an 8-azaguanine resistant mutant was isolated and an additional *guaBA* operon was introduced. Second, to augment the activity of GCHI, the *folE* gene from *E. coli* was replaced by the *mtrA* gene from *Bacillus subtilis*. These modifications provided us with a strain showing significantly higher productivity, up to 4.0 g of biopterin/L of culture broth. The results suggest the possibility of commercial BH4 production by our method.
© 2003 Elsevier Inc. All rights reserved.

Keywords: Tetrahydrobiopterin; Production; Metabolic engineering; *Escherichia coli*

1. Introduction

Tetrahydrobiopterin (BH4) is known as an essential cofactor in higher organisms used by various enzymes, e.g., phenylalanine hydroxylase, tyrosine hydroxylase, tryptophan hydroxylase, and three isoforms of NO synthase. The cofactor has great importance in human physiology (reviewed by Thöny et al., 2000; Werner-Felmayer et al., 2002). Recent studies suggest that several neurological diseases, including Dopa-responsive dystonia and Parkinson's disease, are the consequence of limited BH4 availability (Nakamura et al., 2001). BH4 is used as a medicine (generic name, sapropterin hydrochloride) for the treatment of patients with atypical hyperphenylalaninemia which is caused by the inherited deficiency of the integral BH4 biosynthetic pathway.

Since 1992 native BH4 for medical use has been chemically synthesized. Having three asymmetric carbons, the chemical synthesis of BH4 is difficult. It requires expensive materials, uses many synthetic steps, and requires complicated handling procedures. This makes it a good target for the development of alternate production methods.

All three genes for the enzymes involved in the biosynthetic pathway of GTP to BH4 in mammals have been cloned; these are GTP cyclohydrolase I (GCHI), 6-pyruvoyl-tetrahydropterin synthase (PTPS), and sepiapterin reductase (SPR) (see Fig. 1). Reaction mechanisms of these biosynthetic enzymes for de novo BH4 production have been investigated and their mode of action proposed in many reports (Takikawa et al., 1986; Milstien and Kaufman, 1989; Thöny et al., 2000). Therefore, alternative production methods of BH4 can now be considered. Hatakeyama et al. (1993) succeeded in synthesizing BH4 with purified GCHI, PTPS and SPR in vitro in a single reactor containing GTP and NADPH. The method, however, has not been applied to

*Corresponding author.
E-mail address: katsuhiko_yamamoto@dsup.co.jp (K. Yamamoto).

1096-7176/$ - see front matter © 2003 Elsevier Inc. All rights reserved.
doi:10.1016/S1096-7176(03)00046-6

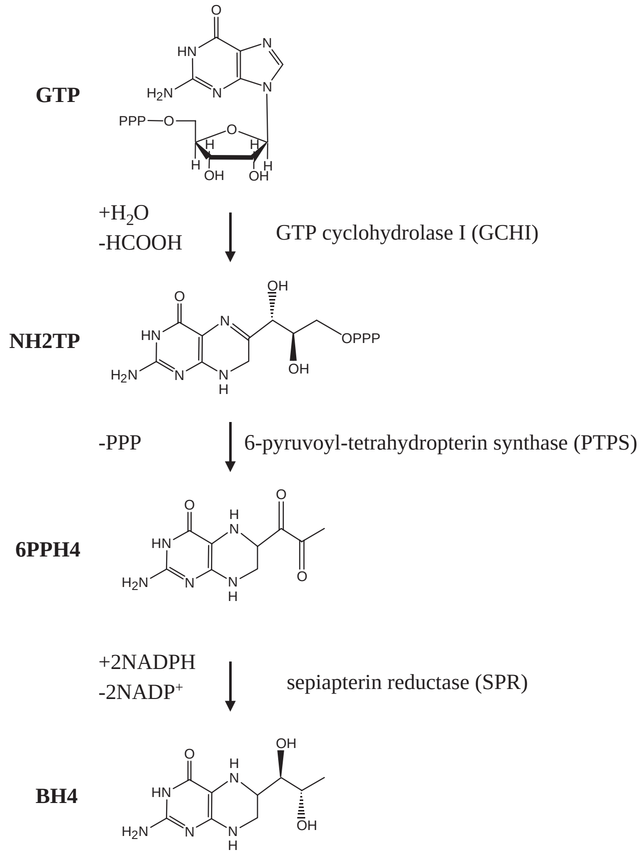

Fig. 1. The de novo BH4 biosynthetic pathway. Mammals synthesize BH4 from GTP de novo in a three-step reaction through the actions of GCHI, PTPS, and SPR. Abbreviations used: GTP, guanosine 5′-triphosphate; NH2TP, 7,8-dihydroneopterin triphosphate; 6PPH4, 6-pyruvoyl tetrahydropterin; PPP, triphosphate; BH4, 5,6,7,8-tetrahydrobiopterin.

production at an industrial scale, since costly materials, GTP and NADPH, are needed and sufficient preparations of the enzymes are not available.

We therefore set out to produce BH4 using microbial fermentation with strains developed utilizing recombinant gene technology. The present study reports that recombinant *E. coli* strains harboring recombinant BH4 biosynthetic genes were capable of high BH4 productivity. To accomplish this we used techniques including host cell mutagenesis, optimizing gene expression, directing carbon source to the BH4 synthetic pathway and high-density cultivation. The maximum BH4 productivity so far achieved reaches approximately 4.0 g/L of culture broth. Although further developments including purification processing and larger scale cultivation remain to be explored, the BH4 producing bacterial strains could be the basis of a commercially attractive production process and provide us with many advantages in cost, operational procedures and yield compared to the chemical synthesis method.

2. Materials and methods

2.1. Bacterial strains and isolation of purine analogue-resistant mutants

E. coli JM101 was used as the host strain for BH4 production. Isolation of purine analogue-resistant mutants was carried out as described (Miller, 1972). The cells were mutagenized with 50 μg/ml *N*-methyl-*N*′-nitro-*N*-nitrosoguanidine (NTG), streaked and screened on M9 minimum media agar plate containing 100 μg/ml 8-azaguanine or 500 μg/ml decoyinine.

E. coli JM109 was used for routine genetic manipulations. Genomic DNA from *E. coli* strain W3110 was used to obtain the *folE*, *guaBA*, and *gmk* genes. *Bacillus subtilis* strain 1A1, which was kindly provided by Prof. Y. Fujita (Fukuyama Univ., Japan), was used to obtain the *mtrA* gene. All cells were maintained as 15% (w/v) glycerol stocks at −80°C after growth in Luria-Bertani medium. The preparation and transformation of competent *E. coli* cells were performed as reported (Inoue et al., 1990).

2.2. Construction of expression plasmids

PCR amplification was used to generate the *folE* gene [GenBank X63910] from *E. coli* W3110 genomic DNA, the genes encoding PTPS [NM_017220] and SPR [M36410] from rat liver cDNA, and the *mtrA* [M37320] gene from *B. subtilis* 1A1 genomic DNA. *E. coli* and *B. subtilis* genomic DNA was extracted by standard methods (Silhavy et al., 1984; Lovett and Keggins, 1979).

Genomic DNA or cDNA was used as a template and the first PCR was carried out with a pair of primers, whose sequence was derived from the GenBank database. Amplified PCR fragments were used as the template in a second PCR reaction using a pair of primers containing restriction enzyme sites. The restriction enzyme sites used for each primer pair were *Eco*RI/*Sal*I (*folE* and PTPS), *Hind*III/*Hind*III (SPR) and *Eco*RI/*Xba*I (*mtrA*). The PCR fragments and expression vectors (Table 1) were digested by the appropriate restriction enzymes and ligated so as to construct the various expression plasmids. The vectors pUC18, pTWV228, pSTV28 and pMW218 were used for *folE*, PTPS and *mtrA*, and pUC19, pTWV229, pSTV29 and pMW219 for SPR. In these expression plasmids, the *E. coli lac* promoter transcribed each gene and the expressed protein had 7 or 8 amino acids derived from the vector at their amino terminus. These short amino acid sequences were the same in pSTV28GPS or pSTV28MPS.

Construction of the expression plasmid pSTV28GPS was carried out by the following procedures. A DNA fragment spanning from the *lac* promoter to the

Table 1
Expression plasmids used

Name	Marker gene	*ori* sequence from	Copy number
pUC18/19	Amp^r	pUC18	+ + + +
pTWV228/229	Amp^r	pBR322	+ + +
pSTV28/29	Cm^r	pACYC184	+ +
pMW218/219	Km^r	pSC101	+

All genes introduced were inserted into a common expression system consisting of a lac promoter/operator and a multicloning site that leads to a strong expression of an inserted gene, but using plasmids with different ori sequence which controls the copy number per cell. The marks (+, + +, + + +, + + + +) indicate relative copy number in *E. coli*. Abbreviations used: Amp^r, ampicillin resistant gene; Cm^r, chloramphenicol resistant gene; Km^r, kanamycin resistant gene.

termination codon of PTPS was obtained by PCR. The plasmid pUC18PTPS was used as template and a sense primer containing a *Sal*I site and an antisense primer containing a *Bam*HI site were used. PCR from pUC19SPR was carried out in the same way: a DNA fragment containing the *lac* promoter and SPR gene with a *Bam*HI site at the 5′ end and *Sph*I site at the 3′ end was obtained. The *Sal*I-*Bam*HI fragment containing PTPS and *Bam*HI-*Sph*I fragment containing SPR were inserted into a 3.9 kb DNA fragment obtained from pSTV28folE digested with *Xho*I and *Sph*I.

pSTV28MPS was constructed from three fragments; the *mtrA* fragment from pUC18 mtrA digested with *Eco*RI and *Xba*I, the 1.4 kb PTPS-SPR fragment from pSTV28GPS digested with *Xba*I and *Sph*I, and the 3.0 kb fragment from pSTV28GPS digested with *Eco*RI and *Sph*I. Each gene encoded an additional 7 or 8 amino acids derived from the original vector at the amino terminus.

The *guaBA* operon [M10101], and *gmk* [AE000442] were obtained from *E. coli* W3110 genomic DNA by PCR. Each PCR product, including the original promoter, was cloned into the vector pCR2.1 (Invitrogen, CA, USA) to prepare pCR2.1guaBA and pCR2.1gmk, respectively. These plasmids were digested with *Bam*HI and *Xho*I, and the *guaBA* or *gmk* fragment was inserted into pTWV228 or pMW218.

All DNA primers for PCR were purchased from Amersham Pharmacia Biotech (Uppsala, Sweden), expression plasmids pUC18/19 and pMW218/219 were from Nippon Gene (Tokyo, Japan) and pTWV228/229 and pSTV28/29 were from Takara Shuzo (Kyoto, Japan).

2.3. Tube culture conditions

For tube cultures, seed cultures were prepared by growing cells in 3 ml of Luria-Bertani medium at 37°C overnight. Pre-cultured BH4 producing cells were inoculated in NUCA medium, which consists of the following components (per liter): glycerol, 20 g; yeast extract, 4 g; KH_2PO_4, 4 g; K_2HPO_4, 4 g; Na_2HPO_4, 2.8 g; $(NH_4)_2SO_4$, 1.2 g; NH_4Cl, 0.2 g; $MgSO_4 \cdot 7H_2O$, 2 g; casein hydrolysate, 10 g; $FeSO_4 \cdot 7H_2O$, 40 mg; $CaCl_2 \cdot 2H_2O$, 40 mg; $MnSO_4 \cdot 5H_2O$, 10 mg; $AlCl_3 \cdot 6H_2O$, 10 mg; $CoCl_2 \cdot 6H_2O$, 4 mg; $ZnSO_4 \cdot 7H_2O$, 2 mg; $Na_2MoO_4 \cdot 2H_2O$, 2 mg; $CuCl_2 \cdot 2H_2O$, 1 mg; and H_3BO_3, 0.5 mg. Isopropyl-β-D-thiogalactopyranoside (IPTG) was added to a concentration of 0.5 mM. Antibiotics (chloramphenicol, ampicillin and kanamycin) were used as required by the plasmids at appropriate concentrations. All tube cultures were done in a shaking incubator for the time described at 37°C, 200 rpm. After 40 h of cultivation, cell growth, pH and BH4 productivity were measured. Cell growth was monitored by measuring the absorbance at 660 nm with a spectrophotometer (UV-120-01, Shimadzu, Kyoto, Japan).

2.4. Fermentation

During fermentation, the dissolved oxygen concentration and pH value were controlled automatically by a computerized system (Biott, Tokyo, Japan). Medium composition was optimized in a tube culture scale study for carbon source, pH and casein hydrolysate supplement (data not shown). BH4 producing strains were pre-cultured for 10 h followed by inoculation into the NUCA medium. Glycerol was used as the carbon source (2% w/v) in the initial medium, and after consumption, an 80% (w/v) glycerol solution was continuously added by pump feeding at 10–15 ml/h.

2.5. Detection of BH4

The BH4 productivity was expressed as the amount of biopterin (BP) in the culture medium after oxidation of the BH4 and 7,8-dihydrobiopterin (BH2) in the broth to BP (Smith and Nichol, 1986). For the conversion, a 20% volume of a potassium iodide solution (1 N hydrochloric acid solution containing 0.9% I_2 and 1.8% KI) was added to the culture broth, which was allowed to stand for 1 h shielded from the light. The incubated mixture was centrifuged at 12,000 rpm for 5 min. The supernatant was diluted 100-fold with phosphate buffer (10 mM sodium phosphate, 1 mM EDTA, pH 7.0). The treated samples were subjected to fluorometer and HPLC analyses.

To estimate the quantity of BP, a FluoroskanII (Flow Laboratories, Lugano, Switzerland) fluorometer was used with a 96-well microplate at 355 nm excitation and 460 nm emission wavelengths. Preliminary experiments showed that the intensity of fluorescence was positively correlated with BP area obtained by HPLC analyses.

HPLC analysis was performed to precisely measure BP amount. Samples were injected onto a C18 reversed-phase column (COSMOSIL 5C18-AR, Nacalai Tesque, Kyoto, Japan) of 4.6 mm × 250 mm equilibrated with phosphate buffer (10 mM sodium phosphate, 1 mM EDTA, pH 7.0) at a flow rate of 0.8 ml/min. Isocratic elution was done with equilibration buffer. The effluent was monitored by both absorbance at 214 nm and a fluorescent detector (excitation wavelength at 350 nm and emission wavelength at 440 nm) to quantify BP fluorometrically.

3. Results

3.1. Construction of an *E. coli* strain producing BH4

Unlike higher organisms, most microorganisms including *E. coli* do not have the BH4 biosynthetic pathway. *E. coli*, though, has an endogenous GCHI gene *folE*, which participates in folic acid biosynthesis. We chose therefore to reconstruct the mammalian BH4 biosynthetic pathway in *E. coli* with recombinant DNA technology.

A variety of expression plasmids carrying the *folE* gene from *E. coli*, and the rat liver cDNA genes expressing the PTPS and SPR enzymes were constructed, all of which utilized the *E. coli lac* promoter for expression from pUC18/19, pSTV28/29, or pMW218/219. These expression plasmids (Table 1) have different origins of replication, resulting in different plasmid copy numbers in the host, as well as different selection markers. Using this set of plasmids, the gene products can be expressed at different levels depending upon copy number. In addition, choosing different plasmids allows the co-expression of any combinations of genes. We tested various three-plasmid combinations in order to best assemble the pathway from GTP to BH4 in *E. coli* JM101. In a few combinations, such as JM101/(pSTV28folE, pUC18PTPS, pMW219SPR) and JM101/(pSTV28folE, pMW218PTPS, pUC19SPR), BP and pterin (P) were slightly detectable in the culture media, whereas they were not detected in culture media of native host cells (data not shown). These results strongly suggested that these recombinant *E. coli* strains gained the ability for BH4 production. BP and P were likely formed by natural oxidization and/or degradation of BH4 (Davis et al., 1988; see Fig. 2).

Next, we constructed pSTV28GPS (Fig. 3A), which carried all three essential genes for BH4 production. In that plasmid, the three genes are located in the order of enzymatic reactions and each has a *lac* promoter/operator region so that each gene can be induced simultaneously. We transformed pSTV28GPS into JM101, and confirmed production of FolE and PTPS proteins by SDS-PAGE (data not shown). SPR protein

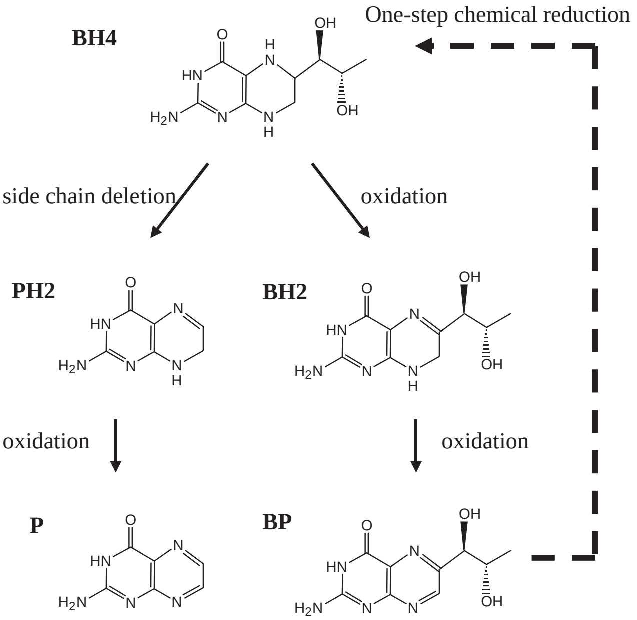

Fig. 2. Hypothetical BH4 oxidation and degradation pathways. BH4 is susceptible to natural oxidation and side chain deletion, yielding biopterin and pterin compounds which were detected in culture broths. BH4 was measured as BP after biopterin compounds were converted to BP by an oxidizing agent. When necessary, BH4 is obtained by one-step reduction of BP. Abbreviations used: PH2, dihydropterin; P, pterin; BH2, 7,8-dihydrobiopterin; BP, biopterin.

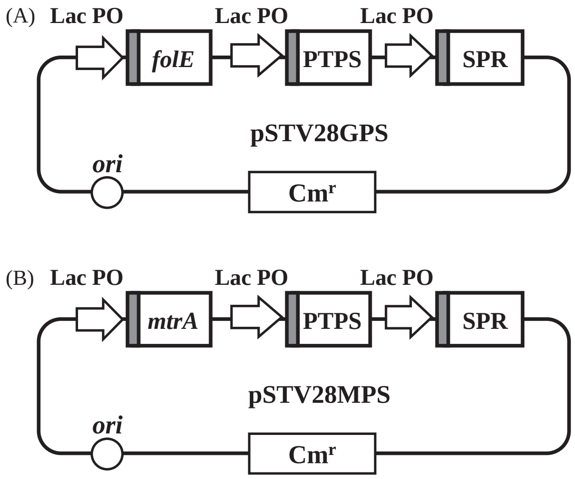

Fig. 3. The structures of expression vectors pSTV28GPS and pSTV28MPS. The vector pSTV28GPS (A) or pSTV28MPS (B), which contains *folE* gene [X63910] from *E. coli* or *mtrA* gene [M37320] from *B. subtilis* for GCH I, PTPS gene [NM_017220] from rat liver, and SPR gene [M36410] also from rat liver, was introduced into *E. coli* cells to have them produce biopterin compounds. Each gene has short amino acid sequence tag at its N terminus indicated by a shaded box. The tag is MTMITNS for *folE, mtrA* and PTPS and MTMITPSL for SPR, these are derived from the N-terminus of the *lacZ* gene sequence. LacPO, *lac* promoter and operator sequence.

could not be identified by the analysis. The strain was cultured for 48 h in test tubes and biopterin compounds were detected in the culture media by fluorescence

HPLC analysis. The BH4 productivity of this primary *E. coli* strain was 23 mg/l of culture broth.

3.2. Host strain improvement by purine analogue-resistant mutations

GTP is the starting material for BH4 biosynthesis; therefore, we hypothesized that the capacity of de novo GTP synthesis in host cells is a limiting factor for high BH4 production. We isolated purine analogue-resistant mutants in order to obtain host cells that were released from negative feedback of purine nucleotide(s) synthesis and have enhanced de novo GTP supply (Perkins et al., 1999). More than thirty 8-azaguanine-resistant mutants were obtained by mutagenesis of JM101 strain and evaluated for BH4 productivity by transforming with pSTV28GPS. HPLC analysis showed that each resistant clone had several-fold increased BH4 production (data not shown). Among those clones, the AG14/pSTV28GPS strain had BH4 productivity up to 429 mg/L of culture broth. This improvement suggested that enhancing the purine nucleotide(s) supply was one of the most critical elements to achieving high productivity.

We performed additional mutagenesis of JM101 and AG14 cells followed by selection with 8-azaguanine or decoyinine, another purine analogue. However, no mutant having higher productivity than AG14/pSTV28GPS was obtained (data not shown). We adopted the AG14 as the host cell line in the following studies.

3.3. Introduction of guaBA gene expression to enhance BH4 productivity

In the culture medium of AG14/pSTV28GPS, BH2, BP, and P were identified by fluorescent HPLC analysis before oxidization. Several unknown intermediates or possible by-products were detected as well. One of those peaks corresponded with the retention time of GMP or IMP, which was indistinguishable by HPLC (data not shown). As shown in Fig. 4, many metabolic enzymes are involved in the biosynthetic pathway from pRpp to GTP in *E. coli*. IMP is converted to GMP via XMP by *guaB* and *guaA* gene products, and GMP to GDP by *gmk* (Zalkin and Nygaard, 1996). The appearance of intermediate(s) of the GTP synthetic pathway suggested that the 8-azaguanine-resistant strain was released from negative feedback regulation of purine nucleotide(s) synthesis as expected, which resulted in accumulation of IMP, GMP or other intermediate(s). Thus, we assumed that an additional introduction of *guaBA* and/or *gmk* genes would lead to further increased GTP supply.

We examined the effects of adding the *guaBA* and/or *gmk* gene, at different copy numbers to vary the expression levels in AG14/pSTV28GPS by using two

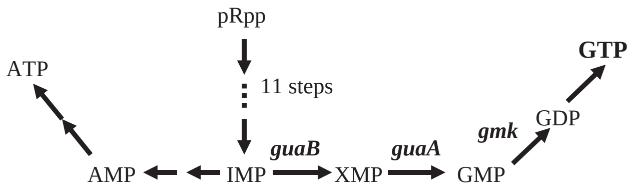

Fig. 4. The purine nucleotide synthetic pathways. IMP is synthesized from pRpp in an 11-step reaction, and is the starting material for GTP and ATP biosyntheses. The genes *guaB*, *guaA* and *gmk* encode IMP dehydrogenase, GMP synthase and GMP kinase respectively, and each enzyme catalyzes the indicated reaction. Abbreviations used: pRpp, 5-phosphoribosyl-α-1-pyrophosphate; IMP, inosine 5′-monophosphate; AMP, adenosine 5′-monophosphate; ATP, adenosine 5′-triphosphate; XMP, xanthosine 5′-monophosphate; GMP, guanosine 5′-monophosphate; GDP, guanosine 5′-diphosphate; GTP, guanosine 5′-triphosphate.

plasmids pTWV228 and pMW218. Genes carried by pTWV228 should be expressed at high levels and by pMW218 at lower levels (Table 1, Fig. 5A). We transformed them into AG14/pSTV28GPS in various combinations and investigated the growth and BH4 productivity.

Data of three clones from each plasmid combination are shown as a scatter-plot in Fig. 5B. Strains carrying *guaBA* in low-copy resulted in higher BH4 productivity and cell growth (Fig. 5B, cell type 1). Higher expression of *guaBA* gene (cell type 2) did not lead to higher productivity than cell type 1. The data showed that no improvement in BH4 productivity was obtained by expression of *gmk* at either level (Fig. 5B, cell types 3 and 4 compared to control); neither did the expression of *gmk* improve the increased BH4 productivity by *guaBA* (cell type 5 compared to cell type 1, and cell type 6 compared to cell type 2). The unidentified HPLC peak described above was significantly decreased by the presence of *guaBA* but not *gmk* (data not shown), supporting our assumption. The maximum productivity of BH4 in strain AG14/(pSTV28GPS, pMW218guaBA) was 718 mg/L broth in a tube culture for 40 h (cell type 1).

3.4. Expression balance of folE, PTPS and SPR in AG14/(pSTV28GPS pMW218guaBA)

In the previous studies, we performed host cell mutagenesis and tested the role of *guaBA* addition. These two improvements enhanced the supply of GTP, and BH4 productivity reached to 718 mg/L compared to 23 mg/L of the primary BH4 producing cell strain.

Next, we focused on the pathway from GTP to BH4 and investigated the expression balance of FolE, PTPS and SPR. An additional single gene (*folE* or PTPS or SPR) was introduced into AG14/(pSTV28GPS, pMW218guaBA) so as to optimize exogenous gene expression. In this experiment, we used pTWV228 or

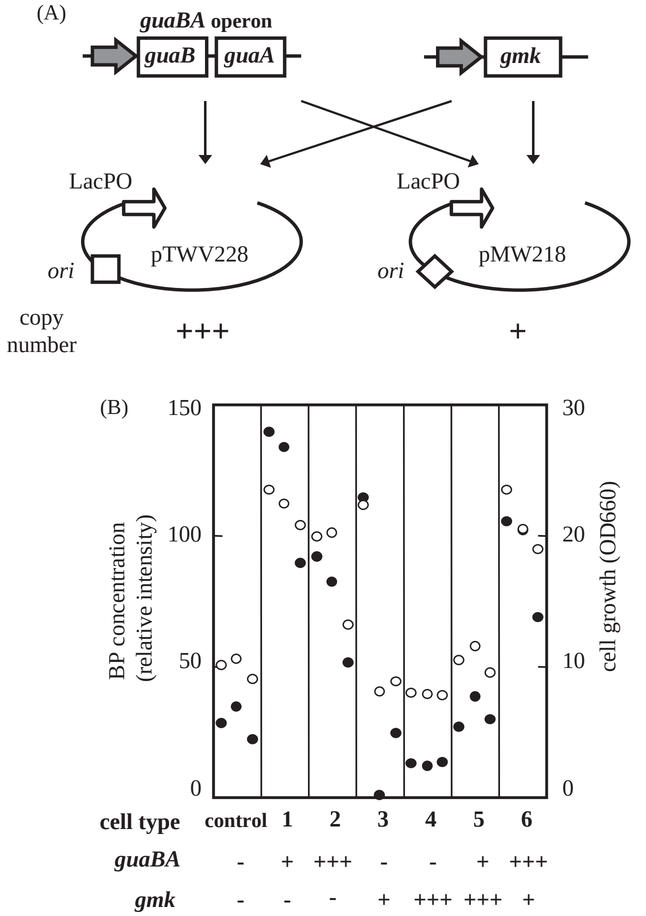

Fig. 5. The effect of introducing of *guaBA* and *gmk* genes. Construction of expression vectors (A). The *guaBA* operon or the *gmk* gene, including their native promoters indicated by shadowed arrows, were inserted into pTWV228 or pMW218 expression plasmids. The marks (+, + + +) indicate relative copy number in *E. coli*. BP concentration (solid circle) and cell growth (open circle) of transformed cells (B). Each plasmid was transformed into BH4 producing cell AG14/pSTV28GPS in some combination. Three clones from each genotype were tube-cultured for 40 h and analyzed for BP concentration by fluorometer and cellular growth. The marks (+, + + +) indicate relative copy number in *E. coli*. The minus symbol (–) means that the gene was not introduced.

pTWV229 as the expression vector, which can strongly express the inserted gene and is selected by ampicillin.

Results of SDS-PAGE, OD_{660} and HPLC analysis in a 3 ml scale tube culture experiment are shown in Fig. 6. In all the three strains, BH4 productions were decreased compared to the control. SDS-PAGE analysis demonstrated that the amount of protein in each strain seemed to be nearly saturated and did not show a simple one gene product augmentation that we had expected. The strain harboring the additional *folE* gene (Fig. 6, cell type 1) did not enhance expression of FolE but showed decreased expression of PTPS compared to control. The PTPS augmented strain (cell type 2) showed increased PTPS but decreased FolE and GuaB/A. The SPR augmented strain (cell type 3) did not show a clear

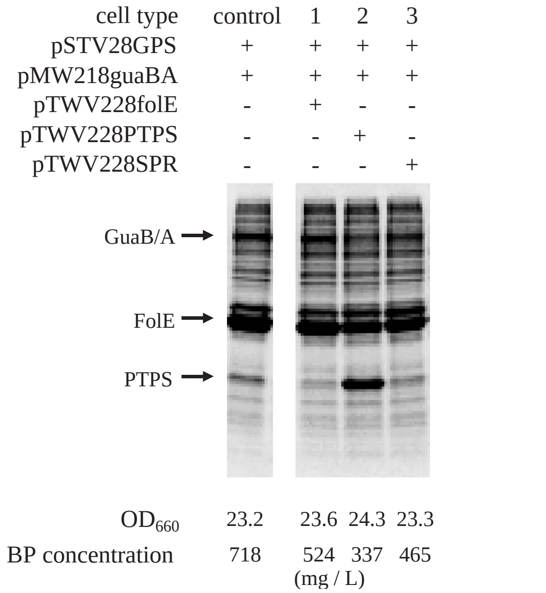

Fig. 6. The expression patterns of exogenous genes in BH4 producing cells. The genes *folE*, PTPS and SPR were introduced (cell types 1, 2, and 3, respectively) into AG14/(pSTV28GPS, pMW218guaBA) strain (control). The cell growth (OD_{660}) of the four strains was similar, and BP concentration decreased compared to the control by introducing these genes. SDS-PAGE analysis showed a positive correlation between FolE expression level and BH4 productivity (see the text).

SPR protein band despite the findings that FolE and GuaB/A levels were decreased. The results suggested that the capacity of protein synthesis of the control strain AG14/(pSTV28GPS, pMW218guaBA) was at its maximum, and that the protein expression balance in the control cells was comparatively appropriate. This leads to the conclusion that further introduction of synthetic genes had affected the protein expression balance and caused a detrimental effect on BH4 productivity. Since the amount of expressed proteins was estimated to be more than 50% of total protein, even in the control strain, introduction of additional genes might decrease cell activity, resulting in lower BH4 productivity.

However, when we compared the control with strains carrying additional PTPS and SPR genes (control, cell types 2 and 3), we could see a positive correlation between FolE expression level and BH4 productivity. Although the amount of FolE protein is considerably higher than PTPS, SPR and GuaB/A in control cells, BH4 productivity decreased with the decreased expression of FolE. Therefore, the possibility that an improvement of the conversion efficiency of GCHI without decreased PTPS and SPR expression would augment BH4 productivity was expected.

3.5. Utilization of mtrA, a GCHI homologue of B. subtilis

The enzymatic characterization of GCHI from several different organisms, such as *Streptomyces tubercidicus*,

E. coli, *B. subtilis*, mouse, rat and human, has been thoroughly studied and described (Yoo et al., 1998; Cha et al., 1991; Hatakeyama et al., 1989; Shen et al., 1989). *B. subtilis* GCHI, encoded by *mtrA,* has been reported to have an apparent Km value for GTP of 4 μM (de Saizieu et al., 1995), whereas another group reported that purified FolE enzyme from *E. coli* has a higher Km of 100 μM (Schoedon et al., 1992). We expected that using the *mtrA* gene instead of *folE* might improve the conversion efficiency of the GCHI catalyzed reaction.

The expression plasmid pSTV28MPS was constructed by substituting the *mtrA* gene for *folE* (Fig. 3B). Then, we compared the production of BH4 between the strains, AG14/(pSTV28GPS, pMW218guaBA) and AG14/(pSTV28MPS, pMW218guaBA), in 3 ml tube cultures. In the latter strain BH4 productivity was slightly increased, while the cell growth had decreased (Fig. 7A). These data suggest that the enzyme activity of the MtrA protein might be higher than that of FolE in the strain, which led to higher BH4 productivity.

BH4 productivity of the established strains, i.e., AG14/(pSTV28GPS, pMW218guaBA) and AG14/(pSTV28MPS, pMW218guaBA), was evaluated by glycerol fed-batch fermentation in a 3L bench scale fermentor (Fig. 7B). Cellular growth was steadily increased and reached an OD_{660} of 90 in both cell lines by 40 h. As for BH4 productivity, 4.0 g/L of BP was contained in the final culture broth of AG14/(pSTV28MPS, pMW218guaBA), and 2.4 g/L in AG14/(pSTV28GPS, pMW218guaBA). Their cellular growth, BP and glycerol concentrations are shown in Fig. 7B. Thus, a 1.5-fold improvement in BH4 productivity was obtained by the substitution of GCHI genes. These interesting results suggested that use of the foreign *mtrA* gene from *B. subtilis* was more efficacious than the native enzyme of the host.

Protein expression patterns of AG14/(pSTV28GPS, pMW218guaBA) and AG14/(pSTV28MPS, pMW218 guaBA) strains were analyzed by SDS-PAGE (data not shown). The protein expression patterns were quite similar between the two strains. However, inclusion body formation was observed in the latter strain but not in the former. During cultivation, at least 30% of expressed MtrA protein was found in inclusion bodies (data not shown). These data suggest a further improvement of BH4 productivity might be achieved by preventing inclusion body formation.

4. Discussion

When we began this study, no information was available to predict whether the physical properties and characteristics of BH4 made it feasible for its biosynthetic production by recombinant microorganism for the following reasons. First, since biopterin compounds have a similar pteridine ring structure as folic acid, there was the possibility that large amounts of biopterin compounds might act as folic acid analogues that have harmful effects on host cells. This turned out not to be the case in our study. Second, BH4 is labile to oxidation, and is naturally oxidized readily to BH2 or BP (see Fig. 2), which we detected in culture broths. We therefore estimated BH4 production levels by measuring BP, which is relatively stable and can be chemically reduced to BH4 through a single step when necessary. We carried out the oxidation of BH4 or BH2 in culture broth to BP, which was detected quantitatively by fluorescence HPLC analysis.

Since three exogenous genes had to be introduced coordinately, we co-transformed three different plasmids harboring *folE*, and PTPS and SPR expressing genes, respectively, in various combinations for the initial experiments (see Table 1). SDS-PAGE and HPLC

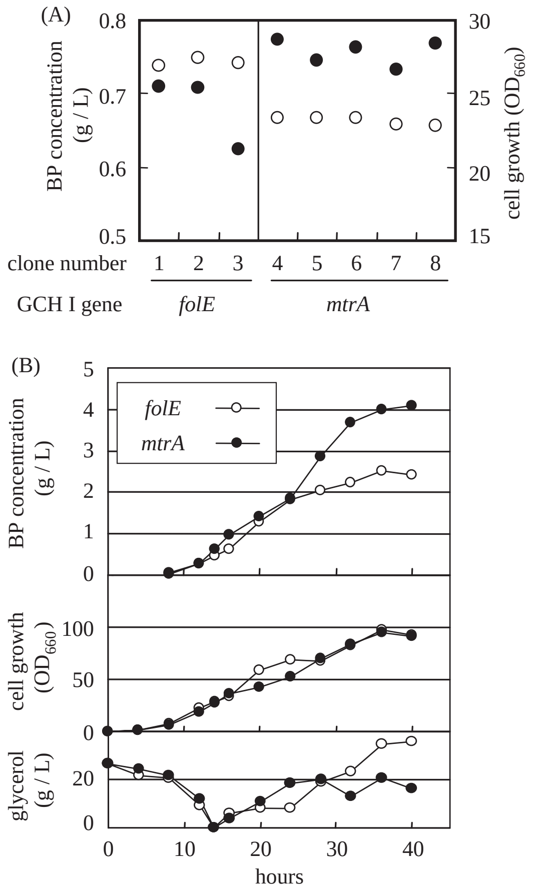

Fig. 7. Comparison of the *B. subtilis mtrA* gene with *folE.* A. Scatter plot showing BH4 productivity as BP concentration determined by HPLC analysis and cell growth data of each clone. Cell clones 1–3, AG14/(pSTV28GPS, pMW218guaBA); cell clones 4–8, AG14/(pSTV28MPS, pMW218guaBA) were compared in 3 ml tube cultures. B. Fermentation study showing BP concentration, cellular growth and glycerol in culture media as indicated. The strains AG14/(pSTV28GPS, pMW218guaBA) (open circle) and AG14/(pSTV28MPS, pMW218guaBA) (solid circle) were shown.

analyses of those experiments provided us with some insights on the characteristics of each enzyme when expressed in *E. coli*. For instance, the specific activity of SPR was relatively high since its reaction was readily catalyzed even though the expressed protein could not be identified by SDS-PAGE analysis (see Fig. 6). When we introduced an SPR gene that was strongly expressed from the high copy number plasmid pUC19, inclusion bodies were formed in the host cells (data not shown), which generally should be avoided so as not to inactivate the expressed enzymes with the subsequent loss of energy and protein coding ability.

To optimize the expression balance of GCHI, PTPS and SPR, we constructed various expression plasmids. However, combinations of multiple plasmids are not optimal for industrial production, because they require multiple antibiotics for selection and maintenance and may still be unstable in fermentations. Fortunately, the vector pSTV28GPS (Fig. 3A), which carries all three genes and is stable in host cells with chloramphenicol, demonstrated superior expression balance and satisfactory BH4 productivity compared to strains with three plasmids. Thus, BH4 producing *E. coli* strain harboring pSTV28GPS became the basis of the present study for the improvements.

Fig. 8 summarizes the results of the present investigations. First, we assumed that it was necessary to redirect and enhance the carbon flux to the target pathway. The starting material for BH4 synthesis is GTP; therefore, we increased its supply in two ways. The use of an 8-azaguanine resistant mutant led to a considerable improvement. Next, *guaBA* addition also made a significant contribution in augmenting BH4 productivity. These improvements were accomplished by combination of a classical mutant selection and rational targeting of specific enzyme reactions that were rate-limiting.

Second, we noted that the strain with the pSTV28MPS plasmid (Fig. 3B) containing the GCHI gene *mtrA* derived from *B. subtilis* was superior to the strain with pSTV28GPS harboring *folE* from *E. coli* for BH4 production (Fig. 7A). It was quite interesting that an enzyme derived from the host cells was less suitable than the homologous enzyme from a foreign species. A high-density fermentor cultivation experiment showed a more distinct difference of the two strains, in which AG14/(pSTV28MPS, pMW218guaBA) reached higher BP concentration than AG14/(pSTV28GPS, pMW218guaBA) while both strains showed similar growth curves. According to enzymatic analysis of each protein, two possibilities can be proposed. One is the difference of Km values for GTP; the other is that FolE enzyme activity, but not MtrA, could be inhibited by BH4 (de Saizieu et al., 1995; Schoedon et al., 1992). In addition, we observed inclusion body formation in AG14/(pSTV28MPS, pMW218guaBA) but not in

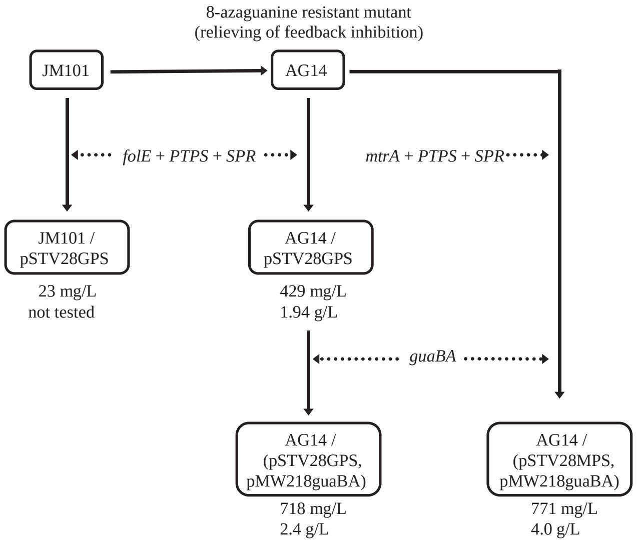

Fig. 8. A series of established BH4 producing *E. coli* strains. Expression plasmid and *E. coli* strain are shown in boxes. Solid arrows indicate alterations of recombinant cells starting from JM101. Broken arrows indicate the introduced gene(s). The concentrations of BP in 3 ml tube culture and 2L fermentation are written under each box (upper, tube culture; lower, fermentor). The data are the highest value of each strain from many independent experiments.

AG14/(pSTV28GPS, pMW218guaBA). SDS-PAGE analysis indicated that the inclusion bodies were mostly composed of *mtrA* gene product (data not shown). This suggests that AG14/(pSTV28MPS, pMW218guaBA) may have even further room for improvement.

In the present study, we observed a positive correlation between BH4 production and cell proliferation (Fig. 5B). In other words, we did not obtain any strains with low cell growth and high BH4 productivity. The fact might be explained by the various roles of GTP in living cells. GTP, the starting material for the BH4 synthetic pathway, also functions as an essential molecule for cellular activity, such as a material for RNA synthesis or an energy source for translation. Cell growth requires adequate GTP and activates GTP synthesis, while deceleration of cell growth might inhibit the GTP supply. Therefore, to attain higher BH4 productivity, recombinant *E. coli* would require culture conditions suitable for cell growth in order to retain adequate GTP supplies.

The final goal of our study is to establish an industrially attractive production method for BH4. Much work, including further genetic improvements of the host, purification of BP, and establishing re-reduction processes will be required. However, this report demonstrates the potential for BH4 production in large amounts. When established, BH4 production using recombinant *E. coli* will be superior to chemical synthesis in terms of both cost and operational procedures. It may also contribute to progress in research on this cofactor.

Acknowledgments

We thank Dr. F. Iwasa for critical reading of the manuscript and helpful discussions. We also thank Prof. Y. Fujita (Fukuyama Univ., Japan) for the *B. subtilis* strain.

References

Cha, K.W., Jacobson, K.B., Yim, J.J., 1991. Isolation and characterization of GTP cyclohydrolase I from mouse liver. Comparison of normal and the *hph*-1 mutant. J. Biol. Chem. 266, 12294–12300.

Davis, M.D., Kaufman, S., Milstien, S., 1988. The auto-oxidation of tetrahydrobiopterin. Eur. J. Biochem. 173, 345–351.

de Saizieu, A., Vankan, P., van Loon, A.P.G.M., 1995. Enzymic characterization of *Bacillus subtilis* GTP cyclohydrolase I. Evidence for a chemical dephosphorylation of dihydroneopterin triphosphate. Biochem. J. 306, 371–377.

Hatakeyama, K., Harada, T., Suzuki, S., Watanabe, Y., Kagamiyama, H., 1989. Purification and characterization of rat liver GTP cyclohydrolase I. Cooperative binding of GTP to the enzyme. J. Biol. Chem. 264, 21660–21664.

Hatakeyama, K., Hoshiga, M., Suzuki, S., Kagamiyama, H., 1993. Enzymatic synthesis of 6R-[U-^{14}C]tetrahydrobiopterin from [U-^{14}C]GTP. Anal. Biochem. 209, 1–5.

Inoue, H., Nojima, H., Okayama, H., 1990. High efficiency transformation of *E. coli* with plasmids. Gene 96, 23–28.

Lovett, P.S., Keggins, K.M., 1979. *Bacillus subtilis* as a host for molecular cloning. Methods Enzymol. 68, 342–357.

Miller, J.H., 1972. Nitrosoguanidine mutagenesis. In: Experiments in Molecular Genetics. Cold Spring Harbor Laboratory, Cold Spring Harbor, New York, pp. 125–129.

Milstien, S., Kaufman, S., 1989. The biosynthesis of tetrahydrobiopterin in rat brain. Purification and characterization of 6-pyruvoyl tetrahydropterin (2′-oxo) reductase. J. Biol. Chem. 264, 8066–8073.

Nakamura, K., Bindokas, V.P., Kowlessur, D., Elas, M., Milstien, S., Marks, J.D., Halpern, H.J., Kang, U.J., 2001. Tetrahydrobiopterin scavenges superoxide in dopaminergic neurons. J. Biol. Chem. 276, 34402–34407.

Perkins, J.B., Sloma, A., Hermann, T., Theriaut, K., Zachgo, E., Erdenberger, T., Hannett, N., Chatterjee, N.P., Williams, V.II, Rufo Jr., G.A., Hatch, R., Pero, J., 1999. Genetic engineering of *Bacillus subtilis* for the commercial production of riboflavin. J. Ind. Microbiol. Biotechnol. 22, 8–18.

Schoedon, G., Redweik, U., Frank, G., Cotton, R.G., Blau, N., 1992. Allosteric characteristics of GTP cyclohydrolase I from *Escherichia coli*. Eur. J. Biochem. 210, 561–568.

Shen, R.S., Alam, A., Zhang, Y.X., 1989. Human liver GTP cyclohydrolase I: purification and some properties. Biochimie 71, 343–349.

Silhavy, T.J., Berman, M.L., Enquist, L.W., 1984. DNA extraction from bacterial cells. In: Experiments with Gene Fusions. Cold Spring Harbor Laboratory, Cold Spring Harbor, New York, pp. 137–139.

Smith, G.K., Nichol, C.A., 1986. Synthesis, utilization, and structure of the tetrahydropterin intermediates in the bovine adrenal medullary *de novo* biosynthesis of tetrahydrobiopterin. J. Biol. Chem. 261, 2725–2737.

Takikawa, S., Curtius, H.C., Redweik, U., Leimbacher, W., Ghisla, S., 1986. Biosynthesis of tetrahydrobiopterin. Purification and characterization of 6-pyruvoyl-tetrahydropterin synthase from human liver. Eur. J. Biochem. 161, 295–302.

Thöny, B., Auerbach, G., Blau, N., 2000. Tetrahydrobiopterin biosynthesis, regeneration and functions. Biochem. J. 347, 1–16.

Werner-Felmayer, G., Golderer, G., Werner, E.R., 2002. Tetrahydrobiopterin biosynthesis, utilization and pharmacological effects. Curr. Drug Metab. 3, 159–173.

Yoo, J.C., Han, J.M., Ko, O.H., Bang, H.J., 1998. Purification and characterization of GTP cyclohydrolase I from *Streptomyces tubercidicus*, a producer of tubercidin. Arch. Pharm. Res. 21, 692–697.

Zalkin, H., Nygaard, P., 1996. Biosynthesis of purine nucleotide. In: Neidhardt, F.C., Editor in Chief. *Escherichia coli* and *Salmonella* Cellular and Molecular Biology, 2nd Edition, Vol. 1. ASM Press, Washington, DC, pp. 561–579.
