# Supplementary material for: 5-Hydroxytryptophan (5-HTP): Natural Occurrence, Analysis, Biosynthesis, Biotechnology, Physiology and Toxicology
Source: Int J Mol Sci. 2020 Dec 26;22(1):181. doi: 10.3390/ijms22010181 (PMC7796270; doi:10.3390/ijms22010181)
Supplement: Supplementary file 1 [file ijms-22-00181-s001.zip › 5-HTP.Data/PDF/3951989297/Luo-2020-Microbial-synthesis-of-human-hormon.pdf]

ACS Synthetic Biology

pubs.acs.org/synthbio Letter

Microbial Synthesis of Human-Hormone Melatonin at Gram Scales

Hao Luo,*,‖ Konstantin Schneider,‖ Ulla Christensen, Yang Lei, Markus Herrgard, and Bernhard Ø. Palsson

Cite This: *ACS Synth. Biol.* 2020, 9, 1240−1245 Read Online

ACCESS | Metrics & More | Article Recommendations | Supporting Information

ABSTRACT: Melatonin is a commercially attractive tryptophan-derived hormone. Here we describe a bioprocess for the production of melatonin using *Escherichia coli* to high titers. The first engineered strain produced 0.13 g/L of melatonin from tryptophan under fed-batch fermentation conditions. A 4-fold improvement on melatonin titer was further achieved by (1) protein engineering of rate-limiting tryptophan hydroxylase to improve 5-hydroxytryptophan biosynthesis and (2) chromosomal integration of aromatic-amino-acid decarboxylase to limit byproduct formation and to minimize gene toxicity to the host cell. Fermentation optimization improved melatonin titer by an additional 2-fold. Deletion of *yddG*, a tryptophan exporter, exhibited an additive beneficial effect. The final engineered strain produced ∼2.0 g/L of melatonin with tryptophan supplemented externally and ∼1.0 g/L with glucose as the sole carbon source for tryptophan supply. This study lays the foundation for further developing a commercial melatonin-producing *E. coli* strain.

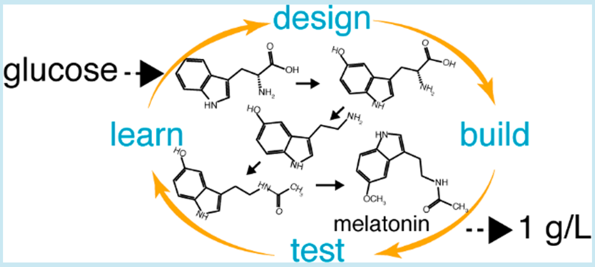

KEYWORDS: *fermentation, glucose, tryptophan, E. coli, high titer*

Downloaded via UNIV DEGLI STUDI DI TORINO on November 17, 2020 at 16:57:50 (UTC).
See https://pubs.acs.org/sharingguidelines for options on how to legitimately share published articles.

Melatonin is a tryptophan-derived compound. It is a natural hormone that is primarily released by the pineal gland and regulates the circadian cycle in humans. Medicinally, it is used as a sleep aid and can be purchased over-the-counter as a dietary supplement in some countries. Today, industrial production of melatonin relies on chemical synthesis, which is neither sustainable nor environmentally friendly. Bioproduction of melatonin using engineered microbes and renewable feedstocks offers a sustainable manufacturing alternative.

The melatonin biosynthetic pathway consists of four enzymatic steps starting from tryptophan (Figure 1). The first step is hydroxylation of tryptophan to form 5-hydroxytryptophan (5HTP) catalyzed by tryptophan hydroxylase (TrpH). This is followed by decarboxylation of 5HTP by aromatic-amino-acid decarboxylase (Ddc) resulting in the synthesis of 5-hydroxytryptamine (5HT). 5HT is further acetylated to produce acetyl-5-hydroxytryptamine (AcHT) by aralkylamine acetyltransferase (Aanat). Lastly, AcHT is methylated to form melatonin through the action of acetylserotonin *O*-methyltransferase (Asmt) that is *S*-adenosylmethionine (SAM) dependent. Microbial production of melatonin at a titer of 14 mg/L from glucose using *Saccharomyces cerevisiae* has been described previously.[1] Additional improvements were hampered, however, by the efficacy of tryptophan hydroxylation and methylation in yeast. Findings based on directed metabolic pathway evolution suggest that efficient aromatic-amino-acid hydroxylation and SAM-dependent methylation are achievable in *Escherichia coli*.[2,3] In this communication, bioproduction of melatonin to high titers is investigated by introducing the recombinant melatonin biosynthetic pathway to *E. coli*.

RESULTS AND DISCUSSION

Initial Melatonin-Producing Strains. We started to assemble the first melatonin-producing strain by bioprospecting functional Ddc, Aanat, and Asmt. Each of the candidate gene was expressed in a medium copy plasmid under the control of a constitutive promoter. The enzyme activity was tested in a small-scale bioproduction assay (Methods) by feeding the substrates. The *ddc* genes were widely distributed in the biological kingdoms and seven *ddc* genes originating from bacteria and human were selected for evaluation. Bioconversion of 5HT from 5HTP was assessed directly in whole cells and results showed those from *Candidatus Koribacter versatilis Ellin* 345 and *Oryza sativa Japonica Group* outperformed the others (Table S1). The *ddc* gene from *C. Koribacter versatilis Ellin* was selected due to its low tryptamine (TRPM)-forming activity, a feature that reduces byproduct formation (Figure 1). Five *aanat* genes were tested for biotransformation of 5HT to AcHT. Cells bearing *aanat* from human and *Streptomyces griseofuscus* accumulated the

Received: February 7, 2020
Published: June 5, 2020

ACS Publications © 2020 American Chemical Society

https://dx.doi.org/10.1021/acssynbio.0c00065
ACS Synth. Biol. 2020, 9, 1240−1245

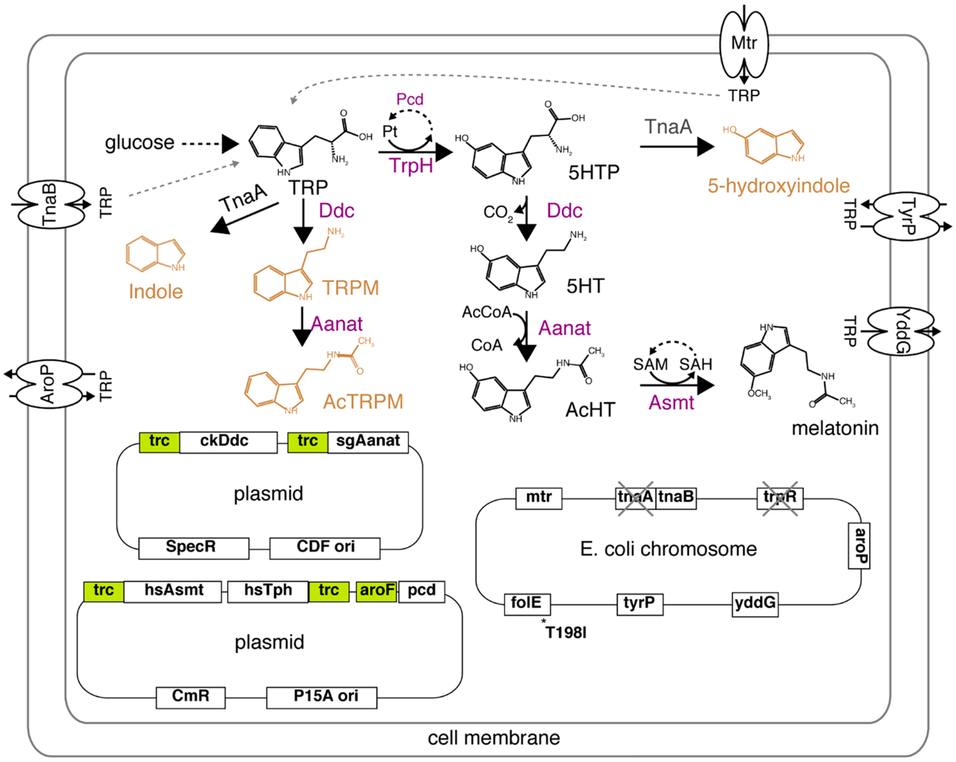

Figure 1. Melatonin pathway. Enzymatic transformations of melatonin from tryptophan are shown. Recombinant enzymes are colored in purple and native proteins and enzymes are colored in black. Byproducts of the melatonin pathway are colored in orange. Cross indicates deletion of *E. coli* genes. Plasmids carrying the heterologous genes encoding melatonin pathway are illustrated.

most AcHT under testing conditions. The *S. griseofuscus aanat* gene was chosen to construct the melatonin strain due to its bacterial origin. Lastly, seven mammalian *asmt* genes were studied for direct synthesis of melatonin from AcHT in *E. coli* (Table S1). The human *asmt* gene accumulated the most melatonin and was selected.

The first melatonin-producing strain, HM175, was assembled. Recombinant genes encoding the melatonin pathway were expressed from two plasmids (Figure 1). The *ddc* and *aanat* genes were harbored on a medium-copy-number plasmid. The *asmt* gene was placed with *trpH* and *pcd*, encoding pterin-4α-carbinolamine dehydratase required for tryptophan hydroxylation, in a low-copy-number plasmid. Heterologous TrpH was a truncated form of human TrpH2 with an E2K mutation for improved protein abundance.[2] Expression of the recombinant genes were individually achieved from a trc promoter except for *pcd*, whose expression was under the control of the *E. coli* aroF promoter. The background *E. coli* strain was a plasmidless isolate described in Luo *et al.* (2020)[2] and it carried a chromosomal FolE (T198I) mutation that was essential for achieving efficient tryptophan hydroxylation. Deletion of *tnaA* (tryptophanase) and *trpR* (tryptophan transcription repressor) genes was additionally introduced. Δ*tnaA* was necessary to prevent 5HTP degradation[4] and Δ*trpR* was to deregulate expression of genes involved in tryptophan synthesis and transportation.[5,6]

HM175 was characterized by fed-batch fermentation. When glucose and tryptophan were fed, it was observed that melatonin production correlated strongly to the growth of *E. coli* cells and its level reached a maximum titer of 130 mg/L at 50 h when *E. coli* growth ceased (Figure 2A). Two main byproducts observed in the fermentation broth were AcHT and acetyl-tryptamine (AcTRPM). Formation of AcTRPM was likely due to decarboxylation of tryptophan and subsequent acetylation *via* Aanat. AcHT was continuously produced even when cell growth was arrested, suggesting active growth might be required for SAM-dependent methylation. During fermentation, the *E. coli* cells grew at a maximum growth rate of 0.1 h^{-1} even though a feeding rate for growth of 0.25 h^{-1} was applied. Only 13 g/L of dry biomass was achieved while the wildtype strain, BW25113, could reach 60 g/L under similar conditions.

Improvement on biomass formation was attempted next, since melatonin production might require actively growing cells. It was observed acetate was produced continuously during the course of fermentation to levels over 100 mM which could prevent *E. coli* growth (Figure 2A).[7,8] One cause of acetate accumulation could be attributed to the lack of essential growth factors such that *E. coli* cells started to ferment glucose for energy production *via* mixed acid fermentation.[9,10] Notably, the *E. coli* strain used was Δ*tyrA*, implemented as a part of the genetic selection for improving tryptophan hydroxylation.[2] Deletion of *tyrA* resulted in tyrosine auxotrophy. Although *de novo* tyrosine synthesis from phenylalanine *via* TrpH could be achieved in HM175, it might be limited under studied conditions thus representing a potential contributing factor for acetate formation during fermentation.

The next melatonin-producing strain, HM231, was generated by restoring *tyrA*. Under comparable fermentation conditions to HM175, biomass formation was improved by ~3 fold (30 g/L, $p < 0.00295$) with little acetate secretion observed (Figure 2B, Table 1). AcTRPM (up to 250 mg/L) was the only byproduct that dominantly appeared in the fermentation broth (Figure 2B). The melatonin titer was

https://dx.doi.org/10.1021/acssynbio.0c00065
ACS Synth. Biol. 2020, 9, 1240−1245

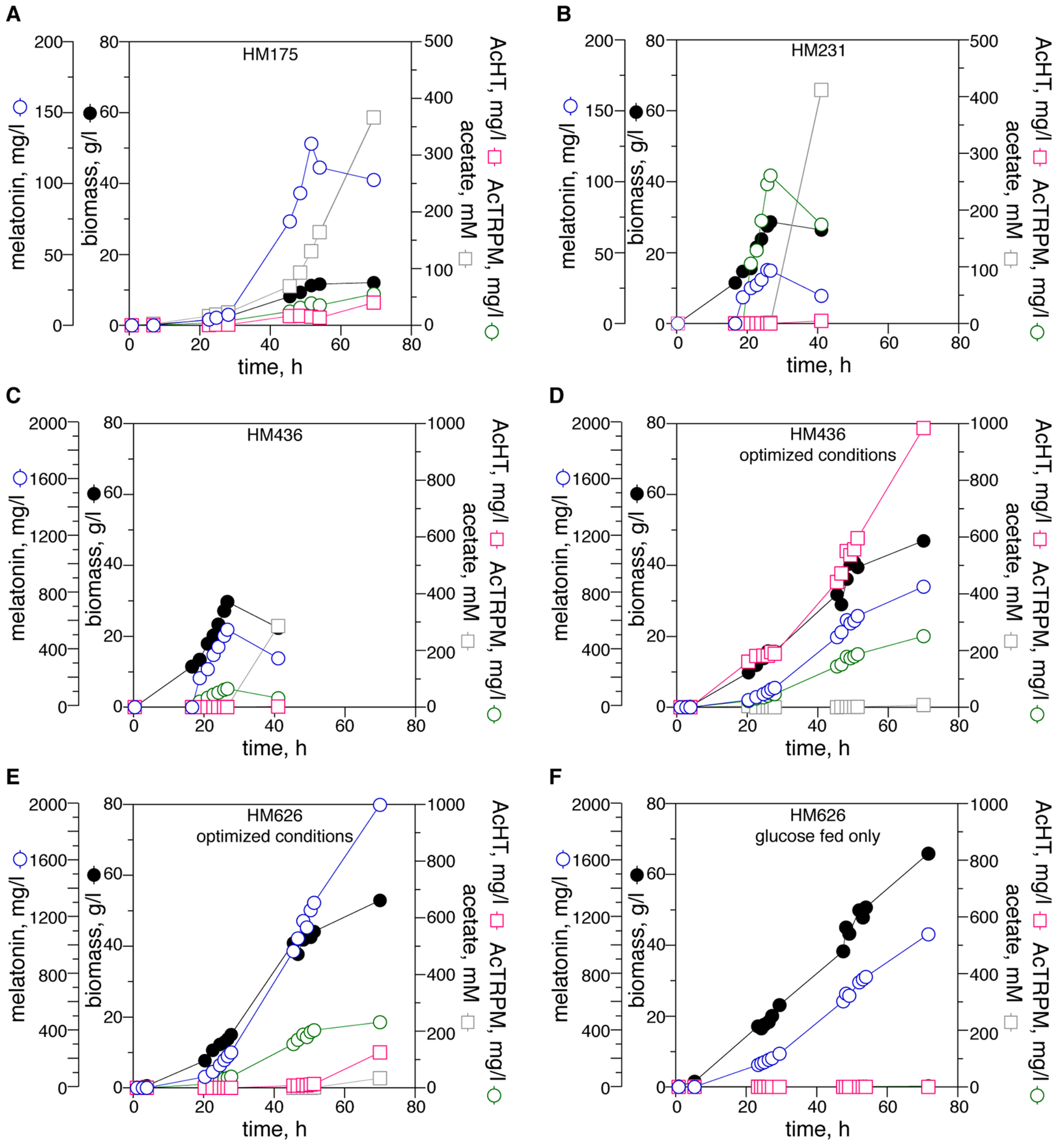

Figure 2. Representative performance of melatonin-producing strains in fed-batch fermentation. Tryptophan was fed for all runs expect Panel F. HM175 (A), HM231 (B), HM436 (C), HM436 under optimized conditions (D), HM626 under optimized conditions (E), HM626 in the absence of tryptophan feeding (F).

Table 1. Performance of Melatonin Producing Strains in Fed-Batch Fermentation

parameters[c]	HM175 (n = 3)	HM231 (n = 2)	HM436 (n = 3)	HM436[a] (n = 2)	HM626[a] (n = 2)	HM626[a,b] (n = 2)
5HTP (mg/L)	5.0 ± 0.5	0	1.5 ± 0.9	0	0	0
HT (mg/L)	0	0	1.5 ± 0.3	7.4 ± 0.01	6.4 ± 1.2	0
AcHT (mg/L)	14.5 ± 7.8	22 ± 30	0.8 ± 0.6	463 ± 188	11.7 ± 3.5	0
AcTRPM (mg/L)	116 ± 104	250 ± 17	66 ± 10	185 ± 3	182 ± 3	3.0 ± 1
Melatonin (mg/L)	128 ± 25	30	571 ± 33	841 ± 279	1538 ± 530	851 ± 321
Acetate (mM)	366[d]	146.5 ± 30	2.0 ± 0.6	2.0 ± 0.1	1.4 ± 0.2	0.6 ± 0.53
Dry biomass (g/L)	13.7 ± 3.6	27.3 ± 4.1	30 ± 2	42 ± 4	44 ± 0.2	65 ± 2
Growth rate (h^{-1})	0.1	0.1	0.1	0.055	0.055	0.055

[a]Performed under optimized fermentation conditions. [b]Fermentation using glucose only. [c]Error bars indicate standard deviations of replicates. [d]n = 1.

reduced by almost 4 fold to 30 mg/L. Reduction in melatonin titer was probably due to limited metabolic turnover on TrpH-dependent hydroxylation since this step was previously forced to run at a high efficiency for tyrosine synthesis in the Δ*tyrA* HM175 strain. Thus, hydroxylation of tryptophan became rate limiting.

https://dx.doi.org/10.1021/acssynbio.0c00065
ACS Synth. Biol. 2020, 9, 1240−1245

Next, tryptophan hydroxylation was improved by adjusting tryptophan availability and directed evolution of TrpH. First, chromosomal integration of *ddc* was performed in order to (a) improve intracellular tryptophan availability by limiting byproduct formation (in particular AcTRPM), and (b) reduce the cytotoxic effect of *ddc* when expressed at high levels, as reported previously.[3] Additionally, directed evolution of TrpH was attempted using a previously described growth-coupled selection based on the promiscuous phenylalanine hydroxylation activity of TrpH (Figure 3A).[2] Using a tyrosine

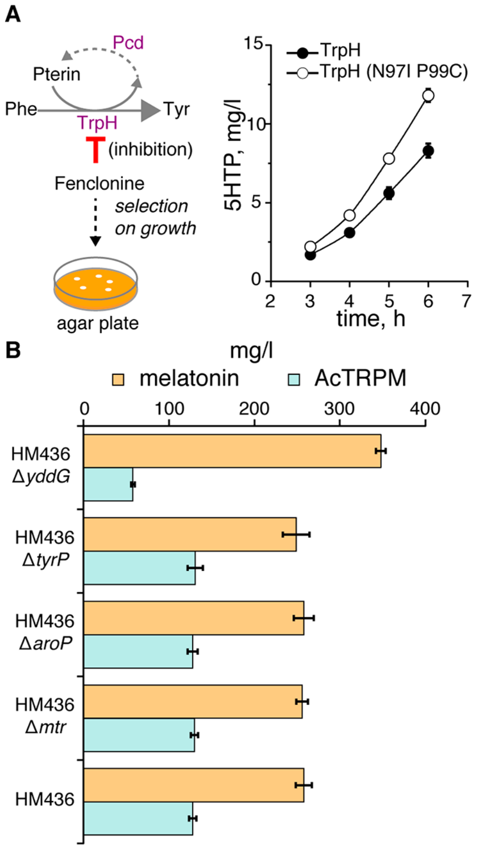

Figure 3. Approaches for improving melatonin production. (A) Directed evolution of TrpH using fenclonine and growth selection. Production rate of 5HTP was compared between wildtype and engineered TrpH in whole cells. (B) Deletion of four aromatic-amino-acid transporters and their impact on melatonin and AcTRPM titer.

auxotrophic host, TrpH variants were tested against fenclonine, a potent inhibitor of phenylalanine hydroxylase.[11] Fenclonine-resistant colonies were subjected to direct measurements on bioproduction of 5HTP from tryptophan. Eleven isolates showed improvements on 5HTP synthesis. Two hot-spot residues, N97 (7 out of 11) and P99 (4 out of 11), were identified. Combinatorial mutagenesis on both residues resulted in a TrpH (N97I P99C) variant that improved the production rate of 5HTP by ~35% in whole cells (Figure 3A). Upon implementing these changes, the third melatonin-producing strain, HM436, was made. Results showed this strain was able to accumulate 30 g/L biomass in 30 h with little acetate formation during fed-batch fermentation (Figure 2C). It produced ~550 mg/L melatonin that was 18-fold ($p < 0.003565$) and 4-fold ($p < 0.000443$) higher than that of HM231 and HM175, respectively (Table 1). The AcTRPM level was also significantly reduced from 260 mg/L observed in HM231 to 60 mg/L ($p < 0.000771$).

Fermentation and Strain Optimization. Next, the fermentation parameters for HM436 were optimized. It was found that ~850 mg/L melatonin could be produced when cell growth was maintained at 0.05 h^{-1} fed with a mixture containing 300 g/L glucose and 7.2 g/L tryptophan and at 30 °C. Under these conditions, cells were able to maintain active growth for 70 h and reached 42 g/L in biomass (Figure 2D). The AcTRPM level was observed at 250 mg/L. A large amount of melatonin-precursor AcHT was detected at ~500 mg/L, suggesting a limitation on Asmt. The Asmt (A258E) variant, a kinetically more active variant,[3] was next recruited. Deletion of *yddG*, a known tryptophan exporter, was also performed due to a beneficial effect observed on melatonin titer and reduction of AcTRPM in small-scale cultivations (Figure 3B). Together, these changes led to the construction of HM626.

Fed-batch fermentations were performed on HM626. When glucose and tryptophan were fed, measured melatonin titer was nearly twice the amount of HM436, reaching ~2000 mg/L and the AcHT level was reduced to a marginally detectable level (Figure 2E and Table 1). The biomass level was comparable to that of HM436. However, high levels of AcTRPM at ~200 mg/L was noted, an issue of concern for downstream applications. Enzyme engineering of Ddc to abolish tryptophan decarboxylation activity was considered but it was reasoned a total prevention of such activity appeared to be challenging due to the striking resemblance of the substrates (tryptophan and 5HTP). Alternatively, limiting tryptophan availability by performing fed-batch fermentation in the absence of tryptophan supplementation and using glucose as the sole carbon source for tryptophan supply was considered to be practical and was attempted. A melatonin titer of ~1000 mg/L was acquired and the levels of byproducts including AcTRPM became negligible. Meanwhile, the biomass level was increased to 65 g/L, a welcomed benefit for enabling SAM-dependent methylation in future development.

CONCLUSIONS

The elusive goal of achieving bioproduction of melatonin at high titers in *E. coli* was accomplished through iterative design−test−learn cycles. In some runs, the final strain achieved a record level of over 2000 mg/L melatonin that represented ~20-fold improvement ($p < 0.026107$) over the initial strain. The same strain also resulted in ~1000 mg/L melatonin using glucose as the sole carbon source for tryptophan supply and negligible levels of byproducts. Thus, this study lays the foundation for the further development of a commercial melatonin-producing *E. coli* strain.

METHODS

Bacteria Strains, Media, and Growth Conditions. The *E. coli* strains used in this study were BW25113 derivatives and were constructed using λ-red recombination.[12] A list of strains used is shown in Table 2. Unless stated otherwise, all strains were maintained at 37 °C in LB (Lennox) (Sigma-Aldrich). Ampicillin and spectinomycin were used at 50 mg/L; chloramphenicol and kanamycin were used at 25 mg/L.

Plasmids. Plasmid DNA assemblies were performed by Gibson Assembly or USER cloning.[13,14] A list of plasmids and their DNA sequences used in this study can be found in Table 2 and the Supporting Information.

https://dx.doi.org/10.1021/acssynbio.0c00065
ACS Synth. Biol. 2020, 9, 1240−1245

Table 2. List of Strains and Plasmids Used[a]

strains	
strains	genotype
BW25113 (wild type)	F-, Δ(araBAD)567, ΔlacZ4787(::rrnB3), Δ(rhaBAD) 568, rph-1, hsdR514
HMP168	BW25113 FolE (T198I) YnbB (V197A) Δ*tnaA* Δ*tyrA* Δ*trpR*
HMP175	HMP168 pHM5 pHM6
HMP258	HMP168 tyrA+ ObgE (E350A) Δ*trpR(ddc)*
HMP436	HMP258 pHM18 pHM39
HMP626	HMP258 Δ*yddG* pHM39 pHM64
plasmids	
plasmids	genotype
pKD46	Para::gam-bet-exo AmR SC101(ts)
pCP20	flp cI857(ts) AmR *Cm*R SC101(ts)
pHM4	Ptrc::trpH ParoF::RBS(recA)::pcd Ptrc::asmt *Cm*R P15A
pHM5	Ptrc::*ddc* Ptrc::*aanat* SmR CDF
pHM6	Ptrc::trpH (E2K) ParoF::RBS(recA)::pcd Ptrc::asmt *Cm*R P15A
pHM18	Ptrc::trpH (E2K N97I P99C) ParoF::RBS(recA)::pcd Ptrc::asmt *Cm*R P15A
pHM39	Ptrc::aanat SmR CDF
pHM64	Ptrc::trpH (E2K N97I P99C) ParoF::RBS(recA)::pcd Ptrc::asmt (A258E) *Cm*R P15A

[a]YnbB (V197A) and ObgE (E350A) were acquired spontaneously during genetic modifications.

TrpH Engineering. A *trpH* random mutagenesis library was prepared using a GeneMorph II Random Mutagenesis Kit (Agilent). An estimate library of 250 000 cells were selected against 2 g/L fenclonine on M9 plates supplemented with 200 mg/L phenylalanine at 37 °C. Two hundred 60 fenclonine-resistant colonies were subjected to direct measurements on bioproduction of 5HTP from tryptophan. Eleven isolates showed improvements on 5HTP synthesis with 25% or more improvements. Two hot-spot residues N97 (7 out of 11) and P99 (4 out of 11) were identified. Combinatorial mutagenesis on N97 and P99 were performed using PCR. Approximately 400 variants were tested for bioproduction of 5HTP.

Small-Scale Bioproduction. Bioproduction from whole cells were performed using a Duetz 96-deepwell system (Enzyscreen) coupled to an Innova 44 shaker (5 cm orbit) (New Brunswick Scientific) in 400 μL of M9 minimal media containing 200 mg/L substrate of interests and antibiotics at 37 °C and 300 rpm. For batch production measurements such as bioprospecting studies, cells in triplicates were grown for 24 h and exometabolites were analyzed as described previously.[3] Time-series measurements were performed essentially the same except the Duetz 24-deepwell system (Enzyscreen) was used and three biological replicates were grown in 2 mL tryptophan-supplemented M9 media. Samples were withdrawn periodically for exometabolites analysis.

Fed-Batch Fermentation. Fed-batch fermentations were performed in 1L fermenters (Sartorius). Per liter of fermentation media was composed of the following: 10 g $(NH_4)SO_4$, 14 g KH_2PO_4, pH 6.0, 20 mL of Stock solution 1 (5 g/L $ZnCl_2$, 7.5 g/L $FeSO_4 \cdot 7H_2O$, 75 g/L sodium citrate), 1 mL Trace elements (2 g/L Al_2 $(SO_4)_3 \cdot 18H_2O$, 0.75 g/L $CoSO_4 \cdot 7H_2O$, 2.5 g/L $CuSO_4 \cdot 5H_2O$, 0.5 g/L H_3BO_3, 24 g/L $MnSO_4 \cdot H_2O$, 3 g/L $Na2MoO_4 \cdot 2H_2O$, 2.5 g/L $NiSO_4 \cdot 6H_2O$, 15 g/L $ZnSO_4 \cdot 7H_2O$), 2 mM $MgSO_4$, 0.1 mM $CaCl_2$, 1 mL vitamin solution (10 mg/L pyridoxin·HCl, 5 mg/L thiamine·HCl, 5 mg/L riboflavin, 5 mg/L nicotinic acid, 5 mg/L phantothenate, 5 mg/L *p*-aminobenzonic acid, 5 mg/L thiotic acid, 2 mg/L biotin, 2 mg/L folic acid and 0.1 mg/L vitamin B12), 0.3 mL/L antifoam 204 (Sigma) and antibiotics. For the batch phase, glucose concentration was 20 g/L and media volume was 0.4 l. Per liter of feed media consisted of 400 g glucose, 18 g $MgSO_4 \cdot 7H_2O$, 14 g KH_2PO_4, 5 g tryptophan, 20 mL Stock solution 1, 0.1 mM $CaCl_2$, 3 mL Trace elements, 3 mL vitamin solution, 1 mL antifoam and antibiotics. All runs were performed at 37 °C, stirring speed was set to 800 rpm, dissolved oxygen was controlled at 40%, and the pH was maintained at 6.0 with 15% $(NH_4)OH$. The feed solution was supplied at a rate to maintain a growth rate between 0.1 to 0.25 h^{-1}. Switch of batch phase to fed-batch phase started automatically when changes in CO_2 off-gas emission dropped below 0.4% and up to 0.4 l feed media was added. For optimized conditions, all runs were performed at 30 °C and cell growth was maintained 0.05 h^{-1} using a feed mixture containing 300 g/L glucose and 7.2 g/L tryptophan. Analysis of the compounds was performed as described previously.[3]

Statistics. Paired two-tailed *t* tests was performed to evaluated statistical significance of the changes in production levels using Microsoft Excel (version 16.35).

■ ASSOCIATED CONTENT

Supporting Information

The Supporting Information is available free of charge at https://pubs.acs.org/doi/10.1021/acssynbio.0c00065.

Supplementary table, supplementary DNA sequences (PDF)

■ AUTHOR INFORMATION

Corresponding Author

Hao Luo − *Novo Nordisk Foundation Center for Biosustainability, Technical University of Denmark, Kongens Lyngby 2800, Denmark*; orcid.org/0000-0001-5703-1033; Email: hluo@biosustain.dtu.dk

Authors

Konstantin Schneider − *Novo Nordisk Foundation Center for Biosustainability, Technical University of Denmark, Kongens Lyngby 2800, Denmark*

Ulla Christensen − *Novo Nordisk Foundation Center for Biosustainability, Technical University of Denmark, Kongens Lyngby 2800, Denmark*

Yang Lei − *Novo Nordisk Foundation Center for Biosustainability, Technical University of Denmark, Kongens Lyngby 2800, Denmark*

Markus Herrgard − *Novo Nordisk Foundation Center for Biosustainability, Technical University of Denmark, Kongens Lyngby 2800, Denmark*

Bernhard Ø. Palsson − *Novo Nordisk Foundation Center for Biosustainability, Technical University of Denmark, Kongens Lyngby 2800, Denmark; Department of Bioengineering and Department of Pediatrics, University of California, San Diego, La Jolla, California 92093, United States*

Complete contact information is available at: https://pubs.acs.org/10.1021/acssynbio.0c00065

Author Contributions

‖Hao Luo and Konstantin Schneider contributed equally to this work.

Notes

The authors declare the following competing financial interest(s): Luo H is an inventor of the patent applications WO2013127914 and WO2013127915.

■ ACKNOWLEDGMENTS

Authors acknowledge financial support from the Novo Nordisk Foundation (NNF10CC1016517). Authors thank Christina Lenhard, Camilla C. Andersen, Svetlana Sherstyk, Charlotte Brøchner, Inger R. Madsen, Mette Kristensen, Milica Randelovic, Christoffer Knudsen, and Hanne B. Christensen and Jochen Förster.

■ ABBREVIATIONS

5HTP, 5-hydroxytryptophan; AcHT, acetyl-5-hydroxytryptamine; TRPM, tryptamine; AcTRPM, acetyl-tryptamine; SAM, S-adenosylmethionine; TrpH, tryptophan hydroxylase; Ddc, aromatic-amino-acid decarboxylase; Aanat, aralkylamine acetyltransferase; Asmt, acetylserotonin *O*-methyltransferase.

■ REFERENCES

(1) Germann, S. M., Baallal Jacobsen, S. A., Schneider, K., Harrison, S. J., Jensen, N. B., Chen, X., Stahlhut, S. G., Borodina, I., Luo, H., Zhu, J., Maury, J., and Förster, J. (2016) Glucose-based microbial production of the hormone melatonin in yeast Saccharomyces cerevisiae. *Biotechnol. J. 11*, 717−24.

(2) Luo, H., Yang, L., Kim, S. H., Wulff, T., Feist, A. M., Herrgard, M., and Palsson, B. Ø. (2020) Directed metabolic pathway evolution enables functional pterin-dependent aromatic-amino-aicd hydroxylation in Escherichia coli. *ACS Synth. Biol. 9*, 494−499.

(3) Luo, H., Hansen, A. S. L., Yang, L., Schneider, K., Kristensen, M., Christensen, U., Christensen, H. B., Du, B., Ozdemir, E., Feist, A. M., Keasling, J. D., Jensen, M. K., Herrgard, M. J., and Palsson, B. O. (2019) Coupling S-adenosylmethionine-dependent methylation to growth: Design and uses. *PLoS Biol. 17*, No. e2007050.

(4) Hara, R., and Kino, K. (2013) Enhanced synthesis of 5- hydroxy-l-tryptophan through tetrahydropterin regeneration. *AMB Express 3*, 70.

(5) Lawley, B., and Pittard, A. J. (1994) Regulation of aroL expression by TyrR protein and Trp repressor in Escherichia coli K-12. *J. Bacteriol. 176*, 6921−30.

(6) Heatwole, V. M., and Somerville, R. L. (1991) The tryptophan-specific permease gene, mtr, is differentially regulated by the tryptophan and tyrosine repressors in Escherichia coli K-12. *J. Bacteriol. 173*, 3601−4.

(7) Roe, A. J., O'Byrne, C., McLaggan, D., and Booth, I. R. (2002) Inhibition of Escherichia coli growth by acetic acid: a problem with methionine biosynthesis and homocysteine toxicity. *Microbiology 148*, 2215−2222.

(8) Han, K., Lim, H. C., and Hong, J. (1992) Acetic acid formation in Escherichia coli fermentation. *Biotechnol. Bioeng. 39*, 663−671.

(9) Kleman, G. L., and Strohl, W. R. (1994) Acetate metabolism by Escherichia coli in high-cell-density fermentation. *Appl. Environ. Microbiol. 60*, 3952−3958.

(10) Keseler, I. M., Mackie, A., Santos-Zavaleta, A., Billington, R., Bonavides-Martínez, C., Caspi, R., Fulcher, C., Gama-Castro, S., Kothari, A., Krummenacker, M., Latendresse, M., Muñiz-Rascado, L., Ong, Q., Paley, S., Peralta-Gil, M., Subhraveti, P., Velázquez-Ramírez, D. A., Weaver, D., Collado-Vides, J., Paulsen, I., and Karp, P. D. (2017) The EcoCyc database: reflecting new knowledge about Escherichia coli K-12. *Nucleic Acids Res. 45*, D543−D550.

(11) Woo, S. L., Gillam, S. S., and Woolf, L. I. (1974) The isolation and properties of phenylalanine hydroxylase from human liver. *Biochem. J. 139*, 741−749.

(12) Datsenko, K. A., and Wanner, B. L. (2000) One-step inactivation of chromosomal genes in Escherichia coli K-12 using PCR products. *Proc. Natl. Acad. Sci. U. S. A. 97*, 6640−6645.

(13) Gibson, D. G., Young, L., Chuang, R. Y., Venter, J. C., Hutchison, C. A., 3rd, and Smith, H. O. (2009) Enzymatic assembly of DNA molecules up to several hundred kilobases. *Nat. Methods 6*, 343−345.

(14) Nour-Eldin, H. H., Geu-Flores, F., and Halkier, B. A. (2010) USER cloning and USER fusion: the ideal cloning techniques for small and big laboratories. *Methods Mol. Biol. 643*, 185−200.
