# Supplementary material for: 5-Hydroxytryptophan (5-HTP): Natural Occurrence, Analysis, Biosynthesis, Biotechnology, Physiology and Toxicology
Source: Int J Mol Sci. 2020 Dec 26;22(1):181. doi: 10.3390/ijms22010181 (PMC7796270; doi:10.3390/ijms22010181)
Supplement: Supplementary file 1 [file ijms-22-00181-s001.zip › 5-HTP.Data/PDF/4006831027/Morgan-2012-Chromatographic-assay-to-study-the.pdf]

View Article Online / Journal Homepage / Table of Contents for this issue
Dynamic Article Links

Analyst

Cite this: *Analyst*, 2012, **137**, 1409

www.rsc.org/analyst

PAPER

Chromatographic assay to study the activity of multiple enzymes involved in the synthesis and metabolism of dopamine and serotonin†

Lindsay D. Morgan, Hannah Baker, Mark S. Yeoman and Bhavik Anil Patel*

Received 7th December 2011, Accepted 24th January 2012
DOI: 10.1039/c2an16227j

Published on 31 January 2012. Downloaded by UNIVERSITÀ DEGLI STUDI DI TORINO on 11/19/2020 5:19:57 PM.

Serotonin and dopamine are crucial regulators of signalling in the peripheral and central nervous systems. We present an *ex-vivo*, isocratic chromatographic method that allows for the measurement of tyrosine, L-3,4-dihydroxyphenylalanine (L-DOPA), dopamine, 3,4-dihydroxyphenylacetic acid (DOPAC), tryptophan, 5-hydroxytryptophan (5-HTP), serotonin and 5-hydroxy-3-indoleacetic acid (5-HIAA) in a model central nervous (CNS) system, to study the role of key enzymes involved in the synthesis and metabolism of serotonin and dopamine. By utilising a sample splitting technique, we could test a single CNS sample at multiple time points under various pharmacological treatments. In, addition, we were able to conduct this assay by utilising the endogenous biochemical components of the CNS to study the synthesis and metabolism of serotonin and dopamine, negating the requirement of additional enzyme activators or stabilisers in the biological matrix. Finally we utilised NSD-1015, an aromatic amino acid decarboxylase enzyme inhibitor used to study the synthesis of dopamine and serotonin to monitor alterations in levels of key neurochemicals. 3-hydroxybenzylhydrazine dihydrochloride (NSD-1015) was able to reduce levels of serotonin and dopamine, whilst elevating precursors L-DOPA and 5-HTP.

Manuscript text

Serotonin (5-HT) and dopamine (DA) have important regulatory roles in the peripheral and central nervous system. They are crucial to a variety of core biological functions, and alterations in the levels of these transmitters have been implicated in a variety of disorders.[1–3] Although the detection of 5-HT and DA is relatively straightforward with currently established analytical methods, very limited simple methods exist for studying the physiological mechanisms that regulate the levels of neurotransmitters.[4,5] Differences in the levels of neurotransmitters are explained by the biochemical steps that are involved in their synthesis, release and metabolism (see Scheme 1). These biochemical steps are conducted by key enzymes. However, current methods utilised to measure these biochemical steps are complex as they require time consuming biochemical assays that measure levels of a single neurotransmitter, amounts of an enzyme involved in a biochemical step or are limited to measure only single time points.[6–10]

5-HT and DA result respectively from the hydroxylation of tryptophan and tyrosine to 5-hydroxytryptamine (5-HTP) and L-3,4-dihydroxyphenylalanine (L-DOPA; Scheme 1). These initial steps are catalysed by the oxygenase enzymes tryptophan and tyrosine hydroxylase and are the rate-limiting step in the synthesis of 5-HT and DA. 5-HTP and L-DOPA are then converted to 5-HT (5-hydroxytryptamine) and DA (3,4-dihydroxyphenylethylamine) respectively *via* a decarboxylation reaction catalysed by L-aromatic amino acid decarboxylase (L-AADC). Metabolism of 5-HT and DA occurs through oxidation to products 5-hydroxyindoleacetic acid (5-HIAA) and 3,4-dihydroxyphenylacetic acid (DOPAC) *via* monoamine oxidase enzymes.

Analytical methods currently exist for monitoring the activity of single enzymatic steps in the pathways described above. These include spectroscopic, fluorescent, electroanalytical and chromatographic approaches.[11–15] Of these approaches chromatography provides the means to monitor multiple neurochemicals and thus multiple enzymatic steps within a single assay.[16–18] However, existing chromatographic approaches are unable to study the activities of multiple enzymes involved in the synthesis and metabolism of more than one neurotransmitter within a single assay. This is because additional coenzymes and cofactors were required to preserve normal enzymatic activity and often interfered with the analytes of interest, limiting the scope of the assay.[11,13,19,20] For this reason chromatographic studies have been limited to investigations of the activity of a single enzymatic step involved in neurotransmitter synthesis[7,10–12,19,20] or limited by the number of neurochemicals that can be monitored.[21,22] These additional exogenous matrix constituents can interfere with the chromatographic analysis in a number of ways. For

Centre for Biomedical and Health Sciences Research, University of Brighton, School of Pharmacy and Biomolecular Sciences, Brighton, BN2 4GJ. E-mail: b.a.patel@brighton.ac.uk; Fax: +44 (0)1273 679333; Tel: +44 (0)1273 642418

† Electronic supplementary information (ESI) available. See DOI: 10.1039/c2an16227j

View Article Online

Published on 31 January 2012. Downloaded by UNIVERSITÀ DEGLI STUDI DI TORINO on 11/19/2020 5:19:57 PM.

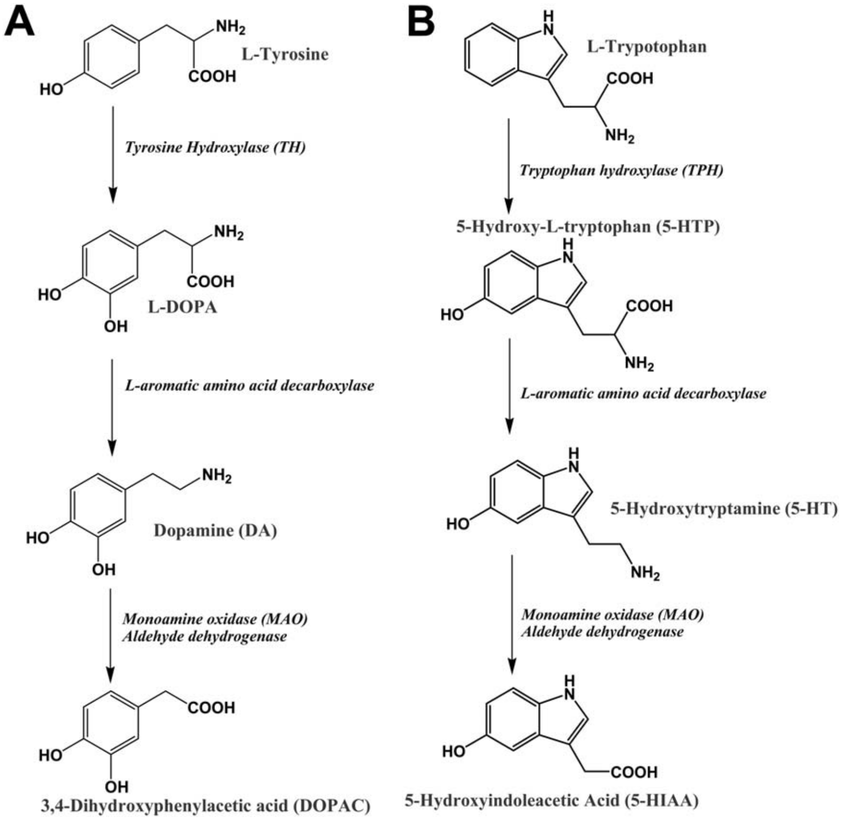

Scheme 1 Biosynthesis pathway for 5-HT and DA, showing all the key enzymes present in the production and metabolism of these two neurotransmitters.

example, ascorbic acid at useful concentrations, masked the L-DOPA peak and therefore other agents were utilised which, although were less effective, did not interfere with the assay.[8,9,11,23] Unfortunately, unlike ascorbic acid, these additives are often not found in useful amounts within a biological system, or are completely alien and thus making the system 'artificial' rather than physiological.

This paper describes a chromatographic assay for the measurement of multiple enzymatic steps involved in the production and metabolism of two key neurotransmitters, DA and 5-HT, using a simple assay buffer with minimal exogenous additions. Analysis of the key neurochemicals was conducted using high performance liquid chromatography with electrochemical detection (HPLC-ED). We show that our developed sample procedure and chromatographic method allow for the means of studying activity from a model central nervous system (CNS) preparation. The method allows for intervention using established pharmacological agents to inhibit key steps in the synthesis and metabolism process and we discuss the ability of the current approach to study multiple enzyme kinetics.

Experimental section

Chemicals

3,4 dihydroxyphenylethylamine (Dopamine, DA), 5-hydroxytryptamine (serotonin, 5-HT), tyrosine, tryptophan, 3,4-dihydroxyphenylacetic acid (DOPAC), 5-hydroxy-3-indoleacetic acid (5-HIAA), 5-hydroxytryptophan (5-HTP), L-3,4-dihydroxyphenylalanine (levodopa, L-DOPA), 3-hydroxybenzylhydrazine dihydrochloride (NSD-1015), p-chlorophenylalanine (pCPA), α-methyl-p-tyrosine (αMPT), citric acid and sodium acetate were obtained from Sigma Aldrich and used as received. All other chemicals were obtained from Sigma and used as received. All solutions were prepared in class A volumetric glassware.

Sample preparation

The methodology for the preparation of the tissue samples was adapted from previous methods.[24,25] The CNS was removed from the pond snail *Lymnaea stagnalis* and pinned out in a silicone elastomer (Sylgard) – lined dish filled with ice-cold 4-(2-hydroxyethyl)-1-piperazineethanesulfonic acid (HEPES)-buffered saline (consisting of 10 mM HEPES, 50 mM NaCl, 1.7 mM KCl, 2 mM $MgCl_2 \cdot 6H_2O$ and 4.0 mM $CaCl_2 \cdot 2H_2O$, buffered to pH 7.9) to facilitate the dissection of the CNS segments. The buccal ganglia and associated lateral and ventral buccal nerves, cerebrobuccal connective and cerebral ganglia were utilized for this assay.[18] This region is well known to be associated with the feeding behaviour of the animal, a behaviour which is quantifiable and has been used by a number of groups to measure age-related neurological changes.[26,27]

 This journal is © The Royal Society of Chemistry 2012

View Article Online

Published on 31 January 2012. Downloaded by UNIVERSITÀ DEGLI STUDI DI TORINO on 11/19/2020 5:19:57 PM.

Assay buffer mixture

Tissue was placed in 50 µl of ice-cold assay buffer (the basic assay buffer mixture consisted of HEPES-buffered saline, 10 µM L-tyrosine, 10 µM L-tryptophan, protease inhibitor cocktail (SIGMA) and PHOSstop phosphatase inhibitor cocktail (Roche)) and manually homogenised on ice using a mortar and Eppendorf until no solid particles were evident (around 20 rotations). A further 60 µl of ice-cold reaction mixture was used to rinse the homogeniser down to maximise the tissue yield and the sample was then divided into either two or three 50 µl aliquots. An equal volume of ice-cold 0.1 M perchloric acid was added to one aliquot to precipitate out proteins in the sample, thereby halting enzymatic reactions. This sample constituted time T_0. To the second sample, either 10 µM NSD-1015, 10 µM pCPA or 10 µM αMPT were added or the sample was simply left at 21 °C for 30 min, before the addition of 0.1 M ice-cold perchloric acid to give the second time point, T_{30}. Immediately after the addition of perchloric acid, samples were centrifuged at 4 °C for 10 min at 14,000 *g* before 80% of the supernatant was removed and filtered through 0.2 µm regenerated cellulose membrane syringe filters (Phenomenex®) to remove any suspended, precipitated solids. Samples were stored on ice prior to injection for analysis.

Chromatography

The HPLC system consisted of a Shimadzu pump and Rheodyne manual injector equipped with a 20 µl loop. The KINETIC® ODS 2.6µm 100 mm × 2.1 mm i.d. analytical column with a KrudKatcher™ Ultra in-line filter (Phenomenex®, Macclesfield, UK) was used. The HPLC system was used in isocratic mode at ambient temperature for the determination of neurotransmitters, run with a 150 µl min^{-1} flow rate and an injected sample volume of 20 µl. CHI 1001A (CH Instruments, Austin, TX, USA) was used to control the detector voltage and record the current. A 3 mm glassy carbon electrode (Radial flow cell, Bioanalytical Systems Inc.) served as the working electrode and was used with a Ag|AgCl reference electrode and a stainless steel auxiliary block as the counter electrode. The working electrode was set at a potential of +850 mV *versus* Ag|AgCl reference electrode. Control and data collection/processing were handled through CHI 1001A software.

The stock buffer for the mobile phase comprised the following: 0.1 M sodium acetate, 0.1 M citric acid and 27 µM disodium ethylene-diamine-tetra-acetate (EDTA) dissolved in 1 l of deionized, distilled water and buffered to pH 3.5 using concentrated sodium hydroxide. To prepare the mobile phase, the acetate buffer was mixed with methanol (HiperSOLV® for HPLC, BDH) in the ratio 95 : 5 (v/v) and degassed by sonication after mixing.

Standards and calibration

Standard solutions were prepared from a 500 µM stock standard of each analyte prepared in 0.1 M perchloric acid. Each of the standard solutions were prepared on the day of analysis and stored at 4 °C between injections. A calibration range was obtained by running, individually, a range of concentrations of 5-HIAA, tryptophan, tyrosine, 5-HT, DA, DOPAC, L-DOPA and 5-HT through the developed chromatographic method. Concentrations investigated ranged from 0.01 to 5 µM for L-DOPA, 5-HTP, DOPAC, and 5-HIAA. For 5-HT and DA the range was 0.1 to 5 µM and for tryptophan and tyrosine, calibrations were carried out between 1–100 µM.

Data analysis and interpretation

A mixture of all the standards was run freshly each day to ensure the retention times remained constant and samples were analysed against this standard. The peak areas of each of the neurochemicals from the various treatments were measured and their concentrations determined from calibration plots for each analyte. The concentration of each analyte was then normalised to the amount of protein in that sample, determined using the Bradford assay.[28] For analysis, the ratio of normalised responses at T_0, T_{30} and T_{30} in the presence of a pharmacological agent were compared using non-parametric tests. Data was presented as mean ± St.dev.

Results and discussion

Chromatography method development

A potential of +850 mV *vs.* Ag|AgCl reference electrode was chosen as the detection potential for the chromatographic assay based on quasi-steady state hydrodynamic voltammograms (see Supplementary Figure 1†). Many studies do not characterise the optimal potential for detection of analytes and in some cases lower potentials have previously been used.[29] In both cases the use of a potential where the diffusion limited current for all compounds is not obtained can lead to poor precision and accuracy especially where, as in the case for carbon electrodes, the heterogeneous rate constant can be influenced by adsorption, and the current response subsequently altered.

Chromatographic responses are shown in Fig. 1, where all the analytes were separated using an isocratic mode. Based on responses in Fig. 1A, 5% methanol was utilised in the final mobile phase to allow sufficient separation of DA and tyrosine. As flow rate correlated linearly with retention time (Fig. 1B), a flow rate of 150 µl min^{-1} was utilised. This flow rate is fit for purpose but could be increased to reduce the overall run time; however we were limited due to the capabilities of our pump. The chromatographic assay is similar to other methods; however, we are able to show separation of all the key neurochemicals in an isocratic mode instead of the more complex gradient elution mode.[13,30] No interference was observed from other key neurochemicals such as norepinephrine (NE) and homovanillic acid (HVA). An internal standard was not utilised for the assay, as exogenous tryptophan and tyrosine were used as retention time markers. All separations were obtained with a capacity factor of less than 5 (longest retention time of approximately 17 min).

Retention times of L-DOPA, tyrosine, DA, 5-HTP, 5-HT, DOPA, tryptophan and 5-HIAA were 2.1, 2.6, 2.8, 4.3, 6.3, 8.1, 10.9 and 16.6 min respectively and the chromatograph is shown in Fig. 1C. The number of theoretical plates (N) for the separation was high, with the greatest value obtained for 5-HIAA (12197 plates) and the lowest for the elution of tryptophan (6605 plates). Minimal peak tailing was observed for the entire

View Article Online

Published on 31 January 2012. Downloaded by UNIVERSITÀ DEGLI STUDI DI TORINO on 11/19/2020 5:19:57 PM.

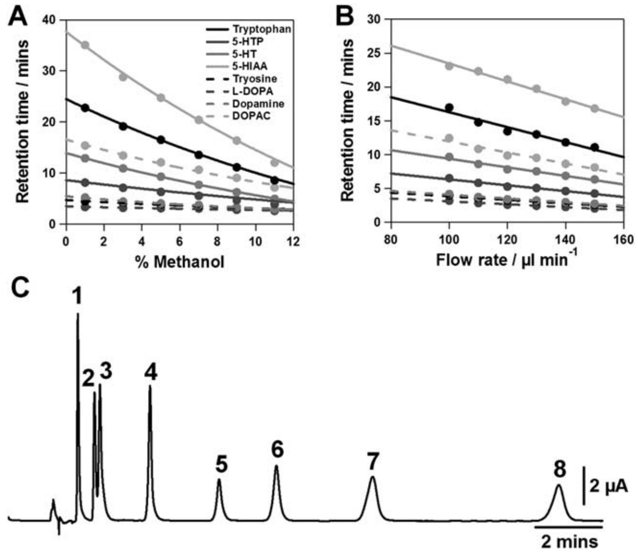

Fig. 1 Chromatographic method parameters. (A) The influence of increasing methanol content on retention time when the flow rate is 100 µL min^{-1}. (B) The changes in retention time with increases in flow rate at 5% methanol. (C) A chromatograph of a standard mixture at 150 µL min^{-1} flow rate using 5% methanol, 95% 0.1 M acetate buffer. **Standards:** 1 – L-DOPA (10 µM); 2 – tyrosine (100 µM); 3 – DA (10 µM); 4 – 5-HTP (10 µM); 5 – 5-HT (10 µM); 6 – DOPAC (10 µM); 7 – tryptophan (100 µM) and 8 – 5-HIAA (10 µM).

separation (values within 0.8 and 1.3), except for L-DOPA and 5-HTP, where responses were 1.52 and 1.35 respectively. Baseline resolution was observed for the separation (R_s < 3.0) for all components except for DA and tyrosine, where R_s was 0.45. Although this value is lower than that which is normally considered adequate, peak responses were easily identified for both analytes. Using a mobile phase consisting of 95% 0.1 M acetate buffer and 5% methanol, all major analytes were separated within 17 min. Previous HPLC assays that monitor enzymatic activity have run times from 6 to 12 min but only monitor one neurotransmitter.[9,12,19,20]

When the sample mixture was repeatedly injected, the reproducibility of chromatographic peak areas were within 2% (n = 15, data not shown) suggestive that the electrode was not influenced by electrode fouling. Supplementary Table 1 shows the calibration range of the analyte of choice, the limit of detection (LOD, based on the 3 standard deviations of the y intercept using least-squares regression) and the correlation coefficient (R^2).† The metabolites and precursor molecules are present in varying concentrations within the CNS of *Lymnaea* and therefore the concentration range was based on previous methods.[18] The limits of detection for L-DOPA, tyrosine, DA, 5-HTP, 5-HT, DOPAC, tryptophan and 5-HIAA were 24 nM, 1.12 µM, 67 nM, 20 nM, 98 nM, 35 nM, 1.24 µM and 29 nM respectively. These limits of detection for metabolites are similar to a previous method that was utilised for monitoring levels of neurochemicals from brain homogenates.[31–33] The limits of detection are also significantly higher for tyrosine and tryptophan than all other components; however for this assay these components are usually added in excess in order to study enzymatic activity and so the higher limit of detection is not prohibitive.

Overall this short simple method provides a clear analysis of the key neurochemicals involved in the synthesis and metabolism of DA and 5-HT and thus provides a means to study the enzymatic activity within the pathways of both key neurotransmitters using a single assay.

Influence of buffer constituents on studying enzymatic activity

The major goal of this work is the ability to study the activity of the enzymes integral to the biosynthesis of both 5-HT and DA in a single assay. Previous studies have examined the enzymatic output of tyrosine and tryptophan hydroxylase; however these methods, which focused on monitoring a single neurotransmitter system, required a complicated assay buffer in order to maximise the output of the enzymes and also utilised a variety of post-assay sample preparation steps to observe peaks of the key analytes.[6,8,9,19] All of these features made these assays laborious, complicated and far removed from the endogenous biological conditions. We have utilised a simple sample preparation method that allows such measurements to be made without interference from any assay buffer constituents on the chromatogram or require the addition of enzymatic stabilisers to the mixture and elevate activity.

In other assay buffers, three types of agents are commonly added: 1) an anti-oxidant such as catalase, 2) reducing agents such as mercaptoethanol (BME) or dithothreitol (DTT) and 3) enzyme co-factors such as tetrahydrobiopterin (BH_4).[11] However these agents interfere with the separation of analytes of interest (*e.g.* ascorbic acid interfering with L-DOPA[11,12]). We, however, observed no interference from ascorbic acid with the responses of all analytes during chromatographic analysis (see Fig. 2A). This is an advantage as, compared to BME or DTT, ascorbic acid induces the highest activity of tyrosine hydroxylase and also acts as an antioxidant, preserving some of the more fragile downstream neurochemicals from oxidation.[11] Furthermore, ascorbic acid is also endogenous to the CNS and present in large amounts, negating the need to add an exogenous supply. Both catalase and BH_4 are also frequently added to assay buffers. As our system contains endogenous ascorbate and will also contain an amount of endogenous BH_4. We investigated whether the addition of catalase and BH_4 would a) interfere with any peaks of interest and b) was required to improve the activity of the enzyme. Fig. 2A shows chromatographic responses of 1mg ml^{-1} catalase and 1 µM BH_4. Catalase and BH_4 have retention times of 2.1 and 2.5 min respectively. The peaks of catalase and BH_4 overlap and therefore interfere with responses of DA and tyrosine in our assay. Similar responses have been observed by other researchers.[9,12,19] When catalase and BH_4 were introduced to the assay buffer along with a CNS homogenate, only the serotonergic components could be analysed due to the masking of the dopaminergic analytes. No noticeable differences were observed between the chromatograms produced at T_{30}, in the presence or absence of catalase and BH_4 (data not shown). Therefore, we have chosen to use the endogenous ascorbic acid present within the CNS of *Lymnaea* to act as the key antioxidant within the assay buffer to maximise the activity of the enzymes, and to rely upon the endogenous activators and co-factors contained within the CNS homogenate to promote and stabilise the reaction.

 This journal is © The Royal Society of Chemistry 2012

View Article Online
Published on 31 January 2012. Downloaded by UNIVERSITÀ DEGLI STUDI DI TORINO on 11/19/2020 5:19:57 PM.

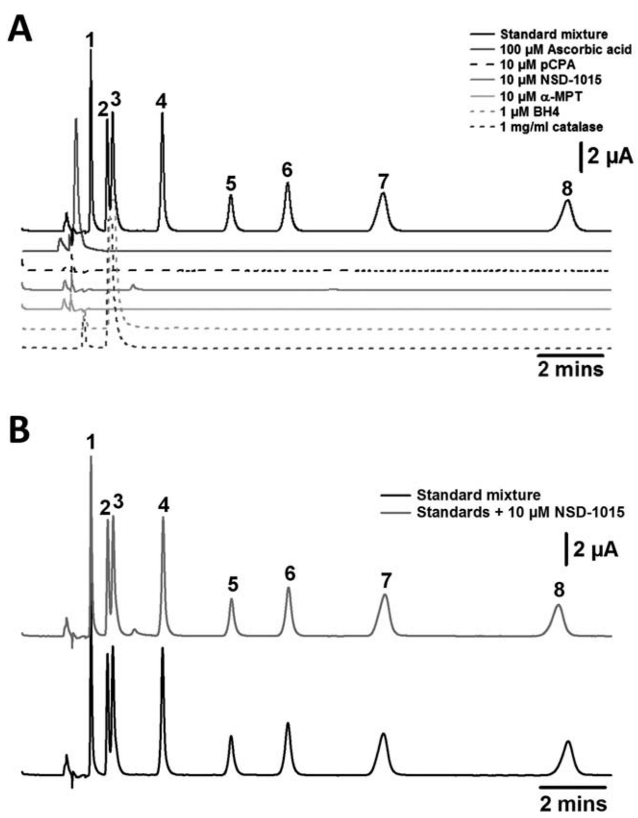

Fig. 2 In (A) the influence of assay buffer constituents on chromatographic separation of all neurochemicals. A standard mixture of all eight components is shown with responses of individual injections of enzymatic activators (100 μM ascorbic acid, 1 μM BH_4 and 1mg ml^{-1} catalase) and inhibitors (NSD-1015, pCPA and αMPT; all 10μM). In (B) chromatographic responses of standards in the presence and absence of 10 μM NSD-1015 in assay buffer. Chromatographic conditions as in Fig. 1C. **Standards**: 1 – L-DOPA (10 μM); 2 – tyrosine (100 μM); 3 – DA (10 μM); 4 – 5-HTP (10 μM); 5 – 5-HT (10 μM); 6 – DOPAC (10 μM); 7 – tryptophan (100 μM) and 8 – 5-HIAA (10 μM). Chromatographic signals were not seen for either 10 μM pCPA or 10 μM αMPT. A signal was detected for 10 μM NSD-1015, but this did not occur at a retention time that would inhibit the observation of any of the analytes of interest.

Another potential limitation of such assays is the interference of pharmacological agents utilised to study the activity of relevant enzymes. Fig. 2 shows chromatographic responses from i) NSD-1015, a L-AADC inhibitor, ii) pCPA, a tryptophan hydroxylase inhibitor, and iii) αMPT, a specific tyrosine hydroxylase inhibitor. From these components only NSD-1015 produced a peak on the chromatogram with a retention time of 3.4 min. At the concentrations tested, the peak produced by NSD-1015 does not overlap those of the neurochemicals of interest, allowing us to study the two, key, rate-limiting enzymatic steps involved in the formation of 5-HT and DA (Scheme 1). Also these components may interfere with the levels of these neurochemicals. As shown in Fig. 2B, there is no difference in the chromatographic responses of all analytes with or without the presence of the assay buffer constituents and 10 μM NSD-1015.

To summarise, we have been able to use the natural biochemical matrix of the CNS homogenate, containing endogenous ascorbic acid and all the necessary co-factors to study the role of enzymes responsible for the formation of 5-HT and DA. At the concentrations used, the enzyme inhibitors did not interfere with the chromatogram nor did they significantly alter the levels of neurochemical standards. This method is advantageous in that it provides the endogenous matrix components are sufficient to stabilise and study CNS enzymes.

Investigation of enzymatic activity under *ex vivo* conditions

To determine whether enzymatic activity could be monitored from a single CNS homogenate, the feasibility of splitting a sample into identical aliquots to allow for sampling at multiple time points was studied. No differences in the amount of neurochemicals were observed from either split following normalisation to protein content (see Supplementary Figure 2†). Sample splitting of the cerebro-buccal sample allowed us to study neurochemical levels at T_0 and T_{30} in the absence of any pharmacological agents (Fig. 3). Changes in the levels of neurochemicals were used to gain some understanding of the enzymatic steps that were active in the synthesis and metabolism of 5-HT and DA.

All neurochemicals were observed in the cerebro-buccal sample at T_0, with 98.7 nM L-DOPA, 10.0 μM tyrosine, 82.0 nM DA, 23.2 nM 5-HTP, 650.9 nM 5-HT, 42.9 nM DOPAC, 9.9 μM tryptophan and 74.7 nM 5-HIAA being detected (Fig. 3). These levels were similar to other studies from invertebrates.[18,34] Previous studies detailing the distribution and morphology of various neurons in the *Lymnaea* CNS showed the cerebro-buccal region to be rich in serotonergic neurons, with a small number of dopaminergic cells.[35,36] This was reflected in the concentrations of 5-HT and DA observed in the current study, with around 6 times more 5-HT being present than DA. Enzymatic activity was investigated by studying changes in the levels of neurochemicals between T_0 and T_{30}. After 30 min there was a decrease in tryptophan (loss of 5.0 ± 3.58% compared to T_0) and tyrosine (loss of 6.1 ± 4.9% compared to T_0). Both tyrosine and tryptophan were used to spike the samples so the decreases in the amounts are small due to both sample losses and enzymatic activity. Levels of DA and 5-HTP also decreased after 30 mins. Increased levels of L-DOPA, 5-HT, DOPAC and 5-HIAA were observed at T_{30}

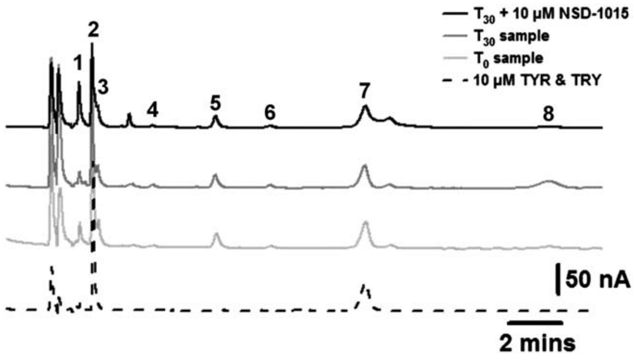

Fig. 3 Chromatographs from CNS tissue measurements. Responses are shown for (i) assay buffer containing 10 μM tyrosine and tryptophan, which was used to spike CNS samples, (ii) T_0 sample, (iii) T_{30} sample and (iv) T_{30} sample in the presence of 10 μM NSD-1015. Chromatographic conditions as in Fig. 1C. **Solutes**: 1 – L-DOPA; 2 – tyrosine; 3 – DA; 4 – 5-HTP; 5 – 5-HT; 6 – DOPAC; 7 – tryptophan and 8 – 5-HIAA.

View Article Online

Published on 31 January 2012. Downloaded by UNIVERSITÀ DEGLI STUDI DI TORINO on 11/19/2020 5:19:57 PM.

compared to T_0. The greatest increases were observed for L-DOPA (which increased by 61.1 ± 10.1%) and 5-HIAA (which increased by 105 ± 11.3%; Fig. 4). High levels of 5-HIAA at T_{30} suggest a rapid conversion of tryptophan to 5-HT and then its metabolism to 5-HIAA. DA synthesis was however much slower with high levels of L-DOPA observed at T_{30} and a decrease in DA levels, all enzymes within the serotonergic pathway must have greater efficiency compared to the dopamine pathway under these conditions, whereas greater levels of L-DOPA are generated and the post enzymatic reactions are slower. Additionally, buffer conditions (pH, final concentration of co-factors) may confer favour to elements of the serotonergic pathway relative to that of the dopamine.

Influence of L-AADC inhibitor on neurochemical levels

In Fig. 4, enzymatic activity is investigated using the L-AADC inhibitor NSD-1015, which blocks the conversion of L-DOPA to DA and 5-HTP to 5-HT, thereby preserving levels of L-DOPA and 5HTP and allowing the activity of the enzymes tyrosine and tryptophan hydroxylase, to be investigated. The cerebro-buccal homogenate was split into 3 samples and each spiked with 10 μM of tyrosine and tryptophan to ensure that the system was saturated with substrate. Chromatograms were obtained for T_{30} samples in the presence and absence of 10 μM NSD-1015 and these were compared to T_0 (Fig. 3). The ratio of normalised responses is shown in Fig. 4, where the ratio of T_{30}:T_0 for the neurochemical of interest is shown for samples untreated or treated with either 10 or 30 μM NSD-1015.

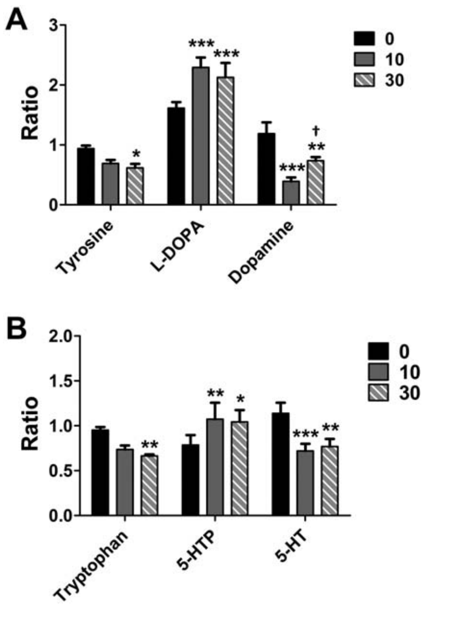

Fig. 4 Responses showing the influence of the L-aromatic decarboxylase inhibitor NSD-1015 on the levels of the key analytes. Responses of key analytes influenced in the (A) dopaminergic and (B) serotoninergic systems are shown. Where (0) samples are untreated responses taken at T_{30} and treated responses are T_{30} samples in the presence of 10 or 30 μM NSD-1015. Responses shown as mean ± St.Dev., n = 3, *p < 0.05, **p < 0.01 or ***p < 0.001 using a one-way ANOVA with a post-hoc Tukey test. Where * shows differences between samples treated with and without NSD and † shows differences between 10 μM and 30 μM NSD-1015.

For the dopaminergic system, L-DOPA levels significantly increased in the presence of both 10 and 30 μM NSD-1015 ($p < 0.001$, $n = 3$, Fig. 4A). There was no difference in the response between 10 and 30 μM NSD-1015. These increases in L-DOPA resulted in significant decreases in DA levels. DA levels were significantly diminished ($p < 0.001$, $n = 3$) by 66.7 ± 5.0% between controls responses and those in the presence of 10 μM. Levels of DA decreased also in the presence of 30 μM NSD-1015 ($p < 0.01$, $n = 3$, Fig. 4A). Collectively these data demonstrate that the pharmacological inhibitor NSD-1015 was able to block the L-AADC in the dopaminergic system.

As the same enzyme is involved at the equivalent step in the serotonergic system, NSD-1015 should prevent the formation of 5-HT from 5-HTP. As observed from Fig. 4B, this also is the case. 5-HTP levels significantly increased by 40.7 ± 44.3% ($p < 0.01$, $n = 3$) in the presence and 10 μM NSD-1015, and was also significant higher in the presence of 30 μM of the inhibitor ($p < 0.05$, $n = 3$). Significant decreases in the levels of 5-HT were also observed in the presence of 10 μM ($p < 0.001$, $n = 3$) and 30 μM NSD-1015 ($p < 0.01$, $n = 3$, Fig. 4B).

The influence of the inhibitor was greater for the doampinergic system compared to the serotoninergic system. This was surprising, considering that the L-AADC enzyme responsible for controlling both reactions is the same, however 5-HTP and L-DOPA may have different substrate specificities for the enzyme and therefore the rate of conversion to 5-HT and DA respectively may not be comparable. Overall, the addition of the inhibitor to facilitate blockade of a particular enzymatic step was clearly reflected within the levels of the neurochemicals observed and did not require the addition of supplementary assay buffer constituents as other methods have required.[6,8,9,12,37]

Conclusions

We have developed an efficient method for the analysis of the neurochemicals involved in the production and metabolism of 5-HT and DA utilising a simple chromatographic assay and an easy-to-prepare assay buffer mixture. This combination allows for basic sample preparation steps to be used for studying enzymes in biological systems. The sample splitting approach provides a means to study the enzymatic activity at varying time points in the absence or presence of pharmacological intervention. The assay was able to study the influence of a pharmacological agent designed to inhibit a specific enzymatic step without the addition of further agents to the assay buffer. As well as the direct quantification of a range of neurochemicals, this approach has the potential for application in the study of discrete enzyme activity in these two neurotransmitter systems.

Acknowledgements

The authors would like to thank Roger Clayton for animal husbandry. BAP and MSY would like acknowledge support from BBRSC grant (BB/G015147/1).

References

1 M. G. Berger, J.A. and B. L. Roth, *Annu. Rev. Med.*, 2009, **60**, 355–366.
2 S. D. Iversen and L. L. Iversen, *Trends Neurosci.*, 2007, **30**, 188–193.

 This journal is © The Royal Society of Chemistry 2012

View Article Online

Published on 31 January 2012. Downloaded by UNIVERSITÀ DEGLI STUDI DI TORINO on 11/19/2020 5:19:57 PM.

3 *Handbook of the Behavioural Neurobiology of Serotonin (Handbook of Behavioural Neuroscience)*. Elsevier Science and Technology, 2009.
4 B. P. Jackson, S. M. Dietz and R. M. Wightman, *Anal. Chem.*, 1995, **67**, 1115–1121.
5 P. S. Cahill, Q. D. Walker, J. M. Finnegan, G. E. Mickelson, E. R. Travis and R. M. Wightman, *Anal. Chem.*, 1996, **68**, 3180–3186.
6 T. Kato, S. Horiuchi, A. Togari and T. Nagatsu, *Experientia*, 1981, **37**, 809–811.
7 D. Boonyarat, K. Kojima and T. Nagatsu, *J. Chromatogr., Biomed. Appl.*, 1983, **274**, 331–336.
8 Y. Hirata, A. Togari and T. Nagatsu, *J. Neurochem.*, 1983, **40**, 1585–1589.
9 M. Naoi, T. Takahashi and T. Nagatsu, *J. Chromatogr., Biomed. Appl.*, 1988, **427**, 229–238.
10 J. Trocewicz, N. Kato, K. Oka and T. Nagatsu, *J. Chromatogr.*, 1982, **223**, 328–333.
11 T. Nagatsu, *J. Chromatogr., Biomed. Appl.*, 1991, **566**, 287–307.
12 T. Nagatsu, K. Oka and T. Kato, *J. Chromatogr., Biomed. Appl.*, 1979, **163**, 247–252.
13 A. T. Wood and M. R. Hall, *J. Chromatogr., Biomed. Appl.*, 2000, **744**, 221–225.
14 S. Bohm, D. Pijanowska, W. Olthuis and P. Bergveld, *Biosens. Bioelectron.*, 2001, **16**, 391–397.
15 J. Wang, Y. Jiang, C. Zhou and X. Fang, *Anal. Chem.*, 2005, **77**, 3542–3546.
16 F.-C. Cheng and J.-S. Kuo, *J. Chromatogr., Biomed. Appl.*, 1995, **665**, 1–13.
17 T. Huang, R. E. Shoup and P. T. Kissinger, *Current Separations*, 1990, **9**, 139–143.
18 B. A. Patel, M. Arundell, K. H. Parker, M. S. Yeoman and D. O'Hare, *J. Chromatogr., B: Anal. Technol. Biomed. Life Sci.*, 2005, **818**, 269–276.
19 C. LeRoy Blank and R. Pike, *Life Sci.*, 1976, **18**, 859–865.
20 J. Olšovská, J. Novotná, M. Flieger and J. Spížek, *Biomed. Chromatogr.*, 2007, **21**, 1252–1258.
21 M. Le Masurier, W. Oldenzeil, C. Lehman, P. Cowen and T. Sharp, *Neuropsychopharmacology*, 2005, **31**, 310–317.
22 D.-K. Kim, T. J. Tolliver, S.-J. Huang, B. J. Martin, A. M. Andrews, C. Wichems, A. Holmes, K.-P. Lesch and D. L. Murphy, *Neuropharmacology*, 2005, **49**, 798–810.
23 T. Nagatsu, *J. Chromatogr., Biomed. Appl.*, 1991, **566**, 287–307.
24 B. A. Patel, M. Arundell, K. H. Parker, M. S. Yeoman and D. O'Hare, *Phys. Chem. Chem. Phys.*, 2010, **12**, 10065–10072.
25 M. S. Yeoman, A. W. Pieneman, G. P. Ferguson, A. T. Maat and P. R. Benjamin, *Journal of Neurophysiology*, 1994a, **72**, 1357–1371.
26 M. Arundell, B. A. Patel, V. Straub, M. C. Allen, C. Janse, D. O'Hare, K. Parker, P. R. Gard and M. S. Yeoman, *Neurobiol. Aging*, 2006, **27**, 1880–1891.
27 B. A. Patel, M. Arundell, M. C. Allen, P. Gard, D. O'Hare, K. Parker and M. S. Yeoman, *Neurobiol. Aging*, 2006, **27**, 1892–1901.
28 M. M. Bradford, *Anal. Biochem.*, 1976, **72**, 248–254.
29 U. Kumar, A. Baragli and R. C. Patel, *TrAC, Trends Anal. Chem.*, 2003, **22**, 537–543.
30 B. H. C. Westerink, *J. Chromatogr., Biomed. Appl.*, 2000, **747**, 21–32.
31 G. M. Anderson, J. G. Young, D. K. Batter, S. N. Young, D. J. Cohen and B. A. Shaywitz, *J. Chromatogr., Biomed. Appl.*, 1981, **223**, 315–320.
32 G. S. Mayer and R. E. Shoup, *J. Chromatogr., A*, 1983, **255**, 533–544.
33 J. Wagner, P. Vitali, M. G. Palfreyman, M. Zraika and S. Huot, *J. Neurochem.*, 1982, **38**, 1241–1254.
34 M. Yeoman, in *Age*, Editon edn, 2007, vol. 29, pp. 111–112.
35 K. Elekes, G. Kemenes, L. Hiripi, M. Geffard and P. R. Benjamin, *J. Comp. Neurol.*, 1991, **307**, 214–224.
36 Kemenes, K. Elekes, L. Hiripi and P. R. Benjamin, *J. Neurocytol.*, 1989, **18**, 193–208.
37 G. W. Bennett, M. P. Brazell and C. A. Marsden, *Life Sci.*, 1981, **29**, 1001–1007.
