# Supplementary material for: 5-Hydroxytryptophan (5-HTP): Natural Occurrence, Analysis, Biosynthesis, Biotechnology, Physiology and Toxicology
Source: Int J Mol Sci. 2020 Dec 26;22(1):181. doi: 10.3390/ijms22010181 (PMC7796270; doi:10.3390/ijms22010181)
Supplement: Supplementary file 1 [file ijms-22-00181-s001.zip › 5-HTP.Data/PDF/4012388103/1-s2.0-S0165614718300567-main.pdf]

Trends in Pharmacological Sciences

Review

Peripheral Serotonin Synthesis as a New Drug Target

Susann Matthes[1,2] and Michael Bader[1,2,3,4,5,*]

The first step in serotonin (5-HT) biosynthesis is catalyzed by tryptophan hydroxylase (TPH). There are two independent sources of the monoamine that have distinct functions: first, the TPH1-expressing enterochromaffin cells (ECs) of the gut; second, TPH2-expressing serotonergic neurons. TPH1-deficient mice revealed that peripheral 5-HT plays important roles in platelet function and in inflammatory and fibrotic diseases of gut, pancreas, lung, and liver. Therefore, TPH inhibitors were developed which cannot pass the blood–brain barrier to specifically block peripheral 5-HT synthesis. They showed therapeutic efficacy in several rodent disease models, and telotristat ethyl is the first TPH inhibitor to be approved for the treatment of carcinoid syndrome. We review this development and discuss further therapeutic options for these compounds.

Highlights

5-HT is synthesized by two tryptophan hydroxylases, TPH1 in ECs of the gut and TPH2 in hindbrain raphe nuclei. EC-derived 5-HT is transported by circulating platelets and released upon their activation. Moreover, there is local TPH1 and 5-HT synthesis in lung, pancreatic β cells, and adipocytes, as well as local TPH2-mediated 5-HT synthesis in enteric neurons.

TPH1-derived 5-HT causes the gastrointestinal symptoms associated with carcinoid tumors (carcinoid syndrome) and is thought to be involved in several other disorders such as pulmonary hypertension, inflammatory and fibrotic diseases, thrombosis, and obesity. Its effects on osteoporosis are controversial.

Inhibition of peripheral 5-HT synthesis is a novel therapeutic strategy for these diseases. TPH2-mediated 5-HT synthesis in the brain should be spared because its inhibition would cause adverse effects such as depression.

Telotristat ethyl is the first TPH inhibitor with FDA approval for the treatment of carcinoid syndrome. This and other compounds of this novel drug class need to prove their efficacy for the treatment of other diseases in future preclinical and clinical studies.

The clinical use of telotristat ethyl and other non-selective TPH inhibitors will clarify whether their exclusion from the brain is sufficient to avoid adverse central nervous system effects, or if it will be necessary to develop novel compounds which do not inhibit TPH2. The first such TPH1-selective compounds were recently discovered, but their allosteric mechanisms of action and efficacy remain to be further validated.

The 5-HT System Defined by TPH1 and TPH2

Initially discovered as a vasoconstricting substance in serum [1], the biogenic monoamine serotonin, **5-hydroxytryptamine** (5-HT; see Glossary) is now known to be involved in a broad spectrum of physiological processes including behavior, vascular function, hemostasis, liver regeneration, gut motility, insulin secretion, erythropoiesis, mammary gland plasticity, adipocyte differentiation, immune responses, fibrosis, and heart and brain development [2–9]. For most of these functions the source of 5-HT in the brain is the raphe nuclei in the hindbrain, whereas in the periphery 5-HT is mainly synthesized in ECs in the intestine and distributed by platelets in the circulation (Box 1). However, local 5-HT generation in enteric neurons, fat, lung, and pancreas has been described and may contribute to its local actions.

The serotonergic signaling system in vertebrates is very complex and is composed of multiple transporters (**serotonin transporter**, SERT; vesicular monoamine transporter, VMAT) and seven families of 14 receptor subtypes (5-HT_{1-7}) [10]. 5-HT biosynthesis and metabolism are described in Box 1 and Figure 1. The initial and rate-limiting step in the biosynthesis of 5-HT is catalyzed by **tryptophan hydroxylase** (TPH). Together with phenylalanine hydroxylase and tyrosine hydroxylase, TPH constitutes the third member of the highly conserved superfamily of iron- and tetrahydrobiopterin (BH4)-dependent aromatic amino acid hydroxylases (AAAHs) [11]. Although knockout of the tyrosine hydroxylase gene resulted in an embryonic lethal phenotype [12], genetic ablation of what was then known as TPH (now TPH1) unraveled the existence of a second isoform (TPH2), exposed by the finding of normal 5-HT levels in the brain of *Tph1*$^{-/-}$ mice [13]. This newly found duality of the 5-HT system with two independently generated pools of 5-HT – one in the brain and another in the blood – was once more confirmed when targeted deletion of *Tph2* reduced brain 5-HT to below 1% without changing circulating 5-HT levels [14–16]. Interestingly, homozygous *Tph1*$^{-/-}$ mice are alive, healthy, and fertile, but 5-HT concentration in blood remains ~10% of the amount reported in wild-type mice. These

 https://doi.org/10.1016/j.tips.2018.03.004
© 2018 Elsevier Ltd. All rights reserved.

CellPress REVIEWS

Box 1. Metabolism of 5-HT

The first and rate-limiting step in 5-HT biosynthesis is performed by tryptophan hydroxylase (TPH) enzymes that catalyze the conversion of the essential amino acid L-tryptophan (Trp) to 5-hydroxytryptophan (5-HTP) in the presence of molecular oxygen and the cofactor (6R)-L-erythro-5,6,7,8-tetrahydrobiopterin (BH4). There are two isoforms of this enzyme: TPH1, that is expressed mainly in ECs in the gut, in the pineal gland, and in some other cell types in the periphery, and TPH2 that is nearly exclusively expressed in neurons of the hindbrain raphe nucleus and the enteric nervous system. 5-HTP is further converted to 5-HT by the ubiquitously expressed enzyme aromatic amino acid decarboxylase (AADC). In most cells synthesizing 5-HT, it is subsequently stored in vesicles by the vesicular monoamine transporters 1 (VMAT1, SLC18A1) or 2 (VMAT2, SLC18A2) for subsequent release upon activation of the cell. ECs can be induced mechanically or chemically to release the monoamine into the extracellular space, from where it can either reach the gut lumen or the bloodstream, or directly activate adjacent enteric neurons. In the bloodstream, 5-HT is very effectively taken up via the 5-HT transporter (SERT, SLC6A4) by platelets passing through the intestinal vessels, and is again stored in their dense granules by VMAT2 for later release upon activation. 5-HT in the blood, which is not rescued from degradation by platelets, is taken up by SERT or other transporters into cells of the intestine, liver, and lung. There it cannot be stored in vesicles and is degraded to 5-hydroxyindole acetic acid (5-HIAA), mainly by monoamine oxidase A (MAO-A) on mitochondria and aldehyde dehydrogenase (ALDH). 5-HIAA is largely excreted with the urine. In the pineal gland, 5-HT is further metabolized to melatonin by serotonin *N*-acetyltransferase (SNAT) and hydroxyindole *O*-methyltransferase (HIOMT).

[1]Max-Delbrück Center for Molecular Medicine (MDC), Robert-Rössle-Straße 10, 13125 Berlin-Buch, Germany
[2]University of Lübeck, Institute for Biology, Ratzeburger Allee 160, 23562 Lübeck, Germany
[3]Berlin Institute of Health (BIH), Anna-Louisa-Karsch-Straße 2, 10178 Berlin, Germany
[4]Charité University Medicine, Charitéplatz 1, 10117 Berlin, Germany
[5]German Center for Cardiovascular Research (DZHK), Partner Site Berlin, Berlin, Germany

*Correspondence: mbader@mdc-berlin.de (M. Bader).

residual amounts of 5-HT are also still found in the blood of knockout mice lacking both TPH1 and TPH2 [14], giving rise to the idea that phenylalanine hydroxylase may be another source of 5-HT synthesis *in vivo* because it is known that the enzyme is able to catalyze the hydroxylation of tryptophan to 5-hydroxytryptophan *in vitro* [17].

TPH1 and TPH2 are highly homologous proteins, but differ in their kinetic properties and, more notably, in their tissue distribution [18,19]. TPH2 is predominantly expressed in raphe neurons of the brainstem [15,18] and myenteric neurons in the gut [20,21], and is considered to be the source of the central neurotransmitter pool of 5-HT. The majority of blood 5-HT is synthesized by TPH1-expressing ECs of the gastrointestinal (GI) tract and enters the circulation packed in dense granules of thrombocytes where it mediates its hormonal actions upon platelet release at the site of activation. TPH1 is also found in other peripheral tissues such as pancreas, fat, and lung, as well as in the pineal gland where 5-HT serves as a precursor molecule for melatonin biosynthesis (Figure 1) [4,18,20]. TPH2 expression in enteric neurons does not noticeably contribute to the peripheral 5-HT pool because, as mentioned above, mice lacking both TPH1 and TPH2 exhibit the same blood concentrations of the monoamine as TPH1-deficient animals [14]. Likewise, TPH1 expression in the pineal gland does not contribute to the central 5-HT pool because TPH2-deficient animals have less than 1% of normal 5-HT levels in the brain [16].

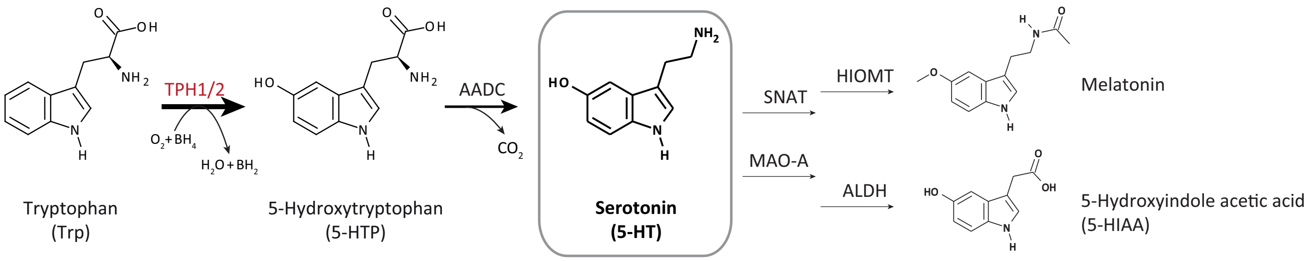

Figure 1. Metabolism of Serotonin (5-HT). Tryptophan hydroxylases TPH1/2 catalyze the conversion of the essential amino acid L-tryptophan (Trp) to 5-hydroxytryptophan (5-HTP) in the presence of molecular oxygen and the cofactor (6*R*)-L-erythro-5,6,7,8-tetrahydrobiopterin (BH4). 5-HTP is further converted to 5-HT by aromatic amino acid decarboxylase (AADC). 5-HT can be metabolized to melatonin by serotonin *N*-acetyltransferase (SNAT) and hydroxyindole *O*-methyltransferase (HIOMT), or degraded to 5-hydroxyindole acetic acid (5-HIAA) by monoamine oxidase A (MAO-A) and aldehyde dehydrogenase (ALDH).

In the past, therapeutics involving the serotonergic system only targeted 5-HT transporters and receptors, and were mostly aimed at central indications such as depression, psychosis, and migraine [22–24]. Nevertheless, the dualistic character of the 5-HT system, enforced by the inability of 5-HT to freely cross the blood–brain barrier in either direction, gave rise to the opportunity to pharmacologically target central and peripheral 5-HT biosynthesis in an independent manner. Advances and ideas in exploring TPH2 as a novel drug target have been comprehensively reviewed [25–27].

Analysis of *Tph1*$^{-/-}$ mice showed that an imbalance in peripheral 5-HT metabolism is associated with different diseases and pathophysiological processes such as GI disorders, **pulmonary arterial hypertension** (PAH), fibrosis, and inflammation. Most of these associated diseases are multifaceted in origin, which makes an effective treatment a challenge and has hampered the development of potent therapeutics because of the lack of knowledge of specific disease mechanisms and the non-applicability of the ‘one drug, one target, one indication’ paradigm. However, owing to the increasing medical need to treat multi-morbid patients, the pharmaceutical industry itself has suggested a new strategy – to address targets which influence common pathophysiological mechanisms, such as inflammation, angiogenesis, fibrosis, and cellular proliferation (all of which are linked to 5-HT), that are of therapeutic relevance in several diseases [28]. Hence in this review we aim to collect recent insights into TPH inhibitor drug development (Table 1) as well as first clinical studies (Supplemental information online) that consider peripheral, non-neuronal 5-HT biosynthesis as a novel therapeutic target.

Development of Inhibitors of TPH1

Even before the discovery of TPH2, naturally occurring unspecific inhibitors of TPH (and indoleamine metabolism) have been reported, including dopamine-derived tetrahydroisoquinolines such as salsolinol [29] and tetrahydropapaverine [30], and the food-derived carcinogenic heterocyclic amines Trp-P-1 and Trp-P-2 [31] (Supplemental information online). The low molecular weight phenylalanine analogs *p*-chlorophenylalanine (fenclonine, PCPA) and *p*-ethynylphenylalanine (PEPA) (Supplemental information online) were the first TPH inhibitors to be studied *in vivo*. PCPA has been used to treat patients with **carcinoid syndrome** and chemotherapy-induced emesis, but the compound was not suitable for therapeutic use because of unwanted central nervous system (CNS)-mediated side effects and its general ability to inhibit all members of the AAAH family [32,33]. PEPA is a more potent TPH inhibitor that showed less inhibitory activity towards phenylalanine hydroxylase and tyrosine hydroxylase, but again significantly reduced 5-HT concentration in the brain following intraperitoneal injection, which also restricted its therapeutic potential [34].

Since the functional classification of the TPH isoforms in 2003, both academia and the pharmaceutical industry have worked on establishing TPH1 as a pharmacological target (Figure 2), and the list of filed patents for novel small-molecule compounds inhibiting TPH to treat 5-HT-associated diseases is growing constantly (Box 2). Virtual and *in vitro* screening approaches were enabled by the successful production of functional TPH1 and TPH2 proteins within recombinant expression systems and the subsequent collection of X-ray co-crystal structures in the Protein Data Bank.

Lexicon Pharmaceuticals were the first to discover and characterize a novel series of substituted phenylalanine derivatives that selectively inhibit 5-HT biosynthesis in the GI tract [35]. The therapeutically essential selectivity for peripheral 5-HT inhibition can be achieved by developing inhibitors that either only act locally in ECs of the gut from the luminal side without entering the

Glossary

5-Hydroxytryptamine (5-HT): another name for serotonin. There are seven families of receptors for 5-HT, 5-HT_1 to 5-HT_7.

Bleomycin: a chemotherapeutic drug used in cancer treatment. Treatment is limited by the most important adverse effect, pulmonary fibrosis. This side effect is used in animal models of pulmonary fibrosis induced by intratracheal application of the drug.

Carcinoid syndrome: neuroendocrine tumors (NETs) of the gut often generate high amounts of 5-HT and other mediators, and thereby cause carcinoid syndrome, in particular after metastasizing to the liver.

Irritable bowel syndrome (IBS): IBS is characterized by abdominal pain and altered patterns of bowel movements with unknown etiology, which occur over a long time and lead to frequent diarrhea or constipation.

Pulmonary arterial hypertension (PAH): a rare lung disease with high mortality, characterized by increased pulmonary arterial pressure and remodeling, as well as by right ventricular hypertrophy.

Serotonin transporter (SERT, SLC6A4): 5-HT transporter on the plasma membrane.

Serotonylation: a receptor-independent intracellular signaling mechanism in which 5-HT is covalently bound to small GTPases by transglutaminase 2.

Telotristat ethyl: the free base form of the hippurate salt telotristat etiprate. Also referred to as LX-1032, LX-1606, and LP-778914. The first FDA-approved TPH inhibitor drug (Xermelo^{TM}).

Telotristat etiprate: hippurate salt form of telotristat ethyl.

Tryptophan hydroxylase (TPH): the first and rate-limiting enzyme in 5-HT synthesis from tryptophan. There are two isoforms, TPH1 in enterochromaffin cells (ECs) of the gut, and TPH2 in raphe nuclei in the brain.

Table 1. Overview of the *In Vivo* Effects of Specific TPH Inhibitors in Different Disease Models[a]

Inhibitor	Disease model (species)	Administration	Effect	Refs
LP-615819	Cisplatin-induced emesis (ferret)	20, 60, and 180 mg/kg twice daily oral gavage for 5 days	Non-significant 50% reduction in emesis severity	[35]
LP-533401	High-fat diet-induced obesity (mouse)	100 mg/kg daily by gavage for 12 weeks	Improved metabolic parameters; no change in weight gain	[76]
LP-533401	High-fat diet-induced obesity (mouse)	25 mg/kg daily by intraperitoneal injection for 12 weeks	Reduced gain of weight and fat; improved metabolic parameters	[74]
LP-533401	High-fat diet-induced obesity (mouse)	30 mg/kg daily by intraperitoneal injection for 14 weeks	Reduced gain of weight and fat; browning of white fat	[75]
LP-533401	Pulmonary hypertension (mouse)	250 mg/kg daily by oral gavage for 14 days	Reduced pulmonary artery muscularization, right ventricular pressure, and hypertrophy	[125]
LP-533401	Ligature-induced periodontal disease (rat)	25 mg/kg daily by gavage for 28 days	No reduction in bone loss	[121]
LP-533401	Osteoporosis (ovariectomy in mouse and rat)	1–250 mg/kg daily in diet for 28 days	Normalization of bone mass	[116]
LP-533401	Osteoporosis (ovariectomy in mouse)	5–25 mg/kg daily in diet for 6 or 12 weeks	Increase in bone mass	[126]
LP-923941[b]	Osteoporosis (ovariectomy in mouse and rat)	250 mg/kg daily by oral gavage for 28 days	No significant effect on bone mass	[119]
LX-1606[c]	Bone loss (rat strain with high platelet 5-HT)	25 mg/kg daily by oral gavage for 36 days	Non-significant increase in bone mass	[127]
LP-920540[d]	Necrotizing enterocolitis (mouse)	200 mg/kg daily by gavage for 5 days	Reduced weight loss; improved survival and histology	[70]
LP-920540[d] and LX-1032[c]	Trinitrobenzene sulfonic acid-induced colitis (mouse)	30 mg/kg daily LP-920540[d] for 5 days; 200 mg/kg daily LX1032[c] for 5 days	Reduced weight loss; improved stool consistency and histology	[72]
LX-1606[c]	Dextran sulfate-induced colitis (mouse)	300 mg/kg daily oral gavage for 5 or 6 days	Delayed onset and severity	[71]
LX-1606[c]	*Trichuris muris*-induced colitis (mouse)	300 mg/kg daily by oral gavage for 14 or 21 days	Enhanced worm expulsion and goblet cell number	[71]
KAR5585 and KAR5416	Pulmonary hypertension (rat)	100 or 200 mg/kg daily by oral gavage for 28 days	Reduced pulmonary arterial pressure, vessel wall thickness, and occlusion	[45]
mol002291	Trinitrobenzene sulfonic acid-induced colitis (rat)	40, 80, or 160 mg/kg daily for 3 weeks	Decreased visceral hyperalgesia	[46]

[a]Studies using PCPA were excluded owing to the nonspecific effects of this drug.
[b]Active enantiomer of LP-533401.
[c]Now referred to as telotristat ethyl.
[d]Active enantiomer of LP-615819.

bloodstream, are unable to cross the blood–brain barrier, or do not inhibit TPH2 (Table 2). However, most inhibitors developed so far are non-selective and equally block TPH1 and TPH2, probably owing to the very high homology between the two enzymes at the active site [18]. The new TPH inhibitors of Lexicon (e.g., LP-521834, LP-534193, LP-533401 and its active enantiomer LP-923941, LP-615819 and its active enantiomer LP-920540; Supplemental information online) were found to competitively bind to the tryptophan pocket of both TPH isoforms [36] and selectively reduce peripheral 5-HT levels in mice after oral administration by 50% because of their inability to penetrate the blood–brain barrier [37,38]. LX-1031 and the more potent back-up compound LX-1033 (Supplemental information online) were specifically optimized for restricted TPH inhibition in ECs of the GI tract because they do not pass the

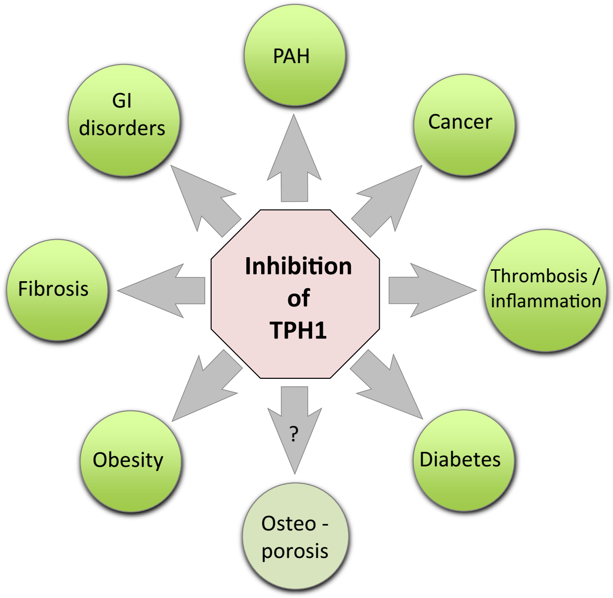

Figure 2. Schematic Representation of the Therapeutic Options for TPH1 Inhibitors. Selective blockade of TPH1 enzyme activity results in reduced blood and non-neuronal tissue levels of 5-HT, with a potentially beneficial outcome for disease-related pathophysiological parameters in various disorders of multifaceted origin. The same effects would be expected for non-selective TPH inhibitors which do not pass the blood–brain barrier. Abbreviations: GI, gastrointestinal; PAH, pulmonary arterial hypertension, TPH, tryptophan hydroxylase.

intestinal barrier and were the first compounds to enter clinical trials for GI diseases such as **irritable bowel syndrome** (IBS; Supplemental information online) [39]. In parallel, **telotristat ethyl** (also known as LX-1032/LX-1606/**telotristat etiprate**; Supplemental information online) was developed to inhibit 5-HT in the periphery but not in the brain because it enters the bloodstream but cannot pass the blood–brain barrier. It has recently been approved by the FDA as the first small-molecule, peripheral TPH inhibitor to treat carcinoid syndrome diarrhea in combination with somatostatin analog therapy in adults [40,41]. Karos Pharmaceuticals used a structure-based design methodology approach to further develop the structural scaffolds of the Lexicon compounds, and discovered a new class of peripherally restricted TPH inhibitors that lack the characteristic phenylalanine moiety [42,43]. These spirocyclic proline-based TPH1 inhibitors were chemically and pharmacokinetically optimized (KAR5585, KAR5417; Supplemental information online) [44], successfully tested *in vivo* [45], and have already entered a Phase I first-in-human study (Supplemental information online).

Recent publications claim to have discovered the first specific inhibitors of TPH1. High-throughput, docking-based, virtual screening of a Traditional Chinese Medicines database identified 1-galloypedunculagin (mol002291, Supplemental information online) as a potential TPH inhibitor, a molecule derived from the Chinese herb *Rheum officinale*. The molecule specifically inhibited TPH1 *in vitro* and reduced colonic 5-HT content, and also attenuated visceral hyperalgesia *in vivo* [46] (Table 1). Another recently published Novartis-funded study featured *in vitro* and *in vivo* efficacy data of a novel allosteric TPH1-selective inhibitor that was found in a high-throughput proximity assay screen [47]. An overview of the *in vivo* effects of the

Box 2. Patents and Patent Applications on TPH Inhibitors

Devasagayaraj, A. *et al*. Lexicon Pharmaceuticals, Inc. Multicyclic amino acid derivatives and methods of their use, WO2007089335:A2.2007

Devasagayaraj, A. *et al*. Lexicon Pharmaceuticals, Inc. 4-Phenyl-6-(2,2,2-trifluoro-1-phenylethoxy)pyrimidine-based compounds and methods of their use, WO2008073933:A2.2008

Bomont, C. *et al*. Lexicon Pharmaceuticals, Inc. Tryptophan hydroxylase inhibitors and methods of their use, WO2010039957:A1.2010

Imura, S. *et al*. Lexicon Pharmaceuticals, Inc. Process for the preparation of substituted phenylalanines, WO2011063072.A2.2011

Bur, D. *et al*. Actelion Pharmaceuticals, Ltd. Tricyclic piperidine compounds, WO2015075023:A1.2015

Bur, D. *et al*. Actelion Pharmaceuticals, Ltd. Tricyclic imidazole compounds as inhibitors of tryptophan hydroxylase, WO2015075025:A1.2015

De Lombaert, S. *et al*. Karos Pharmaceuticals, Inc. Spirocyclic compounds as tryptophan hydroxylase inhibitors, WO2015035113:A1.2015

Bader, M. *et al*. Max-Delbrück-Centrum für Molekulare Medizin, Forschungsverbund Berlin E.V. Xanthine derivatives, their use as a medicament, and pharmaceutical preparations comprising the same, WO2016135199:A1.2016

Table 2. Subclasses of TPH Inhibitors with Different Bioavailabilities and Isoform Selectivities, and Their Effects on TPH Activity and 5-HT Levels in Different Tissues[a]

Subclass of TPH Inhibitors	Examples	TPH1 in ECs	TPH1 in other tissues	TPH2 in raphe nuclei	TPH2 in enteric neurons	Blood 5-HT	Brain 5-HT
Non-selective; do not pass the intestinal barrier	LX-1031, LX-1033	↓	–	–	–	↓	–
Non-selective; do not pass the blood–brain barrier	Telotristat ethyl, KAR5585, KAR5417	↓	↓	–	↓	↓	–
Non-selective; pass the intestinal and blood–brain barriers	PCPA, PEPA	↓	↓	↓	↓	↓	↓
TPH1-selective	mol002291	↓	↓	–	–	↓	–

[a]Symbols: ↓, downregulation; –, no change.

newly discovered TPH inhibitors in different disease models and in clinical trials is given in Table 1.

5-HT Function and Therapeutic Implications in Peripheral Organs

Cancer

Multiple studies have found an involvement of 5-HT in tumor biology and cancer progression, influencing tumor growth, angiogenesis, and cancer cell differentiation and migration in the affected organs [48]. Among the most prominent examples are neuroendocrine tumors (NETs),

a genetically and clinically heterogeneous group of GI, pancreatic, and bronchial tumors. Upon systemic release of different hormonal mediators and vasoactive amines, NETs are able to induce a series of symptoms, called carcinoid syndrome, characterized by diarrhea, cutaneous flushing, wheezing, bronchoconstriction, cardiac valve disease, and mesenteric fibrosis [49]. Carcinoid syndrome is associated with up to 80% of GI NETs and about 1% of pancreatic NETs [41]. These mainly metastatic carcinoid tumors overexpress TPH1 and excessively secrete 5-HT into circulation [50], where it is involved in the development of acute and chronic pathophysiologic symptoms [33,49]. Thus, carcinoid syndrome became the first indication that was successfully targeted by an FDA-approved oral, small-molecule inhibitor of TPH1 (telotristat ethyl; Supplemental information online) [41,51]. Several Phase II and Phase III clinical trials have proven the safety and efficacy of this drug to alleviate the symptoms of this disease (Supplemental information online) [52–55].

The high 5-HT-synthesizing activity in NETs can also be exploited to inhibit their growth by applying the TPH inhibitor and suicide compound, 7-hydroxytryptophan, which is converted to the toxin 5,7-dihydroxytryptamine in TPH- and AADC-expressing cells (Figure 1) [56,57]. This strategy was successfully employed *in vitro*, but so far there are no published *in vivo* studies.

Elevated 5-HT levels, resulting from increased TPH1 and decreased expression of the 5-HT-degrading enzyme MAO-A (Figure 1), are also found in pancreatic ductal adenocarcinoma (PDAC), and correlated with cancer stage and patient survival time [58]. The same group also showed 5-HT-dependent tumor growth in PDAC-derived xenografts in mice [58]. Furthermore, malignant breast cancer progression was shown to be accompanied by increased TPH1 expression, 5-HT production and alterations in 5-HT receptor expression and signaling, leading to stimulated proliferation and resistance to apoptosis [59,60]. *In vitro* studies with MDA-MB-231, a cell line derived from a highly aggressive breast cancer subtype called triple-negative breast cancer, showed elevated expression levels of TPH1, which were further augmented upon 5-HT treatment. The stimulatory effect of 5-HT on MDA-MB-231 cells promoted invasion and proliferation via different downstream signaling pathways of the 5-HT_7 receptor, suggesting a stronger influence during the first stage of metastasis (invasion and migration) than during the later proliferative phase [61]. In addition to antagonists of SERT and 5-HT receptors, the TPH1 inhibitor LP-533401 reportedly compromised the activity of breast tumor initiating cells in a functional sphere-forming *in vitro* assay [62].

Not only tumor cells but also tumor-infiltrating macrophages are targets of 5-HT. 5-HT deficiency in *Tph1*$^{-/-}$ mice leads to increased generation of angiostatin in these cells, which inhibits the growth of tumor-supporting vessels [63]. Thus, peripheral 5-HT could represent a target for the treatment of several cancers, and TPH inhibitors might be of therapeutic use to prevent cancer progression into invasive and metastatic forms which are associated with poor prognosis.

GI Diseases

In the periphery, GI diseases are the only disorders for which serotonergic drugs are already in clinical use. 5-HT_3 antagonists are used as anti-emetics and, together with 5-HT_4 agonists, they are also effective drugs for IBS [64]. The predominance of the gut as a target for serotonergic drugs is not surprising because the gut and its ECs express nearly all TPH1 in the body and are therefore the most important source of 5-HT in the organism. Surprisingly, *Tph1*$^{-/-}$ mice thrived normally and did not confirm the expected essential function of EC-derived 5-HT in gut mucosal integrity, motility, and/or secretion [13,14,65,66]. Nonetheless, one study found significant impairments in colonic propulsion and peristaltic reflexes [67]. TPH2

in myenteric neurons seems to be more important for gut motility because it was shown to be altered in *Tph2*$^{-/-}$ mice [65,66]. Nevertheless, 5-HT synthesis in the gut was suggested as a target for GI diseases such as IBS. Accordingly, *Tph1*$^{-/-}$ mice and animals treated with LP-920540 or telotristat ethyl are protected in several different colitis models, including necrotizing enterocolitis and colitis induced by oral dextran sulfate sodium administration or trinitrobenzene sulfate, as well as in an infection-induced intestinal inflammation model [68–72] (Table 1). Based on these results, a Phase II clinical trial with LX-1031 was performed in diarrhea-predominant IBS, and slight but significant symptom relief was achieved in the patients (Supplemental information online) [39,73]. Thus, TPH inhibitors may be promising drugs for the treatment of inflammatory bowel diseases.

Obesity and Diabetes

Two recent seminal studies with *Tph1*$^{-/-}$ mice suggest a central role of peripheral 5-HT in obesity [74,75]. Tissue-specific knockout models revealed that TPH1 in adipocytes and local 5-HT generation in the fat is more important for resistance against a high-fat diet than is EC-derived 5-HT [75,76] The absence of 5-HT or TPH inhibition by LP-533401 facilitated norepinephrine-induced browning and stimulated thermogenesis in brown adipocytes [74,75], explaining their anti-obesity effect (Table 1).

Moreover, improved glucose tolerance and increased glucose uptake, at least in some tissues, was observed in mice deficient in TPH1 and after LP-533401 treatment [74–76] (Table 1). On the other hand, insulin secretion of pancreatic β cells is dependent on **serotonylation** of small G proteins, and is therefore impaired in *Tph1*$^{-/-}$ mice [77]. Again, local generation of TPH1 and 5-HT is pivotal for the phenotype, as shown by a β cell-specific *Tph1* knockout mouse [78]. This local 5-HT synthesis is also essential for the adaptation of insulin-producing cells to the additional needs during pregnancy [79].

Because most of these metabolic effects have not only been seen in *Tph1*$^{-/-}$ mice but also after treatment with TPH inhibitors, these compounds have potential as novel anti-obesity or anti-diabetic drugs. However, whether TPH1 inhibition in pancreatic β cells may have deleterious effects, in particular in pregnancy, needs further clarification.

Thrombosis and Inflammation

The first phenotype observed in *Tph1*$^{-/-}$ mice was a prolonged tail bleeding time [80]. The reason for this phenotype was impaired release of von Willebrand factor and other proteins from platelet α granules, hampering platelet aggregation and adhesion to the basal membrane, and consequently thrombus formation [80,81]. Accordingly, *Tph1*$^{-/-}$ mice were protected from thrombosis induced by endothelial damage or treatment with epinephrine and collagen [80]. Mechanistically, 5-HT activates the release of platelet α granules in two interconnected ways – binding to the 5-HT_{2A} receptor on the plasma membrane and serotonylation of small G proteins after uptake by SERT into the cytoplasm [80].

Platelets are the main source of 5-HT in the periphery, and release it at sites of injury and inflammation, with marked effects on cells of the immune system. Neutrophils in particular seem to react to platelet-derived 5-HT in inflammation because neutrophil-driven shock states are less pronounced in *Tph1*$^{-/-}$ mice and in animals treated with PCPA [82–85]. In addition, other cell types of the immune system may also be responsive to platelet-derived 5-HT, such as dendritic cells. In *Tph1*$^{-/-}$ mice this leads to reduced allergic airway inflammation as a result of defective T cell priming [86]. Moreover, postoperative abdominal adhesion formation, which is

also thought to be an inflammatory process, was reduced in *Tph1*$^{-/-}$ and PCPA-treated mice [87].

However, in acute pancreatitis *Tph1*$^{-/-}$ mice exhibit a dual phenotype: they are protected from early inflammation based on attenuated leukocyte infiltration, but for so far unknown reasons show accelerated disease progression [88]. An aggravated pathology in *Tph1*$^{-/-}$ mice was also reported for other inflammation models such as autoimmune arthritis [89] and experimental autoimmune encephalitis (a model of multiple sclerosis) [90]. Thus, the use of TPH inhibitors in inflammatory diseases will probably be restricted to specific disease entities which need to be defined by further preclinical and clinical studies.

Fibrosis

Systemic fibrotic diseases (e.g., systemic sclerosis) and organ-specific fibrosis (e.g., kidney fibrosis, idiopathic pulmonary fibrosis, liver cirrhosis) share pathogenetic mechanisms. In all these diseases, activated fibroblasts release augmented amounts of extracellular matrix that disrupt tissue structure, leading to organ dysfunction and to high morbidity and mortality in affected patients [91]. 5-HT is released upon activation of platelets and is elevated in the blood of systemic sclerosis patients [6]. More than 50 years ago a pathogenetic role of 5-HT in tissue fibrosis was postulated based on observations in carcinoid patients with increased blood 5-HT [92,93]. Moreover, patients and mice with lung fibrosis exhibit upregulated expression of 5-HT_{2A} receptors, and lung 5-HT is increased in **bleomycin**-treated mice [94,95]. Furthermore, 5-$HT_{2A/B}$ receptor antagonists exert antifibrotic effects in the same [94,96] and other animal models of tissue fibrosis [95,97]. In a mouse model of systemic sclerosis, 5-HT levels are also elevated, and genetic deficiency of 5-HT_{2B} receptors or of TPH1 reduced dermal fibrosis [95]. In addition to the circulating 5-HT, local biosynthesis of 5-HT occurs in the lung itself, contributing to tissue 5-HT levels. Accordingly, in the bleomycin-induced lung fibrosis rat model, lung 5-HT levels and synthesis were correlated with lung *Tph1* mRNA levels, suggesting a contribution from local 5-HT synthesis [98].

Thus, 5-HT in peripheral tissues consistently promotes fibrotic diseases, and therefore TPH inhibition is promising as a novel therapeutic strategy against these diseases.

Pulmonary Arterial Hypertension

There is ample evidence that 5-HT plays a major role in the pathogenesis of PAH. Accordingly, appetite-suppressants releasing 5-HT, such as fenfluramine, had to be retracted from the market because of an unacceptably high rate of drug-induced PAH [99]. Patients and animals with platelet storage disease and thereby increased plasma 5-HT levels often develop PAH [100,101]. Moreover, there is evidence for local expression of TPH1 and local 5-HT synthesis in the lung, with significant impact on the pathogenesis of PAH [102,103]. Genetic depletion of TPH1 protects mice from experimentally induced PAH [103–107]. Moreover, overexpression of SERT in lung arteries causes PAH [108], and patients with PAH show increased 5-HT levels in lung and blood [109]. Very recently, it has been shown that TPH inhibition ameliorates PAH in two different animal models [45] (Table 1).

Therefore, PAH is most likely one of the next disease indications for which TPH inhibitors will be tested in clinical trials, in particular because PAH is a rare disease, which will facilitate regulatory approval of this application.

Cell Press REVIEWS

Liver Diseases

In the liver the effects of 5-HT are bivalent. It has been shown that *Tph1*$^{-/-}$ mice cannot regenerate their liver after resection or ischemia–reperfusion damage [110,111]. In addition, these mice are also more severely affected by liver injury through bile duct ligation or acetaminophen treatment [112,113]. However, in a nonalcoholic steatohepatitis model, *Tph1*$^{-/-}$ mice are protected due to reduced generation of reactive oxygen species [114]. The same is true for viral hepatitis; in this case the effects of platelet 5-HT on the microcirculation and subsequent virus elimination are causative [115].

Thus, the use of TPH inhibitors in liver diseases depends on the etiology of the disease, and needs further preclinical and clinical evaluation.

Osteoporosis

The most prominent example for the therapeutic use of TPH inhibitors was published in 2010, showing that osteoporosis could be blunted in rodent models by LP-533401 [116] (Table 1). These experiments were based on findings of the same group that mice deficient in EC TPH1 develop increased bone density [117]. This group also showed a reduced bone mass phenotype in *Tph2*$^{-/-}$ mice, and postulated a mechanism by which TPH2-derived 5-HT stimulates bone growth, in contrast to 5-HT synthesized by TPH1 in the gut, which blocks it [118]. However, there are also conflicting studies by other authors which do not show effects of LP-923941 (the active enantiomer of LP-533401) on osteoporosis in animal models [119,120] (Table 1). Moreover, bone loss in a rat periodontitis model could not be inhibited by LP-533401 [121] (Table 1). There has been a public debate about these inconsistencies [122,123], but the reasons for the discrepant results have not yet been resolved. Therefore, the therapeutic application of TPH inhibitors in osteoporosis remains controversial, and further (clinical) studies will be necessary to clarify the issue.

Concluding Remarks and Future Perspectives

The duality of the serotonergic system in vertebrates, revealed by the discovery of the neuron-specific TPH2 through *Tph1*$^{-/-}$ mice [13], has paved the way for directed interference with central and peripheral 5-HT functions. Pathophysiological processes in several complex disorders such as carcinoid tumors, PAH, and IBS are associated with gut-derived and/or locally synthesized 5-HT. Consequently, TPH1 emerged as a possible therapeutic target for the development of inhibitors. In February 2017 the drug telotristat ethyl (Xermelo™, Lexicon Pharmaceuticals) was the first-in-class, small-molecule, peripheral TPH inhibitor to be approved in the USA for the treatment of carcinoid syndrome diarrhea in combination with somatostatin analog therapy [40]. This shows that the diversity of 5-HT actions constitutes a challenge as well as an opportunity because many of the disorders mentioned in this review lack adequate therapy because of their multifaceted pathophysiology. Hence it is anticipated that a (co)-treatment with TPH inhibitors might also be beneficial in other inflammatory and fibrotic diseases, where a combination of different treatment options seems to be a promising way to improve therapeutic outcome. However, owing to the essential role of TPH1-derived 5-HT in embryo development [2,3], and in the adjustment of maternal insulin production [79] and mammary gland function [124], the use of TPH inhibitors in pregnant and lactating women needs further investigation (see Outstanding Questions). Moreover, it remains to be clarified whether it is sufficient to avoid adverse CNS effects of TPH inhibitors by using drugs reported to not pass the blood–brain barrier, such as telotristat ethyl, because the exclusion from the brain may not be complete. Alternatively, it may be necessary to develop novel compounds which do not inhibit TPH2 [46,47]. However, based on the very high homology between TPH1 and TPH2, in particular at the active site, the development of selective drugs will be a challenge.

Outstanding Questions

Will it be possible to generate efficient TPH1-specific inhibitors or is it sufficient to have drugs that do not pass the blood–brain barrier?

Will TPH inhibition also fulfill the promises, based on animal studies, in clinical trials – in particular in pulmonary hypertension, fibrotic disease, and osteoporosis?

Will TPH inhibition be a safe treatment without intolerable side effects, and will it be acceptable to treat pregnant or lactating women?

It is expected that the use of the novel TPH inhibitors in preclinical disease models as well as the deep phenotyping of TPH knockout mice will reveal additional indications for the use of these drugs. Moreover, such studies may reveal the downstream pathways by which 5-HT acts, and thereby unravel novel targets for pharmacological intervention for particular diseases.

Acknowledgments

Research by the authors was supported by grants of the German Ministry for Education and Research (16V0276 and the DZHK, German Centre for Cardiovascular Research).

Supplemental Information

Supplemental information associated with this article can be found online at https://doi.org/10.1016/j.tips.2018.03.004.

References

1. Rapport, M.M. *et al.* (1948) Partial purification of the vasoconstrictor in beef serum. *J. Biol. Chem.* 174, 735–741
2. Cote, F. *et al.* (2007) Maternal serotonin is crucial for murine embryonic development. *Proc. Natl. Acad. Sci. U. S. A.* 104, 329–334
3. Bonnin, A. *et al.* (2011) A transient placental source of serotonin for the fetal forebrain. *Nature* 472, 347–350
4. Amireault, P. *et al.* (2013) Life without peripheral serotonin: insights from tryptophan hydroxylase 1 knockout mice reveal the existence of paracrine/autocrine serotonergic networks. *ACS Chem. Neurosci.* 4, 64–71
5. Monassier, L. *et al.* (2010) Contribution of gene-modified mice and rats to our understanding of the cardiovascular pharmacology of serotonin. *Pharmacol. Ther.* 128, 559–567
6. Mann, D.A. and Oakley, F. (2013) Serotonin paracrine signaling in tissue fibrosis. *Biochim. Biophys. Acta* 1832, 905–910
7. Mosienko, V. *et al.* (2015) Life without brain serotonin: reevaluation of serotonin function with mice deficient in brain serotonin synthesis. *Behav. Brain Res.* 277, 78–88
8. Gershon, M.D. (2013) 5-Hydroxytryptamine (serotonin) in the gastrointestinal tract. *Curr. Opin. Endocrinol. Diabetes Obes.* 20, 14–21
9. Watts, S.W. *et al.* (2012) Serotonin and blood pressure regulation. *Pharmacol. Rev.* 64, 359–388
10. Filip, M. and Bader, M. (2009) Overview on 5-HT receptors and their role in physiology and pathology of the central nervous system. *Pharmacol. Rep.* 61, 761–777
11. Fitzpatrick, P.F. (1999) Tetrahydropterin-dependent amino acid hydroxylases. *Annu. Rev. Biochem.* 68, 355–381
12. Kobayashi, K. *et al.* (1995) Targeted disruption of the tyrosine hydroxylase locus results in severe catecholamine depletion and perinatal lethality in mice. *J. Biol. Chem.* 270, 27235–27243
13. Walther, D.J. *et al.* (2003) Synthesis of serotonin by a second tryptophan hydroxylase isoform. *Science* 299, 76
14. Savelieva, K.V. *et al.* (2008) Genetic disruption of both tryptophan hydroxylase genes dramatically reduces serotonin and affects behavior in models sensitive to antidepressants. *PLoS One* 3, e3301
15. Gutknecht, L. *et al.* (2008) Deficiency of brain 5-HT synthesis but serotonergic neuron formation in Tph2 knockout mice. *J. Neural Transm.* 115, 1127–1132
16. Alenina, N. *et al.* (2009) Growth retardation and altered autonomic control in mice lacking brain serotonin. *Proc. Natl. Acad. Sci. U. S. A.* 106, 10332–10337
17. Renson, J. *et al.* (1962) Hydroxylation of tryptophan by phenylalanine hydroxylase. *J. Biol. Chem.* 237, 2261–2264
18. Walther, D.J. and Bader, M. (2003) A unique central tryptophan hydroxylase isoform. *Biochem. Pharmacol.* 66, 1673–1680
19. McKinney, J. *et al.* (2005) Different properties of the central and peripheral forms of human tryptophan hydroxylase. *J. Neurochem.* 92, 311–320
20. Cote, F. *et al.* (2003) Disruption of the nonneuronal Tph1 gene demonstrates the importance of peripheral serotonin in cardiac function. *Proc. Natl. Acad. Sci. U. S. A.* 100, 13525–13530
21. Neal, K.B. *et al.* (2009) Strain-specific genetics, anatomy and function of enteric neural serotonergic pathways in inbred mice. *J. Physiol.* 587, 567–586
22. Stahl, S.M. *et al.* (2013) Serotonergic drugs for depression and beyond. *Curr. Drug Targets* 14, 578–585
23. Werner, F.M. and Covenas, R. (2014) Safety of antipsychotic drugs: focus on therapeutic and adverse effects. *Expert Opin. Drug Saf.* 13, 1031–1042
24. Derry, C.J. *et al.* (2014) Sumatriptan (all routes of administration) for acute migraine attacks in adults – overview of Cochrane reviews. *Cochrane Database Syst. Rev.* CD009108
25. Matthes, S. *et al.* (2010) Tryptophan hydroxylase as novel target for the treatment of depressive disorders. *Pharmacology* 85, 95–109
26. Torrente, M.P. *et al.* (2012) Boosting serotonin in the brain: is it time to revamp the treatment of depression? *J. Psychopharmacol.* 26, 629–635
27. Waloen, K. *et al.* (2017) Tyrosine and tryptophan hydroxylases as therapeutic targets in human disease. *Expert Opin. Ther. Targets* 21, 167–180
28. Nielsch, U. *et al.* (2007) One target – multiple indications: a call for an integrated common mechanisms strategy. *Drug Discov. Today* 12, 1025–1031
29. Ota, M. *et al.* (1992) Inhibition of tryptophan hydroxylase by (R)- and (S)-1-methyl-6,7-dihydroxy-1,2,3,4-tetrahydroisoquinolines (salsolinols). *Neuropharmacology* 31, 337–341
30. Kim, E.I. *et al.* (2004) Inhibitory effects of tetrahydropapaverine on serotonin biosynthesis in murine mastocytoma P815 cells. *Life Sci.* 75, 1949–1957
31. Naoi, M. *et al.* (1991) Inhibition of tryptophan hydroxylase by food-derived carcinogenic heterocyclic amines, 3-amino-1-methyl-5H-pyrido[4,3-b]indole and 3-amino-1,4-dimethyl-5H-pyrido[4,3-b]indole. *Biochem. Pharmacol.* 41, 199–203
32. Engelman, K. *et al.* (1967) Inhibition of serotonin synthesis by para-chlorophenylalanine in patients with the carcinoid syndrome. *N. Engl. J. Med.* 277, 1103–1108
33. Kvols, L.K. (1994) Metastatic carcinoid tumors and the malignant carcinoid syndrome. *Ann. N. Y. Acad. Sci.* 733, 464–470
34. Zimmer, L. *et al.* (2002) Short- and long-term effects of p-ethynylphenylalanine on brain serotonin levels. *Neurochem. Res.* 27, 269–275
35. Liu, Q. *et al.* (2008) Discovery and characterization of novel tryptophan hydroxylase inhibitors that selectively inhibit serotonin synthesis in the gastrointestinal tract. *J. Pharmacol. Exp. Ther.* 325, 47–55
36. Cianchetta, G. *et al.* (2010) Mechanism of inhibition of novel tryptophan hydroxylase inhibitors revealed by co-crystal structures and kinetic analysis. *Curr. Chem. Genomics* 4, 19–26

CellPress REVIEWS

37. Shi, Z.C. *et al.* (2008) Modulation of peripheral serotonin levels by novel tryptophan hydroxylase inhibitors for the potential treatment of functional gastrointestinal disorders. *J. Med. Chem.* 51, 3684–3687
38. Jin, H. *et al.* (2009) Substituted 3-(4-(1,3,5-triazin-2-yl)-phenyl)-2-aminopropanoic acids as novel tryptophan hydroxylase inhibitors. *Bioorg. Med. Chem. Lett.* 19, 5229–5232
39. Camilleri, M. (2011) LX-1031, a tryptophan 5-hydroxylase inhibitor, and its potential in chronic diarrhea associated with increased serotonin. *Neurogastroenterol. Motil.* 23, 193–200
40. Markham, A. (2017) Telotristat ethyl: first global approval. *Drugs* 77, 793–798
41. Masab, M. and Saif, M.W. (2017) Telotristat ethyl: proof of principle and the first oral agent in the management of well-differentiated metastatic neuroendocrine tumor and carcinoid syndrome diarrhea. *Cancer Chemother. Pharmacol.* 80, 1055–1062
42. Goldberg, D.R. *et al.* (2016) Discovery of acyl guanidine tryptophan hydroxylase-1 inhibitors. *Bioorg. Med. Chem. Lett.* 26, 2855–2860
43. Goldberg, D.R. *et al.* (2016) Discovery of spirocyclic proline tryptophan hydroxylase-1 inhibitors. *Bioorg. Med. Chem. Lett.* 26, 1124–1129
44. Goldberg, D.R. *et al.* (2017) Optimization of spirocyclic proline tryptophan hydroxylase-1 inhibitors. *Bioorg. Med. Chem. Lett.* 27, 413–419
45. Aiello, R.J. *et al.* (2017) Tryptophan hydroxylase 1 inhibition impacts pulmonary vascular remodeling in two rat models of pulmonary hypertension. *J. Pharmacol. Exp. Ther.* 360, 267–279
46. Shi, H. *et al.* (2018) Discovery and characterization of a novel tryptophan hydroxylase 1 inhibitor as a prodrug. *Chem. Biol. Drug Des.* 91, 202–212
47. Petrassi, M. *et al.* (2017) Identification of a novel allosteric inhibitory site on tryptophan hydroxylase 1 enabling unprecedented selectivity over all related hydroxylases. *Front. Pharmacol.* 8, 240
48. Sarrouilhe, D. *et al.* (2015) Serotonin and cancer: what is the link? *Curr. Mol. Med.* 15, 62–77
49. Molina-Cerrillo, J. *et al.* (2016) Inhibition of peripheral synthesis of serotonin as a new target in neuroendocrine tumors. *Oncologist* 21, 701–707
50. Lovenberg, W. *et al.* (1967) Tryptophan hydroxylation: measurement in pineal gland, brainstem, and carcinoid tumor. *Science* 155, 217–219
51. Lamarca, A. *et al.* (2016) Telotristat ethyl: a new option for the management of carcinoid syndrome. *Expert Opin. Pharmacother.* 17, 2487–2498
52. Kulke, M.H. *et al.* (2017) Telotristat ethyl, a tryptophan hydroxylase inhibitor for the treatment of carcinoid syndrome. *J. Clin. Oncol.* 35, 14–23
53. Pavel, M. *et al.* (2015) Telotristat etiprate for carcinoid syndrome: a single-arm, multicenter trial. *J. Clin. Endocrinol. Metab.* 100, 1511–1519
54. Kulke, M.H. *et al.* (2014) Telotristat etiprate, a novel serotonin synthesis inhibitor, in patients with carcinoid syndrome and diarrhea not adequately controlled by octreotide. *Endocr. Relat. Cancer* 21, 705–714
55. Lapuerta, P. *et al.* (2015) Telotristat etiprate, a novel inhibitor of serotonin synthesis for the treatment of carcinoid syndrome. *Clin. Invest.* 5, 447–456
56. Drozdov, I. *et al.* (2009) Autoregulatory effects of serotonin on proliferation and signaling pathways in lung and small intestine neuroendocrine tumor cell lines. *Cancer* 115, 4934–4945
57. Walther, D.J. *et al.* (2002) 7-Hydroxytryptophan, a novel, specific, cytotoxic agent for carcinoids and other serotonin-producing tumors. *Cancer* 94, 3135–3140
58. Jiang, S.H. *et al.* (2017) Increased serotonin signaling contributes to the warburg effect in pancreatic tumor cells under metabolic stress and promotes growth of pancreatic tumors in mice. *Gastroenterology* 153, 277–291
59. Pai, V.P. *et al.* (2009) Altered serotonin physiology in human breast cancers favors paradoxical growth and cell survival. *Breast Cancer Res.* 11, R81
60. Leoncikas, V. *et al.* (2016) Generation of 2,000 breast cancer metabolic landscapes reveals a poor prognosis group with active serotonin production. *Sci. Rep.* 6, 19771
61. Gautam, J. *et al.* (2016) Tryptophan hydroxylase 1 and 5-HT7 receptor preferentially expressed in triple-negative breast cancer promote cancer progression through autocrine serotonin signaling. *Mol. Cancer* 15, 75
62. Gwynne, W.D. *et al.* (2017) Serotonergic system antagonists target breast tumor initiating cells and synergize with chemotherapy to shrink human breast tumor xenografts. *Oncotarget* 8, 32101–32116
63. Nocito, A. *et al.* (2008) Serotonin regulates macrophage-mediated angiogenesis in a mouse model of colon cancer allografts. *Cancer Res.* 68, 5152–5158
64. Terry, N. and Margolis, K.G. (2017) Serotonergic mechanisms regulating the GI tract: experimental evidence and therapeutic relevance. *Handb. Exp. Pharmacol.* 239, 319–342
65. Li, Z. *et al.* (2011) Essential roles of enteric neuronal serotonin in gastrointestinal motility and the development/survival of enteric dopaminergic neurons. *J. Neurosci.* 31, 8998–9009
66. Gross, E.R. *et al.* (2012) Neuronal serotonin regulates growth of the intestinal mucosa in mice. *Gastroenterology* 143, 408–417
67. Heredia, D.J. *et al.* (2013) Important role of mucosal serotonin in colonic propulsion and peristaltic reflexes: *in vitro* analyses in mice lacking tryptophan hydroxylase 1. *J. Physiol.* 591, 5939–5957
68. Ghia, J.E. *et al.* (2009) Serotonin has a key role in pathogenesis of experimental colitis. *Gastroenterology* 137, 1649–1660
69. Li, N. *et al.* (2011) Serotonin activates dendritic cell function in the context of gut inflammation. *Am. J. Pathol.* 178, 662–671
70. Gross, M.K. *et al.* (2017) Enteric serotonin and oxytocin: endogenous regulation of severity in a murine model of necrotizing enterocolitis. *Am. J. Physiol. Gastrointest. Liver Physiol.* 313, G386–G398
71. Kim, J.J. *et al.* (2015) Blocking peripheral serotonin synthesis by telotristat etiprate (LX1032/LX1606) reduces severity of both chemical- and infection-induced intestinal inflammation. *Am. J. Physiol. Gastrointest. Liver Physiol.* 309, G455–G465
72. Margolis, K.G. *et al.* (2014) Pharmacological reduction of mucosal but not neuronal serotonin opposes inflammation in mouse intestine. *Gut* 63, 928–937
73. Brown, P.M. *et al.* (2011) The tryptophan hydroxylase inhibitor LX1031 shows clinical benefit in patients with nonconstipating irritable bowel syndrome. *Gastroenterology* 141, 507–516
74. Crane, J.D. *et al.* (2015) Inhibiting peripheral serotonin synthesis reduces obesity and metabolic dysfunction by promoting brown adipose tissue thermogenesis. *Nat. Med.* 21, 166–172
75. Oh, C.M. *et al.* (2015) Regulation of systemic energy homeostasis by serotonin in adipose tissues. *Nat. Commun.* 6, 6794
76. Sumara, G. *et al.* (2012) Gut-derived serotonin is a multifunctional determinant to fasting adaptation. *Cell Metab.* 16, 588–600
77. Paulmann, N. *et al.* (2009) Intracellular serotonin modulates insulin secretion from pancreatic β-cells by protein serotonylation. *PLoS Biol.* 7, e1000229
78. Kim, K. *et al.* (2015) Functional role of serotonin in insulin secretion in a diet-induced insulin-resistant state. *Endocrinology* 156, 444–452
79. Kim, H. *et al.* (2010) Serotonin regulates pancreatic beta cell mass during pregnancy. *Nat. Med.* 16, 804–808
80. Walther, D.J. *et al.* (2003) Serotonylation of small GTPases is a signal transduction pathway that triggers platelet α-granule release. *Cell* 115, 851–862

81. Ziu, E. *et al.* (2014) Effect of serotonin on platelet function in cocaine exposed blood. *Sci. Rep.* 4, 5945
82. Duerschmied, D. *et al.* (2012) Platelet serotonin promotes the recruitment of neutrophils to sites of acute inflammation in mice. *Blood* 121, 1008–1015
83. Herr, N. *et al.* (2014) Acute fluoxetine treatment induces slow rolling of leukocytes on endothelium in mice. *PLoS One* 9, e88316
84. Zhang, J. *et al.* (2015) Role of serotonin in MODS: deficiency of serotonin protects against zymosan-induced multiple organ failure in mice. *Shock* 43, 276–284
85. Zhang, J. *et al.* (2017) 5-HT drives mortality in sepsis induced by cecal ligation and puncture in mice. *Mediators Inflamm.* 2017, 6374283
86. Durk, T. *et al.* (2013) Production of serotonin by tryptophan hydroxylase 1 and release via platelets contribute to allergic airway inflammation. *Am. J. Respir. Crit. Care Med.* 187, 476–485
87. Bi, J. *et al.* (2017) Peripheral serotonin regulates postoperative intra-abdominal adhesion formation in mice. *Sci. Rep.* 7, 10001
88. Sonda, S. *et al.* (2013) Serotonin regulates amylase secretion and acinar cell damage during murine pancreatitis. *Gut* 62, 890–898
89. Chabbi-Achengli, Y. *et al.* (2016) Serotonin is involved in autoimmune arthritis through Th17 immunity and bone resorption. *Am. J. Pathol.* 186, 927–937
90. Nowak, E.C. *et al.* (2012) Tryptophan hydroxylase-1 regulates immune tolerance and inflammation. *J. Exp. Med.* 209, 2127–2135
91. Friedman, S.L. *et al.* (2013) Therapy for fibrotic diseases: nearing the starting line. *Sci. Transl. Med.* 5, 167sr1
92. Hallen, A. (1964) Fibrosis in the carcinoid syndrome. *Lancet* 1, 746–747
93. MacDonald, R.A. *et al.* (1958) Dermal fibrosis following subcutaneous injections of serotonin creatinine sulphate. *Proc. Soc. Exp. Biol. Med.* 97, 334–337
94. Fabre, A. *et al.* (2008) Modulation of bleomycin-induced lung fibrosis by serotonin receptor antagonists in mice. *Eur. Respir. J.* 32, 426–436
95. Dees, C. *et al.* (2011) Platelet-derived serotonin links vascular disease and tissue fibrosis. *J. Exp. Med.* 208, 961–972
96. Konigshoff, M. *et al.* (2010) Increased expression of 5-hydroxytryptamine2A/B receptors in idiopathic pulmonary fibrosis: a rationale for therapeutic intervention. *Thorax* 65, 949–955
97. Janssen, W. *et al.* (2015) 5-HT2 B receptor antagonists inhibit fibrosis and protect from RV heart failure. *Biomed Res. Int.* 2015, 438403
98. Welford, R.W. *et al.* (2016) Serotonin biosynthesis as a predictive marker of serotonin pharmacodynamics and disease-induced dysregulation. *Sci. Rep.* 6, 30059
99. Bazan, I.S. and Fares, W.H. (2016) Review of the ongoing story of appetite suppressants, serotonin pathway, and pulmonary vascular disease. *Am. J. Cardiol.* 117, 1691–1696
100. Herve, P. *et al.* (1990) Primary pulmonary hypertension in a patient with a familial platelet storage pool disease: role of serotonin. *Am. J. Med.* 89, 117–120
101. Sato, K. *et al.* (1992) Factors influencing the idiopathic development of pulmonary hypertension in the fawn hooded rat. *Am. Rev. Respir. Dis.* 145, 793–797
102. Eddahibi, S. *et al.* (2006) Cross talk between endothelial and smooth muscle cells in pulmonary hypertension: critical role for serotonin-induced smooth muscle hyperplasia. *Circulation* 113, 1857–1864
103. Morecroft, I. *et al.* (2007) Effect of tryptophan hydroxylase 1 deficiency on the development of hypoxia-induced pulmonary hypertension. *Hypertension* 49, 232–236
104. Izikki, M. *et al.* (2007) Tryptophan hydroxylase 1 knockout and tryptophan hydroxylase 2 polymorphism: effects on hypoxic pulmonary hypertension in mice. *Am. J. Physiol. Lung Cell. Mol. Physiol.* 293, L1045–L1052
105. Dempsie, Y. *et al.* (2008) Converging evidence in support of the serotonin hypothesis of dexfenfluramine-induced pulmonary hypertension using novel transgenic mice. *Circulation* 117, 2928–2937
106. Ciuclan, L. *et al.* (2013) Imatinib attenuates hypoxia-induced pulmonary arterial hypertension pathology via reduction in 5-hydroxytryptamine through inhibition of tryptophan hydroxylase 1 expression. *Am. J. Respir. Crit. Care Med.* 187, 78–89
107. Rothman, R.B. *et al.* (2011) Altered gene expression in pulmonary tissue of tryptophan hydroxylase-1 knockout mice: implications for pulmonary arterial hypertension. *PLoS One* 6, e17735
108. Guignabert, C. *et al.* (2006) Transgenic mice overexpressing the 5-hydroxytryptamine transporter gene in smooth muscle develop pulmonary hypertension. *Circ. Res.* 98, 1323–1330
109. Herve, P. *et al.* (1995) Increased plasma serotonin in primary pulmonary hypertension. *Am. J. Med.* 99, 249–254
110. Lesurtel, M. *et al.* (2006) Platelet-derived serotonin mediates liver regeneration. *Science* 312, 104–107
111. Nocito, A. *et al.* (2007) Platelets and platelet-derived serotonin promote tissue repair after normothermic hepatic ischemia in mice. *Hepatology* 45, 369–376
112. Jang, J.H. *et al.* (2012) Serotonin protects mouse liver from cholestatic injury by decreasing bile salt pool after bile duct ligation. *Hepatology* 56, 209–218
113. Zhang, J. *et al.* (2015) Serotonin deficiency exacerbates acetaminophen-induced liver toxicity in mice. *Sci. Rep.* 5, 8098
114. Nocito, A. *et al.* (2007) Serotonin mediates oxidative stress and mitochondrial toxicity in a murine model of nonalcoholic steatohepatitis. *Gastroenterology* 133, 608–618
115. Lang, P.A. *et al.* (2008) Aggravation of viral hepatitis by platelet-derived serotonin. *Nat. Med.* 14, 756–761
116. Yadav, V.K. *et al.* (2010) Pharmacological inhibition of gut-derived serotonin synthesis is a potential bone anabolic treatment for osteoporosis. *Nat. Med.* 16, 308–312
117. Yadav, V.K. *et al.* (2008) Lrp5 controls bone formation by inhibiting serotonin synthesis in the duodenum. *Cell* 135, 825–837
118. Yadav, V.K. *et al.* (2009) A serotonin-dependent mechanism explains the leptin regulation of bone mass, appetite, and energy expenditure. *Cell* 138, 976–989
119. Cui, Y. *et al.* (2011) LRP5 functions in bone to regulate bone mass. *Nat. Med.* 17, 684–691
120. Lam, D.D. *et al.* (2011) Leptin does not directly affect CNS serotonin neurons to influence appetite. *Cell Metab.* 13, 584–591
121. Lima, G.M. *et al.* (2016) The effect of an inhibitor of gut serotonin (LP533401) during the induction of periodontal disease. *J. Periodontal Res.* 51, 661–668
122. Cui, Y. *et al.* (2014) Reply to Lrp5 regulation of bone mass and gut serotonin synthesis. *Nat. Med.* 20, 1229–1230
123. Kode, A. *et al.* (2014) Lrp5 regulation of bone mass and serotonin synthesis in the gut. *Nat. Med.* 20, 1228–1229
124. Matsuda, M. *et al.* (2004) Serotonin regulates mammary gland development via a novel autocrine-paracrine loop. *Dev. Cell* 6, 193–203
125. Abid, S. *et al.* (2012) Inhibition of gut- and lung-derived serotonin attenuates pulmonary hypertension in mice. *Am. J. Physiol. Lung Cell. Mol. Physiol.* 303, L500–L508
126. Inose, H. *et al.* (2011) Efficacy of serotonin inhibition in mouse models of bone loss. *J. Bone Miner. Res.* 26, 2002–2011
127. Erjavec, I. *et al.* (2016) Constitutively elevated blood serotonin is associated with bone loss and type 2 diabetes in rats. *PLoS One* 11, e0150102
