# Supplementary material for: 5-Hydroxytryptophan (5-HTP): Natural Occurrence, Analysis, Biosynthesis, Biotechnology, Physiology and Toxicology
Source: Int J Mol Sci. 2020 Dec 26;22(1):181. doi: 10.3390/ijms22010181 (PMC7796270; doi:10.3390/ijms22010181)
Supplement: Supplementary file 1 [file ijms-22-00181-s001.zip › 5-HTP.Data/PDF/4067180500/Xu-2020-Enhanced-production-of--hydroxytryp.pdf]

Applied Microbiology and Biotechnology (2020) 104:2481–2488
https://doi.org/10.1007/s00253-020-10371-y

BIOTECHNOLOGICAL PRODUCTS AND PROCESS ENGINEERING

Check for updates

Enhanced production of 5-hydroxytryptophan through the regulation of L-tryptophan biosynthetic pathway

Da Xu[1] · Mengjun Fang[2,3] · Haijiao Wang[2,3] · Lei Huang[2,3] · Qinyang Xu[1] · Zhinan Xu[2,3]

Received: 20 October 2019 / Revised: 1 January 2020 / Accepted: 12 January 2020 / Published online: 31 January 2020
© Springer-Verlag GmbH Germany, part of Springer Nature 2020

Abstract
5-Hydroxytryptophan (5-HTP) is the precursor of the neurotransmitter serotonin and has been used for the treatment of various diseases such as depression, insomnia, chronic headaches, and binge eating associated obesity. The production of 5-HTP had been achieved in our previous report, by the development of a recombinant strain containing two plasmids for biosynthesis of L-tryptophan (L-trp) and subsequent hydroxylation. In this study, the L-trp biosynthetic pathway was further integrated into the *E. coli* genome, and the promoter strength of 3-deoxy-7-phosphoheptulonate synthase, which catalyzes the first step of L-trp biosynthesis, was engineered to increase the production of L-trp. Hence, the 5-HTP production could be manipulated by the regulation of copy number of L-trp hydroxylation plasmid. Finally, the 5-HTP production was increased to 1.61 g/L in the shaking flasks, which was 24% improvement comparing to the original producing strain, while the content of residual L-trp was successfully reduced from 1.66 to 0.2 g/L, which is beneficial for the downstream separation and purification. Our work shall promote feasible progresses for the industrial production of 5-HTP.

Keywords 5-Hydroxytryptophan · *E. coli* · Plasmid copy number · Promoter engineering

Introduction

5-Hydroxytryptophan (5-HTP) is the direct precursor of the neurotransmitter serotonin. Serotonergic dysfunction in the central nervous system (CNS) has been implicated in the etiology of depression (Turner et al. 2006), while depression is a prevalent mental disorder with high disability and heavy disease burden, which affected 4.4% of the world's population and led to ~ 1 million suicides per year (World Health Organization 2017). 5-HTP can easily pass through the blood-brain barrier and increase serotonin supplied by oral administration, therefore relieving the symptom of depression. In addition, previous reports have showed that the 5-HTP administration was also effective in treating fibromyalgia, obesity, chronic headaches, and insomnia (Birdsall 1998).

5-HTP is natively produced in humans and animals via the hydroxylation of L-trp (L-trp) catalyzed by tryptophan hydroxylase (TPH). As the essential cofactor of TPH (Fitzpatrick 2003), tetrahydrobiopterin (BH4) is synthesized from GTP through a three-step pathway catalyzed by GTP cyclohydrolase I (GCHI), 6-pyruvate-tetrahydropterin synthase (PTPS), and sepiapterin reductase (SPR) and is oxidized to pterin-4α-carbinolamine (BH3OH) during the hydroxylation of L-trp. Meanwhile, BH3OH could be regenerated to BH4 by the catalysis of pterin-4α-carbinolamine dehydratase (PCD) and dihydropteridine reductase (DHPR) (Hara and Kino 2013).

Without effective chemical approach, the current commercial resource of 5-HTP is through the extraction from

Electronic supplementary material The online version of this article (https://doi.org/10.1007/s00253-020-10371-y) contains supplementary material, which is available to authorized users.

✉ Haijiao Wang
willyw@zju.edu.cn

✉ Zhinan Xu
znxu@zju.edu.cn

[1] College of Bioengineering, Tianjin University of Science & Technology, Tianjin, China

[2] Key Laboratory of Biomass Chemical Engineering (Education Ministry), College of Chemical and Biological Engineering, Zhejiang University, Hangzhou 310027, China

[3] Institute of Biological Engineering, College of Chemical and Biological Engineering, Zhejiang University, Hangzhou 310027, China

the seeds of African plant *Griffonia simplicifolia*. However, such plant origin raw materials are seasonally and regionally dependent, which limited efficient production and applications of 5-HTP. Various studies have been reported for the engineering of microbial production of 5-HTP. The recombinant mammalian TPHs and mutated prokaryotic L-phenylalanine-4-hydroxylases (PAHs) could functionally hydroxylate L-trp in the presence of BH4, O_2, and Fe^{2+} (Nielsen et al. 2008; Windahl et al. 2009; Carkaci-Salli et al. 2006; Murphy et al. 2008; Kino et al. 2009), which could be further enhanced by the co-expression of BH4 regeneration pathway (Hara and Kino 2013). The introduction of the three-step BH4 biosynthetic pathway allowed the recombinant *E. coli* cells to convert L-trp into 5-HTP in the absence of external BH4 (Knight et al. 2013). In addition to BH4, the prokaryotic PAHs could utilize tetrahydromonapterin (MH4), which is natively produced by *E. coli* (Ikemoto et al. 2002), as the pterin coenzyme instead of BH4 (Pribat et al. 2010). Co-expression of PAHs and pterin regeneration pathway could also achieve efficient hydroxylation of L-trp in *E. coli* (Mora-Villalobos and Zeng 2017; Lin et al. 2014), and the de novo synthesis of 5-HTP could be realized by coupling the L-trp biosynthetic pathway and the hydroxylation pathway (Lin et al. 2014; Mora-Villalobos and Zeng 2018). However, the mediocre yields of 5-HTP (no more than 160 mg/L in shaking flasks) and the relative high accumulation of L-trp in these works are still unable to meet the demands of industrial-scale 5-HTP production.

In our previous report, we have developed a recombinant *E. coli* strain with a high 5-HTP-producing capacity (Wang et al. 2018), which produced 1.29 g/L and 5.1 g/L 5-HTP in shake flask and fed-batch fermentation, respectively. Similarly, this strain also accumulated a high amount of L-trp (1.66 g/L in shake flask and 8.6 g/L in fed-batch fermentation), and the hydroxylation rate was decreased at the late fermentation stage because of the low stability of hydroxylation pathway plasmid pACHTP-LMT. In this study, the L-trp biosynthetic pathway was integrated into the *E. coli* genome in order to increase the stability of hydroxylation pathway. The copy number of hydroxylation plasmid was regulated by manipulating its replication origin, and the promoter of $aroH^{fbr}$ gene encoding 3-deoxy-7-phosphoheptulonate synthase (catalyzes the first step of L-trp biosynthesis) was substituted. The resulted recombinant strain TRPmut/pSCHTP-LMT was able to produce 1.61 g/L 5-HTP in shake flasks, which presented a 24.8% improvement comparing to the original strain. Besides, the concentration of L-trp was significantly decreased to 0.25 g/L, and the concentration ratio between 5-HTP and L-trp increased from 0.78:1 to 6.44:1, which shall facilitate the downstream isolation and purification of 5-HTP.

Methods and materials

Strains and media

The bacterial strains and plasmids used in this study were listed in Table S1. Primers were listed in Table S2. *E. coli* DH5α was used for plasmid construction and propagation. *E. coli* BL21ΔtnaA was used as the host for 5-HTP production. Gene deletions and insertions were performed using CRISPR-Cas9 system according to the published protocol (Jiang et al. 2015). Luria-Bertani (LB) medium, containing 10 g/L tryptone, 5 g/L yeast extract, and 10 g/L sodium chloride, was used for cultivation. Glycerol mineral medium was used for 5-HTP production (Wang et al. 2018). When necessary, the medium was supplemented with kanamycin, spectinomycin, ampicillin, or chloramphenicol at appropriate concentrations. L-trp from Sangon Biotech (Shanghai, China) and 5-HTP from Aladdin (Shanghai, China) were used as standards. Other reagents were of analytical purity.

Genome integration of L-trp biosynthetic pathway

The upstream and downstream homologous arms of *aroH* gene were amplified using primer pairs aroH-up-F/aroH-up-R and aroH-down-F1/aroH-down-R, respectively, and fused by PCR. The *aro*H-sgRNA expression plasmid was amplified with primer pair aroH-sgRNA1-F/aroH-sgRNA1-R and circularized using Gibson Assembly to construct pTarget-aroH1. The *aroH* homologous arm and pTarget-aroH1 were transformed into BL21ΔtnaA/pCas by electric shock to knock out *aroH* (Jiang et al. 2015).

Primer pairs aroH-up-F/aroH-up-R, aroH-down-F2/aroH-down-R, and tac-aroH-F/tac-aroH-R were used to amplify the upstream and downstream homologous arms and $aroH^{fbr}$ gene with tac promoter (tac_p), respectively, and fused by PCR. Primer pair aroH-sgRNA2-F/aroH-sgRNA2-R was used to amplify the sgRNA plasmid and circularized using Gibson Assembly to construct pTarget-aroH2. The homologous-armed tac_p-$aroH^{fbr}$ fragment and pTarget-aroH2 were transformed into BL21ΔtnaAΔaroH/pCas by electric shock to insert $aroH^{fbr}$ into the original *aroH* gene locus (Jiang et al. 2015). The genomic *trpE* gene was substituted with $trpE^{fbr}$ following the same procedure.

Regulation of hydroxylation plasmid copy number

The L-trp hydroxylation pathway fragment was amplified from plasmid pACHTP-LMT with primer pair HTP-F/HTP-R and circularized with pSC101 fragment (containing pSC101ori and the ampicillin resistant gene) or the pET fragment (containing pBR322ori and the ampicillin resistant gene) via Gibson Assembly to construct pSCHTP-LMT or pETHTP-LMT. The pSC101 fragment was amplified from

pSA101trp using primer pair pSC101-F/pSC101-R, and the pET fragment was amplified from pETDuet-1 using primer pair pET-F/pET-R.

Promoter substitution of *aroH*^{fbr}

Primer pairs tac-aroH-up-F/tac-aroH-up-R and tac-aroH-down-F/tac-aroH-down-R were used to amplify the upstream and downstream homologous arm of tac_p of *aroH*^{fbr} from the genome of the *E. coli* strain TRP0. M1 promoters (Lu et al. 2012) were amplified using M1promoter-F/M1promoter-R. The homologous arms and M1 promoters were fused by PCR. Promoter tac_p was substituted using CRISPR-Cas9 system. The sgRNA plasmid pTarget-tac was constructed with primer pair tac-sgRNA-F/tac-sgRNA-R.

Cell cultivation

Cell cultivation conditions were the same as previously reported (Wang et al. 2018). *E. coli* stains were first plated on LB agar plates. Single colonies were inoculated into 5 mL LB media and incubated at 37 °C, 200 rpm overnight. About 1 mL of such cultures were transformed into 30 g/L glycerol mineral medium and cultivated at 37 °C, 200 rpm until OD600 reached 4.5~5. Then, 5-HTP production was induced by adding 0.01 mM IPTG into the culture. The culture was further cultivated at 30 °C, 200 rpm. OD600 values were sampled at regular intervals, and the contents of 5-HTP and L-trp in the fermentation liquid were determined by HPLC using the previously reported protocol (Wang et al. 2018).

Plasmid stability determination

The fermentation culture was diluted by an appropriate factor and plated on LB agar plates and incubated at 37 °C for 24 h. About 100 single colonies were picked to inoculate LB plates with or without corresponding antibiotics and incubated at 37 °C for 24 h. The number of colonies on the antibiotic plate and the nonantibiotic plate was compared to access the plasmid stability.

Results

Integration of L-trp biosynthetic pathway into *E. coli* genome

In the fed-batch fermentation of 5-HTP production, the 5-HTP productivity of HTP101-LMT cells was significantly reduced at the late fermentation stage, while the production of L-trp was increased along with the cell density and remained stable till the end of fermentation. The stability of pSA101trp was found to be 100% during the fermentation process, while pACHTP-LMT was unstable at the late fermentation period (Table S3), which may be the main cause of the decreased productivity of 5-HTP .

To test if the double plasmids expression system may be responsible for the low stability of plasmid pACHTP-LMT, pACHTP-LMT was transformed into BL21ΔtnaA alone and performed the same fermentation process to detect its stability. The result showed that pACHTP-LMT maintained good retention in BL21ΔtnaA cells throughout the fermentation (Table S4). Based on this, the L-trp biosynthetic pathway was chosen to be integrated into the genome of BL21ΔtnaA, in order to maintain the stability of pACHTP-LMT and reduce the accumulation L-trp.

The native L-trp biosynthetic pathway is strictly regulated by L-trp feedback inhibition on the transcription of L-trp operon, which directly affects the expressions and activities of 3-deoxy-7-phosphoheptulonate synthase (encoded by *aroH*) and anthranilate synthase (encoded by *trpE*), and such inhibition could be released by the substitution of promoters in feedback-resistant mutant (*aroH*^{fbr} and *trpE*^{fbr}). Thus, the native *aroH* and *trpE* in the BL21ΔtnaA genome were replaced with *aroH*^{fbr} and *trpE*^{fbr} under the control of tac_p, respectively. The resulted strain TRP0 showed enhanced production of both L-trp and 5-HTP comparing to BL21ΔtnaA. With the transformation of pACHTP-LMT (Fig. 1), the growth of TRP0/pACHTP-LMT in 30 g/L glycerol mineral medium was similar to that of HTP101-LMT; the final cell density after 90-h cultivation was obtained as OD600 = 16.04 and OD600 = 15.97, respectively. However, the production of L-trp (0.8 g/L) and 5-HTP (0.92 g/L) in TRP0/pACHTP-LMT was decreased comparing to those in HTP101-LMT (1.54 g/L L-trp and 1.21 g/L 5-HTP). On the other hand, the plasmid retention rate of pACHTP-LMT was still as high as 91.4% (Table 1), which was beneficial for the hydroxylation pathway and led to the higher content of 5-HTP than that of L-trp (Fig. 1).

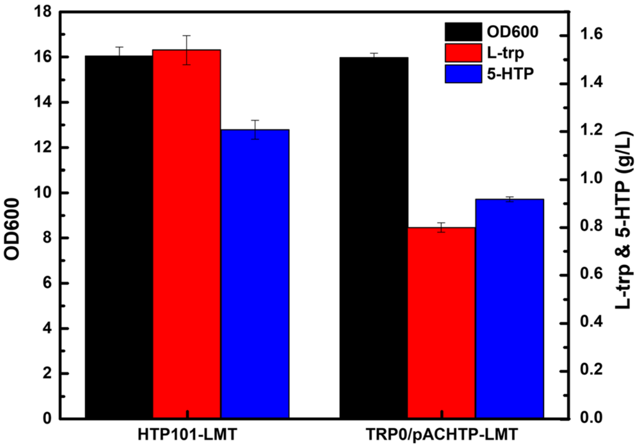

Fig. 1 Production of 5-HTP with *E. coli* strains HTP101-LMT and TRP0/pACHTP-LMT

Table 1 Plasmid stability of TRP0/pACHTP-LMT cells during the fermentation

Fermentation time	Colonies on Cm plate	Colonies on control plate	Plasmid stability
11 h	106	108	98.1%
48 h	93	106	88.6%
90 h	96	105	91.4%

Regulation of hydroxylation plasmid copy number to improve L-trp hydroxylation

To increase the hydroxylation rate of L-trp, the replication origin of pACHTP-LMT was replaced with different origins to regulate the copy number of hydroxylation plasmid (Table 2). The comparison suggested that the growth pressure of strain seemed to increase accompanying with the higher copy number of hydroxylation plasmid (Fig. 2). TRP0/pSCHTP-LMT showed the highest and steadily increased growth rate during the entire fermentation process, while the growth rate of TRP0/pACHTP-LMT was similarly increased as that of TRP0/pSCHTP-LMT in the early period but slowed down after then. The growth rate of TRP0/pETHTP-LMT was the lowest among all three strains. At the end of fermentation, the OD600 values of three strains were 22.47, 19.98, and 13.31, respectively (Fig. 2A). On the other hand, the increased copy number of hydroxylation plasmid evidently reduced the hydroxylation rate of L-trp in TRP0/pETHTP-LMT, only 0.4 g/L 5-HTP was obtained, which was half of that of TRP0/pACHTP-LMT (Fig. 2C). In contrast, reducing the copy number of hydroxylation pathway not only enhanced cell growth but also increased the hydroxylation efficiency of L-trp. After the induction, the content of 5-HTP in TRP0/pSCHTP-LMT was accumulated at almost twice of that in TRP0/pACHTP-LMT and reached the maximum yield of 1.28 g/L at 84 h (Fig. 2C). Meanwhile, the content of L-trp was maintained at a low level, and only 0.29 g/L L-trp was produced at the end of fermentation (Fig. 2B). Hence, TRP0/pSCHTP-LMT not only restored the 5-HTP production level but also successfully reduced the accumulation of L-trp, with the 5-HTP/L-trp ratio of 3.8:1. The results demonstrated that the vigorous cell growth was more beneficial to the L-trp hydroxylation efficiency, possibly because of more sufficient substrates (GTP, BH4) and energy supply (ATP, NADH, NADPH) for the reaction.

Table 2 The replication origins and copy numbers of three L-trp hydroxylation plasmids

Plasmids	Replication origin	Copy number
pSCHTP-LMT	pSC101ori	~5
pACHTP-LMT	p15Aori	~15
pETHTP-LMT	pBR322ori	~40

Replacement of the genome *aroH*^{fbr} gene promoter to regulate L-trp accumulation

DAHP synthase encoded by *aroH*^{fbr} is the key enzyme catalyzing the first step of L-trp synthesis, in which 2-dehydro-3-deoxy-D-arabino-heptonate-7-phosphate (DAHP) is

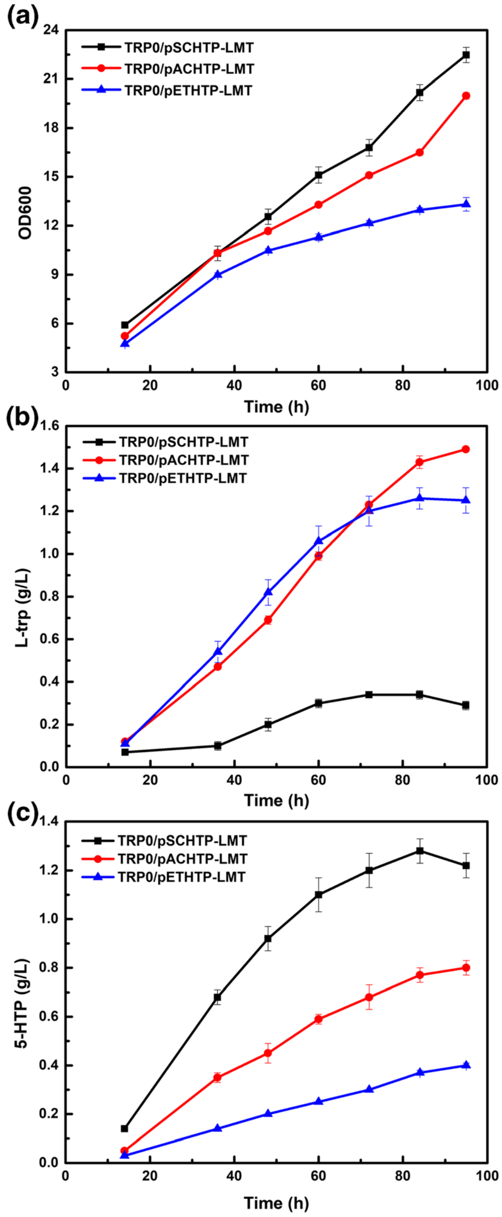

Fig. 2 Effects of copy numbers of L-trp hydroxylation plasmid on (A) cell growth, (B) L-trp, and (C) 5-HTP production

synthesized from phosphoenolpyruvate (PEP) and D-Erythrose-4-phosphate. It was reported that the high-intensity expression of *aroH^{fbr}* gene may result in a decrease in intracellular PEP concentration and attenuating the intensity of central metabolic pathway in cell, thus affecting the biosynthesis of substances and energy required for cell growth (Ran and Frost 2007; Cui et al. 2019). Therefore, the overexpression of *aroH^{fbr}* gene seems unfavorable to cell growth and possibly affects the biosynthesis of L-trp and 5-HTP. To further regulate the strength of L-trp biosynthetic pathway, the tac promoter in front of the *aroH^{fbr}* gene was respectively replaced by five promoters with different strengths (Table 3) (Lu et al. 2012). As shown in Fig. 3A, the cell growth was negatively correlated with the strength of promoter, only except M1–37. Since the expression intensities of M1 series promoters were varied in different media and dissolved oxygen conditions (Lu et al. 2012), the less effect of M1–37 promoter on the cell growth may be related to its low expression intensity in glycerol medium.

Besides cell growth, the promoter substitution also exerted significant impact on the production of L-trp and 5-HTP (Fig. 3B, C). Comparing to TRP0/pSCHTP-LMT, only TRP12/pSCHTP-LMT presented better performance on cell growth and 5-HTP production, due to the lowest promoter strength of M1–12, while other four mutants all showed reduced production of both L-trp and 5-HTP in fermentation. As a result, the maximum content of 1.19 g/L 5-HTP was obtained from TRP12/pSCHTP-LMT after 70-h fermentation, which was a 12.3% increase than that from TRP0/pSCHTP-LMT (1.06 g/L) (Fig. 3C). Moreover, the concentration of L-trp in the fermentation broth of TRP12/pSCHTP-LMT was further reduced to 0.15 g/L, a 50% decrease comparing to that in TRP0/pSCHTP-LMT (Fig. 3B). The production ratio of 5-HTP/L-trp in the fermentation of TRP12/pSCHTP-LMT was 7.67: 1, which greatly facilitated the subsequent separation and purification of 5-HTP.

Interestingly, a mutant of TRP12 named TRPmut, which possessed three consecutive base mutations in the flanking region between the M1–12 promoter sequence and the ribosome-binding site (RBS) sequence (Fig. 4 A), was isolated during the promoter substitution. The cell growth and L-trp biosynthesis in mutant strain TRPmut/pSCHTP-LMT were similar to those of TRP12/pSCHTP-LMT, but the 5-HTP productivity was further improved to 1.61 g/L, which was 16.7% and 32% higher than that in TRP12/pSCHTP-LMT and TRP0/pSCHTP-LMT, respectively, and the production ratio of 5-HTP/L-trp was increased to 8:1 (Fig. 5). The translation initiation rate of *aroH^{fbr}* gene in either TRP12 or TRPmut was calculated based on the RBS Calculator (https://salislab.net/software/reverse) (Espah Borujeni et al. 2014), in which the translational efficiency score of TRP12 was 849.0 and 222.7 for TRPmut. Hence, the further reduced expression intensity of *aroH^{fbr}* gene in TRPmut could contribute to the additional improvement of 5-HTP production.

Discussion

The microbial production of 5-HTP via the hydroxylation of L-trp often results in high accumulation of L-trp intermediate (Lin et al. 2014; Mora-Villalobos and Zeng 2018; Wang et al. 2018), which was sometimes even more than 20 times higher than the concentration of 5-HTP (Mora-Villalobos and Zeng 2018). Furthermore, the very similar structures of L-trp and 5-HTP, which are only different at a hydroxyl group, also bring difficulties to the downstream purification of 5-HTP. Based on our previous work for the 5-HTP production via a two-plasmid system (Wang et al. 2018), some improvements were

Table 3 Sequences and relative strengths of M1 promoters

Name	Sequence*	Strength**
lacZ	**TTTACA**CTTTATGCTTCCGGCTCG**TATGTTG**TGTGGAATTGTGAG CGGATAACAATTTCACACAGGAAACAGCT	0.3
M1–12	TTATCTCTGGCGGTG**TTGACA**AGAGATAACAACGTTGA**TATAAT** TGAGCCCTTTTGGTGCGTCAGTCAGTTTAAACCAGGAAACAGCT	0.1 ± 0.01
M1–30	TTATCTCTGGCGGTG**TTGACA**AGAGATAACAACGTTGA**TATAAT** TGAGCCTGAGGTGGCTTATTATTCGTTTAAACCAGGAAACAGCT	0.7 ± 0.04
M1–37	TTATCTCTGGCGGTG**TTGACA**AGAGATAACAACGTTGA**TATAAT** TGAGCCACTGGCTCGTAATTTATTGTTTAAACCAGGAAACAGCT	1.9 ± 0.12
M1–46	TTATCTCTGGCGGTG**TTGACA**AGAGATAACAACGTTGA**TATAAT** TGAGCCTCTCGCCCCACCAATTCGGTTTAAACCAGGAAACAGCT	1.5 ± 0.1
M1–93	TTATCTCTGGCGGTG**TTGACA**AGAGATAACAACGTTGA**TATAAT** TGAGCCCGTATTGTTAGCATGTACGTTTAAACCAGGAAACAGCT	4.0 ± 0.12

* Bold: −35 region and −10 region of the promoter. Underlined: flanking sequence between promoters and RBS sequence

** The promoter strength was represented as the relative strength in glucose media and aerobic conditions, where the strength of induced lacZ promoted by in LB medium and aerobic conditions (Lu et al. 2012)

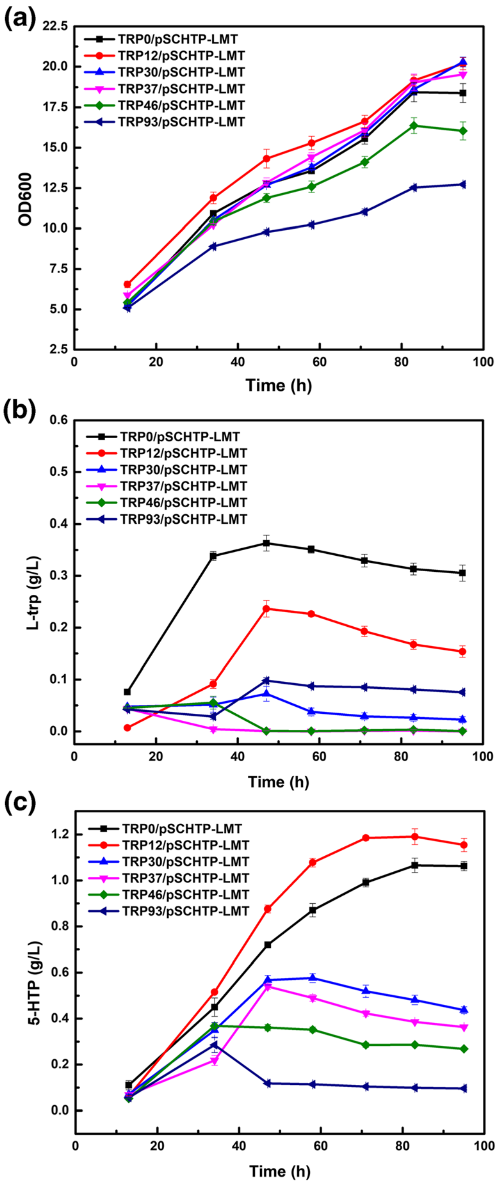

Fig. 3 Effects of promoter substitution of *aroH*^{fbr} on (A) cell growth, (B) L-trp, and (C) 5-HTP production

addressed in this study. First, the L-trp biosynthetic pathway was integrated into the genome of *E. coli* to reduce L-trp accumulation and also provided a major advantage to stabilize the L-trp hydroxylation plasmid pACHTP-LMT, whose low stability caused the decrease of 5-HTP production in the late fermentation stage. However, the final productivity of 5-HTP in such system was even lower than that in the original two-

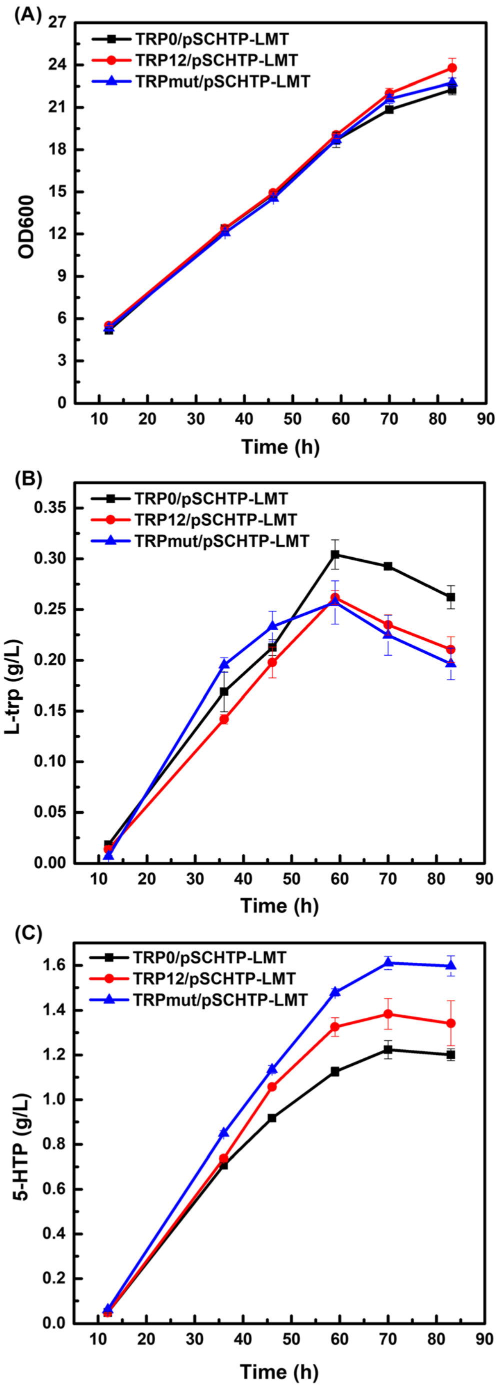

Fig. 5 Comparison of (**A**) cell growth, (B) L-trp, and (**C**) 5-HTP production among TRP0/pSCHTP-LMT, TRP12/pSCHTP-LMT, and TRPmut/pSCHTP-LMT

M1-12 promoter: TTATCTCTGGCGGTG**TTGACAAGAGATAACAACGTTGATATAAT**TGAGCCCTTTT
Mutant promoter: TTATCTCTGGCGGTG**TTGACAAGAGATAACAACGTTGATATAAT**TGAGCCCTTTT

M1-12 promoter: GGTGCGTCAGTCAGTTTAAACC**AGGAAACAGCT**ATGAATTATCAG
Mutant promoter: GGTGCGT***TGT***TCAGTTTAAACC**AGGAAACAGCT**ATGAATTATCAG

Fig. 4 A sequence comparison between the mutant promoter and M1–12 promoter. Green, M1–12 promoter sequence; blue, RBS sequence; yellow, initial coding sequence for *aroH*^{fbr}; framed, mutation sites

plasmid system, probably due to the evidently reduced production of L-trp. Fortunately, the low-copy-number hydroxylation plasmid pSCHTP-LMT provided a better balance to the metabolic change between L-trp and 5-HTP and thus showed better performance in both cell growth and 5-HTP production, with significantly reduced accumulation of L-trp (Fig. 2). In addition, the manipulation of *aroH*^{fbr} expression strength through promoter substitution also regulated the L-trp biosynthetic pathway, in which the lowest expression of *aroH*^{fbr} was also beneficial to cell growth and 5-HTP production. Therefore, the synchronization of metabolic pathways seemed more important to balanced production of final product and intermediates.

Plasmid-copy-number modulation is often used for the balancing and optimization of metabolic pathways for higher productions (Lim et al. 2016, Xu et al. 2013). We expected that a high-copy number of L-trp hydroxylation pathway would help improve the conversion of L-trp to 5-HTP, thus increasing 5-HTP production. However, TRP0/pETHTP-LMT showed both decreased cell growth and L-trp and 5-HTP production compared with TRP0/pACHTP-LMT (Fig. 2). On the other hand, a low-copy-number plasmid pSCHTP-LMT led to better cell growth and higher 5-HTP production, indicating that lower expression of L-trp hydroxylation pathway was sufficient to convert L-trp to 5-HTP. Xu et al. (2013) obtained similar results with higher fatty acid production using low-copy number plasmids for the expression of the ACA module. These results demonstrated that higher expression intensity was not always necessary for high metabolite production (Keasling 2010).

Gene *aroH*^{fbr} encodes DAHP synthase, which catalyzed synthesis of DAHP from PEP and D-Erythrose-4-phosphate. Since PEP is an important intermediate in central carbon flux, which is the key pathway for substance and energy supplementation for cell metabolism, excessive withdraw of PEP from the central pathway may cause insufficient support for cell growth. Indeed, we observed decreased cell growth as the promoter strength increased for *aroH*^{fbr} (Fig. 3A). This in turn affected both L-trp and 5-HTP synthesis which are both substance and energy extensive procedure. The decreased L-trp and 5-HTP production demonstrated the negative effect of *aroH*^{fbr} been expressed under stronger promoters (Fig. 3). Thus, regulation of *aroH*^{fbr} demonstrated a critical role for balancing cell metabolism and 5-HTP synthesis. Since the translational efficiency of *aroH*^{fbr} gene in TRPmut was further reduced compared with TRP12, which could result in decreased overall DAHP synthase activity and more PEP through TCA cycle, TRPmut would be able to provide more energy and substance for L-trp synthesis and hydroxylation.

Through these efforts, 1.61 g/L 5-HTP and 0.20 g/L L-trp were produced in the final mutant strain TRPmut/pSCHTP-LMT, which represented a 24.8% improvement of 5-HTP production and an 88% decrease of L-trp, respectively, comparing with those in the starting strain HTP101-LMT (Wang et al. 2018). In addition, the 5-HTP/L-trp ratio was significantly increased from 0.78:1 to 8:1, which would be very favorable to scaled up production of 5-HTP and subsequent purification process. This work provided further progresses to achieve the industrial microbial production of 5-HTP.

Funding information This work was financially supported by the National Natural Science Foundation of China (Nos. 21576232, 21606205, and 21808199), the National Key Research and Development Program of China (No. 2018YFC1603900), and the Natural Science Foundation of Zhejiang Province (No. LY18B060002).

Compliance with ethical standards

Conflict of interest All authors confirm that they have no conflict of interests.

Human and animal rights This article does not contain any studies with human participants or animals performed by any of the authors.

References

Birdsall TC (1998) 5-Hydroxytryptophan: a clinically-effective serotonin precursor. Altern Med Rev 3(4):271–280

Carkaci-Salli N, Flanagan JM, Martz MK, Salli U, Walther DJ, Bader M, Vrana KE (2006) Functional domains of human tryptophan hydroxylase 2 (hTPH2). J Biol Chem 281(38):28105–28112. https://doi.org/10.1074/jbc.M602817200

Cui D, Deng A, Bai H, Yang Z, Liang Y, Liu Z, Qiu Q, Wang L, Liu S, Zhang Y, Shi Y, Qi J, Wen T (2019) Molecular basis for feedback inhibition of tyrosine-regulated 3-deoxy-d-arabino-heptulosonate-7-phosphate synthase from *Escherichia coli*. J Struct Biol 206(3):322–334. https://doi.org/10.1016/j.jsb.2019.04.001

Espah Borujeni A, Channarasappa AS, Salis HM (2014) Translation rate is controlled by coupled trade-offs between site accessibility, selective RNA unfolding and sliding at upstream standby sites. Nucleic Acids Res 42(4):2646–2659. https://doi.org/10.1093/nar/gkt1139

Fitzpatrick PF (2003) Mechanism of aromatic amino acid hydroxylation. Biochemistry 42(48):14083–14091. https://doi.org/10.1021/bi035656u

Hara R, Kino K (2013) Enhanced synthesis of 5-hydroxy-l-tryptophan through tetrahydropterin regeneration. AMB Express 3(1):70. https://doi.org/10.1186/2191-0855-3-70

Ikemoto K, Sugimoto T, Murata S, Tazawa M, Nomura T, Ichinose H, Nagatsu T (2002) (6R)-5,6,7,8-tetrahydro-L-monapterin from *Escherichia coli*, a novel natural unconjugated tetrahydropterin. Biol Chem 383(2):325–330. https://doi.org/10.1515/BC.2002.035

Jiang Y, Chen B, Duan C, Sun B, Yang J, Yang S (2015) Multigene editing in the *Escherichia coli* genome via the CRISPR-Cas9 system. Appl Environ Microb 81(7):2506–2514. https://doi.org/10.1128/AEM.04023-14

Keasling JD (2010) Manufacturing molecules through metabolic engineering. Science 330(6009):1355–1358. https://doi.org/10.1126/science.1193990

Kino K, Hara R, Nozawa A (2009) Enhancement of L-tryptophan 5-hydroxylation activity by structure-based modification of L-phenylalanine 4-hydroxylase from *Chromobacterium violaceum*. J Biosci Bioeng 108(3):184–189. https://doi.org/10.1016/j.jbiosc.2009.04.002

Knight EM, Zhu J, Förster J, Luo H (2013) Microorganisms for the production of 5-hydroxytryptophan. WO2013127914A1
Lim HG, Noh MH, Jeong JH, Park S, Jung GY (2016) Optimum rebalancing of the 3-hydroxypropionic acid production pathway from glycerol in *Escherichia coli*. ACS Synth Biol 5(11):1247–1255. https://doi.org/10.1021/acssynbio.5b00303
Lin Y, Sun X, Yuan Q, Yan Y (2014) Engineering bacterial phenylalanine 4-hydroxylase for microbial synthesis of human neurotransmitter precursor 5-hydroxytryptophan. ACS Synth Biol 3(7):497–505. https://doi.org/10.1021/sb5002505
Lu J, Tang J, Liu Y, Zhu X, Zhang T, Zhang X (2012) Combinatorial modulation of galP and glk gene expression for improved alternative glucose utilization. Appl Microbiol Biotechnol 93(6):2455–2462. https://doi.org/10.1007/s00253-011-3752-y
Mora-Villalobos JA, Zeng AP (2018) Synthetic pathways and processes for effective production of 5-hydroxytryptophan and serotonin from glucose in *Escherichia coli*. J Biol Eng 12:3. https://doi.org/10.1186/s13036-018-0094-7
Mora-Villalobos JA, Zeng AP (2017) Protein and pathway engineering for the biosynthesis of 5-hydroxytryptophan in *Escherichia coli*. Eng Life Sci 17:892–899. https://doi.org/10.1002/elsc.201700064
Murphy KL, Zhang X, Gainetdinov RR, Beaulieu JM, Caron MG (2008) A regulatory domain in the N terminus of tryptophan hydroxylase 2 controls enzyme expression. J Biol Chem 283(19):13216–13224. https://doi.org/10.1074/jbc.M706749200
Nielsen MS, Petersen CR, Munch A, Vendelboe TV, Boesen J, Harris P, Christensen HE (2008) A simple two step procedure for purification of the catalytic domain of chicken tryptophan hydroxylase 1 in a form suitable for crystallization. Protein Expr Purif 57(2):116–126. https://doi.org/10.1016/j.pep.2007.10.016
World Health Organization (2017) Depression and other common mental disorders: global health estimates. https://apps.who.int/iris/handle/10665/254610
Pribat A, Blaby IK, Lara-Nunez A, Gregory JF, Crecy-Lagard V, Hanson AD (2010) FolX and FolM are essential for tetrahydromonapterin synthesis in *Escherichia coli* and *Pseudomonas aeruginosa*. J Bacteriol 192(2):475–482. https://doi.org/10.1128/JB.01198-09
Ran N, Frost JW (2007) Directed evolution of 2-keto-3-deoxy-6-phosphogalactonate aldolase to replace 3-deoxy-d-arabino-heptulosonic acid 7-phosphate synthase. J Am Chem Soc 129(19):6130–6139. https://doi.org/10.1021/ja067330p
Turner EH, Loftis JM, Blackwell AD (2006) Serotonin a la carte: supplementation with the serotonin precursor 5-hydroxytryptophan. Pharmacol Ther 109(3):325–338. https://doi.org/10.1016/j.pharmthera.2005.06.004
Wang H, Liu W, Shi F, Huang L, Lian J, Qu L, Cai J, Xu Z (2018) Metabolic pathway engineering for high-level production of 5-hydroxytryptophan in *Escherichia coli*. Metab Eng 48:279–287. https://doi.org/10.1016/j.ymben.2018.06.007
Windahl MS, Boesen J, Karlsen PE, Christensen HE (2009) Expression, purification and enzymatic characterization of the catalytic domains of human tryptophan hydroxylase isoforms. Protein J 28(9–10):400–406. https://doi.org/10.1007/s10930-009-9207-y
Xu P, Gu Q, Wang W, Wong L, Bower A, Collins CH, Ma K (2013) Modular optimization of multi-gene pathways for fatty acids production in *E. coli*. Nat Commun 4(1):1409. https://doi.org/10.1038/ncomms2425

Publisher's note Springer Nature remains neutral with regard to jurisdictional claims in published maps and institutional affiliations.
