# Supplementary material for: 5-Hydroxytryptophan (5-HTP): Natural Occurrence, Analysis, Biosynthesis, Biotechnology, Physiology and Toxicology
Source: Int J Mol Sci. 2020 Dec 26;22(1):181. doi: 10.3390/ijms22010181 (PMC7796270; doi:10.3390/ijms22010181)
Supplement: Supplementary file 1 [file ijms-22-00181-s001.zip › 5-HTP.Data/PDF/4223652276/nihms227565.pdf]

NIH Public Access
Author Manuscript
Biochemistry. Author manuscript; available in PMC 2011 September 7.

Published in final edited form as:
Biochemistry. 2010 September 7; 49(35): 7563–7571. doi:10.1021/bi100744r.

Single Turnover Kinetics of Tryptophan Hydroxylase: Evidence for a New Intermediate in the Reaction of the Aromatic Amino Acid Hydroxylases

Jorge Alex Pavon[‡], **Bekir Eser**[||], **Michaela T. Huynh**[‡], and **Paul F. Fitzpatrick**[‡,§,*]

[‡] Department of Biochemistry and Biophysics, Texas A&M University, College Station TX 77843-2128

[||] Department of Chemistry, Texas A&M University, College Station TX 77843-2128

[§] Department of Biochemistry and Center for Biomedical Neuroscience, University of Texas Health Science Center at San Antonio, San Antonio, TX 78229-3900, and Department of Chemistry, University of Texas at San Antonio, San Antonio, TX 78249-1644

Abstract

Tryptophan hydroxylase (TrpH) uses a non-heme mononuclear iron center to catalyze the tetrahydropterin-dependent hydroxylation of tryptophan to 5-hydroxytryptophan. The reactions of the TrpH·Fe(II), TrpH·Fe(II)·tryptophan, TrpH·Fe(II)·6MePH$_4$·tryptophan, and TrpH·Fe(II)·6MePH$_4$·phenylalanine complexes with O_2 were monitored by stopped-flow absorbance spectroscopy and rapid quench methods. The second-order rate constant for the oxidation of TrpH·Fe(II) has a value of 104 $M^{-1}s^{-1}$ irrespective of the presence of tryptophan. Stopped-flow absorbance analyses of the reaction of the TrpH·Fe(II)·6MePH$_4$·tryptophan complex with oxygen are consistent with the initial step being reversible binding of oxygen, followed by the formation with a rate constant of 65 s^{-1} of an intermediate I that has maximal absorbance at 420 nm. The rate constant for decay of I, 4.4 s^{-1}, matches that for formation of the 4a-hydroxypterin product monitored at 248 nm. Chemical-quench analyses show that 5-hydroxytryptophan forms with a rate constant of 1.3 s^{-1}, and that overall turnover is limited by a subsequent slow step, presumably product release, with a rate constant of 0.2 s^{-1}. All of the data with tryptophan as substrate can be described by a five-step mechanism. In contrast, with phenylalanine as substrate, the reaction can be described by three steps: a second-order reaction with oxygen to form I, decay of I as tyrosine forms, and slow product release.

Tryptophan hydroxylase (TrpH)1 catalyzes the formation of 5-hydroxytryptophan (5-HO-trp) from tryptophan, the first and rate-limiting step in the biosynthesis of melatonin and serotonin (1,2). The enzyme belongs to the family of aromatic amino acid hydroxylases that also includes phenylalanine hydroxylase (PheH) and tyrosine hydroxylase (TyrH) (3). These three enzymes catalyze the hydroxylation of their corresponding substrates utilizing a tetrahydropterin and molecular oxygen (Scheme 1) (4–6). While the physiological reactions of PheH, TyrH, and TrpH are all aromatic hydroxylations, these enzymes will also catalyze benzylic and aliphatic hydroxylation (7–9). The eukaryotic forms of each enzyme are

*Address correspondence to: Paul F. Fitzpatrick, Department of Biochemistry, MC 7760, University of Texas Health Science Center at San Antonio, San Antonio, TX 78229-3900, fitzpatrick@biochem.uthscsa.edu, ph: 210-567-8264; fax: 210-567-8778.

†This work was supported by NIH grant R01 GM047291 and Welch Foundation Grant A1245 to PFF and NIH grant F31 GM077092 to JAP.

1Abbreviations: TyrH, tyrosine hydroxylase; PheH, phenylalanine hydroxylase; TrpH, tryptophan hydroxylase; TauD, taurine:α-ketoglutarate dioxygenase; 6MePH$_4$, 6-methyltetrahydropterin; 4a-HO-6MePH$_3$, 4a-hydroxypterin; 5-HO-trp, 5-hydroxytryptophan.

NIH-PA Author Manuscript
NIH-PA Author Manuscript
NIH-PA Author Manuscript

NIH-PA Author Manuscript NIH-PA Author Manuscript NIH-PA Author Manuscript

homotetramers, and alignment of sequences from different organisms reveals that each monomer is composed of a N-terminal regulatory domain and a C-terminal catalytic domain (10). The latter domains of about 300 amino acids are homologous (11–13), containing an active site Fe(II) facially coordinated by two histidines and a glutamate ($(His)_2$(Glu)-facial triad motif). A large number of non-heme iron dependent enzymes use a similar $(His)_2$(Asp/Glu)-facial triad motif to activate O_2 for substrate oxidation (14).

Scheme 2 shows the proposed chemical mechanism for the hydroxylation of tryptophan by TrpH (4). The initial steps in this mechanism require the irreversible activation of O_2 utilizing two electrons from a tetrahydropterin to form a high-valent Fe(IV)O intermediate (15) and 4a-hydroxypterin (4a-HO-$6MePH_3$), the first product (4). The Fe(IV)O intermediate subsequently reacts with the side chain of the aromatic amino acid through electrophilic aromatic substitution (16,17). Our present understanding of this mechanism has come primarily from spectroscopy of enzyme-substrate or enzyme-inhibitor complexes and steady-state kinetics. The changes in the ligands to the iron as substrates or analogs bind have been studied by magnetic circular dichroism and X-ray absorbance spectroscopy for both PheH (18,19) and TyrH (20). These results establish that binding of both the amino acid and tetrahydropterin results in a change in the coordination of the iron from 6-coordinate to 5-coordinate, presumably opening a coordination site for oxygen. In the case of PheH, the change in coordination was subsequently confirmed by X-ray spectroscopy, which showed that the glutamate becomes bidentate and two of the three water molecules are lost upon formation of the ternary complex (21). The ability of hydrogen peroxide to support hydroxylation of phenylalanine by the aromatic amino acid hydroxylases (22) is consistent with a high-valent iron species as the hydroxylating intermediate. The proposed electrophilic aromatic substitution mechanism for hydroxylation is supported by the observation of inverse secondary deuterium kinetic isotope effects for all the aromatic amino acid hydroxylases (17,23–25) and the kinetics of hydroxylation of 4-substituted phenylalanines by TyrH (16). The subsequent hydride shift was demonstrated by analyses of the products containing deuterium or tritium at the site of hydroxylation (17,26–28).

We have recently begun applying rapid-reaction methods to identify specific intermediates in the reactions of the aromatic amino acid hydroxylases and to determine the intrinsic rate constants for individual steps in catalysis. Stopped-flow kinetic analyses of TyrH confirm that binding of both ligands results in a substantial increase in the reactivity of the iron with O_2 (20). The proposal that the hydroxylating intermediate in this enzyme family is a Fe(IV)O has been confirmed by rapid freeze-quench ^{57}Fe Mössbauer spectroscopy and rapid chemical-quench experiments for TyrH (15). The quadrupole doublet and the isomer shift (δ) of the Fe(IV) intermediate in TyrH are similar to those for several members of the alpha-ketoglutarate-dependent family of non-heme enzymes (29–31). A combination of these approaches has been used to determine the rate constants for formation and decay of the Fe(IV)O in TyrH and to show that product release is rate-limiting for that enzyme (32). We describe here the results of similar studies of the reaction of TrpH, including the detection of a new intermediate that precedes formation of the Fe(IV)O.

Experimental Procedures

Materials

6-Methyltetrahydropterin ($6MePH_4$) was from B. Schircks Laboratories (Jona, Switzerland). Ampicillin was from USB Corporation (Cleveland Ohio). Dithiothreitol and isopropyl β-thiogalactopyranoside were from Inalco (Milano, Italy). L-Tryptophan, L-phenylalanine D,L-5-HO-trp, EDTA, nitrilotriacetic acid, glycerol, and monobasic and dibasic sodium phosphate were from Sigma-Aldrich Chemical Co. (Milwaukee, WI). Ferrous ammonium

NIH-PA Author Manuscript

NIH-PA Author Manuscript

NIH-PA Author Manuscript

sulfate, ammonium sulfate, ferric chloride, LB broth and HEPES were from Fisher (Pittsburgh, PA).

Expression and purification of TrpH

The catalytic core of rabbit TrpH, lacking 101 and 28 residues from the amino and carboxyl termini respectively, was purified according to previously published methods (17,23,33) with several modifications. C41DE3 *E. coli* cells were transformed with the plasmid pEWOHΔ101ΔH (33), and the cells were incubated overnight at 37 °C on a plate containing LB plus 100 μg/mL ampicillin. A single colony from the plate was selected and allowed to grow in 100 mL of LB broth plus 100 μg/mL ampicillin for about 12 h at 37 °C; 15 mL of the overnight culture was then used to inoculate 2 L of LB broth containing 100 μg/mL ampicillin, 1 mM ferric chloride and 4 mM magnesium sulfate. The cultures were grown until the A_{600} was between 0.3 and 0.5. The temperature was decreased to 20 °C and cell growth was continued until the A_{600} reached a value of 1.0–1.3. Isopropyl-β-thiogalactoside was then added to a final concentration of 500 μM. Cells were harvested by centrifugation after an additional 20–24 h at 20 °C. All subsequent purification steps for TrpH were performed at 4 °C as described previously (23,33,34). Typically 1.2–1.5 g of pure enzyme were obtained from 24 L.

Preparation of apo-TrpH

The purified enzyme obtained from 24 L of LB broth was precipitated with ammonium sulfate at 60% saturation. The enzyme pellet was resuspended in 50 to 80 mL of 150 mM HEPES/NaOH, 200 mM ammonium sulfate, 20% glycerol, 20 mM nitrilotriacetic acid, 20 mM EDTA, pH 7.0, and dialyzed against 1 L the same buffer with three buffer changes over 24 h. The enzyme was subsequently dialyzed against 800 mL of 150 mM HEPES/NaOH, 100 mM ammonium sulfate, 20% glycerol, pH 7.0 (buffer A), with four changes over a 28 h period. TrpH treated this way has no detectable activity in the absence of ferrous ammonium sulfate.

Stopped-flow absorption spectroscopy

Kinetic measurements were performed using an Applied Photophysics SX-18MV stopped-flow spectrophotometer. When data were collected at 350 nm and below, the path length was 2 mm, while it was 10 mm at longer wavelengths. Solutions of water and buffers were made anaerobic by alternating exposure to argon and vacuum.. The stopped-flow instrument was initially flushed with anaerobic water several times and then incubated for 1 to 2 h with 50 mM sodium dithionite. At this point apo-TrpH was placed in a tonometer with or without tryptophan. The TrpH or the TrpH· tryptophan sample was made anaerobic by alternating vacuum and argon cycles at 5 °C. The tonometer was very briefly opened to the atmosphere to allow addition of a stoichiometric amount of ferrous ammonium sulfate prepared in 5 mM HCl. Additional vacuum and argon gas cycles were performed at this stage. For experiments involving $6MePH_4$, the pterin was prepared by adding 1 ml buffer A to the appropriate amount of pterin under argon in a 2 mL Eppendorf tube to yield a final concentration of 50–80 mM; a volume corresponding to 1.5 to 2 equivalents of enzyme was withdrawn with a 1 mL air-tight syringe and placed in the side arm of the tonometer. The tonometer was exposed to additional vacuum and argon cycles for 10 min prior to mixing the $6MePH_4$ with the TrpH/Fe(II)· tryptophan solution. At this point the sodium dithionite was removed from the stopped-flow instrument by flushing it with anaerobic water followed immediately by anaerobic buffer A. The anaerobic enzyme sample was then mounted on the stopped-flow instrument. The reaction was initiated by mixing the respective complex with buffer A containing different concentrations of O_2. The different O_2 concentrations were obtained by mixing argon and O_2 with a modified MaxBlend medical oxygen blender (Maxtec) and bubbling the mixture of gases directly into the solution through an air-tight syringe.

NIH-PA Author Manuscript
NIH-PA Author Manuscript
NIH-PA Author Manuscript

Rapid-quench analyses

Rapid-mixing chemical-quench experiments were performed at 5 °C with a BioLogic QFM-400 quench-flow instrument. The instrument was calibrated using the reaction of 2,4-dinitrophenylacetate and hydroxide (35). The instrument and the TrpH/Fe(II)·tryptophan·6MePH$_4$ complex were prepared anaerobically as described for the stopped-flow experiments. The ~2 mM O_2 concentration for these experiment was obtained by placing buffer in a stirring round bottom flask sealed with a septum and bubbling with 100 % O_2 for at least 30 min while keeping the solution on ice. The reaction was initiated by mixing the TrpH/Fe(II)·tryptophan·6MePH$_4$ complex with O_2 at 5 °C using a 216 μL sample loop; the reaction time was varied by altering the flow rate. The reaction was quenched with an equal volume of 5 M HCl. Two to four samples were collected for each time point. The precipitated enzyme was removed from the samples by centrifugation at 15000 g for at least 10 min. The supernatant (100 μL) was diluted w15–30 fold with 2 M HCl and injected onto a Phenomenex Gemini C18 HPLC column (250 × 4.60 mm). The amino acid substrate and product were separated with an isocratic mobile phase of 15 mM sodium phosphate, pH 7.0, at a flow rate of 1.0 mL/min and detected using a Waters 2475 Multi λ Fluorescence Detector with excitation at 270 nm and emission at 310 nm.

Data analysis

Preliminary analysis of single stopped-flow traces and fitting to eq 1–4 were done using KaleidaGraph (Synergy Software). Stopped-flow kinetic traces obtained at multiple wavelengths were fit globally using the program Specfit32 (Spectrum Software Associates) to obtain spectra of the individual species. The program KinTek Explorer Pro (36) was used for global analyses of combined stopped-flow and rapid-quench results; confidence intervals were calculated using the FitSpace module and a χ^2 multiple of 1.2 (37).

Results

Stopped-flow studies of the reaction between TrpH/Fe(II) and O_2

The UV-visible spectrum of the non-heme iron center of TrpH is nearly identical to that previously described for TyrH (38). There is a weak charge-transfer absorbance band extending from 300 to about 400 nm, with the ferric form having a higher extinction coefficient than the ferrous enzyme over the entire range. The increased absorbance at 315 nm of about 800 M^{-1} cm^{-1} serves as an excellent spectroscopic probe for monitoring the oxidation of the ferrous enzyme. To determine the reactivity of ferrous TrpH alone with O_2, an anaerobic solution containing TrpH/Fe(II) was mixed with an equal volume of buffer containing different concentrations of O_2. Figure 1A shows the resulting absorbance traces; an increase in absorbance accompanying oxidation of the iron is clearly seen. A similar analysis was carried out in which tryptophan was added to the enzyme prior to the reaction with oxygen; the results are illustrated in Figure 1B. While the individual traces could be fit reasonably well as exponential increases in absorbance, these experiments were not carried out under pseudo-first order conditions, inasmuch as the concentration of oxygen varied from twice to one-fourth the enzyme concentration. Accordingly, the reaction was analyzed as an irreversible bimolecular reaction (Scheme 3) using the program KinTek Explorer Pro to fit the absorbance traces at all O_2 concentrations with a single second-order rate constant. The data in both the absence and the presence of tryptophan could be fit well using a rate constant of 104 ± 6 M^{-1} s^{-1} (Figure 1).

Stopped-flow studies of the reaction between TrpH/Fe(II)·6MePH$_4$·tryptophan and O_2

Stopped-flow absorption spectroscopy was next used to examine the kinetics of the complete TrpH reaction. Initially, a solution containing the TrpH/Fe(II)

NIH-PA Author Manuscript
NIH-PA Author Manuscript
NIH-PA Author Manuscript

$\cdot$6MePH$_4$$\cdot$tryptophan2 complex was mixed with oxygen-saturated buffer to yield final concentrations of ~400 μM enzyme and 625 μM O_2.

Figure 2A shows representative traces at 248, 318, and 335 nm. The wavelength 248 nm was selected to monitor the formation of the product 4a-HO-6MePH$_3$ (33, 39, 40); an increase at this wavelength is clearly seen. A high-valent Fe(IV)O intermediate has been detected at 315 to 365 nm in taurine:α-ketoglutarate dioxygenase (TauD), a mononuclear non-heme enzyme with a Fe(II) center similar to that of TrpH (30, 41, 42); consequently, the reaction was followed at 318 and 335 nm. Neither wavelength shows evidence of an intermediate in the first second of the reaction. Instead, the absorbance decreases at both wavelengths, consistent with the formation of 4a-HO-6MePH$_3$ (43).

In contrast to the relatively uninformative traces between 250 and 350 nm, monitoring the reaction at longer wavelengths showed the transient formation of an intermediate with a maximum near 400 nm. Accordingly, the kinetics of this intermediate were examined in more detail. Figure 3A shows the results when the reaction of the TrpH/Fe(II)$\cdot$6MePH$_4$$\cdot$tryptophan complex with oxygen is monitored at 400 nm. The formation of an intermediate is clearly seen in the first 100 ms. In addition, the intermediate forms at earlier times as the concentration of oxygen increases, suggesting that the rate constant for its formation depends on the oxygen concentration. The kinetics of formation and decay of the intermediate at each oxygen concentration could be fit individually as the sum of two exponential processes (results not shown). The rate constant for the first phase increased with increasing oxygen and the rate constant for the second phase at the two higher concentrations of oxygen was identical within error at 4.3 s^{-1}. The data at all three oxygen concentrations were then analyzed globally using KinTek Explorer. A fit of the data to a model involving an irreversible reaction of enzyme with oxygen to form the intermediate followed by decay of the intermediate (Scheme 4) gave a rate constant for the reaction with oxygen of 56 ± 2 $mM^{-1}s^{-1}$ and a rate constant for the second step of 4.1 ± 0.1 s^{-1}. However, the agreement between the data and the absorbance traces predicted by the model was poor. A model involving an additional step, reversible binding of oxygen, prior to formation of the intermediate (Scheme 5) gave a substantially better fit to the data (χ^2 = 3,960 versus 10,384 for the model of Scheme 4), and values for K_{O2}, k_2, and k_3 of 37 ± 3 μM, 54 ± 3 s^{-1}, and 4.4 ± 0.04 s^{-1}.[3] Similar evidence for initial reversible binding of oxygen has also been found with tyrosine hydroxylase (20).

To obtain the absorbance spectrum of the intermediate detected at 400 nm, the TrpH/Fe(II)$\cdot$6MePH$_4$$\cdot$tryptophan complex was mixed with an ~2-fold excess of oxygen, and reaction was monitored at 5 nm intervals from 360–470 nm. A spectrum of the intermediate (Figure 3B) was then generated by globally fitting all the data (Specfit32) from 2–300 ms to a mechanism with two sequential first order processes. The intermediate has its maximum absorbance at about 420 nm, with an extinction coefficient at this wavelength of 220 M^{-1} cm^{-1}.

Stopped-flow studies of the reaction between TrpH/Fe(II)·6MePH$_4$·phenylalanine and O_2

To determine if the identity of the amino acid substrate has an effect on the reaction between TrpH and O_2, similar stopped-flow experiments were performed with phenylalanine as the amino acid substrate. Previous studies have shown that phenylalanine is a good substrate for TrpH (33,44). The anaerobic TrpH/Fe(II)$\cdot$6MePH$_4$$\cdot$phenylalanine complex was mixed with buffer containing different O_2 concentrations, and the reaction was monitored at 248, 335

[2]The concentrations of substrates were set to be at least 10 times the K_m values while avoiding concentrations of free substrates that yield significant substrate inhibition (33).

[3]The confidence intervals calculated by FitSpace are: K_{O2}, 26 – 72 μM; k_2, 34 – 90 s^{-1}; k_3, 4.1 – 4.8 s^{-1}.

NIH-PA Author Manuscript

NIH-PA Author Manuscript

NIH-PA Author Manuscript

and 400 nm (Figures 2B and 3C). There were differences in the magnitudes of the absorbance changes from those seen with tryptophan, establishing that the identity of the amino acid substrate affects the kinetics of the intermediates. As is the case with tryptophan as substrate, an increase in absorbance can be seen at 248 nm with phenylalanine. Again, this can be assigned to formation of 4a-HO-6MePH$_3$. Unlike the case with tryptophan, the absorbance trace at 335 nm shows an increase after ~200ms. This increase occurs on a time scale consistent with product release (see below), suggesting that the spectrum of the pterin is altered in the enzyme active site compared to the spectrum in solution. The intermediate detectable at 400 nm is also seen with phenylalanine as substrate (Figure 3C). The formation of this intermediate is associated with an ~3-fold lower absorbance change at 400 nm with phenylalanine as substrate compared to that with tryptophan, requiring a higher concentration for analysis of the kinetics. Given the small absorbance change, no attempt was made to obtain the complete spectrum of the intermediate with phenylalanine. The kinetics of the absorbance change at 400 nm with phenylalanine also varied with the concentration of oxygen. In this case the results at different oxygen concentrations could be modeled using the mechanism of Scheme 4, which lacks a separate step for oxygen binding, to give rate constants of 280 ± 1 $mM^{-1}s^{-1}$ and 1.7 ± 0.02 s^{-1}.[4] The results were also analyzed using the mechanism of Scheme 5, which includes the additional binding step. However, to fit the data the value of the rate constant for formation of the intermediate (k_2 in Scheme 5) had to be set well above (>100x) that for its decay (k_3), so that its value had no effect on the kinetics. Consequently, this model was discarded for phenylalanine as substrate.

Rapid-mixing chemical quench studies

Rapid-quench methods were used to determine more directly the rate constant for formation of 5-HO-trp. Figure 4 shows the results of experiments in which the TrpH/Fe(II)· 6MePH$_4$· tryptophan (Figure 4A) or TrpH/Fe(II)·6MePH$_4$· phenylalanine (Figure 4B) complex was mixed with an equal volume of buffer containing 0.9–1.0 mM O_2 (concentration after mixing) and the reaction was quenched at the indicated times with 5 M HCl. With both substrates there is a clear burst in the formation of the hydroxylated amino acid. Accordingly, the data for were fit to eq 1, where $(HO\text{-}aa/TrpH)_t$ is the ratio of hydroxylated amino acid to the enzyme at time t, $(HO\text{-}aa/TrpH)_0$ is the burst amplitude, k_{burst} is the rate constant for the burst phase, and k_{linear} is the steady state rate. With tryptophan as substrate, this gave values for the burst amplitude of 0.46 ± 0.19, for k_{burst} of 1.3 ± 0.6 s^{-1}, and for k_{linear} of 0.25 ± 0.05 s^{-1}. With phenylalanine as substrate the values of the burst amplitude, k_{burst}, and k_{linear} were 0.53 ± 0.11, 2.6 ± 1.2 s^{-1} and 0.26 ± 0.02 s^{-1}, respectively. With both substrates the value for k_{linear} is equal to the steady-state k_{cat} value.

$$(HO-aa/TrpH)_t=(HO-aa/TrpH)_0 \bullet (1-e^{-k_{burst}t})+k_{linear}t \quad (1)$$

While a burst amplitude of zero would establish that hydroxylation is fully rate-limiting and an amplitude of one would establish that a subsequent step is fully rate-limiting, intermediate values such those observed with TrpH are ambiguous. It may be due to neither hydroxylation nor product release being fully rate-limiting or it may be due to only a fraction of the enzyme population being active. To distinguish between these possibilities, a series of chemical quench experiments were performed with tryptophan as substrate in which the final concentration of TrpH varied from 100 to 300 μM. The resulting data were analyzed using the kinetic model in Scheme 6. Here, $k_{3'}$ is the net rate constant for

[4] The confidence intervals calculated by FitSpace are: k_1, 260 – 310 $mM^{-1}s^{-1}$; k_2, 1.4 – 2.0 s^{-1}.

conversion of the free enzyme to the enzyme-product complex, and k_4 is the rate constant for formation of the free enzyme from the enzyme-product complex. For such a model, the burst amplitude, k_{burst}, and k_{linear} are related to $k_{3'}$, k_4, the concentration of catalytically active enzyme ($[TrpH]_a$), and the total enzyme concentration ($[TrpH]_{tot}$) by eqs 2–4 (45). The average values from the analyses of six different experiments were $[TrpH]_a/[TrpH]_{tot}$ = 0.56 ± 0.22, $k_{3'}$ = 1.6 ± 0.6, and k_4 = 0.30 ± 0.12. A similar analysis of the data for phenylalanine shown in Figure 4B yielded values of the active fraction, $k_{3'}$, and k_4 as 0.67 ± 0.21, 2.3 ± 1.2 s^{-1} and 0.29 ± 0.02 s^{-1}. These data show that most preparations of TrpH contain a significant fraction of inactive enzyme; for the experiment shown in Figure 4A, the fraction of active enzyme was 0.92, while it was 0.75 for the experiment in Figure 4B.

$$(HO-aa/TrpH)_0=[TrpH]_a/[TrpH]_{tot} \bullet \{k_{3'}/(k_4+k_{3'})\}^2 \quad (2)$$

$$k_{burst}=k_{3'}+k_4 \quad (3)$$

$$k_{linear}=([TrpH]_a/[TrpH]_{tot}) \bullet [k_{3'} \bullet k_4/(k_{3'}+k_4)] \quad (4)$$

Global analysis of the kinetics

All the stopped-flow and rapid-quench data shown in Figures 2, 3, and 4 were analyzed globally to obtain a single set of kinetic parameters for each substrate. In the case of tryptophan as substrate, the initial fitting described above of the absorbance traces at 400 nm required three kinetically important steps with values for K_{O2}, k_2, and k_3 of 37 μM, 54 s^{-1}, and 4.4 s^{-1}. The rapid-quench data required two slower steps with rate constants of 1.6 s^{-1}, and k_4 = 0.3 s^{-1}. Consequently, The data were modeled with a mechanism involving reversible oxygen binding followed by four first order steps (Scheme 7). To do so, the K_{O2} value was fixed at 37 μM, but all the individual rate constants were allowed to vary). The resulting values are shown in Table 1. The results calculated from these values are shown as the lines in the corresponding figures. All of the data are accommodated reasonably well by the model. In addition, the results from the global analysis that includes stopped-flow data at multiple wavelengths are consistent with the more limited analyses of the stopped-flow data at 400 nm and the rapid-quench data.

In the case of phenylalanine as substrate, the initial analysis of the stopped-flow data at 400 nm did not justify a separate oxygen binding step. In addition, the rate constant for the second step in that analysis, 1.7 s^{-1}, is comparable to the rate constant for the burst phase in the rapid-quench analysis, 2.3 s^{-1}. Consequently, the much simpler mechanism of Scheme 8, which only includes three steps, was used to model all of the data with phenylalanine. The resulting rate constants are given in Table 2, while the lines in Figures 2B, 3C, and 4B illustrate the agreement of the model with the data.

Discussion

The present results establish that the ternary complex of TrpH with tryptophan and tetrahydropterin reacts much more rapidly and productively with oxygen than the enzyme alone. While the active site iron in TrpH does react with oxygen in the absence of substrates, the result is simply oxidation of the iron, and the reaction is well-described as a simple second-order reaction with no evidence for intermediates. Binding of the amino acid

NIH-PA Author Manuscript NIH-PA Author Manuscript NIH-PA Author Manuscript

NIH-PA Author Manuscript NIH-PA Author Manuscript NIH-PA Author Manuscript

substrate alone has no effect on the oxygen reactivity of TrpH in the absence of the tetrahydropterin. In contrast, the reaction is much more rapid when both amino acid and tetrahydropterin are bound, and the product of the reaction is different. With either phenylalanine or tryptophan as the amino acid substrate, the effective second-order rate constant for formation of the first detectable intermediate is more than 1,000-fold greater than the rate constant for the oxidation of the Fe(II).

The effects of substrates on the reaction of TyrH with oxygen have also been examined (20,38); as with TrpH, the reaction is similar in the presence and absence of the amino acid substrate. The value of the rate constant for TyrH in the absence of any substrate, 37 $M^{-1}s^{-1}$ at 5 °C, is comparable to the value reported here for TrpH at the same temperature 104 $M^{-1}s^{-1}$, consistent with the similar reactivities of the iron sites in the two proteins (9). (4-Hydroxyphenyl)-pyruvate dioxygenase is a member of the α-ketoglutarate-dependent dioxygenase family and contains a $(His)_2$(Asp/Glu)-facial triad similar to that in the aromatic amino acid hydroxylases (46). The oxidation by O_2 of the Fe(II) in that enzyme is also an irreversible bimolecular reaction, with a second order rate constant of 46 M^{-1} s^{-1} (47,48), comparable to those for TyrH and TrpH, and binding of the substrate (4-hydroxyphenyl)-pyruvate increases the reactivity with oxygen 3600-fold. The low magnitude of these rate constants for the substrate-free enzymes establishes that the Fe(II) centers in these nonheme iron enzymes not suitable for a reaction with O_2 in the absence of one or both substrates. Productive oxygen activation requires changes in the coordination of the iron that occur upon binding of both the reducing substrate and the substrate to be hydroxylated (5,49).

The reaction of the ternary TrpH-amino acid-pterin complex with oxygen is clearly more complex than the autooxidation reaction. The combined data presented here for the complete hydroxylation reaction of TrpH with tryptophan as substrate describe a reaction with a minimum of four discrete steps after oxygen binding: formation and decay of a species with maximum absorbance in the visible range at 420 nm (TrpH˙I˙trp), hydroxylation of tryptophan to form HO-tryptophan, and a slow final step that is likely to be product release. The data are consistent with the identification of these steps shown in Scheme 7. The details of the reversible binding of oxygen are not established by the present analysis. Simulations of oxygen binding to several redox-active proteins have implicated the protein matrix as the initial site of interaction with oxygen (50). In the present case, binding of oxygen to the TrpH˙6-$MePH_4$˙trp complex produces TrpH˙I˙trp. Decay of TrpH˙I˙trp yields TrpH˙trp containing bound Fe(IV)O and hydroxypterin. The reaction between the Fe(IV)O and tryptophan yields 5-HO-trp and Fe(II). Finally, release of 5-HO-trp and hydroxypterin regenerates the free enzyme with Fe(II). Given the proposed chemical mechanism in Scheme 2 for TrpH, possible candidates for I are the iron-peroxy-pterin species, an intermediate preceding it, and the Fe(IV)O plus hydroxypterin.5 It is unlikely that I, which forms at rates approaching 65 s^{-1}, contains the Fe(IV)O. Formation of Fe(IV)O necessarily involves formation of the hydroxypterin product, while its decay involves formation of 5-HO-trp. In the stopped-flow experiment depicted in Figure 2, the maximum changes in the absorbance at 248 nm, where the hydroxypterin has an absorbance maximum, occur during the steps with rate constants of 4.4 and 0.2 s^{-1}. The value 4.4 s^{-1} matches that for the decay of I, consistent with I not containing the Fe(IV)O, but instead preceding it in the reaction.

[5]Rapid freeze-quench Mössbauer and EPR spectroscopy experiments were performed to characterize the oxidation state of the iron in the intermediate detected at 400 nm and to detect the Fe(IV)O intermediate in TrpH. When the TrpH/Fe(II)·6$MePH_4$·tryptophan complex [2 mM] was mixed with 2 mM O_2 no significant change in the Mössbauer spectrum from that of the enzyme was observed. Subsequently, in a rapid-chemical quench experiments under the same conditions the initial rate of 5-hydroxytryptophan formation was 10% of the expected value, suggesting that TrpH is inactive at a concentration of 1 mM. Previous ultracentrifugation analyses of the TrpH form used in these analyses showed formation of higher order species with increasing concentrations, consistent with aggregation of the protein at high (>0.5 mM) concentrations (33).

NIH-PA Author Manuscript

NIH-PA Author Manuscript

NIH-PA Author Manuscript

Just as the rate constant for formation of the Fe(IV)O can be obtained from the absorbance changes at 248 nm due to formation of the hydroxypterin, the rate constant for its decay can be obtained from the rapid-quench analyses of 5-HO-trp formation. Analysis of the burst kinetics yields a rate constant for formation of 5-HO-trp that is within error of the rate constant of 1.3 s^{-1} for the fourth step in the overall reaction. While this rules out I as containing the Fe(IV)O, it does not establish its identity. To our knowledge there is no precedent for a mononuclear iron peroxo, although a peroxodiron species has been detected in a mutant protein of toluene dioxygenase (51). At present the identity of I must remain speculative.

The observation of burst kinetics for the formation of hydroxylated amino acid establishes that a slow step follows hydroxylation. The most likely candidate for this is product release. The data do not address directly whether it is release of the pterin or the hydroxylated amino acid that is slower. The increase in the absorbance of the pterin at 248 nm during the last step is most readily explained as a change in the pterin spectrum occurring upon its release from the enzyme active site, possibly due to a change in the protonation state of the hydroxypterin. The reaction of TyrH is also limited by slow release of a product from the active site (32). Binding of pterin to TyrH is associated with closure of an active site loop containing Phe184 (52) in a step that is sensitive to the viscosity of the solvent (32). Release of the hydroxypterin would presumably require movement of this loop away from the active site. Mutations of residues 181–189 affect the k_{cat} value of TyrH (53), consistent with such a model. However, the position of this loop in the X-ray structures of all three hydroxylases is unchanged when a pterin is bound (12,21,54), so that the role of loop motions in ligand binding by these enzymes remains unsettled.

The single turnover kinetics with phenylalanine as substrate appear to be simpler than those with tryptophan. The experimental results in this case can be accommodated using only three steps, formation and decay of I and release of tyrosine from the enzyme. The lack of kinetic evidence for an initial oxygen-enzyme species may reflect weaker binding when the non-physiological substrate phenylalanine is bound or it may simply reflect the limitations imposed by the weak absorbance of this chromophore and resulting limited range of oxygen concentrations that could be used experimentally. In this case the decay of I cannot be resolved from formation of tyrosine, suggesting that the reaction of the Fe(IV)O with phenylalanine to form tyrosine is significantly faster than formation of the Fe(IV)O from I. As is the case with tryptophan as substrate, the slowest step in the overall reaction can be assigned to product release based on the burst of tyrosine seen in the rapid-quench experiment.

Previous steady-state kinetic analyses of TrpH showed that the k_{cat} value with tryptophan as substrate is ~50% of the value with phenylalanine as substrate (33). The present results at a lower temperature show that this difference can be attributed to faster release of the product tyrosine from the enzyme than of HO-tryptophan. The previous observation that the k_{cat} values for the poor substrates 4-methyl- and 4-azatryptophan are greater than that for tryptophan can similarly be attributed to faster product release with those substrates (55).

An inverse deuterium kinetic isotope effect on the k_{cat} value for TrpH was previously reported with 5-^{2}H-tryptophan as evidence for rehybridization of C5 upon formation of the new carbon oxygen bond in an electrophilic aromatic substitution reaction (17). The present results suggest that the reported value is not the intrinsic isotope effect, since k_4 (hydroxylation) is 5–7 fold faster than k_5 (product release), and thus does not accurately reflect the structure of the transition state for hydroxylation. The slower product release will result in a $^{D}k_{cat}$ value less inverse than the intrinsic isotope effect for formation of the new C-O bond.6 If the $^{D}k_{cat}$ value is taken to be 0.96, within the error limits of the reported

NIH-PA Author Manuscript

NIH-PA Author Manuscript

NIH-PA Author Manuscript

isotope effect (17), the intrinsic isotope effect for C-O bond formation can be calculated to be between 0.66 and 0.77 using the confidence limits in Table 1. Use of the rate constants derived only from the chemical quench analyses yields an intrinsic isotope effect of 0.75 with lower precision.

In conclusion the results presented here establish that TrpH only reacts productively with O_2 in the presence of both the tetrahydropterin and the amino acid substrate. The ternary complex reacts rapidly with O_2 to form an intermediate detectable at 420 nm. This intermediate decays to form the Fe(IV)O hydroxylating intermediate. Overall turnover is limited by release of a product, either the hydroxypterin or the hydroxylated amino acid.

Acknowledgments

We thank Drs. J. Martin Bollinger and Carsten Krebs of Pennsylvania State University for their assistance with the rapid-quench Mössbauer analyses.

References

1. Jequier E, Lovenberg W, Sjoerdsma A. Tryptophan hydroxylase inhibition: The mechanism by which p-chlorophenylalanine depletes brain serotonin. Mol Pharmacol. 1967; 3:274–278. [PubMed: 6037686]
2. Lovenberg W, Jequier E, Sjoerdsma A. Tryptophan hydroxylation: Measurement in pineal gland, brainstem, and carcinoid tumor. Science. 1967; 155:217–219. [PubMed: 6015530]
3. Fitzpatrick, PF. The aromatic amino acid hydroxylases. In: Purich, DL., editor. Adv Enzymol Relat Areas Mol Biol. John Wiley & Sons, Inc; 2000. p. 235-294.
4. Fitzpatrick PF. Mechanism of aromatic amino acid hydroxylation. Biochemistry. 2003; 42:14083–14091. [PubMed: 14640675]
5. Costas M, Mehn MP, Jensen PM, Que L Jr. Dioxygen activation at mononuclear nonheme iron active sites: enzymes, models, and intermediates. Chem Rev. 2004; 104:939–986. [PubMed: 14871146]
6. Solomon EI, Brunold TC, Davis MI, Kemsley JN, Lee SK, Lehnert N, Neese F, Skulan AJ, Yang YS, Zhou J. Geometric and electronic structure/function correlations in non-heme iron enzymes. Chem Rev. 2000; 100:235–349. [PubMed: 11749238]
7. Carr RT, Balasubramanian S, Hawkins PCD, Benkovic SJ. Mechanism of metal-independent hydroxylation by *Chromobacterium violaceum* phenylalanine hydroxylase. Biochemistry. 1995; 34:7525–7532. [PubMed: 7779797]
8. Frantom PA, Pongdee R, Sulikowski GA, Fitzpatrick PF. Intrinsic deuterium isotope effects on benzylic hydroxylation by tyrosine hydroxylase. J Am Chem Soc. 2002; 124:4202–4203. [PubMed: 11960436]
9. Pavon JA, Fitzpatrick PF. Intrinsic isotope effects on benzylic hydroxylation by the aromatic amino acid hydroxylases: evidence for hydrogen tunneling, coupled motion, and similar reactivities. J Am Chem Soc. 2005; 127:16414–16415. [PubMed: 16305226]
10. Ledley FD, DiLella AG, Kwok SCM, Woo SLC. Homology between phenylalanine and tyrosine hydroxylases reveals common structural and functional domains. Biochemistry. 1985; 24:3389–3394. [PubMed: 2412578]
11. Kobe G, Jennings IG, House CM, Feil SC, Michell BJ, Tiganis T, Parker MW, Cotton RGH, Kemp BE. Regulation and crystallization of phosphorylated and dephosphorylated forms of truncated dimeric phenylalanine hydroxylase. Protein Sci. 1997; 6:1352–1357. [PubMed: 9194198]

[6]The rate constants in Table 1 show that the k_{cat} value is limited by k_4 and k_5. Equation 5 gives the relationship of k_4, k_5 and the isotope effect on k_{cat} ($^Dk_{cat}$) to the intrinsic isotope effect ($^Dk_4$).

$$^Dk_{cat}=(^Dk_4+k_4/k_5)/(1+k_4/k_5) \quad (5)$$

NIH-PA Author Manuscript
NIH-PA Author Manuscript
NIH-PA Author Manuscript

12. Wang L, Erlandsen H, Haavik J, Knappskog PM, Stevens RC. Three-dimensional structure of human tryptophan hydroxylase and its implications for the biosynthesis of the neurotransmitters serotonin and melatonin. Biochemistry. 2002; 41:12569–12574. [PubMed: 12379098]
13. Goodwill KE, Sabatier C, Marks C, Raag R, Fitzpatrick PF, Stevens RC. Crystal structure of tyrosine hydroxylase at 2.3 Ångstrom and its implications for inherited neurodegenerative diseases. Nat Struct Biol. 1997; 4:578–585. [PubMed: 9228951]
14. Koehntop KD, Emerson JP, Que L Jr. The 2-His-1-carboxylate facial triad: a versatile platform for dioxygen activation by mononuclear non-heme iron(II) enzymes. J Biol Inorg Chem. 2005; 10:87–93. [PubMed: 15739104]
15. Eser BE, Barr EW, Frantom PA, Saleh L, Bollinger JM Jr, Krebs C, Fitzpatrick PF. Direct spectroscopic evidence for a high-spin Fe(IV) intemediate in tyrosine hydroxylase. J Am Chem Soc. 2007; 129:11334–11335. [PubMed: 17715926]
16. Hillas PJ, Fitzpatrick PF. A mechanism for hydroxylation by tyrosine hydroxylase based on partitioning of substituted phenylalanines. Biochemistry. 1996; 35:6969–6975. [PubMed: 8679520]
17. Moran GR, Derecskei-Kovacs A, Hillas PJ, Fitzpatrick PF. On the catalytic mechanism of tryptophan hydroxylase. J Am Chem Soc. 2000; 122:4535–4541.
18. Loeb KE, Westre TE, Kappock TJ, Mitic N, Glasfeld E, Caradonna JP, Hedman B, Hodgson KO, Solomon EI. Spectroscopic characterization of the catalytically competent ferrous site of the resting, activated, and substrate-bound forms of phenylalanine hydroxylase. J Am Chem Soc. 1997; 119:1901–1915.
19. Kemsley JN, Mitic N, Zaleski KL, Caradonna JP, Solomon EI. Circular dichroism and magnetic circular dichroism spectroscopy of the catalytically competent ferrous active site of phenylalanine hydroxylase and its interaction with pterin cofactor. J Am Chem Soc. 1999; 121:1528–1536.
20. Chow MS, Eser BE, Wilson SA, Hodgson KO, Hedman B, Fitzpatrick PF, Solomon EI. Spectroscopy and kinetics of wild-type and mutant tyrosine hydroxylase: Mechanistic insight into O_2 activation. J Amer Chem Soc. 2009; 131:7685–7698. [PubMed: 19489646]
21. Andersen OA, Flatmark T, Hough E. Crystal structure of the ternary complex of the catalytic domain of human phenylalanine hydroxylase with tetrahydrobiopterin and 3-(2-thienyl)-L-alanine, and its implications for the mechanism of catalysis and substrate activation. J Mol Biol. 2002; 320:1095–1108. [PubMed: 12126628]
22. Pavon JA, Fitzpatrick PF. Demonstration of a peroxide shunt in the tetrahydropterin-dependent aromatic amino acid monooxygenases. J Am Chem Soc. 2009; 131:4582–4583. [PubMed: 19281164]
23. Pavon JA, Fitzpatrick PF. Insights into the catalytic mechanisms of phenylalanine and tryptophan hydroxylase from kinetic isotope effects on aromatic hydroxylation. Biochemistry. 2006; 45:11030–11037. [PubMed: 16953590]
24. Panay AJ, Fitzpatrick PF. Kinetic Isotope Effects on Aromatic and Benzylic Hydroxylation by *Chromobacterium violaceum* Phenylalanine Hydroxylase as Probes of Chemical Mechanism and Reactivity. Biochemistry. 2008; 47:11118–11124. [PubMed: 18817418]
25. Frantom PA, Fitzpatrick PF. Uncoupled forms of tyrosine hydroxylase unmask kinetic isotope effects on chemical steps. J Am Chem Soc. 2003; 125:16190–16191. [PubMed: 14692751]
26. Guroff G, Levitt M, Daly J, Udenfriend S. The production of meta-tritiotyrosine from p-tritio-phenylalanine by phenylalanine hydroxylase. Biochem Biophys Res Commun. 1966; 25:253–259. [PubMed: 5971770]
27. Renson JD, Daly J, Weissbach H, Witkop B, Udenfriend S. Enzymatic conversion of 5-tritiotryptophan to 4-tritio-5-hydroxytryptophan. Biochem Biophys Res Commun. 1966; 25:504–513.
28. Daly J, Levitt M, Guroff G, Udenfriend S. Isotope studies on the mechanism of action of adrenal tyrosine hydroxylase. Arch Biochem Biophys. 1968; 126:593–598. [PubMed: 5672518]
29. Galonic DP, Barr EW, Walsh CT, Bollinger JM Jr, Krebs C. Two interconverting Fe(IV) intermediates in aliphatic chlorination by the halogenase CytC3. Nature Chem Biol. 2007; 3:113–116. [PubMed: 17220900]

NIH-PA Author Manuscript
NIH-PA Author Manuscript
NIH-PA Author Manuscript

30. Price JC, Barr EW, Tirupati B, Bollinger JM, Krebs C. The first direct characterization of a high-valent iron intermediate in the reaction of an alpha-ketoglutarate-dependent dioxygenase: a high-spin Fe(IV) complex in taurine/alpha-ketoglutarate dioxygenase (TauD) from *Escherichia coli*. Biochemistry. 2003; 42:7497–7508. [PubMed: 12809506]
31. Hoffart LM, Barr EW, Guyer RB, Bollinger JM Jr, Krebs C. Direct spectroscopic detection of a C-H-cleaving high-spin Fe(IV) complex in a prolyl-4-hydroxylase. Proc Natl Acad Sci USA. 2006; 103:14738–14743. [PubMed: 17003127]
32. Eser BE, Fitzpatrick PF. Measurement of Intrinsic Rate Constants in the Tyrosine Hydroxylase Reaction. Biochemistry. 2010; 49:645–652. [PubMed: 20025246]
33. Moran GR, Daubner SC, Fitzpatrick PF. Expression and characterization of the catalytic core of tryptophan hydroxylase. J Biol Chem. 1998; 273:12259–12266. [PubMed: 9575176]
34. Daubner SC, Hillas PJ, Fitzpatrick PF. Expression and characterization of the catalytic domain of human phenylalanine hydroxylase. Arch Biochem Biophys. 1997; 348:295–302. [PubMed: 9434741]
35. Gutfreund H. Resolution of optical and sampling methods. Method Enzymol. 1969; 16:229–249.
36. Johnson KA, Simpson ZB, Blom T. Global Kinetic Explorer: A new computer program for dynamic simulation and fitting of kinetic data. Anal Biochem. 2009; 387:20–29. [PubMed: 19154726]
37. Johnson KA, Simpson ZB, Blom T. FitSpace Explorer: An algorithm to evaluate multidimensional parameter space in fitting kinetic data. Anal Biochem. 2009; 387:30–41. [PubMed: 19168024]
38. Frantom PA, Seravalli J, Ragsdale SW, Fitzpatrick PF. Reduction and oxidation of the active site iron in tyrosine hydroxylase: Kinetics and specificity. Biochemistry. 2006; 45:2372–2379. [PubMed: 16475826]
39. Ellis HR, Daubner SC, Fitzpatrick PF. Mutation of serine 395 of tyrosine hydroxylase decouples oxygen-oxygen bond cleavage and tyrosine hydroxylation. Biochemistry. 2000; 39:4174–4181. [PubMed: 10747809]
40. Davis MD, Kaufman S. Evidence for the formation of the 4a-carbinolamine during the tyrosine-dependent oxidation of tetrahydrobiopterin by rat liver phenylalanine hydroxylase. J Biol Chem. 1989; 264:8585–8596. [PubMed: 2722790]
41. Krebs C, Price JC, Baldwin J, Saleh L, Green MT, Bollinger JM Jr. Rapid freeze-quench Mossbauer spectroscopy: monitoring changes of an iron-containing site during a biochemical reaction. Inorg Chem. 2005; 44:742–757. [PubMed: 15859243]
42. Grzyska PK, Ryle MJ, Monterosso GR, Liu J, Ballou DP, Hausinger RP. Steady-state and transient kinetic analyses of taurine/α-ketoglutarate dioxygenase: Effects of oxygen concentration, alternative sulfonates, and active-site variants on the FeIV-oxo intermediate. Biochemistry. 2005; 44:3845–3855. [PubMed: 15751960]
43. Dix TA, Benkovic SJ. Mechanism of ‘uncoupled’ tetrahydropterin oxidation by phenylalanine hydroxylase. Biochemistry. 1985; 24:5839–5846. [PubMed: 4084494]
44. Daubner SC, Moran GR, Fitzpatrick PF. Role of tryptophan hydroxylase Phe313 in determining substrate specificity. Biochem Biophys Res Commun. 2002; 292:639–641. [PubMed: 11922614]
45. Fersht, A. Enzyme Structure and Mechanism. 2. W. H. Freeman and Company; New York: 1985.
46. Moran GR. 4-Hydroxyphenylpyruvate dioxygenase. Arch Biochem Biophys. 2005; 433:117–128. [PubMed: 15581571]
47. Pumpero VM, Moran GR. Catalytic, noncatalytic, and inhibitory phenomena: kinetic analysis of (4-hydroxyphenyl)pyruvate dioxygenase from *Arabidopsis thaliana*. Biochemistry. 2006; 45:6044–6055. [PubMed: 16681377]
48. Johnson-Winters K, Pumpero VM, Kavana M, Nelson T, Moran GR. (4-hydroxyphenyl) pyruvate dioxygenase from *Streptomyces avermitilis*: The basis for ordered substrate addition. Biochemistry. 2003; 42:2072–2080. [PubMed: 12590595]
49. Bassan A, Borowski T, Siegbahn Per EM. Quantum chemical studies of dioxygen activation by mononuclear non-heme iron enzymes with the 2-His-1-carboxylate facial triad. Dalton Trans. 2004; 20:3153–3162. [PubMed: 15483690]

NIH-PA Author Manuscript
NIH-PA Author Manuscript
NIH-PA Author Manuscript

50. Baron R, McCammon JA, Mattevi A. The oxygen-binding vs. oxygen-consuming paradigm in biocatalysis: structural biology and biomolecular simulation. Curr Opin Struct Biol. 2009; 19:672–679. [PubMed: 19896366]
51. Song WJ, Behan RK, Naik SG, Huynh BH, Lippard SJ. Characterization of a Peroxodiiron(III) Intermediate in the T201S Variant of Toluene/o-Xylene Monooxygenase Hydroxylase from *Pseudomonas sp.* OX1. J Am Chem Soc. 2009; 131:6074–6075. [PubMed: 19354250]
52. Sura GR, Lasagna M, Gawandi V, Reinhart GD, Fitzpatrick PF. Effects of ligands on the mobility of an active-site loop in tyrosine hydroxylase as monitored by fluorescence anisotropy. Biochemistry. 2006; 45:9632–9638. [PubMed: 16878998]
53. Daubner SC, McGinnis JT, Gardner M, Kroboth SL, Morris AR, Fitzpatrick PF. A flexible loop in tyrosine hydroxylase controls coupling of amino acid hydroxylation to tetrahydropterin oxidation. J Mol Biol. 2006; 359:299–307. [PubMed: 16618490]
54. Goodwill KE, Sabatier C, Stevens RC. Crystal structure of tyrosine hydroxylase with bound cofactor analogue and iron at 2.3 Å resolution: self-hydroxylation of phe300 and the pterin-binding site. Biochemistry. 1998; 37:13437–13445. [PubMed: 9753429]
55. Moran GR, Phillips RS, Fitzpatrick PF. The influence of steric bulk and electrostatics on the hydroxylation regiospecificity of tryptophan hydroxylase: Characterization of methyltryptophans and azatryptophans as substrates. Biochemistry. 1999; 38:16283–16289. [PubMed: 10587452]

NIH-PA Author Manuscript

NIH-PA Author Manuscript

NIH-PA Author Manuscript

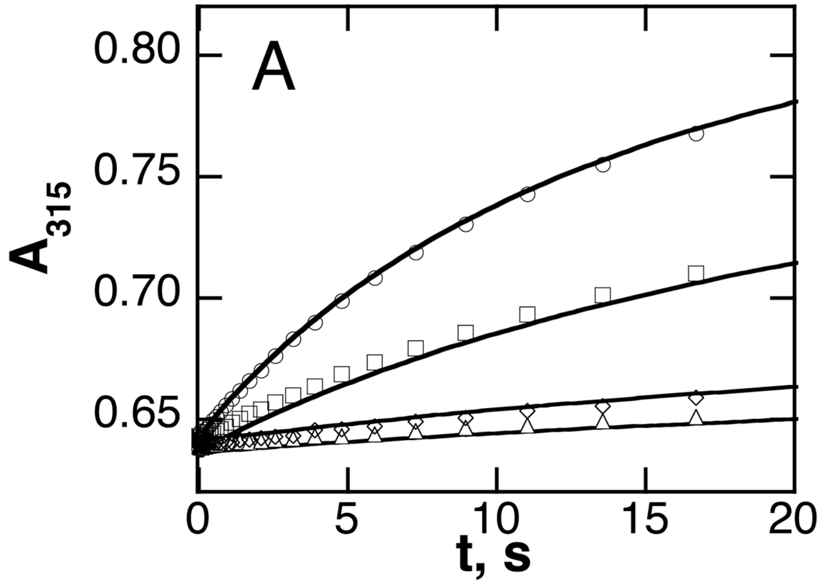

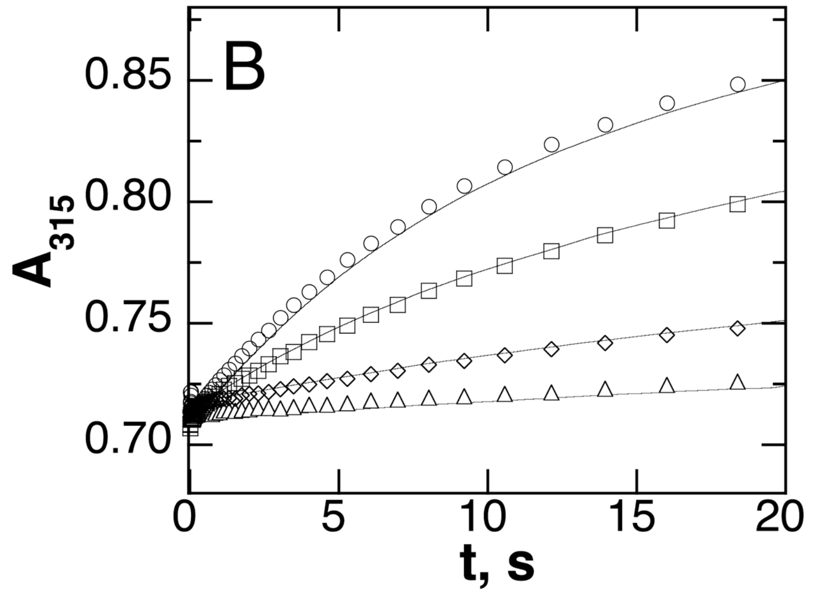

Figure 1.

Stopped-flow absorbance traces for the reaction of 300 μM TrpH/Fe(II) with oxygen in the absence (A) or presence of 400 μM tryptophan (B). The final concentrations of oxygen were 600 μM (circles), 300 μM (squares), 125 μM (diamonds) and 80 μM (triangles). Only 5% of the points are shown for clarity. The lines are from global simulations (KinTek Explorer Pro) of the reactions at all four oxygen concentrations using a second order irreversible reaction with a rate constant of 104 $M^{-1}s^{-1}$. Conditions: 150 mM HEPES/NaOH, 100 mM ammonium sulfate, 20% glycerol, pH 7.0, 5 °C

NIH-PA Author Manuscript

NIH-PA Author Manuscript

NIH-PA Author Manuscript

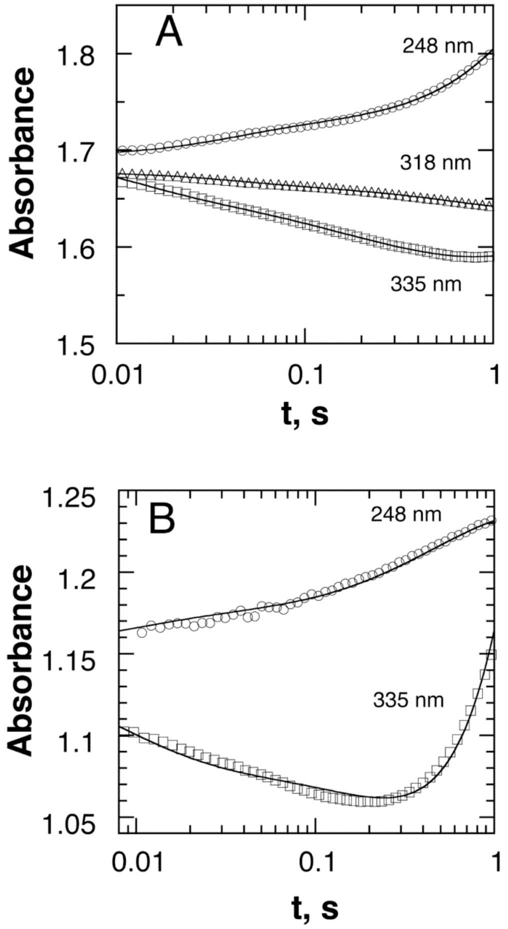

Figure 2.

Absorbance changes during TrpH turnover. A: Stopped-flow traces at 335 nm (squares), 318 nm (triangles), and 248 nm (circles) during the reaction of 380 μM TrpH/Fe(II)·700 μM tryptophan·1000 μM 6MePH$_4$ with 400 μM oxygen. The lines are from a global fit of the data in Figures 2A, 3A, and 4A to the mechanism in Scheme 7 using the rate constants in Table 1. B: Stopped-flow traces at 335 nm (squares) and 248 nm (circles) during the reaction of 350 μM TrpH/Fe(II)·550 μM phenylalanine·550 μM 6MePH$_4$ with 625 μM oxygen. The lines are from a global fit of the data in Figures 2B, 3C, and 4B to the mechanism in Scheme 8 using the rate constants in Table 2. In both A and B, only a portion of the points are shown for clarity, and the traces have been offset to allow them to be shown more clearly. Conditions as described for Figure 1.

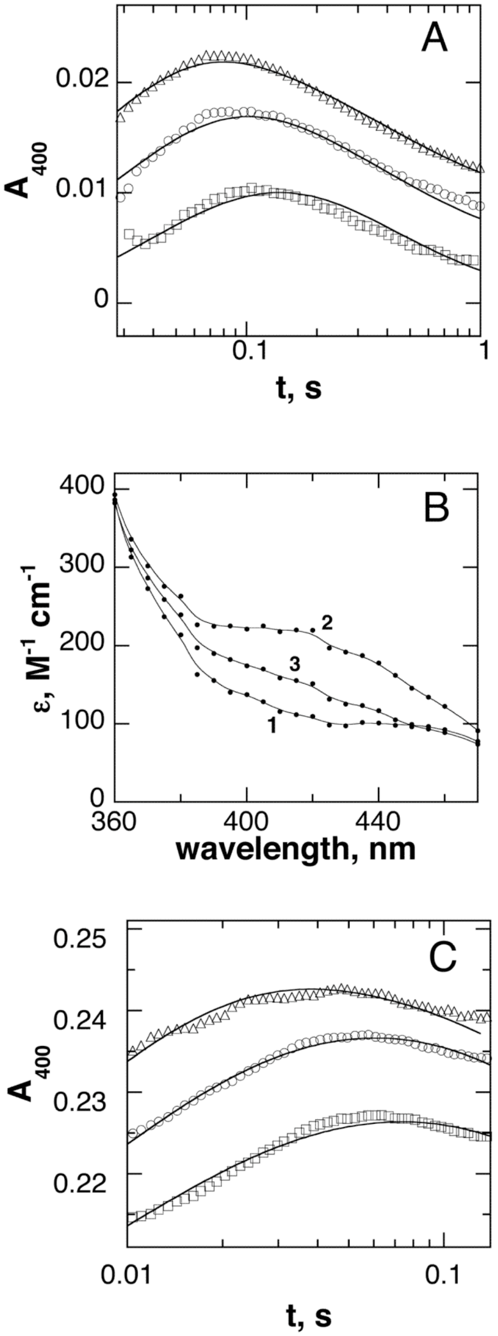

Figure 3.

Detection of an early intermediate in the reaction of the TrpH reaction. A: Stopped-flow traces at 400 nm during the reaction of 125 μM TrpH/Fe(II)·500 μM tryptophan·1000 μM $6MePH_4$ with 230 μM (squares), 400 μM (circles), or 650 μM O_2 (triangles). The lines are from a global fit of the data in Figures 2A, 3A, and 4A to the mechanism in Scheme 7 using the rate constants in Table 1. B: Calculated spectra of the initial TrpH/Fe(II) ·tryptophan·$6MePH_4$ complex (1), the intermediate formed in the initial reaction with O_2 (2), and the species formed upon decay of this intermediate (3). TrpH·Fe(II)(350 μM)·500 μM tryptophan·1000 μM $6MePH_4$ and 625 μM O_2 (final concentrations) were mixed in the stopped-flow instrument, and the reaction was monitored at 5 nm intervals from 360 to 470

NIH-PA Author Manuscript NIH-PA Author Manuscript NIH-PA Author Manuscript

nm. The resulting data were fit globally (Specfit32) to a two step mechanism to obtain the spectra. C: Absorbance changes during the reaction of 350 μM TrpH/Fe(II)·550 μM phenylalanine·550 μM 6MePH$_4$ with 625 μM (triangles), 450 μM (circles), or 300 μM O_2 (squares). The lines are from a global fit of the data in Figures 2B, 3C, and 4B to the mechanism in Scheme 8 using the rate constants in Table 2. In A and C, only a portion of the points are shown, and the individual traces are offset for clarity. Conditions as described for Figure 1.

NIH-PA Author Manuscript
NIH-PA Author Manuscript
NIH-PA Author Manuscript

NIH-PA Author Manuscript

NIH-PA Author Manuscript

NIH-PA Author Manuscript

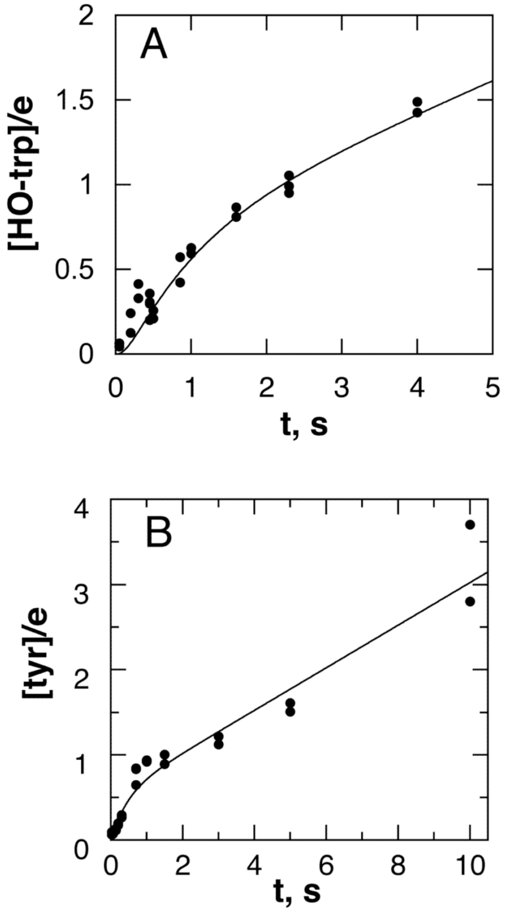

Figure 4.

Chemical quench analyses of the TrpH reaction: A, the reaction of 100 μM TrpH/Fe(II)·1.5 mM $6MePH_4$·1 mM tryptophan and 1.0 mM O_2; B, the reaction of 150 μM TrpH/Fe(II)·1.5 mM $6MePH_4$·1.5 mM phenylalanine and 1.0 mM O_2. The lines are from simulations using the mechanism and rate constants from Scheme 7 and Table 1 in A and Scheme 8 and Table 2 in B. Conditions as described for Figure 1.

NIH-PA Author Manuscript
NIH-PA Author Manuscript
NIH-PA Author Manuscript

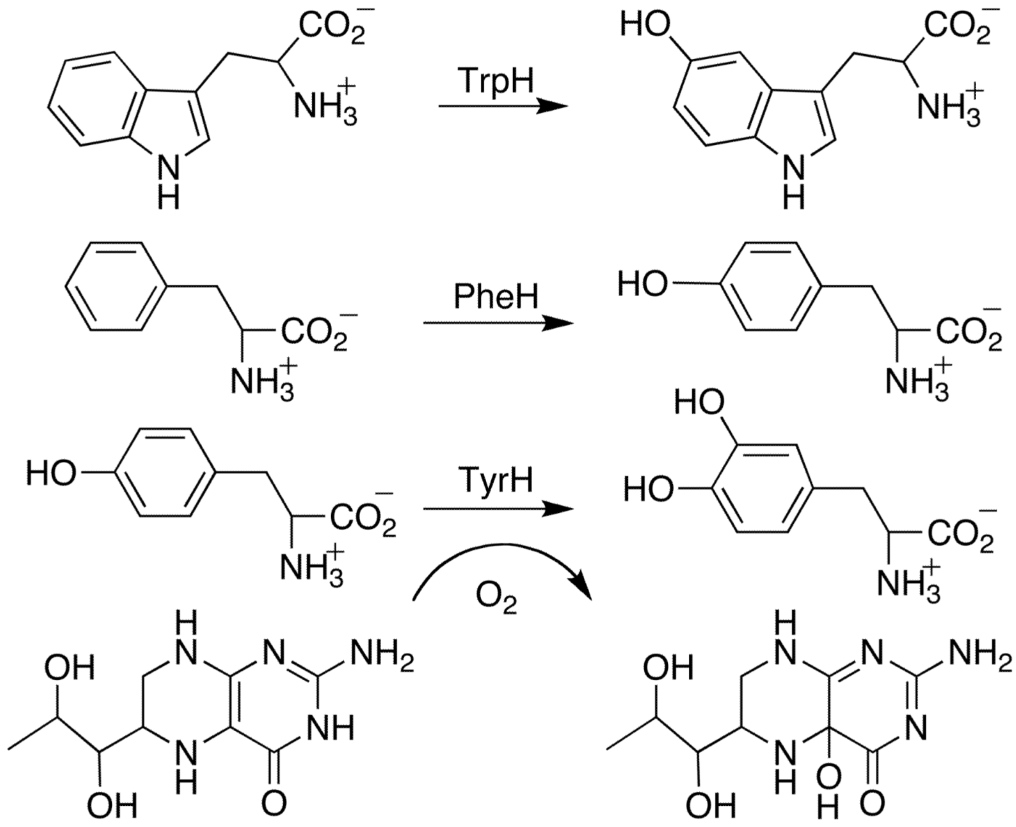

Scheme 1.

NIH-PA Author Manuscript

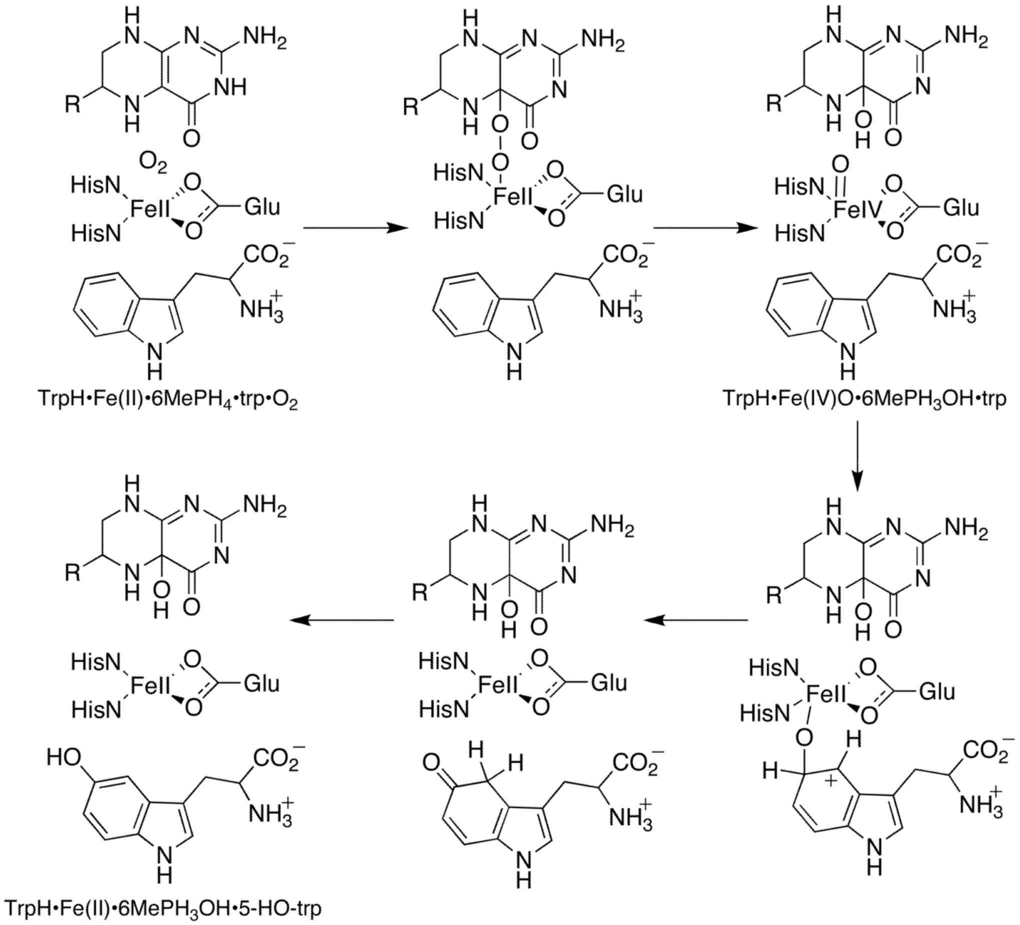

Scheme 2.

NIH-PA Author Manuscript

$$\mathrm{TrpH{\bullet}Fe(II)} + O_2 \xrightarrow{k_{ox}} \mathrm{TrpH{\bullet}Fe(III)}$$

Scheme 3.

NIH-PA Author Manuscript

NIH-PA Author Manuscript

NIH-PA Author Manuscript

NIH-PA Author Manuscript

NIH-PA Author Manuscript

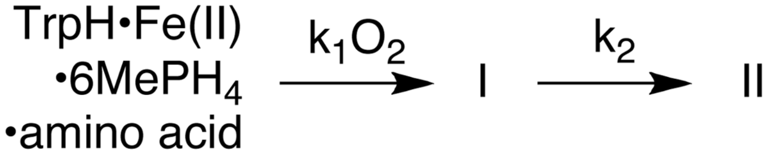

Scheme 4.

NIH-PA Author Manuscript

NIH-PA Author Manuscript

NIH-PA Author Manuscript

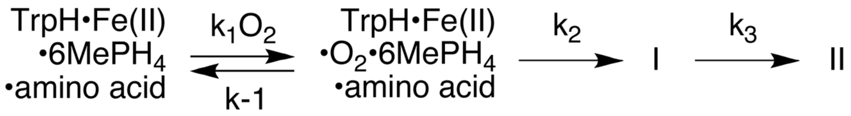

Scheme 5.

NIH-PA Author Manuscript
NIH-PA Author Manuscript
NIH-PA Author Manuscript

$$\begin{array}{c}\text{TrpH}\bullet\text{Fe(II)}\\ +\ O_2 + 6\text{MePH}_4 + \text{trp}\end{array} \xrightarrow{k_{3'}} \begin{array}{l}\text{TrpH}\bullet\text{Fe(II)}\\ \bullet 6\text{MePH}_3\text{OH}\\ \bullet 5\text{-OH-trp}\end{array} \xrightarrow{k_4} \begin{array}{l}\text{TrpH}\bullet\text{Fe(II)}\\ +\ 6\text{MePH}_3\text{OH}\\ +\ 5\text{-HO-trp}\end{array}$$

Scheme 6.

NIH-PA Author Manuscript

NIH-PA Author Manuscript

NIH-PA Author Manuscript

$$\text{TrpH•Fe(II)•6MePH}_4\text{•trp} + \text{O}_2 \overset{K_{O2}}{\rightleftharpoons} \text{TrpH•Fe(II)•6MePH}_4\text{•trp•O}_2$$

$$\text{TrpH•Fe(II)•6MePH}_4\text{•trp•O}_2 \xrightarrow{k_2} \text{TrpH•I•trp}$$

$$\text{TrpH•I•trp} \xrightarrow{k_3} \text{TrpH•Fe(IV)O•6MePH}_3\text{OH•trp}$$

$$\text{TrpH•Fe(IV)O•6MePH}_3\text{OH•trp} \xrightarrow{k_4} \text{TrpH•Fe(II)•6MePH}_3\text{OH•5-HO-trp}$$

$$\text{TrpH•Fe(II)•6MePH}_3\text{OH•5-HO-trp} \xrightarrow{k_5} \text{TrpH•Fe(II)} + \text{6MePH}_3\text{OH} + \text{5-HO-trp}$$

Scheme 7.

NIH-PA Author Manuscript

NIH-PA Author Manuscript

NIH-PA Author Manuscript

$$\text{TrpH•Fe(II)•6MePH}_4\text{•phe} + \text{O}_2 \xrightarrow{k_1} \text{TrpH•I•phe}$$

$$\text{TrpH•I•phe} \xrightarrow{k_2} \text{TrpH•Fe(II)•6MePH}_3\text{OH•tyr}$$

$$\text{TrpH•Fe(II)•6MePH}_3\text{OH•tyr} \xrightarrow{k_3} \text{TrpH•Fe(II)} + \text{6MePH}_3\text{OH} + \text{tyr}$$

Scheme 8.

NIH-PA Author Manuscript
NIH-PA Author Manuscript
NIH-PA Author Manuscript

Table 1

Rate constants from global analysis of tryptophan hydroxylase rapid-reaction kinetics with tryptophan as substrate

kinetic parameter[a]	value[b]	confidence interval[a]
K_{O2}, μM^{-1}	37	36 – 39
k_2, s	65	58 – 74
k_3, s^{-1}	4.4	4.1 – 4.8
k_4, s^{-1}	1.3	1.1 – 1.5
k_5, s^{-1}	0.21	0.20 – 0.23

[a] See Scheme 7.

[b] Confidence intervals calculated using the FitSpace option in KinTek Explorer (36,37).

NIH-PA Author Manuscript

NIH-PA Author Manuscript

NIH-PA Author Manuscript

Table 2

Rate constants from global analysis of tryptophan hydroxylase rapid-reaction kinetics with phenylalanine as substrate

kinetic parameter[a]	value[b]	confidence interval[a]
k_1, $\mu M^{-1}s^{-1}$	0.281	0.23 – 0.38
k_2, s^{-1}	1.8	1.4 – 2.7
k_3, s^{-1}	0.39	0.34 – 0.43

[a]See Scheme 8.

[b]Confidence intervals calculated using the FitSpace option in KinTek Explorer (36,37).
